# Probing Antibacterial and Anticancer Potential of *Selenicereus undatus*, *Pistacia vera* L. and *Olea europaea* L. against Uropathogens, MCF-7 and A2780 Cancer Cells

**DOI:** 10.3390/molecules28248148

**Published:** 2023-12-18

**Authors:** Sahar Safdar, Saba Shamim, Maryam Khan, Ali Imran, Mudassar Ali Khan, Qurban Ali, Shiming Han

**Affiliations:** 1 School of Biological Sciences and Technology, Liupanshui Normal University, Liupanshui 553004, China; drsaharmphil@gmail.com; 2 Institute of Molecular Biology and Biotechnology (IMBB), The University of Lahore, Lahore 54000, Pakistan; maryamkkhan246@gmail.com (M.K.); aliimran1232002@gmail.com (A.I.); 3 Department of Physiology, Rashid Latif Medical College, Lahore 54000, Pakistan; mudassar.ali@rlmc.edu.pk; 4 Department of Plant Breeding and Genetics, Faculty of Agricultural Sciences, University of the Punjab, Lahore 54590, Pakistan; qurbanali.pbg@pu.edu.pk

**Keywords:** UTI, *Selenicereus undatus*, *Pistacia vera*, *Olea europaea*, lipoteichoic acid, DNA gyrase, MCF-7 cells, A2780 cells

## Abstract

Urinary tract infection is an infectious disease that requires immediate treatment. It can occur in any age group and involves both genders equally. The present study was to check the resistance of some antibiotics and to assess the antibacterial potential of three extracts of three plants against notorious bacteria involved in urinary tract infections. Along with assessing the antibacterial activity of plant extracts, we checked for the anticancer potential of these extracts against the cancer cell lines MCF-7 and A2780. Cancer is the leading cause of mortality in developed countries. Determinations of total flavonoid content, total phenolic content, total alkaloid content, total tannin content, total carotenoid content, and total steroid content were performed. The disk diffusion method was used to analyze the antibacterial activity of plant extracts. Ethanolic extract of *Selenicereus undatus* showed sensitivity (25–28 mm) against bacteria, whereas chloroform and hexane extracts showed resistance against all bacteria except *Staphylococcus* (25 mm). Ethanolic extract of *Pistacia vera* L. showed sensitivity (22–25 mm) against bacteria, whereas chloroform and hexane extracts showed resistance. Ethanolic extract of *Olea europaea* L. showed sensitivity (8–16 mm) against all bacteria except *Staphylococcus*, whereas chloroform and hexane extracts showed resistance. Positive controls showed variable zones of inhibition (2–60 mm), and negative control showed 0–1 mm. The antibiotic resistance was much more prominent in the case of hexane and chloroform extracts of all plants, whereas ethanolic extract showed a sensitivity of bacteria against extracts. Both cell lines, MCF-7 and A2780, displayed decreased live cells when treated with plant extracts.

## 1. Introduction

*Selenicereus undatus* (Haw.) D.R. Hunt, a dragon fruit, is a flowering plant that belongs to the genus *Hylocereus*, which contains many species used in traditional medicines. It is native to Mexico and Central America, but has now spread to South Africa, South America, Asia and the Caribbean region. The health-promoting potential of pitaya fruit is due to the presence of bioactive compounds related to numerous benefits, such as antidiabetic, anti-inflammatory, antioxidant, anticancer, and antimicrobial. *Pistacia vera* L., native to mountainous regions of Iran, Syria, Kyrgyzstan, Turkmenistan, Turkey, Greece and west Afghanistan, has also been used in Chinese and Uyghur medicine for treatment of skin diseases, hemorrhage, diarrhea, and many other human ailments [1]. *Olea europaea* L. is regarded as the Olea genus’s best-known component. It is native to Asia, Africa and the European Mediterranean. It contains several bioactive compounds and is beneficial in many ailments, such as blood pressure, uric acid, cholesterol, and glycemia reduction. It also showed neuroprotective, vasodilator, antirheumatic, antidiarrheal, and anti-inflammatory properties. It could be a food supplement to improve human health [2].

Urinary tract infections can involve the prostate, urethra, bladder, and kidneys and are major bacterial disorders [3]. Bacteria produce infections in the urinary tract when they reach the tract via the urethral opening. These infections are more common in developing countries due to less hygienic factors [4]. Urinary tract infections are named differently in different organs: pyelonephritis is an infection of the kidneys and cystitis of the bladder [5]. The urinary tract infection rate is increasing annually [4]. These infections are the second-most common cause of sepsis in hospitals and account for more than one third. Causative pathogenic organisms cause these infections, which may reside in hospitals or in some communities. *Serratia*, *Enterococcus*, *Enterobacter*, *Pseudomonas aeruginosa*, *Proteus*, and *Escherichia coli* are included in the list of hospital-acquired pathogens. The list of community-acquired pathogens includes *Staphylococcus saprophyticus*, *Enterococculs faecalis*, *Proteus mirabilis*, *Klebsiella pneumoniae*, and *E. coli* [6]. These pathogens generally affect the whole population, with some more susceptible than others.

Prognosis of disease solely depends on extensive antibiotic therapy, hospital admission, catheterization time, gender, and age [7]. Nosocomial infections in addition to pyuria, dysuria, abdominal pain, painful urination, back pain, and irregular urination are some of the signs that may be present in patients at the time of infection [8]. These clinical signs depend on the degree of infection, age, immune state of patients, and uropathogens [9]. Culture techniques and microscopy are also helpful in diagnosis and treatment, including broad-spectrum antibiotics [10], although misuse of antibiotics is a major reason for resistance to them worldwide [11]. However, in terms of resistance patterns and demographic information, surveillance studies are very helpful in choosing the best antibiotics for treatment. Some pathogens resist certain antibiotics and some do not, as mentioned in previous studies [11,12,13]. This resistance power of bacteria creates the greatest hurdle in treating bacterial infections and affects societies economically and socially. Sometimes, serious complications arise due to resistance of bacteria towards antibiotics [14,15]. To prevent multidrug resistance in society, the World Health Organization has implemented many intervention measures, including designing baselines to coordinate the surveillance of resistance in pathogens, formulating indicators to evaluate and review the effect of resistance, and formulating a task force for this responsibility [16].

Due to limited treatment options and resources, these interventions were found ineffective in developing countries compared to developed countries. Increased antibiotic resistance was found in South Asian regions, including Pakistan, as mentioned in some studies [17]. This study determined the prevalence and antibiotic sensitivity profiles of uropathogens. Herbal medicines are very popular worldwide for their few side effects, and they contain more than 70 compounds that are proven to be antitumoral. Cancer is defined by uncontrolled cell divisions [18]. MCF-7 and A2780 are commonly studied cell lines in breast cancer [19] and ovarian-derived cancer [20] to evaluate anticancer activity in in vitro conditions.

The aims of this study included isolation and molecular identification of uropathogens, in vitro analysis of medicinal plants based on anti-uropathogenic activity, determination of anti-uropathogenic phytoconstituents via GCMS, in vitro analysis of the selected medicinal plants on the human breast cancer cell line MCF-7 and ovarian cancer cell line A2780 and in silico studies deciphering the anti-uropathogenic role of the plants’ bioactive agents against the potential target sites of the isolated and characterized uropathogens.

## 2. Results

### 2.1. Isolation and Molecular Characterization of Uropathogens

**Figure 1:**
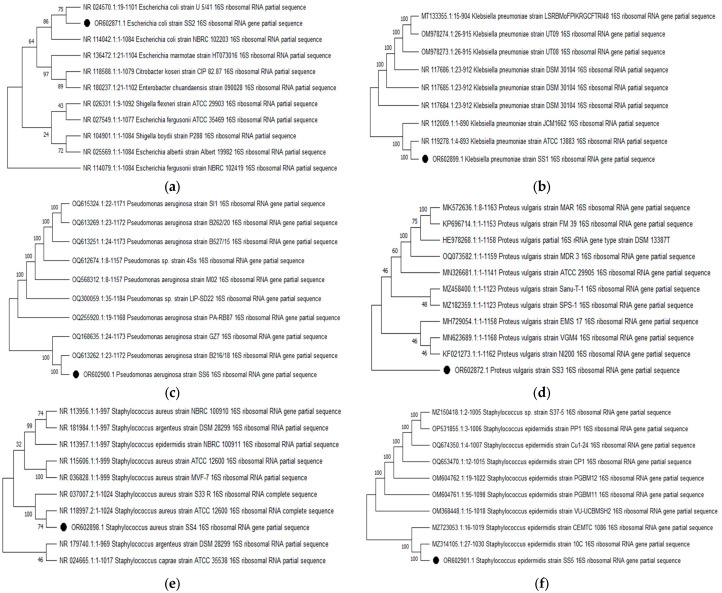
Maximum-likelihood tree of (**a**) *E. coli* OR602871, (**b**) *K. pneumoniae* OR602899, (**c**) *P. aeruginosa* OR602900, (**d**) *P. vulgaris* OR602872, (**e**) *S. aureus* OR602898, and (**f**) *S. epidermidis* OR602901 using neighbor-joining method. Bootstrap values are expressed as a frequency of 1000 replicates, and values less than 50% are not shown. Black dots are showing the bacterial strains isolated, characterized and studied in the current study.

Out of 2697 urine samples collected, 264 (8.89%) samples (males = 84, females = 180) were uropathogen-positive cultures. The 16s rRNA sequences were submitted to the NCBI GenBank to obtain the accession numbers in Table 1. Their antibiograms are given in Table 2 and Table 3. Figure 1a–f demonstrates the phylogenetic trees of the various bacterial strains, illustrating the comparison and closest strains among the homologous sequences obtained from NCBI GenBank.

### 2.2. Quantitative Analysis of S. undatus, P. vera and O. europaea Extracts

The quantitative analysis of ethanol, hexane and chloroform extracts of *S. undatus*, *P. vera*, and *O. europaea* is shown in Table 4.

### 2.3. GC-MS of S. undatus

GC-MS analysis of *S. undatus* is given in Table 5 below. Its phytoconstituents possessing antimicrobial activities are mentioned in bold.

### 2.4. GC-MS of P. vera

GC-MS analysis of *P. vera* is given in Table 6. Its phytoconstituents possessing antimicrobial activities are shown in bold.

### 2.5. GCMS of O. europaea

GCMS analysis of *O. europaea* is given in Table 7. Its phytoconstituents possessing antimicrobial activities are shown in bold.

### 2.6. Antibacterial Sensitivity of S. undatus, P. vera and O. europaea

**Figure 2:**
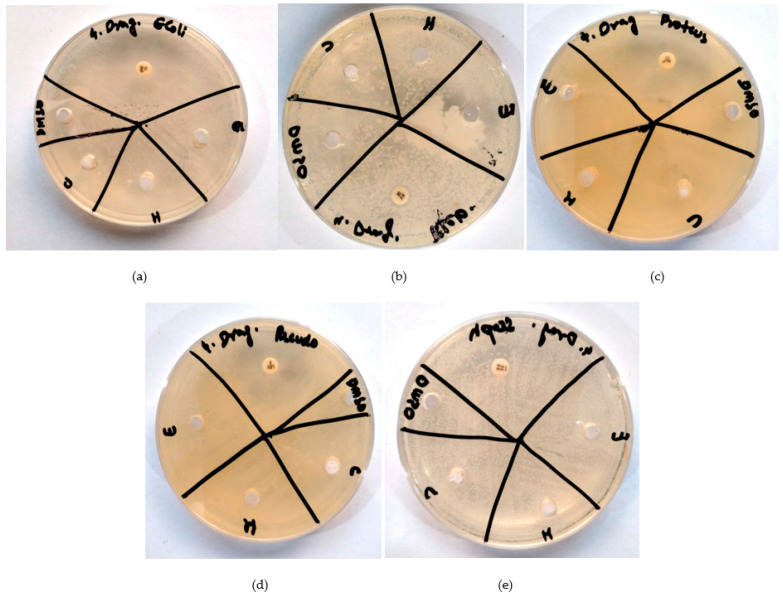
Antibiograms of *S. undatus* (E = extract in ethanol, H = extract in hexane, C = extract in chloroform, DMSO = negative control. (**a**): Against *E. coli*, imipenem as positive control. (**b**): Against *K. pneumoniae*; vancomycin as positive control. (**c**): Against *P. vulgaris*; ciprofloxacin as positive control. (**d**): Against *P. aeruginosa*; levofloxacin as positive control. (**e**): Against *S. aureus*; amikacin as positive control.

**Figure 3:**
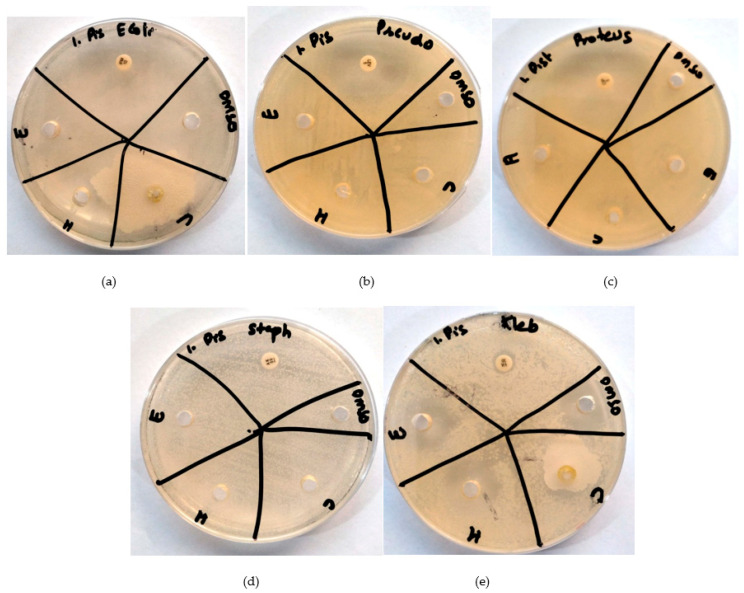
Antibiograms of *P. vera* (E = extract in ethanol, H = extract in hexane, C = extract in chloroform, DMSO = negative control (**a**): against *E. coli*; imipenem as positive control. (**b**): Against *K. pneumonia*; vancomycin as positive control. (**c**): Against *P. vulgaris*; ciprofloxacin as positive control. (**d**): Against *P. aeruginosa*; levofloxacin as positive control. (**e**): Against *S. aureus*; amikacin as positive control).

Each bacterial strain (*E. coli*, *K. pneumoniae*, *P. vulgaris*, *P. aeruginosa*, *S. aureus*, and *S. epidermidis*) was tested against *S. undatus*, *P. vera* and *O. europaea* ethanol, chloroform, and hexane extracts as well as six antibiotics, i.e., imipenem, vancomycin, ciprofloxacin, and levofloxacin (Figure 1, Figure 2 and Figure 3). Amikacin was used as a positive control and DMSO as a negative control. S shows the sensitivity of antibiotics against specific bacteria, whereas R shows the percentage of drug resistance (Table 8a–c).

Figure 2a shows an antibiogram of three extracts of *S. undatus* with imipenem as positive control against *E. coli*. Figure 2b shows an antibiogram of three extracts of *H. undatus* with vancomycin as positive control against *K. pneumoniae*. Figure 2c shows an antibiogram of three extracts of *S. undatus* with ciprofloxacin as positive control against *P. vulgaris*. Figure 2d shows an antibiogram of three extracts of *S. undatus* with levofloxacin as a positive control against *P. aeruginosa*. Figure 2e shows an antibiogram of three extracts of *S. undatus* with amikacin as positive control against *S. aureus*.

Figure 3a shows an antibiogram of three *P. vera* extracts with imipenem as positive control against *E. coli*. Figure 3b shows an antibiogram of three *P. vera* extracts with vancomycin as positive control against *K. pneumoniae*. Figure 3c shows an antibiogram of three *P. vera* extracts with ciprofloxacin as positive control against *P. vulgaris*. Figure 3d shows an antibiogram of three *P. vera* extracts with levofloxacin as positive control against *P. aeruginosa*. Figure 3e shows an antibiogram of three *P. vera* extracts with amikacin as positive control against *S. aureus*.

**Figure 4:**
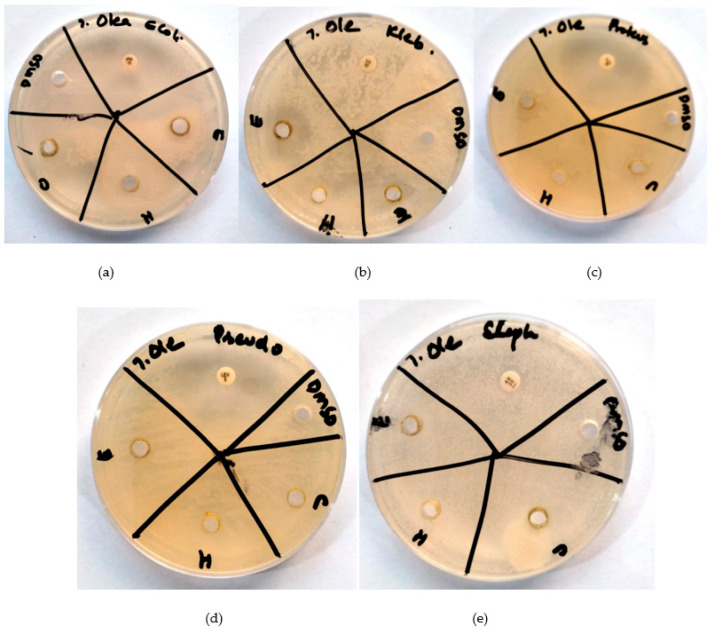
Antibiogram of *O. europaea* (E = extract in ethanol, H = extract in hexane, C = extract in chloroform, DMSO = negative control. (**a**): Against *E. coli*; imipenem as positive control. (**b**): Against *K. pneumoniae*; vancomycin as positive control. (**c**): Against *P. vulgaris*; ciprofloxacin as positive control. (**d**): Against *P. aeruginosa*; levofloxacin as positive control. (**e**): Against *S. aureus*; amikacin as positive control).

Figure 4a shows an antibiogram of three *O. europaea* extracts with imipenem as positive control against *E. coli*. Figure 4b shows an antibiogram of three *O. europaea* extracts with vancomycin as positive control against *K. pneumoniae*. Figure 4c shows an antibiogram of three *O. europaea* extracts with ciprofloxacin as positive control against *P. vulgaris* (d) against *P. aeruginosa*. Figure 4e shows an antibiogram of three *O. europaea* extracts with amikacin as positive control against *S. aureus*.

### 2.7. Effect of H. undatus, P. vera and O. europaea on MCF-7 Cells (Breast Cancer Cell Line)

**Figure 5:**
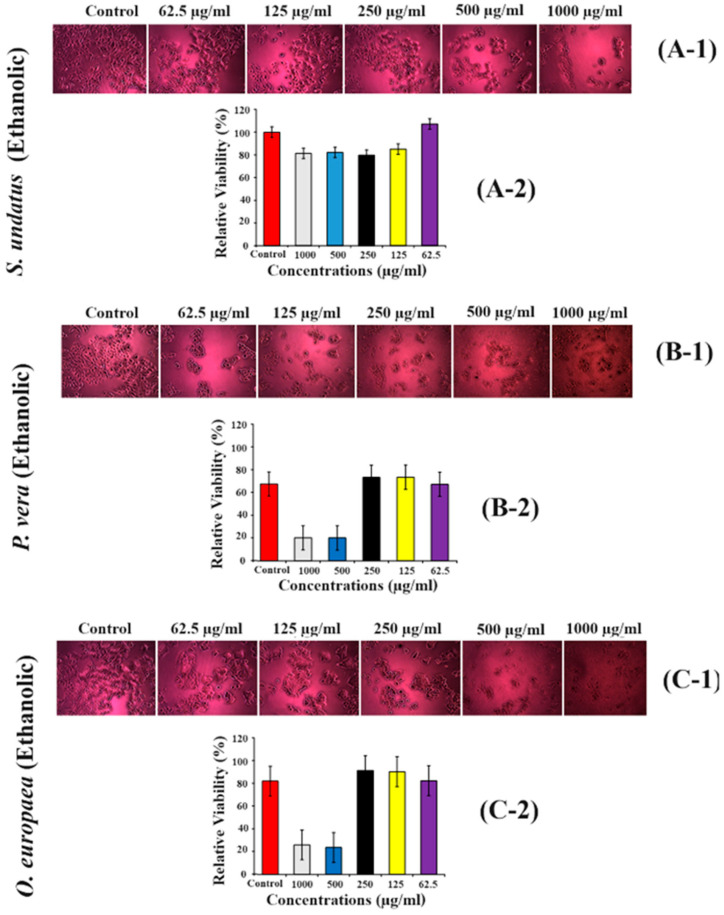
Cytotoxicity assays of *S. undatus* (**A-1**,**A-2**), *P. vera* (**B-1**,**B-2**) and *O. europaea* (**C-1**,**C-2**) extracts on MCF-7 cell line using MTT assay treated with various concentrations (62.5 µg/mL, 125 µg/mL, 250 µg/mL, 500 µg/mL, and 1000 µg/mL).

To determine the effect of ethanolic extract of *S. undatus* on cell survival of the MCF-7 cell line, 62.5 µg/mL, 125 µg/mL, 250 µg/mL, 500 µg/mL, and 1000 µg/mL extract concentrations were used, and cell viability was checked via MTT assay. Several cells were observed dead after treatment compared to the control. Lower concentrations were found to be more efficacious than higher concentrations, but the difference was not obvious (Figure 5(A-1,A-2)), whereas there was a clear difference between lower and higher concentrations (Figure 5(B-1,B-2,C-1,C-2)).

### 2.8. Effect of S. undatus, P. vera and O. europaea on A2780 Cells (Ovarian Cancer Cell Line)

**Figure 6:**
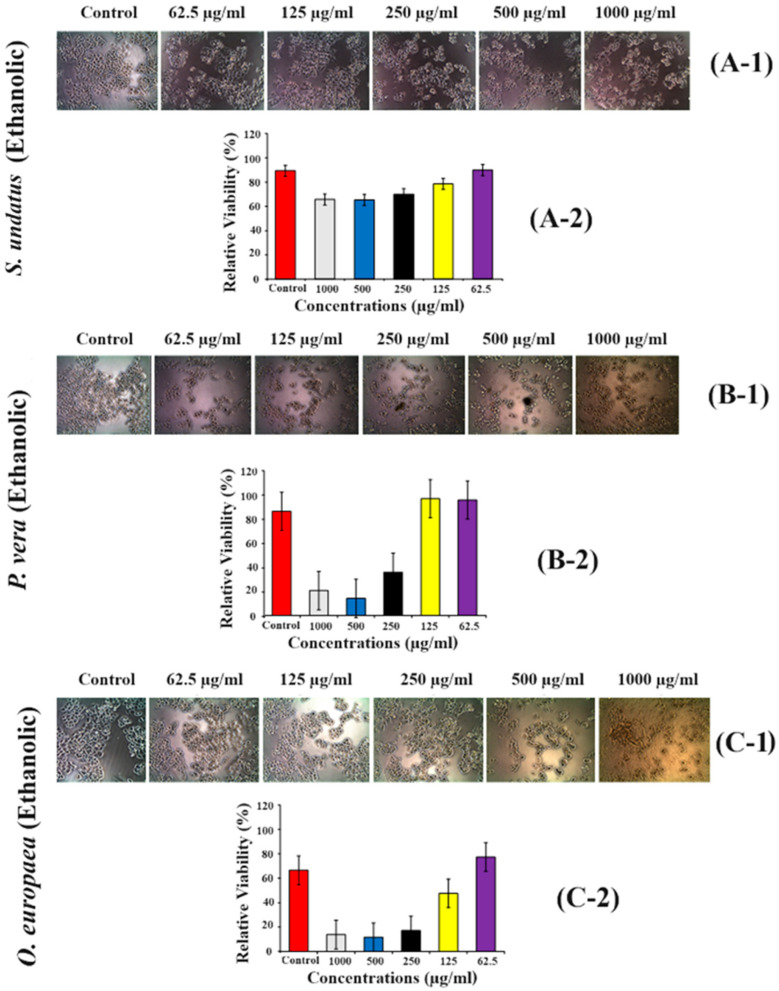
(**A-1**,**A-2**) Cytotoxicity assay of *S. undatus* (**A-1**,**A-2**), *P. vera* (**B-1**,**B-2**), and *O. europaea* (**C-1**,**C-2**) extraction in A2780 cell line using MTT assay treated with various concentrations (62.5 µg/mL, 125 µg/mL, 250 µg/mL, 500 µg/mL, and 1000 µg/mL.

To determine the effect of ethanolic extract of *S. undatus* on cell survival of the A2780 cell line, 62.5 µg/mL, 125 µg/mL, 250 µg/mL, 500 µg/mL, and 1000 µg/mL extract concentrations were used and cell viability was checked via MTT assay. Several cells were observed dead after treatment compared to the control. Lower concentrations were found to be more efficacious than higher concentrations. Still, the difference was not obvious (Figure 6(A-1,A-2)), whereas there was a clear difference between lower and higher concentrations (Figure 6(B-1,B-2,C-1,C-2)).

### 2.9. Molecular Docking Studies

The molecular docking results using lipoteichoic acid and DNA gyrase are shown in Figures S1 and S2 and are provided in the Supplementary Materials.

## 3. Discussion

Urinary tract infections pose a significant threat to the health-care system due to their increasing prevalence in hospitals and communities [21]. There is an urgent need for continuous surveillance of resistance patterns among pathogens responsible for urinary tract infections in specific affected areas. In this five-year study, *E. coli*, *K. pneumoniae*, *P. vulgaris*, *P. aeruginosa*, *S. aureus*, and *S. epidermidis* were the major uropathogens found (Table 1; Figure 1). This is crucial for accurately identifying uropathogens and selecting appropriate antibiotic treatments [22]. Our study has yielded insights into the antibacterial resistance patterns of these uropathogens (Table 2 and Table 3).

Through quantitative analysis of plant extracts, we have determined that flavonoids are present in the highest concentrations across all plant extracts (Table 4), followed by tannins, carotenoids, alkaloids, steroids, and phenols, which accords with the study of Guettaf et al. [23]. GC-MS analyses of all three plants showed the presence of antibacterial phytoconstituents (Table 5, Table 6 and Table 7).

The ethanolic extract of *H. undatus* demonstrated sensitivity to bacteria, while the chloroform and hexane extracts exhibited resistance (Table 8). Specifically, we observed 28 mm sensitivity to *S. undatus* against *E. coli*, 25 mm sensitivity against *K. pneumoniae*, 25 mm sensitivity against *P. vulgaris*, 28 mm sensitivity against *P. aeruginosa*, and 25 mm sensitivity against *S. aureus*. All bacteria resisted the hexane and chloroform extracts of *S. undatus*, except in the case of *S. aureus* (Figure 2a–e). These results were contrary to the study by Nurmahani et al. [24], which showed the highest sensitivity of bacteria to chloroform extract of *S. undatus* peel, followed by hexane and ethanol extracts.

Similarly, the ethanolic extract of *P. vera* displayed sensitivity to bacteria, while the chloroform and hexane extracts showed resistance. Notably, we observed 28 mm sensitivity of *P. vera* against *E. coli*, 25 mm sensitivity against *K. pneumoniae*, 22 mm sensitivity against *P. vulgaris*, 25 mm sensitivity against *P. aeruginosa*, and 22 mm sensitivity against *S. aureus*. All bacteria resisted the hexane and chloroform extracts of *P. vera* (Figure 3a–e). A study by Shirzadi-Ahodashti et al. [25] demonstrated the sensitivity of Gram-negative bacteria against *P. vera* hull, which is as per our study. All bacteria exhibited resistance to the hexane and chloroform extracts of *P. vera*.

In contrast, the ethanolic extract of *O. europaea* exhibited sensitivity to bacteria, while the chloroform and hexane extracts showed resistance. Specifically, we observed 15 mm sensitivity of *O. europaea* against *E. coli*, 16 mm sensitivity against *K. pneumoniae*, 8 mm sensitivity against *P. vulgaris*, and 15 mm sensitivity against *P. aeruginosa*. *O. europaea* demonstrated resistance against *S. aureus*. All bacteria resisted the hexane and chloroform extracts of *O. europaea* (Figure 4a–e). *O. europaea* demonstrated resistance against *Staphylococcus*, contrary to the study by Ben-Amor et al. [26], which showed anti-staphylococcal activity of *O. europaea* leaf extract. Our results were similar to the study by Sahraoui et al. [27].

Moreover, ethanolic extracts of *S. undatus*, *P. vera*, and *O. europaea* had cytotoxic effects on both MCF-7 breast cancer cells (Figure 5) and A2780 ovarian cancer cells (Figure 6). The observed dose-dependent responses indicate that the extracts may contain bioactive compounds with potential anticancer properties [28]. Natural medicines are considered safer than artificial drugs. These medicines contain specific metabolites that have proven effective in treating various diseases under both abiotic and biotic conditions. These metabolites can serve as therapeutic agents, such as apoptotic inducers and tumor suppressors in cancer cells [18]. Our current research has investigated the anticancer potential of plants, particularly at lower concentrations. According to our findings, treatment with plant extracts reduced cell viability in post-treated breast carcinoma and ovarian cancer cells.

The bioinformatics analysis explains molecular docking results for various compounds and their interactions with specific proteins or enzymes. The molecular docking results using bacterial lipoteichoic acid, DNA gyrase are shown in Figures S1 and S2. Bacterial lipoteichoic acid and DNA gyrase were chosen as potential targets for ascertaining the antibacterial potential of medicinal plants under investigation. Molecular docking studies were performed to check whether the phytocomponents present in the plants used in this study can be used as antibacterial drugs as per previous researchers [29,30]. Bacterial lipoteichoic acid [31,32,33,34,35] and DNA gyrase [36,37,38] were potential antibacterial targets. The docking of lipoteichoic acid receptor S domain eLTAS (2w5q) with phytocompounds was performed (Figure S1). The molecular docking studies of eugenol showed inhibitory activity compared to the standard. The enzyme docking in complex with inhibitor eugenol showed initial conformations with several poses of binding free energy. Eugenol, oleuropein, furylhydroxymethyl ketone, and levoglucosan were docked inside the binding pocket to determine their interactions with *S. aureus* LtaS. Receptor (PDB: 2w5q). The docking analysis suggested that OH group of eugenol inside the pocket revealed various interactions with amino acids, such as PHEA353, LEUA384, ASPA349, TRPA354, and HISA416. These amino acids LEUA384 and PHEA353 were linked through π-alkyl and alkyl linkages. TRPA354 and ASPA349 formed conventional hydrogen bonds with receptors (Figure S1a). The same interactions were determined for oleuropein (Figure S1b). However, the interactions of furyl were not considered informative due to unfavorable bumps in the docking analysis. The interactions of levoglucosan showed interactions with ARGA356 and TRPA3534 through hydrogen bonds and conventional hydrogen bonds (Figure S1c). It was further seen that dihydroactinidiolide, stigmasterol, and a-methyl-a-[4-methyl-3-pentenyl] oxirane methanol showed no poses with 2w5q. The docked molecules of luteolin showed interactions of amino acids HISA416, GLUA255, THRA300, and TRPA354 with phenolic groups (Figure S1d). All these amino acids were bound to the ligand through forming water, conventional, and hydrogen bonding. Similarly, N-propylacetamide (Figure S1e) and oleuropein (Figure S1f) showed close interactions with amino acids, such as ARG356 and TRP354, through linking with hydrogen bonds. Phenylacetaldehyde was docked inside the catalytic domain of the protein. TRPA353 showed conventional bonds with the oxygen atoms of benzaldehyde, and ASPA34 interacted through carbon-hydrogen bonding. π-Alkyl and Pi-alkyl interactions were seen with PHEA353 and LEUA384 enzyme residues (Figure S1g). However, the docking analysis of thymol showed interactions between the oxygen atoms of aromatic compound docked deeply within the active side with TRPA354 enzyme residues by π–π stacked interactions. Alkyl and π-alkyl interactions were formed by the aromatic ring containing oxygen with LEUA384, LEUA431, PHEA353 and HISA416 (Figure S1g) and thymol (Figure S1h). In addition, 1-oxaspiro [2.5]octane, 5,5-dimethyl-4-(3-methyl-1,3-butadienyl) showed interactions with ARGA:356 and TRPA:354 through conventional hydrogen bonding (Figure S1i). However, melezitose and oleuropein did not show any poses with 2w5q.

Docking analysis of DNA gyrase (6rku) with phytocompounds was performed (Figure S2). The phenolic group of thymol was found to be linked to the nucleotides, such as DAG:17, DAH:17, and DTH:16. DAG17 and DTH:16 deeply interacted through π–π stacked, and DAH:17 was attached to the OH of benzene ring through conventional hydrogen bonding (Figure S2a). However, the oxygen of stigmasterol was linked to amino acid residue META:120 through a conventional hydrogen bond. Another amino acid residue, ALAA:119, was seen as deeply incorporated into the benzene ring. The nucleotides DTH:16. DTG:16, DAH:17, and DAG:16 were linked to the benzene ring through alkyl π-alkyl interactions (Figure S2b).

The phenol group of melezitose showed interaction with amino acid residue ASPC:82 linked through conventional hydrogen bonds. In addition, the amino acid residue META:120 and nucleotides DAG:17, DAH:17. DTG:16 and DTH:16 interacted via alkyl and π-alkyl linkage (Figure S2c). The benzaldehyde group of phenylacetaldehyde interacted with DAH:17, DAH16, and DTG:16 through conventional hydrogen bonds and π–π stacking (Figure S2d). Furthermore, the oxygen of 1-oxaspiro [2.5]octane, 5,5-dimethyl-4-(3-methyl-1,3-butadienyl) showed interactions with nucleotides DTG:16, DAG:17 and DAH:17 through π-alkyl linkage (Figure S2e). However, oleuropein showed unfavorable acceptor–acceptor bumps, and its results were not considered.

*O. europaea* showed interactions with amino acid residues and nucleotides. ALAA:119 and META:120 interacted with the aromatic ring and oxygen through conventional hydrogen bonding, alkyl, and π-alkyl. However, DAG:17, DRG:16. DAH:17, and DTH:16 interacted with benzene rings of *O. europaea* by alkyl and π-alkyl linkage (Figure S2f). The π-alkyl interactions were seen in N-propyl acetamide and nucleotides DAH:17, DTH:16, and DAG:17 (Figure S2g). The phenolic groups of luteolin interacted with nucleotides DTH:16, DAG:17, DAH:17, and DTG:16 through conventional hydrogen bonds and π–π stacked linkages. These nucleotides showed strong interactions, and low affinities were observed (Figure S2h).

The OH group of levoglucosan interacted with DAH:17 and DTH:16 through conventional hydrogen bonding (Figure S2i). Furylhyrdoxymethyl ketone’s furan ring interacted with nucleotides DCG:18 and DAG:17 through conventional hydrogen bonds (Figure S2j). Eugenol interacted with various nucleotides through different interactions. The docking analysis of eugenol with 6rku suggested that the OH group of the benzene ring attached to DAG:17 through conventional hydrogen bond interactions, whereas DTG:16 showed π–π stacking. In addition, it was seen that DTH:16 and DAH:17 exhibited π-alkyl and carbon–hydrogen bonding interactions with the aromatic ring of eugenol (Figure S2k). However, no affinity and interactions were seen between dihydroactinidiolide and 6rku. The OH groups of -methyl-a-[4-methyl-3-pentenyl] oxiranemethanol interacted with DAH:17 through conventional hydrogen bonding, while DAG:17, DTG:16, and DTH:16 showed π-alkyl interactions (Figure S2l).

The docking analysis of 5eix with all the ligands showed unfavorable bumps due to different clashes between amino acid residues and ligands. All these unfavorable bumps were not considered appropriate to report, and these results were excluded. However, 3e2k showed no pose generation with the ligand, so no docking analysis was performed. The outcomes of these docking studies can provide insights into the potential therapeutic or inhibitory effects of these compounds on the target proteins, which may have implications for drug development and understanding of biological mechanisms. However, it is important to note that successful molecular docking does not guarantee biological activity, and further experimental validation is typically required to confirm the findings.

## 4. Materials and Methods

### 4.1. Collection of Samples, Isolation and Characterization of Uropathogens

A total of 2967 urine samples were collected from September 2016 to December 2021. The samples were collected after the informed consent of the patient. The patients who submitted their complete information (age, gender, occupation, bacteria isolated, culture sensitivity) were included in this study and otherwise excluded. The uropathogens were isolated from the samples by standard microbiological techniques. The bacterial isolates were characterized based on their colonies, morphology, and biochemical profiles, as 16s rRNA sequencing. The nucleotides thus obtained were submitted to BLASTn (https://blast.ncbi.nlm.nih.gov/) (for analysis), NCBI GenBank (for homology), Clustal W in MEGA11 software (MEGA11 version 11.0.13) (for pairwise alignment), and finally phylogeny (neighbor-joining tree method along with 1000 bootstrap replicates) was established [39,40].

### 4.2. Preparation of S. undatus, P. vera and O. europaea Extracts

*S. undatus* fruits were purchased from China, whereas the other two plants (fresh fruits) were collected fresh from local farmlands with geographical coordinates of *P. vera* from district Okara having 30.65° latitude, 74.031° longitude; *O. europaea* from Chakwal having 72° longitude, 32° latitude, 575 m altitude. The collected fruits were dried in shadow. The conventional Soxhlet extraction method was used to prepare the plant extract in three different solvents (ethanol, chloroform, and hexane) in ratio (9:1) (solvent: plant material) using round-bottom flasks. It was placed at 37 °C at 150 rpm for 18–24 h. Next day, it was filtered and concentrated to 20% *w*/*v* on rotary evaporator followed by its drying on a lyophilizer. The dried extracts were dissolved in 10% DMSO [41] and refrigerated for future experiments [42].

### 4.3. Quantitative Estimation of S. undatus, P. vera, and O. europaea Extracts

For each test, crude plant extract (ethanol) was used as 1 mg mL^−1^.

#### 4.3.1. Total Phenolic Content

Baba and Malik’s [43] method was used to ascertain this. Crude extract was prepared by adding 200 µL crude extract, 2 mL distilled water, and 500 µL Folin–Ciocalteu reagent in a vial and mix by pipetting in and out for about 1 min and allowing the mixture to mix at room temperature for about 3 min. After adding 2 mL 20% sodium carbonate, the vial was placed away from light for 1 h, and absorbance was observed at 650 nm. Gallic acid (100 µg mL^−1^) was used as standard. The results were expressed by gallic acid equivalent (GAE) per gram of each plant.

#### 4.3.2. Total Flavonoid Content

For this, in 5 mL volume, 50 µL was constituted by crude extract, in addition to 300 µL sodium nitrate, 1 mL methanol, and 4 mL distilled water. After 5 min, 300 µL 10% aluminum chloride was followed by a further 10 min incubation. At the end, it was supplemented with 2 mL NaOH, and the remaining volume was filled with distilled water up to 10 mL. It was rested at room temperature for 15–20 min. The optical density was recorded at 510 nm. The results are expressed as mg rutin equivalent/g dry weight [43].

#### 4.3.3. Total Tannin Content

Total tannin content was ascertained by adding distilled water (750 µL), Folin–Ciocalteu reagent (500 µL), 35% Na_2_CO_3_ (1000 µL), 100 µL plant extract, and 7650 µL distilled water. As control, plant extract was substituted by water. This mixture was placed at room temperature for half an hour before taking its optical density at 725 nm. The gallic acid was expressed using a standard curve. The results are expressed as GAE/g dry matter [44].

#### 4.3.4. Total Alkaloid Content

This was determined by chloroform extraction. For this, 1000 µL of plant extract was dissolved in a few drops of HCl (2N) followed by its extraction by vigorous mixing using a separating funnel (1:1 phosphate buffer–bromocresol solution). The mixture obtained was diluted with chloroform, and optical density was noted at 470 nm [45].

#### 4.3.5. Total Carotenoid Content

Total carotenoid content was ascertained by Thaipong’s method by following the method of Fitriansyah et al. [46] with slight modifications. The extract was dissolved in *n*-hexane, and absorbance was observed at 470 nm. Beta-carotene was used as a carotenoid standard. The total carotenoid content is expressed as beta-carotene equivalent per 100 g of extract (gBE 100g ^−1^).

#### 4.3.6. Total Steroid Content

This was estimated as per Savithramma et al. [47] with slight modifications. Plant extract (1 mL) was dissolved in 1:1 ratio of chloroform and sulfuric acid (10 mL each). Presence of steroids was confirmed by the appearance of a red layer (upper) and yellow with green fluorescence (below the upper layer).

### 4.4. Gas Chromatography–Mass Spectroscopy of Extracts

The previously mentioned method analyzed GC-MS analyses of plant extracts (ethanol) [48]. The GC-MS analysis was performed using an Agilent GCMS 5975 (Agilent Technologies Inc., Santa Clara, CA, USA) C gas chromatograph (GC 7890 A) and mass spectrometer (MS 5975 C). It was equipped with a capillary column (HP-5MS) (30 m × 250 µm × 0.25 µm). Helium gas was the inert gas (carrier) in the column, with a flow rate of 0.8 mL/min (pressure 5.8112 psi, Average velocity 32.756 cm/s, holdup time 1.526 min). The sample was injected manually. The size of the sample was 1 µL. The temperature of the oven was programmed from 5 °C min^−1^ to 70 °C min^−1^, and 10 °C min^−1^ to 300 °C min^−1^, at 240 °C with a hold for 4 min. MS ionic source and interface were regulated at 240 °C, and 200 °C, respectively. Mass scan range of low and high mass was 30–700 *m*/*z*, with a solvent delay of 4 min. Total run time of the analysis was 29 min. Compounds analyzed were verified by comparison of MS spectra with the NIST MS Search Library, USA.

### 4.5. Preparation of Inoculums

The nutrient growth medium was used to prepare the bacterial inoculums by incubating the test tubes at 30 °C overnight. Next day, the growth was adjusted to 1 × 10^5^ CFU using the nutrient broth [49].

### 4.6. Preparation of Plant Extract Disks

The plant extract disks were prepared (200–1600 μg) using 70% ethanol and dried. Commercially available antibiotics disks were used as standard positive control whereas only ethanol-containing disks were considered negative control [49,50,51].

### 4.7. Agar Disk Diffusion Assay

The method of Wajid et al. [49] was followed for this using Mueller–Hinton agar.

### 4.8. Antimicrobial Susceptibility Testing

Three extracts, i.e., ethanolic, hexane, and chloroform of three plants, i.e., *S. undatus*, *P. vera*, and *O. europaea*, were used to test antimicrobial potential via disk diffusion. Antibiotic susceptibility testing of isolates was performed by using imipenem as a positive control against *E. coli*, vancomycin was used as a positive control against *Klebsiella*, ciprofloxacin was used as a positive control against *Proteus*, levofloxacin was used as a positive control against *Pseudomonas*, and amikacin was used as a positive control against *Staphylococcus.* DMSO served as a negative control [52,53].

### 4.9. Culturing of Cell Lines

Cell lines culturing was performed as per the method of Hadi et al. [54]. Two cell lines were used: the human breast cancer cell line (MCF-7) and ovarian cancer cell line (A2780). Both cell lines were divided into two groups: experimental and control. For the experimental group, both cell lines were treated with 0–1000 µg mL^−1^ of each plant extract in DMEM, whereas plant extracts were not used for the control group. Both cell lines were cultivated separately in DMEM supplemented with 10% fetal bovine serum, 1% nonessential amino acids, and an antibiotic–antimycotic mixture. The cells were placed in a CO_2_ incubator (5% CO_2_) at 37 °C. The proliferation of cells was measured by MTT assay. Furthermore, 200 µL cells were transferred to 96-well microtiter plates (5000 cells per well) and incubated overnight to allow cells to settle at the bottom of each well. Next day, the growth medium was removed, and a new medium containing the plant extract (200 µL) was introduced to each well in different concentrations (0–1000 µg mL^−1^) and incubated for 72 h. Again, MTT assay (5 mg mL^−1^) was used to ascertain the cell proliferation. Finally, the medium was removed, formazan complex was dissolved in DMSO, and absorbance was noted at 545 nm [55].

### 4.10. Molecular Docking

For this, ligands (acarbose for amylase and glucosidase and kojic acid for tyrosinase) were downloaded from the PubChem database as SDF (structured data format) files. The complete protein molecule in .pdb format was converted to pdbqt format, representing the charged entity. The ligands were prepared as PDB files (Protein Data Bank: https://www.rcsb.org/) for molecular retrieval, and different software tools were used, such as auto Dock vina software (AutoDock Vina [version 1.2.0.]), Discovery Studio (BIOVIA Discovery Studio Visualizer 2021), PyRx (https://sourceforge.net/projects/pyrx/), and Babel (Open Babel version 3.0.1). As hydrogen and charges were removed during X-ray crystallography, version 1.5.6. of AutoDockTools was used to add them again. The grid box was then defined by localizing amino acids on the active sites. The total number of runs was 9, giving the output in 9 different poses. The grid box axis, the x, y, and z centers, the NTPS, and exhaustiveness were all stored in a text file in the working directory and were retrieved as needed. Based on the highest binding affinity, the best pose was selected. Nine runs were conducted, resulting in nine different poses. Protein Data Bank enzyme molecules were prepared using Discovery Studio (Discovery Studio 2021 client). Ligand molecules were identified from GC-MS and selected based on major peak areas using Discovery Studio (Discovery Studio 2021 client) [56].

### 4.11. Data Analysis

All data of experimental groups are expressed as means ± SEM for triplicate experiments. ANOVA was used to compare group means, and Bonferroni’s test was used to identify differences between groups using GraphPad (GraphPad Prism 10.1.1) software. *p* ≤ 0.05 was considered significant.

## 5. Conclusions

The findings of our study have important implications for the prompt and effective treatment of the urinary tract, the most common and least common bacterial pathogens causing urinary tract infections. The antibiotic resistance was much more prominent for hexane and chloroform extracts of all plants, whereas ethanolic extract displayed a sensitivity of bacteria against extracts. It is therefore important to analyze biochemical measurements of these successful extracts and test them in clinical trials of patients with urinary tract infections to reduce the increasing trend of antibiotic resistance. These plant extracts also have certain constituents proven as anticancer agents.

## Figures and Tables

**Table 1:** Uropathogens isolated from urine samples.

Sr. No.	Bacterial Isolates	Total
Gram-negative		
1.	*E. coli* OR602871	144
2.	*K. pneumoniae* OR602899	26
3.	*P. aeruginosa* OR602900	15
4.	*P. vulgaris* OR602872	30
Gram-positive		
5.	*S. aureus* OR602898	18
6.	*S. epidermidis* OR602901	31
Total		264

**Table 2:** Resistance profile of Gram-negative uropathogens identified from UTI patients. AMR = antimicrobial resistance.

Antibiotics	Gram Negative Uropathogens														
	*E. coli* OR602871 (*n* = 144)			*K. penumoniae* OR602899 (*n* = 26)			*P. aeruginosa* OR602900 (*n* =15)			*P. vulgaris* OR602872 (*n* = 30)			Total		
	Total	R(*n*)	R(%)	Total	R(*n*)	R(%)	Total	R(*n*)	R(%)	Total	R(*n*)	R(%)	TT	R(*n*)	R(%)
Ampicillin	87	85	97.7	19	19	100.0	5	5	100.0	12	7	58.3	123	116	94.3
Amoxil/Clav	96	82	85.4	17	13	76.5	6	6	100.0	8	8	100.0	127	109	85.8
Ceftriaxone	103	89	86.4	10	10	100.0	7	4	57.1	6	5	83.3	126	108	85.7
Cefixime	3	3	100.0	2	2	100.0	1	0	0.0	0	0	0.0	6	5	83.3
Vancomycin	1	0	0.0	0	0	0.0	2	2	100.0	18	12	66.7	21	14	66.7
Meropenem	26	3	11.5	5	0	0.0	5	1	20.0	4	0	0.0	40	4	10.0
Imipenem	102	9	8.8	6	0	0.0	9	4	44.4	10	0	0.0	127	13	10.2
Gentamycin	98	59	60.2	17	6	35.3	9	5	55.6	18	10	55.6	142	80	56.3
Tobramycin	21	13	61.9	8	0	0.0	3	3	100.0	6	6	100.0	38	22	57.9
Amikacin	92	33	35.9	11	6	54.5	12	2	16.7	16	4	25.0	131	45	34.4
Ciprofloxacin	109	86	78.9	19	13	68.4	8	5	62.5	25	18	72.0	161	122	75.8
Levofloxacin	103	71	68.9	19	6	31.6	8	5	62.5	20	13	65.0	150	95	63.3
Nitrofurantion	74	15	20.3	10	2	20.0	6	0	0.0	8	2	25.0	98	19	19.4
Tazobactam	98	48	49.0	8	6	75.0	13	2	15.4	7	2	28.6	126	58	46.0
Azithromycin	18	12	66.7	1	0	0.0	4	3	75.0	18	17	94.4	41	32	78.0
Doxycyclin	18	14	77.8	2	2	100.0	1	1	100.0	16	3	18.8	37	20	54.1
Overall AMR	1049	622	59.3	154	85	55.2	99	48	48.5	192	107	55.7	1494	862	57.7

**Table 3:** Resistance profile of Gram-positive bacteria identified from UTI patients (overall data).

Antibiotics	Gram-Positive Uropathogens				
	*S. aureus* OR602898 (*n* = 18)		*S. epidermidis* OR602901 (*n* = 31)		Total
	Total	R (%)	Total	R (%)	R (%)
Penicillin	12	91.7	17	100	96.5
Vancomycin	06	0	23	34.8	27.6
Amikacin	05	0	07	71.4	41.7
Ciprofloxacin	13	69.2	20	65.0	66.7
Levofloxacin	07	33	12	58.3	47.4
Imipenem	06	0	08	0	0
Septran	11	100	13	53.8	75.0
Overall AMR for a bacterium	50	66.0	100	57.0	60.0

**Table 4:** Total phenolic, tannins, alkaloids, flavonoids, carotenoids, and steroids in the (a) ethanol, (b) hexane and (c) chloroform fractions of *S. undatus*, *P. vera*, and *O. europaea*.

(a) Ethanol Extract						
Medicinal Plants	Phenols (GAE)	Tannins (GAE)	Alkaloids (ATE)	Flavonoids (QAE)	Carotenoids (GAE)	Steroids (CAE)
*S. undatus*	10.08 ± 1.76	27.87 ± 2.54	17.87 ± 2.58	56.87 ± 3.98	24.09 ± 3.87	10.78 ± 0.67
*P. vera*	9.64 ± 0.65	14.39 ± 1.71	18.84 ± 2.69	30.62 ± 2.31	13.27 ± 1.56	7.34 ± 0.86
*O. europaea*	15.87 ± 2.76	5.87 ± 0.89	28.62 ± 1.31	47.34 ± 0.86	17.27 ± 1.51	12.78 ± 0.57
**(b) Hexane Extract**						
**Medicinal Plants**	**Phenols (GAE)**	**Tannins (GAE)**	**Alkaloids (ATE)**	**Flavonoids (QAE)**	**Carotenoids (GAE)**	**Steroids (CAE)**
*S. undatus*	16.84 ± 2.39	21.87 ± 3.54	25.87 ± 2.38	60.87 ± 4.98	10.27 ± 1.04	9.98 ± 0.67
*P. vera*	12.64 ± 0.64	12.39 ± 1.70	19.84 ± 1.69	71.07 ± 2.67	19.27 ± 1.31	18.84 ± 2.30
*O. europaea*	20.87 ± 1.76	11.87 ± 0.67	35.39 ± 1.31	49.94 ± 5.08	25.37 ± 1.06	8.84 ± 0.69
**(c) Chloroform Extract**						
**Medicinal Plants**	**Phenols (GAE)**	**Tannins (GAE)**	**Alkaloids (ATE)**	**Flavonoids (QAE)**	**Carotenoids (GAE)**	**Steroids (CAE)**
*S. undatus*	13.48 ± 1.45	25.63 ± 3.12	22.63 ± 2.41	58.91 ± 4.71	17.16 ± 2.56	10.32 ± 0.87
*P. vera*	10.65 ± 0.65	13.78 ± 1.45	18.99 ± 1.98	54.18 ± 2.72	15.73 ± 1.45	13.48 ± 1.65
*O. europaea*	17.56 ± 1.96	06.12 ± 0.76	32.54 ± 1.81	48.23 ± 3.45	21.32 ± 1.34	10.81 ± 0.61

Represented values are the average of three analyses (mean) ± standard deviation (SD) in mg GAE/g of extract, where GAE is gallic acid equivalent, QAE/g of extract, QAE is quercetin equivalent, and CAE is cycloartenol equivalent.

**Table 5:** GC-MS analysis of phytoconstituents detected in the ethanolic extract of *S. undatus*.

Phytoconstituents	Molecular Formula (mf)	Molecular Weight (mw)	Retention Index (*n*-alkane Scale in IU)	Antibacterial Activity
2,2-Dimethoxybutane	C_6_H_14_O_2_	118	685	No
Glyceraldehyde	C_3_H_6_O_3_	90	913	Yes
Furfural	C_5_H_4_O_2_	96	831	Yes
Furyl alcohol	C_5_H_6_O_2_	98	885	Yes
Propanoic acid, 3-nitro-, methyl ester	C_4_H_7_NO_4_	133	968	Yes
Dihydroxyacetone	C_3_H_6_O_3_	90	941	Yes
Thymine	C_5_H_6_N_2_O_2_	126	1118	Yes
Methyl furoate	C_6_H_6_O_3_	126	909	Yes
4H-Pyran-4-one, 2,3-dihydro-3,5-dihydroxy-6-methyl-	C_6_H_8_O_4_	144	1269	Yes
Hydroxymethylfurfural	C_6_H_6_O_3_	126	1163	Yes
Acetoglyceride	C_5_H_10_O_4_	134	1091	Yes
2-Amino-2-cyano-4-methylpentanethioamide	C_7_H_13_N_3_S	171	1686	Yes
Vanillin	C_8_H_8_O_3_	152	1392	Yes
Cis-Ethyl3-methyl-3-phenylglycidatephenylethyl2methylbutyrate	C_12_H_14_O_3_	206	1484	Yes
Benzenebutanoic acid, 4-ethyl-ˠ-oxo	C_12_H_14_O_3_	206	1797	No
Phenol, 2,4-ditert-butyl	C_14_H_22_O	206	1555	Yes
Mandelic acid, α-methyl-, DL-	C_12_H_14_O_3_	206	1484	Yes
Atrolactic acid	C_9_H_10_O_3_	166	1441	No
Tetradecanoic acid	C_14_H_28_O_2_	228	1769	Yes
1,4-Hydroxy-4-isopropyl-5-methyl-2-hexyl acetate	C_12_H_20_O_3_	212	1362	No
*n*-Hexadecanoic acid	C_16_H_32_O_2_	256	1968	No
Diisooctyl phthalate	C_24_H_38_O_4_	390	2704	No

**Table 6:** GC-MS analysis of phytoconstituents detected in the ethanolic extract of *P. vera*.

Phytoconstituents	Molecular Formula (mf)	Molecular Weight (mw)	Retention index (*n*-alkane Scale in IU)	Antibacterial Activity
2,2-Dimethoxybutane	C_6_H_14_O_2_	118	685	Yes
Glycerin	C_3_H_8_O_3_	92	967	Yes
4,5-Diamino-2-hydroxypyrimidine	C_4_H_6_N_4_O	126	1512	Yes
Threo-4-Hydroxy-l-lysine lactone	C_6_H_12_N_2_O_2_	144	1433	Yes
N-Methylpyrrole-2-carboxylic acid	C_6_H_7_NO_2_	125	1123	Yes
2-Furancarboxaldehyde,5-(hydroxymethyl)	C_6_H_6_O_3_	126	1176	Yes
4-Hydroxy-2-methylpyrrolidine-2-carboxylic acid	C_6_H_11_NO_3_	145	1424	No
Isosorbide Dinitrate	C_6_H_8_N_2_O_8_	236	-	Yes
Thymol	C_10_H_14_O	150	1266	Yes
Sucrose	C_12_H_22_O_11_	342	3139	Yes
Hexadecanoic acid, methyl ester	C_17_H_34_O_2_	270	1878	Yes
*n*-Hexadecanoic acid	C_16_H_32_O_2_	256	1968	Yes
Hexadecanoic acid, ethyl ester	C_18_H_36_O_2_	284	1978	Yes
9, 12-Octadecanoic acid (*Z*, *Z*)-, methyl ester	C_19_H_34_O_2_	294	2093	Yes
10, Octadecanoic acid, methyl ester	C_19_H_36_O_2_	296	2085	No
9, 12-Octadecadienoic acid	C_18_H_32_O_2_	280	2183	Yes
cis-Vaccenic acid	C_18_H_34_O_2_	282	2175	Yes
9, 12-Octadecanoic acid, ethyl ester	C_20_H_36_O_2_	308	2193	Yes
Octadecanoic acid	C_18_H_36_O_2_	284	-	Yes
Ethyl Oleate	C_20_H_38_O_2_	310	2185	No
5alpha-Cholestan-3beta-ol, 2-methylene-	C_28_H_48_O	400	2652	No
1,2-15,16-Diepoxyhexadecane	C_16_H_30_O_2_	254	1792	No
9-Oximino-2, 7-diethoxyfluorene	C_17_H_17_NO_3_	283	2403	No
1-Heptatriacotanol	C_37_H_76_O	536	3942	No
Ethyl iso-allocholate	C_26_H_44_O_5_	436	3094	Yes
1,2-Benzenedicarboxylic acid, diisooctyl ester	C_24_H_38_O_4_	390	2704	Yes
9,12-Octadecanoic acid (Z,Z)-,2,3-dihydroxypropyl ester	C_21_H_38_O_4_	354	2697	Yes
9-Octadecanoic acid (Z)-, 2-hydroxy-1-(hydroxymethyl) ethyl ester	C_21_H_40_O_4_	356	2705	Yes
ˠ-Tocopherol	C_28_H_48_O_2_	416	3036	Yes

**Table 7:** GC-MS analysis of phytoconstituents detected in the ethanolic extract of *O. europaea*.

Phytoconstituents	Molecular Formula (mf)	Molecular Weight (mw)	Retention Index (*n*-Alkane Scale in IU)	Antibacterial Activity
2,2-Dimethoxybutane	C_6_H_14_O_2_	118	685	No
2,3 butanediol	C_4_H_10_O_2_	90	743	No
3-Hexen-1-ol	C_6_H_12_O	100	868	No
*N*-Propylacetamide	C_5_H_11_NO	101	918	Yes
Phenylmethanol	C_7_H_8_O	108	1036	Yes
Phenylacetaldehyde	C_8_H_8_O	120	1081	No
Cis-2,6-Dimethyl-2,6-octadiene	C_10_H_18_	138	985	No
Β-Lactose	C_12_H_22_O_11_	342	3131	Yes
α-methyl-α-[4-methyl-3-pentenyl]oxiranemethanol	C_10_H_18_O_2_	170	1182	No
Furyl hydroxymethyl ketone	C_6_H_6_O_3_	126	1121	No
Linalyl oxide	C_10_H_18_O_2_	170	1164	Yes
Phenylethyl alcohol	C_8_H_10_O	122	1136	Yes
4H-Pyran-4-one,2,3-dihydro-3,5-dihydroxy-6-methyl-	C_6_H_8_O_4_	144	1269	No
Benzoic acid	C_7_H_6_O_2_	122	1150	Yes
Triethylamine	C_6_H_15_N	101	667	Yes
Benzeneacetic acid	C_8_H_8_O_2_	136	1249	Yes
Nonanoic acid	C_9_H_18_O_2_	158	1272	Yes
Thymol	C_10_H_14_O	150	1262	Yes
Eugenol	C_10_H_12_O_2_	164	1392	Yes
Tyrosol	C_8_H_10_O_2_	138	1356	Yes
4-Hydroxybenzyl cyanide	C_8_H_7_NO	133	1359	No
Levoglucosan	C_6_H_10_O_5_	162	1404	Yes
1-Oxaspiro [2.5]octane,5,5-dimethyl-4-(3-methyl-1,3-butadienyl)-	C_14_H_22_O	206	1431	No
Dihydroactinidiolide	C_11_H_16_O_2_	180	1426	No
Dodecanoic acid	C_12_H_24_O_2_	200	1570	Yes
Fumaric acid, ethyl 2-methylallyl ester	C_10_H_14_O_4_	198	1325	No
3,3,4,6-tetramethyl-1-indanone	C_13_H_16_O	188	1579	No
Vanillacetic acid	C_9_H_10_O_4_	182	1659	No
Heptadecanoic acid	C_17_H_34_O_2_	270	2067	No
2-methyl-6-(4-methylphenyl)hept-2-ene-4-one	C_29_H_50_O_2_	430	-	No
Stigmasterol	C_29_H_48_O	412	2739	No
Oleuropein	C_25_H_32_O_13_	540	2731	Yes

**Table 8:** Antibacterial sensitivity of (a) *S. undatus*, (b) *P. vera* and (c) *O. europaea.* R = resistant; S = sensitive; positive control: standard antibiotics; *E. coli* = imipenem; *K. pneumoniae* = vancomycin; *P. vulgaris* = ciprofloxacin; *P. aeruginosa* = levofloxacin; *S. aureus* = amikacin; negative control: DMSO.

(a) Antibacterial Sensitivity of *S. undatus*						
Sr. No.	Bacterial Isolates	Positive Control	Negative Control	Ethanol	Chloroform	Hexane
1.	*E. coli*	4 mm	1 mm	R	S (25 mm)	R
2.	*K. pneumoniae*	31 mm	0 mm	S (23 mm)	R	R
3.	*P. vulgaris*	39 mm	0 mm	R	S (24 mm)	R
4.	*P. aeruginosa*	41 mm	0 mm	R	R	S (27 mm)
5.	*S. aureus*	2 mm	0 mm	S (26 mm)	R	S (25 mm)
**(b) Antibacterial Sensitivity of *P. vera***						
**Sr. No.**	**Bacterial Isolates**	**Positive Control**	**Negative Control**	**Ethanol**	**Chloroform**	**Hexane**
1.	*E. coli*	3 mm	1 mm	S (28 mm)	R	R
2.	*K. pneumoniae*	38 mm	0 mm	R	R	S (18 mm)
3.	*P. vulgaris*	50 mm	0 mm	S (22 mm)	R	R
4.	*P. aeruginosa*	51 mm	0 mm	S (25 mm)	R	S (21 mm)
5.	*S. aureus*	3 mm	0 mm	S (22 mm)	S (25 mm)	R
**(c) Antibacterial Sensitivity of *O. europaea***						
**Sr. No.**	**Bacterial Isolates**	**Positive Control**	**Negative Control**	**Ethanol**	**Chloroform**	**Hexane**
1.	*E. coli*	35 mm	1 mm	S (15 mm)	R	R
2.	*K. pneumoniae*	1 mm	0 mm	S (16 mm)	R	R
3.	*P. vulgaris*	55 mm	0 mm	S (8 mm)	R	R
4.	*P. aeruginosa*	60 mm	0 mm	S (15 mm)	R	R
5.	*S. aureus*	3 mm	0 mm	R	R	R

## Data Availability

Data are contained within the article and Supplementary Materials.

## Supplementary Materials

The following supporting information can be downloaded at https://www.mdpi.com/article/10.3390/molecules28248148/s1.
