# Supplementary material for: Probing Antibacterial and Anticancer Potential of Selenicereus undatus, Pistacia vera L. and Olea europaea L. against Uropathogens, MCF-7 and A2780 Cancer Cells
Source: Molecules. 2023 Dec 18;28(24):8148. doi: 10.3390/molecules28248148 (PMC10746009; doi:10.3390/molecules28248148)
Supplement: Supplementary file 1 [file molecules-28-08148-s001.zip › Figure S1.pdf]

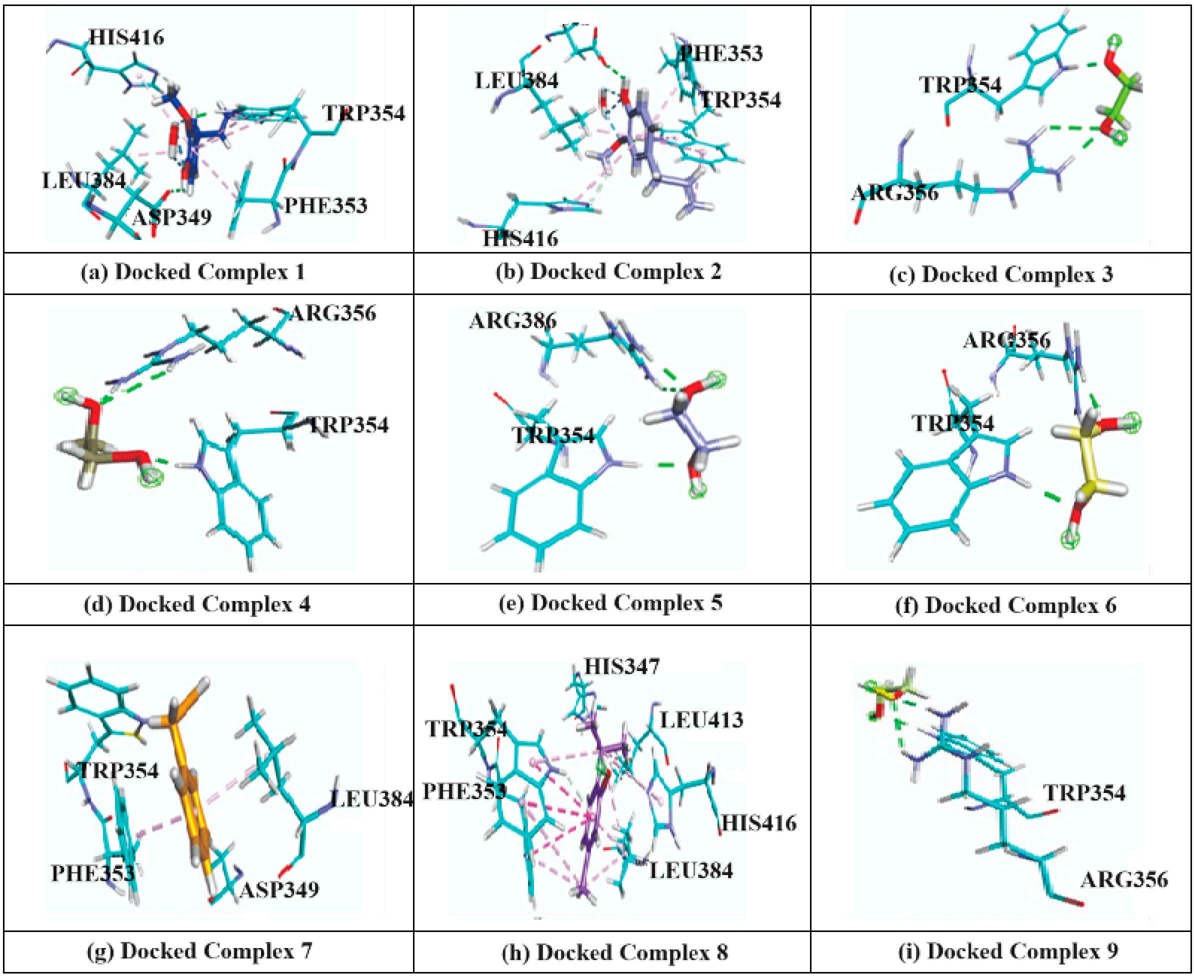

Figure S1. Lypoteichoic acid receptor S domain LTAS (2w5q) with Eugenol (1a), Oleoeuropein (1b), Levoglucosan (1c), luteolin (1d), N-propyl acetamide (1e), Oleuropein (1f), Phenylacetaldehyde (1g), Thymol (1h), 1-Oxaspiro[2.5]octane5,5-dimethyl-4-(3-methyl-1,3-butadienyl)-(1i).
