# Supplementary material for: Probing Antibacterial and Anticancer Potential of Selenicereus undatus, Pistacia vera L. and Olea europaea L. against Uropathogens, MCF-7 and A2780 Cancer Cells
Source: Molecules. 2023 Dec 18;28(24):8148. doi: 10.3390/molecules28248148 (PMC10746009; doi:10.3390/molecules28248148)
Supplement: Supplementary file 1 [file molecules-28-08148-s001.zip › Figure S2.pdf]

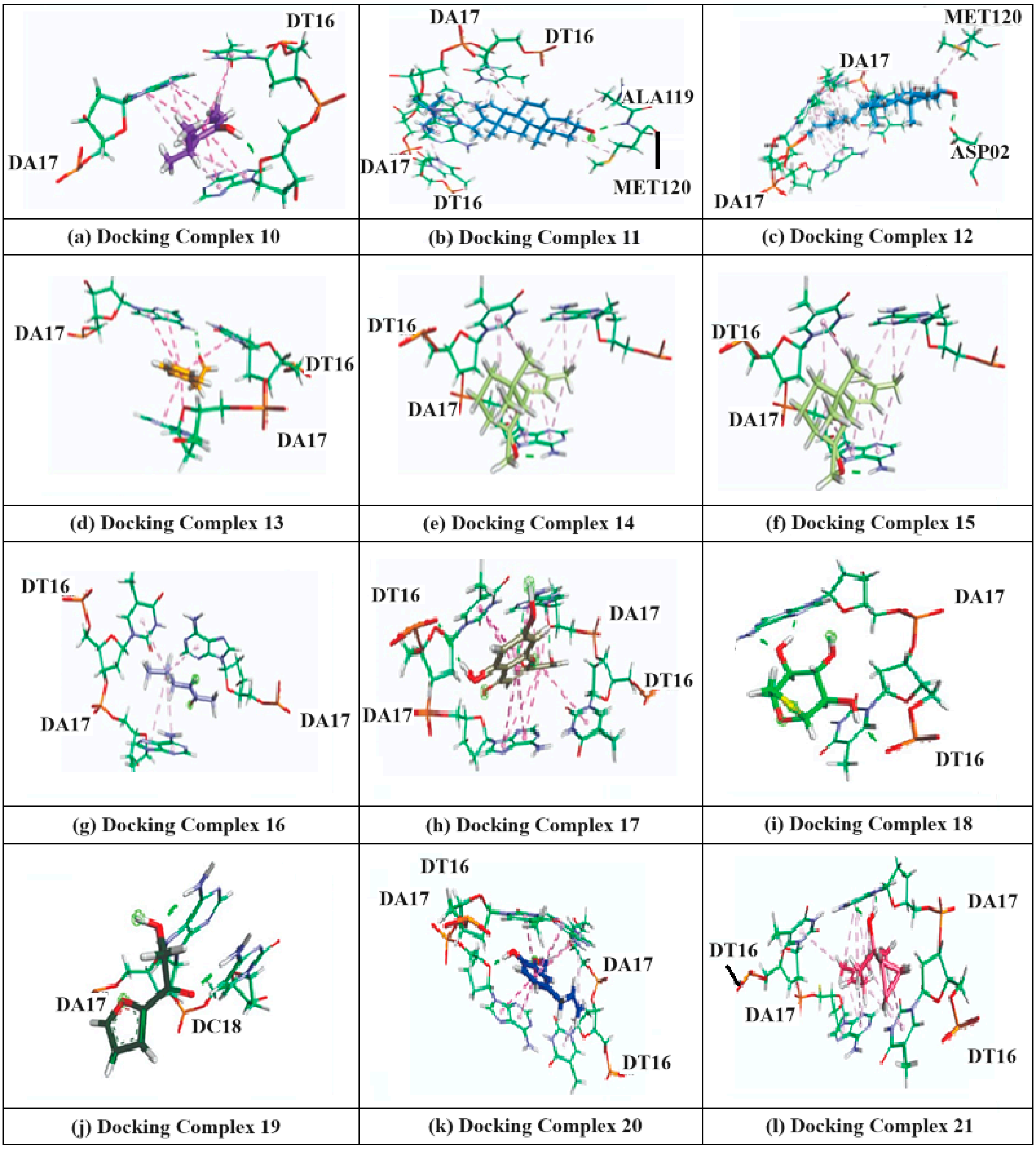

(a) Docking Complex 10 | (b) Docking Complex 11 | (c) Docking Complex 12

(d) Docking Complex 13 | (e) Docking Complex 14 | (f) Docking Complex 15

(g) Docking Complex 16 | (h) Docking Complex 17 | (i) Docking Complex 18

(j) Docking Complex 19 | (k) Docking Complex 20 | (l) Docking Complex 21

Figure S2. DNA Gyrase (6rku) with Thymol (2a), Stigmasterol (2b), Melezitose (2c), Phenylacetaldehyde (2d), 1-Oxaspiro[2.5]octane, 5,5-dimethyl-4-(3-methyl-1,3-butadienyl)- (2e), *Olea europaea* (2f), N-propyl acetamide (2g), Luteolin(2h), Levoglucosan (2i), Furyl hyrdoxymethyl ketone (2j), Eugenol (2k), a-Methyl-a-[4-methyl-3-pentenyl] oxiranemethanol (2l).
