# Supplementary material for: Probing Antibacterial and Anticancer Potential of Selenicereus undatus, Pistacia vera L. and Olea europaea L. against Uropathogens, MCF-7 and A2780 Cancer Cells
Source: Molecules. 2023 Dec 18;28(24):8148. doi: 10.3390/molecules28248148 (PMC10746009; doi:10.3390/molecules28248148)
Supplement: Supplementary file 1 [file molecules-28-08148-s001.zip › SI - 1. GCMS of Selenicerus undatus.pdf]

**Sample Name**	ALI-DRAG-H4-220421-	**Position**	13	**Instrument Name**	GCMS TQQQ	**User Name**	HEJ-G-104-03\Agile
**Inj Vol**	3	**InjPosition**		**SampleType**		**IRM Calibration Status**	Not Applicable
**Data Filename**	ALI-DRAG-H4-220421-.	**ACQ Method**	ALI-IMRAN-140421.M	**Comment**		**Acquired Time**	4/22/2021 1:42:02

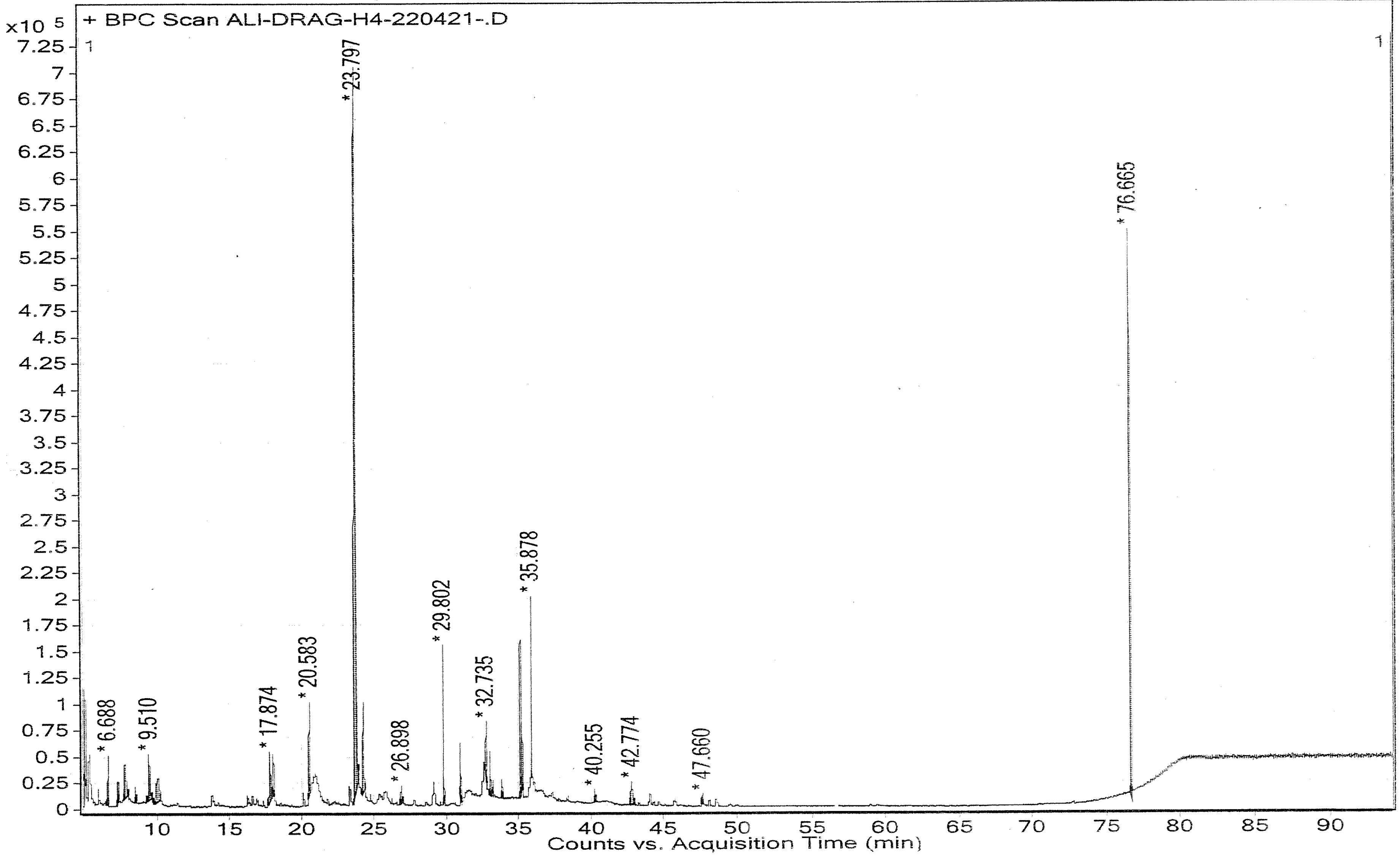

nt

PM

**Sample Name**	ALI-DRAG-H4-220421-	**Position**	13	**Instrument Name**	GCMS TQQQ	**User Name**	HEJ-G-104-03\Agilei
**Inj Vol**	3	**InjPosition**		**SampleType**		**IRM Calibration Status**	Not Applicable
**Data Filename**	ALI-DRAG-H4-220421-.	**ACQ Method**	ALI-IMRAN-140421.M	**Comment**		**Acquired Time**	4/22/2021 1:42:02

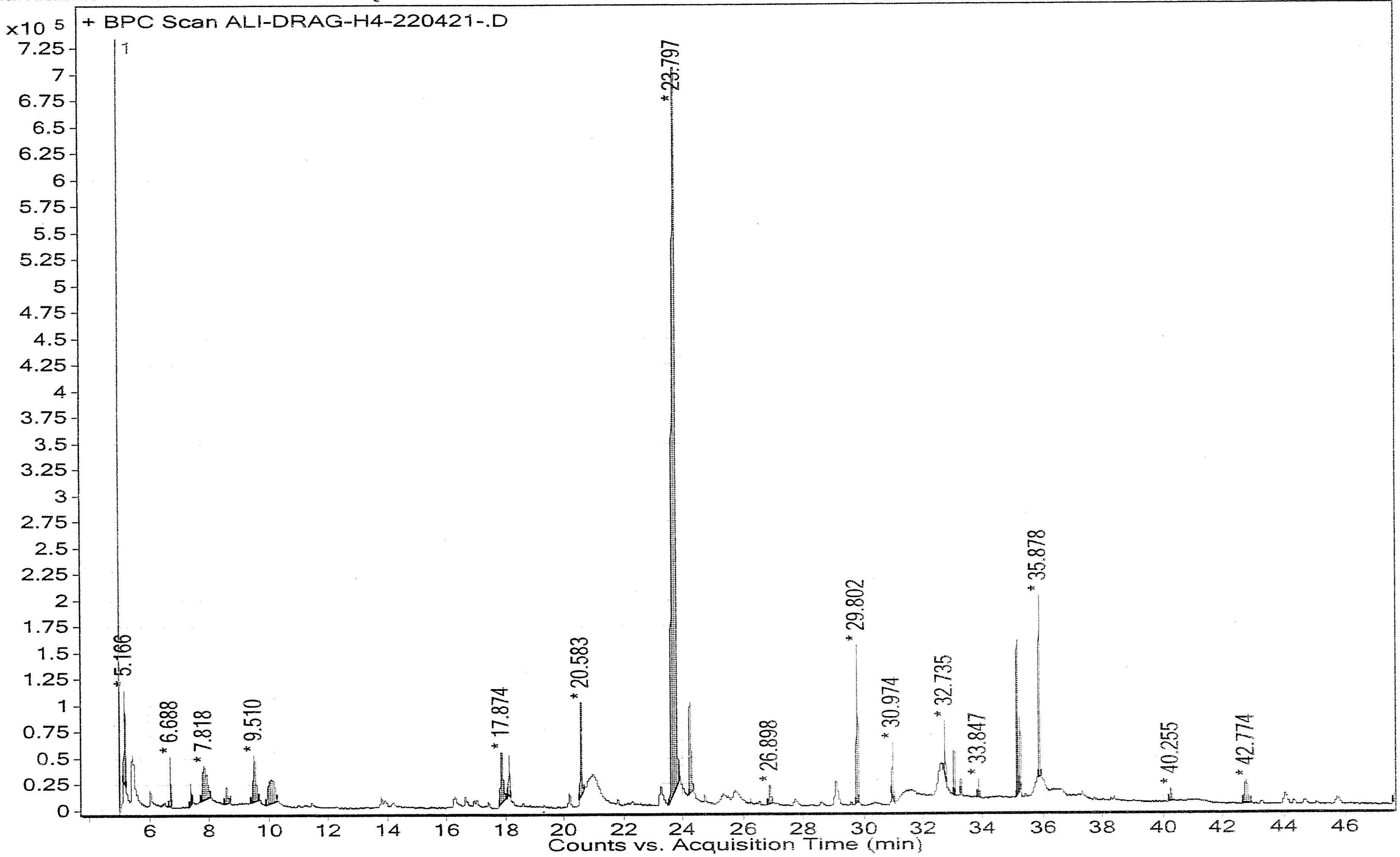

nt

PM

DRAG-H4

Peak Number	RT	Area	Area %	Height	Width	Area Sum %	Height %
1	5.166	344433	6.14	88468	0.114	2.79	12.92
2	6.688	120192	2.14	47537	0.093	0.97	6.94
3	7.358	61537	1.1	21254	0.107	0.5	3.1
4	7.818	349576	6.23	32601	0.342	2.84	4.76
5	8.573	64761	1.15	15362	0.185	0.53	2.24
6	9.51	277201	4.94	44447	0.289	2.25	6.49
7	10.141	353820	6.3	22793	0.431	2.87	3.33
8	17.874	298238	5.31	48641	0.289	2.42	7.1
9	18.131	130153	2.32	38467	0.114	1.06	5.62
10	20.583	348128	6.2	88488	0.153	2.82	12.92
11	23.797	5613910	100	684743	0.328	45.53	100
12	24.26	436526	7.78	85555	0.189	3.54	12.49
13	26.898	81016	1.44	17437	0.164	0.66	2.55
14	29.802	510977	9.1	149294	0.132	4.14	21.8
15	30.974	137729	2.45	51779	0.093	1.12	7.56
16	32.735	110781	1.97	50676	0.078	0.9	7.4
17	33.041	108475	1.93	41739	0.086	0.88	6.1
18	33.266	21541	0.38	13077	0.05	0.17	1.91
19	33.847	39519	0.7	17163	0.075	0.32	2.51
20	35.133	453364	8.08	147822	0.11	3.68	21.59
21	35.229	165399	2.95	66035	0.075	1.34	9.64
22	35.878	493381	8.79	171986	0.1	4	25.12
23	40.255	40843	0.73	11841	0.118	0.33	1.73
24	42.774	184413	3.28	22078	0.303	1.5	3.22
25	47.66	51683	0.92	9885	0.171	0.42	1.44
26	76.665	1532021	27.29	539263	0.135	12.43	78.75
						100.01	

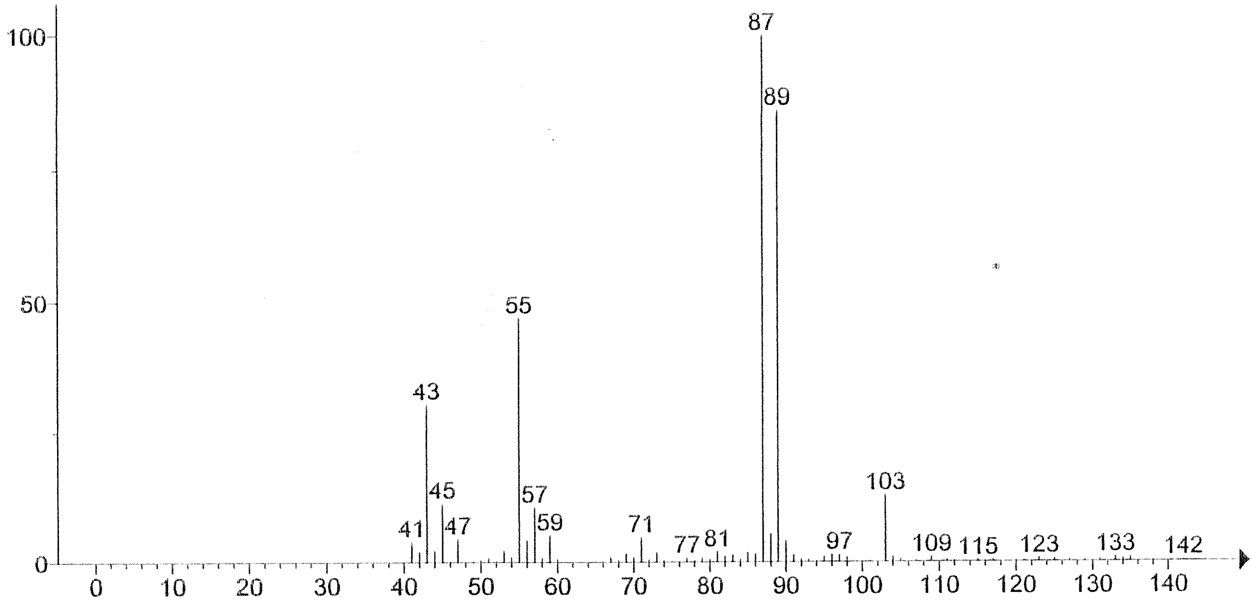

(Text File) +EI Scan (5.166 min) ALI-DRAG-H4-220421-.D Subtract

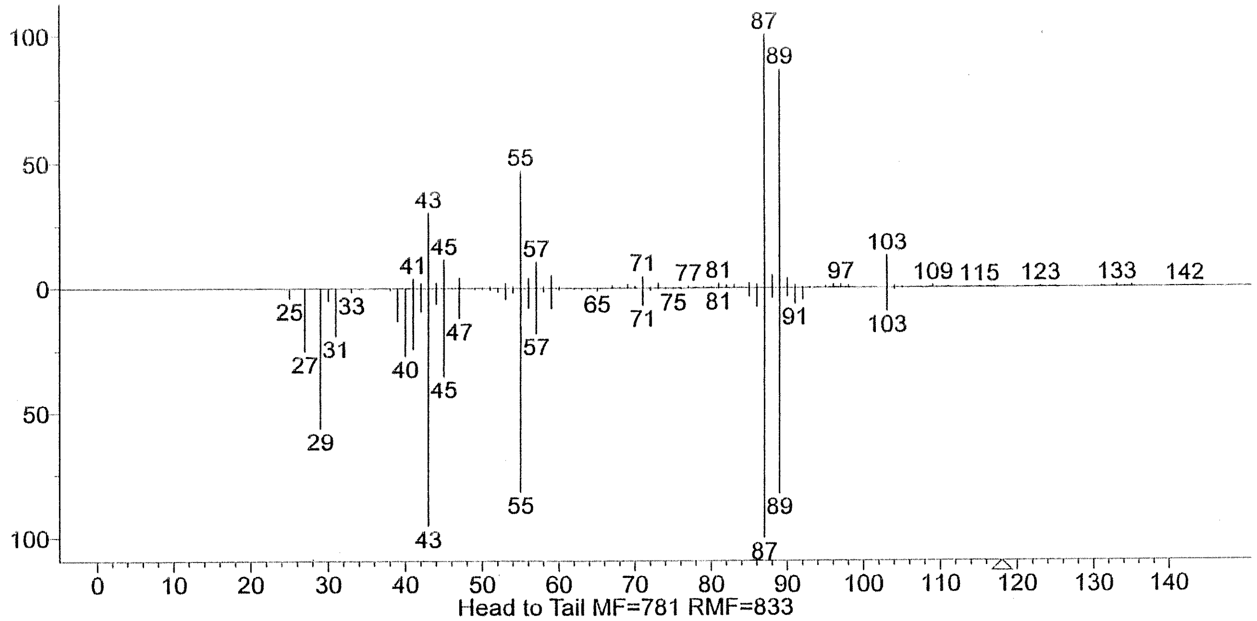

Head to Tail MF=781 RMF=833

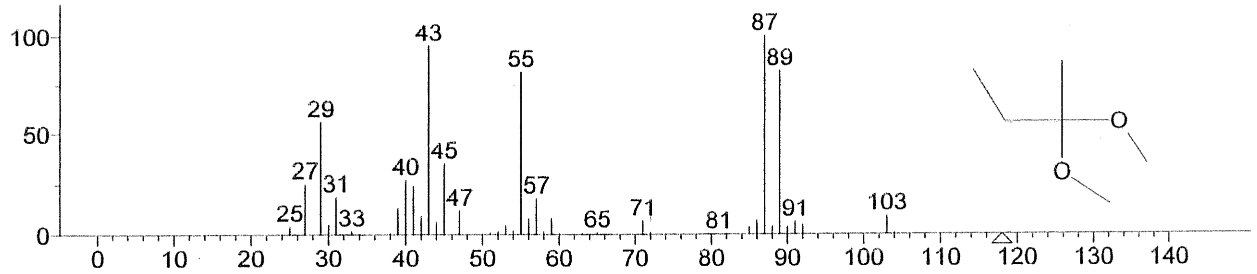

(mainlib) 2,2-Dimethoxybutane

Name: 2,2-Dimethoxybutane
Formula: $C_6H_{14}O_2$
MW: 118 CAS#: 3453-99-4 NIST#: 250250 ID#: 48775 DB: mainlib
Other DBs: None
Contributor: TNO Volatile Compounds in Food - Chemical Concepts
10 largest peaks:
87 999 | 43 950 | 89 822 | 55 817 | 29 560 | 45 351 | 40 270 | 27 251 | 41 242 | 31 188 |
Synonyms:
no synonyms.

Estimated non-polar retention index (n-alkane scale):
Value: 685 iu
Confidence interval (Ethers): 68(50%) 293(95%) iu

Retention index.
1. Value: 748.5 iu
Column Type: Capillary
Column Class: Semi-standard non-polar
Active Phase: SE-54
Column
Length: 30 m
Carrier Gas: He
Column Diameter: 0.25 mm
Phase Thickness: 0.25 um
Data Type: Normal
alkane RI
Program Type: Complex
Description: 40C(5min) =>4C/min => 230C(20min)=> 5C/min => 280C (5min)

Source: Gao, H.; Zhao, T.; Kong, Q.; Chen, X.; Hu, Z., Analysis of unknown organic pollutants in sewage by solid -phase extraction combined with gas chromatography-mass spectrometry, J. Chromatogr. Sci., 42, 2004, 91-99.

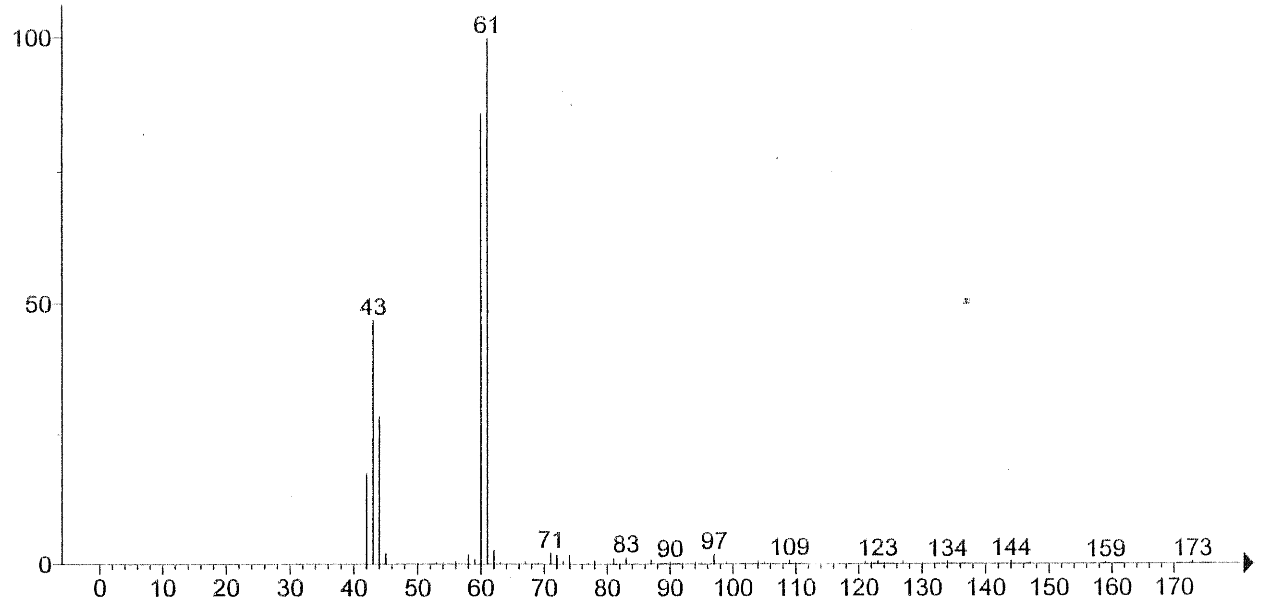

(Text File) +EI Scan (7.354 min) ALI-DRAG-H4-220421-.D Subtract

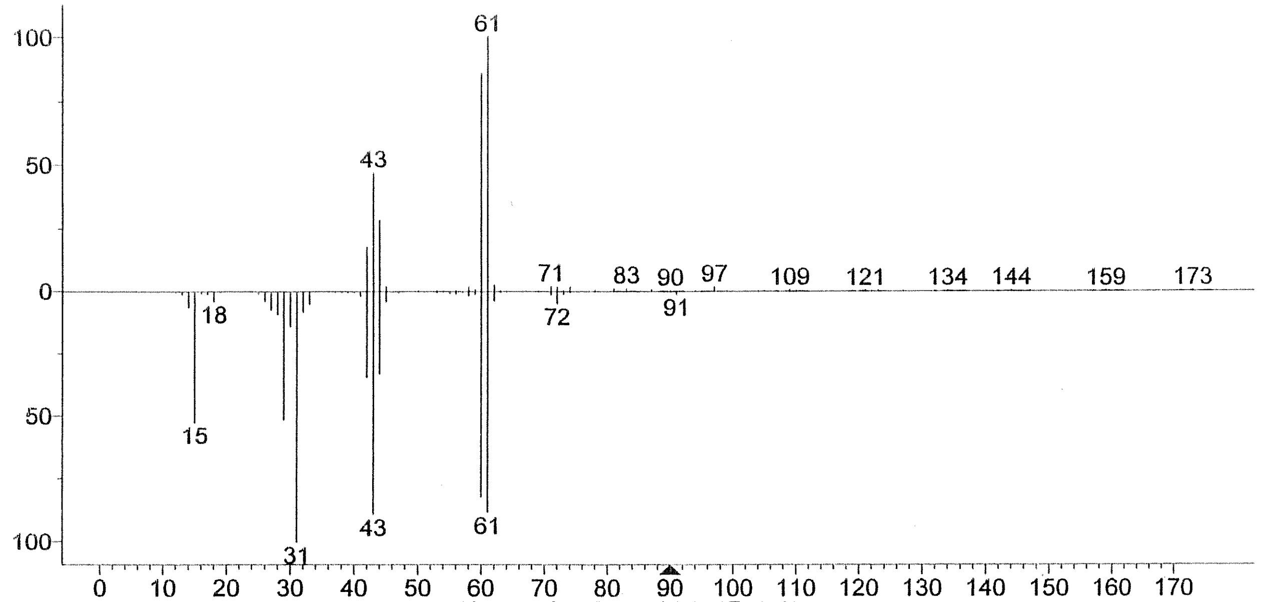

Head to Tail MF=828 RMF=887

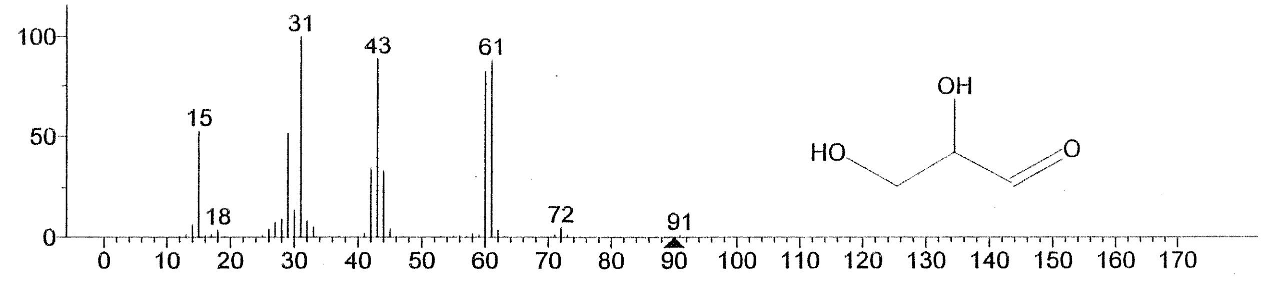

(mainlib) dl-Glyceraldehyde

Name: dl-Glyceraldehyde
Formula: $C_3H_6O_3$
MW: 90 CAS#: 56-82-6 NIST#: 228082 ID#: 1361 DB: mainlib
Other DBs: Fine, TSCA, RTECS, HODOC, EINECS
Contributor: Japan AIST/NIMC Database- Spectrum MS-NW-6286
10 largest peaks:
31 999 | 43 888 | 61 880 | 60 821 | 15 525 | 29 515 | 42 346 | 44 331 | 30 139 | 28 92 |
Synonyms:
1.Propanal, 2,3-dihydroxy-, (±)-
2.Propanal, 2,3-dihydroxy-, (.+-.)-
3.Glyceraldehyde, dl-
4.Glyceraldehyde, (±)-
5.DL-Glyceric aldehyde
6.2,3-Dihydroxypropanal #

Estimated non-polar retention index (n-alkane scale):
Value: 913 iu
Confidence interval (Diverse functional groups): 89(50%) 382(95%) iu

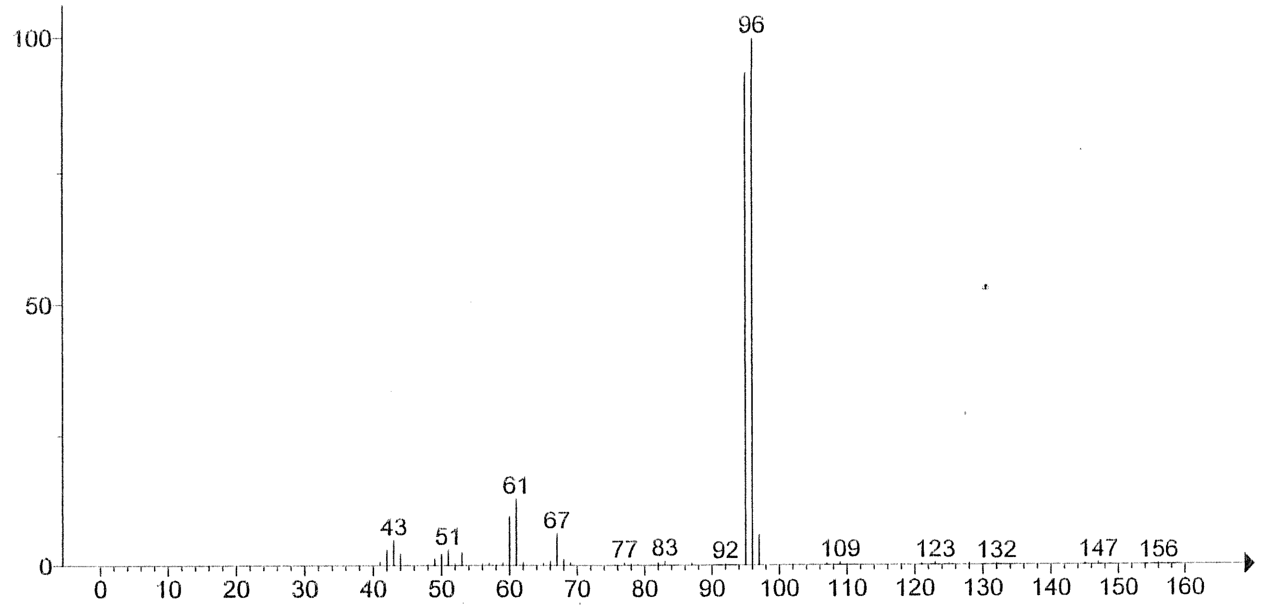

(Text File) +EI Scan (7.818 min) ALI-DRAG-H4-220421-.D Subtract

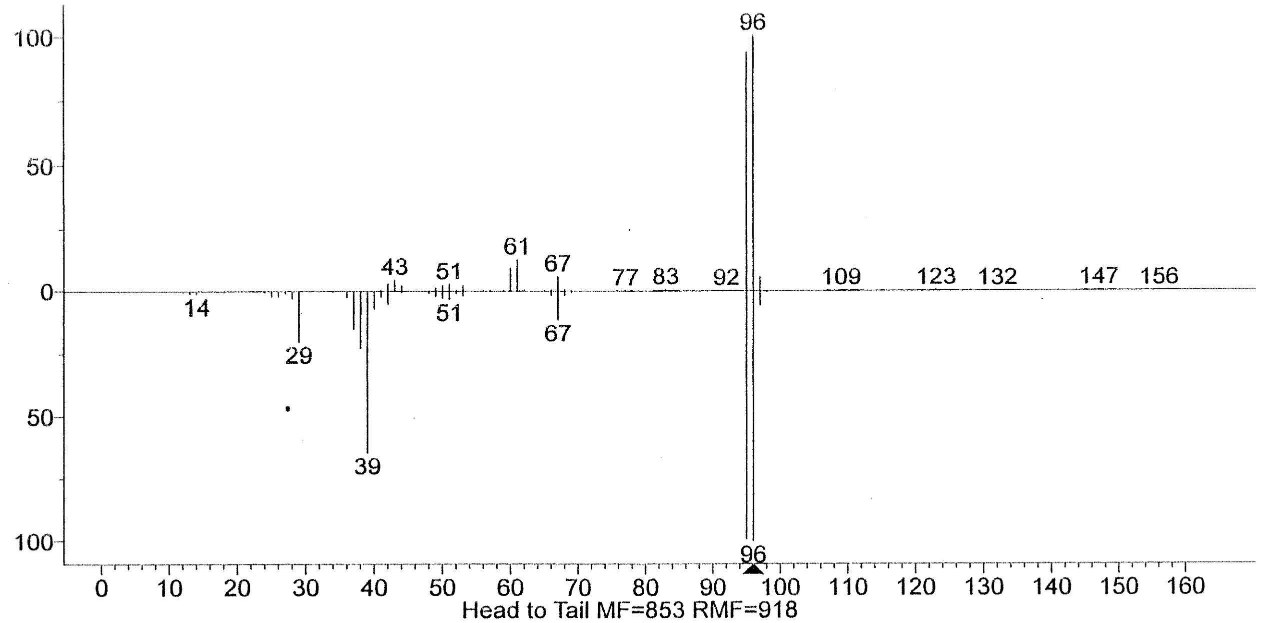

Head to Tail MF=853 RMF=918

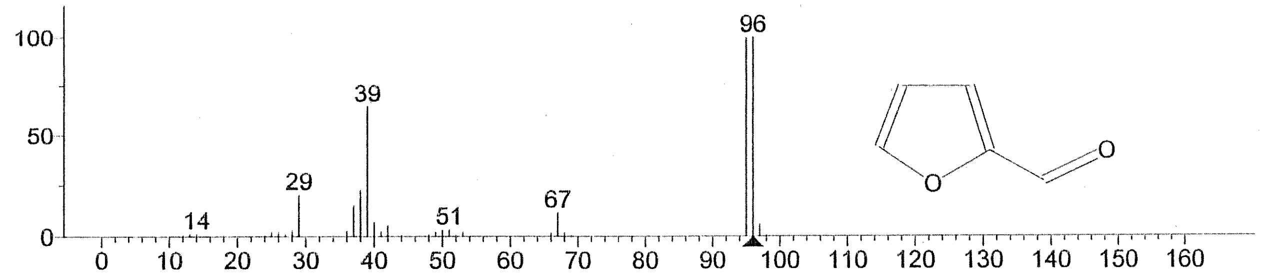

(mainlib) Furfural

Name: Furfural
Formula: $C_5H_4O_2$
MW: 96 CAS#: 98-01-1 NIST#: 118785 ID#: 59378 DB: mainlib
Other DBs: Fine, TSCA, RTECS, EPA, HODOC, NIH, EINECS, IRDB
Contributor: NIST Mass Spectrometry Data Center, 1990.
10 largest peaks:
96 999 | 95 991 | 39 644 | 38 226 | 29 201 | 37 151 | 67 114 | 40 69 | 97 57 | 42 53 |
Synonyms:
1.2-Furancarboxaldehyde
2.2-Furaldehyde
3.α-Furole
4.Artificial ant oil
5.Fural
6.Furaldehyde
7.Furale
8.Furancarbonal
9.Furfuraldehyde
10.Furfurole
11.Furfurylaldehyde
12.Furole
13.Pyromucic aldehyde
14.2-Formylfuran
15.2-Furanaldehyde
16.2-Furancarbonal
17.2-Furfural
18.2-Furfuraldehyde
19.2-Furylaldehyde
20.Furol
21.2-Furylmethanal
22.Artificial oil of ants
23.Furfurale
24.Furfurol
25.Nci-C56177
26.2-Furil-metanale
27.2-Furankarbaldehyd
28.Furfuralu
29.Rcra waste number U125
30.UN 1199
31.2-Furylaldehyde xypropane
32.2-Furylcarboxaldehyde
33.Cyclic aldehyde
34.Qo furfural

Estimated non-polar retention index (n-alkane scale):
Value: 831 iu
Confidence interval (Diverse functional groups): 89(50%) 382(95%) iu

Retention index.
1. Value: 794 iu
Column Type: Capillary
Column Class: Standard non-polar
Active Phase: CP Sil 5 CB
Column
Length: 60 m

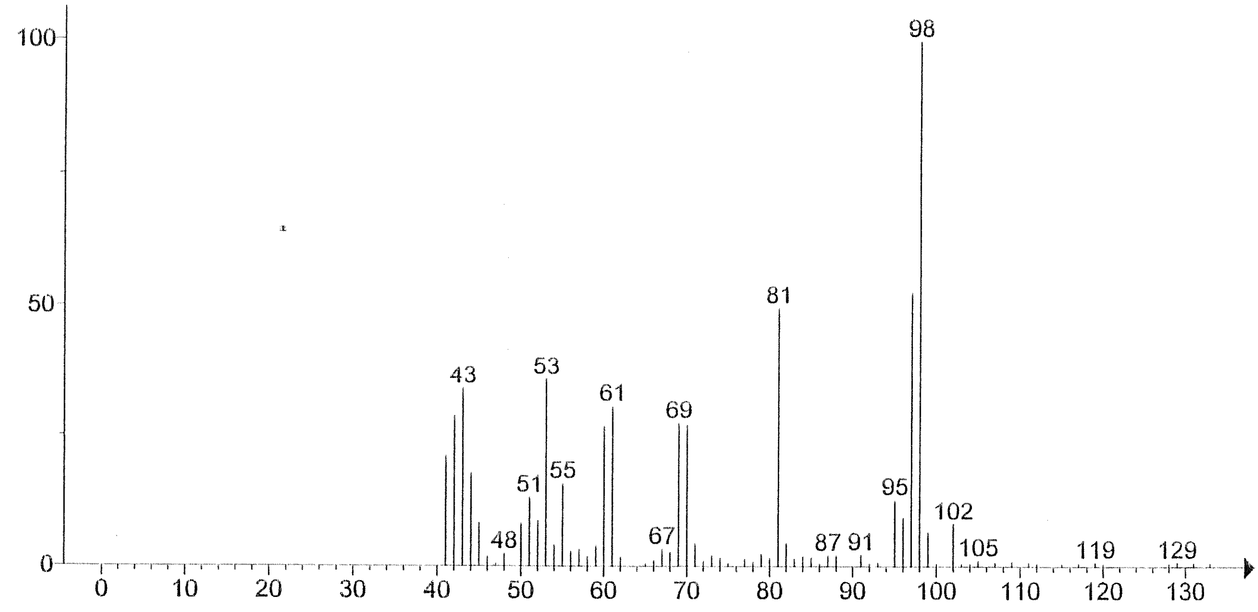

(Text File) +EI Scan (8.584 min) ALI-DRAG-H4-220421-.D

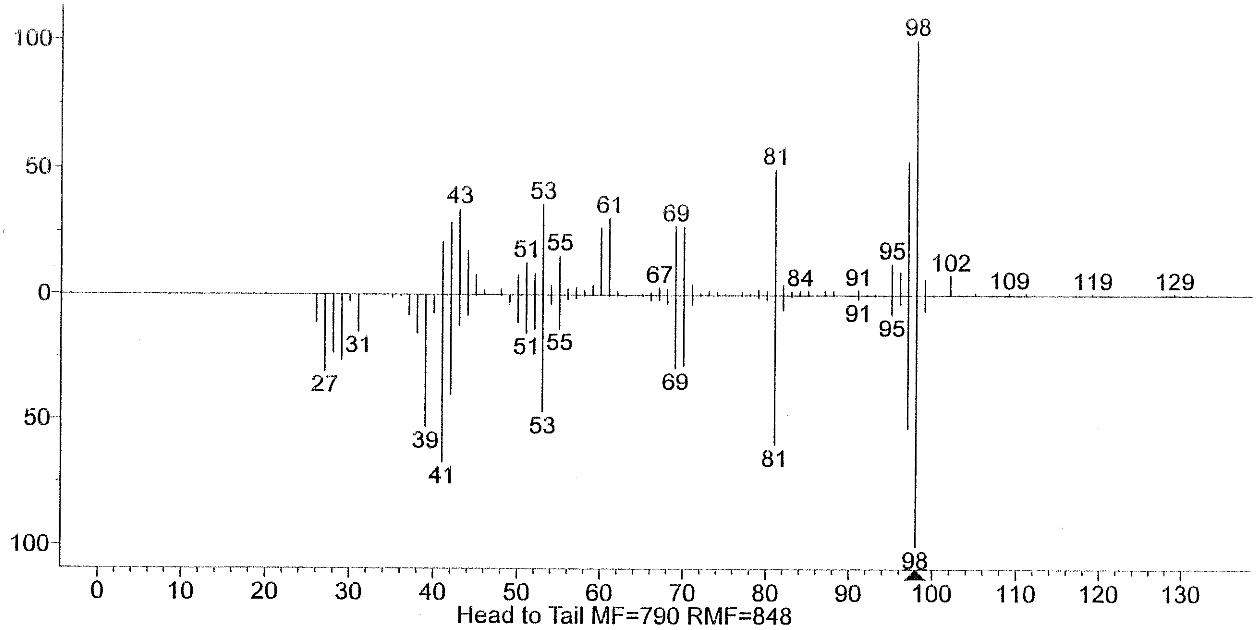

Head to Tail MF=790 RMF=848

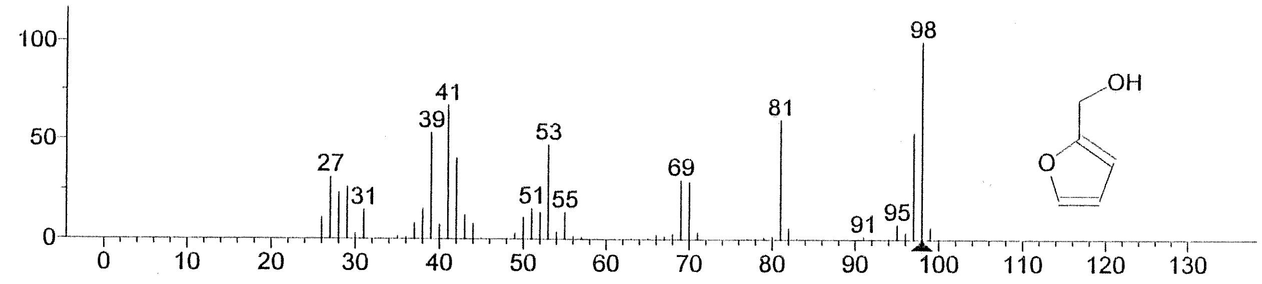

(replib) 2-Furanmethanol

Name: 2-Furanmethanol
Formula: $C_5H_6O_2$
MW: 98 CAS#: 98-00-0 NIST#: 149344 ID#: 12838 DB: replib
Other DBs: Fine, TSCA, RTECS, EPA, HODOC, NIH, EINECS, IRDB
Contributor: Chemical Concepts
10 largest peaks:
98 999 | 41 668 | 81 597 | 97 532 | 39 529 | 53 469 | 42 403 | 27 307 | 69 294 | 70 284 |
Synonyms:
1.Furfuryl alcohol
2.α-Furfuryl alcohol
3.α-Furylcarbinol
4.Furfuralcohol
5.Furyl alcohol
6.Furylcarbinol
7.2-(Hydroxymethyl)furan
8.2-Furancarbinol
9.2-Furanylmethanol
10.2-Furfuryl alcohol
11.2-Furylcarbinol
12.2-Furylmethanol
13.5-Hydroxymethylfuran
14.Furfural alcohol
15.Methanol, (2-furyl)-
16.NCI-C56224
17.2-Furfurylalkohol
18.UN 2874
19.5-Hydroxymethylfuranal
20.FA
21.Qo furfuryl alcohol

Estimated non-polar retention index (n-alkane scale):
Value: 885 iu
Confidence interval (Diverse functional groups): 89(50%) 382(95%) iu

Retention index.
1. Value: 819 iu
Column Type: Capillary
Column Class: Standard non-polar
Active Phase: CP Sil 5 CB
Column
Length: 60 m
Carrier Gas: He
Column Diameter: 0.32 mm
Phase Thickness: 0.25 um
Data Type: Linear
RI
Program Type: Ramp
Start T: 60 C
End T: 280 C
Heat Rate: 3 K/min
Start Time: 10 min
End Time: 60
min
Source: Pino, J.; Almora, K.; Marbot, R., Volatile components of papaya (Carica papaya L., maradol variety)

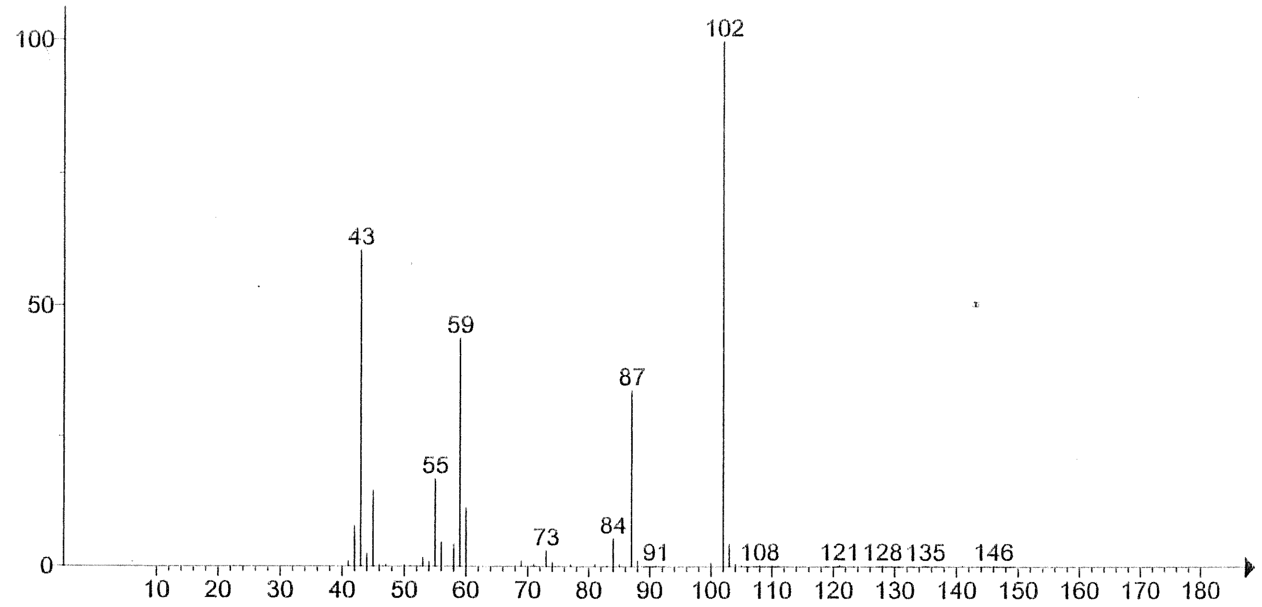

(Text File) +EI Scan (9.514 min) ALI-DRAG-H4-220421-.D Subtract

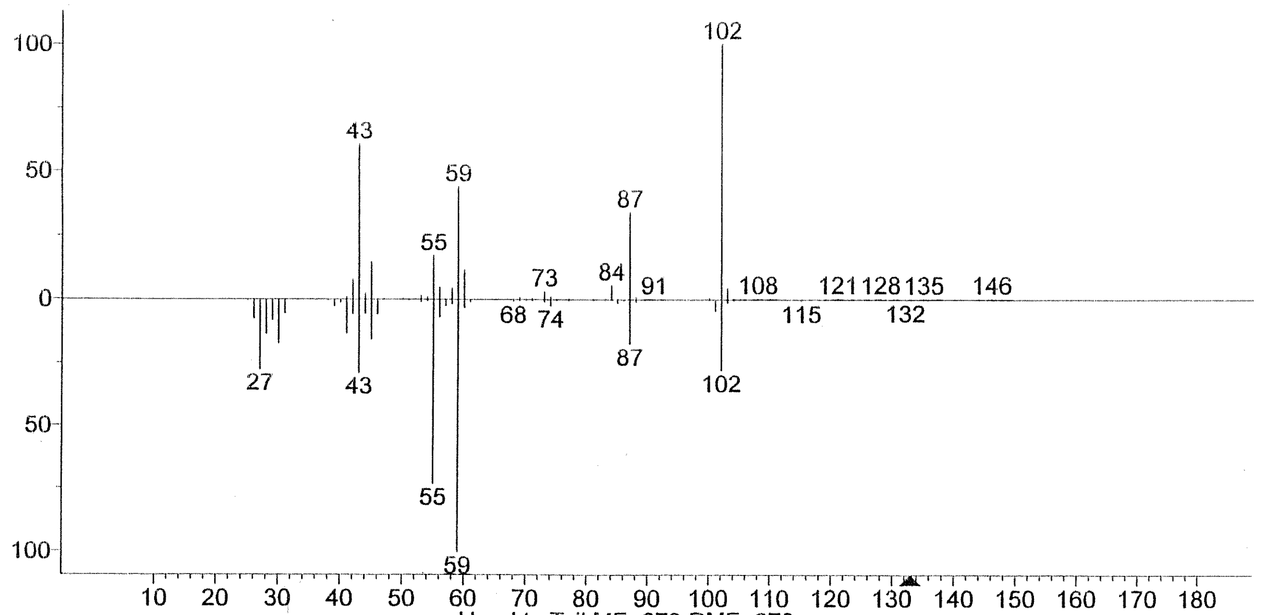

Head to Tail MF=672 RMF=679

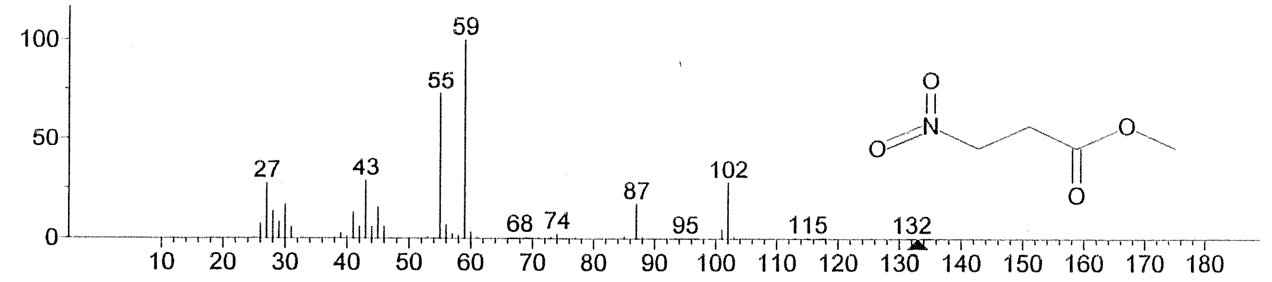

(mainlib) Propanoic acid, 3-nitro-, methyl ester

Name: Propanoic acid, 3-nitro-, methyl ester
Formula: $C_4H_7NO_4$
MW: 133 CAS#: 20497-95-4 NIST#: 188743 ID#: 26333 DB: mainlib
Other DBs: None
Contributor: Chemical Concepts
10 largest peaks:
59 999 | 55 729 | 43 287 | 102 277 | 27 274 | 87 172 | 30 171 | 45 154 | 28 134 | 41 132 |
Synonyms:
1.Methyl 3-nitropropanoate #

Estimated non-polar retention index (n-alkane scale):
Value: 968 iu
Confidence interval (Diverse functional groups): 89(50%) 382(95%) iu

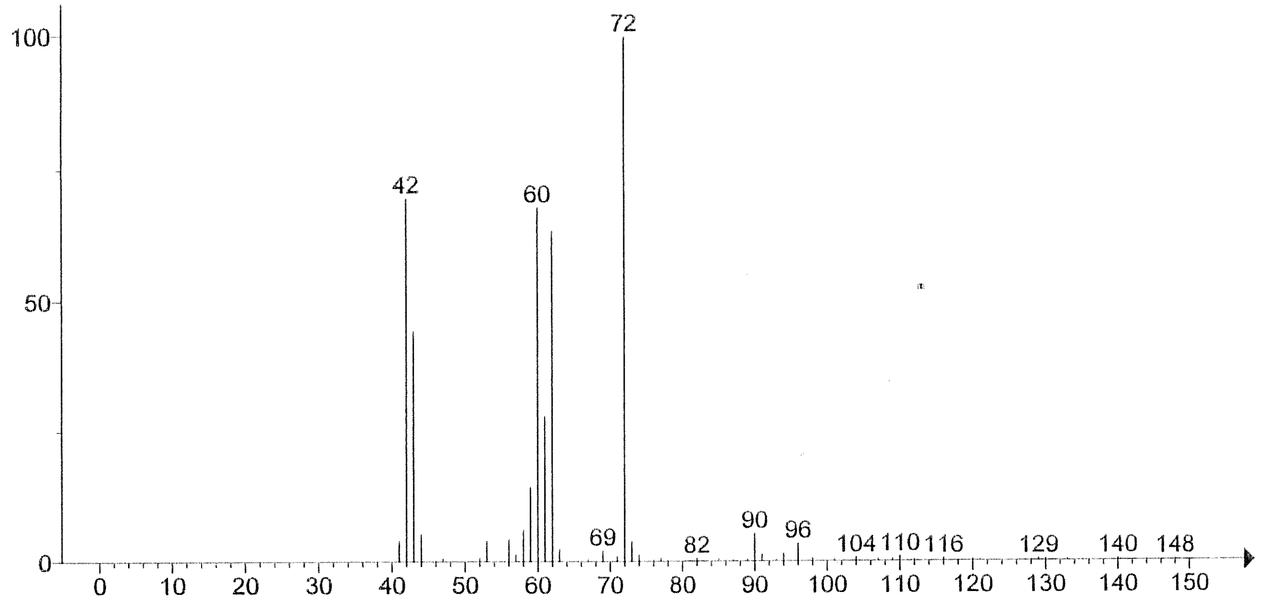

(Text File) +EI Scan (10.106 min) ALI-DRAG-H4-220421-.D Subtract

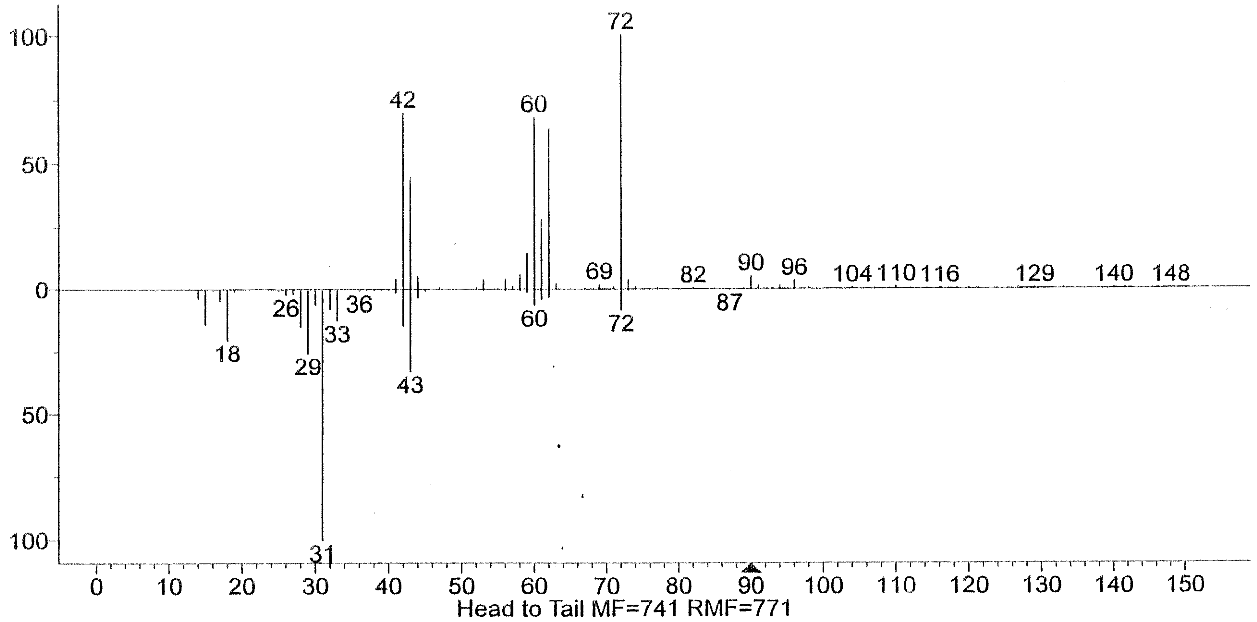

Head to Tail MF=741 RMF=771

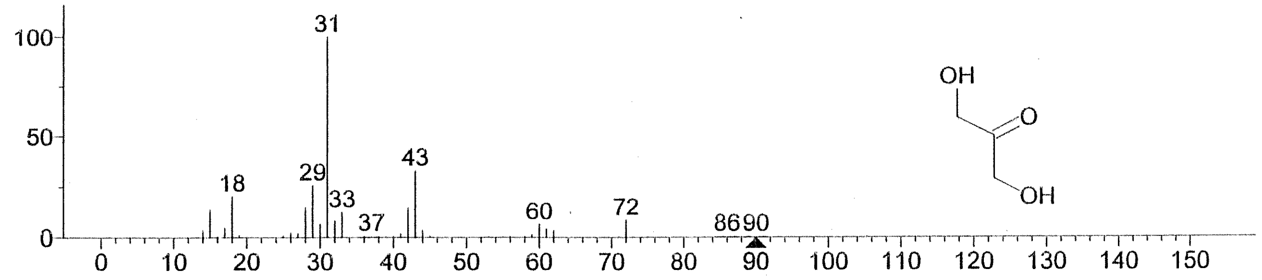

(mainlib) 2-Propanone, 1,3-dihydroxy-

Name: 2-Propanone, 1,3-dihydroxy-
Formula: $C_3H_6O_3$
MW: 90 CAS#: 96-26-4 NIST#: 743 ID#: 1356 DB: mainlib
Other DBs: Fine, TSCA, RTECS, HODOC, NIH, EINECS
10 largest peaks:
31 999 | 43 330 | 29 257 | 18 204 | 28 150 | 42 147 | 15 140 | 33 125 | 72 83 | 32 80 |
Synonyms:
1.Dihydroxyacetone
2.Chromelin
3.Dihyxal
4.Otan
5.Oxantin
6.Oxatone
7.Soleal
8.Triulose
9.Viticolor
10.1,3-Dihydroxy-2-propanone
11.1,3-Dihydroxyacetone
12.NSC-24343
13.1,3-Dihydroxypropanone
14.1,3-Dihydroxydimethyl ketone
15.Aliphatic ketone
16.Ketochromin
17.Protosol

Estimated non-polar retention index (n-alkane scale):
Value: 941 iu
Confidence interval (Diverse functional groups): 89(50%) 382(95%) iu

Retention index.
1. Value: 2068 iu
Column Type: Capillary
Column Class: Standard polar
Active Phase: BP-20
Column Length:
30 m
Carrier Gas: He
Column Diameter: 0.25 mm
Phase Thickness: 0.25 um
Data Type: Normal alkane
RI
Program Type: Complex
Description: 50C(1min) =>2.5C/min =>100C =>2C/min =>180C =>15C/min
=>220C
Source: Pontes, M.; Marques, J.C.; Camara, J.S., Screening of volatile composition from Portuguese multifloral honeys using headspace solid-phase microextraction-gas chromatography-quadrupole mass spectrometry, Talanta, 74, 2007, 91-103.

2. Value: 2075 iu
Column Type: Capillary
Column Class: Standard
polar
Active Phase: BP-20
Column Length: 30 m

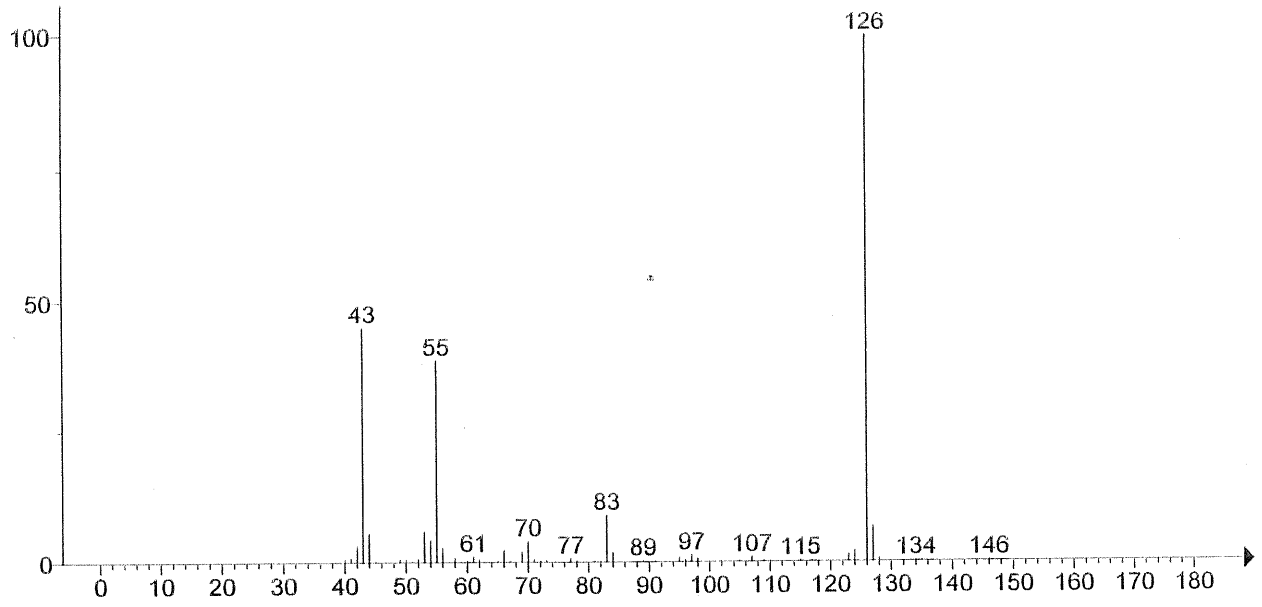

(Text File) +EI Scan (17.871 min) ALI-DRAG-H4-220421-.D Subtract

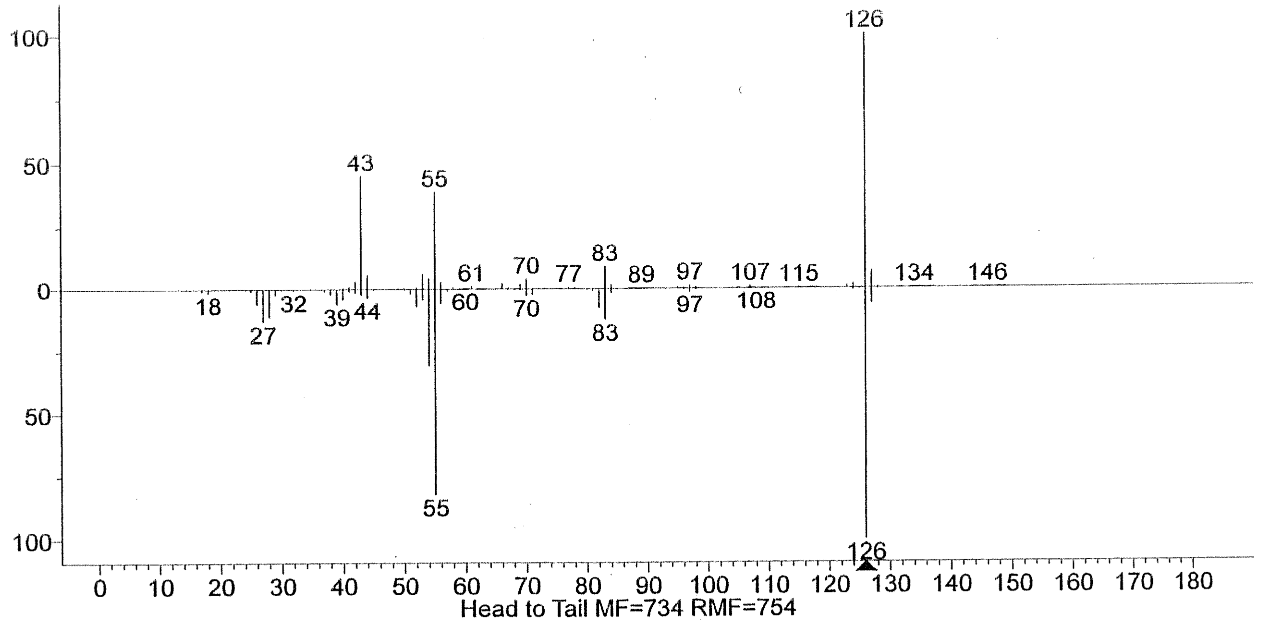

Head to Tail MF=734 RMF=754

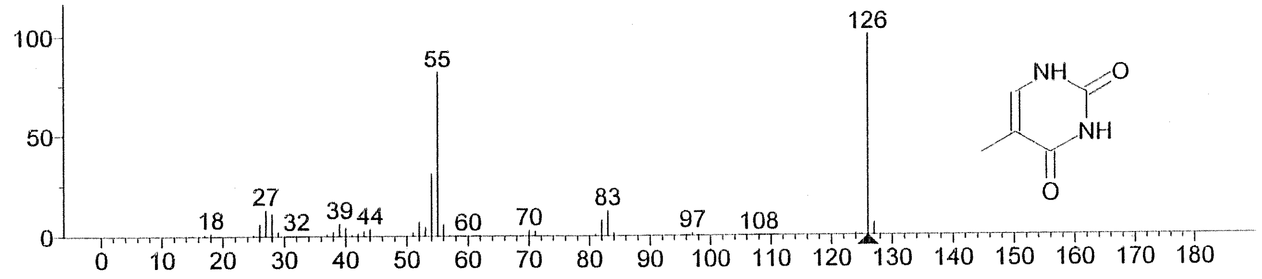

(mainlib) Thymine

Name: Thymine
Formula: $C_5H_6N_2O_2$
MW: 126 CAS#: 65-71-4 NIST#: 228115 ID#: 89146 DB: mainlib
Other DBs: Fine, TSCA, RTECS, HODOC, NIH, EINECS
Contributor: Japan AIST/NIMC Database- Spectrum MS-NW- 639
10 largest peaks:
126 999 | 55 818 | 54 306 | 27 131 | 83 124 | 28 112 | 82 78 | 52 72 | 127 63 | 26 62 |
Synonyms:
1.2,4(1H,3H)-Pyrimidinedione, 5-methyl-
2.Thymin
3.2,4-Dihydroxy-5-methylpyrimidine
4.5-Methyluracil
5.5-Methyl-2,4-dioxypyrimidine
6.5-Methyl-2,4(1H,3H)-pyrimidinedione

Estimated non-polar retention index (n-alkane scale):
Value: 1118 iu
Confidence interval (Nitrogen-containing): 83(50%) 356(95%) iu

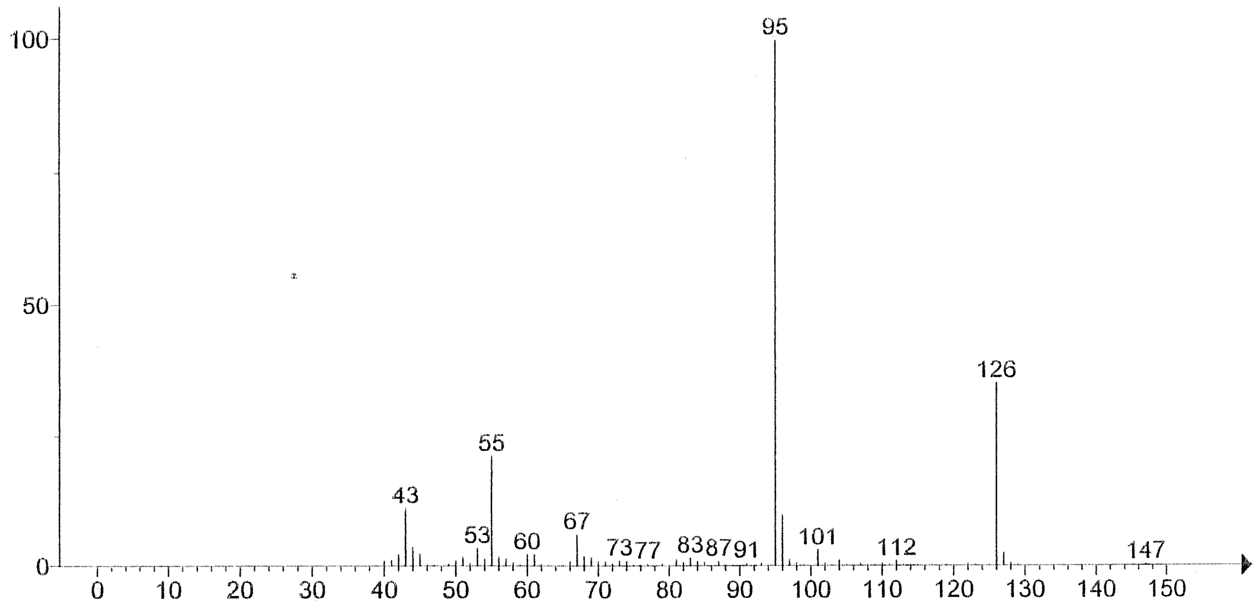

(Text File) +EI Scan (18.135 min) ALI-DRAG-H4-220421-.D

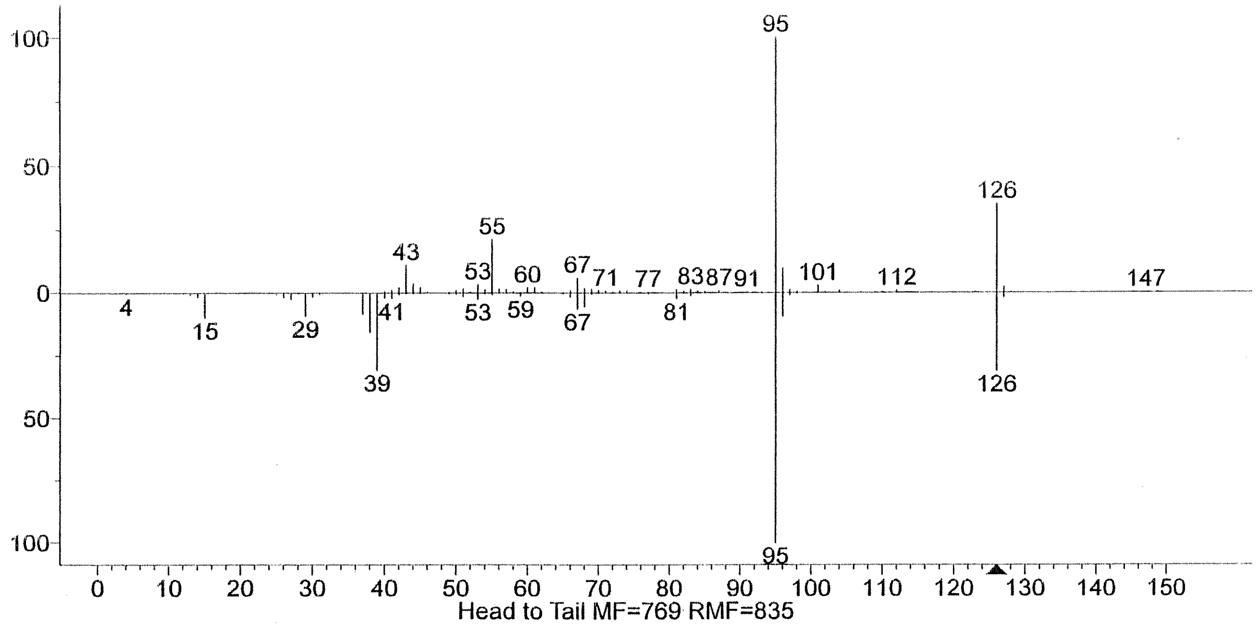

Head to Tail MF=769 RMF=835

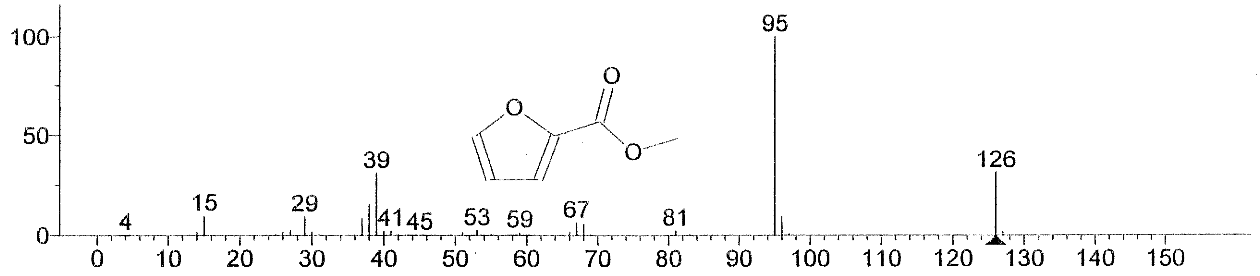

(mainlib) Methyl 2-furoate

Name: Methyl 2-furoate
Formula: $C_6H_6O_3$
MW: 126 CAS#: 611-13-2 NIST#: 134082 ID#: 58739 DB: mainlib
Other DBs: TSCA, RTECS, HODOC, NIH, EINECS, IRDB
Contributor: NIST Mass Spectrometry Data Center, 1994
10 largest peaks:
95 999 | 126 311 | 39 307 | 38 156 | 15 98 | 96 94 | 29 92 | 37 85 | 67 63 | 68 55 |
Synonyms:
1.2-Furancarboxylic acid, methyl ester
2.2-Furoic acid, methyl ester
3.Methyl pyromucate
4.Methyl 2-furancarboxylate
5.Pyromucic acid methyl ester
6.Methyl α-furoate
7.Methyl furoate
8.Furan-α-carboxylic acid methyl ester
9.2-Furancarboxylic acid, methyl ester

Estimated non-polar retention index (n-alkane scale):
Value: 909 iu
Confidence interval (Diverse functional groups): 89(50%) 382(95%) iu

Retention index.
1. Value: 956 iu
Column Type: Capillary
Column Class: Standard non-polar
Active Phase: SE-30
Data Type:
Normal alkane RI
Program Type: Ramp
Source: Vinogradov, B.A., Production, composition, properties and application of essential oils, 2004.

2. Value: 956 iu
Column Type: Capillary
Column Class: Standard non
-polar
Active Phase: OV-101
Column Length: 50 m
Column Diameter: 0.22 mm
Data Type: Normal alkane
RI
Program Type: Ramp
Start T: 80 C
End T: 200 C
Heat Rate: 2 K/min
Source: Egolf, L.M.; Jurs, P.C.,
Quantitative structure-retention and structure-odor intensity relationships for a diverse group of odor-active compounds, Anal. Chem., 65, 1993, 3119-3126.

<...>

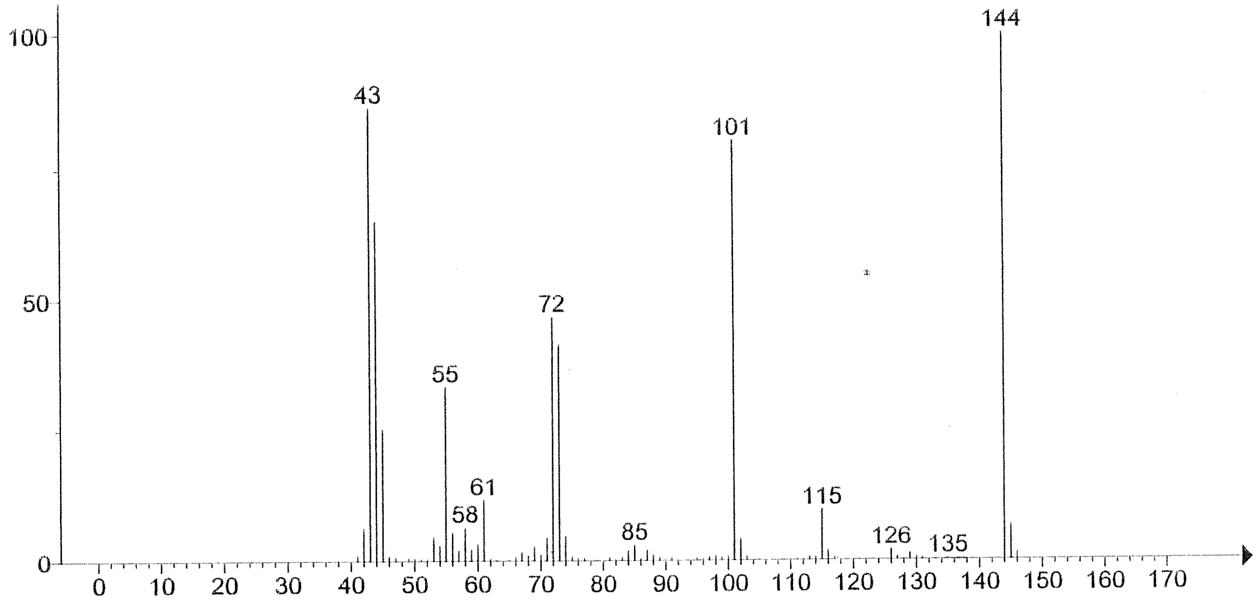

(Text File) +EI Scan (20.579 min) ALI-DRAG-H4-220421-.D

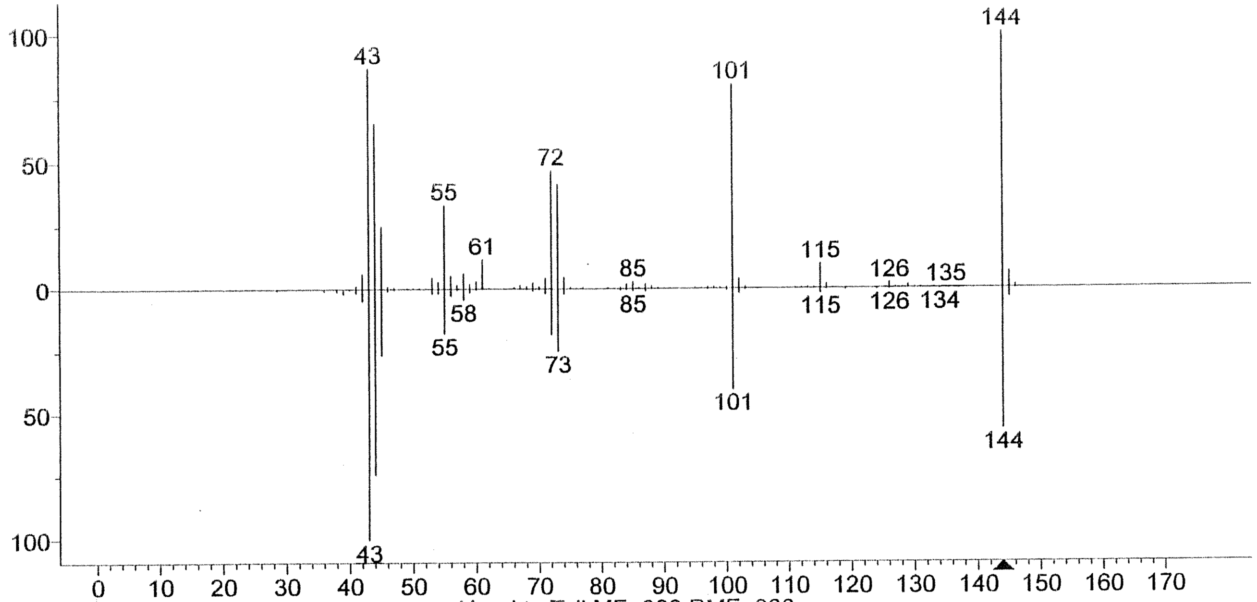

Head to Tail MF=860 RMF=888

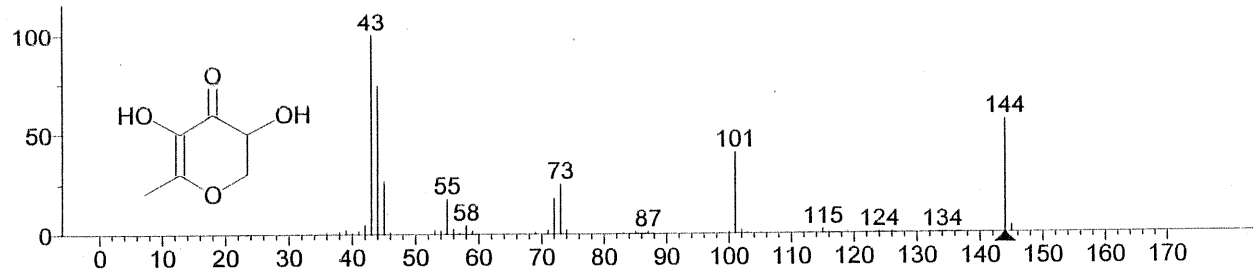

(replib) 4H-Pyran-4-one, 2,3-dihydro-3,5-dihydroxy-6-methyl-

Name: 4H-Pyran-4-one, 2,3-dihydro-3,5-dihydroxy-6-methyl-
Formula: $C_6H_8O_4$
MW: 144 CAS#: 28564-83-2 NIST#: 108691 ID#: 1857 DB: replib
Other DBs: RTECS
Contributor: Philip Morris R&D
10 largest peaks:
43 999 | 44 742 | 144 563 | 101 402 | 45 264 | 73 249 | 72 182 | 55 177 | 42 48 | 58 43 |
Synonyms:
1.3,5-Dihydroxy-6-methyl-2,3-dihydro-4H-pyran-4-one #

Estimated non-polar retention index (n-alkane scale):
Value: 1269 iu
Confidence interval (Diverse functional groups): 89(50%) 382(95%) iu

Retention index.
1. Value: 1119 iu
Column Type: Capillary
Column Class: Standard non-polar
Active Phase: DB-1
Column
Length: 60 m
Carrier Gas: He
Column Diameter: 0.25 mm
Phase Thickness: 0.25 um
Data Type: Linear
RI
Program Type: Complex
Description: -20C (5min) => 10C/min => 100C => 4C/min =>200C => 10C/min =>
280C
Source: Eri, S.; Khoo, B.K.; Lech, J.; Hartman, T.G., Direct thermal desorption-gas chromatography and gas chromatography-mass spectrometry profiling of hop (Humulus lupulus L.) essential oils in support of varietal characterization, J. Agric. Food Chem., 48, 2000, 1140-1149.

2. Value: 1107 iu
Column Type: Capillary
Column
Class: Standard non-polar
Active Phase: DB-1
Column Length: 60 m
Carrier Gas: N2
Column Diameter: 0.25
mm
Phase Thickness: 1 um
Data Type: Linear RI
Program Type: Ramp
Start T: 30 C
End T: 200 C
Heat
Rate: 5 K/min
End Time: 30 min
Source: Wu, C.-M.; Wang, Z.; Wu, Q.H., Volatile compounds produced from monosodium glutamate in common food cooking, J. Agric. Food Chem., 48, 2000, 2438-2442.

<...>

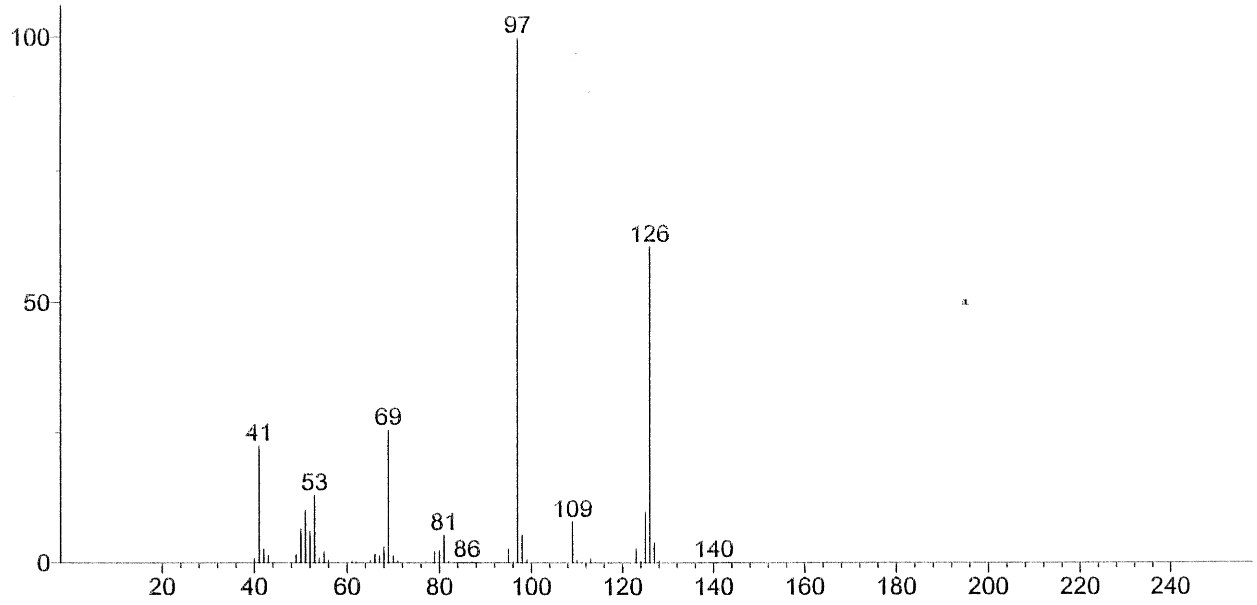

(Text File) +EI Scan (23.786 min) ALI-DRAG-H4-220421-.D Subtract

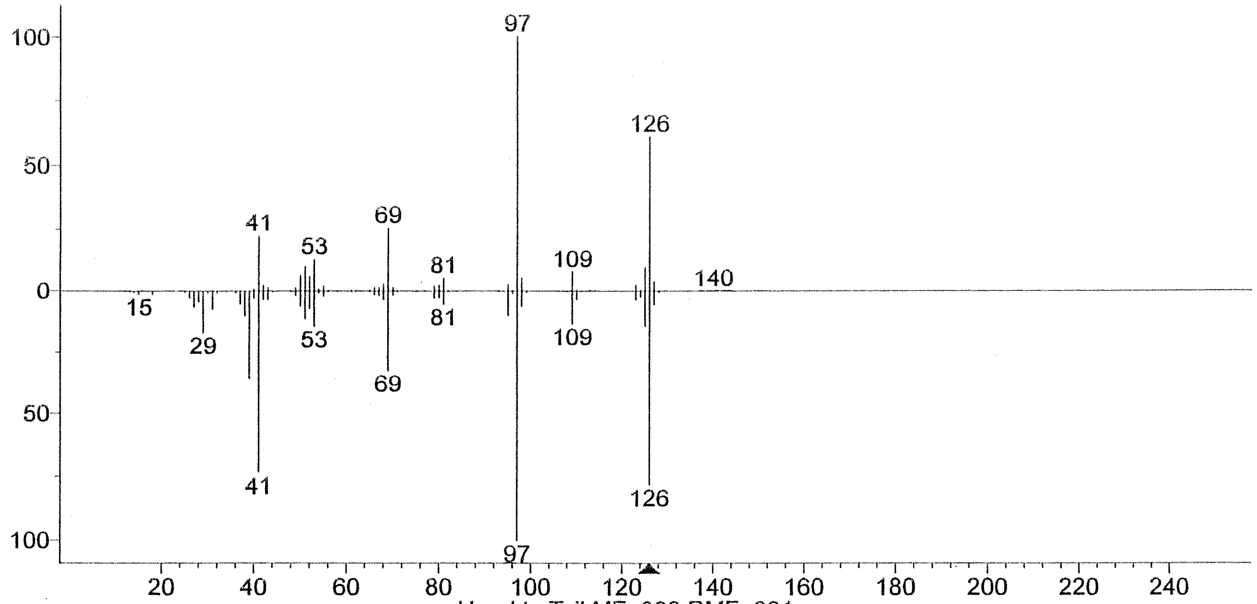

Head to Tail MF=899 RMF=901

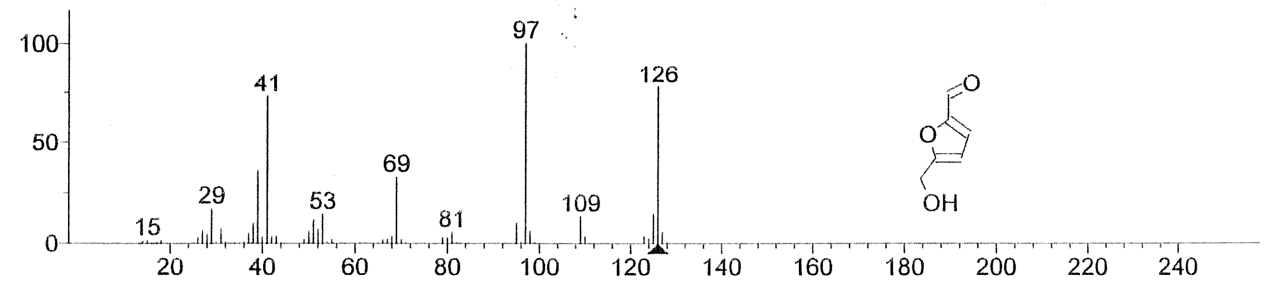

(mainlib) 2-Furancarboxaldehyde, 5-(hydroxymethyl)-

Name: 2-Furancarboxaldehyde, 5-(hydroxymethyl)-
Formula: $C_6H_6O_3$
MW: 126 CAS#: 67-47-0 NIST#: 231276 ID#: 60271 DB: mainlib
Other DBs: Fine, TSCA, RTECS, HODOC, NIH, EINECS
Contributor: Japan AIST/NIMC Database- Spectrum MS-NW-4745
10 largest peaks:
97 999 | 126 779 | 41 732 | 39 358 | 69 326 | 29 171 | 53 145 | 125 145 | 109 134 | 51 115 |
Synonyms:
1.2-Furaldehyde, 5-(hydroxymethyl)-
2.5-Hydrxoymethylfurfural
3.Hydroxymethylfurfurole
4.HMF
5.5-(Hyddroxymethyl)Furfurole
6.5-(Hydroxymethyl)-2-formylfuran
7.5-(Hydroxymethyl)-2-furaldehyde
8.5-(Hydroxymethyl)-2-furancarbonal
9.5-(Hydroxymethyl)-2-furfural
10.5-(Hydroxymethyl)-2-furfuraldehyde
11.5-(Hydroxymethyl)furan-2-aldehyde
12.5-(Hydroxymethyl)furfural
13.5-Hydroxymethylfuraldehyde
14.5-Oxymethylfurfurole
15.5-Hydroxymethylfurfuraldehyde
16.5-Hydroxymethyl-2-furancarbaldehyde
17.Hydroxymethylfurfuraldehyde
18.5-(Hydroxymethyl)-2-furancarboxaldehyde
19.2-Hydroxymethyl-5-furfural

Estimated non-polar retention index (n-alkane scale):
Value: 1163 iu
Confidence interval (Diverse functional groups): 89(50%) 382(95%) iu

Retention index.
1. Value: 1176 iu
Column Type: Capillary
Column Class: Standard non-polar
Active Phase: DB-1
Column
Length: 60 m
Carrier Gas: N2
Column Diameter: 0.25 mm
Phase Thickness: 1 um
Data Type: Linear
RI
Program Type: Ramp
Start T: 30 C
End T: 200 C
Heat Rate: 5 K/min
End Time: 30 min
Source: Wu, C.-M.;
Wang, Z.; Wu, Q.H., Volatile compounds produced from monosodium glutamate in common food cooking, J. Agric. Food Chem., 48, 2000, 2438-2442.

2. Value: 1208 iu

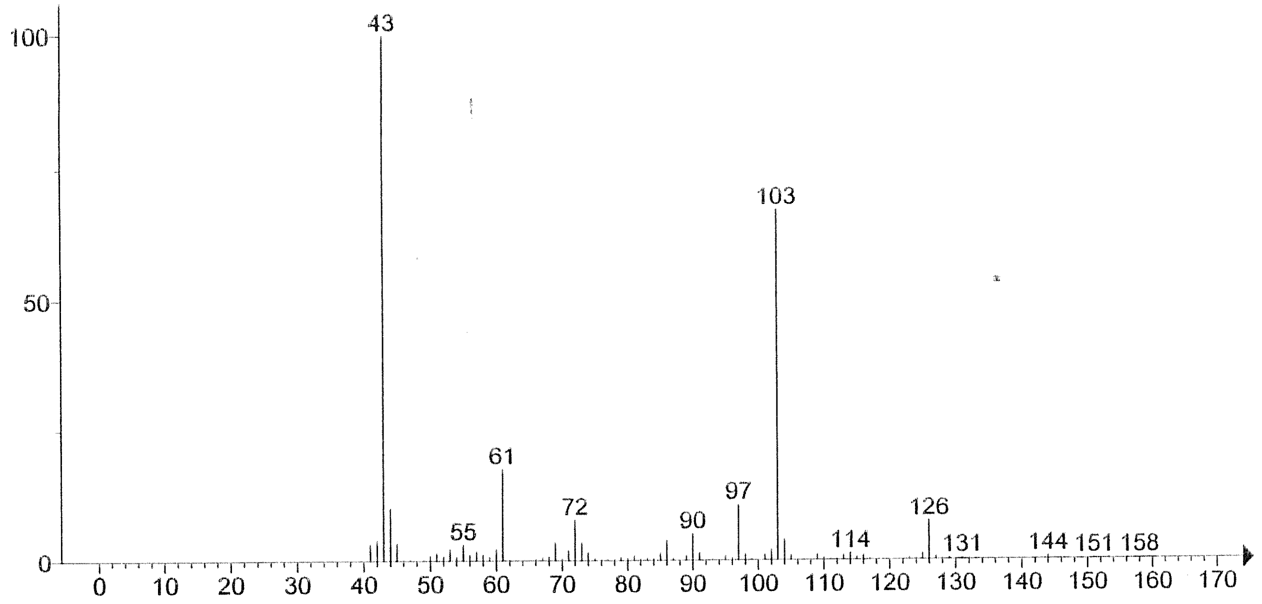

(Text File) +EI Scan (24.264 min) ALI-DRAG-H4-220421-.D

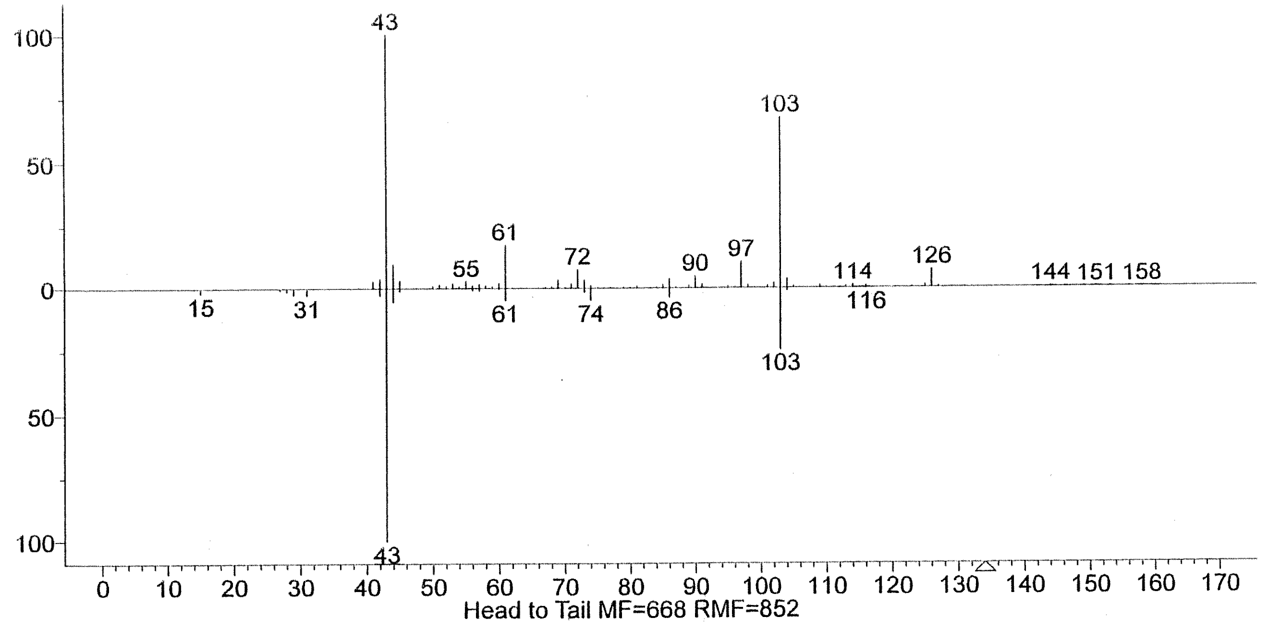

Head to Tail MF=668 RMF=852

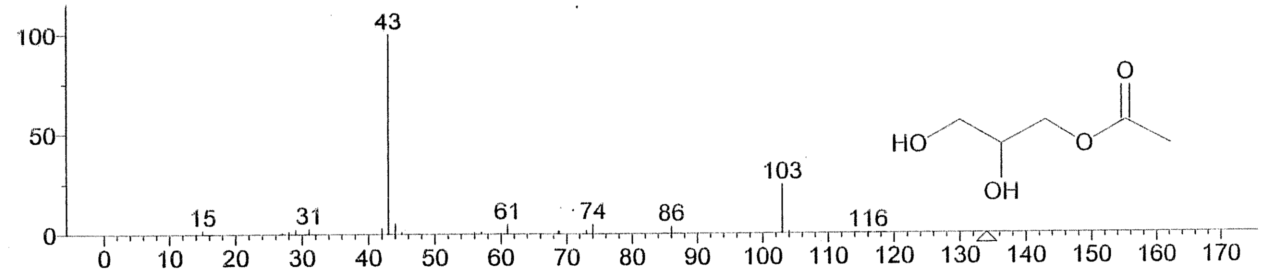

(replib) 1,2,3-Propanetriol, monoacetate

Name: 1,2,3-Propanetriol, monoacetate
Formula: $C_5H_{10}O_4$
MW: 134 CAS#: 26446-35-5 NIST#: 76112 ID#: 2817 DB: replib
Other DBs: Fine, TSCA, RTECS, EINECS
Contributor: RADIAN CORP
10 largest peaks:
43 999 | 103 243 | 44 55 | 61 48 | 74 48 | 86 37 | 42 29 | 31 28 | 29 26 | 15 20 |
Synonyms:
1.Acetin, mono-
2.Acetin
3.Acetoglyceride
4.Acetyl monoglyceride
5.Glycerin monoacetate
6.Glycerol acetate
7.Glycerol monoacetate
8.Glyceryl acetate
9.Glyceryl monoacetate
10.Monoacetin
11.Myvacet
12.Glycerol 1-acetate
13.1-Monoacetin
14.Acetic acid, monoglyceraldehyde
15.Alpha-monoacetin
16.Glycerol α-monoacetate
17.Hallco C-918
18.Monacetin
19.2,3-Dihydroxypropyl acetate #

Estimated non-polar retention index (n-alkane scale):
Value: 1091 iu
Confidence interval (Diverse functional groups): 89(50%) 382(95%) iu

Retention index.
1. Value: 2250 iu
Column Type: Capillary
Column Class: Standard polar
Active Phase: DB-FFAP
Column
Length: 30 m
Carrier Gas: H2
Column Diameter: 0.32 mm
Phase Thickness: 0.25 um
Data Type: Linear
RI
Program Type: Ramp
Start T: 40 C
End T: 240 C
Heat Rate: 5 K/min
Start Time: 2 min
Source: Charles, M.
; Martin, B.; Ginies, C.; Etievant, P.; Coste, G.; Guichard, E., Potent aroma compounds of two red wine vinegars, J. Agric. Food Chem., 48, 2000, 70-77.

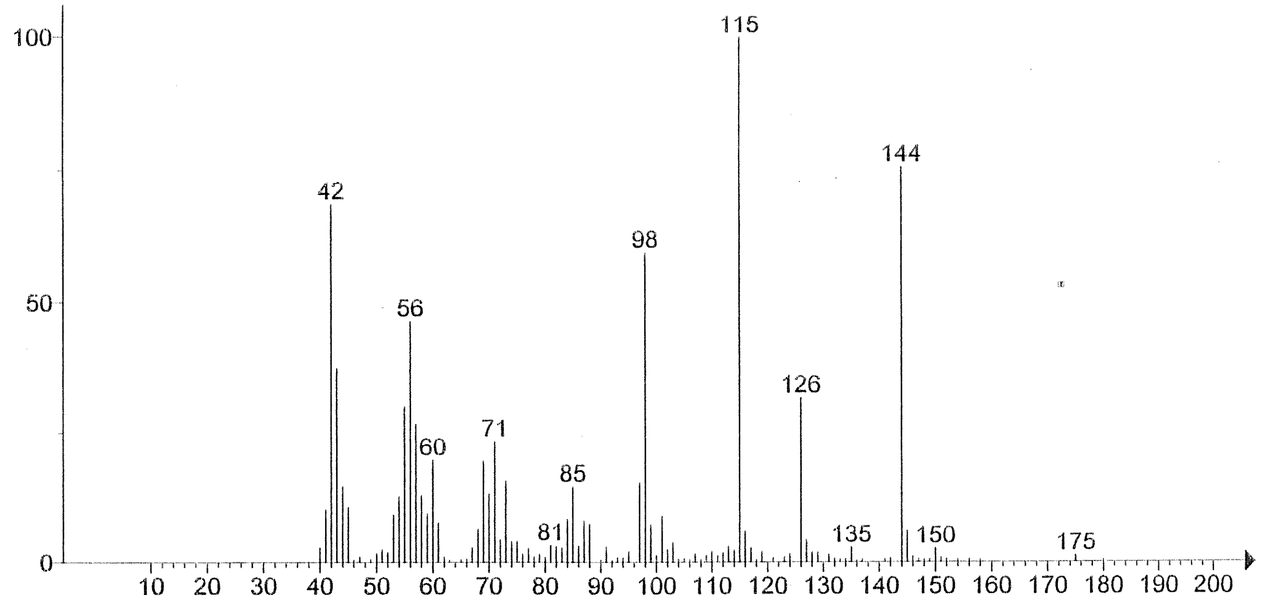

(Text File) +EI Scan (26.905 min) ALI-DRAG-H4-220421-.D

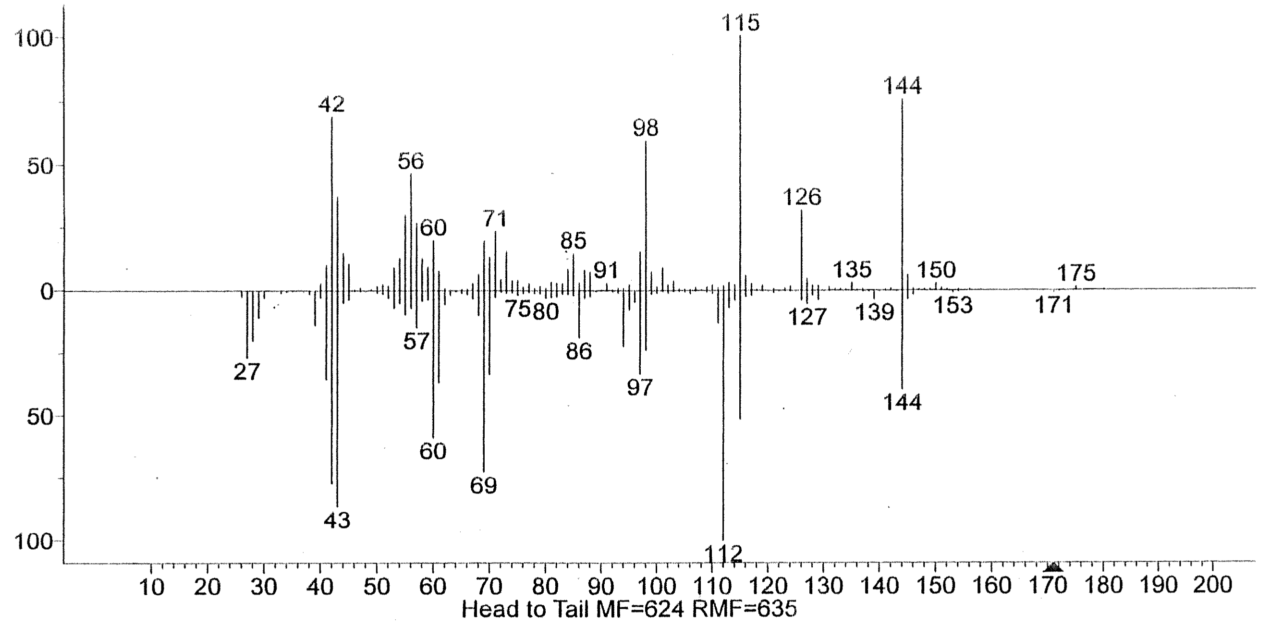

Head to Tail MF=624 RMF=635

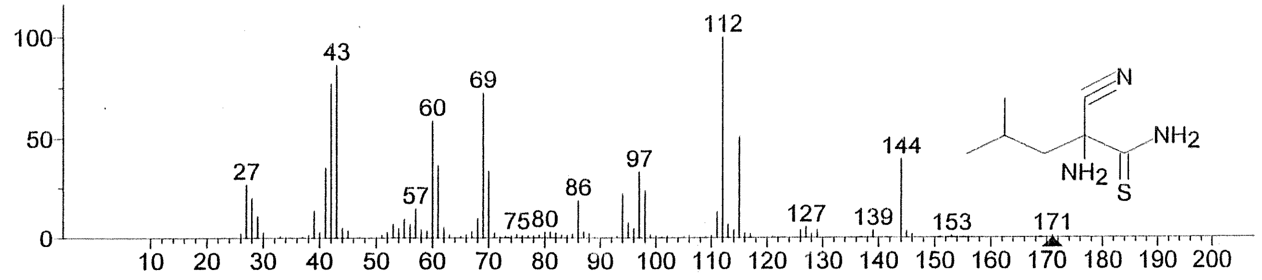

(mainlib) 2-Amino-2-cyano-4-methylpentanethioamide

Name: 2-Amino-2-cyano-4-methylpentanethioamide
Formula: $C_7H_{13}N_3S$
MW: 171 NIST#: 185735 ID#: 75633 DB: mainlib
Contributor: Chemical Concepts
10 largest peaks:
112 999 | 43 862 | 42 770 | 69 724 | 60 586 | 115 512 | 144 394 | 61 367 | 41 353 | 70 334 |
Synonyms:
no synonyms.

Estimated non-polar retention index (n-alkane scale):
Value: 1686 iu
Confidence interval (Diverse functional groups): 89(50%) 382(95%) iu

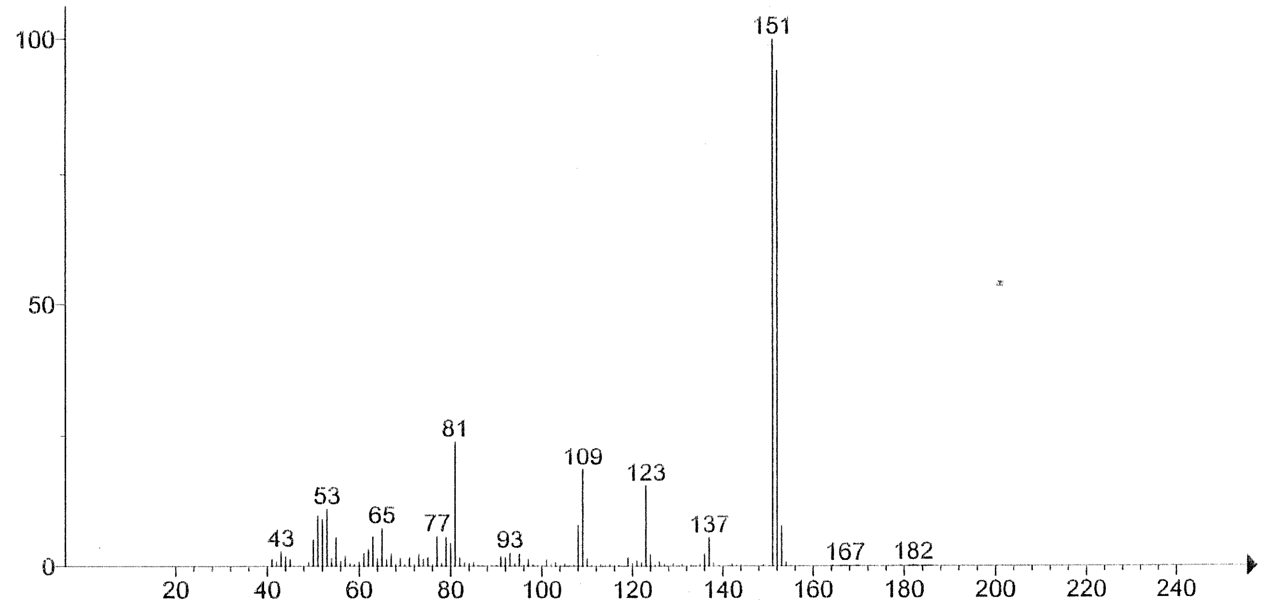

(Text File) +EI Scan (29.805 min) ALI-DRAG-H4-220421-.D

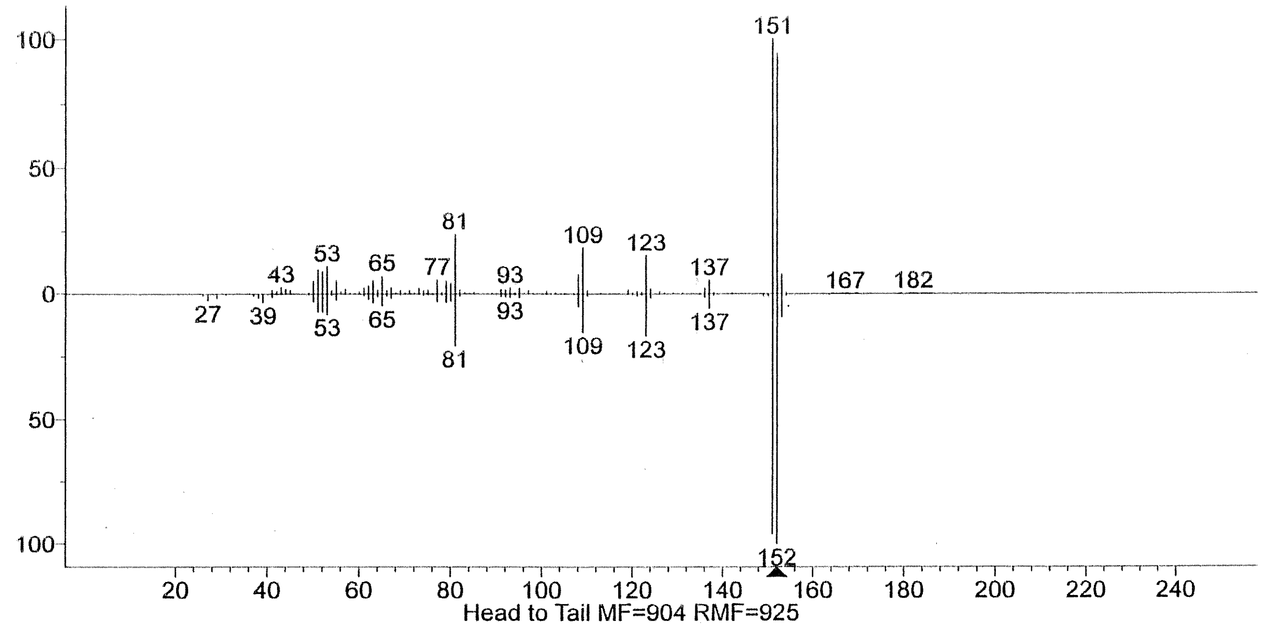

Head to Tail MF=904 RMF=925

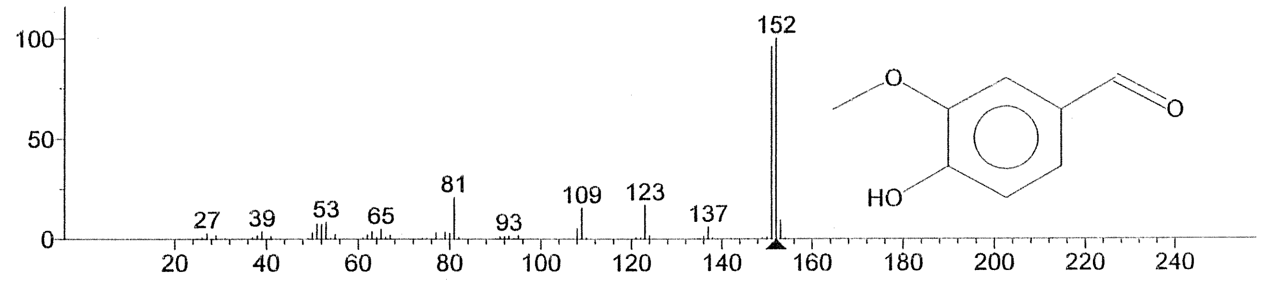

(replib) Vanillin

Name: Vanillin
Formula: $C_8H_8O_3$
MW: 152 CAS#: 121-33-5 NIST#: 192567 ID#: 20417 DB: replib
Other DBs: Fine, TSCA, RTECS, EPA, USP, HODOC, NIH, EINECS, IRDB
Contributor: Chemical Concepts
10 largest peaks:
152 999 | 151 957 | 81 206 | 123 167 | 109 152 | 153 92 | 53 81 | 51 72 | 52 71 | 137 58 |
Synonyms:
1.Benzaldehyde, 4-hydroxy-3-methoxy-
2.p-Hydroxy-m-methoxybenzaldehyde
3.Lioxin
4.Vanillaldehyde
5.Vanillic aldehyde
6.2-Methoxy-4-formylphenol
7.3-Methoxy-4-hydroxybenzaldehyde
8.4-Formyl-2-methoxyphenol
9.4-Hydroxy-3-methoxybenzaldehyde
10.4-Hydroxy-5-methoxybenzaldehyde
11.Vanilla
12.m-Anisaldehyde, 4-hydroxy-
13.Protocatechualdehyde, methyl-
14.Zimco
15.4-Hydroxy-m-anisaldehyde
16.p-Vanillin
17.Vanillin
18.m-Methoxy-p-hydroxybenzaldehyde
19.Methylprotcatechuic aldehyde
20.Methylprotocatechuic aldehyde

Estimated non-polar retention index (n-alkane scale):
Value: 1392 iu
Confidence interval (Diverse functional groups): 89(50%) 382(95%) iu

Retention index.
1. Value: 1403 iu
Column Type: Capillary
Column Class: Standard non-polar
Active Phase: BP-1
Column
Length: 50 m
Carrier Gas: He
Column Diameter: 0.22 mm
Phase Thickness: 0.25 um
Data Type: Linear
RI
Program Type: Ramp
Start T: 60 C
End T: 220 C
Heat Rate: 2 K/min
End Time: 20 min
Source:
Duquesnoy, E.; Dinh, N.H.; Castola, V.; Casanova, J., Composition of a Pyrolytic oil from Cupressus funebris Endl. of Vietnamese origin, Flavour Fragr. J., 21, 2006, 453-457.

30

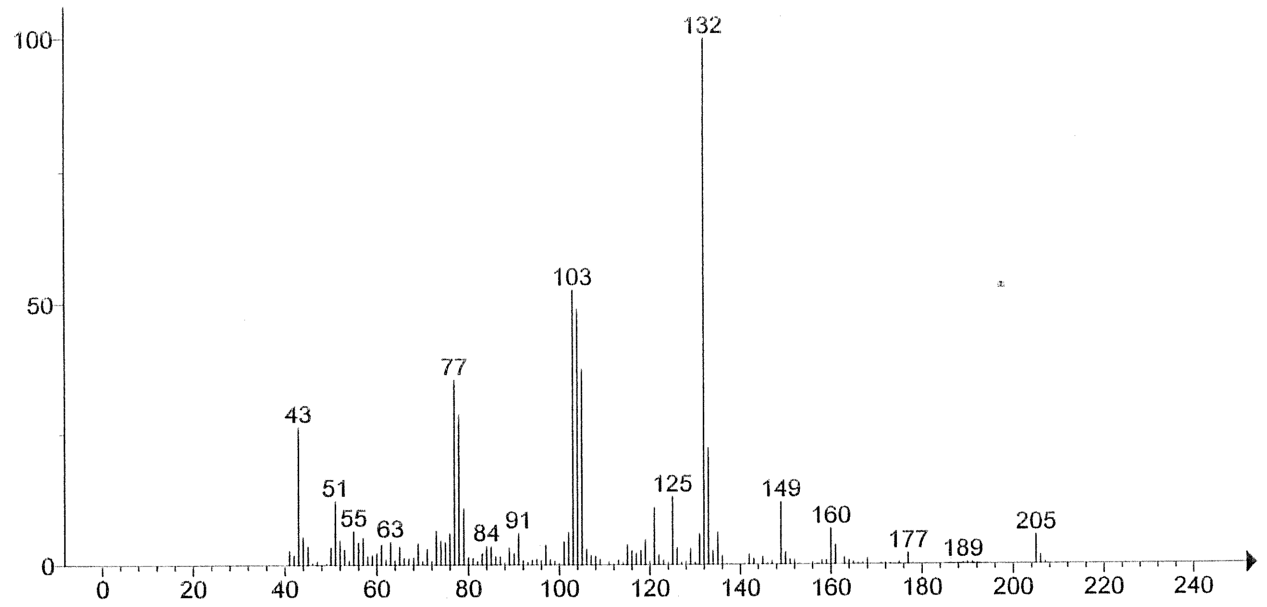

(Text File) +EI Scan (30.978 min) ALI-DRAG-H4-220421-.D

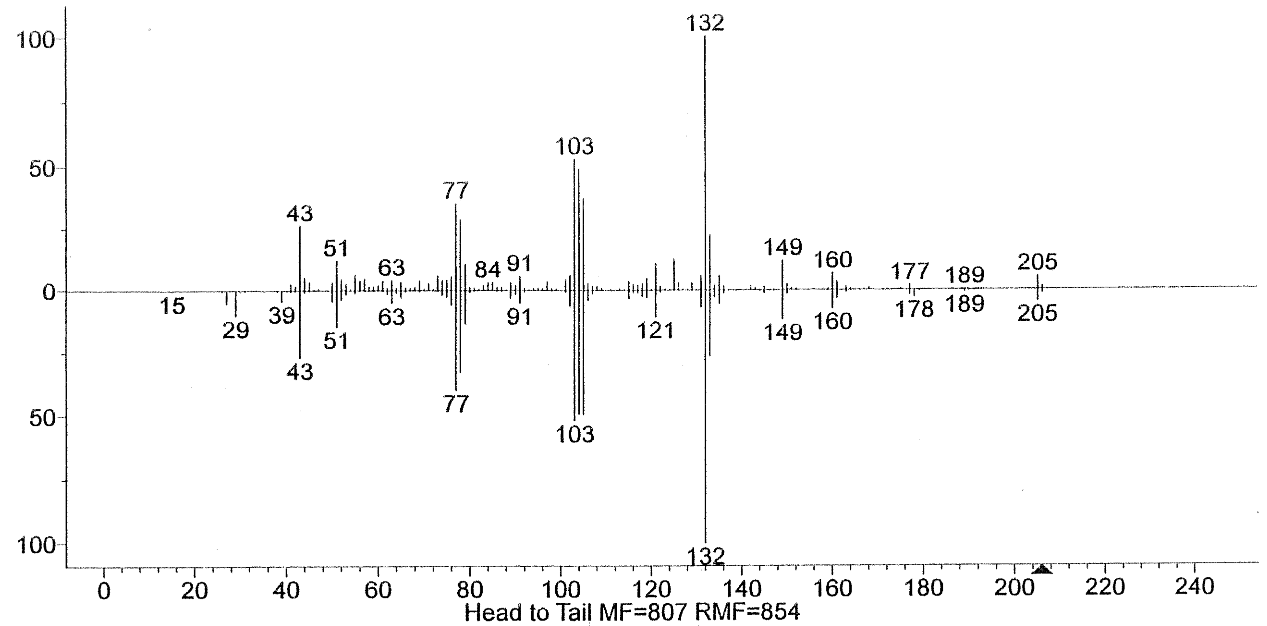

Head to Tail MF=807 RMF=854

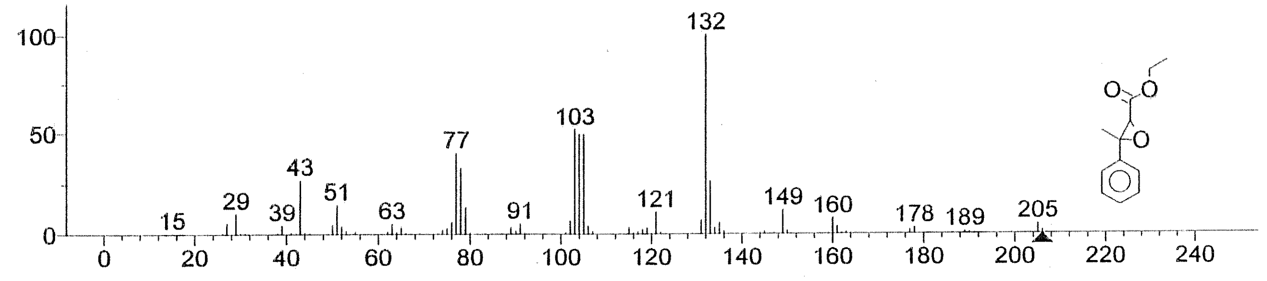

(mainlib) Oxiranecarboxylic acid, 3-methyl-3-phenyl-, ethyl ester, cis-

Name: Oxiranecarboxylic acid, 3-methyl-3-phenyl-, ethyl ester, cis-
Formula: $C_{12}H_{14}O_3$
MW: 206 CAS#: 19464-95-0 NIST#: 291516 ID#: 93991 DB: mainlib
Other DBs: NIH
Contributor: NIST Mass Spectrometry Data Center, 1998.
10 largest peaks:
132 999 | 103 517 | 105 494 | 104 493 | 77 399 | 78 327 | 43 268 | 133 264 | 51 146 | 79 134 |
Synonyms:
1.cis-Ethyl 3-methyl-3-phenylglycidate
2.Ethyl-3-methyl-3-phenylglycidate
3.Hydrocinnamic acid, α,β-epoxy-β-methyl-, ethyl ester, stereoisomer
4.cis-3-Methyl-3-phenylglycidic acid, ethyl ester
5.Ethyl 3-methyl-3-phenyl-2-oxiranecarboxylate #
6.cis-3-Phenyl-3-methylglycidic acid, ethyl ester

Estimated non-polar retention index (n-alkane scale):
Value: 1484 iu
Confidence interval (Diverse functional groups): 89(50%) 382(95%) iu

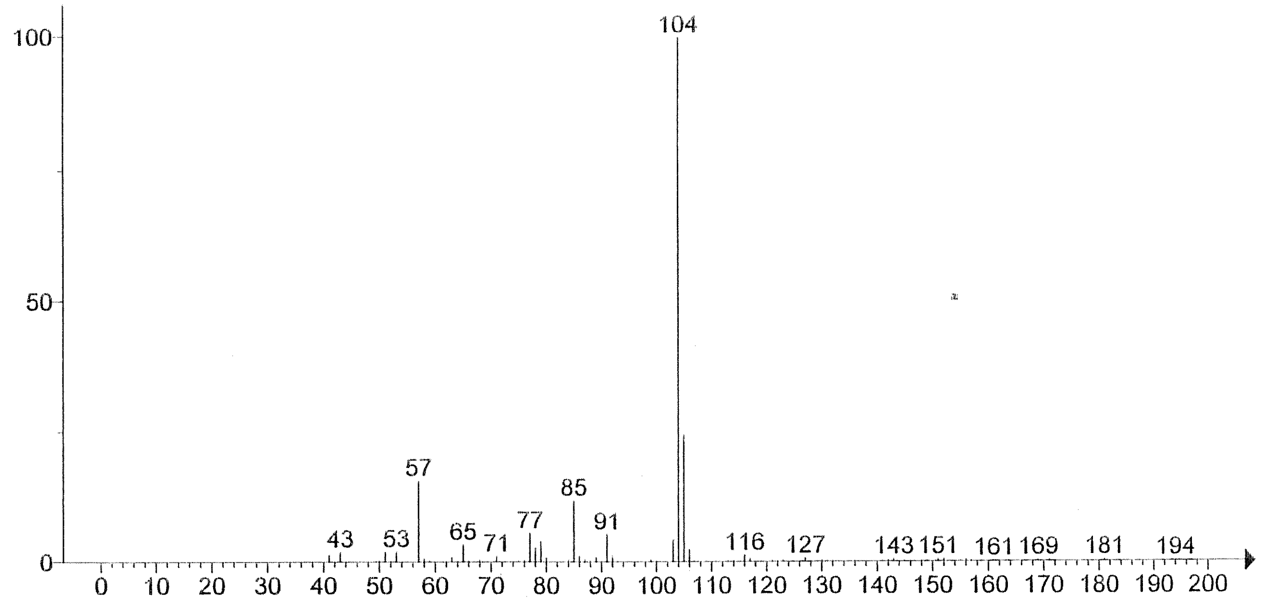

(Text File) +EI Scan (32.738 min) ALI-DRAG-H4-220421-.D Subtract

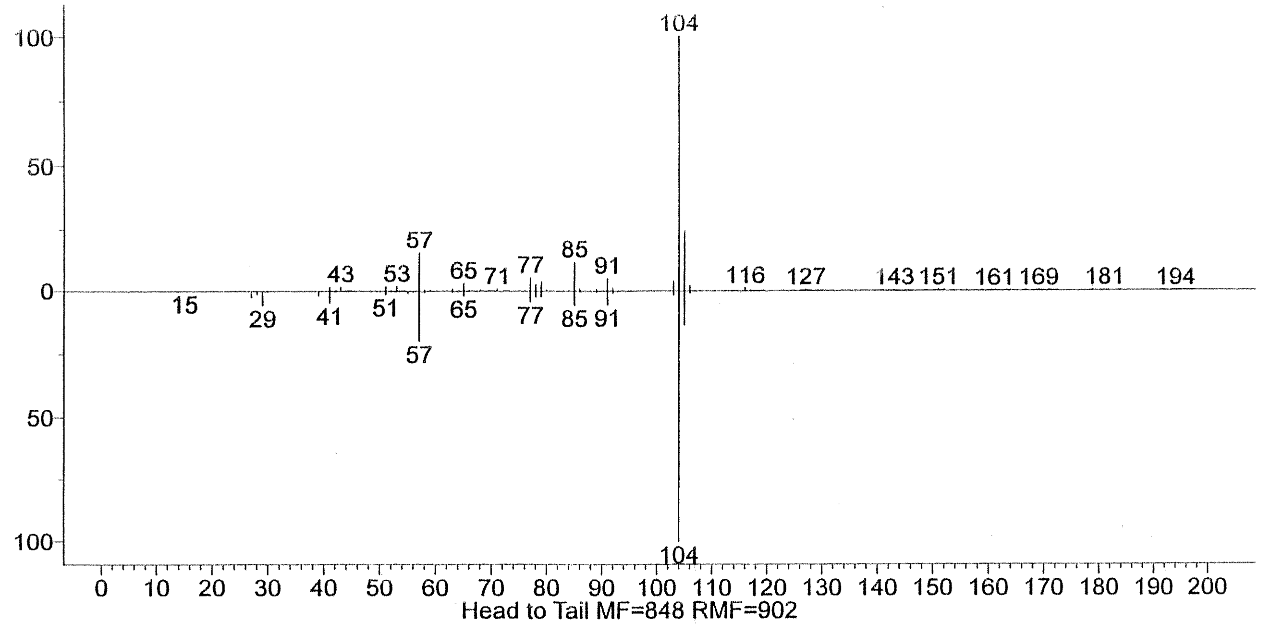

Head to Tail MF=848 RMF=902

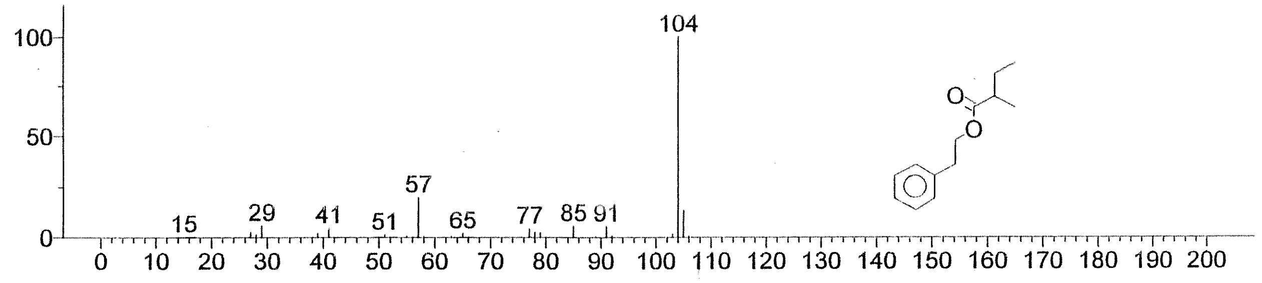

(mainlib) Butanoic acid, 2-methyl-, 2-phenylethyl ester

Name: Butanoic acid, 2-methyl-, 2-phenylethyl ester
Formula: $C_{13}H_{18}O_2$
MW: 206 CAS#: 24817-51-4 NIST#: 118042 ID#: 65225 DB: mainlib
Other DBs: TSCA, RTECS, EINECS
Contributor: Chuck Anderson, Aldrich Chemical Co.
10 largest peaks:
104 999 | 57 197 | 105 135 | 29 57 | 85 56 | 91 55 | 41 47 | 77 43 | 27 26 | 78 25 |
Synonyms:
1.Phenethyl 2-methylbutyrate
2.Phenylethyl 2-methyl butyrate
3.2-Phenylethyl 2-methylbutanoate #

Estimated non-polar retention index (n-alkane scale):
Value: 1493 iu
Confidence interval (Esters): 47(50%) 201(95%) iu

Retention index.
1. Value: 1460 iu
Column Type: Capillary
Column Class: Standard non-polar
Active Phase: RTX-1
Column
Length: 60 m
Carrier Gas: He
Column Diameter: 0.22 mm
Phase Thickness: 0.25 um
Data Type: Linear
RI
Program Type: Ramp
Start T: 60 C
End T: 230 C
Heat Rate: 2 K/min
End Time: 35 min
Source: Paolini, J.;
Costa, J.; Bernardini, A., Analysis of the essential oil from aerial parts of Eupatorium cannabinum subsp. corsicum (L.) by gas chromatography with electron impact and chemical ionization mass spectrometry, J. Chromatogr. A, 1076, 2005, 170-178.

2. Value: 1466 iu
Column Type: Capillary
Column Class: Standard non-polar
Active
Phase: DB-1
Column Length: 30 m
Carrier Gas: He
Phase Thickness: 0.25 um
Data Type: Normal alkane
RI
Program Type: Ramp
Start T: 50 C
End T: 220 C
Heat Rate: 6 K/min
Start Time: 2 min
End Time: 20

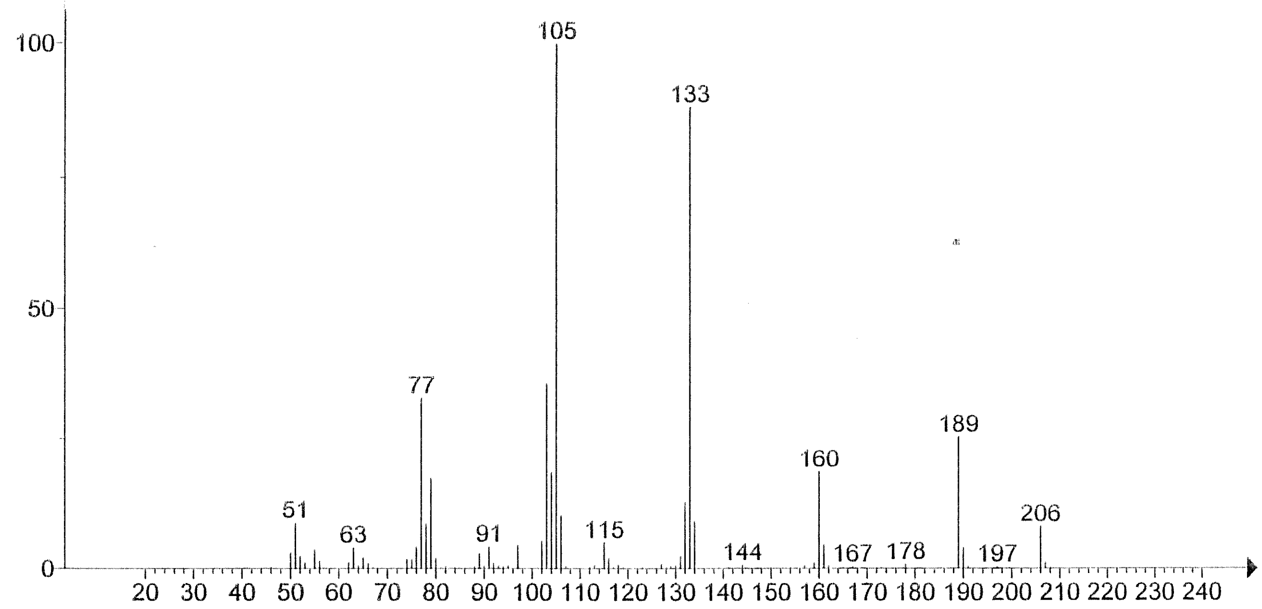

(Text File) +EI Scan (33.038 min) ALI-DRAG-H4-220421-.D Subtract

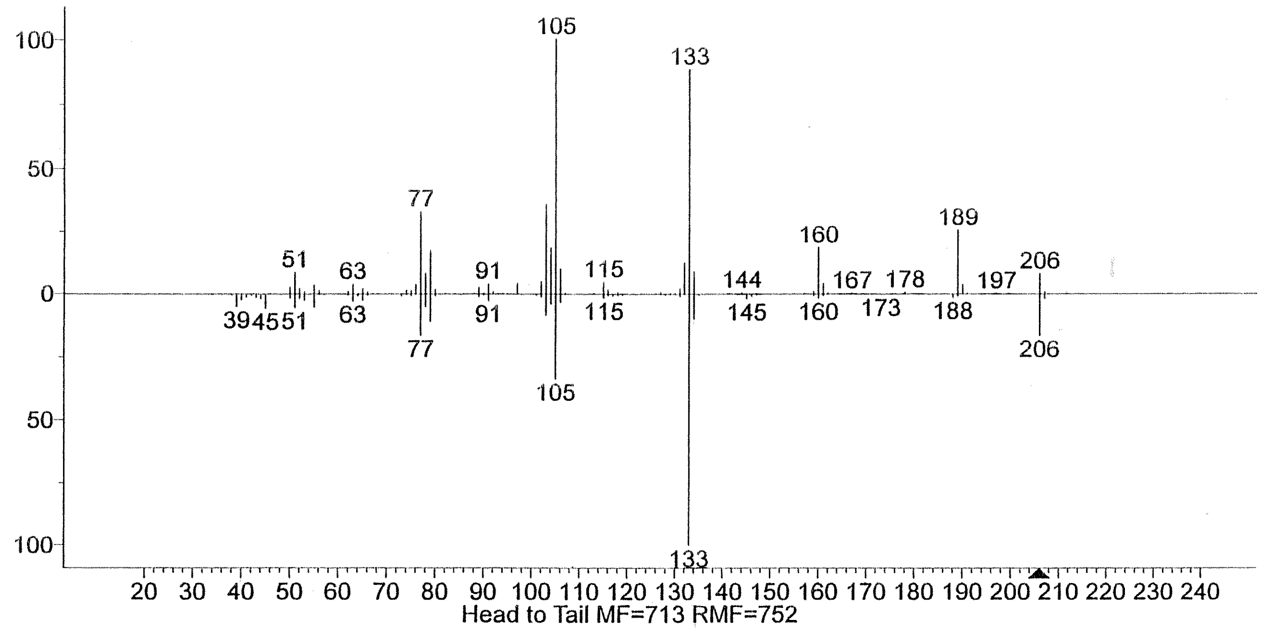

Head to Tail MF=713 RMF=752

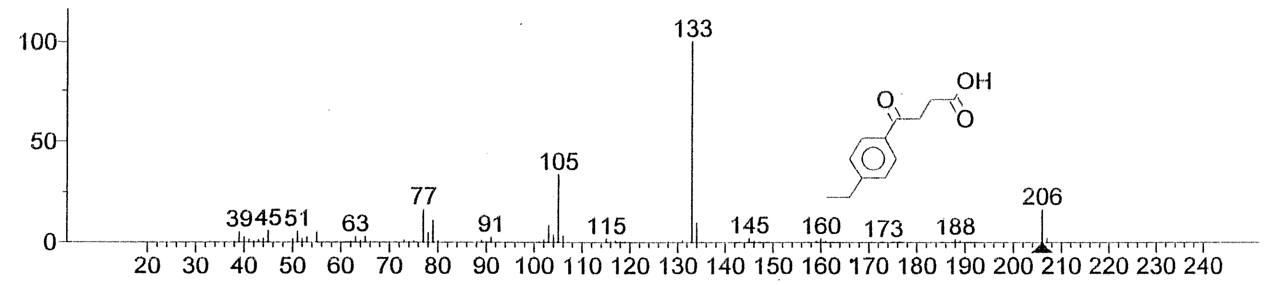

(mainlib) Benzenebutanoic acid, 4-ethyl-γ-oxo-

Name: Benzenebutanoic acid, 4-ethyl-γ-oxo-
Formula: $C_{12}H_{14}O_3$
MW: 206 CAS#: 49594-75-4 NIST#: 271880 ID#: 94812 DB: mainlib.
Other DBs: None
Contributor: A.A.Kutin, Moscow, Russia
10 largest peaks:
133 999 | 105 335 | 206 164 | 77 163 | 79 110 | 134 98 | 103 84 | 45 57 | 51 54 | 55 53 |
Synonyms:
1.3-(4-Ethylbenzoyl)propionic acid
2.4-(4-Ethylphenyl)-4-oxobutanoic acid

Estimated non-polar retention index (n-alkane scale):
Value: 1797 iu
Confidence interval (Diverse functional groups): 89(50%) 382(95%) iu

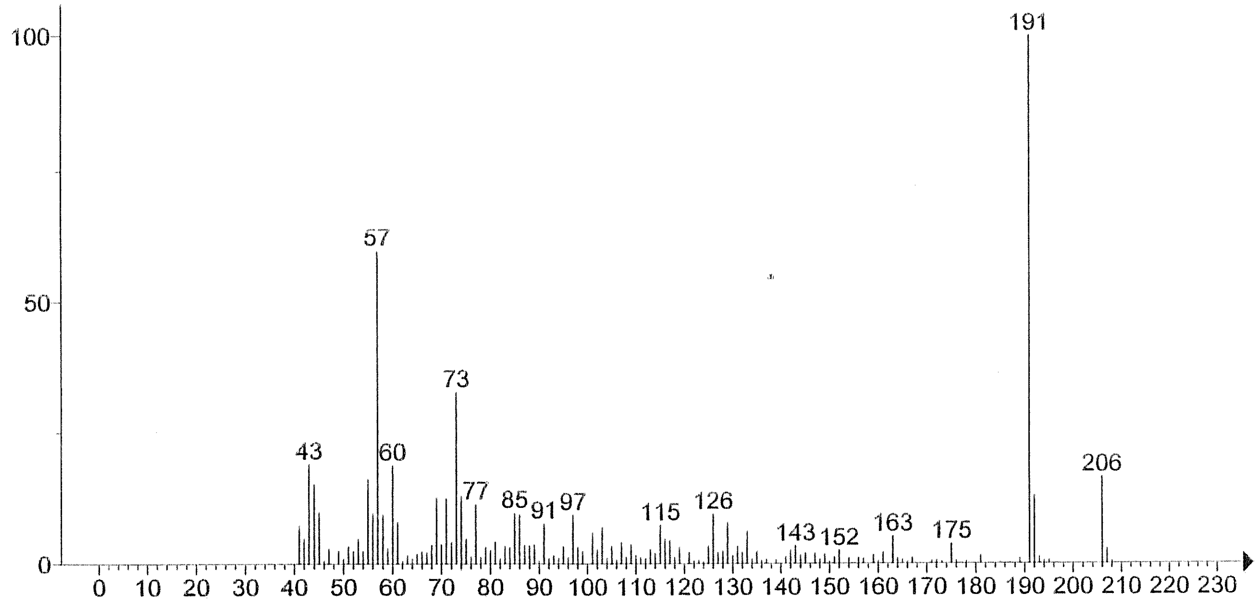

(Text File) +EI Scan (33.266 min) ALI-DRAG-H4-220421-.D

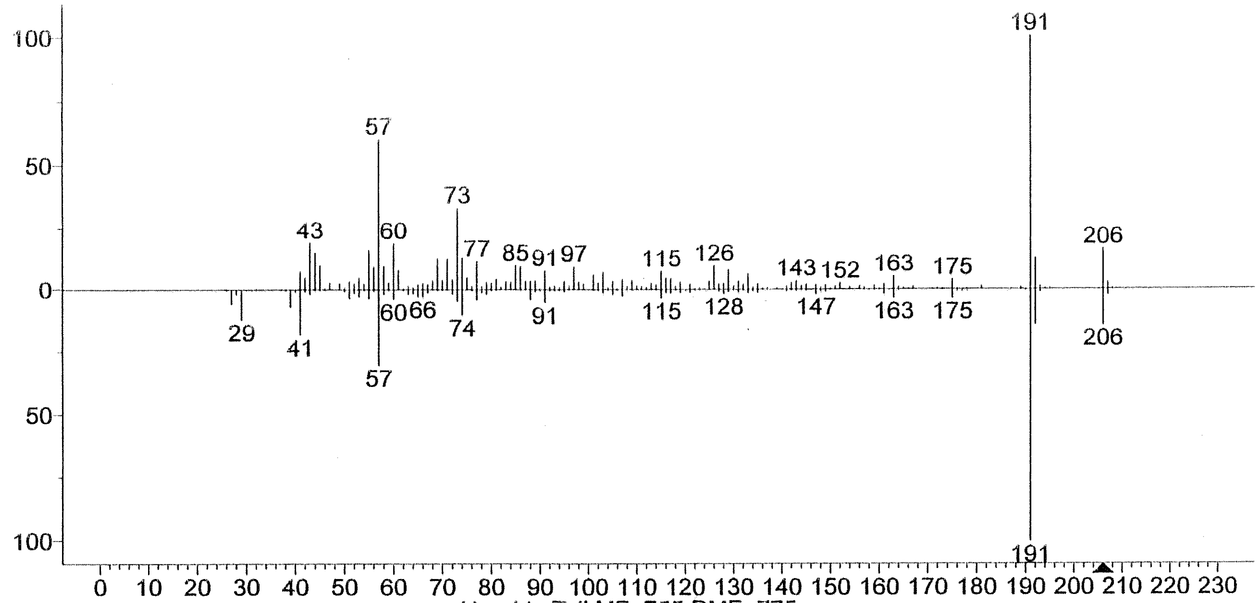

Head to Tail MF=755 RMF=775

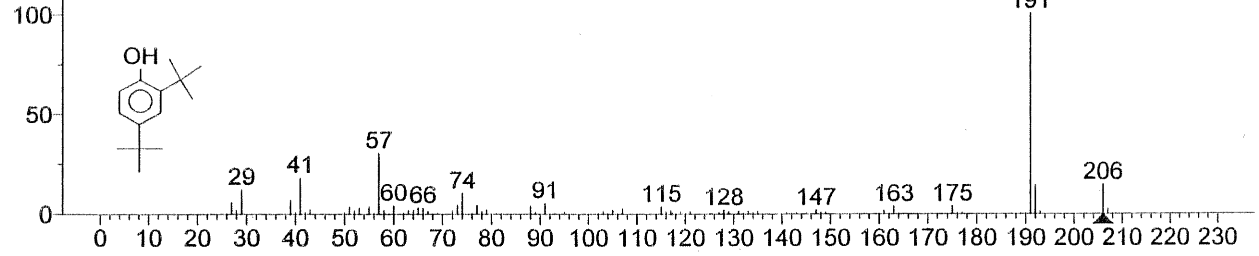

(replib) Phenol, 2,4-bis(1,1-dimethylethyl)-

Name: Phenol, 2,4-bis(1,1-dimethylethyl)-
Formula: $C_{14}H_{22}O$
MW: 206 CAS#: 96-76-4 NIST#: 22572 ID#: 23668 DB: replib
Other DBs: Fine, TSCA, RTECS, EPA, HODOC, NIH, EINECS, IRDB
10 largest peaks:
191 999 | 57 302 | 41 181 | 206 145 | 192 143 | 29 121 | 74 102 | 39 71 | 27 58 | 91 53 |
Synonyms:
1.Phenol, 2,4-di-tert-butyl-
2.2,4-Di-tert-butylphenol
3.2,4-di-t-Butylphenol
4.1-Hydroxy-2,4-di-tert-butylbenzene
5.Antioxidant No. 33
6.Prodox 146
7.Prodox 146A-85X
8.2,4-Bis(1,1-dimethylethyl)phenol

Estimated non-polar retention index (n-alkane scale):
Value: 1555 iu
Confidence interval (Phenols): 70(50%) 301(95%) iu

Retention index.
1. Value: 1539 iu
Column Type: Packed
Column Class: Standard non-polar
Active Phase: SE-30
Column
Length: 2 m
Substrate: GasChrom Q
Data Type: Linear RI
Program Type: Ramp
Start T: 80 C
End T: 250
C
Heat Rate: 4 K/min
Source: Staniewski, J., Gas chromatographic analysis of some hydroxyoxime extractants of metals, Chem. Anal. (Warsaw), 36(2), 1991, 325-333.

2. Value: 1519 iu
Column Type: Packed
Column Class:
Standard non-polar
Active Phase: SE-30
Column Length: 3 m
Carrier Gas: He
Substrate: N_AW_HMDS
Data
Type: Kovats RI
Program Type: Isothermal
Start T: 523 K
Source: Verevkin, S.P.; Nesterov, O.A.; Rempel, P.D.;
Synkova, N.V.; Shashkin, N.P., Chromatographic determination of the products of condensation of alkylphenols with formic aldehyde, Zh. Anal. Khim., 43, 1990, 760-761.

<...>

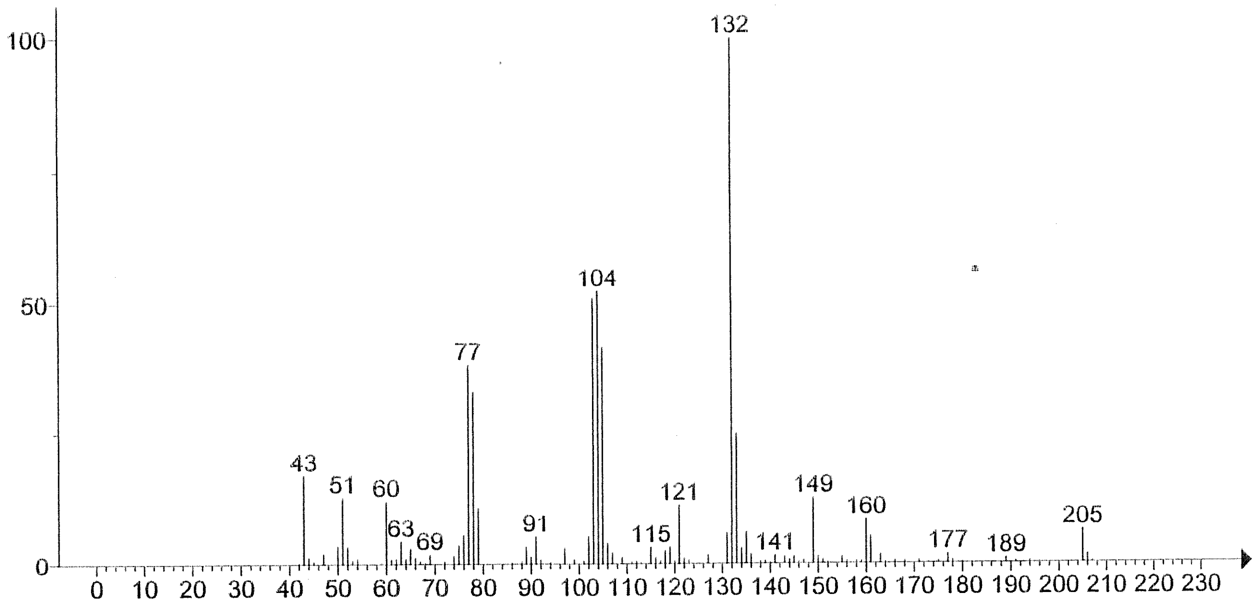

(Text File) +EI Scan (33.854 min) ALI-DRAG-H4-220421-.D Subtract

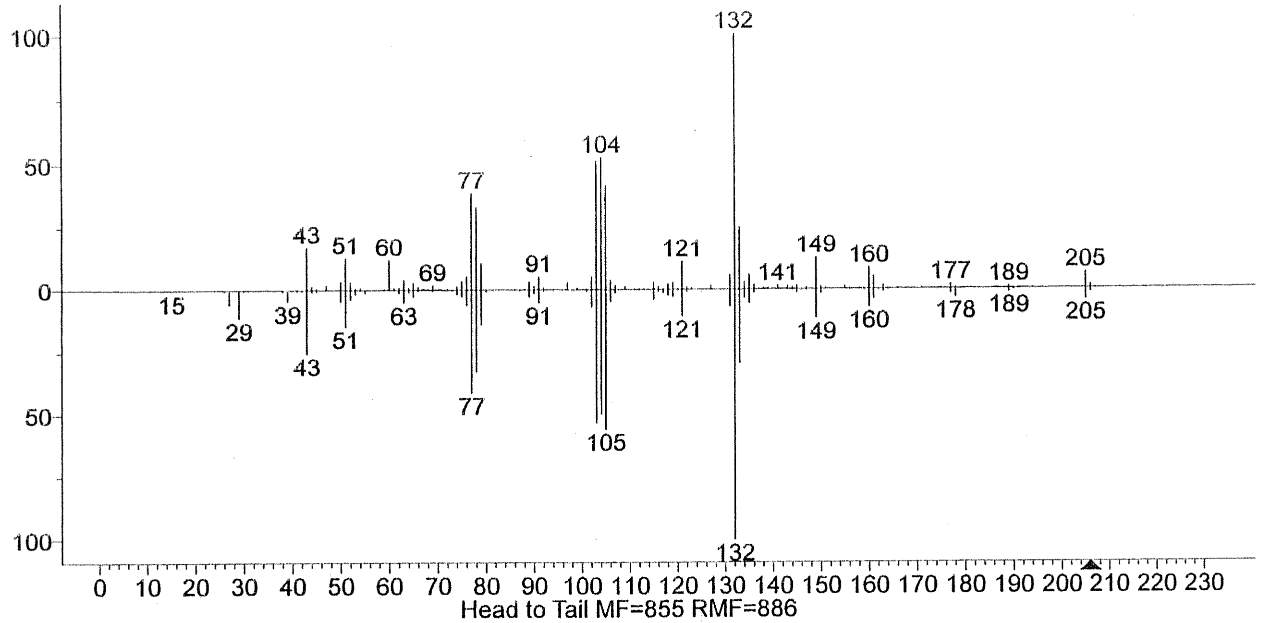

Head to Tail MF=855 RMF=886

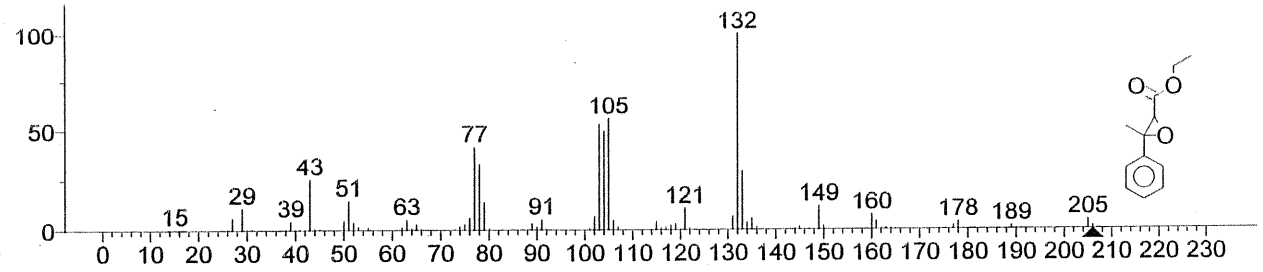

(mainlib) Oxiranecarboxylic acid, 3-methyl-3-phenyl-, ethyl ester, trans-

Name: Oxiranecarboxylic acid, 3-methyl-3-phenyl-, ethyl ester, trans-
Formula: $C_{12}H_{14}O_3$
MW: 206 CAS#: 19464-92-7 NIST#: 291517 ID#: 94061 DB: mainlib
Other DBs: None
Contributor: NIST Mass Spectrometry Data Center, 1998.
10 largest peaks:
132 999 | 105 557 | 103 531 | 104 496 | 77 413 | 78 327 | 133 293 | 43 253 | 51 145 | 79 138 |
Synonyms:
1.trans-Ethyl 3-Methyl-3-phenylglycidate
2.Hydrocinnamic acid, α,β-epoxy-β-methyl-, ethyl ester, trans-
3.Hydrocinnamic acid, α,β-epoxy-β-methyl-, ethyl ester, stereoisomer
4.trans-3-Methyl-3-phenylglycidic acid, ethyl ester
5.Ethyl 3-methyl-3-phenyl-2-oxiranecarboxylate #
6.trans-3-Phenyl-3-methylglycidic acid, ethyl ester

Estimated non-polar retention index (n-alkane scale):
Value: 1484 iu
Confidence interval (Diverse functional groups): 89(50%) 382(95%) iu

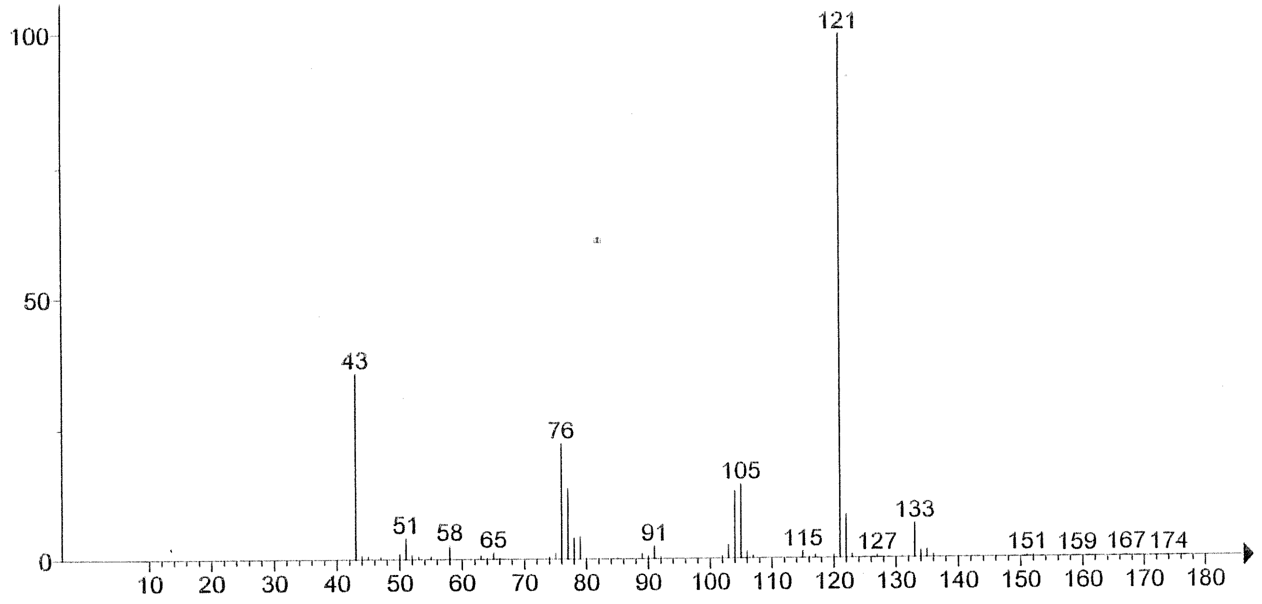

(Text File) +EI Scan (35.133 min) ALI-DRAG-H4-220421-.D Subtract

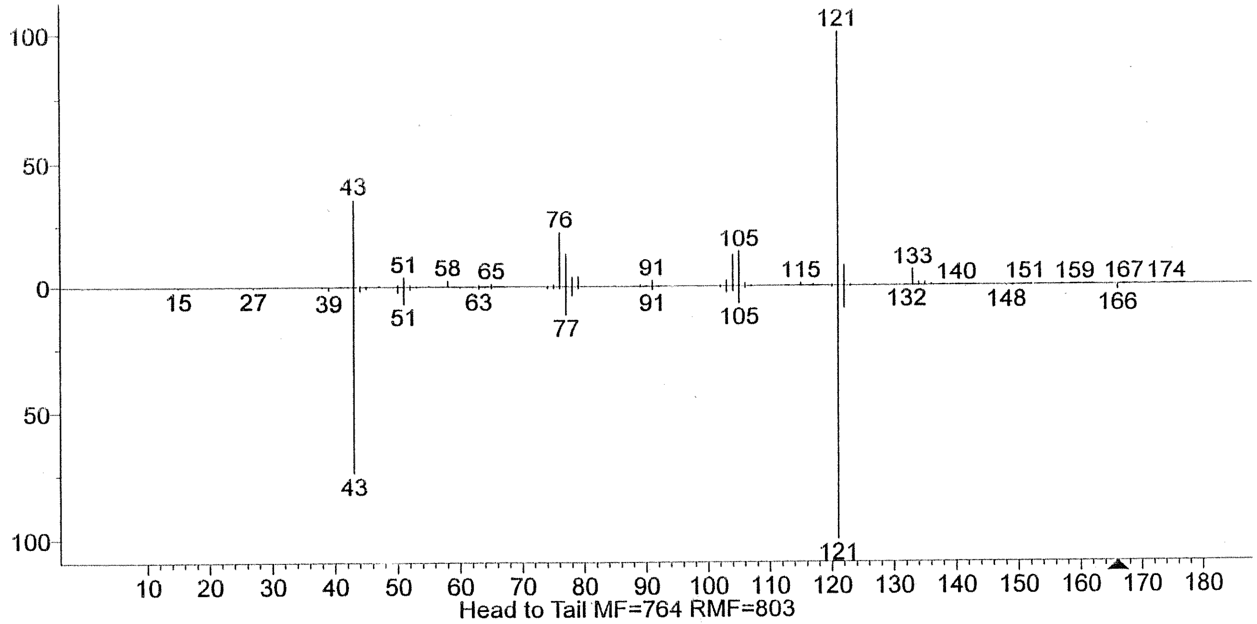

Head to Tail MF=764 RMF=803

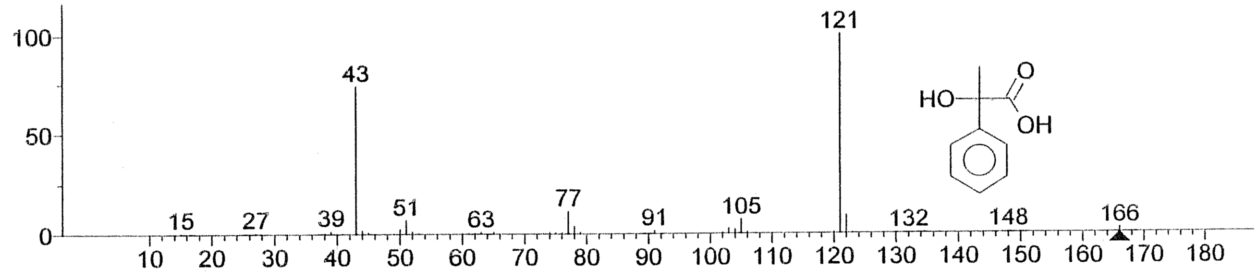

(mainlib) Benzeneacetic acid, α-hydroxy-α-methyl-, (±)-

Name: Benzeneacetic acid, α-hydroxy-α-methyl-, (±)-
Formula: $C_9H_{10}O_3$
MW: 166 CAS#: 4607-38-9 NIST#: 244949 ID#: 82980 DB: mainlib
Other DBs: TSCA, HODOC, NIH, EINECS
Contributor: Japan AIST/NIMC Database- Spectrum MS-IW-5150
10 largest peaks:
121 999 | 43 738 | 77 114 | 122 91 | 105 70 | 51 69 | 78 37 | 103 26 | 50 23 | 104 22 |
Synonyms:
1.DL-α-Phenyllactic acid
2.DL-Atrolactic acid
3.Mandelic acid, α-methyl-, DL-
4.Benzeneacetic acid, α-hydroxy-α-methyl-, (.+-.)-
5.2-Hydroxy-2-phenylpropanoic acid #

Estimated non-polar retention index (n-alkane scale):
Value: 1441 iu
Confidence interval (Diverse functional groups): 89(50%) 382(95%) iu

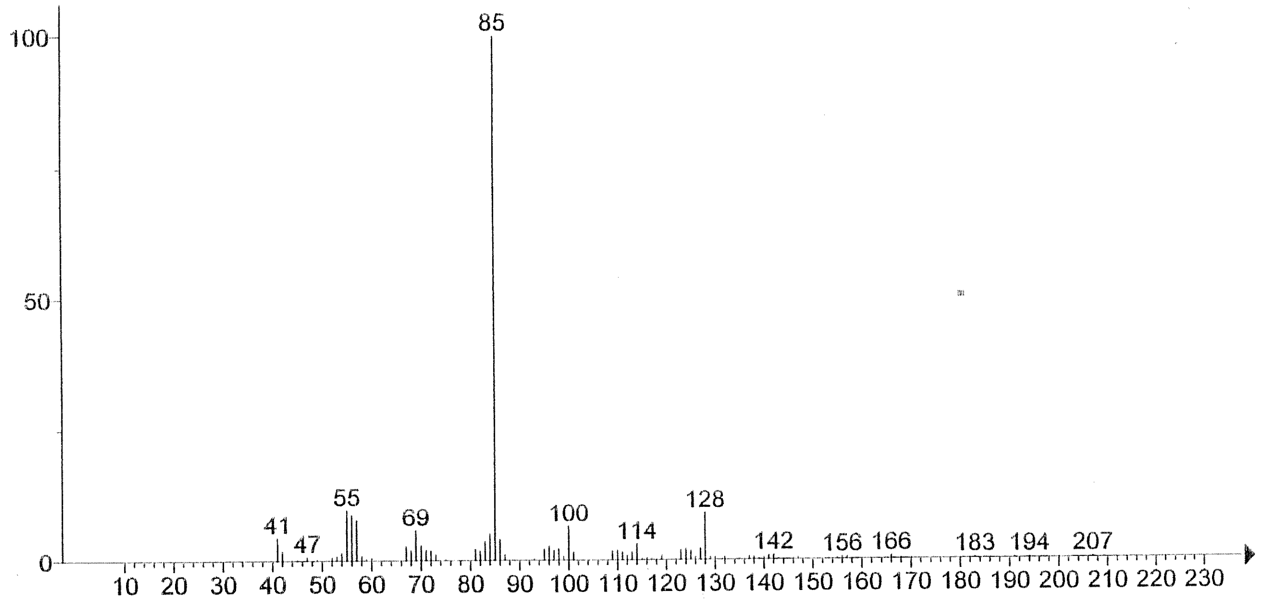

(Text File) +EI Scan (35.226 min) ALI-DRAG-H4-220421-.D Subtract

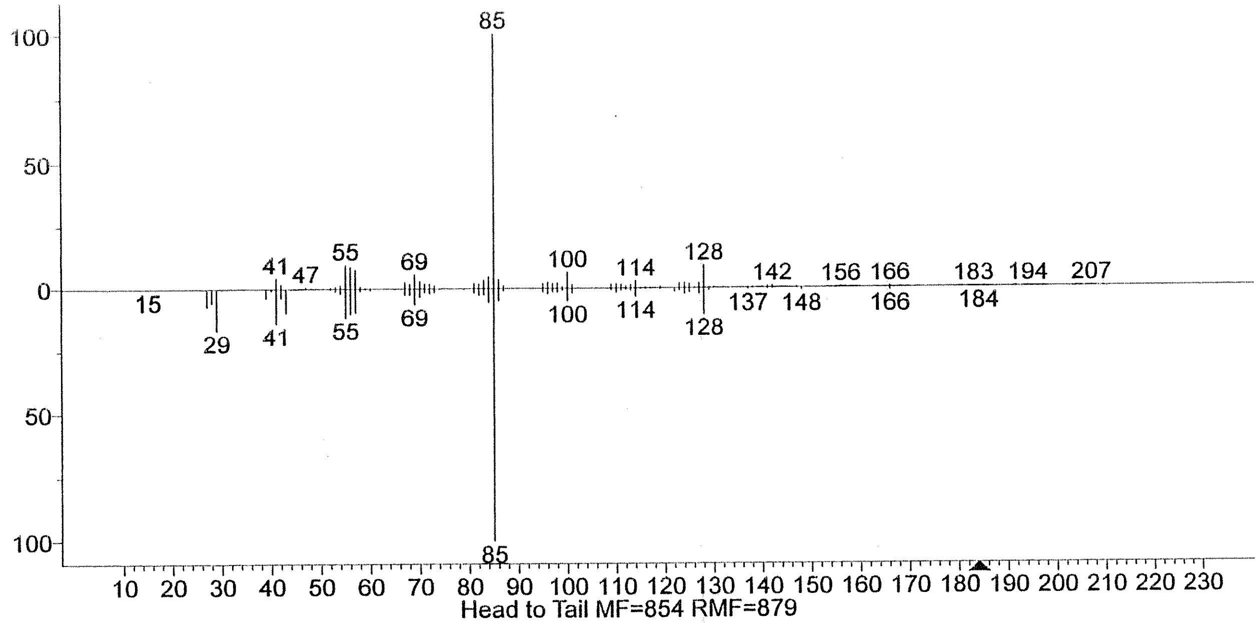

Head to Tail MF=854 RMF=879

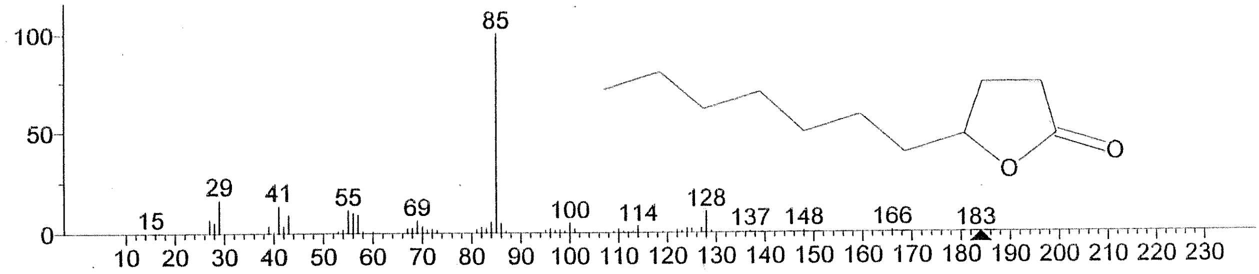

(mainlib) 2(3H)-Furanone, 5-heptyldihydro-

Name: 2(3H)-Furanone, 5-heptyldihydro-
Formula: $C_{11}H_{20}O_2$
MW: 184 CAS#: 104-67-6 NIST#: 7793 ID#: 46596 DB: mainlib
Other DBs: Fine, TSCA, RTECS, HODOC, NIH, EINECS
10 largest peaks:
85 999 | 29 165 | 41 137 | 55 115 | 128 104 | 56 100 | 43 94 | 57 93 | 27 70 | 69 62 |
Synonyms:
1.δ-Undecalactone
2.γ-n-Heptylbutyrolactone
3.γ-Heptyl-γ-butyrolactone
4.γ-Heptylbutyrolactone
5.γ-Undecalactone
6.γ-Undecanolactone
7.γ-Undecanolide
8.Peach lactone
9.Persicol
10.Undecanoic acid, 4-hydroxy-, γ-lactone
11.4-Hydroxyundecanoic acid lactone
12.Undecanoic γ-lactone
13.Undecanolide-1,4
14.γ-Undecalatone
15.Aldehyde C-14
16.Aldehyde C-14 peach
17.Peach aldehyde
18.1,4-Undecanolide
19.4-Hydroxyundecanoic acid, γ-lactone
20.4-Undecanolide
21.γ-Undekalakton
22.5-Heptyldihydro-2(3H)-furanone #

Estimated non-polar retention index (n-alkane scale):
Value: 1483 iu
Confidence interval (Esters): 47(50%) 201(95%) iu

Retention index.
1. Value: 1521.0 iu
Column Type: Capillary
Column Class: Standard non-polar
Active Phase: DB-1
Column
Length: 30 m
Carrier Gas: He
Column Diameter: 0.25 mm
Phase Thickness: 0.25 um
Data Type: Linear
RI
Program Type: Ramp
Start T: 40 C
End T: 325 C
Heat Rate: 3 K/min
Source: Sun, G.; Stremple, P.,
Retention index characterization of flavor, fragrance, and many other compounds on DB-1 and DB-XLB, 2003.

2.

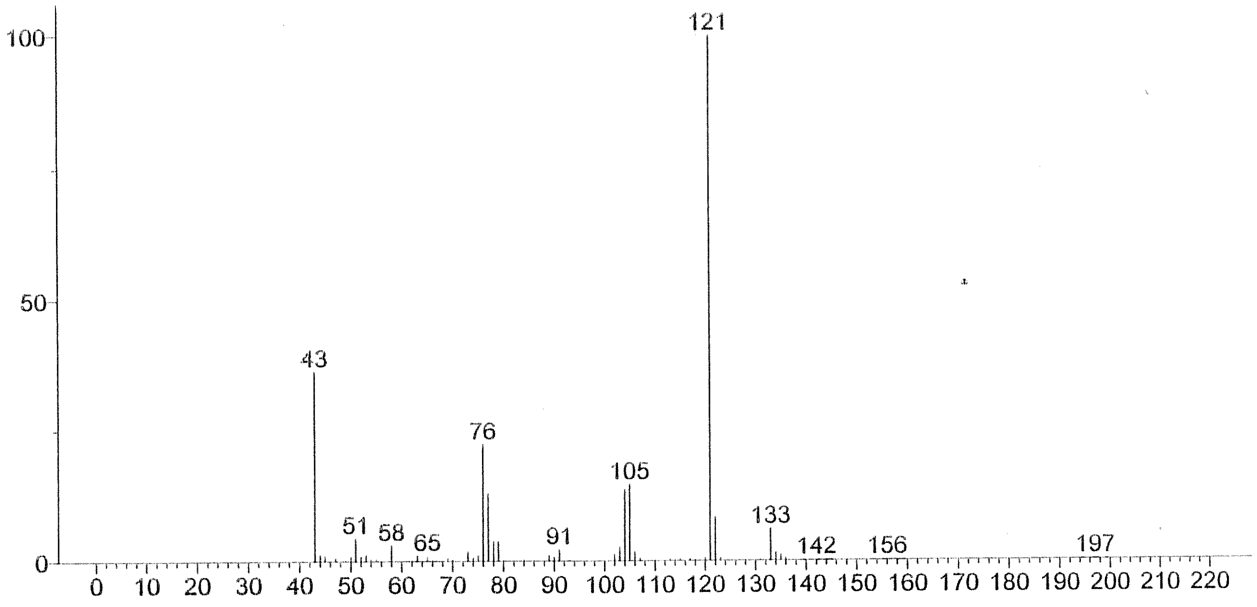

(Text File) +EI Scan (35.878 min) ALI-DRAG-H4-220421-.D Subtract

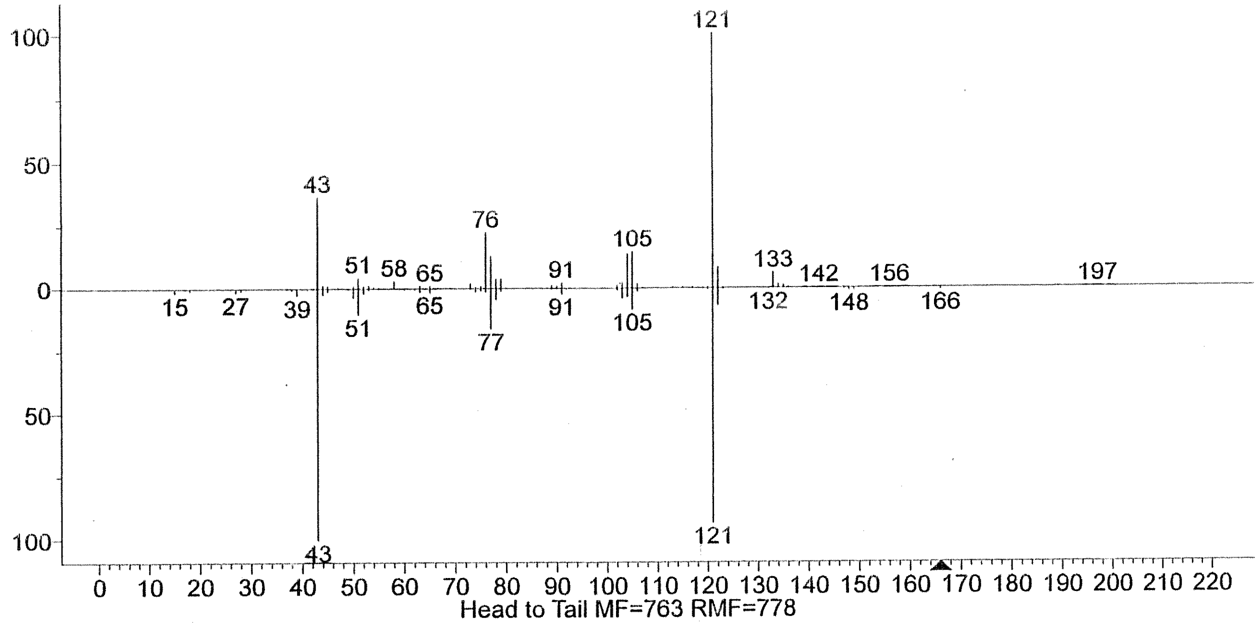

Head to Tail MF=763 RMF=778

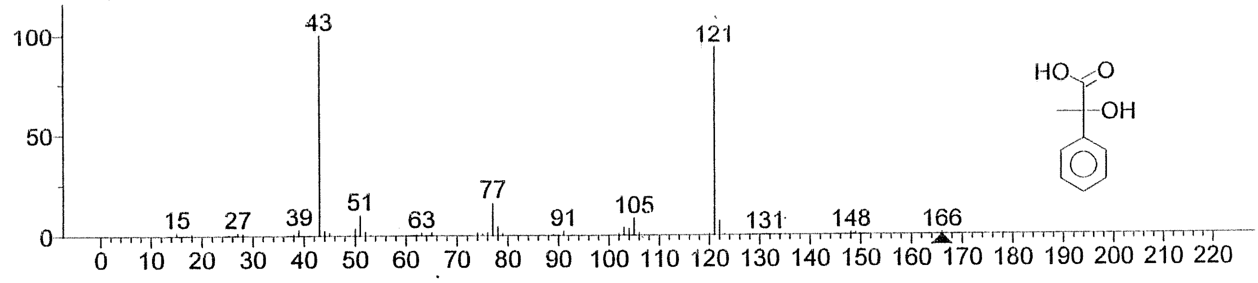

(mainlib) Benzeneacetic acid, α-hydroxy-α-methyl-

Name: Benzeneacetic acid, α-hydroxy-α-methyl-
Formula: $C_9H_{10}O_3$
MW: 166 CAS#: 515-30-0 NIST#: 234792 ID#: 10592 DB: mainlib
Other DBs: Fine, TSCA, HODOC, NIH, EINECS
Contributor: Japan AIST/NIMC Database- Spectrum MS-NW-7445
10 largest peaks:
43 999 | 121 932 | 77 160 | 51 102 | 105 83 | 122 70 | 78 45 | 103 40 | 50 36 | 104 34 |
Synonyms:
1.Mandelic acid, α-methyl-
2.α-Hydroxy-α-phenylpropionic acid
3.α-Phenyllactic acid
4.Atrolactic acid
5.2-Hydroxy-2-phenylpropionic acid
6.2-Phenyl-2-hydroxypropionic acid
7.2-Phenyllactic acid
8.dl-Atrolactic acid
9.2-Hydroxy-2-phenylpropanoic acid #

Estimated non-polar retention index (n-alkane scale):
Value: 1441 iu
Confidence interval (Diverse functional groups): 89(50%) 382(95%) iu

4c

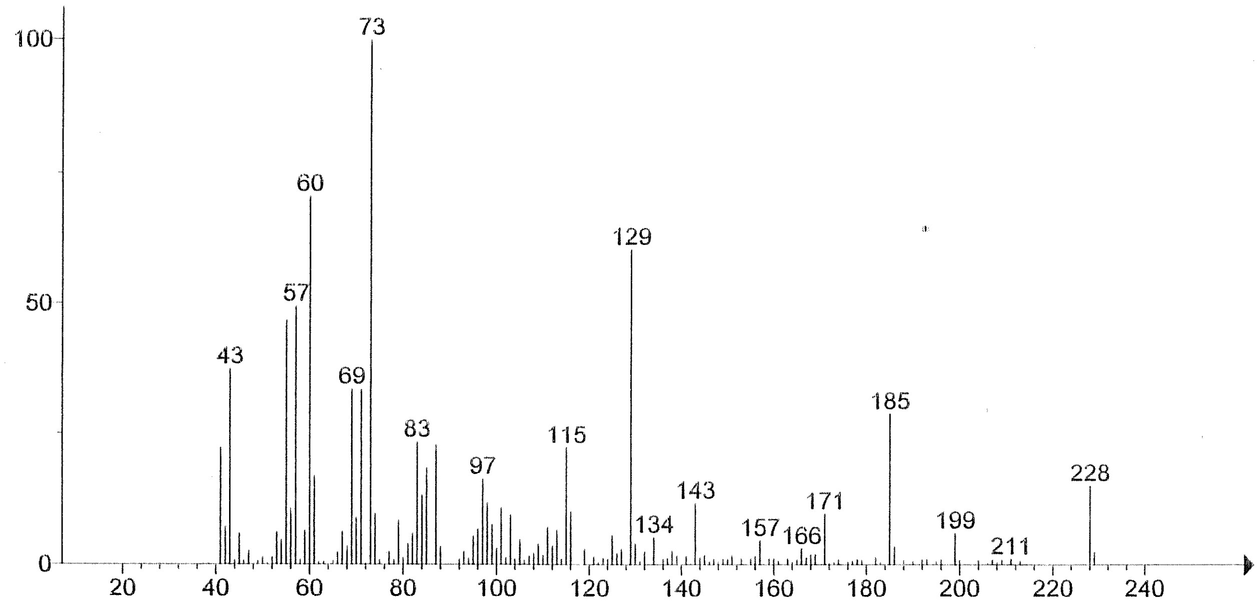

(Text File) +EI Scan (40.258 min) ALI-DRAG-H4-220421-.D Subtract

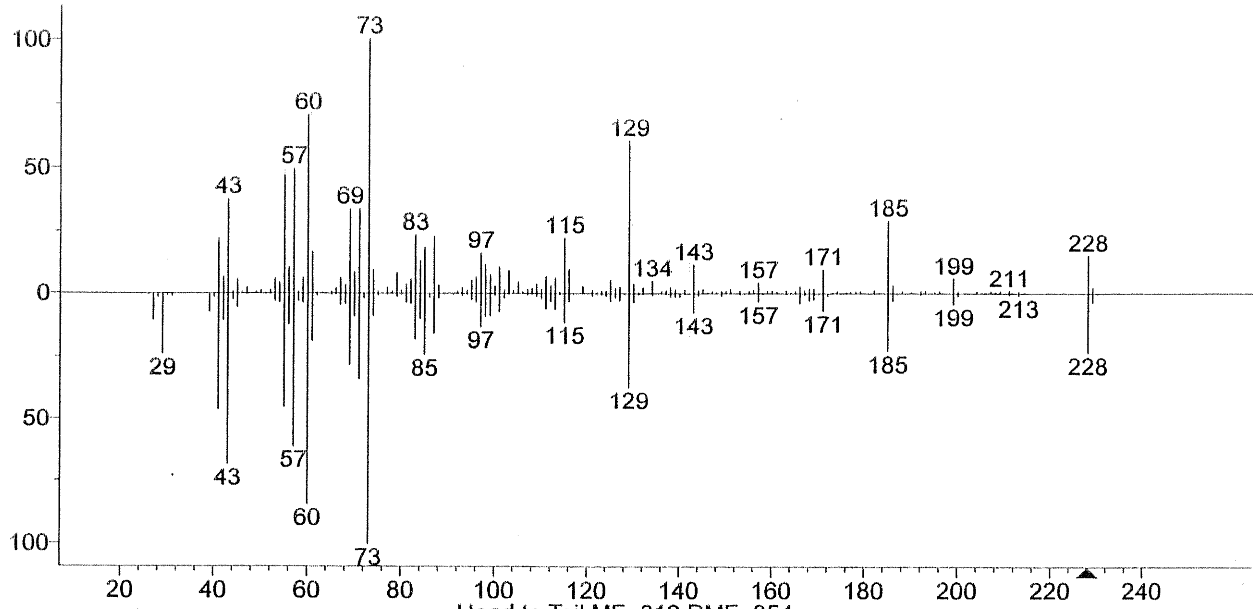

Head to Tail MF=812 RMF=854

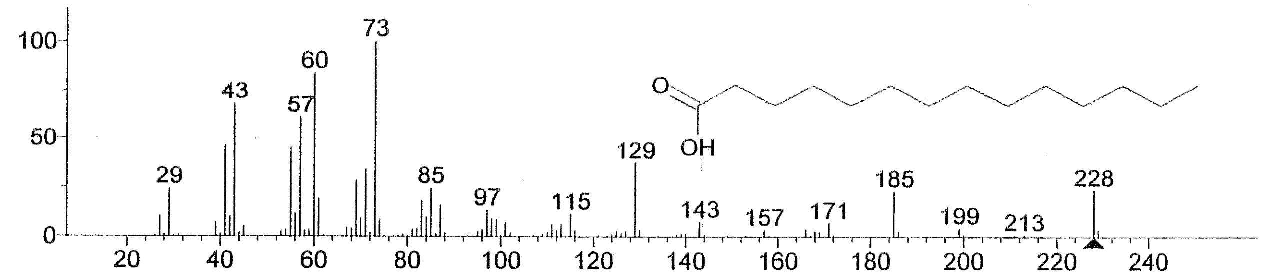

(replib) Tetradecanoic acid

Name: Tetradecanoic acid
Formula: $C_{14}H_{28}O_2$
MW: 228 CAS#: 544-63-8 NIST#: 189107 ID#: 8471 DB: replib
Other DBs: Fine, TSCA, RTECS, EPA, HODOC, NIH, EINECS, IRDB
Contributor: Chemical Concepts
10 largest peaks:
73 999 | 60 841 | 43 681 | 57 609 | 41 461 | 55 451 | 129 371 | 71 338 | 69 282 | 85 241 |
Synonyms:
1.Myristic acid
2.n-Tetradecanoic acid
3.n-Tetradecoic acid
4.Neo-Fat 14
5.Univol U 316S
6.1-Tridecanecarboxylic acid
7.Coconut oil fatty acids
8.Crodacid
9.Emery 655
10.Hydrofol acid 1495
11.Hystrene 9014
12.n-Tetradecan-1-oic acid
13.Emery 654
14.Hystrene 9514
15.Philacid 1400
16.Prifac 2940
17.Prifrac 2940
18.Univol U 3165
19.Univol U320

Estimated non-polar retention index (n-alkane scale):
Value: 1769 iu
Confidence interval (Carboxylic acids): 51(50%) 220(95%) iu

Retention index.
1. Value: 1748 iu
Column Type: Capillary
Column Class: Standard non-polar
Active Phase: RTX-1
Column
Length: 60 m
Carrier Gas: He
Column Diameter: 0.22 mm
Phase Thickness: 0.25 um
Data Type: Linear
RI
Program Type: Ramp
Start T: 60 C
End T: 230 C
Heat Rate: 2 K/min
End Time: 35 min
Source: Paolini, J.;
Muselli, A.; Bernardini, A.-F.; Bighelli, A.; Casanova, J.; Costa, J., Thymol derivatives from essential oil of Doronicum corsicum L., Flavour Fragr. J., 22, 2007, 479-487.

2. Value: 1772 iu

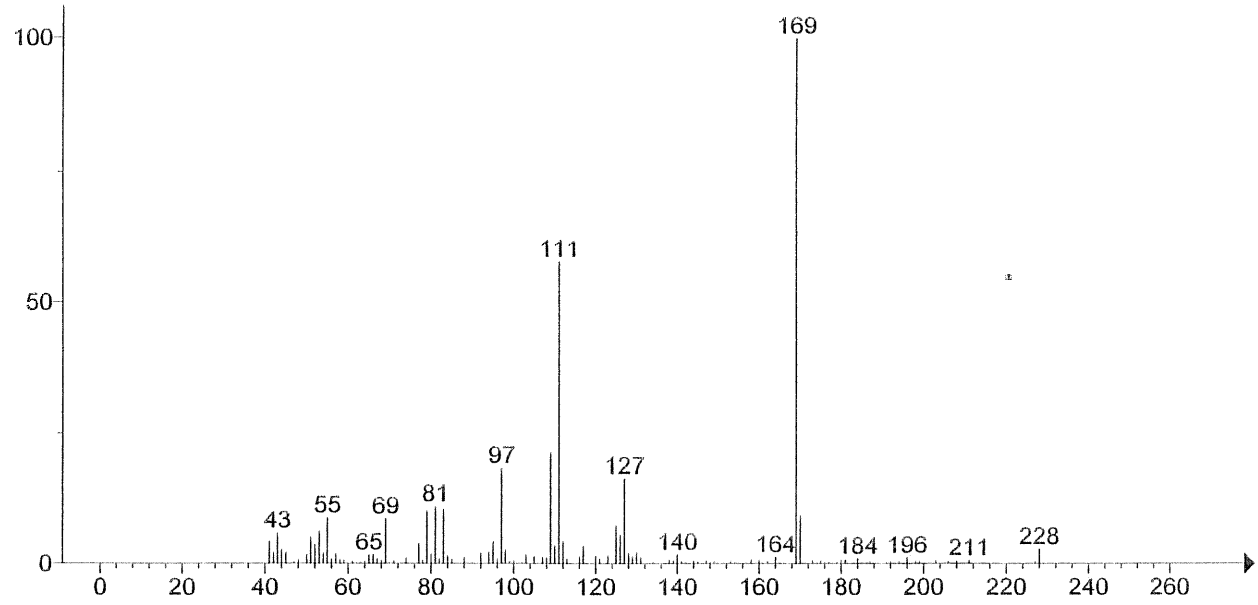

(Text File) +EI Scan (42.771 min) ALI-DRAG-H4-220421-.D Subtract

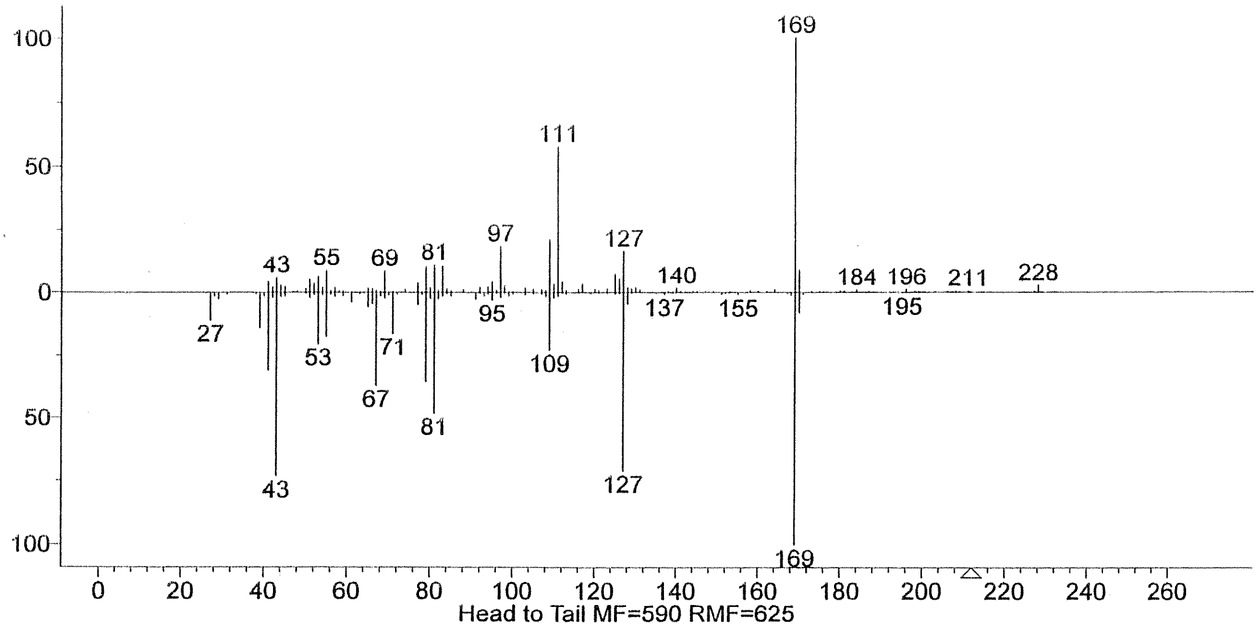

Head to Tail MF=590 RMF=625

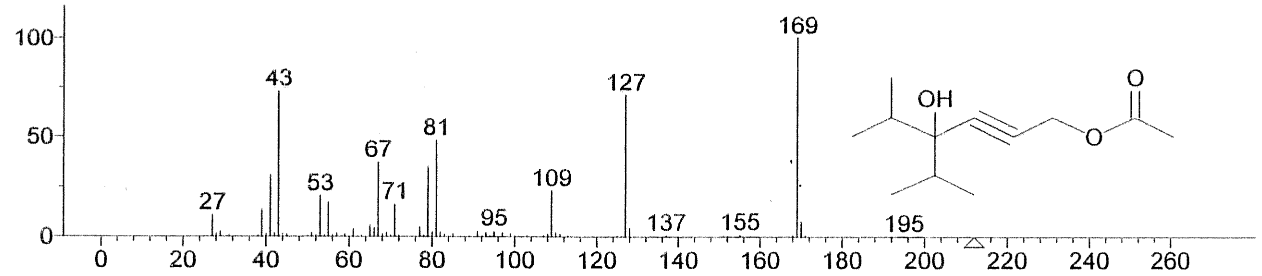

(mainlib) Acetate, [4-hydroxy-4-(1-methylethyl)-5-methyl-2-hexynyl] ester

Name: Acetate, [4-hydroxy-4-(1-methylethyl)-5-methyl-2-hexynyl] ester
Formula: $C_{12}H_{20}O_3$
MW: 212 CAS#: 70372-90-6 NIST#: 196753 ID#: 125443 DB: mainlib
Other DBs: None
Contributor: Chemical Concepts
10 largest peaks:
169 999 | 43 730 | 127 709 | 81 481 | 67 370 | 79 355 | 41 307 | 109 229 | 53 203 | 55 174 |
Synonyms:
1.4-Hydroxy-4-isopropyl-5-methyl-2-hexynyl acetate #

Estimated non-polar retention index (n-alkane scale):
Value: 1362 iu
Confidence interval (Diverse functional groups): 89(50%) 382(95%) iu

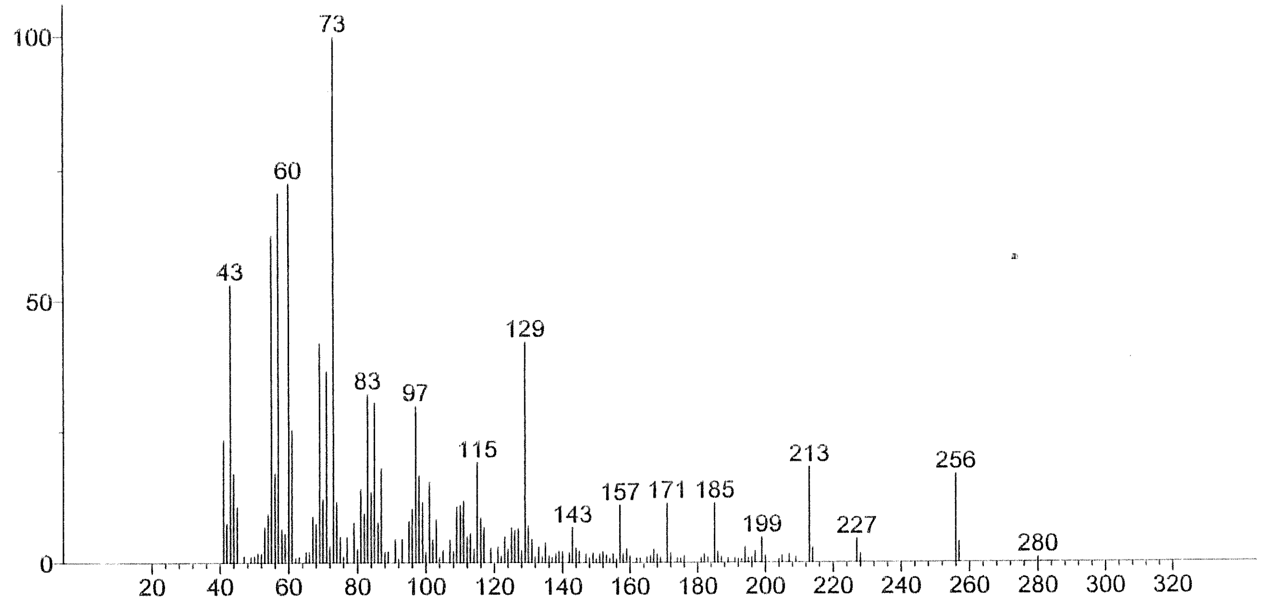

(Text File) +EI Scan (47.657 min) ALI-DRAG-H4-220421-.D

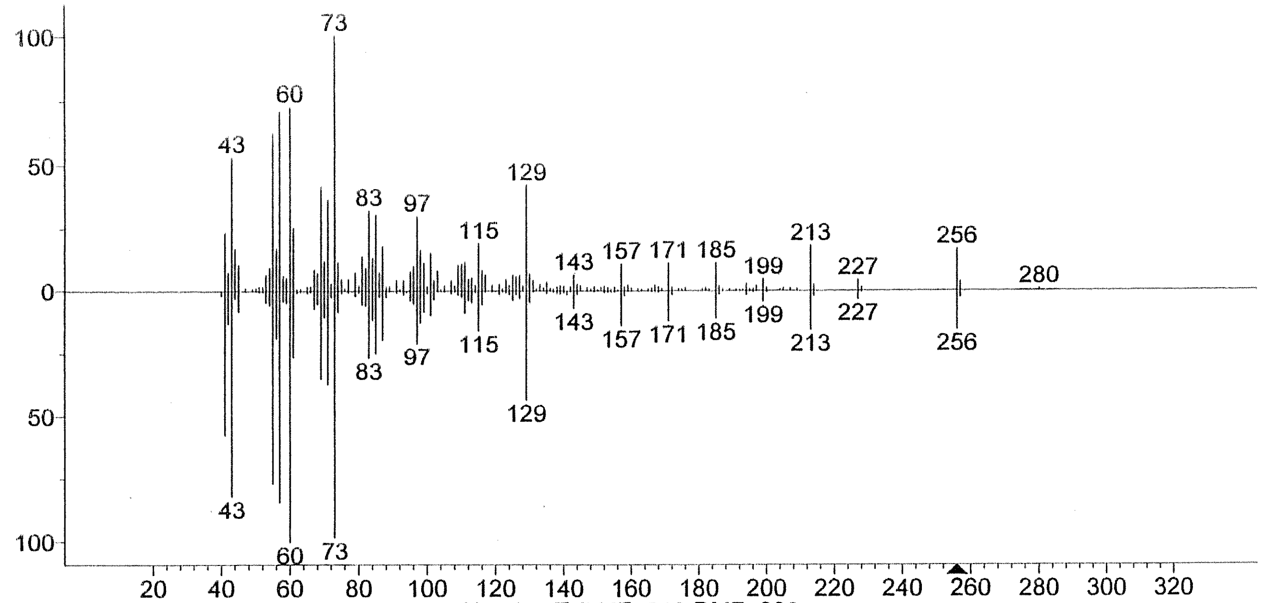

Head to Tail MF=813 RMF=836

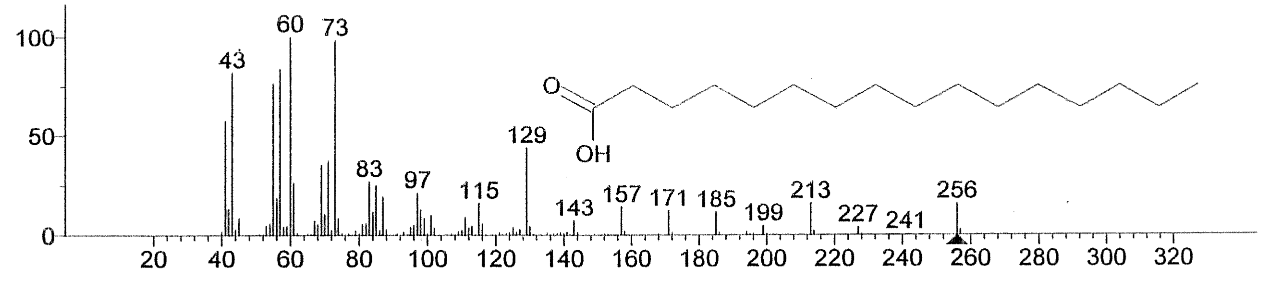

(replib) n-Hexadecanoic acid

51

Name: n-Hexadecanoic acid
Formula: $C_{16}H_{32}O_2$
MW: 256 CAS#: 57-10-3 NIST#: 335494 ID#: 6723 DB: replib
Other DBs: Fine, TSCA, RTECS, EPA, HODOC, NIH, EINECS, IRDB
Contributor: Drug Lab
10 largest peaks:
60 999 | 73 980 | 57 840 | 43 817 | 55 767 | 41 574 | 129 435 | 71 373 | 69 351 | 83 267 |
Synonyms:
1.Hexadecanoic acid
2.n-Hexadecoic acid
3.Palmitic acid
4.Pentadecanecarboxylic acid
5.1-Pentadecanecarboxylic acid
6.Cetylic acid
7.Emersol 140
8.Emersol 143
9.Hexadecylic acid
10.Hydrofol
11.Hystrene 8016
12.Hystrene 9016
13.Industrene 4516
14.Prifrac 2960
15.Glycon P-45
16.Prifac 2960
17.Univol U332

Estimated non-polar retention index (n-alkane scale):
Value: 1968 iu
Confidence interval (Carboxylic acids): 51(50%) 220(95%) iu

Retention index.
1. Value: 1942 iu
Column Type: Capillary
Column Class: Standard non-polar
Active Phase: RTX-1
Column
Length: 60 m
Carrier Gas: He
Column Diameter: 0.22 mm
Phase Thickness: 0.25 um
Data Type: Linear
RI
Program Type: Ramp
Start T: 60 C
End T: 230 C
Heat Rate: 2 K/min
End Time: 35 min
Source: Paolini, J.;
Muselli, A.; Bernardini, A.-F.; Bighelli, A.; Casanova, J.; Costa, J., Thymol derivatives from essential oil of Doronicum corsicum L., Flavour Fragr. J., 22, 2007, 479-487.

2. Value: 1972 iu
Column Type: Capillary
Column

52

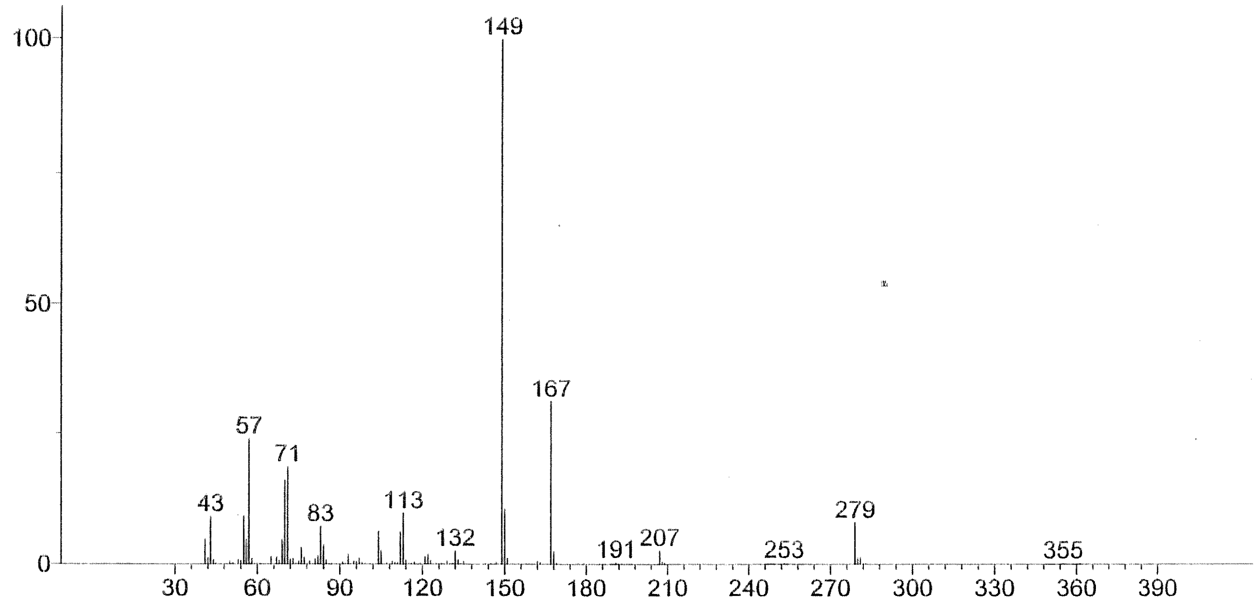

(Text File) +EI Scan (76.665 min) ALI-DRAG-H4-220421-.D

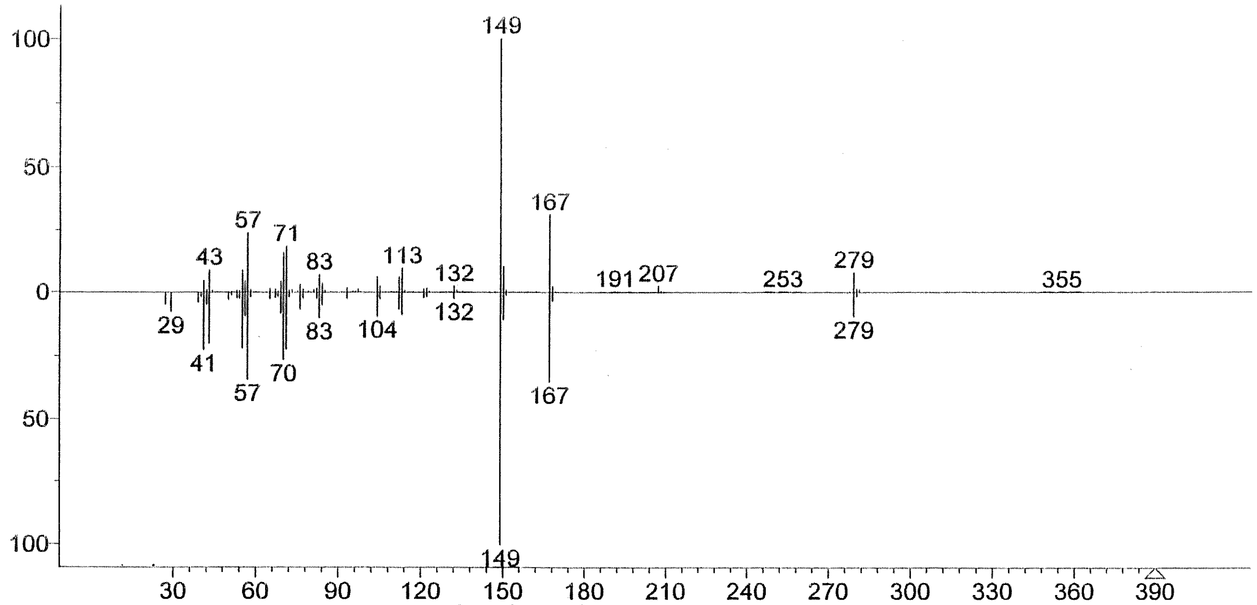

Head to Tail MF=895 RMF=950

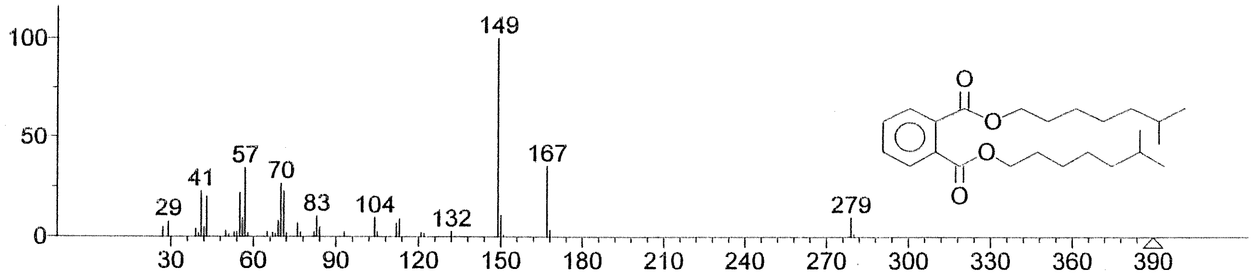

(replib) 1,2-Benzenedicarboxylic acid, diisooctyl ester

53

Name: 1,2-Benzenedicarboxylic acid, diisooctyl ester
Formula: $C_{24}H_{38}O_4$
MW: 390 CAS#: 27554-26-3 NIST#: 113206 ID#: 20061 DB: replib
Other DBs: Fine, TSCA, RTECS, EINECS, IRDB
Contributor: NIST Mass Spectrometry Data Center, 1990.
10 largest peaks:
149 999 | 167 350 | 57 341 | 70 264 | 41 225 | 71 224 | 55 218 | 43 200 | 150 107 | 83 100 |
Synonyms:
1.Diisooctyl phthalate
2.Hexaplas M/O
3.Isooctyl phthalate
4.Corflex 880
5.DIOP
6.Flexol plasticizer diop
7.Morflex 100
8.Palatinol D10
9.Phthalic acid, bis(6-methylheptyl) ester
10.Phthalic acid, diisooctyl ester
11.Witcizer 313
12.Bis(6-methylheptyl) phthalate #

Estimated non-polar retention index (n-alkane scale):
Value: 2704 iu
Confidence interval (Esters): 47(50%) 201(95%) iu
