# Supplementary material for: Probing Antibacterial and Anticancer Potential of Selenicereus undatus, Pistacia vera L. and Olea europaea L. against Uropathogens, MCF-7 and A2780 Cancer Cells
Source: Molecules. 2023 Dec 18;28(24):8148. doi: 10.3390/molecules28248148 (PMC10746009; doi:10.3390/molecules28248148)
Supplement: Supplementary file 1 [file molecules-28-08148-s001.zip › SI - 3. GCMS of O. europeae.pdf]

**Sample Name**	ALI-OLE-H7-220421-	**Position**	12	**Instrument Name**	GCMS TQQQ	**User Name**	HEJ-G-104-03\Agile
**Inj Vol**	3	**InjPosition**		**SampleType**		**IRM Calibration Status**	Not Applicable
**Data Filename**	ALI-OLE-H7-220421-.D	**ACQ Method**	ALI-IMRAN-140421.M	**Comment**		**Acquired Time**	4/22/2021 3:25:56

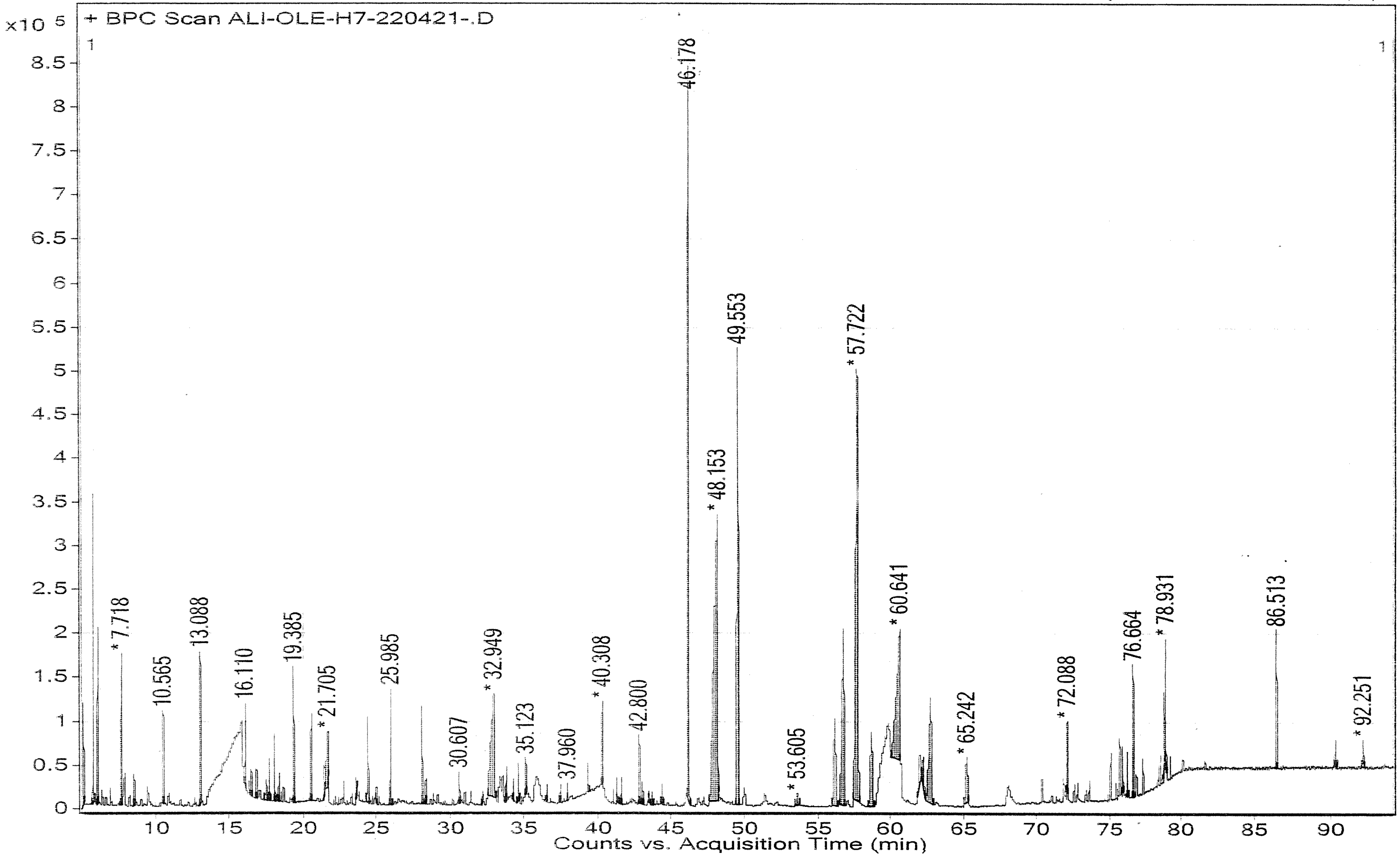

**Sample Name**	ALI-OLE-H7-220421-	**Position**	12	**Instrument Name**	GCMS TQQQ	**User Name**	HEJ-G-104-03\Agiler
**Inj Vol**	3	**InjPosition**		**SampleType**		**IRM Calibration Status**	Not Applicable
**Data Filename**	ALI-OLE-H7-220421-.D	**ACQ Method**	ALI-IMRAN-140421.M	**Comment**		**Acquired Time**	4/22/2021 3:25:56 I

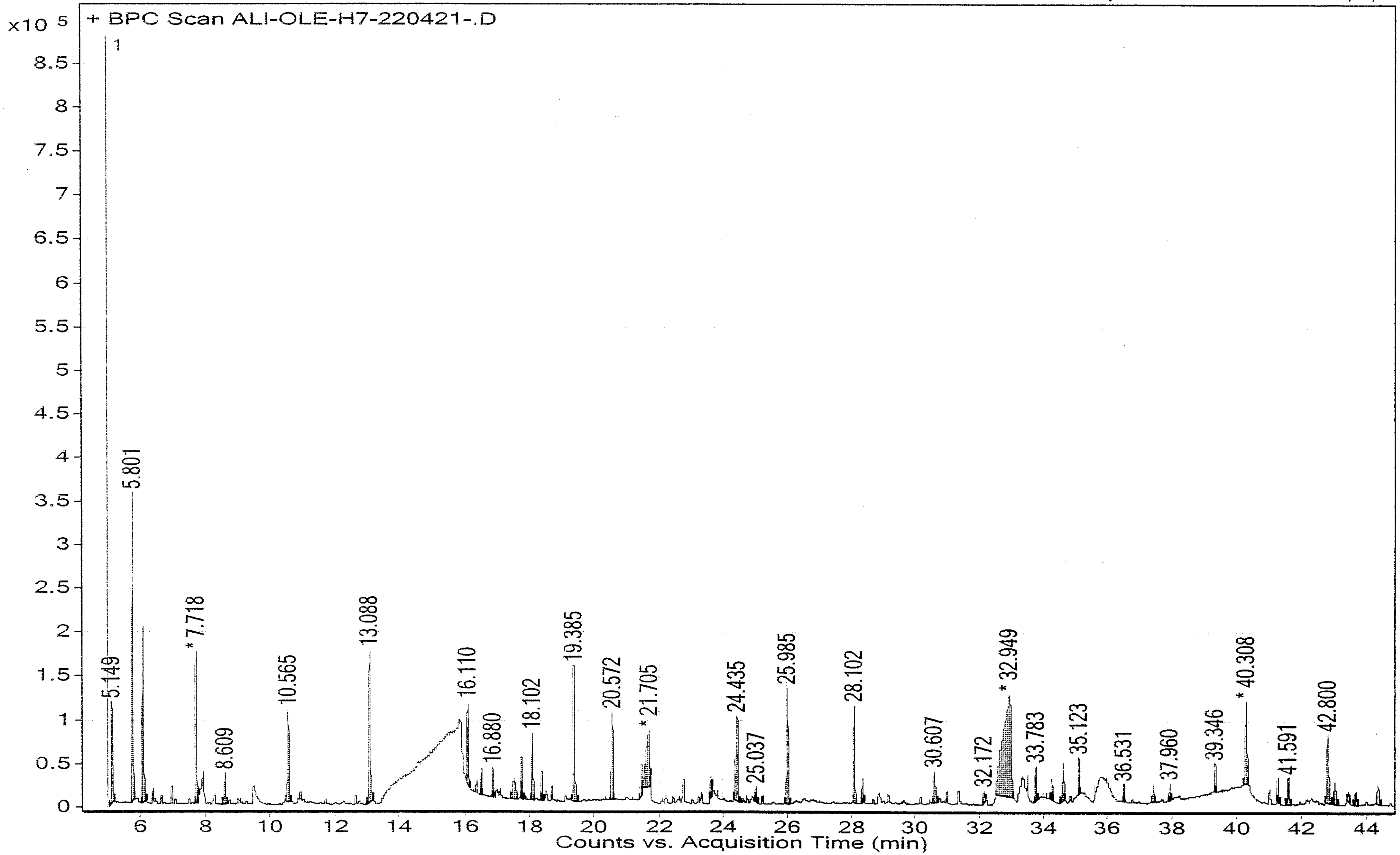

nt

PM

**Sample Name**	ALI-OLE-H7-220421-	**Position**	12	**Instrument Name**	GCMS TQQQ	**User Name**	HEJ-G-104-03\Agile[illegible]
**Inj Vol**	3	**InjPosition**		**SampleType**		**IRM Calibration Status**	Not Applicable
**Data Filename**	ALI-OLE-H7-220421-.D	**ACQ Method**	ALI-IMRAN-140421.M	**Comment**		**Acquired Time**	4/22/2021 3:25:56 [illegible]

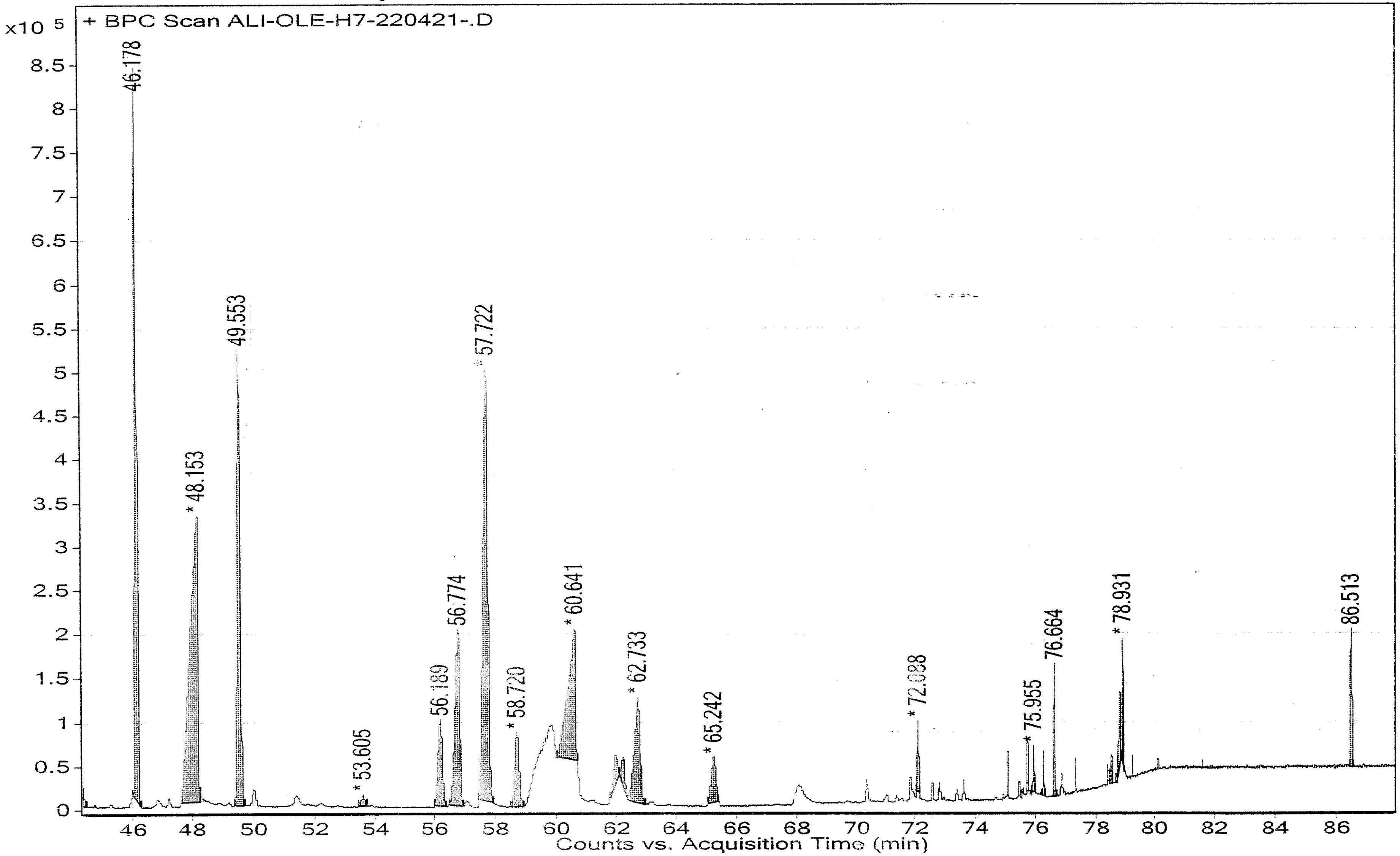

nt

PM

OLE-H7

Peak Number	RT	Area	Area %	Height	Width	Area Sum %	Height %
1	5.149	410717	6.84	116555	0.171	0.94	13.91
2	5.801	946984	15.77	352576	0.132	2.17	42.08
3	6.111	535451	8.92	201475	0.146	1.22	24.05
4	7.718	481259	8.01	172052	0.103	1.1	20.53
5	8.609	114071	1.9	35734	0.171	0.26	4.26
6	10.565	350011	5.83	105624	0.157	0.8	12.61
7	13.088	580500	9.67	173624	0.185	1.33	20.72
8	16.11	274501	4.57	94551	0.096	0.63	11.28
9	16.534	75490	1.26	31203	0.071	0.17	3.72
10	16.88	79231	1.32	33785	0.071	0.18	4.03
11	17.543	159584	2.66	23930	0.203	0.36	2.86
12	17.767	131423	2.19	47755	0.1	0.3	5.7
13	18.102	241267	4.02	76368	0.128	0.55	9.11
14	18.398	88855	1.48	32953	0.103	0.2	3.93
15	19.385	529896	8.82	154480	0.189	1.21	18.44
16	20.572	342596	5.71	99582	0.143	0.78	11.89
17	21.47	114981	1.91	35696	0.1	0.26	4.26
18	21.705	517610	8.62	64345	0.235	1.18	7.68
19	23.623	81349	1.35	25498	0.078	0.19	3.04
20	24.435	411901	6.86	97849	0.189	0.94	11.68
21	25.037	48147	0.8	17408	0.089	0.11	2.08
22	25.985	404538	6.74	132529	0.146	0.92	15.82
23	28.102	329360	5.49	110821	0.128	0.75	13.23
24	28.366	72137	1.2	28120	0.089	0.16	3.36
25	30.607	165311	2.75	36361	0.178	0.38	4.34
26	32.172	48007	0.8	14413	0.103	0.11	1.72
27	32.949	2080576	34.65	116905	0.552	4.76	13.95
28	33.783	122075	2.03	40033	0.1	0.28	4.78
29	34.275	62809	1.05	23908	0.082	0.14	2.85
30	34.638	149877	2.5	42947	0.16	0.34	5.13
31	35.123	100688	1.68	41594	0.078	0.23	4.96
32	36.531	48951	0.82	19967	0.075	0.11	2.38
33	37.45	60431	1.01	20607	0.096	0.14	2.46
34	37.96	48287	0.8	19800	0.075	0.11	2.36
35	39.346	69926	1.16	32429	0.082	0.16	3.87
36	40.308	368077	6.13	93655	0.153	0.84	11.18
37	41.292	78239	1.3	27569	0.086	0.18	3.29
38	41.591	118170	1.97	30911	0.135	0.27	3.69
39	42.8	321397	5.35	77885	0.178	0.73	9.3
40	43.024	99800	1.66	25638	0.164	0.23	3.06
41	43.445	41353	0.69	11947	0.107	0.09	1.43
42	43.676	63220	1.05	15146	0.171	0.14	1.81
43	44.382	102381	1.71	23168	0.171	0.23	2.77
44	46.178	4770040	79.44	837860	0.235	10.91	100
45	48.153	6004681	100	326469	0.62	13.73	38.96
46	49.553	3648713	60.76	522211	0.314	8.34	62.33
47	53.605	94429	1.57	13173	0.264	0.22	1.57
48	56.189	989765	16.48	99146	0.396	2.26	11.83
49	56.774	2158750	35.95	201324	0.442	4.94	24.03
50	57.722	5687920	94.72	492581	0.495	13.01	58.79

51	58.72	882814	14.7	84871	0.41	2.02	10.13
52	60.641	2653154	44.18	149019	0.659	6.07	17.79
53	61.999	314189	5.23	33652	0.296	0.72	4.02
54	62.241	269887	4.49	34077	0.246	0.62	4.07
55	62.733	1545203	25.73	120801	0.592	3.53	14.42
56	65.242	564707	9.4	51598	0.356	1.29	6.16
57	72.088	298458	4.97	81216	0.114	0.68	9.69
58	75.955	144881	2.41	52270	0.1	0.33	6.24
59	76.286	121572	2.02	50804	0.089	0.28	6.06
60	76.664	406628	6.77	151098	0.111	0.93	18.03
61	78.824	392137	6.53	84821	0.125	0.9	10.12
62	78.931	360383	6	142187	0.111	0.82	16.97
63	86.513	677962	11.29	157009	0.139	1.55	18.74
64	90.376	128620	2.14	29812	0.164	0.29	3.56
65	92.251	147538	2.46	31199	0.171	0.34	3.72
						99.96	

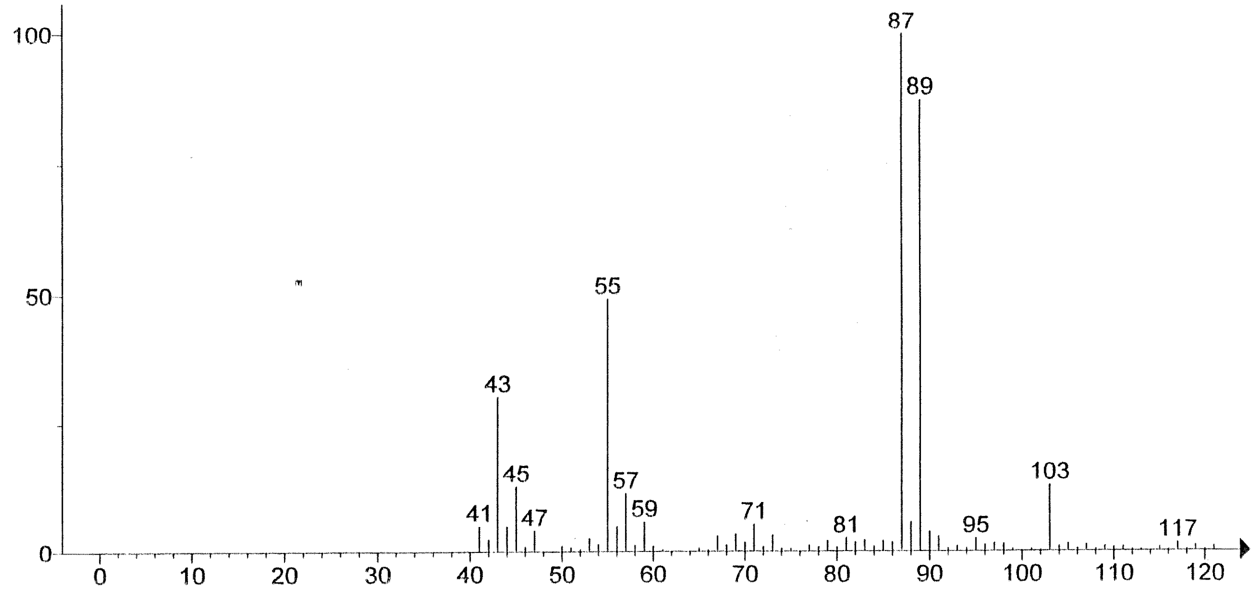

(Text File) +EI Scan (5.149 min) ALI-OLE-H7-220421-.D

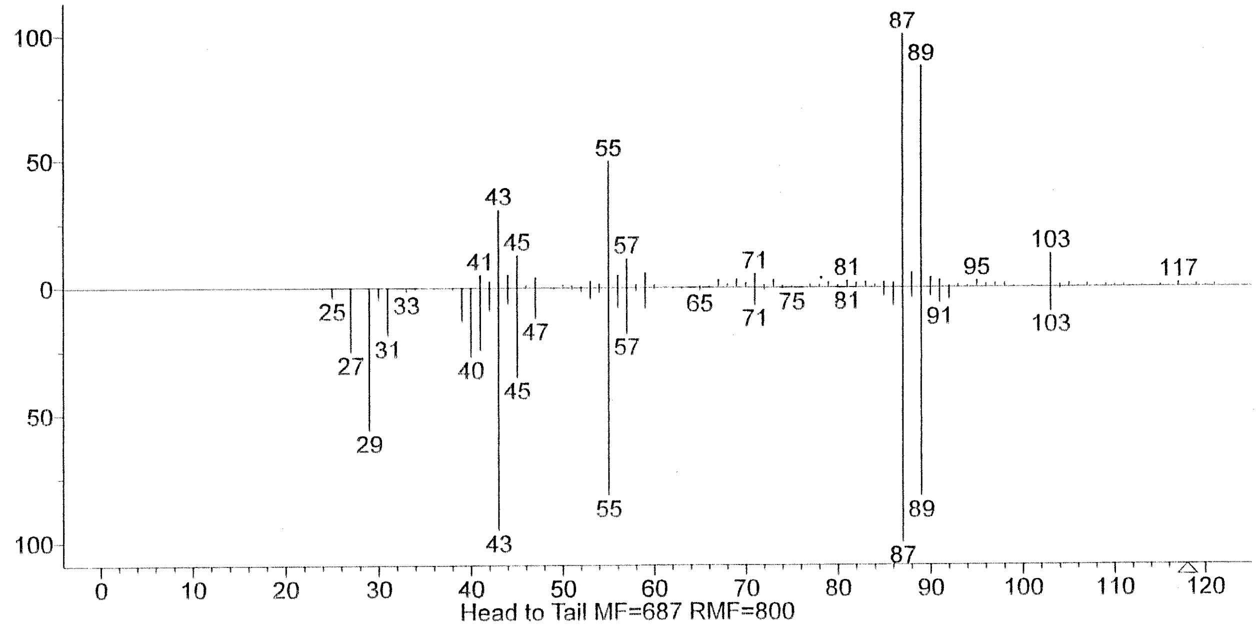

Head to Tail MF=687 RMF=800

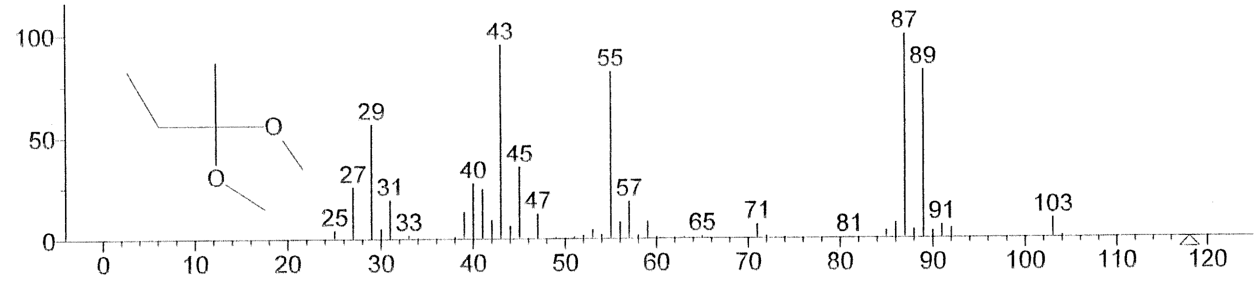

(mainlib) 2,2-Dimethoxybutane

Name: 2,2-Dimethoxybutane
Formula: $C_6H_{14}O_2$
MW: 118 CAS#: 3453-99-4 NIST#: 250250 ID#: 48775 DB: mainlib
Other DBs: None
Contributor: TNO Volatile Compounds in Food - Chemical Concepts
10 largest peaks:
87 999 | 43 950 | 89 822 | 55 817 | 29 560 | 45 351 | 40 270 | 27 251 | 41 242 | 31 188 |
Synonyms:
no synonyms.

Estimated non-polar retention index (n-alkane scale):
Value: 685 iu
Confidence interval (Ethers): 68(50%) 293(95%) iu

Retention index.
1. Value: 748.5 iu
Column Type: Capillary
Column Class: Semi-standard non-polar
Active Phase: SE-54
Column
Length: 30 m
Carrier Gas: He
Column Diameter: 0.25 mm
Phase Thickness: 0.25 um
Data Type: Normal
alkane RI
Program Type: Complex
Description: 40C(5min) =>4C/min => 230C(20min)=> 5C/min => 280C (5min)

Source: Gao, H.; Zhao, T.; Kong, Q.; Chen, X.; Hu, Z., Analysis of unknown organic pollutants in sewage by solid -phase extraction combined with gas chromatography-mass spectrometry, J. Chromatogr. Sci., 42, 2004, 91-99.

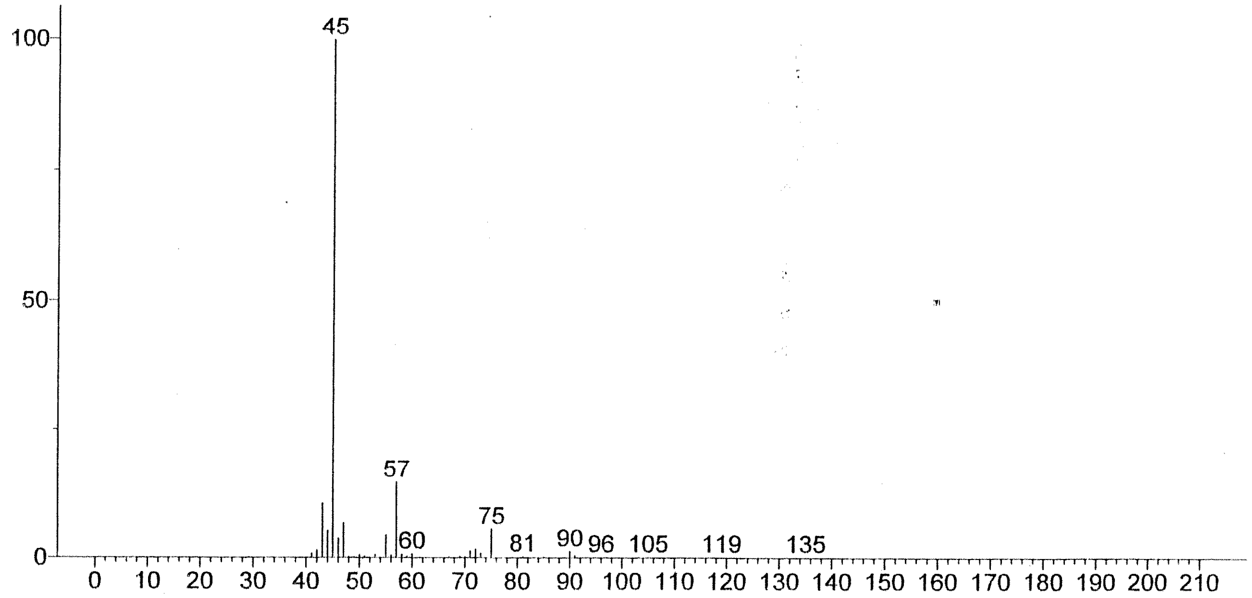

(Text File) +EI Scan (5.804 min) ALI-OLE-H7-220421-.D

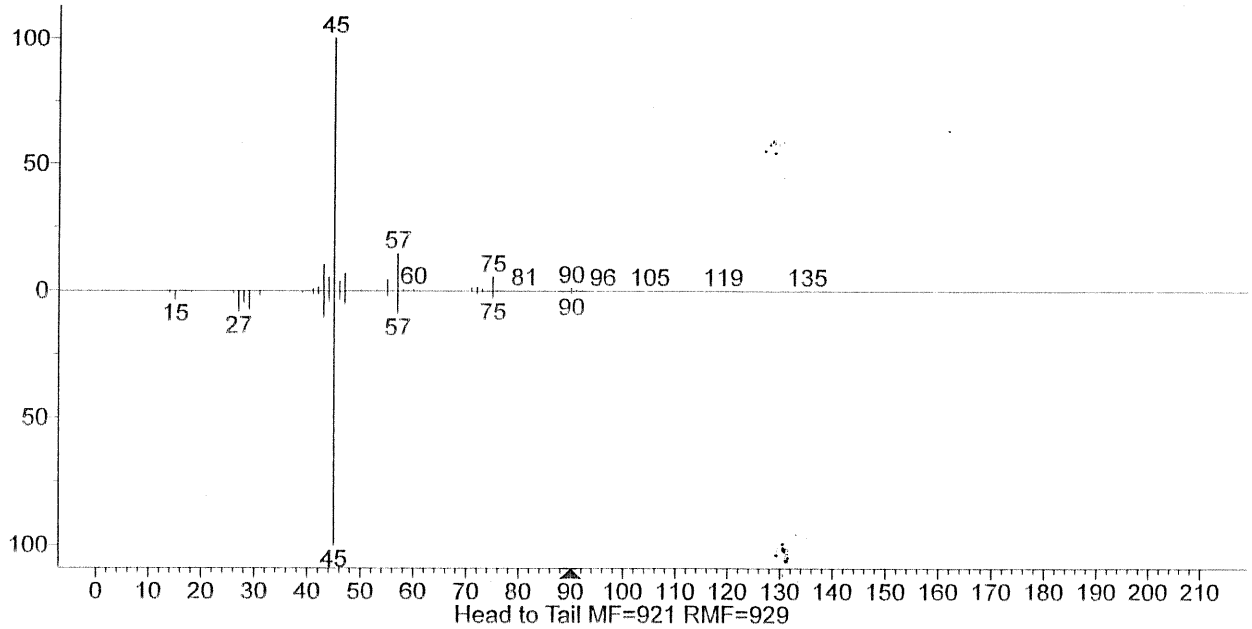

Head to Tail MF=921 RMF=929

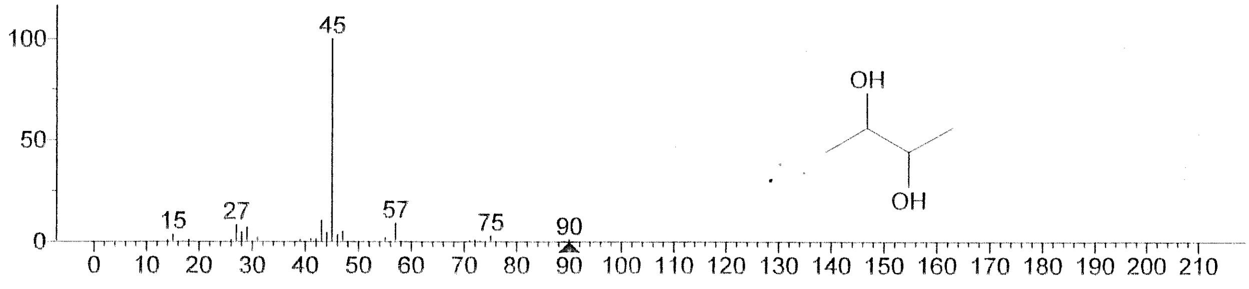

(mainlib) 2,3-Butanediol, [R-(R*,R*)]-

Name: 2,3-Butanediol, [R-(R*,R*)]-
Formula: $C_4H_{10}O_2$
MW: 90 CAS#: 24347-58-8 NIST#: 235815 ID#: 15312 DB: mainlib
Other DBs: HODOC, EINECS
Contributor: Japan AIST/NIMC Database- Spectrum MS-NW-6416
10 largest peaks:
45 999 | 43 104 | 57 89 | 27 82 | 29 71 | 47 51 | 28 46 | 44 45 | 15 35 | 46 34 |
Synonyms:
1.2,3-Butanediol #

Estimated non-polar retention index (n-alkane scale):
Value: 743 iu
Confidence interval (Alcohols): 41(50%) 176(95%) iu

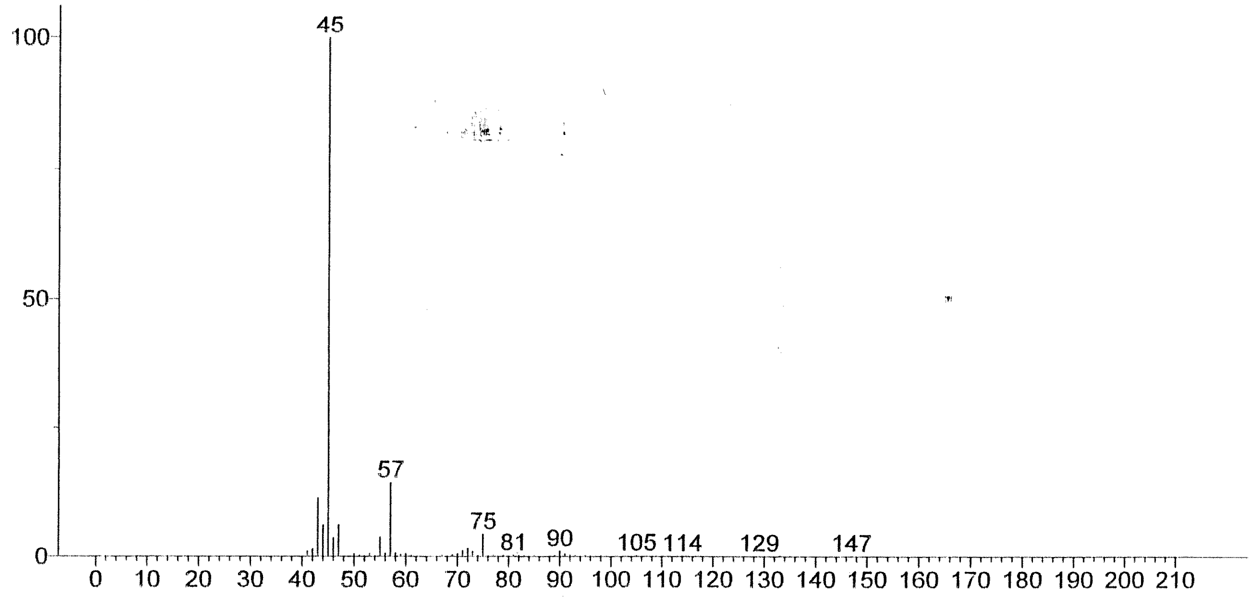

(Text File) +EI Scan (6.111 min) ALI-OLE-H7-220421-.D

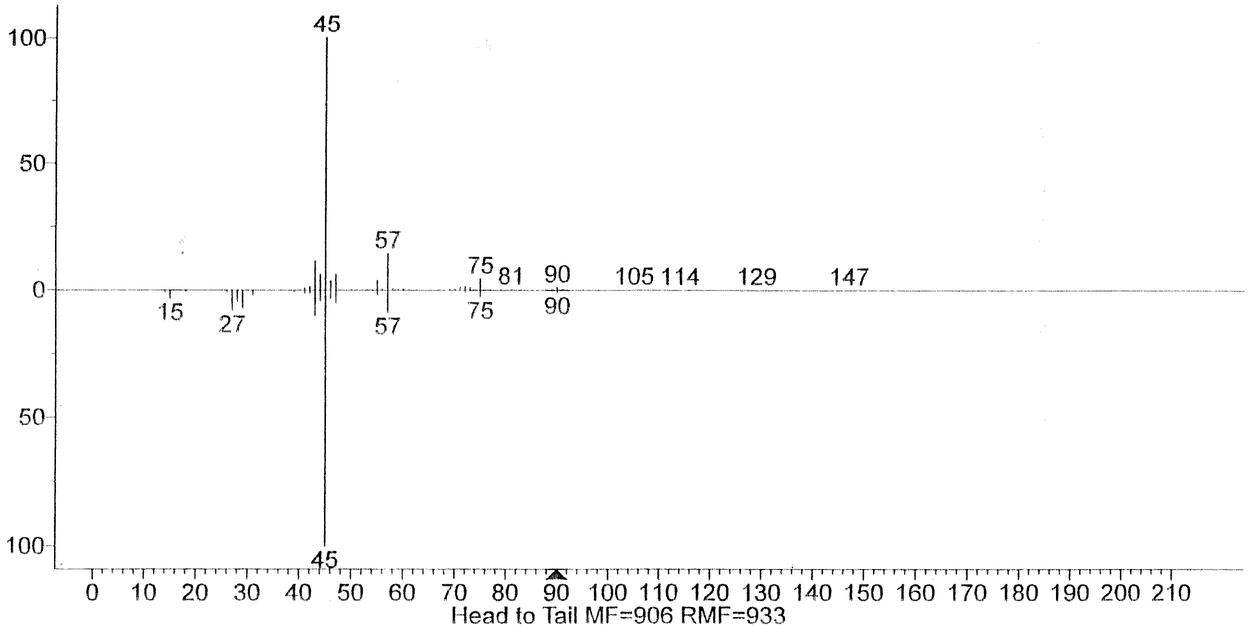

Head to Tail MF=906 RMF=933

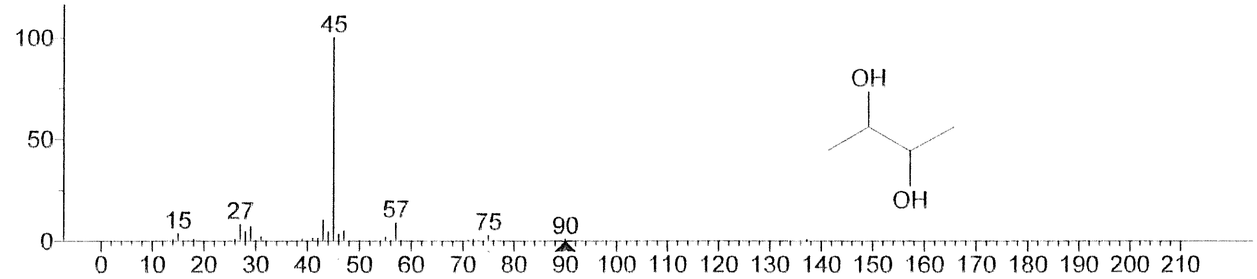

(mainlib) 2,3-Butanediol, [R-(R*,R*)]-

Name: 2,3-Butanediol, [R-(R*,R*)]-
Formula: $C_4H_{10}O_2$
MW: 90 CAS#: 24347-58-8 NIST#: 235815 ID#: 15312 DB: mainlib
Other DBs: HODOC, EINECS
Contributor: Japan AIST/NIMC Database- Spectrum MS-NW-6416
10 largest peaks:
45 999 | 43 104 | 57 89 | 27 82 | 29 71 | 47 51 | 28 46 | 44 45 | 15 35 | 46 34 |
Synonyms:
1.2,3-Butanediol #

Estimated non-polar retention index (n-alkane scale):
Value: 743 iu
Confidence interval (Alcohols): 41(50%) 176(95%) iu

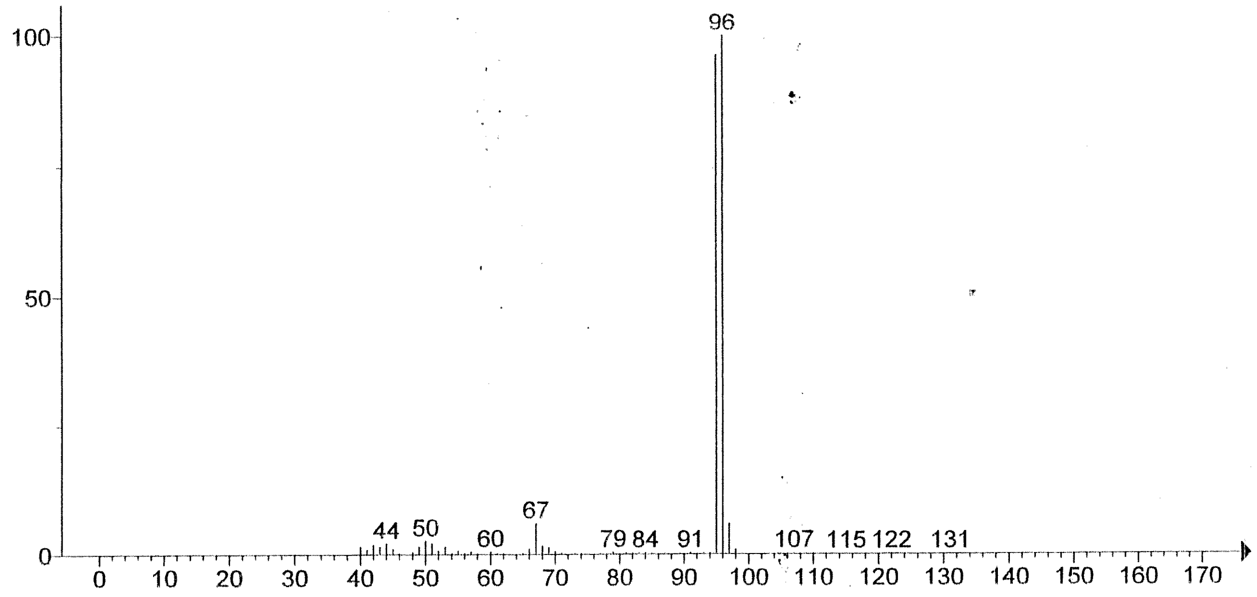

(Text File) +EI Scan (7.718 min) ALI-OLE-H7-220421-.D

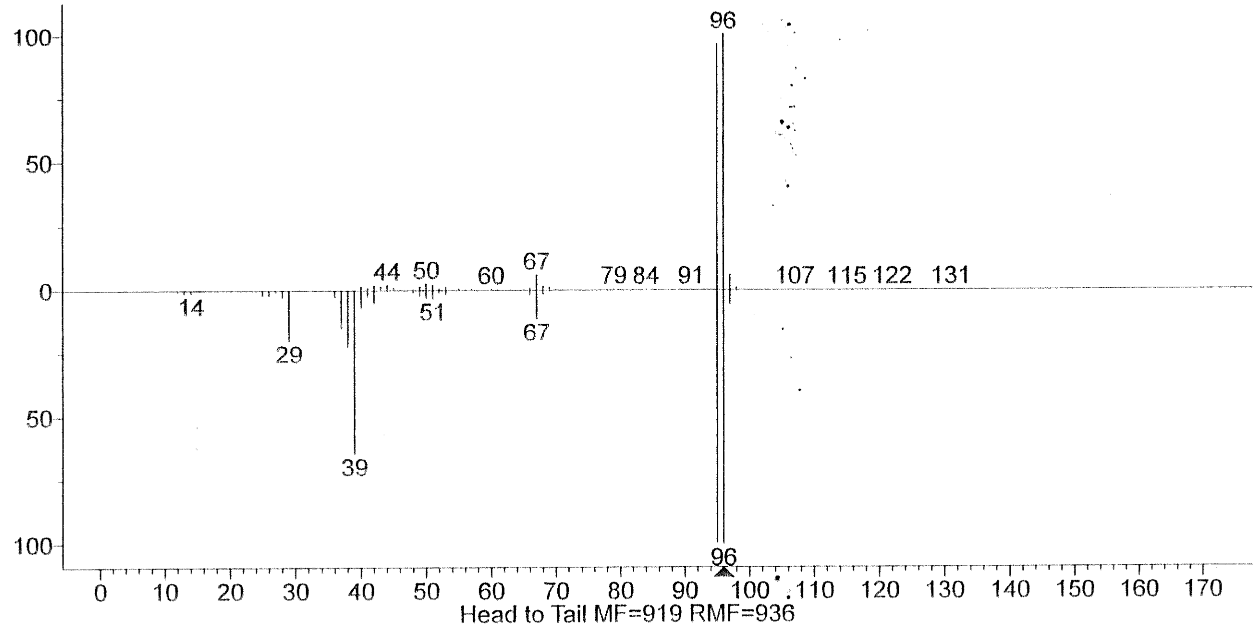

Head to Tail MF=919 RMF=936

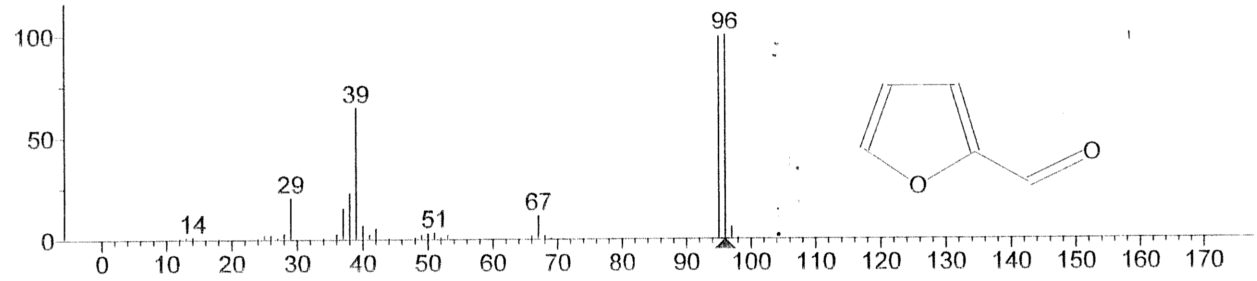

(mainlib) Furfural

Name: Furfural
Formula: $C_5H_4O_2$
MW: 96 CAS#: 98-01-1 NIST#: 118785 ID#: 59378 DB: mainlib
Other DBs: Fine, TSCA, RTECS, EPA, HODOC, NIH, EINECS, IRDB
Contributor: NIST Mass Spectrometry Data Center, 1990.
10 largest peaks:
96 999 | 95 991 | 39 644 | 38 226 | 29 201 | 37 151 | 67 114 | 40 69 | 97 57 | 42 53 |
Synonyms:
1.2-Furancarboxaldehyde
2.2-Furaldehyde
3.α-Furole
4.Artificial ant oil
5.Fural
6.Furaldehyde
7.Furale
8.Furancarbonal
9.Furfuraldehyde
10.Furfurole
11.Furfurylaldehyde
12.Furole
13.Pyromucic aldehyde
14.2-Formylfuran
15.2-Furanaldehyde
16.2-Furancarbonal
17.2-Furfural
18.2-Furfuraldehyde
19.2-Furylaldehyde
20.Furol
21.2-Furylmethanal
22.Artificial oil of ants
23.Furfurale
24.Furfurol
25.Nci-C56177
26.2-Furil-metanale
27.2-Furankarbaldehyd
28.Furfuralu
29.Rcra waste number U125
30.UN 1199
31.2-Furylaldehyde xypropane
32.2-Furylcarboxaldehyde
33.Cyclic aldehyde
34.Qo furfural

Estimated non-polar retention index (n-alkane scale):
Value: 831 iu
Confidence interval (Diverse functional groups): 89(50%) 382(95%) iu

Retention index.
1. Value: 794 iu
Column Type: Capillary
Column Class: Standard non-polar
Active Phase: CP Sil 5 CB
Column
Length: 60 m

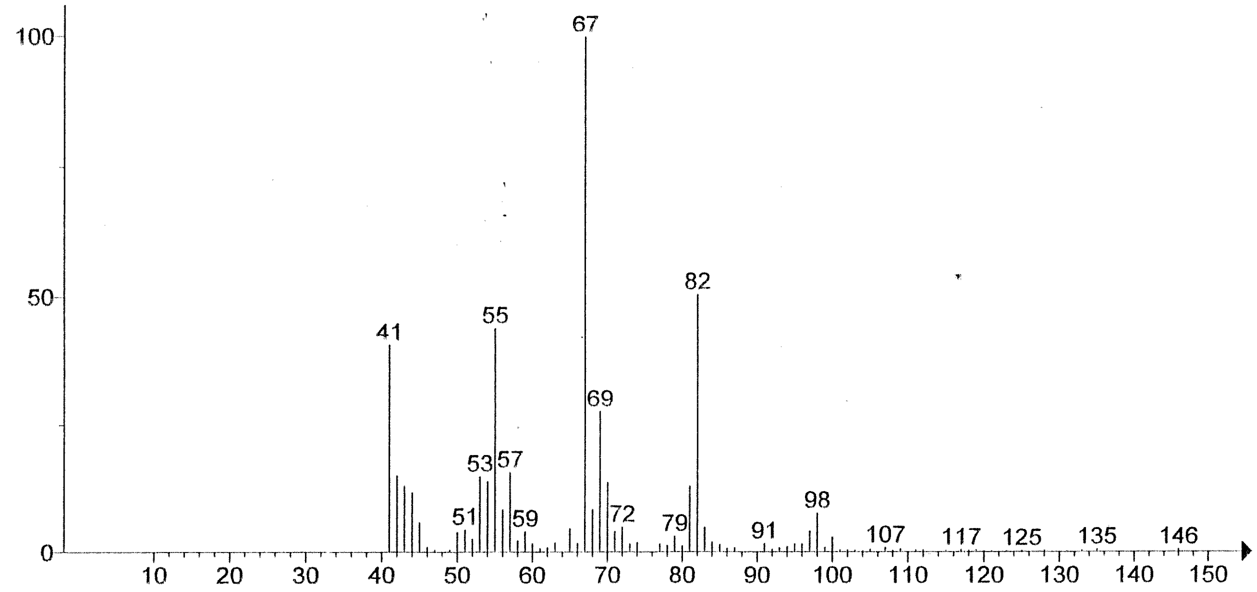

(Text File) +EI Scan (8.609 min) ALI-OLE-H7-220421-.D

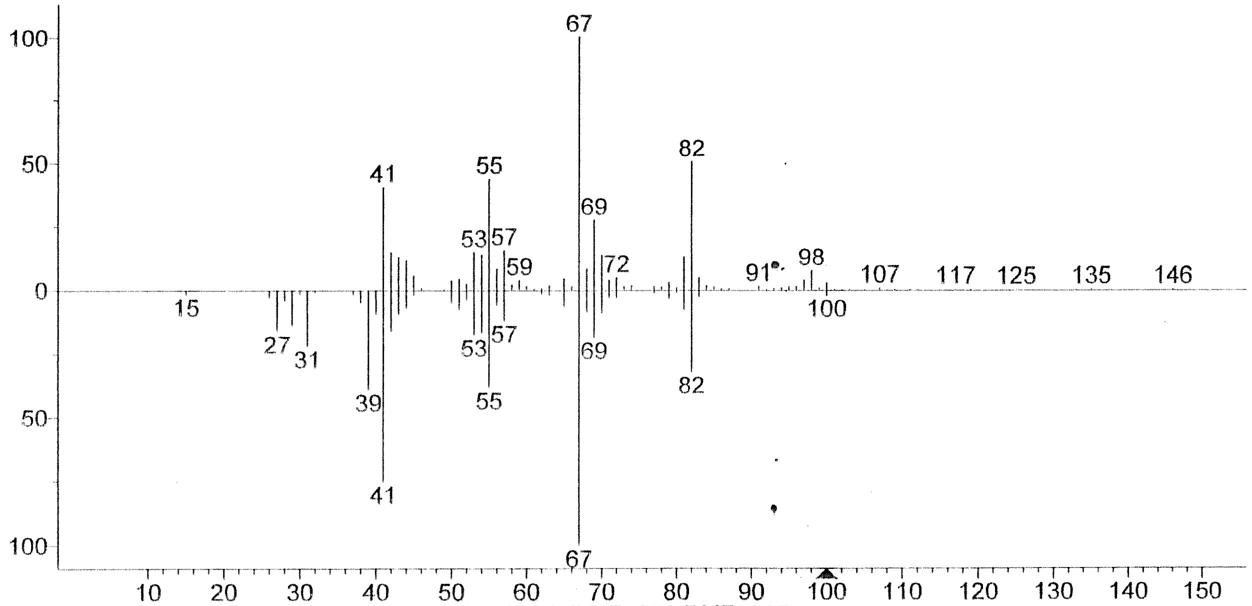

Head to Tail MF=856 RMF=937

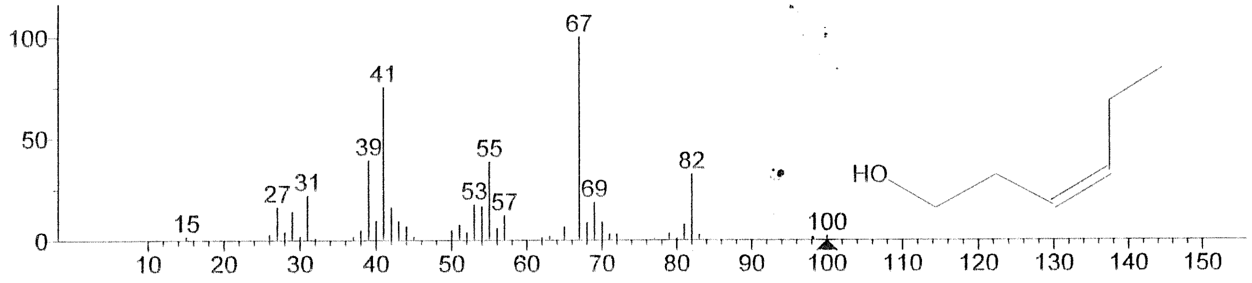

(mainlib) 3-Hexen-1-ol, (Z)-

Name: 3-Hexen-1-ol, (Z)-
Formula: $C_6H_{12}O$
MW: 100 CAS#: 928-96-1 NIST#: 114154 ID#: 28426 DB: mainlib
Other DBs: TSCA, RTECS, HODOC, NIH, EINECS, IRDB
Contributor: NIST Mass Spectrometry Data Center, 1990.
10 largest peaks:

67 999 |	41 750 |	39 390 |	55 382 |	82 321 |	31 218 |	69 184 |	53 174 |	54 164 |	27 161 |

Synonyms:
1.(Z)-Hex-3-en-1-ol
2.cis-3-Hexen-1-ol
3.cis-3-Hexene-1-ol
4.cis-3-Hexenol
5.Blatteralkohol (German)
6.Leaf alcohol
7.Z-3-Hexenol
8.3-(Z)-Hexenol
9.β,γ-Hexenol
10.Blatteralkohol
11.Hexen-30L-1
12.3-Hexen-1-ol
13.3-Hexen-1-ol, cis-
14.3-Hexenol, cis-
15.(3Z)-3-Hexen-1-ol #

Estimated non-polar retention index (n-alkane scale):
Value: 868 iu
Confidence interval (Alcohols): 41(50%) 176(95%) iu

Retention index.
1. Value: 838 iu
Column Type: Capillary
Column Class: Standard non-polar
Active Phase: HP-101
Column
Length: 25 m
Carrier Gas: He
Column Diameter: 0.2 mm
Phase Thickness: 0.2 um
Data Type: Linear
RI
Program Type: Ramp
Start T: 70 C
End T: 200 C
Heat Rate: 3 K/min
Start Time: 2 min
End Time: 15
min
Source: Politeo, O.; Jukic, M.; Milos, M., Chemical composition and antioxidant capacity of free volatile aglycones from basil (Ocimum basilicum L.) compared with its essential oil, Food Chem., 101, 2007, 379-385.

2.
Value: 872 iu
Column Type: Capillary
Column Class: Standard non-polar

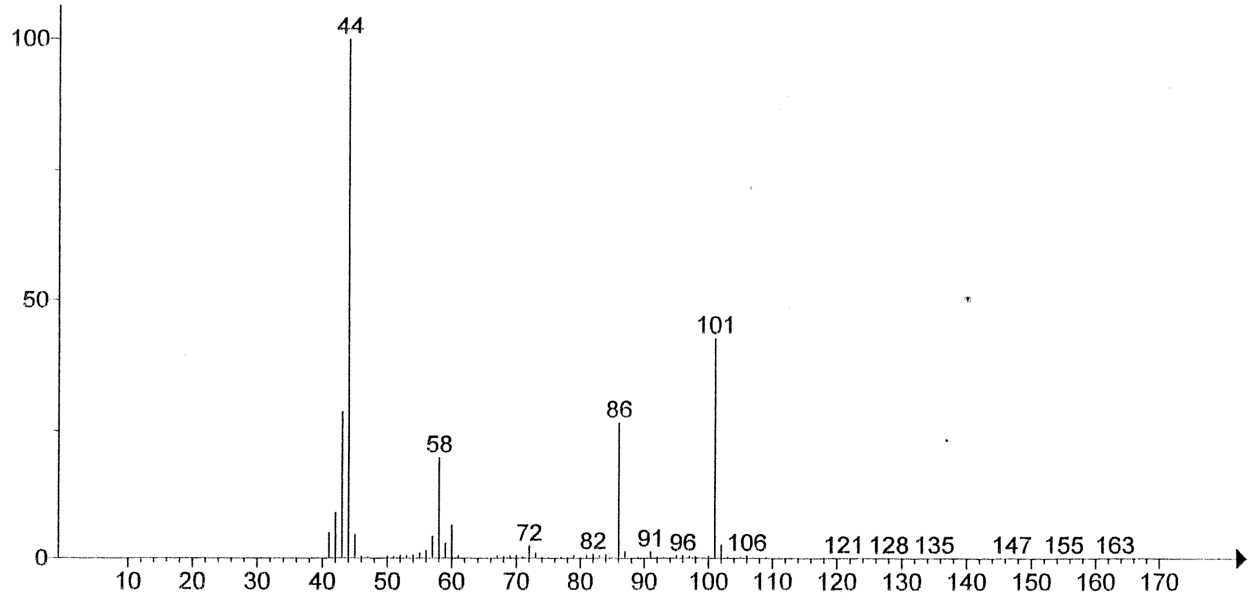

(Text File) +EI Scan (10.562 min) ALI-OLE-H7-220421-.D

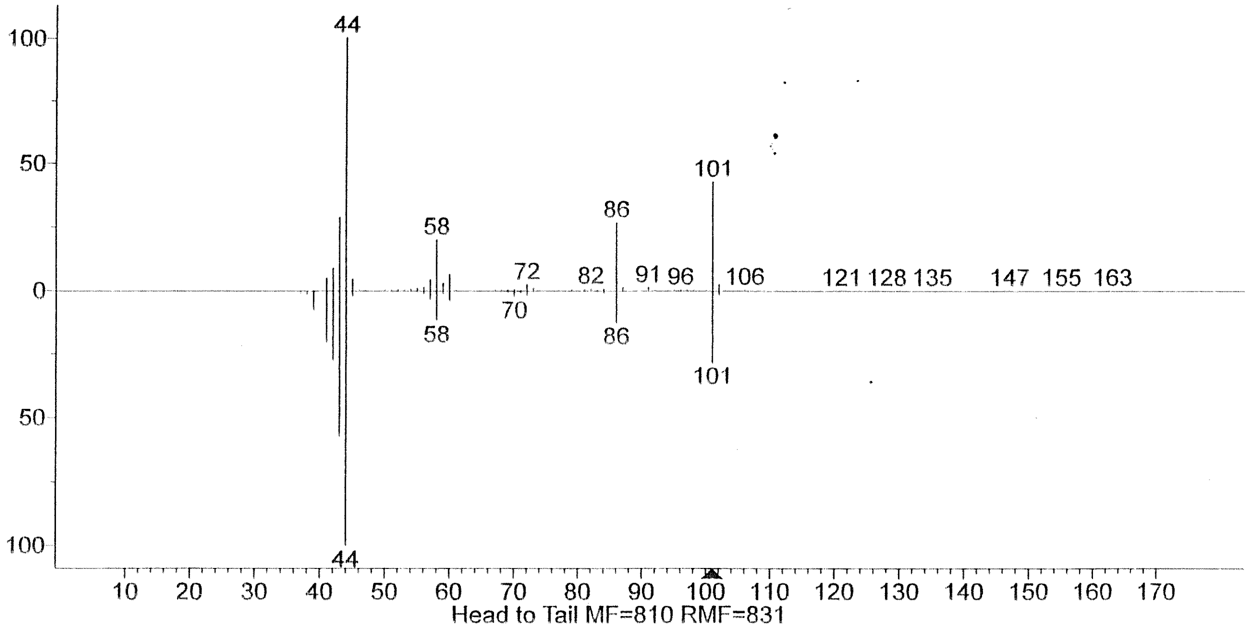

Head to Tail MF=810 RMF=831

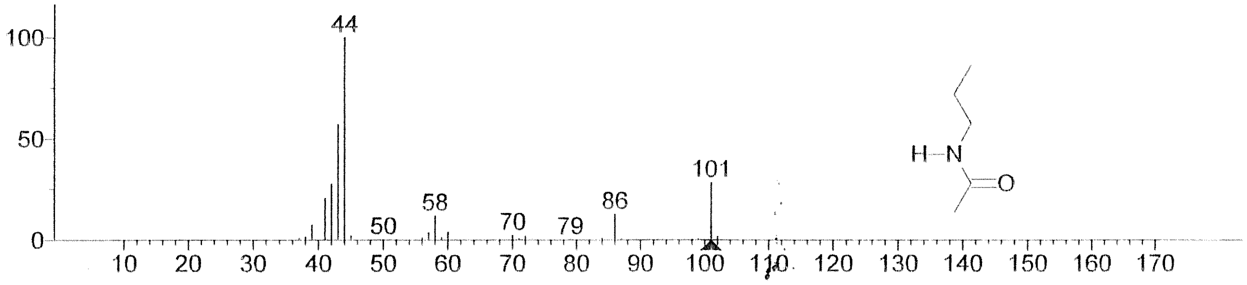

(mainlib) N-(n-Propyl)acetamide

Name: N-(n-Propyl)acetamide
Formula: $C_5H_{11}NO$
MW: 101 CAS#: 5331-48-6 NIST#: 223038 ID#: 13993 DB: mainlib
Other DBs: Fine, EINECS
Contributor: Chemical Concepts
10 largest peaks:
44 999 | 43 571 | 101 283 | 42 274 | 41 203 | 86 126 | 58 118 | 39 74 | 60 39 | 57 35 |
Synonyms:
1.Acetamide, N-propyl-
2.N-Propylacetamide
3.Acetamide, N-n-propyl-,

Estimated non-polar retention index (n-alkane scale):
Value: 918 iu
Confidence interval (Nitrogen-containing): 83(50%) 356(95%) iu

Retention index.
1. Value: 974 iu
Column Type: Packed
Column Class: Standard non-polar
Active Phase: SE-30
Column Length:
1 m
Carrier Gas: N2
Substrate: Chromosorb W AW
Data Type: Kovats RI
Program Type: Isothermal
Start T:
180 C
Source: Krawczyk, W.; Piotrowski, G.T., Relationships Between Structure and Retention Index for N -Substituted Amides of Aliphatic Acids on a Non-Polar Column, J. Chromatogr., 463, 1989, 297-304.

2. Value: 974
iu
Column Type: Capillary
Column Class: Standard non-polar
Active Phase: Polydimethyl siloxanes
Data Type:
Normal alkane RI
Program Type: Ramp
Source: Zenkevich, I.G.; Chupalov, A.A., New Possibilities of Chromato
Mass Pectrometric Identification of Organic Compounds Using Increments of Gas Chromatographic Retention Indices of Molecular Structural Fragments, Zh. Org. Khim. (Rus.), 32(5), 1996, 656-666, In original 656-666.

<...
>

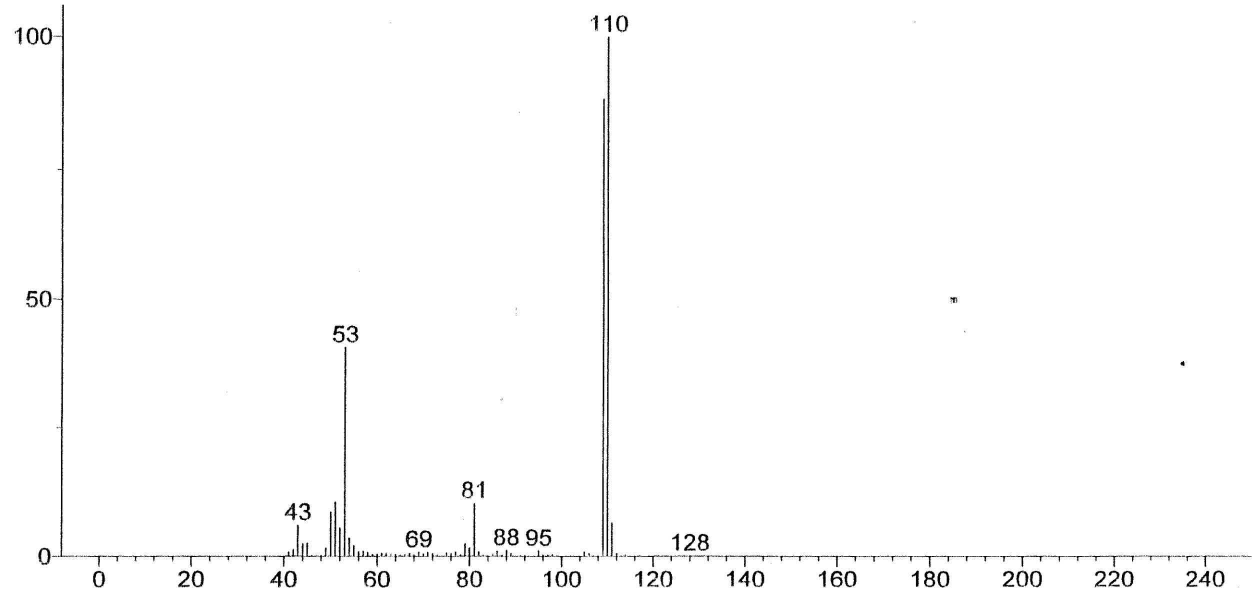

(Text File) +EI Scan (13.092 min) ALI-OLE-H7-220421-.D

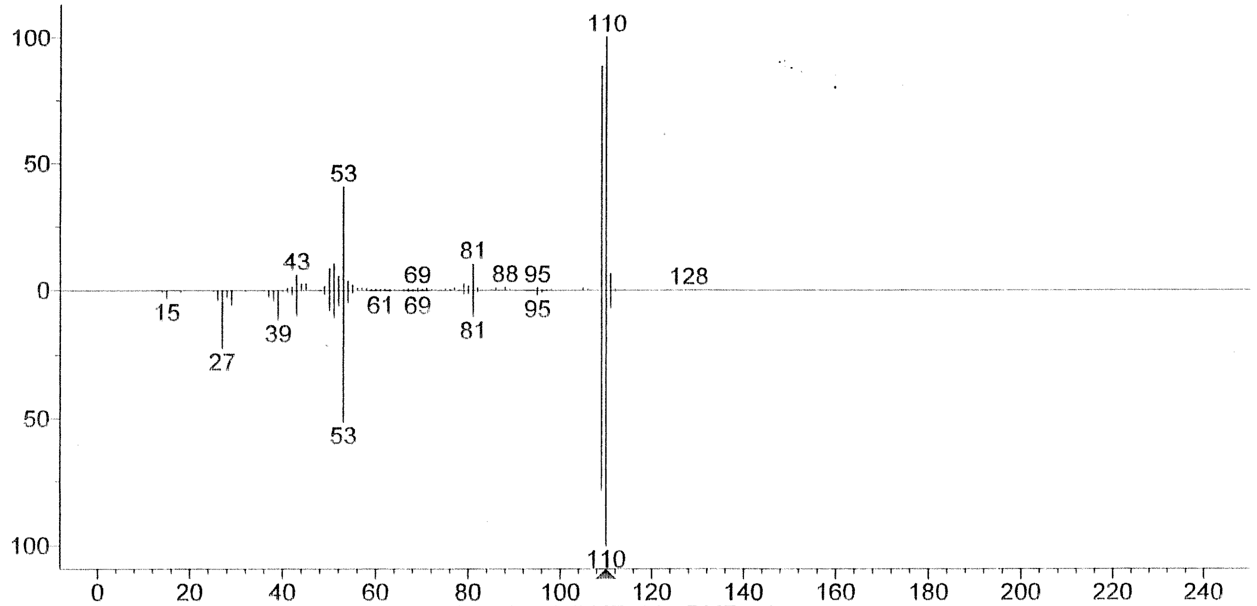

Head to Tail MF=905 RMF=925

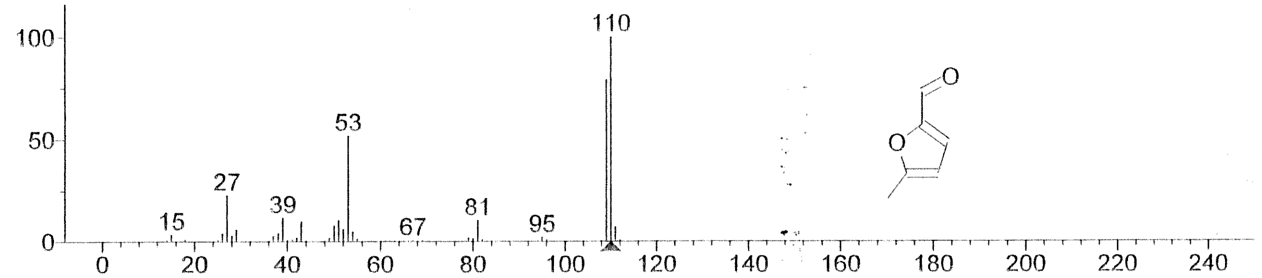

(mainlib) 2-Furancarboxaldehyde, 5-methyl-

Name: 2-Furancarboxaldehyde, 5-methyl-
Formula: $C_6H_6O_2$
MW: 110 CAS#: 620-02-0 NIST#: 233793 ID#: 74019 DB: mainlib
Other DBs: Fine, TSCA, RTECS, HODOC, EINECS, IRDB
Contributor: Japan AIST/NIMC Database- Spectrum MS-NW-1340
10 largest peaks:
110 999 | 109 788 | 53 515 | 27 225 | 39 115 | 51 104 | 81 102 | 43 97 | 50 77 | 111 69 |
Synonyms:
1.2-Furaldehyde, 5-methyl-
2.Furfural, 5-methyl-
3.2-Formyl-5-methylfuran
4.2-Methyl-5-formylfuran
5.5-Methyl-2-furaldehyde
6.5-Methyl-2-furancarboxaldehyde
7.5-Methyl-2-furfural
8.5-Methylfurfural
9.5-Methylfurfuraldehyde
10.5-Methyl furfural

Estimated non-polar retention index (n-alkane scale):
Value: 920 iu
Confidence interval (Diverse functional groups): 89(50%) 382(95%) iu

Retention index.
1. Value: 924 iu
Column Type: Capillary
Column Class: Standard non-polar
Active Phase: CP Sil 5 CB
Column
Length: 50 m
Carrier Gas: He
Column Diameter: 0.32 mm
Phase Thickness: 0.4 um
Data Type: Linear
RI
Program Type: Ramp
Start T: 60 C
End T: 280 C
Heat Rate: 3 K/min
Start Time: 10 min
End Time: 60
min
Source: Pino, J.A.; Marbot, R.; Vázquez, C., Characterization of volatile in Cosa Rican Guava [Psidium friedrichsthalianum (Berg) Niedenzu] fruit, J. Agric. Food Chem., 50, 2002, 6023-6026.

2. Value: 926 iu
Column
Type: Capillary
Column Class: Standard non-polar
Active Phase: SPB-1
Column Length: 60 m
Carrier Gas:
N2
Column Diameter: 0.32 mm

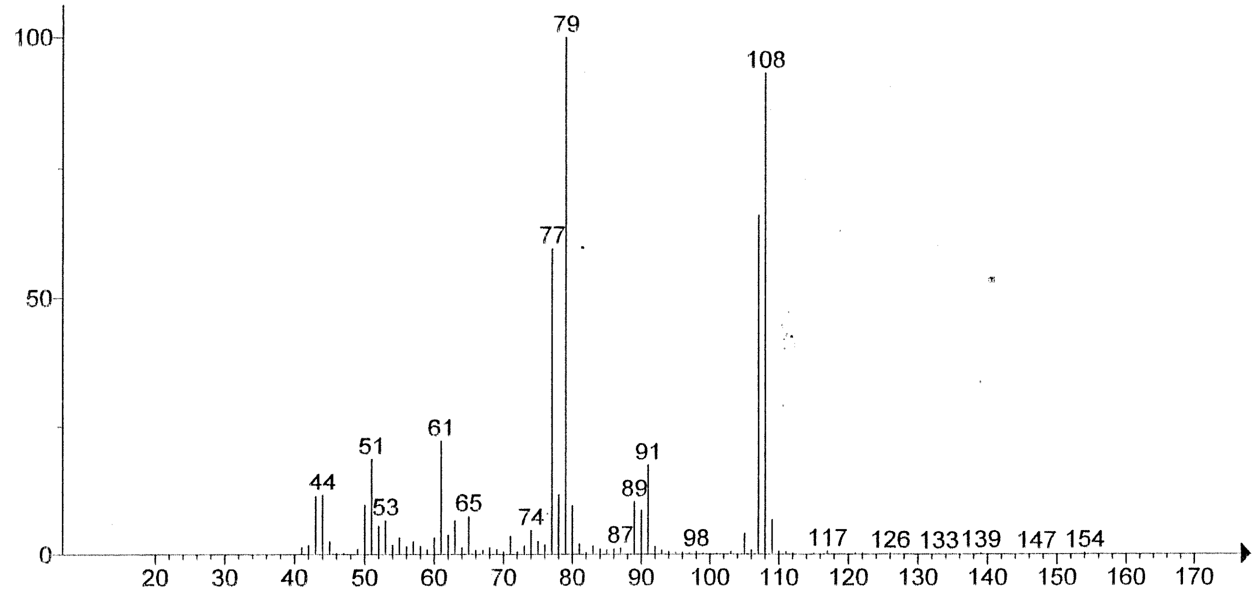

(Text File) +EI Scan (16.110 min) ALI-OLE-H7-220421-.D

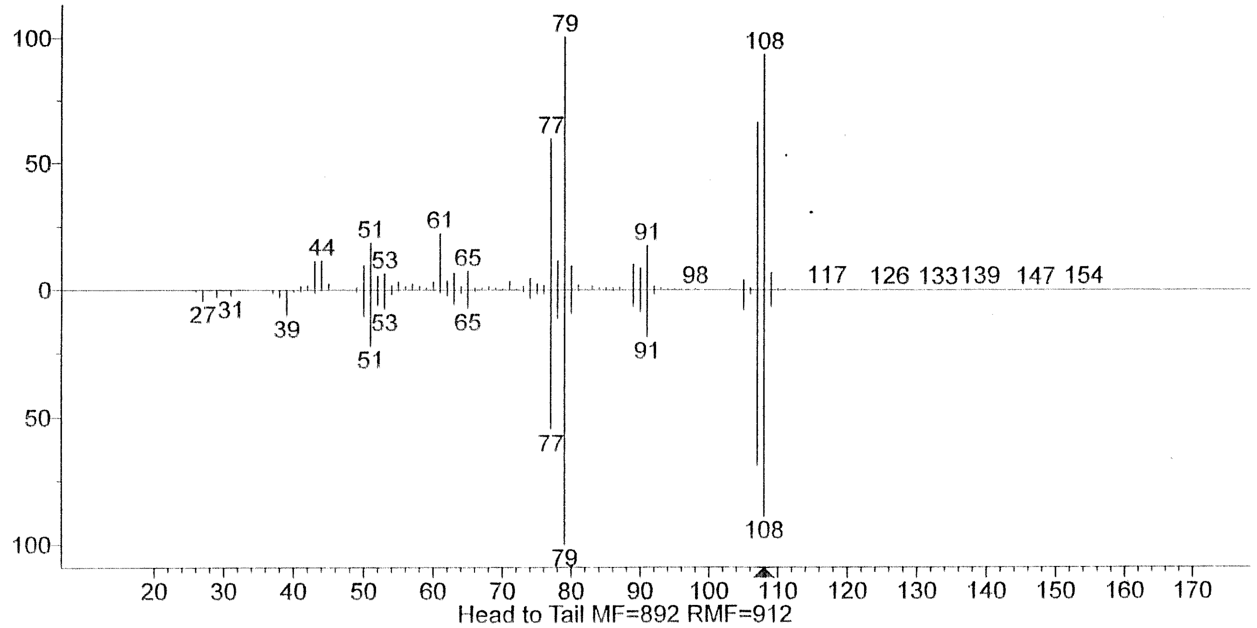

Head to Tail MF=892 RMF=912

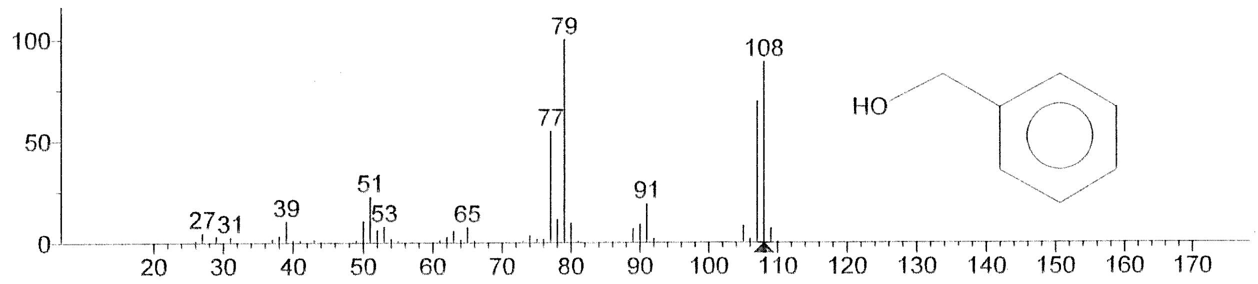

(mainlib) Benzyl Alcohol

Name: Benzyl Alcohol
Formula: C_7H_8O
MW: 108 CAS#: 100-51-6 NIST#: 151560 ID#: 42192 DB: mainlib
Other DBs: Fine, TSCA, RTECS, EPA, USP, HODOC, NIH, EINECS, IRDB
Contributor: Chemical Concepts
10 largest peaks:
79 999 | 108 890 | 107 692 | 77 547 | 51 222 | 91 187 | 78 113 | 50 104 | 39 102 | 80 95 |
Synonyms:
1.Benzenemethanol
2.α-Hydroxytoluene
3.α-Toluenol
4.(Hydroxymethyl)benzene
5.Benzenecarbinol
6.Phenylcarbinol
7.Phenylmethanol
8.Phenylmethyl alcohol
9.Methanol, phenyl-
10.NCI-C06111
11.Hydroxytoluene
12.Benzylalcohol
13.Bentalol
14.Benzoyl alcohol

Estimated non-polar retention index (n-alkane scale):
Value: 1036 iu
Confidence interval (Alcohols): 41(50%) 176(95%) iu

Retention index.
1. Value: 1005.7 iu
Column Type: Capillary
Column Class: Standard non-polar
Active Phase: DB-Petro
Column
Length: 50 m
Column Diameter: 0.2 mm
Phase Thickness: 0.5 um
Data Type: Linear RI
Program Type:
Ramp
Start T: 50 C
End T: 270 C
Heat Rate: 2 K/min
Source: Pang T.; Zhu S.; Lu X.; Xu G., Identification of unknown compounds on the basis of retention index data in comprehensive two-dimensional gas chromatography, J. Sep. Sci., 30, 2007, 868-874.

2. Value: 1005.7 iu
Column Type: Capillary
Column Class: Standard non
-polar
Active Phase: DB-Petro
Column Length: 50 m
Column Diameter: 0.2 mm
Phase Thickness: 0.5 um

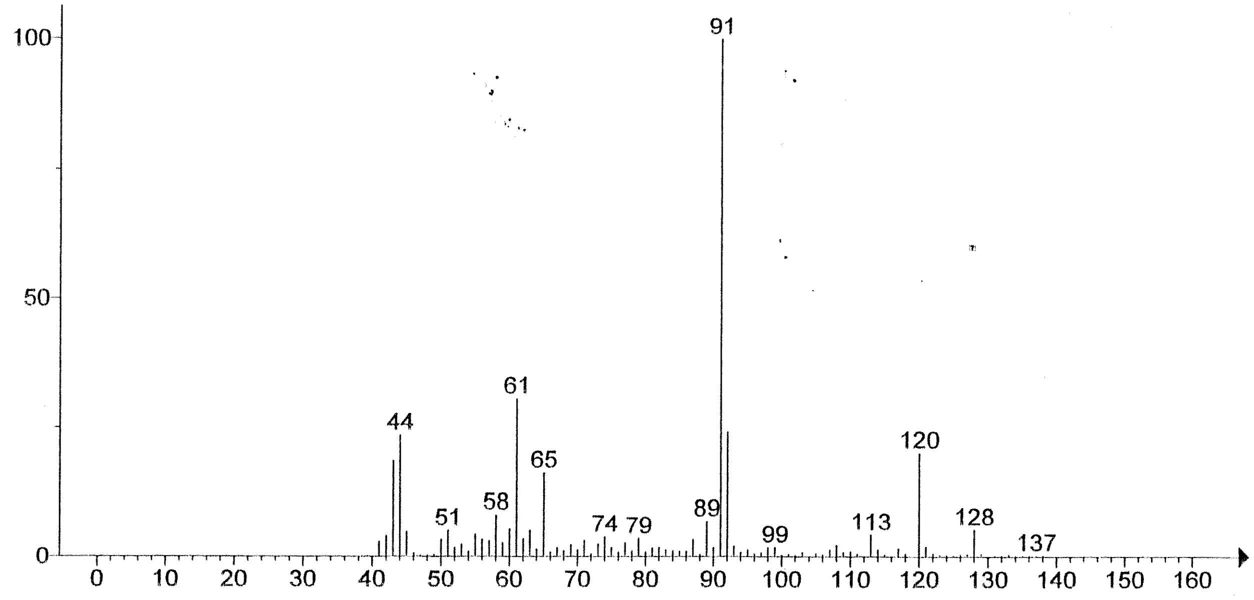

(Text File) +EI Scan (16.534 min) ALI-OLE-H7-220421-.D

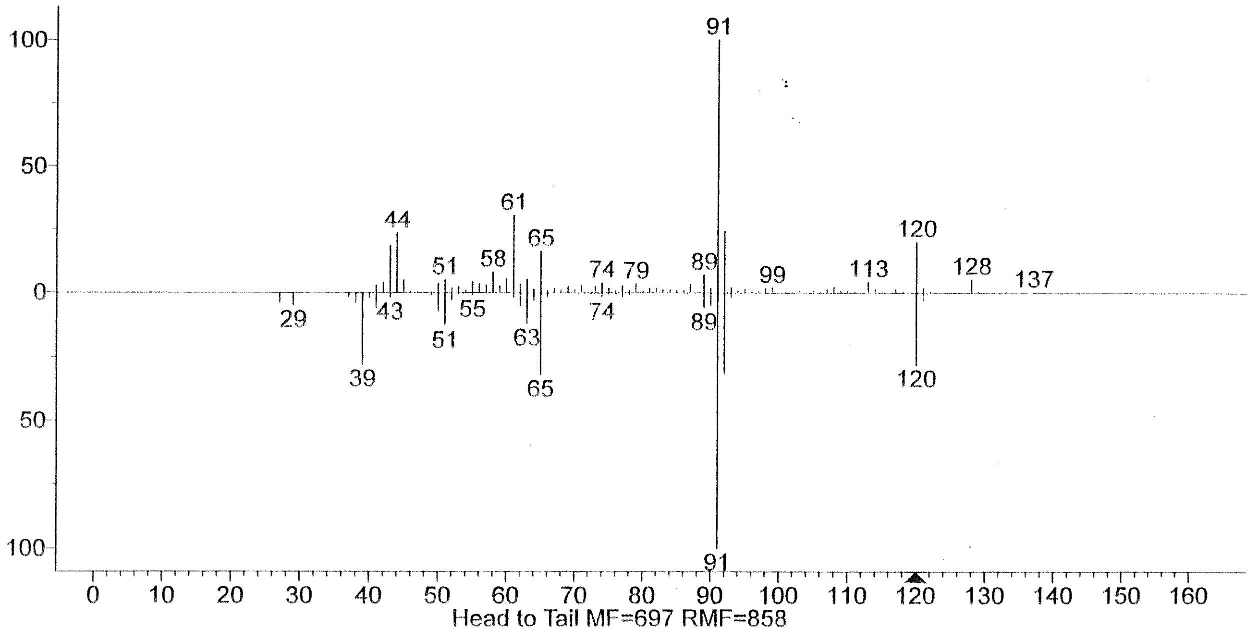

Head to Tail MF=697 RMF=858

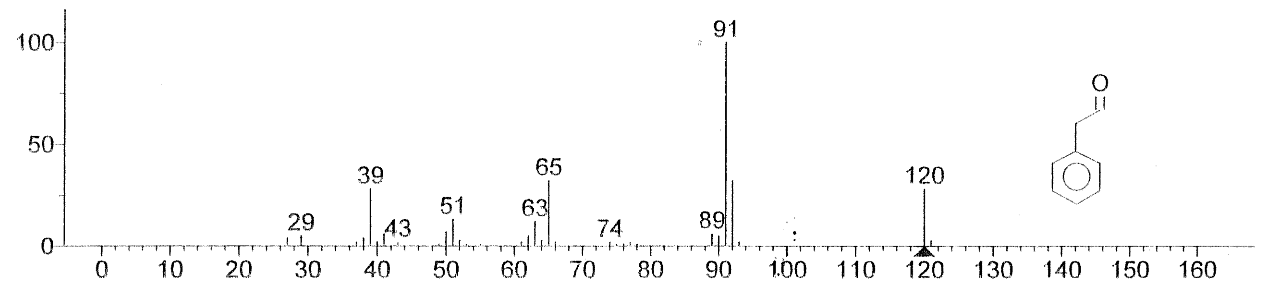

(replib) Benzeneacetaldehyde

Name: Benzeneacetaldehyde
Formula: C_8H_8O
MW: 120 CAS#: 122-78-1 NIST#: 53623 ID#: 11325 DB: replib
Other DBs: Fine, TSCA, RTECS, HODOC, NIH, EINECS, IRDB
Contributor: FOOD RESEARCH REPORT 122, K.E. MURRAY & COLLEAGUES, DIV OF FOOD RESEARCH, CSIRO, AUSTRALIA
10 largest peaks:
91 999 | 65 320 | 92 320 | 39 280 | 120 280 | 51 130 | 63 120 | 50 70 | 41 60 | 89 60 |
Synonyms:
1.Acetaldehyde, phenyl-
2.α-Tolualdehyde
3.α-Toluic aldehyde
4.Hyacinthin
5.Phenylacetaldehyde
6.Phenylacetic aldehyde
7.Phenylethanal
8.2-Phenylethanal
9.PAA
10.Phenyl acetaldehyde

Estimated non-polar retention index (n-alkane scale):
Value: 1081 iu
Confidence interval (Aldehydes): 45(50%) 196(95%) iu

Retention index.
1. Value: 1048 iu
Column Type: Capillary
Column Class: Standard non-polar
Active Phase: DB-1
Column
Length: 30 m
Carrier Gas: He
Column Diameter: 0.25 mm
Phase Thickness: 0.25 um
Data Type: Linear
RI
Program Type: Ramp
Start T: 60 C
End T: 240 C
Heat Rate: 4 K/min
Source: Rezazadeh, S.; Hamedani, M.
P.; Dowlatabadi, R.; Yazdani, D.; Shafiee, A., Chemical composition of the essential oils of Stachys schtschegleevii Sosn. and Stachys balansae Boiss & Kotschy from Iran, Flavour Fragr. J., 21, 2006, 290-293.

2. Value: 1012
iu
Column Type: Capillary
Column Class: Standard non-polar
Active Phase: CP Sil 5 CB
Column Length: 25
m
Carrier Gas: H2
Column Diameter: 0.25 mm
Data Type: Linear RI

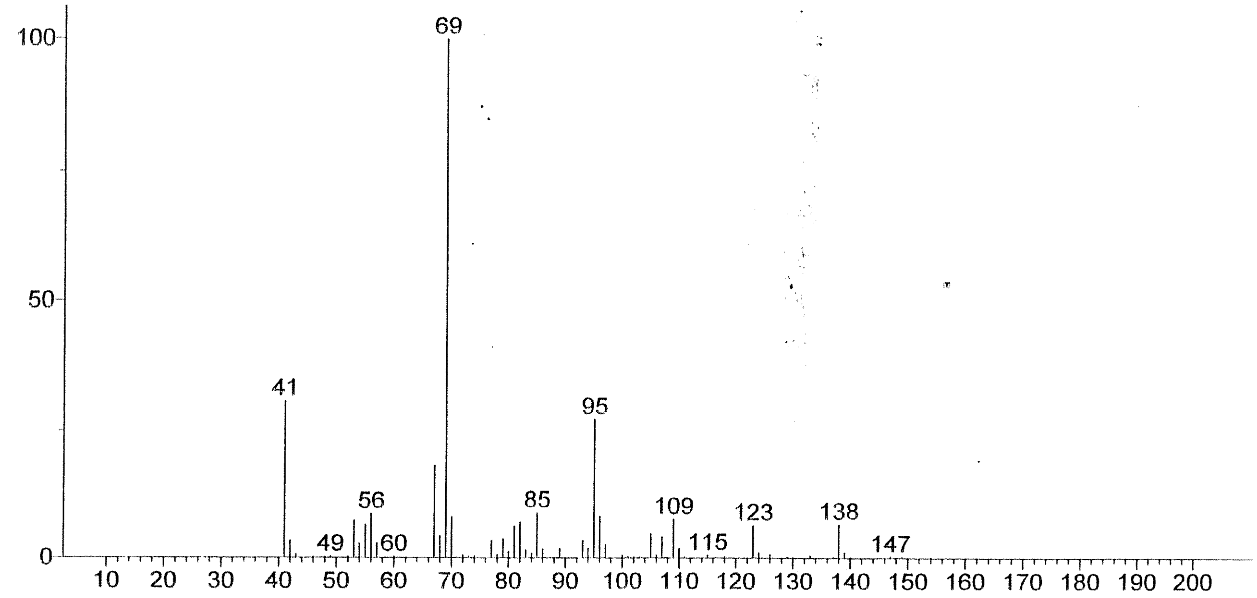

(Text File) +EI Scan (16.877 min) ALI-OLE-H7-220421-.D Subtract

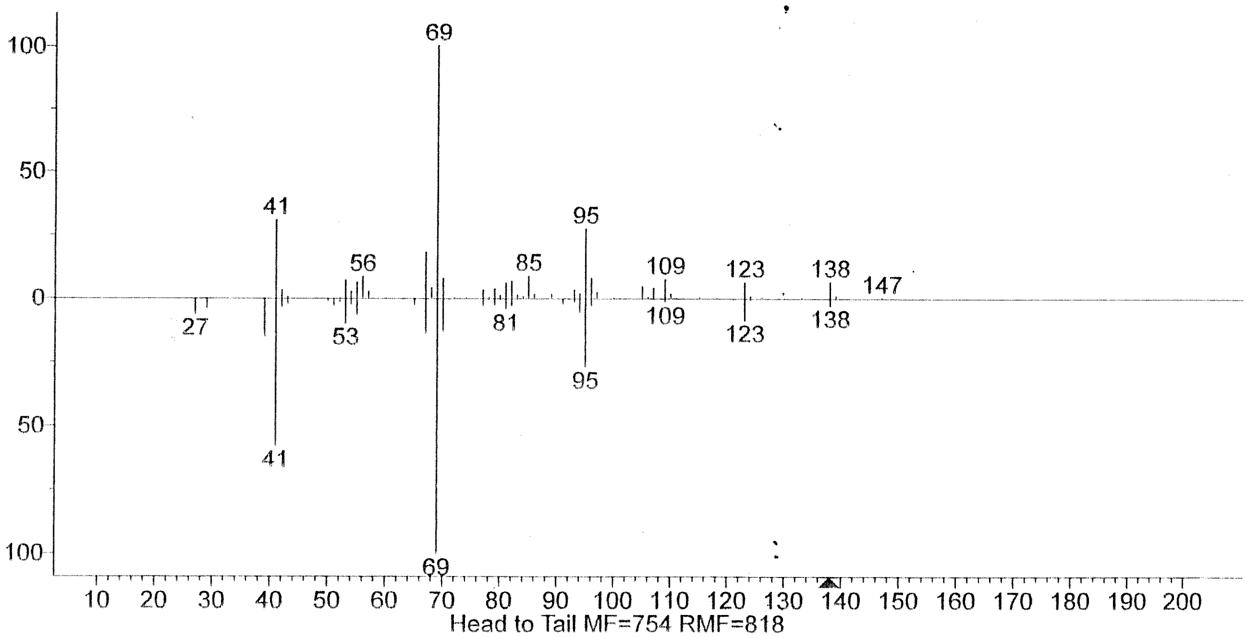

Head to Tail MF=754 RMF=818

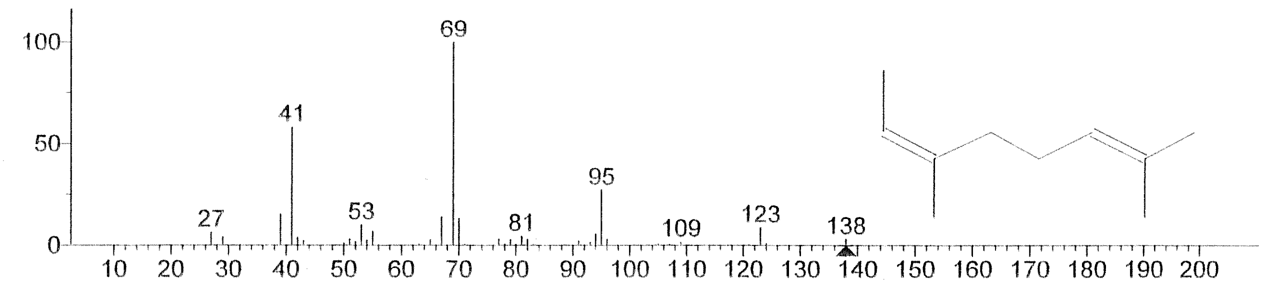

(mainlib) cis-2,6-Dimethyl-2,6-octadiene

Name: cis-2,6-Dimethyl-2,6-octadiene
Formula: $C_{10}H_{18}$
MW: 138 CAS#: 2492-22-0 NIST#: 114539 ID#: 30178 DB: mainlib
Other DBs: IRDB
Contributor: NIST Mass Spectrometry Data Center, 1990.
10 largest peaks:
69 999 | 41 578 | 95 270 | 39 150 | 67 138 | 70 129 | 53 97 | 123 84 | 55 65 | 27 61 |
Synonyms:
1.(6Z)-2,6-Dimethyl-2,6-octadiene #

Estimated non-polar retention index (n-alkane scale):
Value: 985 iu
Confidence interval (Hydrocarbons): 39(50%) 167(95%) iu

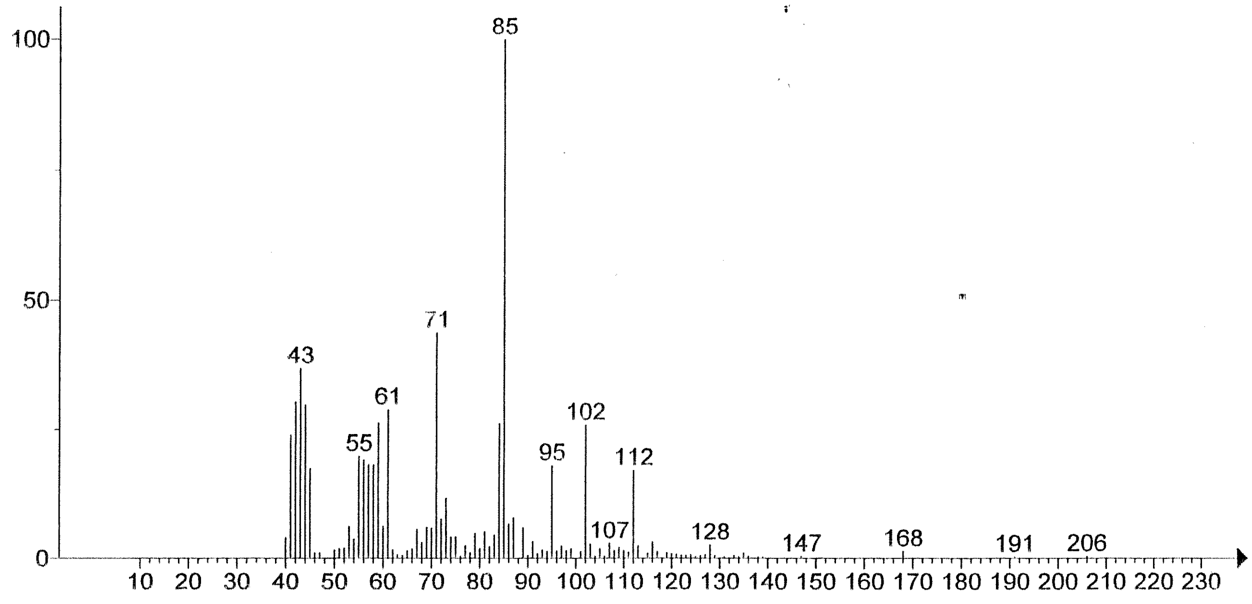

(Text File) +EI Scan (17.547 min) ALI-OLE-H7-220421-.D

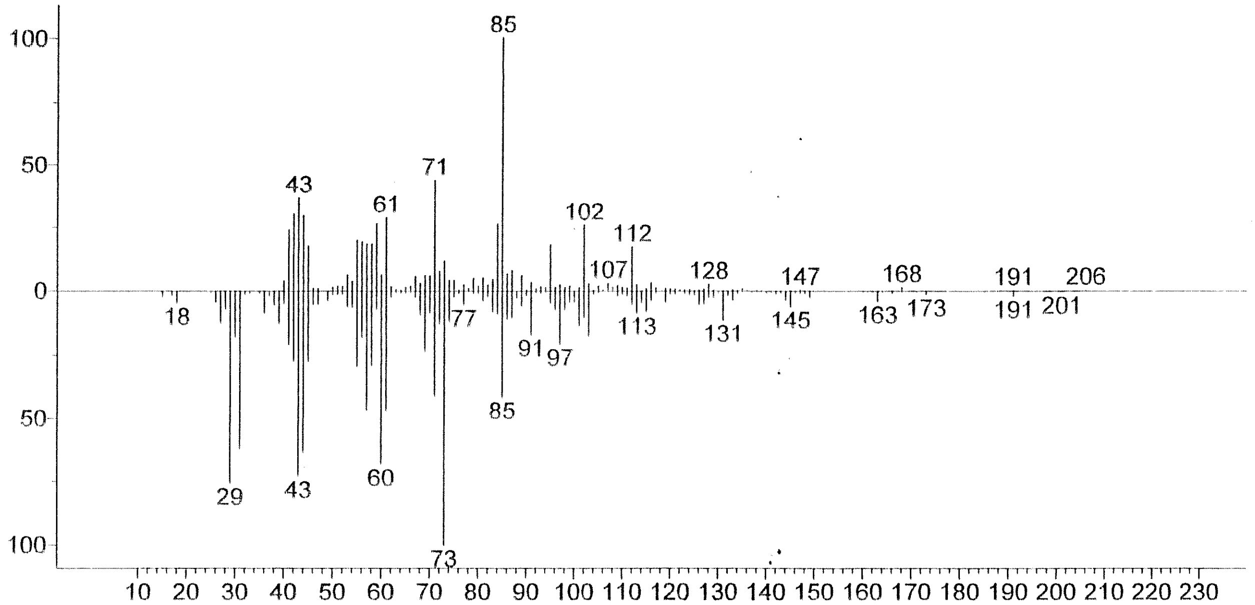

Head to Tail MF=676 RMF=678

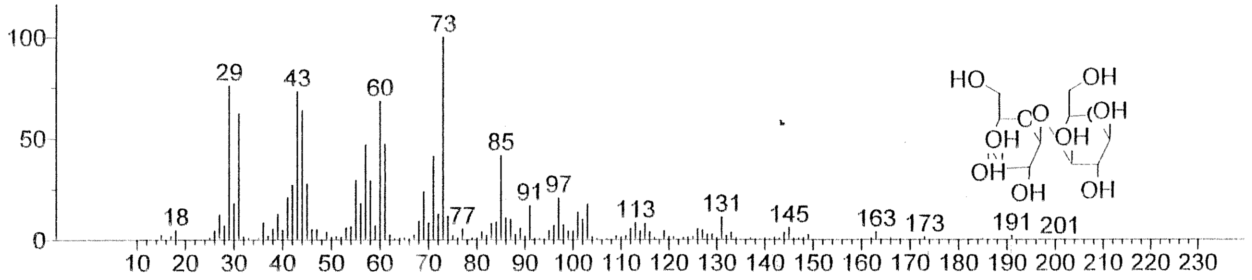

(replib) β-D-Glucopyranose, 4-O-β-D-galactopyranosyl-

Name: β-D-Glucopyranose, 4-O-β-D-galactopyranosyl-
Formula: $C_{12}H_{22}O_{11}$
MW: 342 CAS#: 5965-66-2 NIST#: 76424 ID#: 8344 DB: replib
Other DBs: TSCA, HODOC, EINECS
Contributor: RADIAN CORP
10 largest peaks:
73 999 | 29 757 | 43 729 | 60 681 | 44 636 | 31 620 | 61 470 | 57 468 | 85 416 | 71 412 |
Synonyms:
1.Lactose, β-
2.β-Lactose
3.4-O-Hexopyranosylhexopyranose #

Estimated non-polar retention index (n-alkane scale):
Value: 3131 iu
Confidence interval (Diverse functional groups): 89(50%) 382(95%) iu

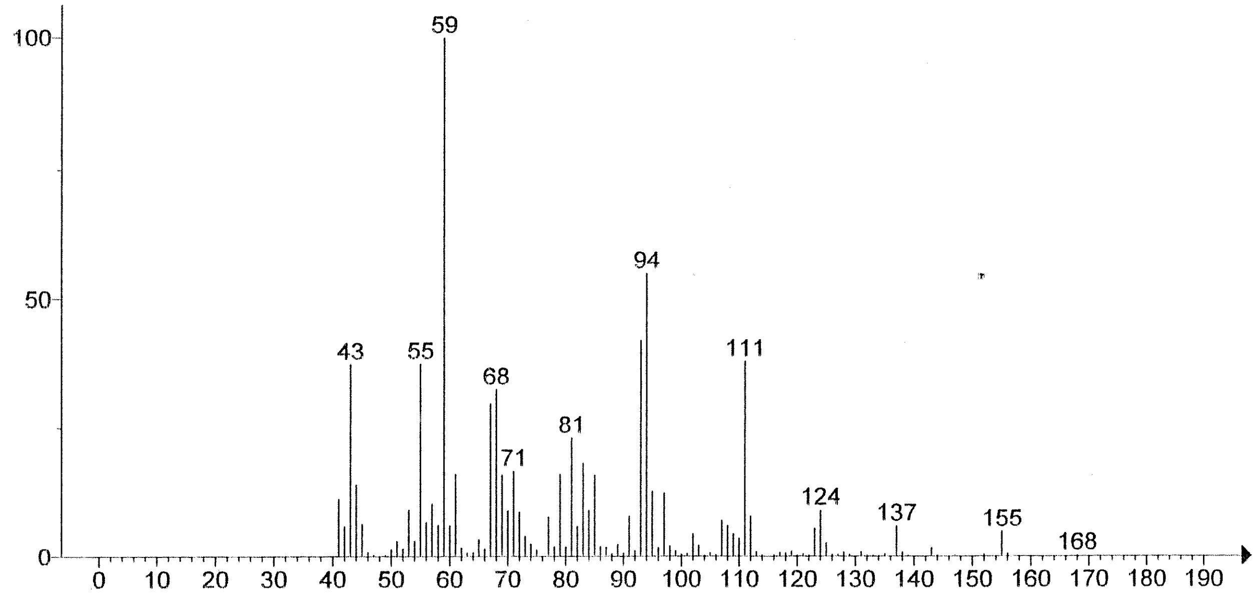

(Text File) +EI Scan (17.767 min) ALI-OLE-H7-220421-.D

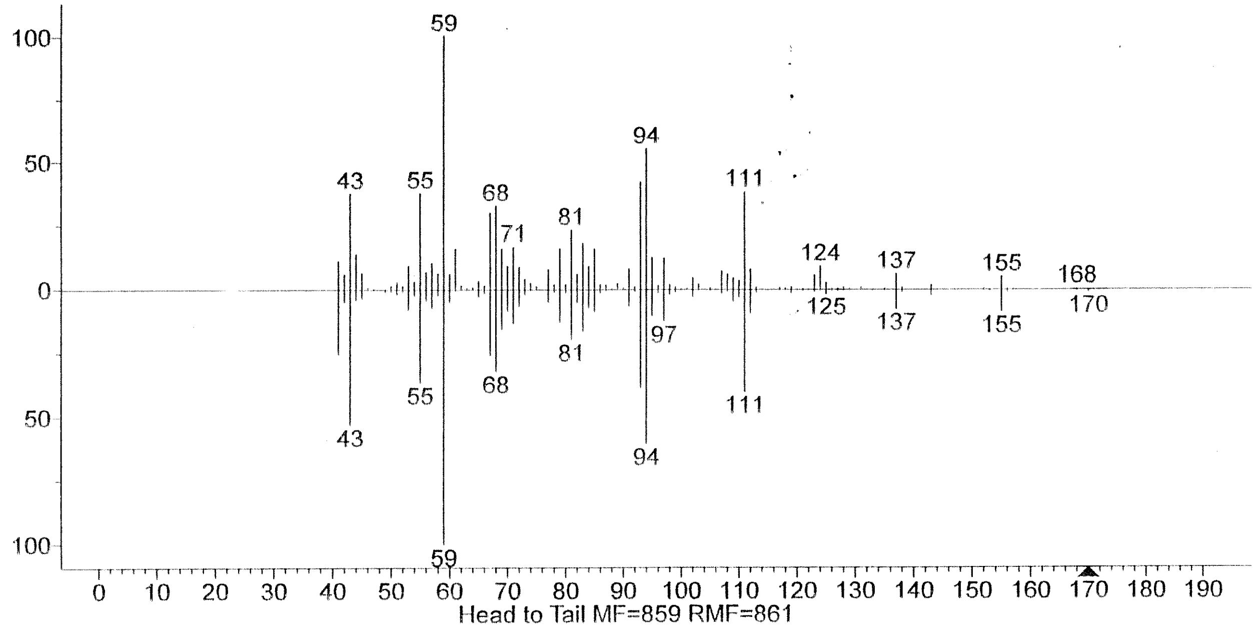

Head to Tail MF=859 RMF=861

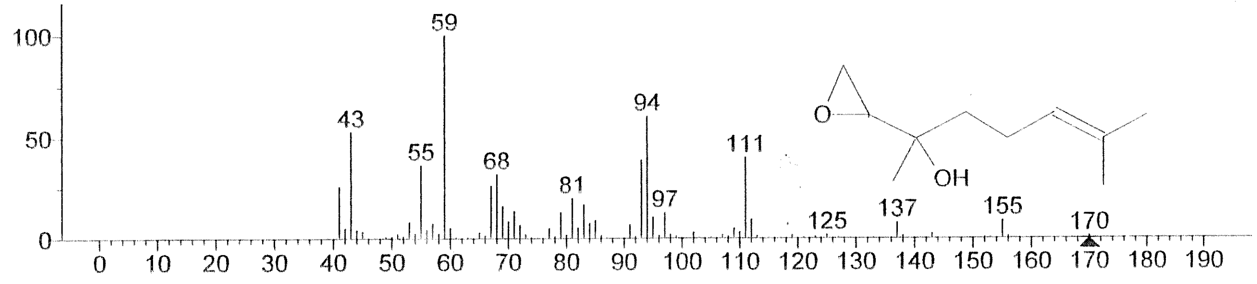

(mainlib) α-Methyl-α-[4-methyl-3-pentenyl]oxiranemethanol

Name: α-Methyl-α-[4-methyl-3-pentenyl]oxiranemethanol
Formula: $C_{10}H_{18}O_2$
MW: 170 NIST#: 132130 ID#: 26674 DB: mainlib
Contributor: Insect Chem. Ecol. Lab., USDA, Beltsville, MD 20705
10 largest peaks:
59 999 | 94 603 | 43 527 | 111 398 | 93 385 | 55 362 | 68 318 | 67 258 | 41 256 | 81 196 |
Synonyms:
no synonyms.

Estimated non-polar retention index (n-alkane scale):
Value: 1182 iu
Confidence interval (Diverse functional groups): 89(50%) 382(95%) iu

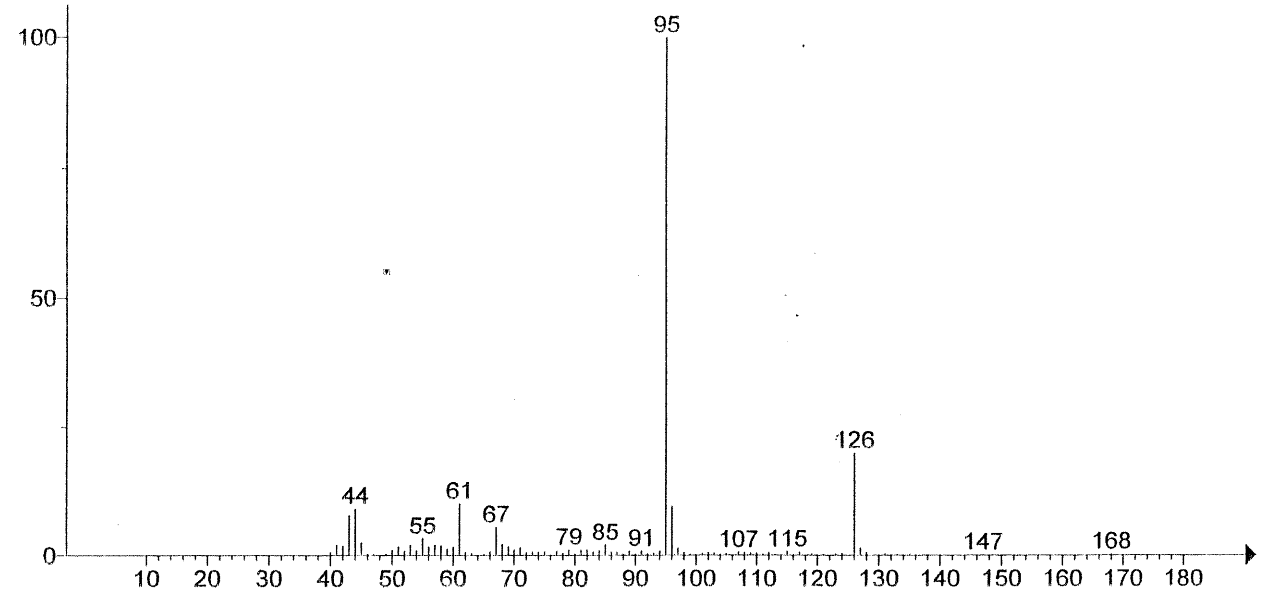

(Text File) +EI Scan (18.099 min) ALI-OLE-H7-220421-.D

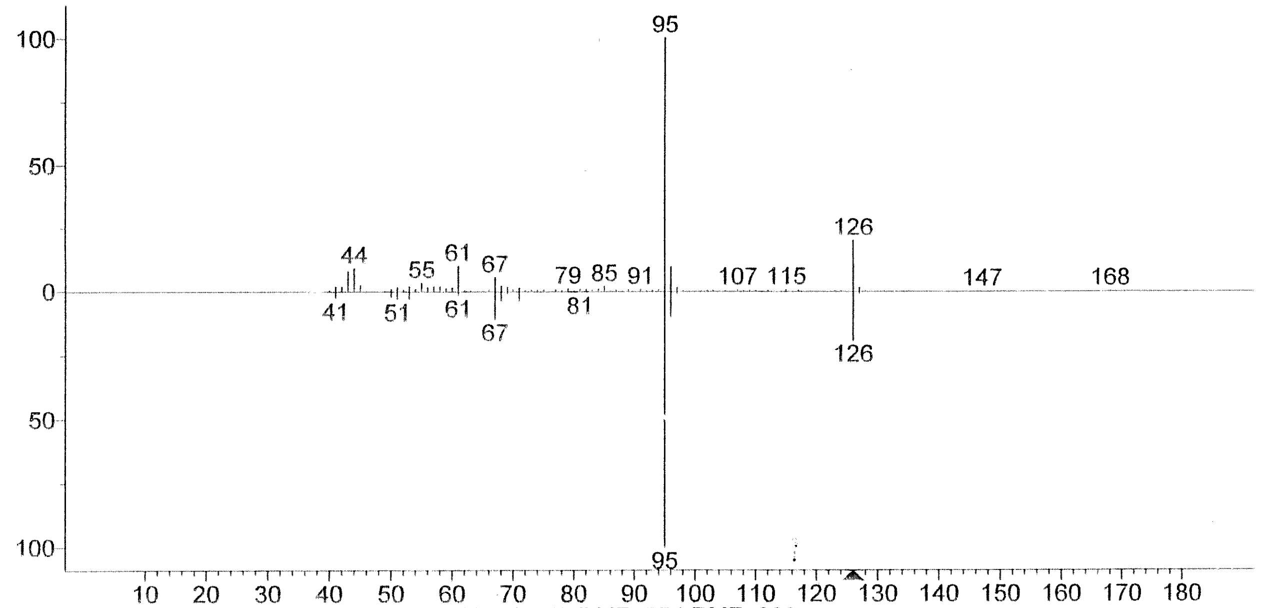

Head to Tail MF=754 RMF=888

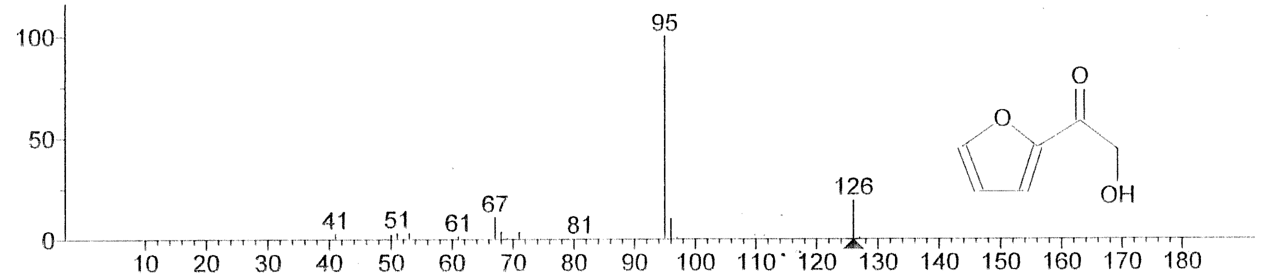

(mainlib) Furyl hydroxymethyl ketone

Name: Furyl hydroxymethyl ketone
Formula: $C_6H_6O_3$
MW: 126 CAS#: 17678-19-2 NIST#: 281423 ID#: 58741 DB: mainlib
Other DBs: None
Contributor: I.Cutzach ET AL.J.Agric.Food Chem.,47,1663(1999)
10 largest peaks:
95 999 | 126 190 | 67 107 | 96 100 | 68 36 | 71 36 | 51 29 | 53 29 | 41 26 | 50 21 |
Synonyms:
1.Ethanone, 1-(2-furanyl)-2-hydroxy-
2.Ketone, 2-furyl hydroxymethyl
3.2-(Hydroxyacetyl)furan
4.2-Furyl hydroxymethyl ketone
5.2-(2'-Hydroxyacetyl)furan
6.2-(1-Oxo-2-hydroxyethyl)furan
7.1-(2-Furyl)-2-hydroxyethanone #

Estimated non-polar retention index (n-alkane scale):
Value: 1121 iu
Confidence interval (Diverse functional groups): 89(50%) 382(95%) iu

Retention index.
1. Value: 1070 iu
Column Type: Capillary
Column Class: Standard non-polar
Active Phase: DB-1
Column
Length: 60 m
Carrier Gas: He
Column Diameter: 0.32 mm
Phase Thickness: 1 um
Data Type: Normal alkane
RI
Program Type: Ramp
Start T: 40 C
End T: 260 C
Heat Rate: 2 K/min
Source: Chen, J.; Ho, C.-T.,
Comparison of volatile generation in serine/threonine/glutamine-ribose/glucose/fructose model systems, J. Agric. Food Chem., 47, 1999, 643-647.

2. Value: 1056 iu
Column Type: Capillary
Column Class: Standard non
-polar
Active Phase: DB-1
Column Length: 60 m
Carrier Gas: He
Column Diameter: 0.32 mm
Phase Thickness:
1.0 um
Data Type: Normal alkane RI
Program Type: Ramp
Start T: 40 C
End T: 280 C

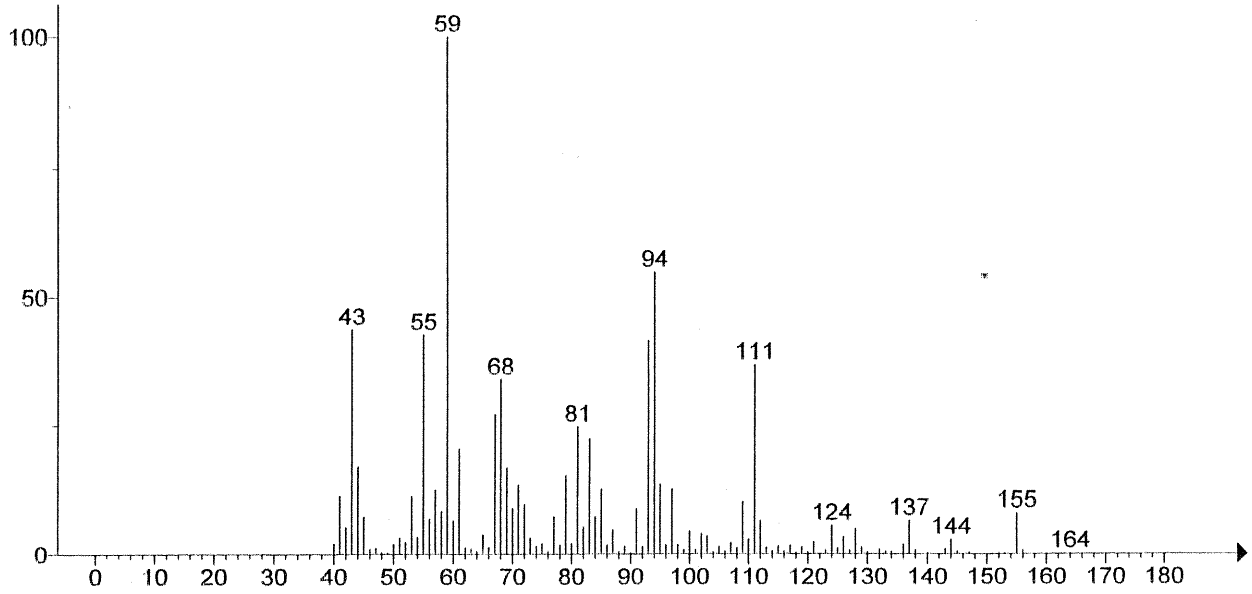

(Text File) +EI Scan (18.398 min) ALI-OLE-H7-220421-.D

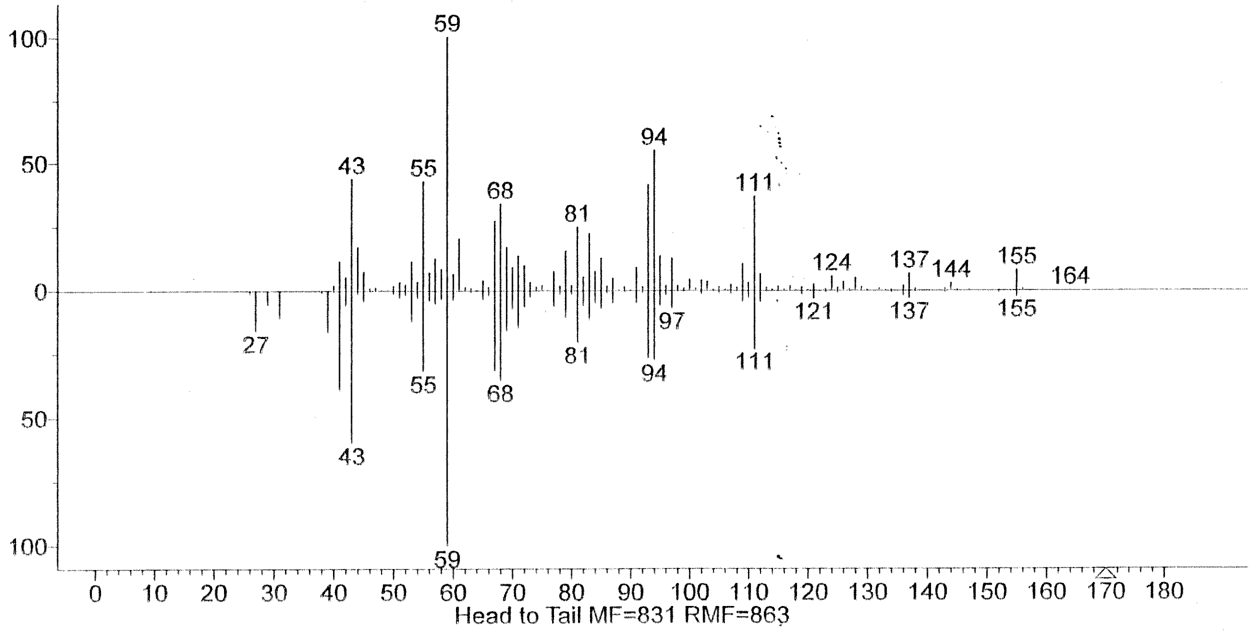

Head to Tail MF=831 RMF=863

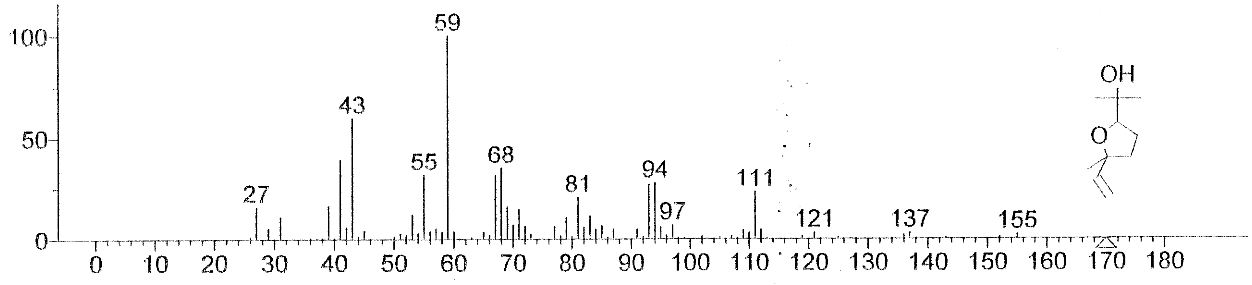

(mainlib) 2-Furanmethanol, 5-ethenyltetrahydro-α,α,5-trimethyl-, cis-

Name: 2-Furanmethanol, 5-ethenyltetrahydro-α,α,5-trimethyl-, cis-
Formula: $C_{10}H_{18}O_2$
MW: 170 CAS#: 5989-33-3 NIST#: 155746 ID#: 26148 DB: mainlib
Other DBs: TSCA, EINECS
Contributor: Chemical Concepts
10 largest peaks:
59 999 | 43 595 | 41 391 | 68 351 | 55 316 | 67 314 | 94 274 | 93 266 | 111 228 | 81 204 |
Synonyms:
1.Furfuryl alcohol, tetrahydro-α,α,5-trimethyl-5-vinyl-
2.cis-Linalool Oxide
3.cis-Linalyl Oxide
4.cis-5-Ethenyltetrahydro-α,α,5-Trimethyl-2-furanmethanol
5.Linalool oxide
6.Linalool oxide B
7.Linalyl oxide
8.Furfuryl alcohol, tetrahydro-α,α,5-trimethyl-5-vinyl- cis-5-Ethenyltetrahydro-α,α,5-trimethyl-2-furanmethanol
9.Linalool oxide cis
10.Linalool oxide, (Z)-
11.2-(5-Methyl-5-vinyltetrahydro-2-furanyl)-2-propanol #

Estimated non-polar retention index (n-alkane scale):
Value: 1164 iu
Confidence interval (Diverse functional groups): 89(50%) 382(95%) iu

Retention index.
1. Value: 1064 iu
Column Type: Capillary
Column Class: Standard non-polar
Active Phase: CP-Sil PONA
GB
Column Length: 100 m
Carrier Gas: He
Column Diameter: 0.25 mm
Phase Thickness: 0.25 um
Data Type:
Linear RI
Program Type: Ramp
Start T: 140 C
End T: 230 C
Heat Rate: 5 K/min
Start Time: 10 min
End Time:
25 min
Source: Cunicao, M.M.; Lopes, A.R.; Côcco, L.C.; Yamamoto, C.I.; Plocharski, R.C.B.; Miguel, M.D.; Junior, A.G.; Auer, C.G.; Miguel, O.G., Phytochemical and antibacterial evaluation of essential oils from Ottonia Martiana Miq. (Piperaceae), J. Braz. Chem. Soc., 18(1), 2007, 184-188.

2. Value: 1089 iu
Column Type:
Capillary
Column Class: Standard non-polar
Active Phase: SPB-1
Column Length: 30 m
Carrier Gas:

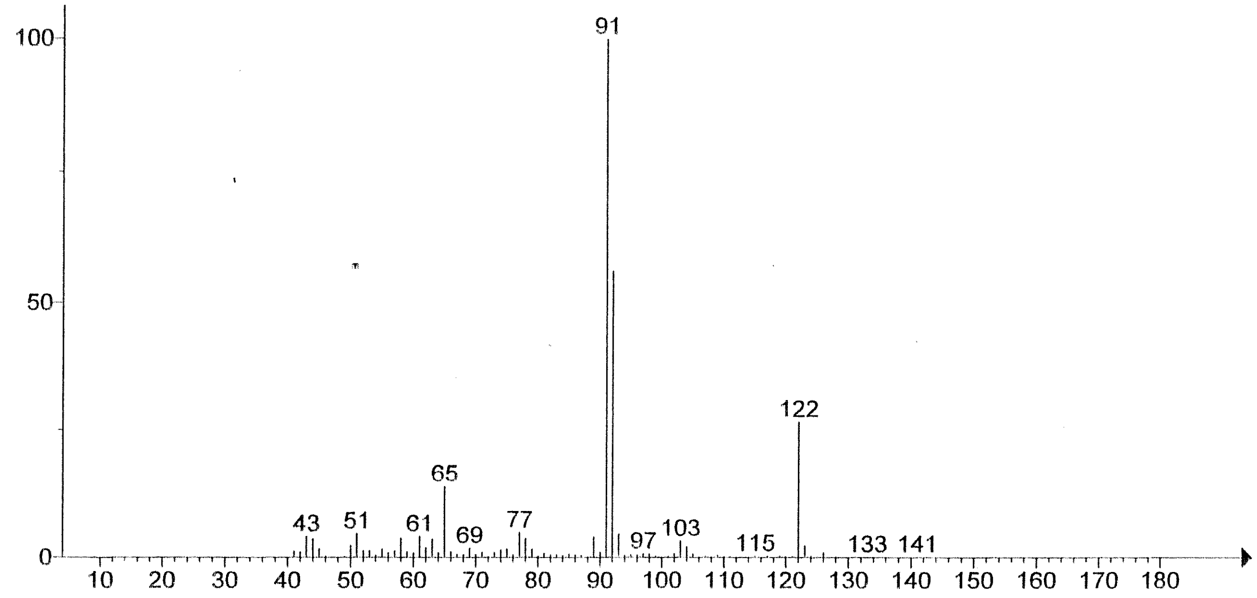

(Text File) +EI Scan (19.382 min) ALI-OLE-H7-220421-.D

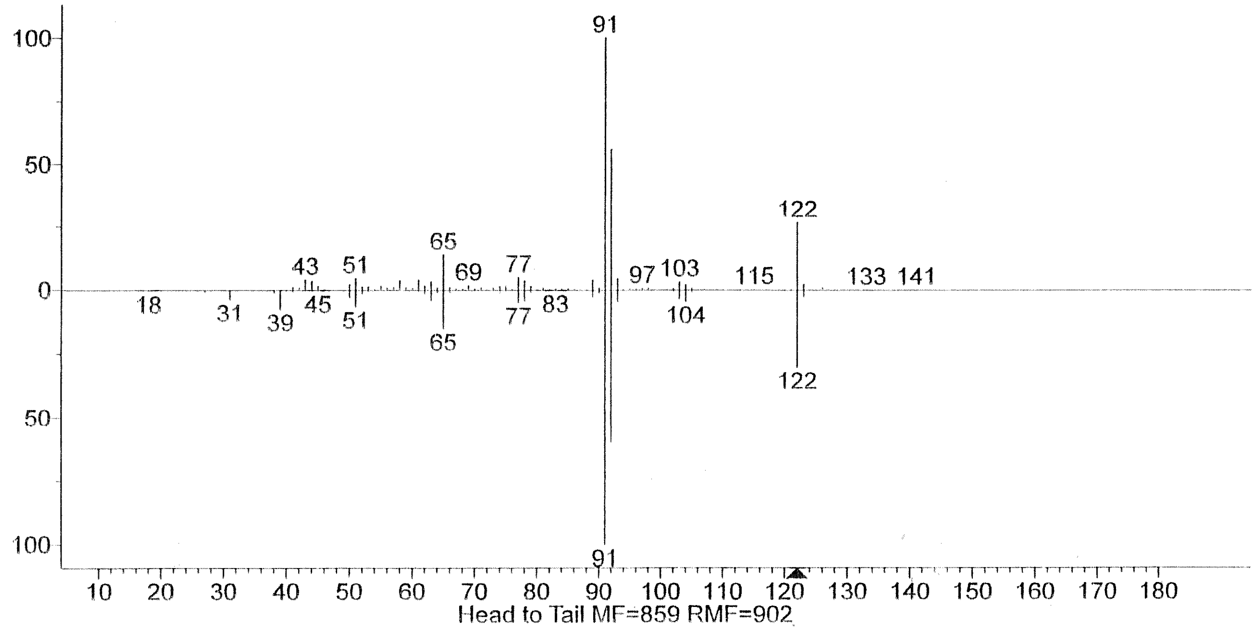

Head to Tail MF=859 RMF=902

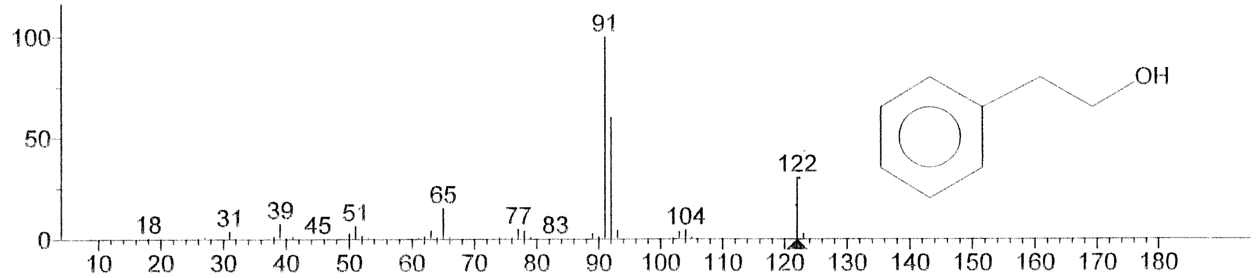

(replib) Phenylethyl Alcohol

Name: Phenylethyl Alcohol
Formula: $C_8H_{10}O$
MW: 122 CAS#: 60-12-8 NIST#: 229405 ID#: 11425 DB: replib
Other DBs: Fine, TSCA, RTECS, EPA, USP, HODOC, NIH, EINECS, IRDB
Contributor: Japan AIST/NIMC Database- Spectrum MS-NW- 710
10 largest peaks:
91 999 | 92 597 | 122 301 | 65 151 | 39 75 | 51 64 | 77 48 | 93 44 | 104 44 | 63 42 |
Synonyms:
1.Benzeneethanol
2.Phenethyl alcohol
3.β-Hydroxyethylbenzene
4.β-Phenethyl alcohol
5.β-Phenylethanol
6.β-Phenylethyl alcohol
7.β-PEA
8.Benzyl Carbinol
9.Ethanol, 2-phenyl-
10.Phenethanol
11.PEA
12.2-Phenethyl Alcohol
13.2-Phenylethanol
14.2-Phenylethyl alcohol
15.Methanol, benzyl-
16.Phenylethanol
17.Phenylethyl, b- alcohol
18.2-PEA
19.β-Fenethylalkohol
20.β-Fenylethanol
21.Orange oil
22.Rose oil
23.β-Phenethanol
24.2-Phenethanol
25.β-P.E.A.
26.1-Phenyl-2-ethanol
27.Hydroxyethylbenzene
28.Mellol
29.Beta-phenylethyl alcohol

Estimated non-polar retention index (n-alkane scale):
Value: 1136 iu
Confidence interval (Alcohols): 41(50%) 176(95%) iu

Retention index.
1. Value: 1082.4 iu
Column Type: Capillary
Column Class: Standard non-polar
Active Phase: DB-Petro
Column
Length: 50 m
Column Diameter: 0.2 mm
Phase Thickness: 0.5 um
Data Type: Linear RI
Program Type:
Ramp

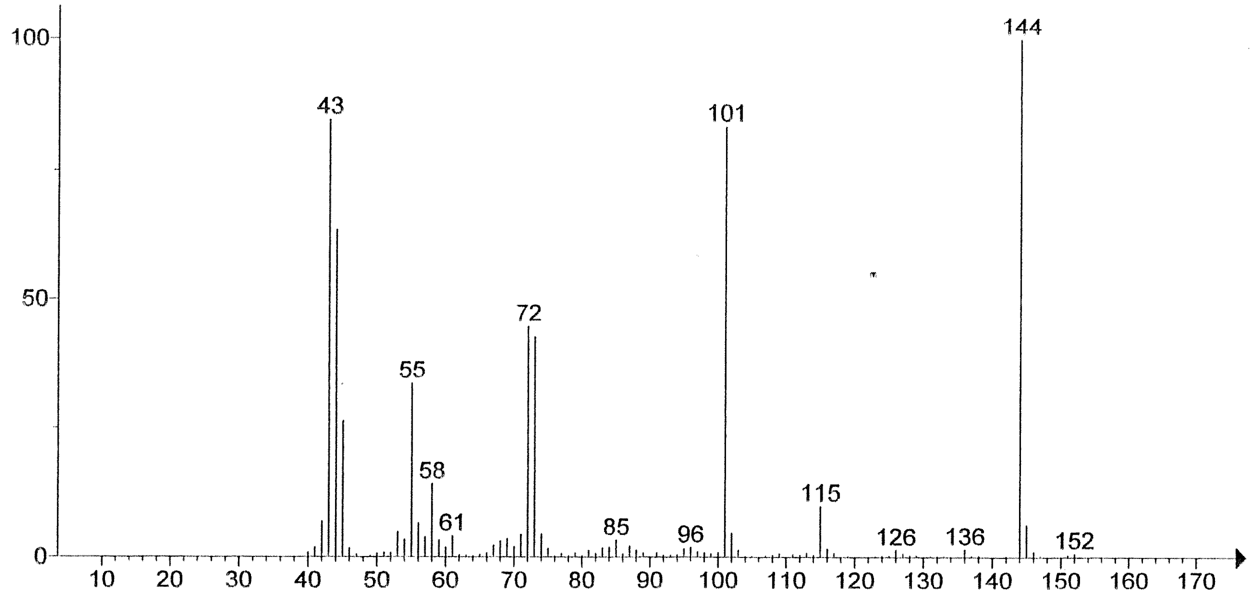

(Text File) +EI Scan (20.572 min) ALI-OLE-H7-220421-.D

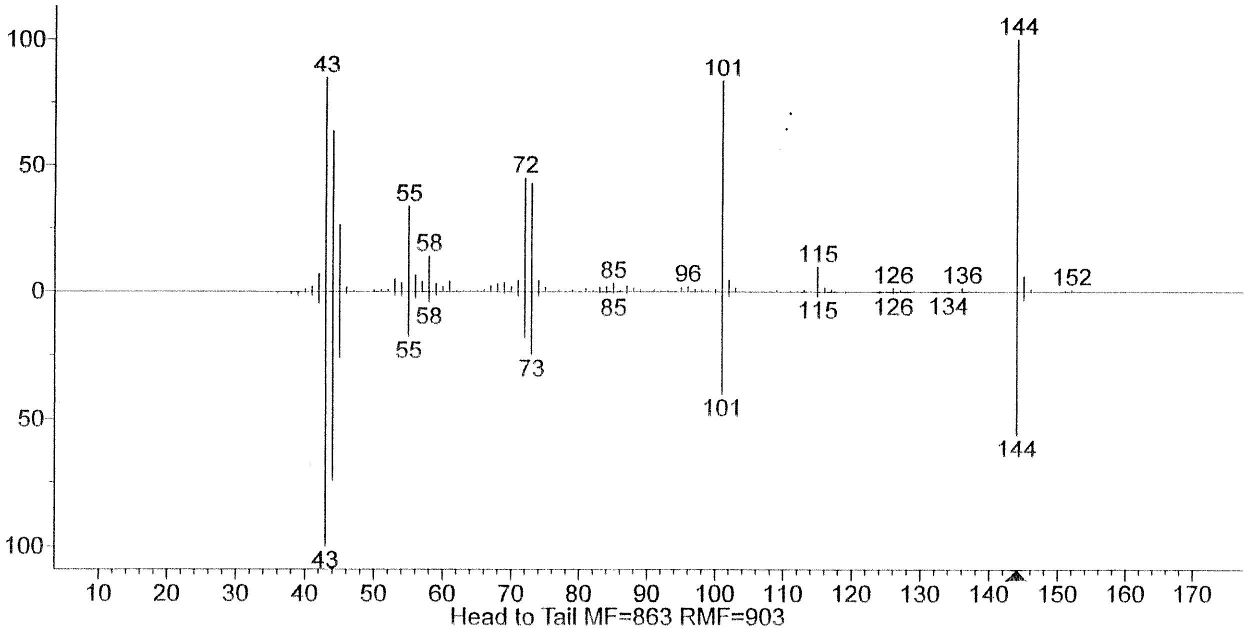

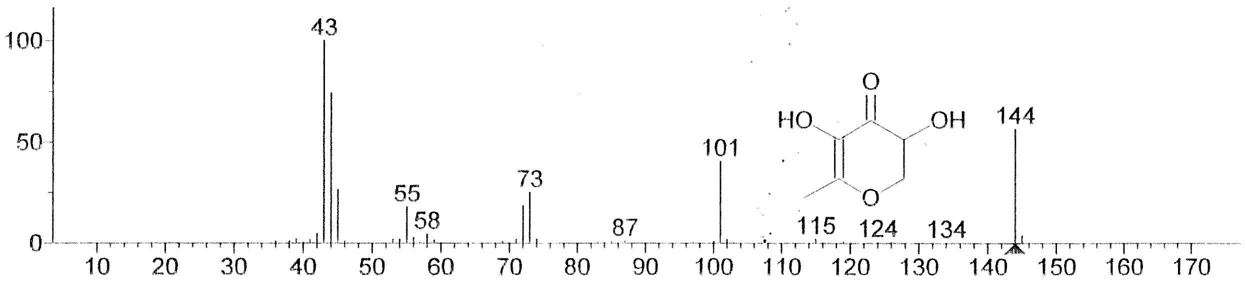

(replib) 4H-Pyran-4-one, 2,3-dihydro-3,5-dihydroxy-6-methyl-

Name: 4H-Pyran-4-one, 2,3-dihydro-3,5-dihydroxy-6-methyl-
Formula: $C_6H_8O_4$
MW: 144 CAS#: 28564-83-2 NIST#: 108691 ID#: 1857 DB: replib
Other DBs: RTECS
Contributor: Philip Morris R&D
10 largest peaks:
43 999 | 44 742 | 144 563 | 101 402 | 45 264 | 73 249 | 72 182 | 55 177 | 42 48 | 58 43 |
Synonyms:
1.3,5-Dihydroxy-6-methyl-2,3-dihydro-4H-pyran-4-one #

Estimated non-polar retention index (n-alkane scale):
Value: 1269 iu
Confidence interval (Diverse functional groups): 89(50%) 382(95%) iu

Retention index.
1. Value: 1119 iu
Column Type: Capillary
Column Class: Standard non-polar
Active Phase: DB-1
Column
Length: 60 m
Carrier Gas: He
Column Diameter: 0.25 mm
Phase Thickness: 0.25 um
Data Type: Linear
RI
Program Type: Complex
Description: -20C (5min) => 10C/min => 100C => 4C/min =>200C => 10C/min =>
280C
Source: Eri, S.; Khoo, B.K.; Lech, J.; Hartman, T.G., Direct thermal desorption-gas chromatography and gas chromatography-mass spectrometry profiling of hop (Humulus lupulus L.) essential oils in support of varietal characterization, J. Agric. Food Chem., 48, 2000, 1140-1149.

2. Value: 1107 iu
Column Type: Capillary
Column
Class: Standard non-polar
Active Phase: DB-1
Column Length: 60 m
Carrier Gas: N2
Column Diameter: 0.25
mm
Phase Thickness: 1 um
Data Type: Linear RI
Program Type: Ramp
Start T: 30 C
End T: 200 C
Heat
Rate: 5 K/min
End Time: 30 min
Source: Wu, C.-M.; Wang, Z.; Wu, Q.H., Volatile compounds produced from monosodium glutamate in common food cooking, J. Agric. Food Chem., 48, 2000, 2438-2442.

<...>

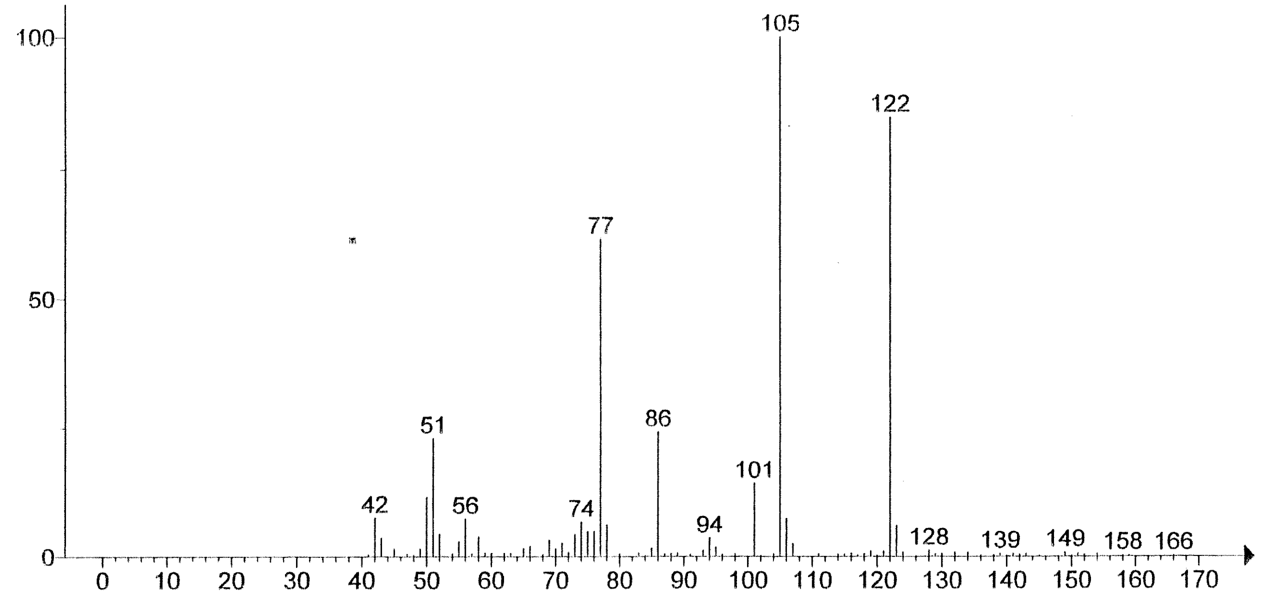

(Text File) +EI Scan (21.467 min) ALI-OLE-H7-220421-.D Subtract

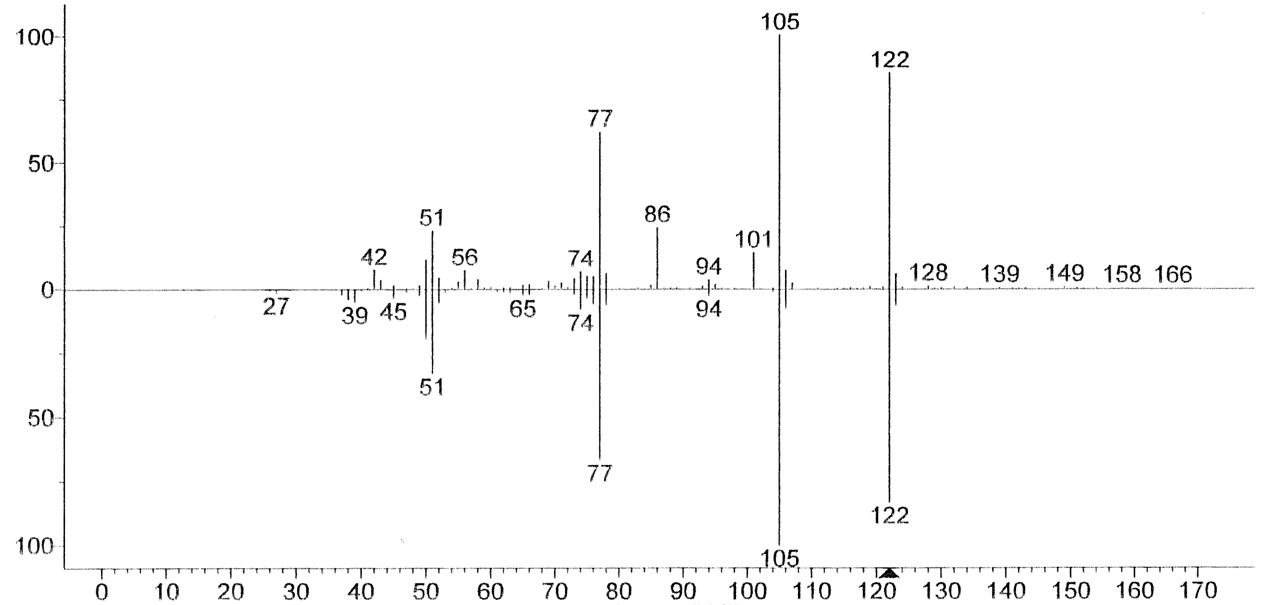

Head to Tail MF=782 RMF=900

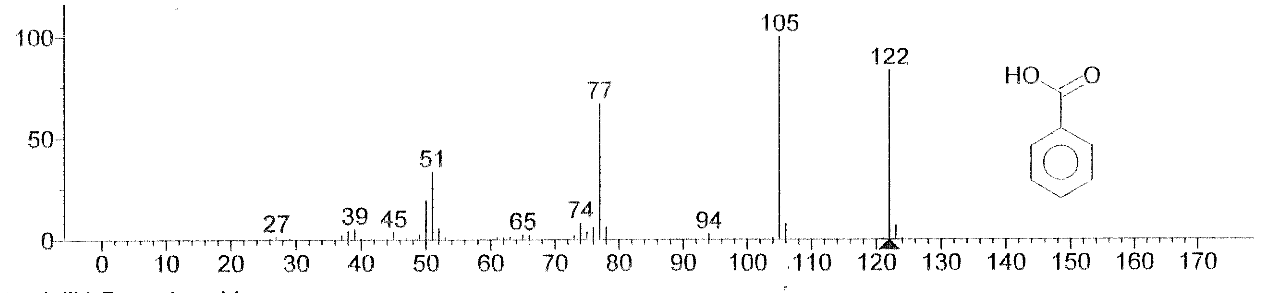

(mainlib) Benzoic acid

Name: Benzoic acid
Formula: $C_7H_6O_2$
MW: 122 CAS#: 65-85-0 NIST#: 290514 ID#: 68652 DB: mainlib
Other DBs: Fine, TSCA, RTECS, EPA, USP, HODOC, NIH, EINECS, IRDB
Contributor: NIST Mass Spectrometry Data Center, 1998.
10 largest peaks:
105 999 | 122 831 | 77 665 | 51 330 | 50 193 | 74 79 | 106 76 | 123 67 | 78 61 | 76 59 |
Synonyms:
1.Benzenecarboxylic acid
2.Benzeneformic acid
3.Benzenemethanoic acid
4.Benzoesaeure GK
5.Benzoesaeure GV
6.Carboxybenzene
7.Dracylic acid
8.Phenylcarboxylic acid
9.Phenylformic acid
10.Retarder BA
11.Retardex
12.Salvo, liquid
13.Solvo, powder
14.Tenn-Plas
15.Acide benzoique
16.Benzoic acid, tech.
17.Kyselina benzoova
18.Salvo
19.Benzoesaeure
20.Salvo powder
21.E 210
22.Ha 1
23.Ha 1 (acid)
24.Salvo liquid
25.Solvo powder
26.Phenylcarboxy
27.Benzenemethonic acid
28.Diacylic acid
29.Flowers of benjamin
30.Flowers of benzoin
31.Nipacide
32.Oracylic acid
33.Predominantly benzoic acid
34.Retarded BA
35.Retarder BA, BAX

Estimated non-polar retention index (n-alkane scale):
Value: 1150 iu
Confidence interval (Carboxylic acids): 51(50%) 220(95%) iu

Retention index.
1. Value: 1160 iu
Column Type: Capillary
Column Class: Standard non-polar
Active Phase: DB-1
Column

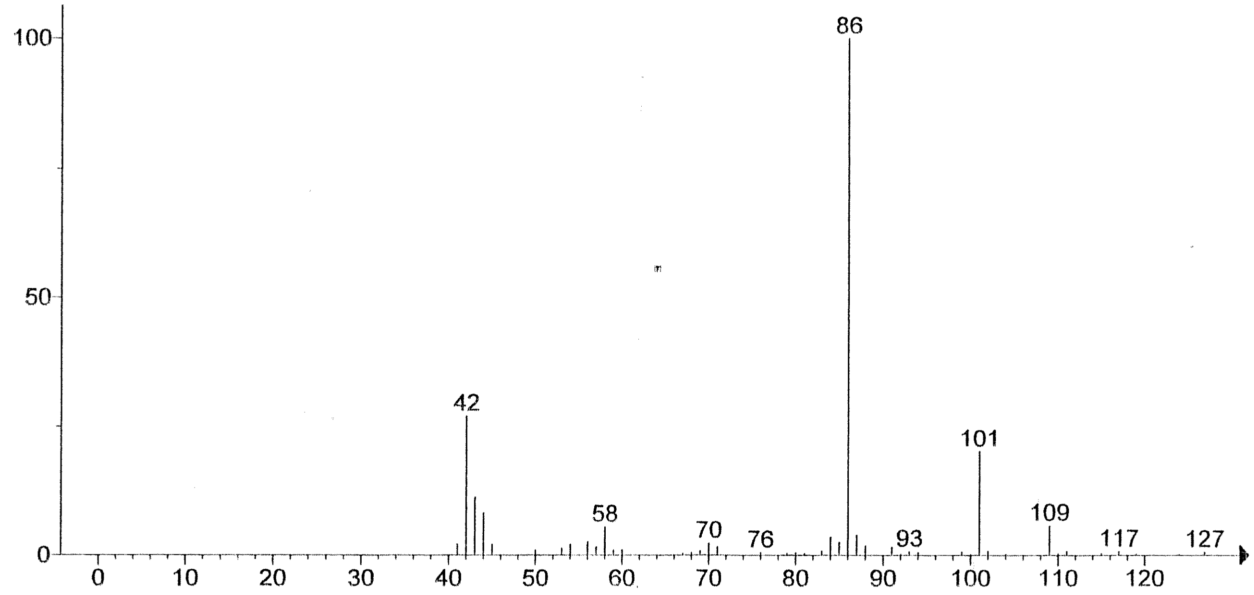

(Text File) +EI Scan (21.702 min) ALI-OLE-H7-220421-.D Subtract

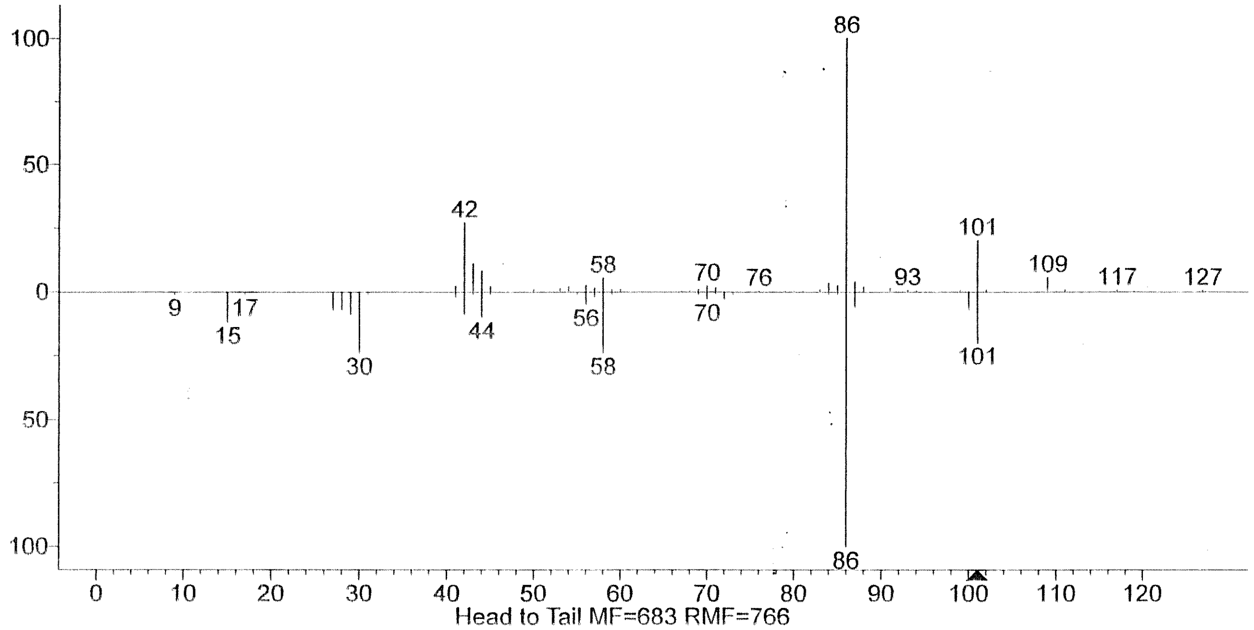

Head to Tail MF=683 RMF=766

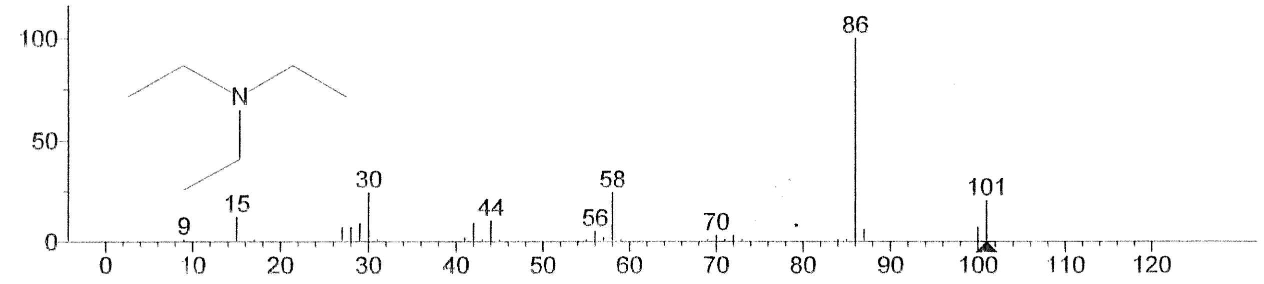

(replib) Triethylamine

Name: Triethylamine
Formula: $C_6H_{15}N$
MW: 101 CAS#: 121-44-8 NIST#: 51537 ID#: 10741 DB: replib
Other DBs: Fine, TSCA, RTECS, EPA, HODOC, EINECS, IRDB
Contributor: CARL DJERASSI DEPT OF CHEM STANFORD UNIV STANFORD CALIF 94305
10 largest peaks:
86 999 | 30 240 | 58 240 | 101 200 | 15 120 | 44 100 | 29 90 | 42 90 | 27 70 | 28 70 |
Synonyms:
1.Ethanamine, N,N-diethyl-
2.(C2H5)3N
3.(Diethylamino)ethane
4.N,N-Diethylethanamine
5.TEN
6.Triaethylamin
7.Trietilamina
8.UN 1296
9.N,N,N-Triethylamine #

Estimated non-polar retention index (n-alkane scale):
Value: 667 iu
Confidence interval (Nitrogen-containing): 83(50%) 356(95%) iu

Retention index.
1. Value: 682 iu
Column Type: Capillary
Column Class: Standard non-polar
Active Phase: DB-1
Column Length:
30 m
Carrier Gas: He
Column Diameter: 0.32 mm
Phase Thickness: 5 um
Data Type: Linear RI
Program Type:
Ramp
Start T: 35 C
End T: 270 C
Heat Rate: 10 K/min
Start Time: 1 min
Source: Bartelt, R.J., Calibration of a
commercial solid-phase microextraction device for measuring headspace concentrations of organic volatiles, Anal. Chem., 69, 1997, 364-372.

2. Value: 724 iu
Column Type: Packed
Column Class: Standard non-polar
Active
Phase: PMS-100
Column Length: 3 m
Carrier Gas: He
Substrate: Celite 545 (44-60 mesh)
Data Type: Kovats
RI
Program Type: Isothermal

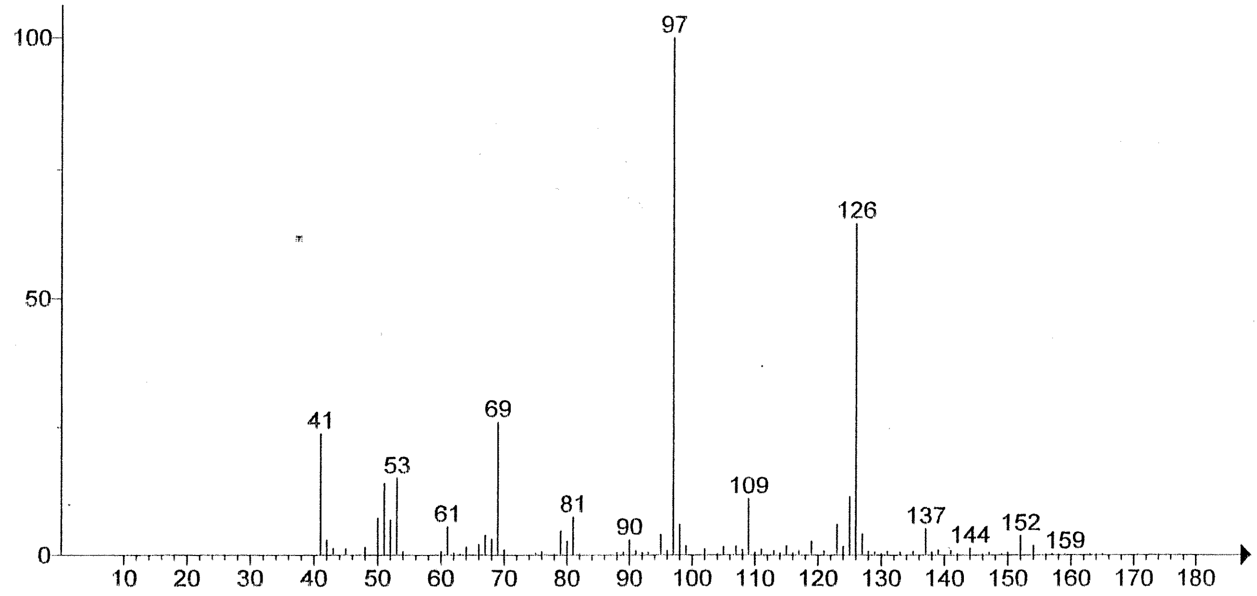

(Text File) +EI Scan (23.623 min) ALI-OLE-H7-220421-.D Subtract

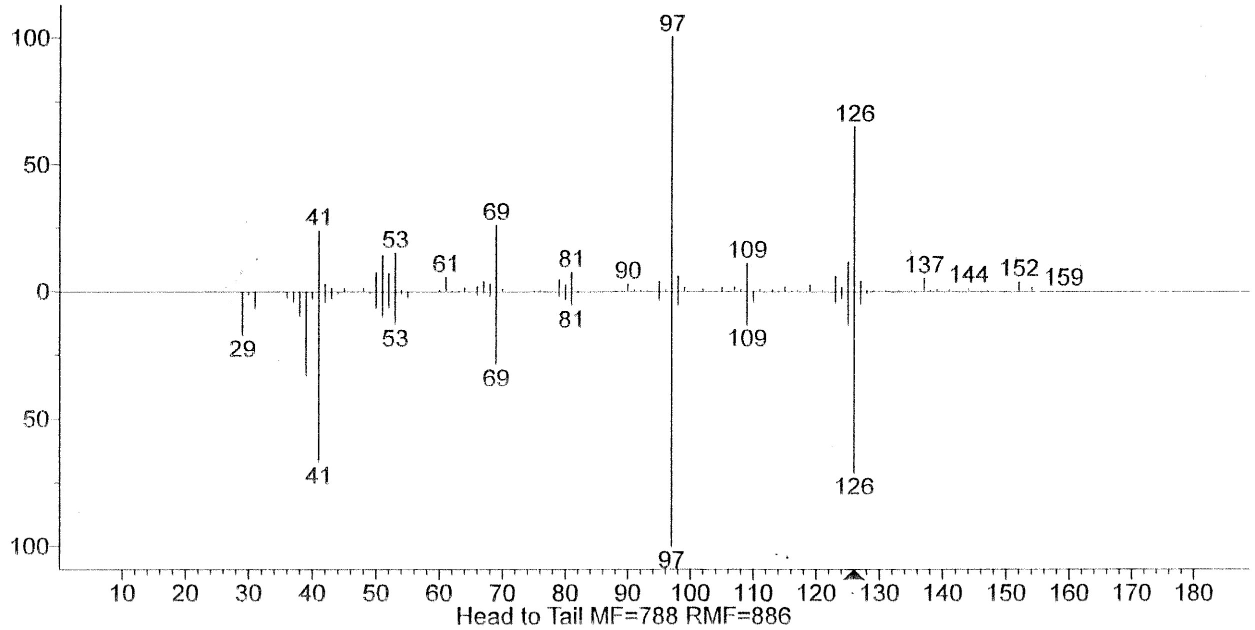

Head to Tail MF=788 RMF=886

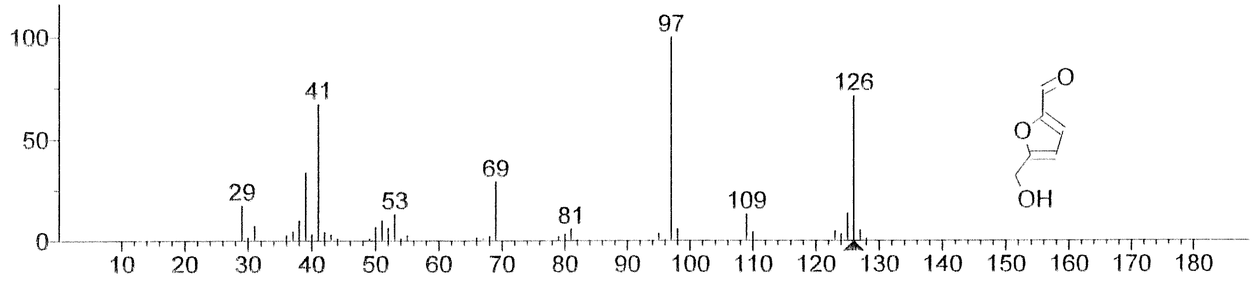

(replib) 2-Furancarboxaldehyde, 5-(hydroxymethyl)-

43

Name: 2-Furancarboxaldehyde, 5-(hydroxymethyl)-
Formula: $C_6H_6O_3$
MW: 126 CAS#: 67-47-0 NIST#: 60544 ID#: 12795 DB: replib
Other DBs: Fine, TSCA, RTECS, HODOC, NIH, EINECS
Contributor: D.HENNEBERG, MAX-PLANCK INSTITUTE, MULHEIM, WEST GERMANY
10 largest peaks:
97 999 | 126 710 | 41 668 | 39 336 | 69 289 | 29 173 | 125 136 | 53 131 | 109 131 | 38 100 |
Synonyms:
1.2-Furaldehyde, 5-(hydroxymethyl)-
2.5-Hydrxoymethylfurfural
3.Hydroxymethylfurfurole
4.HMF
5.5-(Hyddroxymethyl)Furfurole
6.5-(Hydroxymethyl)-2-formylfuran
7.5-(Hydroxymethyl)-2-furaldehyde
8.5-(Hydroxymethyl)-2-furancarbonal
9.5-(Hydroxymethyl)-2-furfural
10.5-(Hydroxymethyl)-2-furfuraldehyde
11.5-(Hydroxymethyl)furan-2-aldehyde
12.5-(Hydroxymethyl)furfural
13.5-Hydroxymethylfuraldehyde
14.5-Oxymethylfurfurole
15.5-Hydroxymethylfurfuraldehyde
16.5-Hydroxymethyl-2-furancarbaldehyde
17.Hydroxymethylfurfuraldehyde
18.5-(Hydroxymethyl)-2-furancarboxaldehyde
19.2-Hydroxymethyl-5-furfural

Estimated non-polar retention index (n-alkane scale):
Value: 1163 iu
Confidence interval (Diverse functional groups): 89(50%) 382(95%) iu

Retention index.
1. Value: 1176 iu
Column Type: Capillary
Column Class: Standard non-polar
Active Phase: DB-1
Column
Length: 60 m
Carrier Gas: N2
Column Diameter: 0.25 mm
Phase Thickness: 1 um
Data Type: Linear
RI
Program Type: Ramp
Start T: 30 C
End T: 200 C
Heat Rate: 5 K/min
End Time: 30 min
Source: Wu, C.-M.;
Wang, Z.; Wu, Q.H., Volatile compounds produced from monosodium glutamate in common food cooking, J. Agric. Food Chem., 48, 2000, 2438-2442.

2. Value: 1208 iu

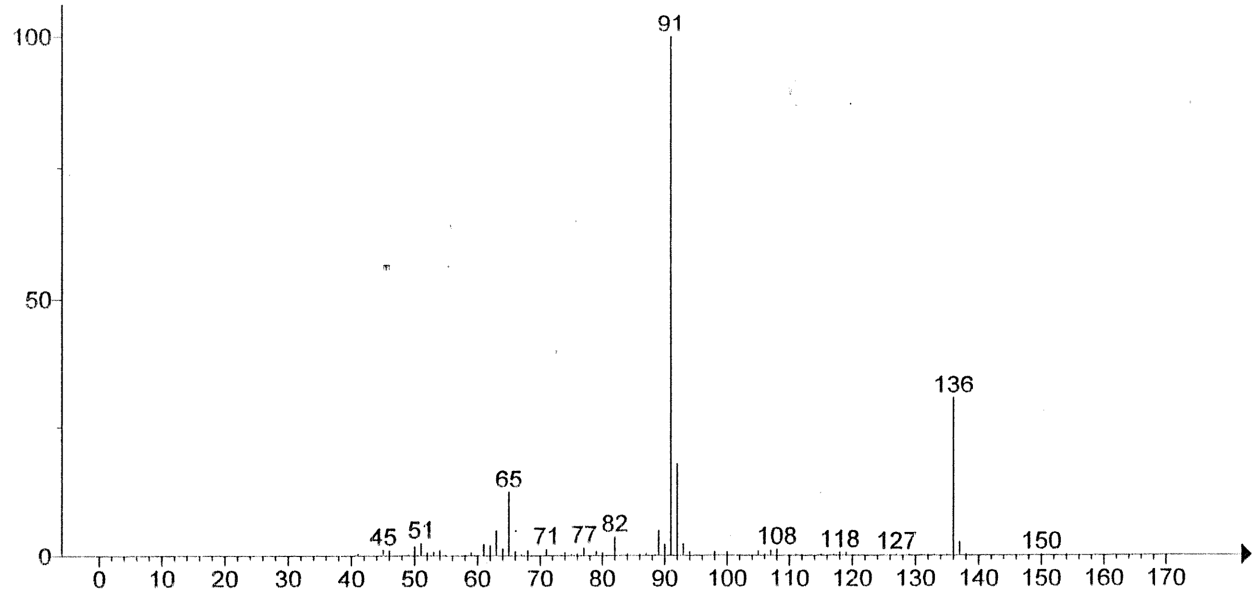

(Text File) +EI Scan (24.424 min) ALI-OLE-H7-220421-.D Subtract

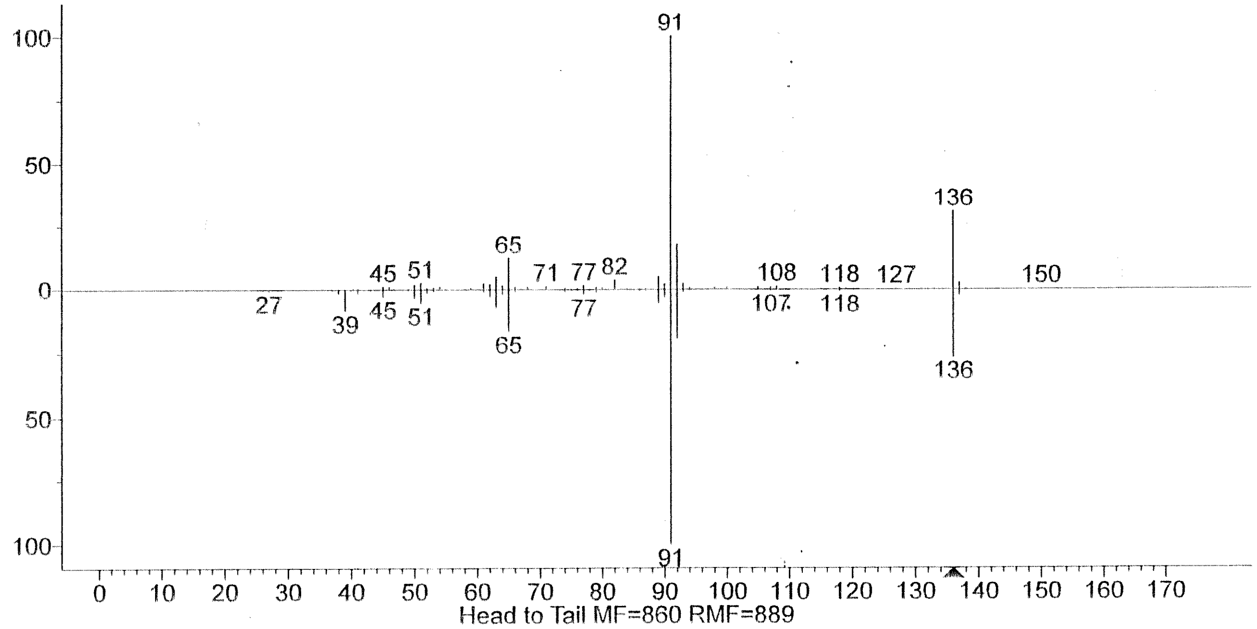

Head to Tail MF=860 RMF=889

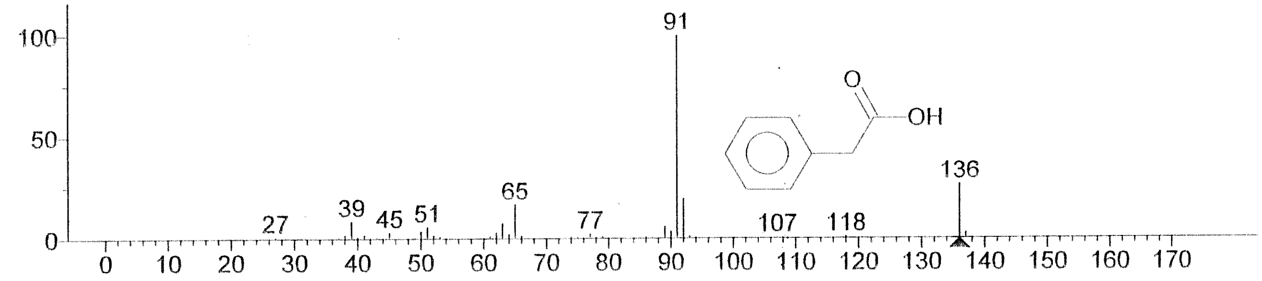

(mainlib) Benzeneacetic acid

Name: Benzeneacetic acid
Formula: $C_8H_8O_2$
MW: 136 CAS#: 103-82-2 NIST#: 352514 ID#: 53145 DB: mainlib
Other DBs: Fine, TSCA, RTECS, EPA, HODOC, NIH, EINECS, IRDB
Contributor: NIST Mass Spectrometry Data Center
10 largest peaks:
91 999 | 136 264 | 92 191 | 65 163 | 39 83 | 63 72 | 89 56 | 51 55 | 50 34 | 90 33 |
Synonyms:
1.Acetic acid, phenyl-
2.α-Toluic acid
3.Benzenacetic acid
4.Phenylacetic acid
5.ω-Phenylacetic acid
6.Phenylethanoic acid
7.Kyselina fenyloctova
8.2-Phenylacetic acid

Estimated non-polar retention index (n-alkane scale):
Value: 1249 iu
Confidence interval (Carboxylic acids): 51(50%) 220(95%) iu

Retention index.
1. Value: 1251 iu
Column Type: Capillary
Column Class: Standard non-polar
Active Phase: DB-1
Column
Length: 25 m
Column Diameter: 0.2 mm
Phase Thickness: 0.33 um
Data Type: Linear RI
Program Type:
Ramp
Start T: 50 C
End T: 300 C
Heat Rate: 4 K/min
Source: Osorio, C.; Alarcon, M.; Moreno, C.; Bonilla, A.;
Barrios, J.; Garzon, C.; Duque, C., Characterization of Odor-Active Volatiles in Champa (Campomanesia lineatifolia R. & P.), J. Agric. Food Chem., 54, 2006, 509-516.

2. Value: 1240 iu
Column Type: Packed
Column Class:
Standard non-polar
Active Phase: SE-30
Column Length: 3.05 m
Carrier Gas: He
Substrate: Supelcoport and
Chromosorb
Data Type: Linear RI
Program Type: Ramp
Start T: 40 C
End T: 250 C
Heat Rate: 10 K/min

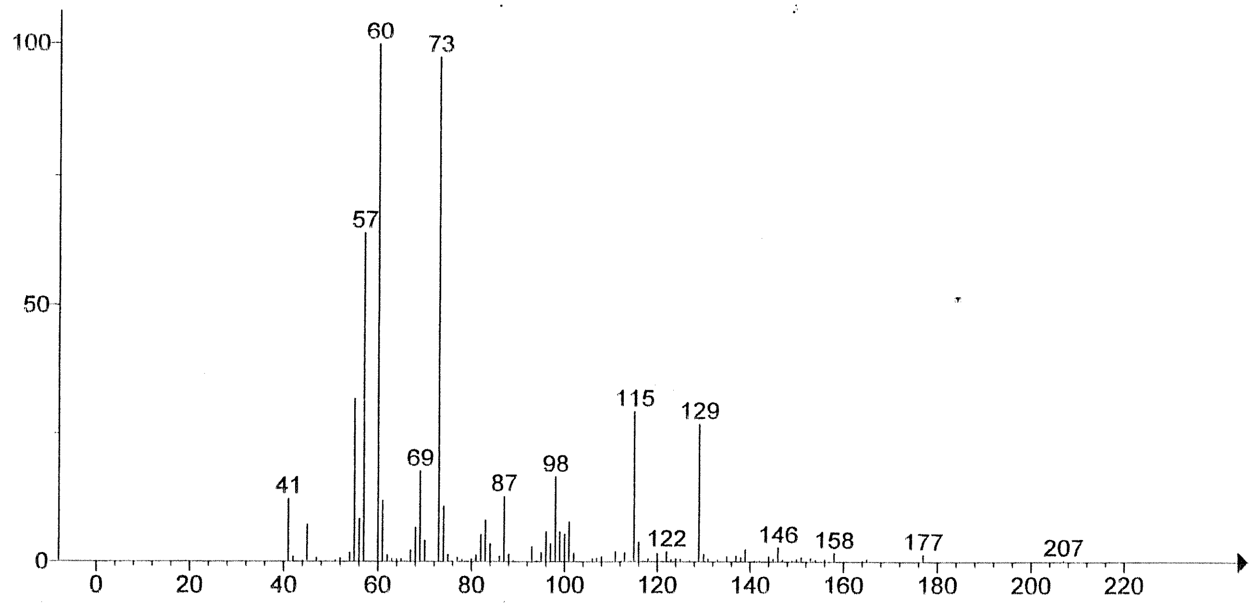

(Text File) +EI Scan (25.027 min) ALI-OLE-H7-220421-.D Subtract

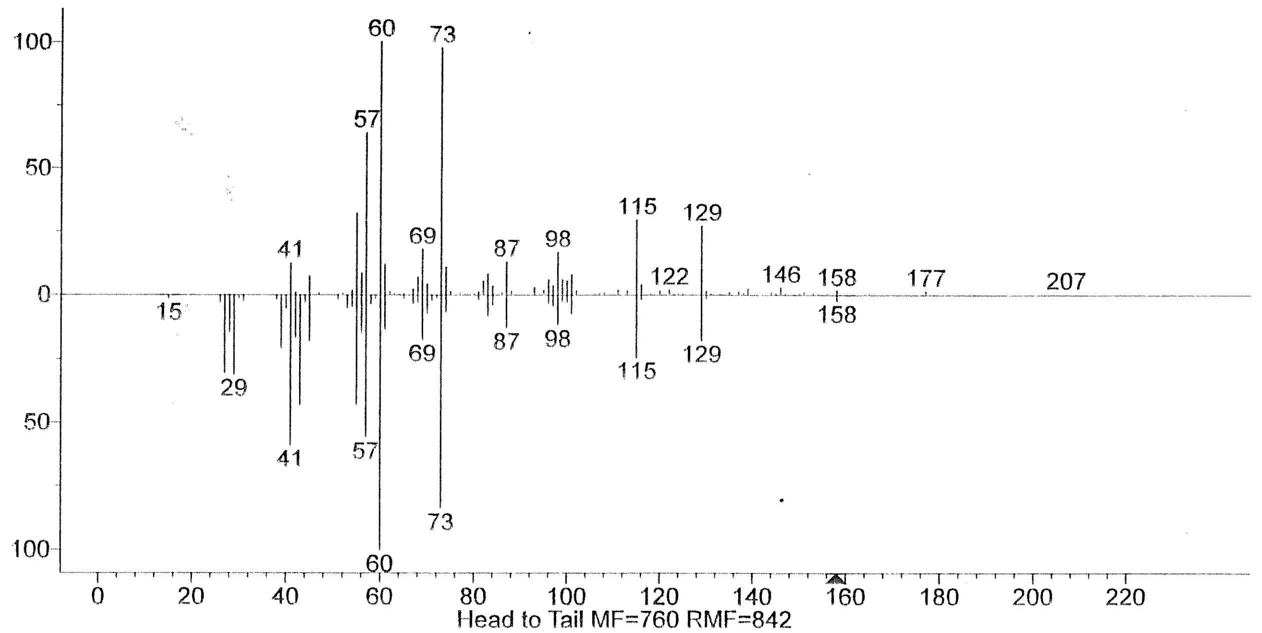

Head to Tail MF=760 RMF=842

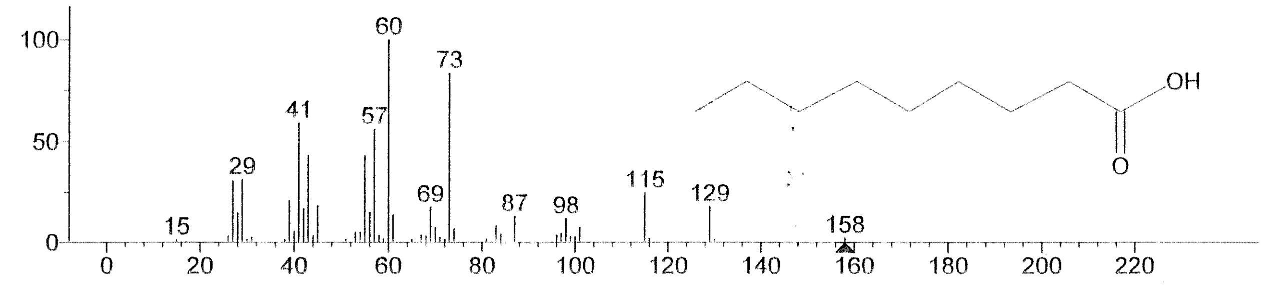

(replib) Nonanoic acid

Name: Nonanoic acid
Formula: $C_9H_{18}O_2$
MW: 158 CAS#: 112-05-0 NIST#: 114735 ID#: 6709 DB: replib
Other DBs: Fine, TSCA, RTECS, EPA, HODOC, NIH, EINECS, IRDB
Contributor: NIST Mass Spectrometry Data Center, 1990.
10 largest peaks:
60 999 | 73 836 | 41 590 | 57 559 | 43 433 | 55 431 | 29 311 | 27 304 | 115 244 | 39 207 |
Synonyms:
1.n-Nonanoic acid
2.n-Nonoic acid
3.n-Nonylic acid
4.Nonoic acid
5.Nonylic acid
6.Pelargic acid
7.Pelargonic acid
8.1-Octanecarboxylic acid
9.Cirrasol 185a
10.Emfac 1202
11.Hexacid C-9
12.Pelargon
13.Emery's L-114
14.Emery 1202

Estimated non-polar retention index (n-alkane scale):
Value: 1272 iu
Confidence interval (Carboxylic acids): 51(50%) 220(95%) iu

Retention index.
1. Value: 1268 iu
Column Type: Capillary
Column Class: Standard non-polar
Active Phase: DB-1
Column
Length: 25 m
Column Diameter: 0.2 mm
Phase Thickness: 0.33 um
Data Type: Linear RI
Program Type:
Ramp
Start T: 50 C
End T: 300 C
Heat Rate: 4 K/min
Source: Osorio, C.; Alarcon, M.; Moreno, C.; Bonilla, A.;
Barrios, J.; Garzon, C.; Duque, C., Characterization of Odor-Active Volatiles in Champa (Campomanesia lineatifolia R. & P.), J. Agric. Food Chem., 54, 2006, 509-516.

2. Value: 1263 iu
Column Type: Capillary
Column Class:
Standard non-polar
Active Phase: CP Sil 5 CB
Column Length: 25 m
Carrier Gas: H2
Column Diameter: 0.25

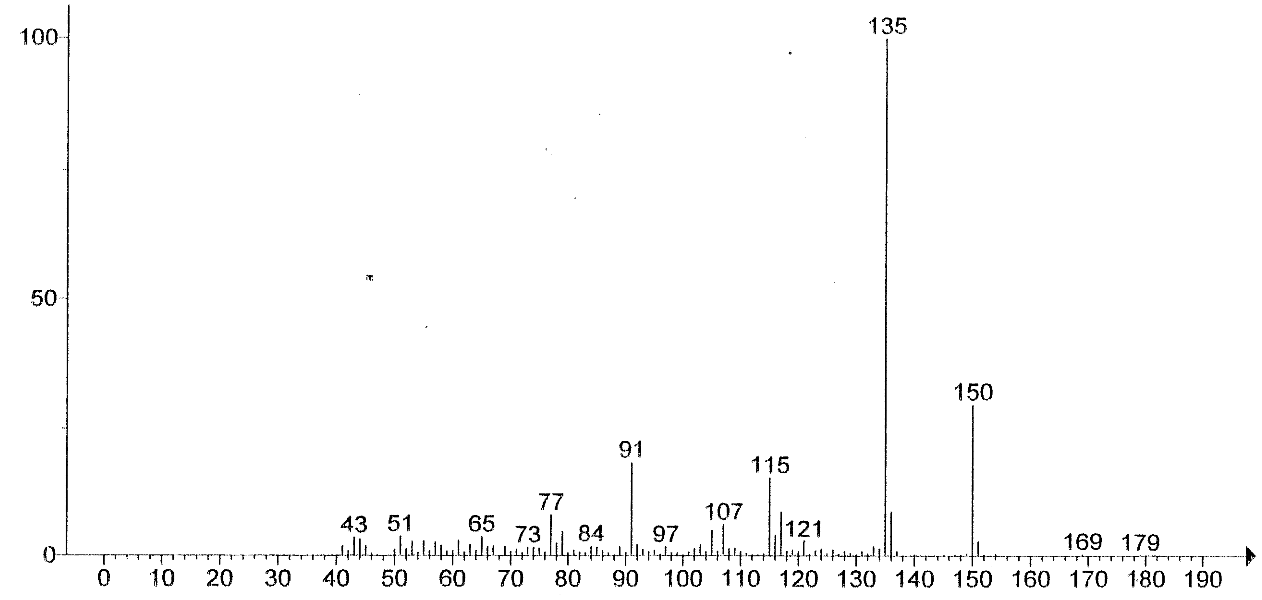

(Text File) +EI Scan (25.985 min) ALI-OLE-H7-220421-.D

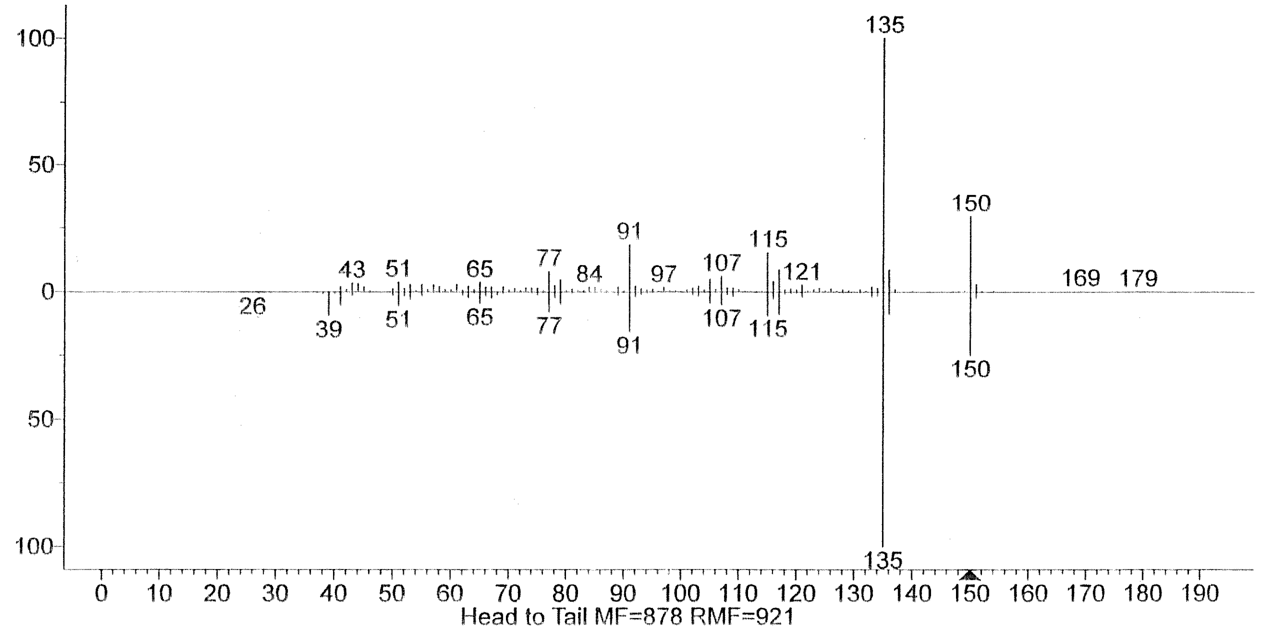

Head to Tail MF=878 RMF=921

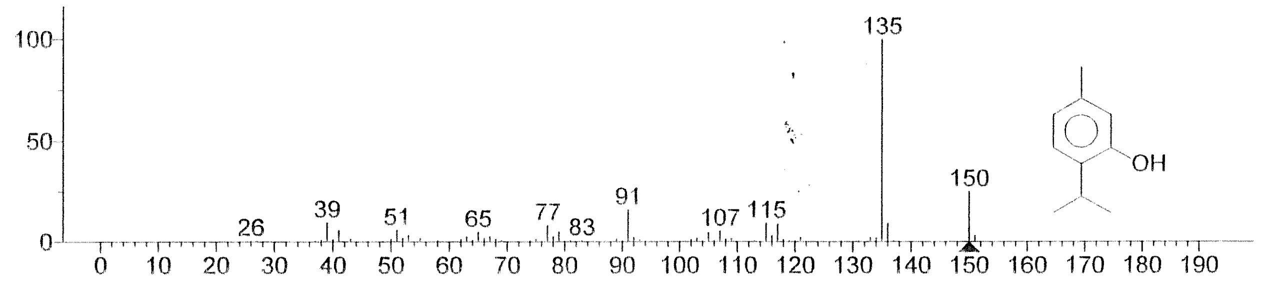

(mainlib) Thymol

Name: Thymol
Formula: $C_{10}H_{14}O$
MW: 150 CAS#: 89-83-8 NIST#: 249386 ID#: 97695 DB: mainlib
Other DBs: Fine, TSCA, RTECS, USP, HODOC, NIH, EINECS
Contributor: TNO Volatile Compounds in Food - Chemical Concepts
10 largest peaks:
135 999 | 150 248 | 91 157 | 39 94 | 115 91 | 136 89 | 117 87 | 77 80 | 51 56 | 41 55 |
Synonyms:
1.Phenol, 5-methyl-2-(1-methylethyl)-
2.p-Cymen-3-ol
3.Thyme camphor
4.2-Isopropyl-5-methylphenol
5.3-Hydroxy-p-cymene
6.3-Methyl-6-isopropylphenol
7.5-Methyl-2-isopropylphenol
8.6-Isopropyl-m-cresol
9.6-Isopropyl-3-methylphenol
10.m-Cresol, 6-isopropyl-
11.p-Cymene, 3-hydroxy-
12.Isopropyl cresol
13.Phenol, 2-isopropyl-5-methyl-
14.Thymic acid
15.1-Hydroxy-5-methyl-2-isopropylbenzene
16.1-Methyl-3-hydroxy-4-isopropylbenzene
17.3-p-Cymenol
18.3-Hydroxy-1-methyl-4-isopropylbenzene
19.5-Methyl-2-isopropyl-1-phenol
20.5-Methyl-2-(1-methylethyl)phenol
21.Isopropyl-m-cresol
22.m-Thymol

Estimated non-polar retention index (n-alkane scale):
Value: 1262 iu
Confidence interval (Phenols): 70(50%) 301(95%) iu

Retention index.
1. Value: 1266 iu
Column Type: Capillary
Column Class: Standard non-polar
Active Phase: RTX-1
Column
Length: 60 m
Carrier Gas: He
Column Diameter: 0.22 mm
Phase Thickness: 0.25 um
Data Type: Linear
RI
Program Type: Ramp
Start T: 60 C
End T: 230 C
Heat Rate: 2 K/min
End Time: 30 min
Source: Bendahou,
M.; Muselli, A.; Grignon-Dubois, M.; Benyoucef, M.; Desjobert, J.-M.; Bernardini, A.-F.; Costa, J., Antimicrobial

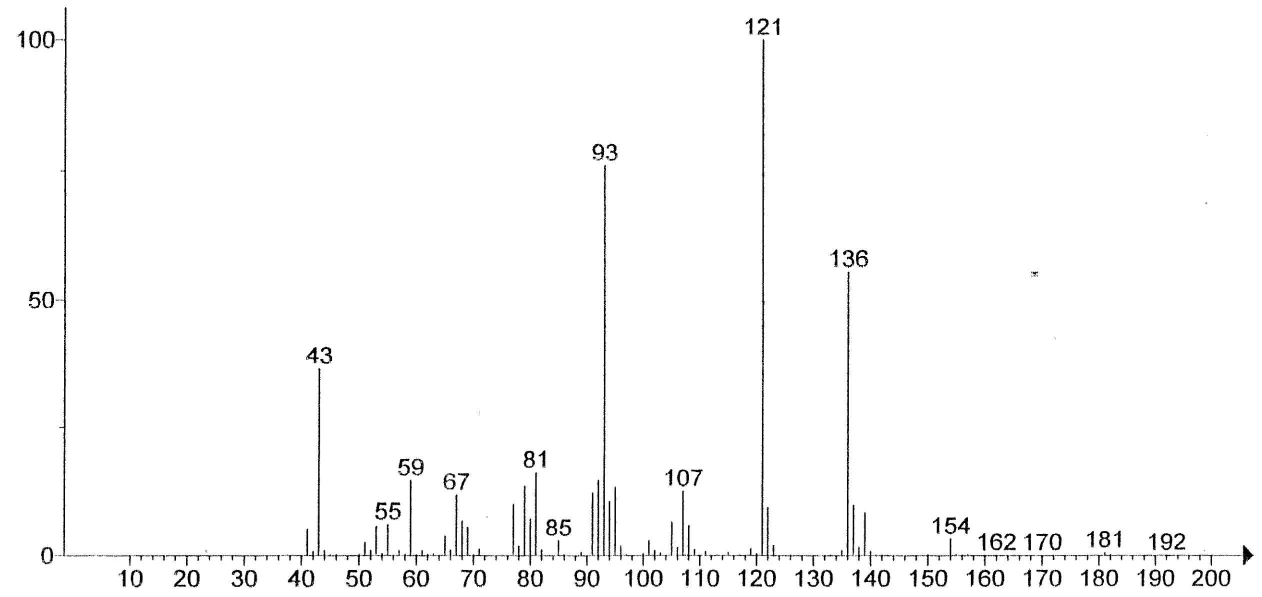

(Text File) +EI Scan (28.102 min) ALI-OLE-H7-220421-.D Subtract

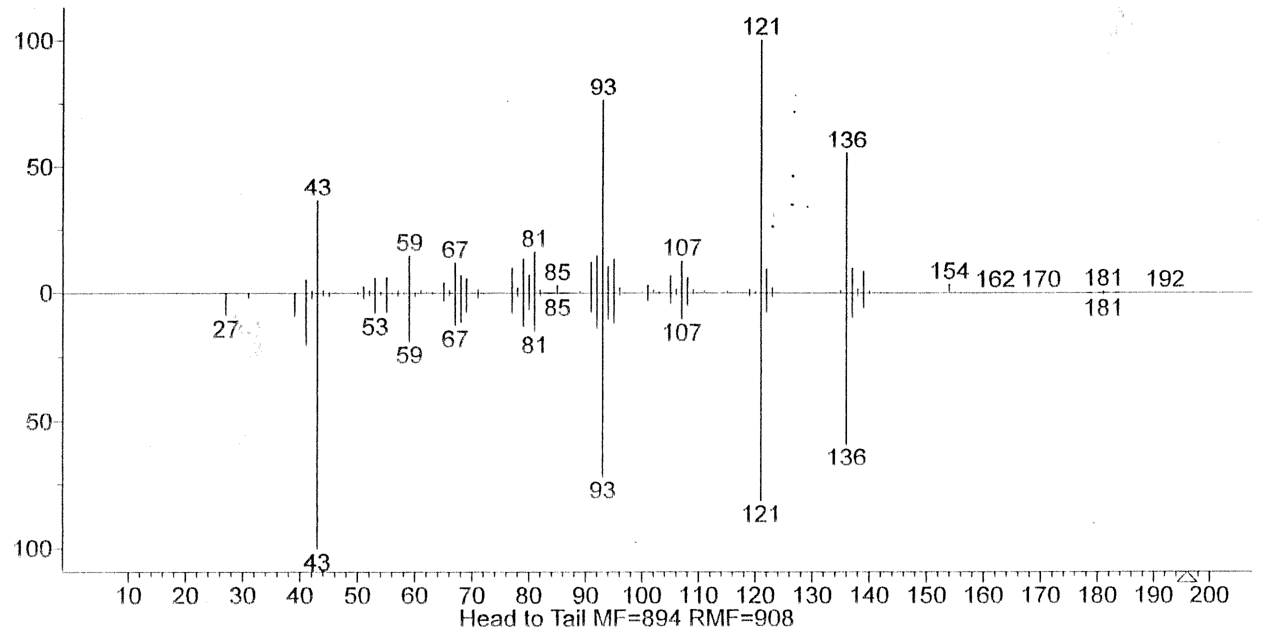

Head to Tail MF=894 RMF=908

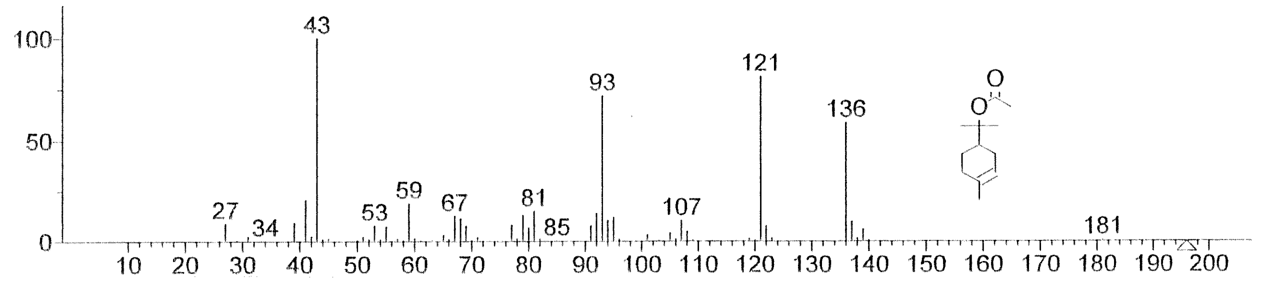

(replib) 3-Cyclohexene-1-methanol, α,α,4-trimethyl-, acetate

Name: 3-Cyclohexene-1-methanol, α,α,4-trimethyl-, acetate
Formula: $C_{12}H_{20}O_2$
MW: 196 CAS#: 80-26-2 NIST#: 249543 ID#: 2926 DB: replib
Other DBs: Fine, TSCA, RTECS, HODOC, EINECS
Contributor: TNO Volatile Compounds in Food - Chemical Concepts
10 largest peaks:
43 999 | 121 812 | 93 718 | 136 586 | 41 205 | 59 188 | 81 150 | 92 138 | 79 129 | 67 126 |
Synonyms:
1.p-Menth-1-en-8-ol, acetate
2.α-Terpineol acetate
3.α-Terpinyl acetate
4.Terpinyl acetate
5.1-Methyl-1-(4-methyl-3-cyclohexen-1-yl)ethyl acetate #

Estimated non-polar retention index (n-alkane scale):
Value: 1333 iu
Confidence interval (Esters): 47(50%) 201(95%) iu

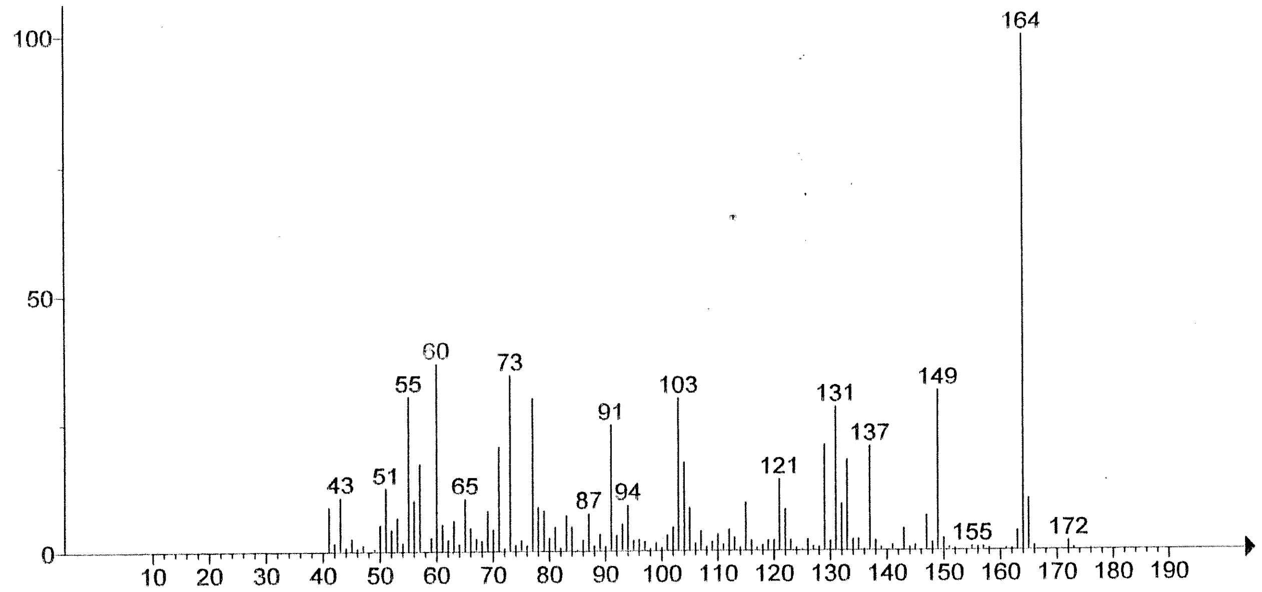

(Text File) +EI Scan (28.366 min) ALI-OLE-H7-220421-.D Subtract

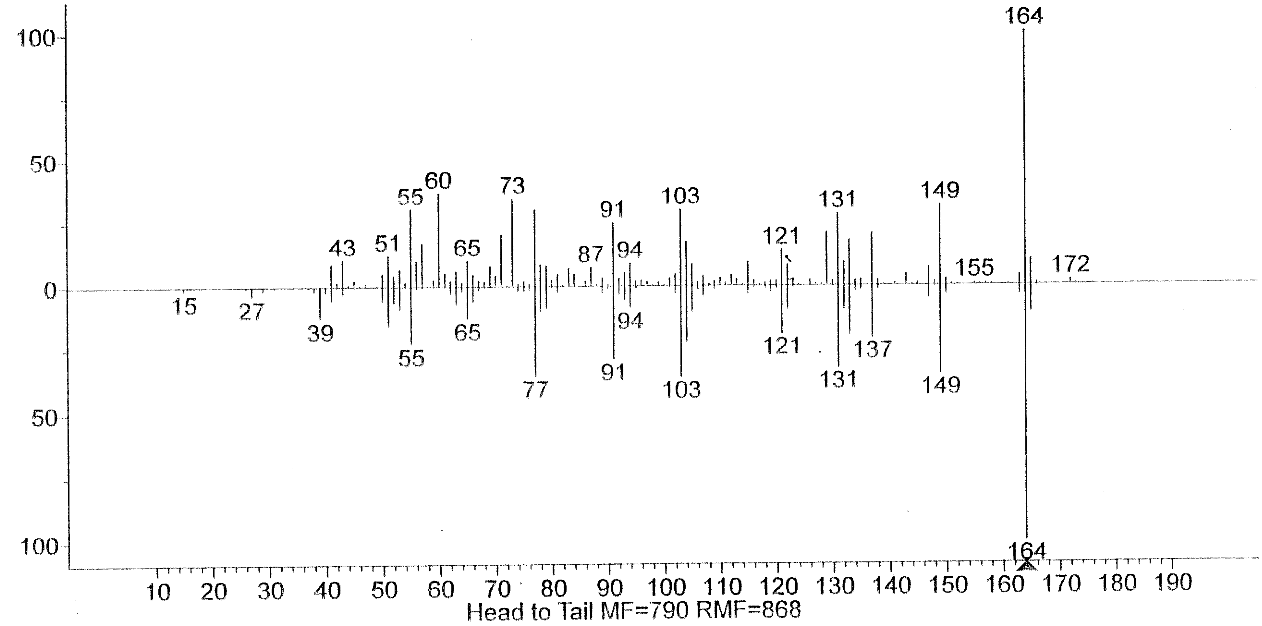

Head to Tail MF=790 RMF=868

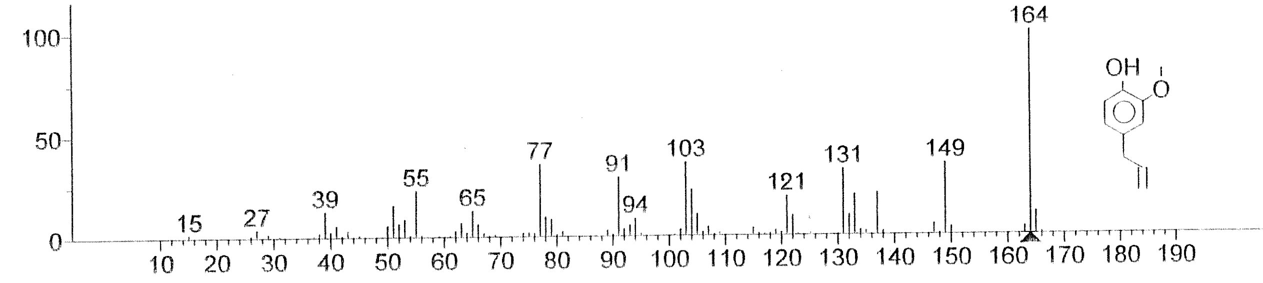

(mainlib) Eugenol

Name: Eugenol
Formula: $C_{10}H_{12}O_2$
MW: 164 CAS#: 97-53-0 NIST#: 291519 ID#: 121999 DB: mainlib
Other DBs: Fine, TSCA, RTECS, EPA, USP, HODOC, NIH, EINECS, IRDB
Contributor: NIST Mass Spectrometry Data Center, 1998.
10 largest peaks:
164 999 | 103 360 | 77 355 | 149 350 | 131 323 | 91 288 | 55 227 | 104 225 | 137 207 | 133 197 |
Synonyms:
1.Phenol, 2-methoxy-4-(2-propenyl)-
2.Phenol, 4-allyl-2-methoxy-
3.p-Allylguaiacol
4.p-Eugenol
5.Caryophyllic acid
6.Engenol
7.Eugenic acid
8.2-Methoxy-1-hydroxy-4-allylbenzene
9.2-Methoxy-4-allylphenol
10.4-Allyl-2-methoxyphenol
11.4-Allylguaiacol
12.4-Hydroxy-3-methoxyallylbenzene
13.NCI-C50453
14.1-Hydroxy-2-methoxy-4-allylbenzene
15.1-Hydroxy-2-methoxy-4-prop-2-enylbenzene
16.2-Methoxy-4-(2-propenyl)phenol
17.2-Methoxy-4-prop-2-enylphenol
18.4-Allyl-1-hydroxy-2-methoxybenzene
19.4-Allylcatechol-2-methyl ether
20.1,3,4-Eugenol
21.FA 100
22.Fema no. 2467
23.2-Metoksy-4-allilofenol
24.2-Hydroxy-5-allylanisole
25.2-Methoxy-4-(2-propenyl)phenyl
26.Allylguaiacol

Estimated non-polar retention index (n-alkane scale):
Value: 1392 iu
Confidence interval (Diverse functional groups): 89(50%) 382(95%) iu

Retention index.
1. Value: 1337 iu
Column Type: Capillary
Column Class: Standard non-polar
Active Phase: BP-1
Column
Length: 50 m
Carrier Gas: He
Column Diameter: 0.22 mm
Phase Thickness: 0.25 um
Data Type: Linear
RI
Program Type: Ramp
Start T: 60 C
End T: 220 C

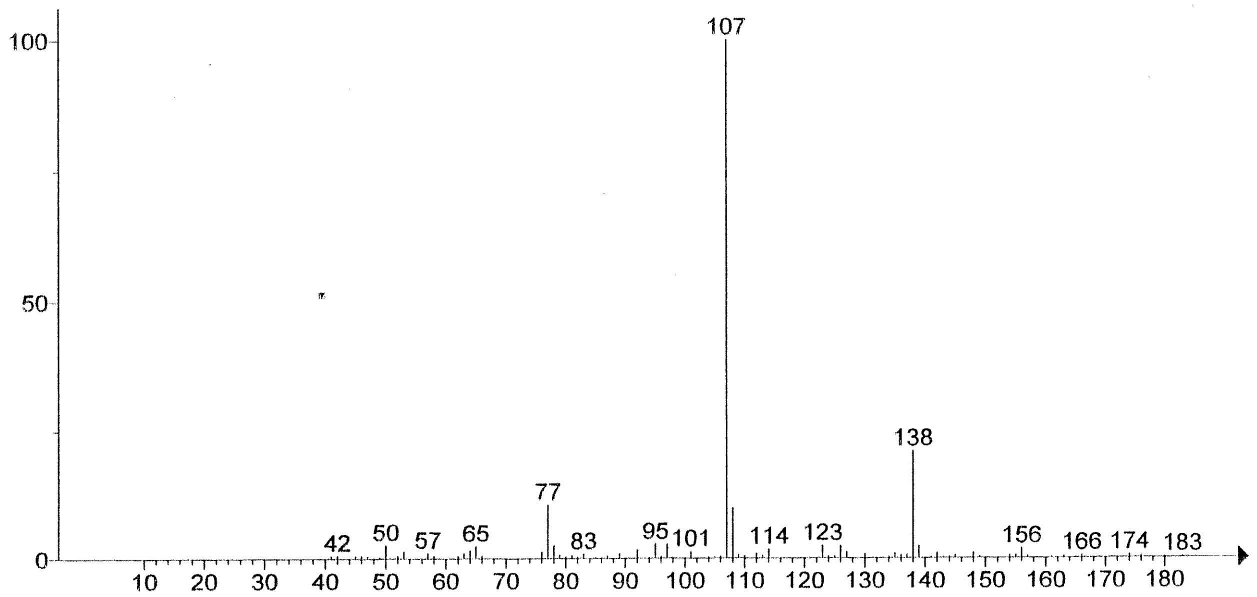

(Text File) +EI Scan (30.607 min) ALI-OLE-H7-220421-.D Subtract

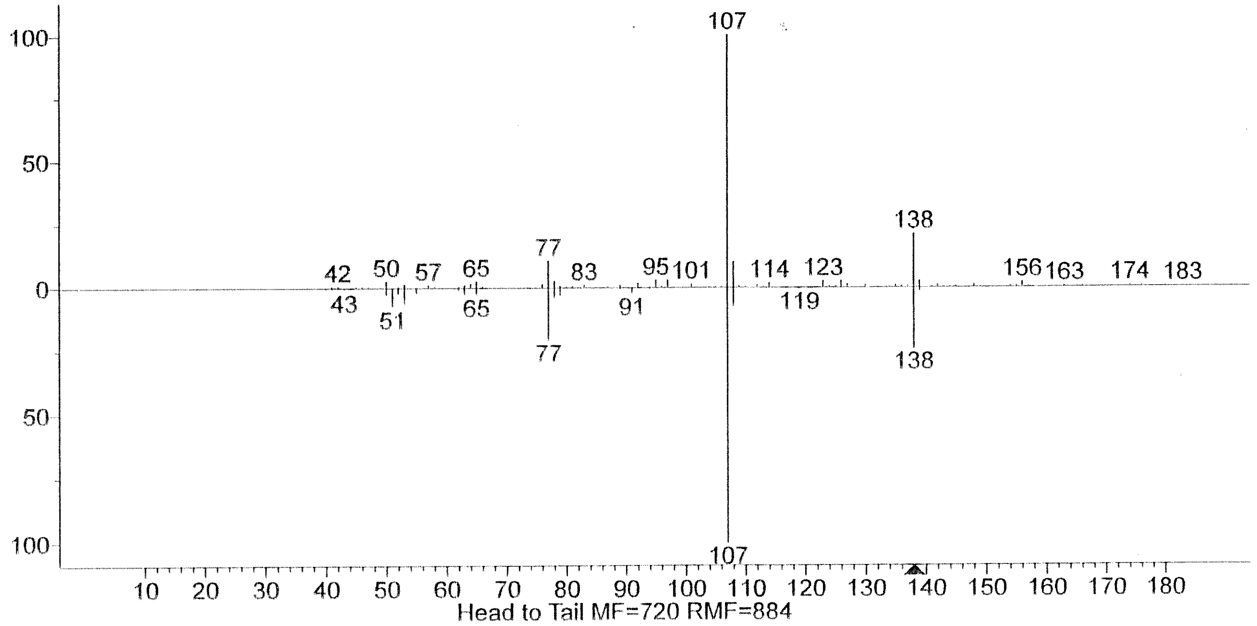

Head to Tail MF=720 RMF=884

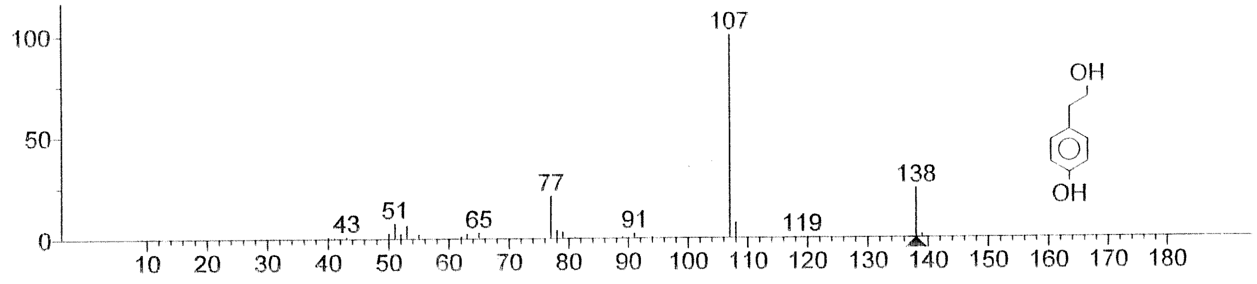

(replib) Benzeneethanol, 4-hydroxy-

Name: Benzeneethanol, 4-hydroxy-
Formula: $C_8H_{10}O_2$
MW: 138 CAS#: 501-94-0 NIST#: 92403 ID#: 14558 DB: replib
Other DBs: Fine, TSCA, HODOC, NIH, EINECS
Contributor: J.E. WILKINSON S-CUBED, SAN DIEGO, CA.
10 largest peaks:
107 999 | 138 239 | 77 205 | 108 74 | 51 73 | 53 62 | 78 39 | 79 31 | 65 27 | 50 24 |
Synonyms:
1.4-(2-Hydroxyethyl)phenol
2.p-Hydroxyphenethyl alcohol
3.4-Hydroxyphenethyl alcohol
4.β-(p-Hydroxyphenyl)ethanol
5.β-(4-Hydroxyphenyl)ethanol
6.2-(p-Hydroxyphenyl)ethanol
7.2-(4-Hydroxyphenyl)ethanol
8.4-Hydroxyphenylethanol
9.Phenethyl alcohol, p-hydroxy-
10.p-Thyrosol
11.Tyrosol
12.p-Tyrosol
13.4-Hydroxyphenylethyl alcohol
14.p-Hydroxyphenylethyl alcohol

Estimated non-polar retention index (n-alkane scale):
Value: 1356 iu
Confidence interval (Diverse functional groups): 89(50%) 382(95%) iu

Retention index.
1. Value: 1451 iu
Column Type: Capillary
Column Class: Semi-standard non-polar
Active Phase: HP
-5MS
Column Length: 30 m
Carrier Gas: He
Column Diameter: 0.25 mm
Phase Thickness: 0.25 um
Data Type:
Normal alkane RI
Program Type: Complex
Description: 40C(3min)=> 2C/min=>180C=>10C/min=>250C(5min)

Source: Alissandrakis, E.; Kibaris, A.C.; Tarantilis, P.A.; Harizanis, P.C.; Polissiou, M., Flavour compounds of Greek cotton honey, J. Sci. Food Agric., 85, 2005, 1444-1452.

2. Value: 1478 iu
Column Type:
Capillary
Column Class: Semi-standard non-polar
Active Phase: SPB-5
Column Length: 30 m
Carrier Gas:
He
Column Diameter: 0.53 mm

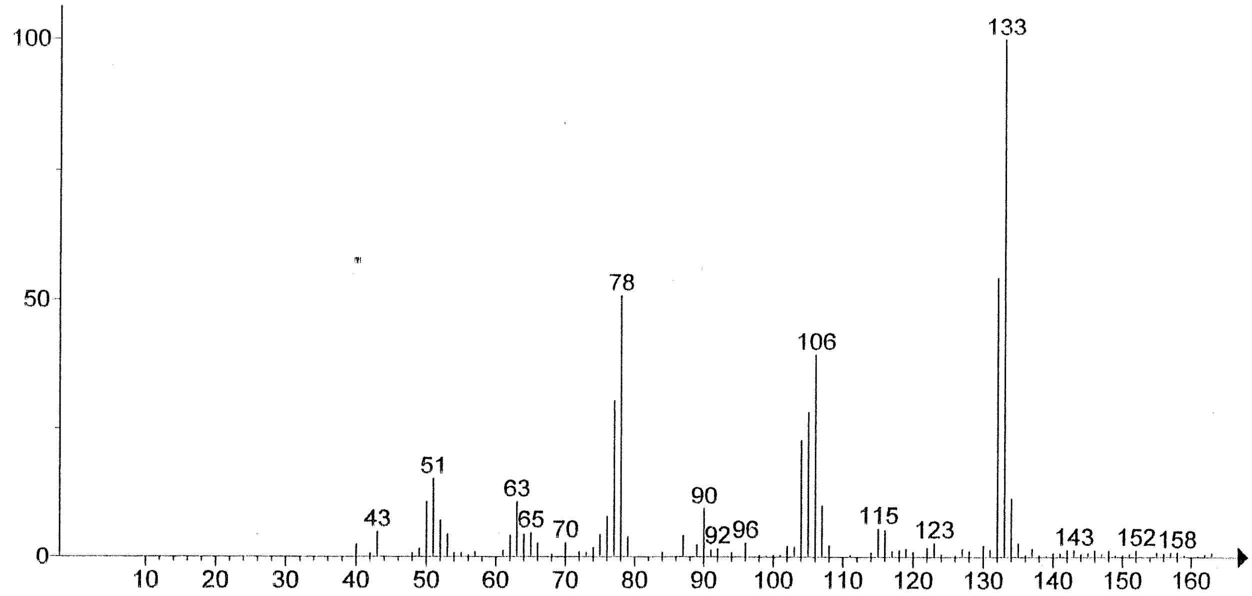

(Text File) +EI Scan (32.172 min) ALI-OLE-H7-220421-.D Subtract

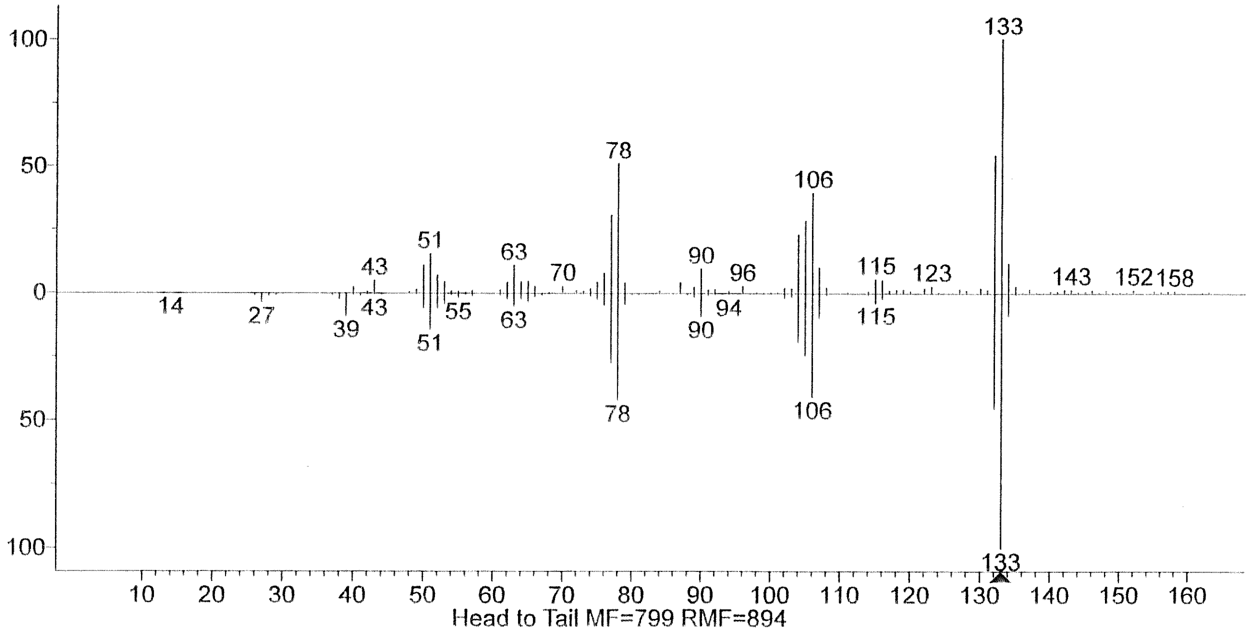

Head to Tail MF=799 RMF=894

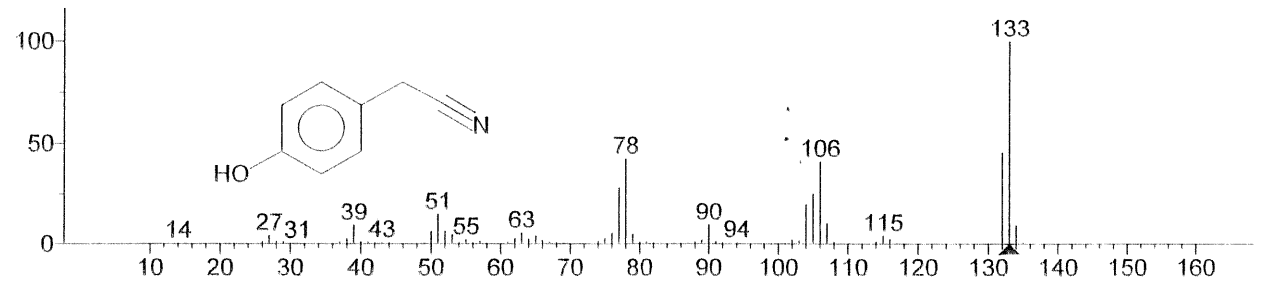

(mainlib) Benzeneacetonitrile, 4-hydroxy-

Name: Benzeneacetonitrile, 4-hydroxy-
Formula: C_8H_7NO
MW: 133 CAS#: 14191-95-8 NIST#: 236771 ID#: 94894 DB: mainlib
Other DBs: Fine, RTECS, HODOC, NIH, EINECS
Contributor: Japan AIST/NIMC Database- Spectrum MS-NW-8839
10 largest peaks:
133 999 | 132 454 | 78 421 | 106 407 | 77 277 | 105 247 | 104 195 | 51 145 | 107 100 | 90 92 |
Synonyms:
1.Acetonitrile, (p-hydroxyphenyl)-
2.(4-Hydroxyphenyl)acetonitrile
3.p-Hydroxybenzyl cyanide
4.p-Hydroxyphenylacetonitrile
5.4-Hydroxybenzyl cyanide

Estimated non-polar retention index (n-alkane scale):
Value: 1359 iu
Confidence interval (Diverse functional groups): 89(50%) 382(95%) iu

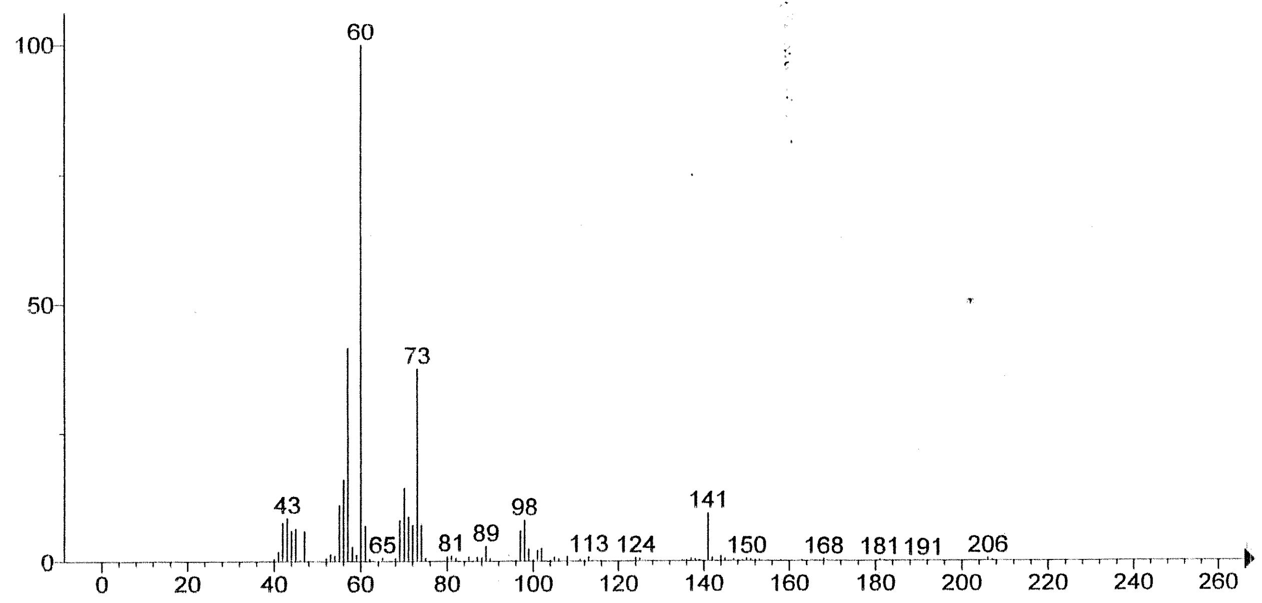

(Text File) +EI Scan (32.956 min) ALI-OLE-H7-220421-.D Subtract

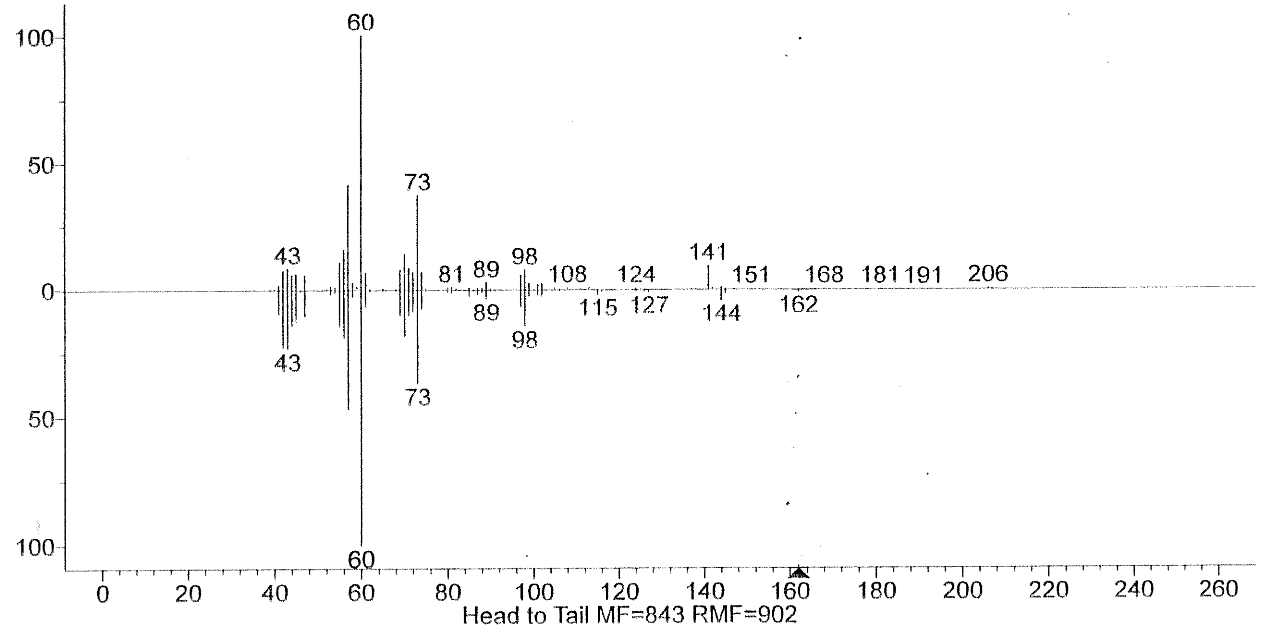

Head to Tail MF=843 RMF=902

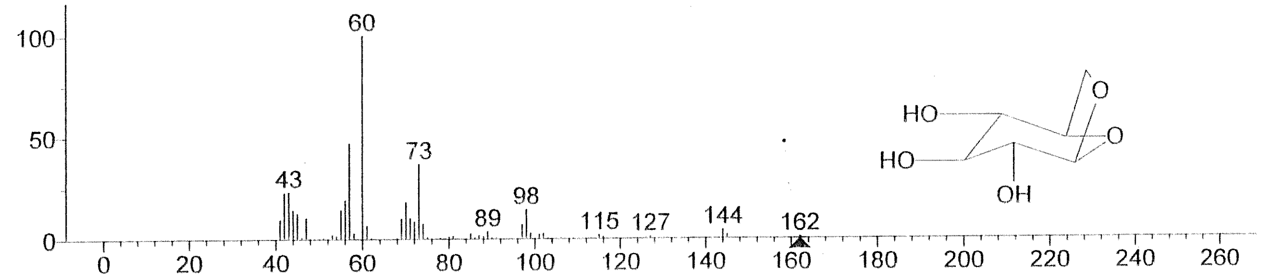

(replib) β-D-Glucopyranose, 1,6-anhydro-

Name: β-D-Glucopyranose, 1,6-anhydro-
Formula: $C_6H_{10}O_5$
MW: 162 CAS#: 498-07-7 NIST#: 127044 ID#: 6692 DB: replib
Other DBs: NIH, EINECS
Contributor: LAC, NIDDK, NIH, Bethesda, MD 20892
10 largest peaks:
60 999 | 57 468 | 73 366 | 43 230 | 42 225 | 56 190 | 70 181 | 55 143 | 98 143 | 44 141 |
Synonyms:
1.Anhydro-d-mannosan
2.Levoglucosan
3.1,6-Anhydro-β-D-glucopyranose

Estimated non-polar retention index (n-alkane scale):
Value: 1404 iu
Confidence interval (Diverse functional groups): 89(50%) 382(95%) iu

Retention index.
1. Value: 1426.2 iu
Column Type: Capillary
Column Class: Standard non-polar
Active Phase: DB-1
Column
Length: 60 m
Carrier Gas: He
Column Diameter: 0.25 mm
Data Type: Normal alkane RI
Program Type:
Ramp
Start T: 50 C
End T: 275 C
Heat Rate: 4 K/min
Start Time: 2 min
Source: Ralph, J.; Hatfield, R.D.,
Pyrolisys-GC-MS Characterization of Forage Materials, J. Agric. Food Chem., 39, 1991, 1426-1437.

2. Value:
1491.1 iu
Column Type: Capillary
Column Class: Semi-standard non-polar
Active Phase: LM-5
Column Length:
30 m
Carrier Gas: He
Column Diameter: 0.25 mm
Phase Thickness: 0.25 um
Data Type: Linear RI
Program
Type: Complex
Description: 60C(2min) =>15C/min =>180C =>5C/min =>280C (10min)
Source: Ré-Poppi, N.;
Santiago-Silva, M., Polycyclic aromatic hydrocarbons and other selected organic compounds in ambient air of Campo Grande City, Brazil, Atmos. Environ., 39, 2005, 2839-2850.

<...>

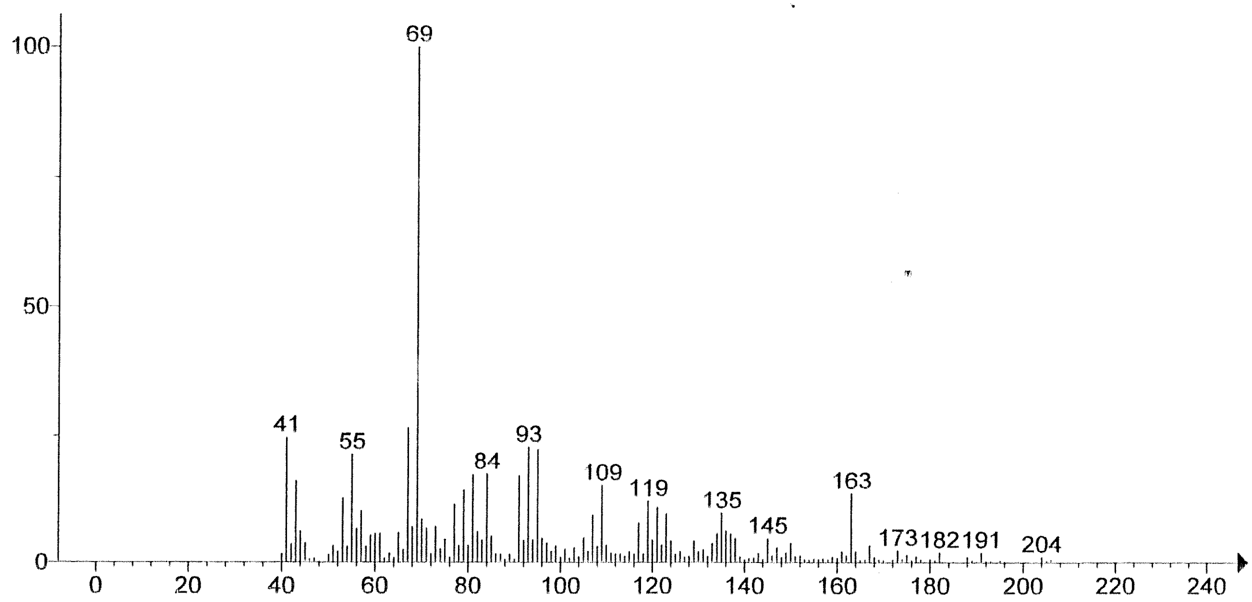

(Text File) +EI Scan (33.779 min) ALI-OLE-H7-220421-.D

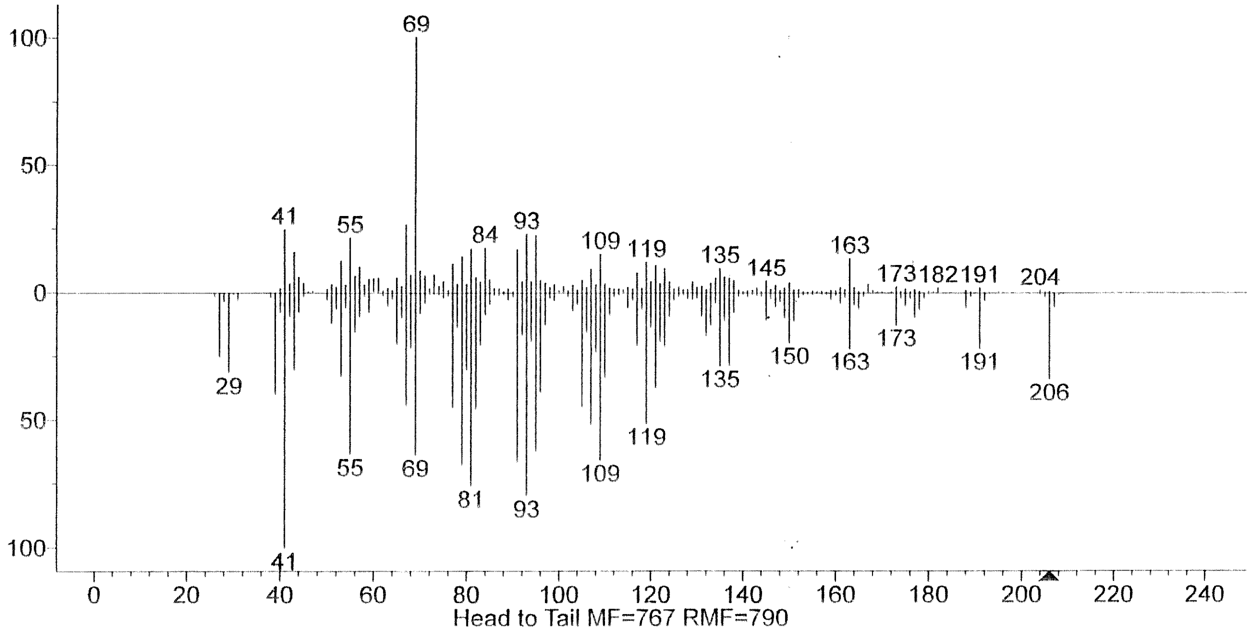

Head to Tail MF=767 RMF=790

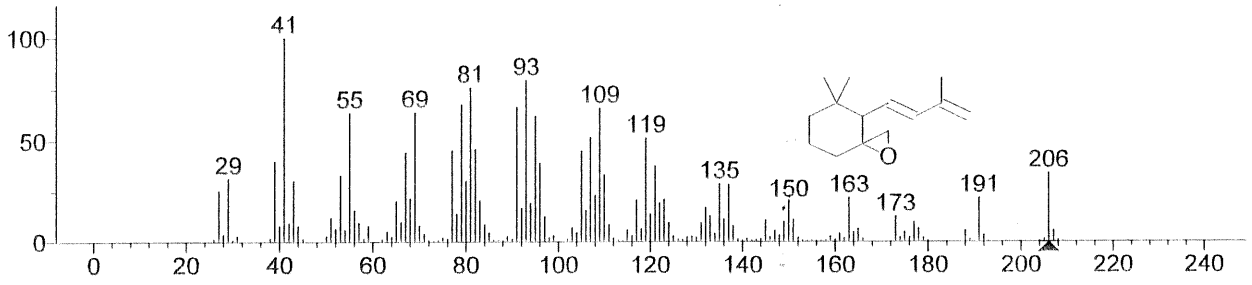

(mainlib) 1-Oxaspiro[2.5]octane, 5,5-dimethyl-4-(3-methyl-1,3-butadienyl)-

Name: 1-Oxaspiro[2.5]octane, 5,5-dimethyl-4-(3-methyl-1,3-butadienyl)-
Formula: $C_{14}H_{22}O$
MW: 206 NIST#: 195921 ID#: 3527 DB: mainlib
Contributor: Chemical Concepts
10 largest peaks:
41 999 | 93 794 | 81 757 | 79 675 | 91 664 | 109 657 | 69 637 | 55 633 | 95 619 | 107 517 |
Synonyms:
no synonyms.

Estimated non-polar retention index (n-alkane scale):
Value: 1431 iu
Confidence interval (Ethers): 68(50%) 293(95%) iu

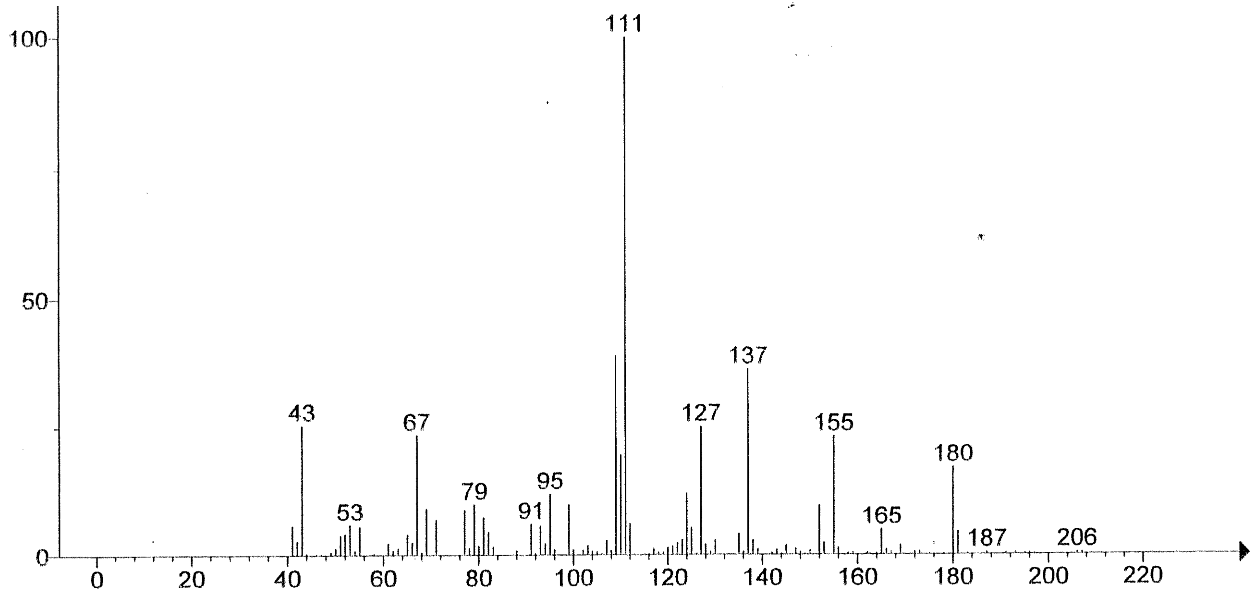

(Text File) +EI Scan (34.275 min) ALI-OLE-H7-220421-.D Subtract

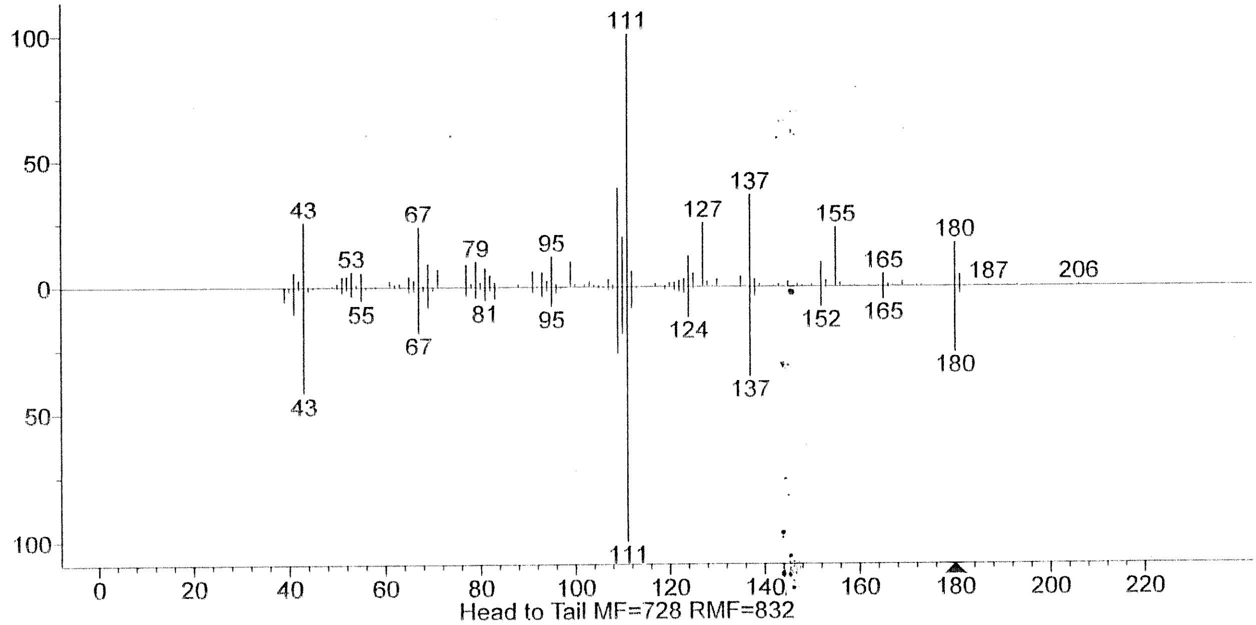

Head to Tail MF=728 RMF=832

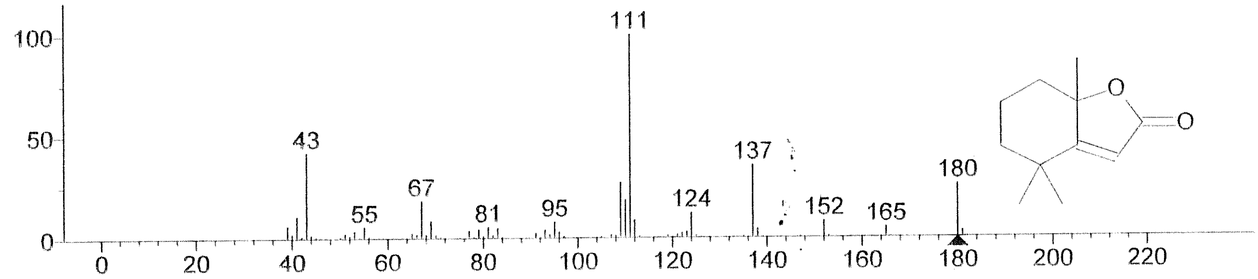

(mainlib) 2(4H)-Benzofuranone, 5,6,7,7a-tetrahydro-4,4,7a-trimethyl-, (R)-

Name: 2(4H)-Benzofuranone, 5,6,7,7a-tetrahydro-4,4,7a-trimethyl-, (R)-
Formula: $C_{11}H_{16}O_2$
MW: 180 CAS#: 17092-92-1 NIST#: 108912 ID#: 74534 DB: mainlib
Other DBs: NIH
Contributor: Philip Morris R&D
10 largest peaks:
111 999 | 43 418 | 137 353 | 109 268 | 180 259 | 110 184 | 67 181 | 124 119 | 41 106 | 112 85 |
Synonyms:
1.2(4H)-Benzofuranone, 5,6,7,7a-tetrahydro-4,4,7a-trimethyl-
2.Actinidiolide, dihydro-
3.2(4H)-Benzofuranone, 5,6,7,7a-tetrahydro-4,4,7a-trimethyl-, (S)-
4.Dihydroactinidiolide
5.4,4,7a-Trimethyl-5,6,7,7a-tetrahydro-1-benzofuran-2(4H)-one #

Estimated non-polar retention index (n-alkane scale):
Value: 1426 iu
Confidence interval (Esters): 47(50%) 201(95%) iu

Retention index.
1. Value: 1532 iu
Column Type: Capillary
Column Class: Standard non-polar
Active Phase: HP-1
Column
Length: 50 m
Carrier Gas: He
Column Diameter: 0.32 mm
Phase Thickness: 1.05 um
Data Type: Linear
RI
Program Type: Complex
Description: 20C(0.5min) =>60C=>4C/min=>250C
Source: Sing, A.S.C.; Smadja, J.;
Brevard, H.; Maignial, L.; Chaintreau, A.; Marion, J.-P., Volatile constituents of faham (Jumellea fragrans (Thou.) Schltr.), J. Agric. Food Chem., 40, 1992, 642-646.

2. Value: 1471 iu
Column Type: Capillary
Column Class:
Standard non-polar
Active Phase: SPB-1
Column Length: 30 m
Carrier Gas: He
Column Diameter: 0.32
mm
Phase Thickness: 0.25 um
Data Type: Kovats RI
Program Type: Complex
Description: 40C(3min)
=>2C/min =>100C=>4C/min =>220C (7min)
Source: Borse, B.B.; Jagan Mohan Rao, L.; Nagalakshmi, S.;
Krishnamurthy, N., Fingerprint of black teas from India: identification of the regio-specific characteristics, Food Chem., 79, 2002, 419-424.

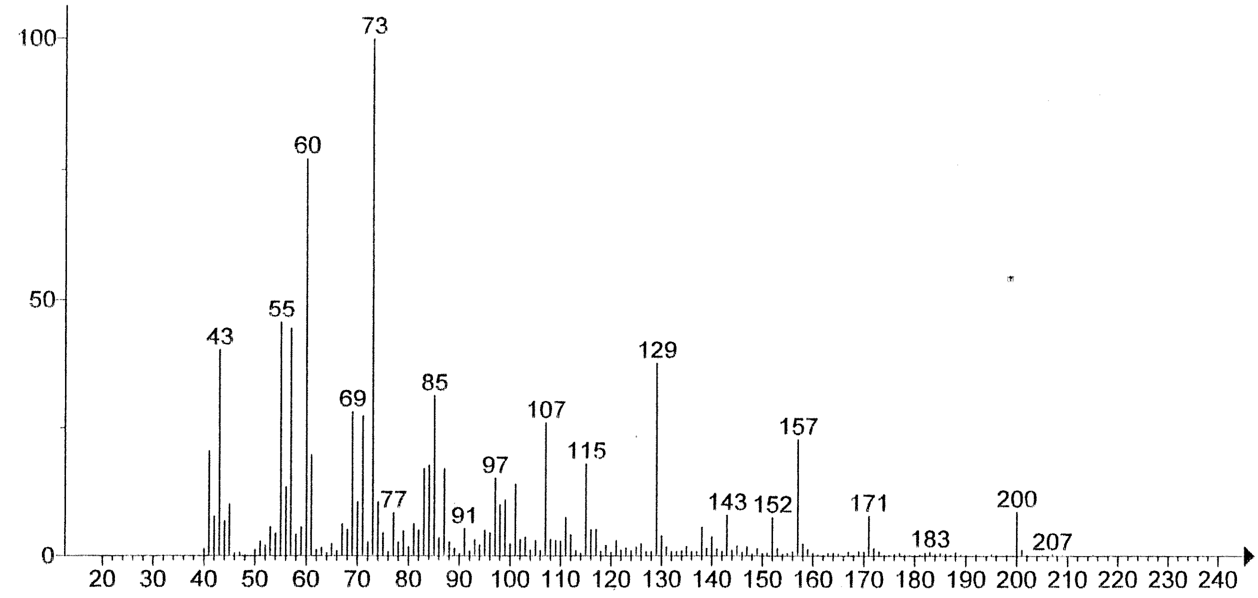

(Text File) +EI Scan (34.635 min) ALI-OLE-H7-220421-.D

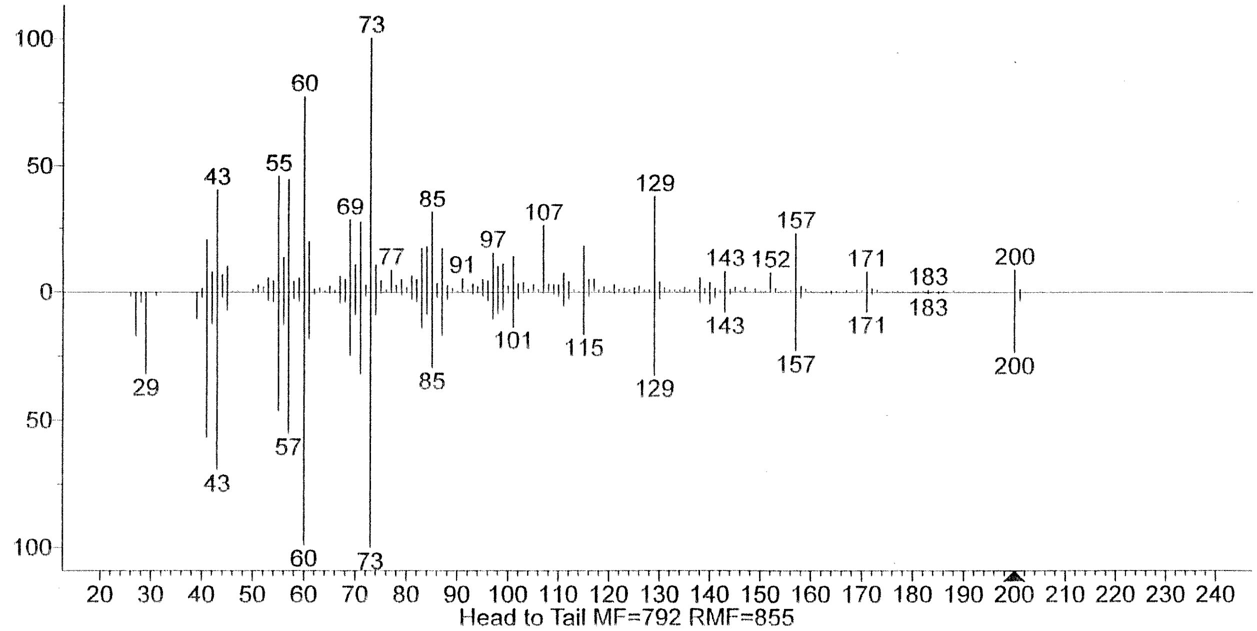

Head to Tail MF=792 RMF=855

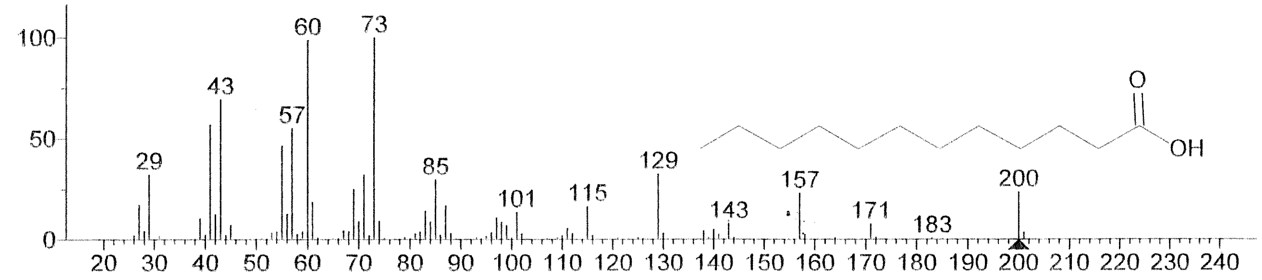

(mainlib) Dodecanoic acid

Name: Dodecanoic acid
Formula: $C_{12}H_{24}O_2$
MW: 200 CAS#: 143-07-7 NIST#: 221043 ID#: 35324 DB: mainlib
Other DBs: Fine, TSCA, RTECS, EPA, HODOC, NIH, EINECS, IRDB
Contributor: Chemical Concepts
10 largest peaks:
73 999 | 60 987 | 43 693 | 41 567 | 57 550 | 55 463 | 129 323 | 29 320 | 71 320 | 85 295 |
Synonyms:
1.n-Dodecanoic acid
2.Neo-fat 12
3.Aliphat no. 4
4.Abl
5.Dodecylic acid
6.Lauric acid
7.Laurostearic acid
8.Neo-fat 12-43
9.Ninol aa62 extra
10.Univol u-314
11.Vulvic acid
12.1-Undecanecarboxylic acid
13.Duodecylic acid
14.C-1297
15.Coconut oil fatty acids
16.Hydrofol acid 1255
17.Hydrofol acid 1295
18.Wecoline 1295
19.Dodecoic acid
20.Hystrene 9512
21.Lunac L 70
22.Duodecyclic acid
23.Emery 650
24.n-Dodecanoate
25.Philacid 1200
26.Prifrac 2920
27.Undecane-1-carboxylic acid

Estimated non-polar retention index (n-alkane scale):
Value: 1570 iu
Confidence interval (Carboxylic acids): 51(50%) 220(95%) iu

Retention index.
1. Value: 1556 iu
Column Type: Capillary
Column Class: Standard non-polar
Active Phase: RTX-1
Column
Length: 60 m
Carrier Gas: He
Column Diameter: 0.22 mm
Phase Thickness: 0.25 um
Data Type: Linear
RI
Program Type: Ramp
Start T: 60 C

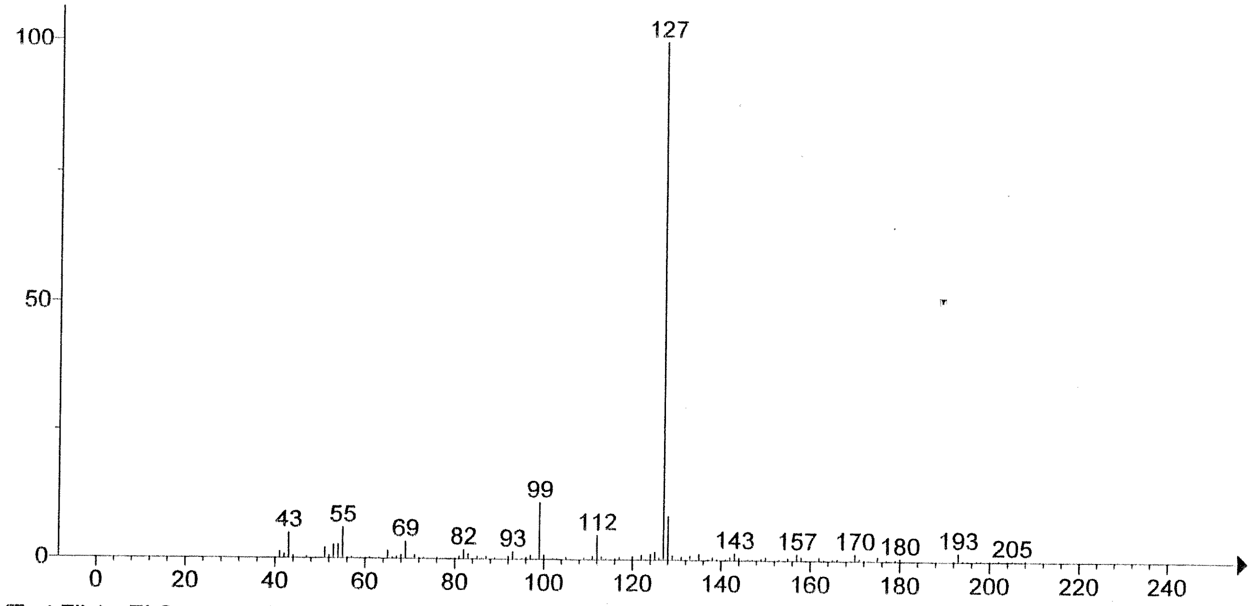

(Text File) +EI Scan (35.123 min) ALI-OLE-H7-220421-.D Subtract

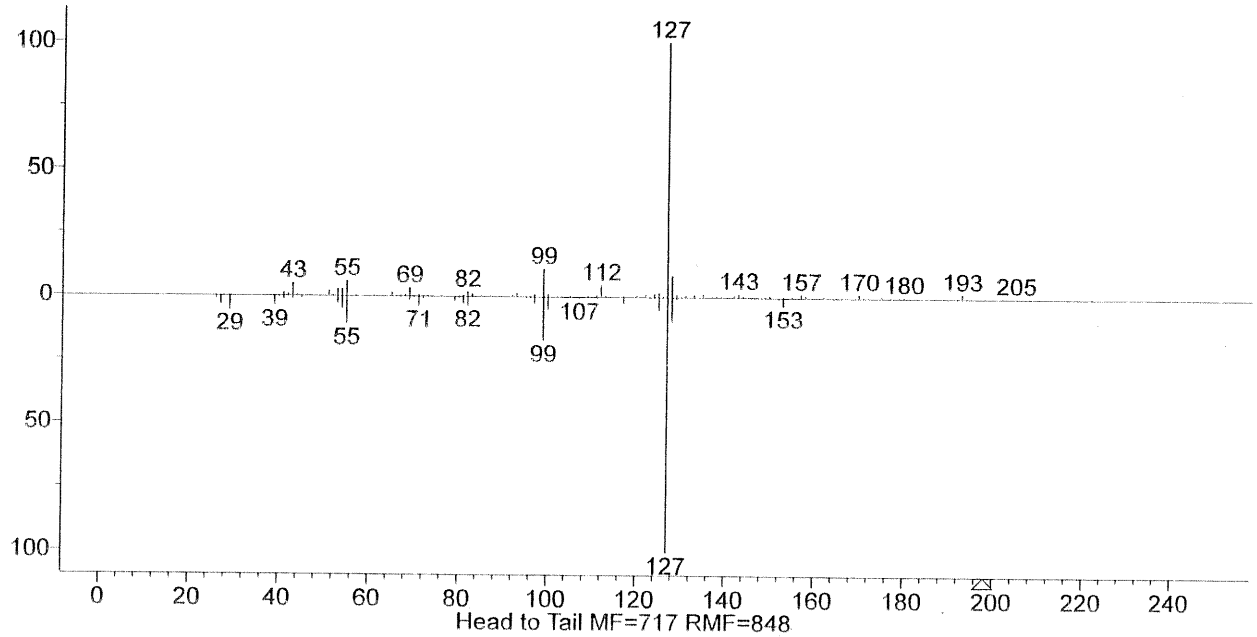

Head to Tail MF=717 RMF=848

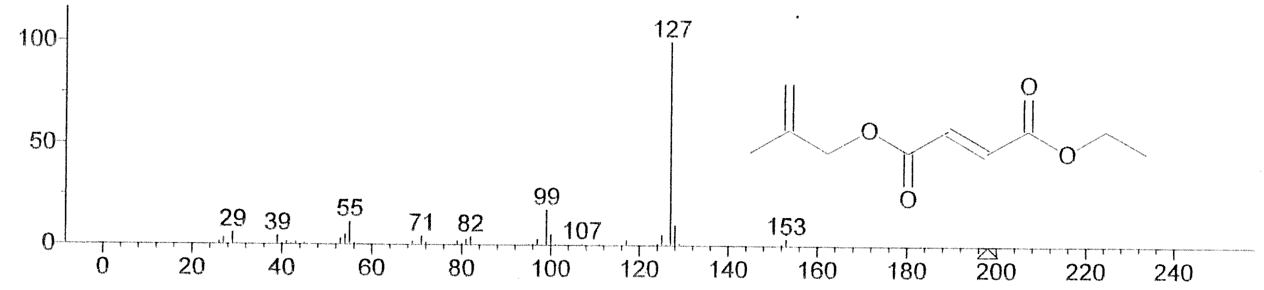

(mainlib) Fumaric acid, ethyl 2-methylallyl ester

Name: Fumaric acid, ethyl 2-methylallyl ester
Formula: $C_{10}H_{14}O_4$
MW: 198 NIST#: 330541 ID#: 89984 DB: mainlib
Contributor: Zaikin V.G., Borisov R.S., TIPS RAS, Moscow, Russia
10 largest peaks:
127 999 | 99 172 | 55 110 | 128 99 | 29 56 | 125 52 | 100 51 | 54 49 | 39 40 | 71 40 |
Synonyms:
no synonyms.

Estimated non-polar retention index (n-alkane scale):
Value: 1325 iu
Confidence interval (Esters): 47(50%) 201(95%) iu

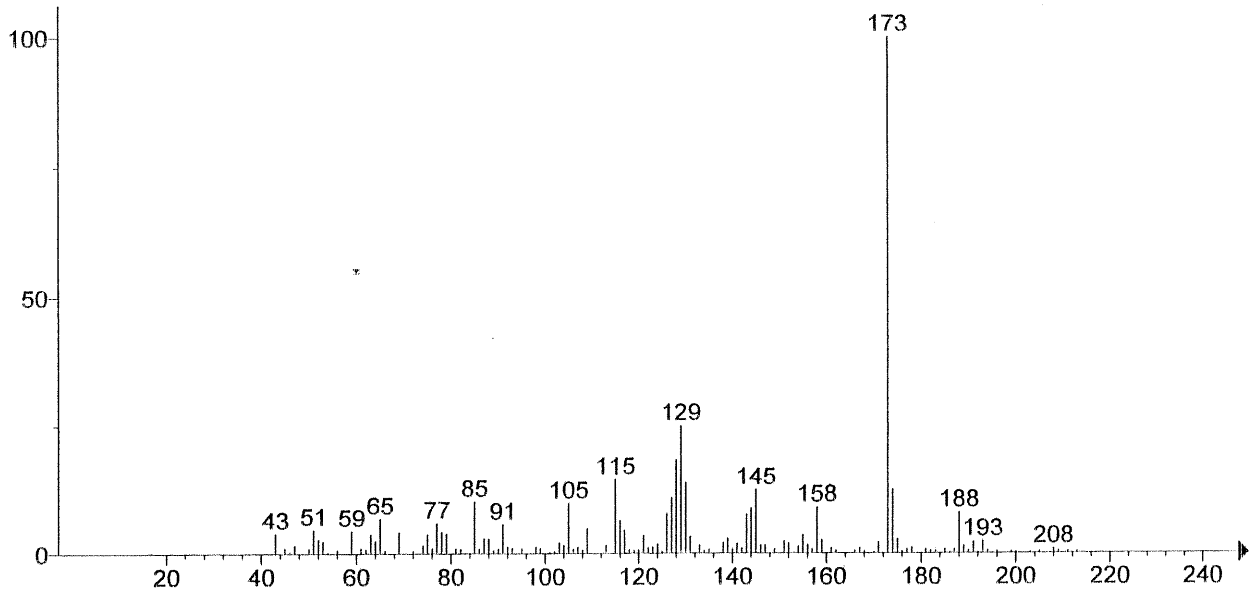

(Text File) +EI Scan (36.531 min) ALI-OLE-H7-220421-.D Subtract

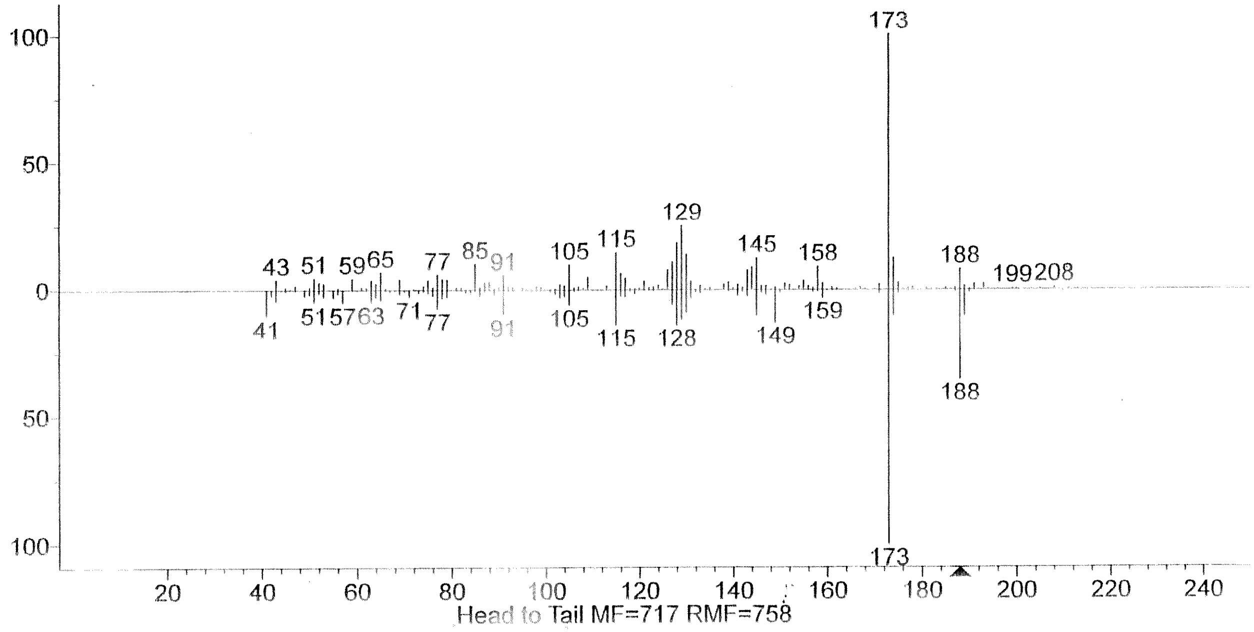

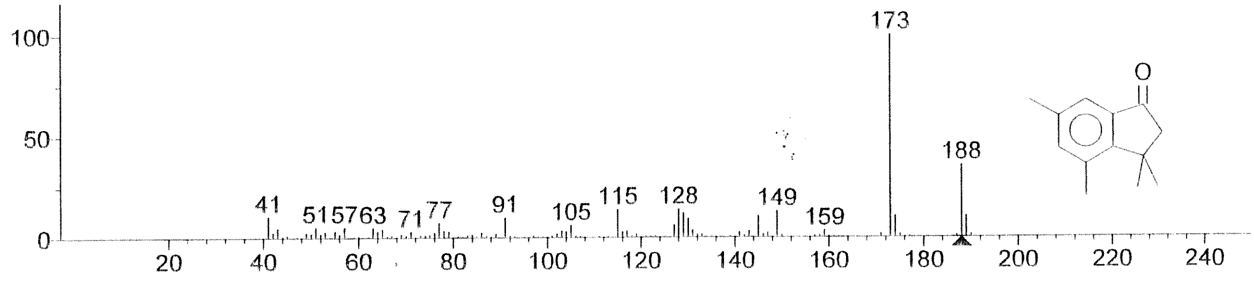

(mainlib) 1H-Inden-1-one, 2,3-dihydro-3,3,4,6-tetramethyl-

Name: 1H-Inden-1-one, 2,3-dihydro-3,3,4,6-tetramethyl-
Formula: $C_{13}H_{16}O$
MW: 188 CAS#: 55255-42-0 NIST#: 129192 ID#: 129096 DB: mainlib
Other DBs: None
Contributor: LAC, NIDDK, NIH, Bethesda, MD 20892
10 largest peaks:
173 999 | 188 353 | 128 139 | 115 138 | 149 129 | 129 119 | 41 105 | 145 105 | 174 105 | 189 105 |
Synonyms:
1.Benzo[b]dihydrofuran, 3,3,4,6-tetramethyl-
2.3,3,4,6-Tetramethyl-1-indanone #

Estimated non-polar retention index (n-alkane scale):
Value: 1579 iu
Confidence interval (Ketones): 57(50%) 246(95%) iu

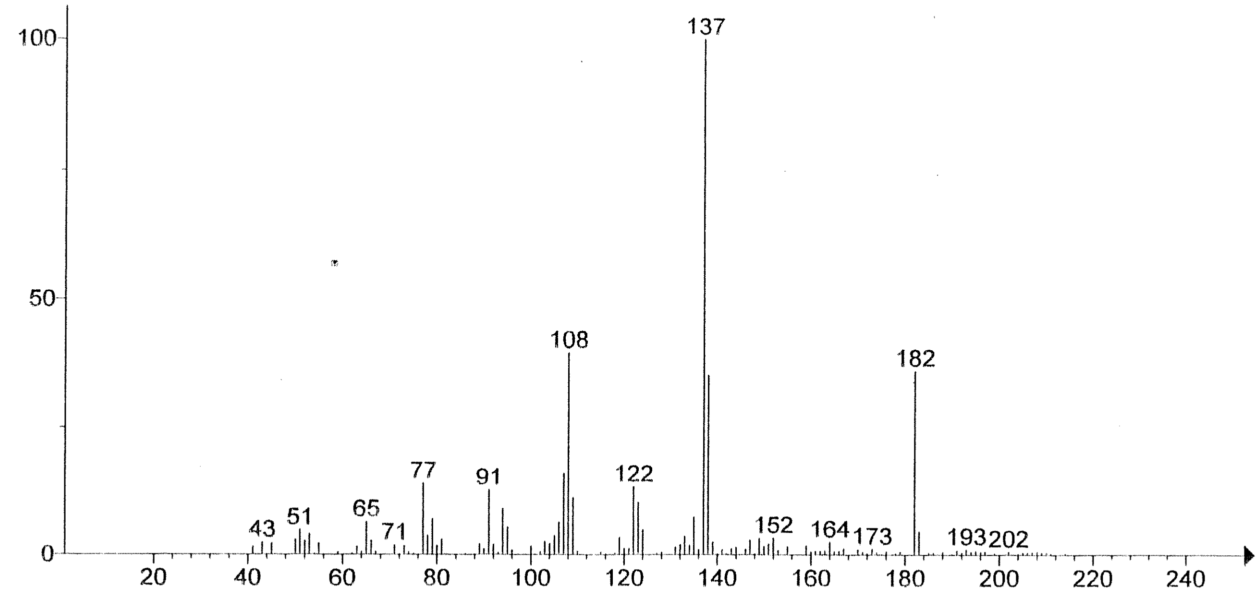

(Text File) +EI Scan (37.454 min) ALI-OLE-H7-220421-.D Subtract

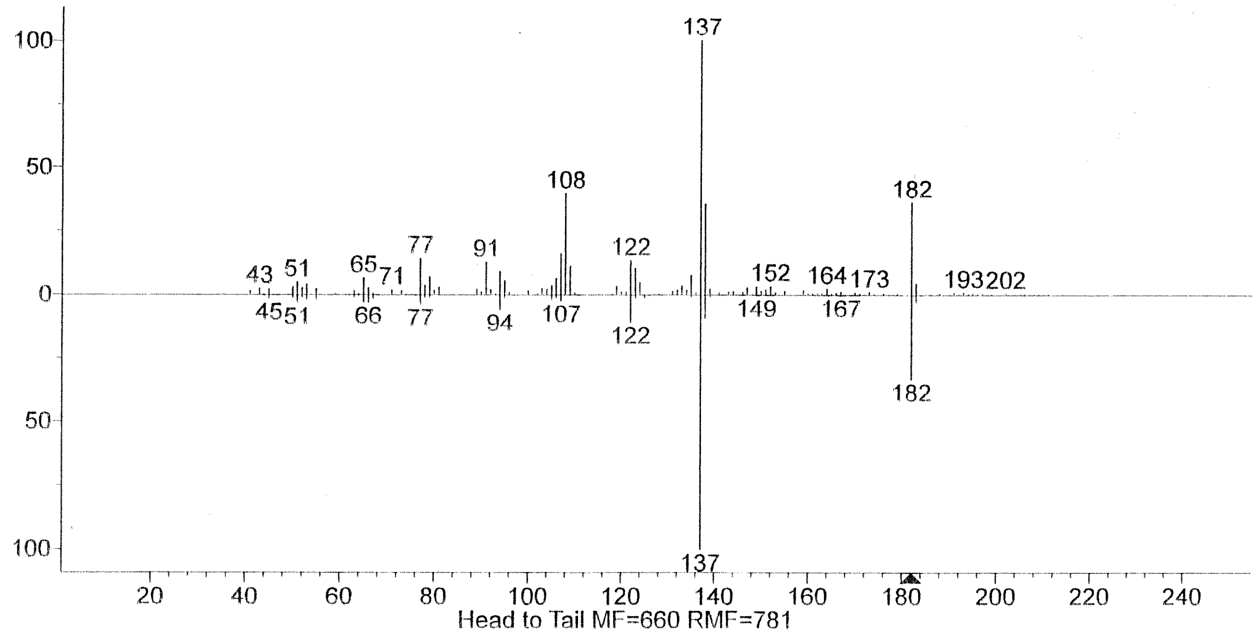

Head to Tail MF=660 RMF=781

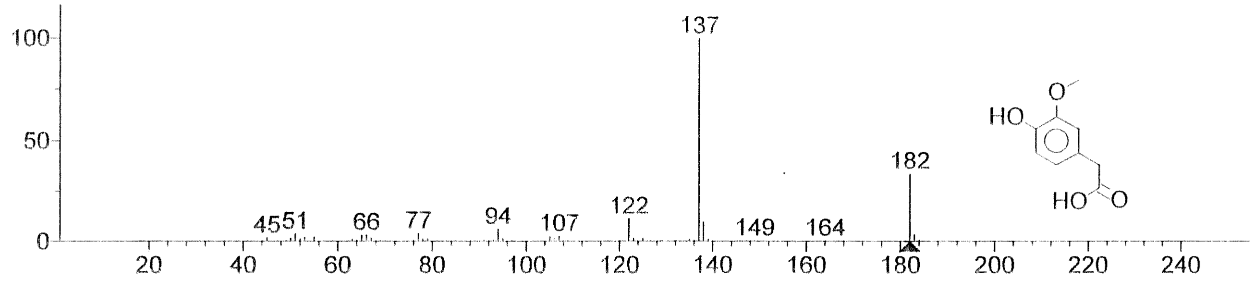

(replib) Benzeneacetic acid, 4-hydroxy-3-methoxy-

71

Name: Benzeneacetic acid, 4-hydroxy-3-methoxy-
Formula: $C_9H_{10}O_4$
MW: 182 CAS#: 306-08-1 NIST#: 248367 ID#: 18756 DB: replib
Other DBs: Fine, TSCA, EPA, HODOC, NIH, EINECS
Contributor: Patti Price, Georgia Bureau of Investigation, Decatur, Georgia
10 largest peaks:
137 999 | 182 334 | 122 110 | 138 95 | 94 59 | 77 39 | 51 35 | 183 32 | 66 31 | 65 29 |
Synonyms:
1.Acetic acid, (4-hydroxy-3-methoxyphenyl)-
2.(4-Hydroxy-3-methoxyphenyl)acetic acid
3.Homovanillic acid
4.HMPA
5.HVA
6.Vanillacetic Acid
7.3-Methoxy-4-hydroxyphenylacetic acid
8.4-Hydroxy-3-methoxybenzeneacetic acid
9.Homovanillinic acid
10.Homovanilic acid
11.Vanilacetic acid

Estimated non-polar retention index (n-alkane scale):
Value: 1659 iu
Confidence interval (Diverse functional groups): 89(50%) 382(95%) iu

Retention index.
1. Value: 1603 iu
Column Type: Capillary
Column Class: Standard non-polar
Active Phase: HP-1
Column
Length: 50 m
Carrier Gas: He
Column Diameter: 0.2 mm
Phase Thickness: 0.5 um
Data Type: Normal alkane
RI
Program Type: Ramp
Start T: 60 C
End T: 250 C
Heat Rate: 2 K/min
End Time: 120 min
Source: Castel,
C.; Fernandez, X.; Lizzani-Cuvelier, L.; Perichet, C.; Lavoine, S., Characterization of the Chemical Composition of a Byproduct from Siam Benzoin Gum, J. Agric. Food Chem., 54, 2006, 8848-8854.

2. Value: 1633 iu
Column
Type: Capillary
Column Class: Semi-standard non-polar
Active Phase: DB-5MS
Column Length: 30 m
Carrier
Gas: H2/N2
Column Diameter: 0.25 mm

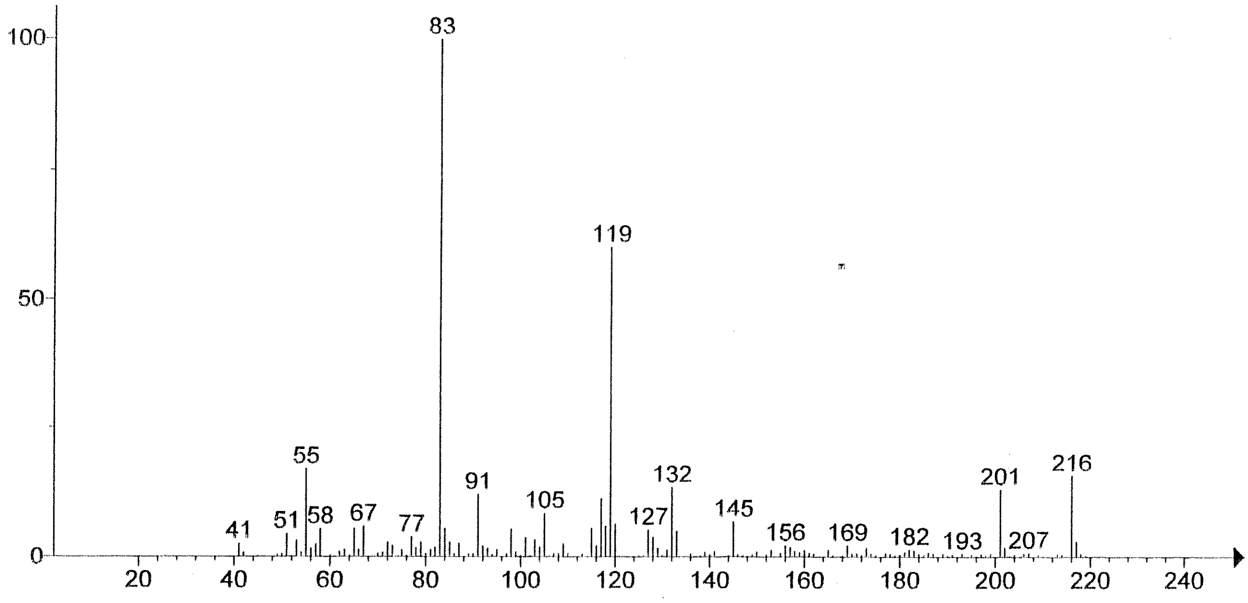

(Text File) +EI Scan (37.963 min) ALI-OLE-H7-220421-.D Subtract

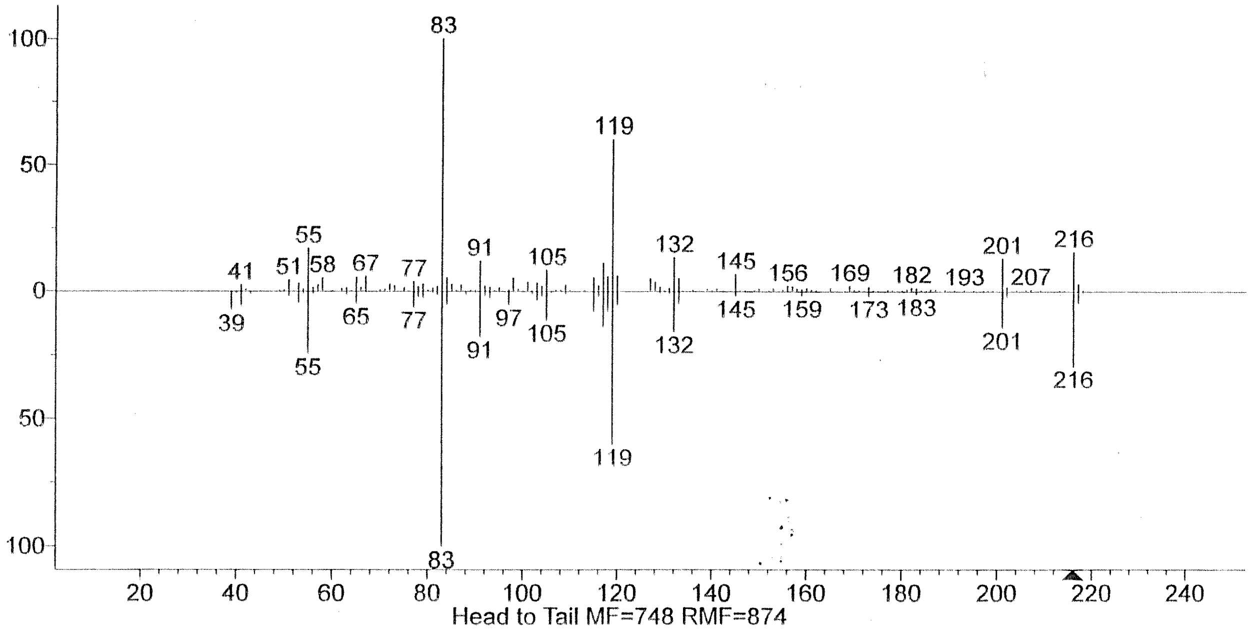

Head to Tail MF=748 RMF=874

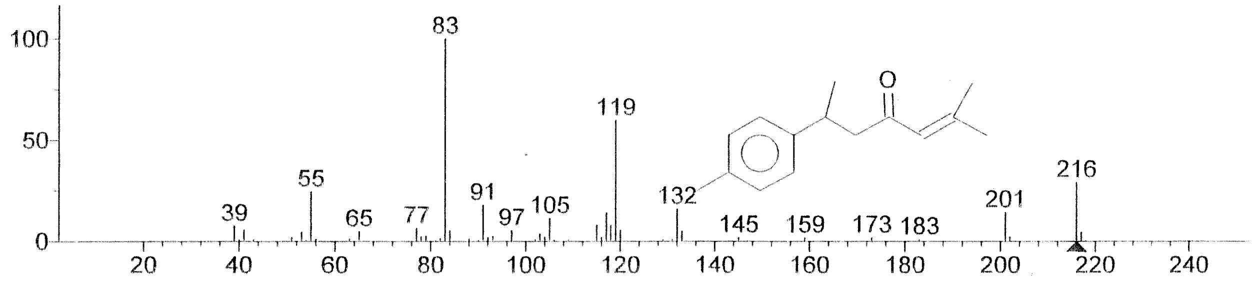

(mainlib) Ar-tumerone

Name: Ar-tumerone
Formula: $C_{15}H_{20}O$
MW: 216 NIST#: 292710 ID#: 45357 DB: mainlib
Contributor: R.Hiserodt ET AL. J.Chrom. 740,51(1996)
10 largest peaks:
83 999 | 119 597 | 216 290 | 55 243 | 91 177 | 132 157 | 201 141 | 117 140 | 105 112 | 118 79 |
Synonyms:
no synonyms.

Estimated non-polar retention index (n-alkane scale):
Value: 1660 iu
Confidence interval (Ketones): 57(50%) 246(95%) iu

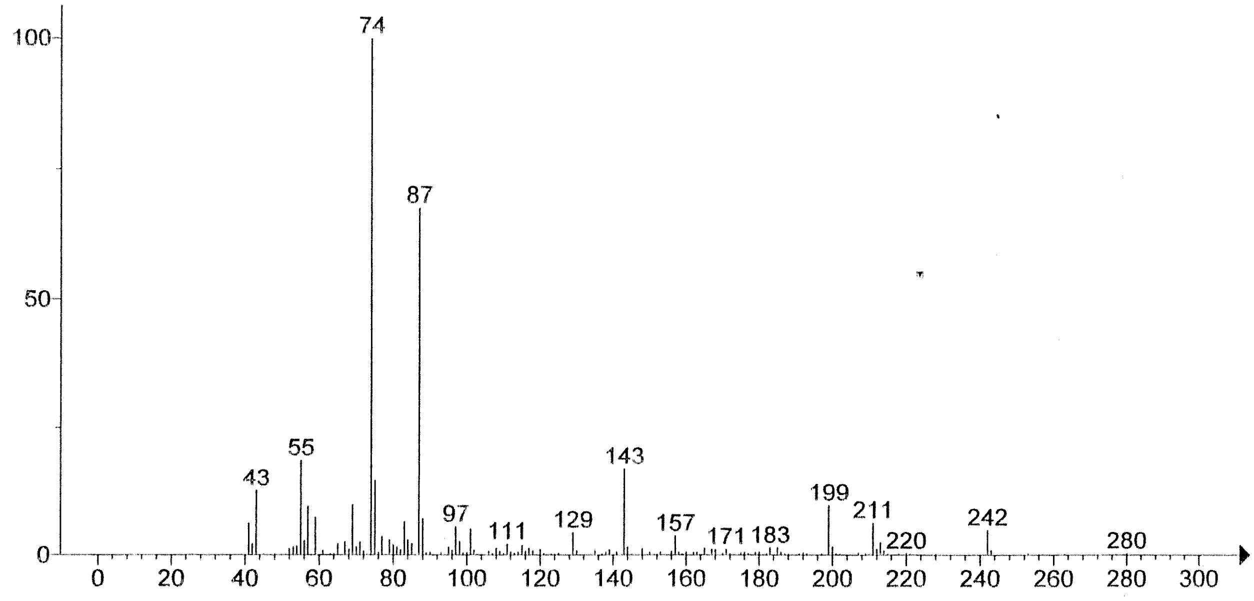

(Text File) +EI Scan (39.346 min) ALI-OLE-H7-220421-.D Subtract

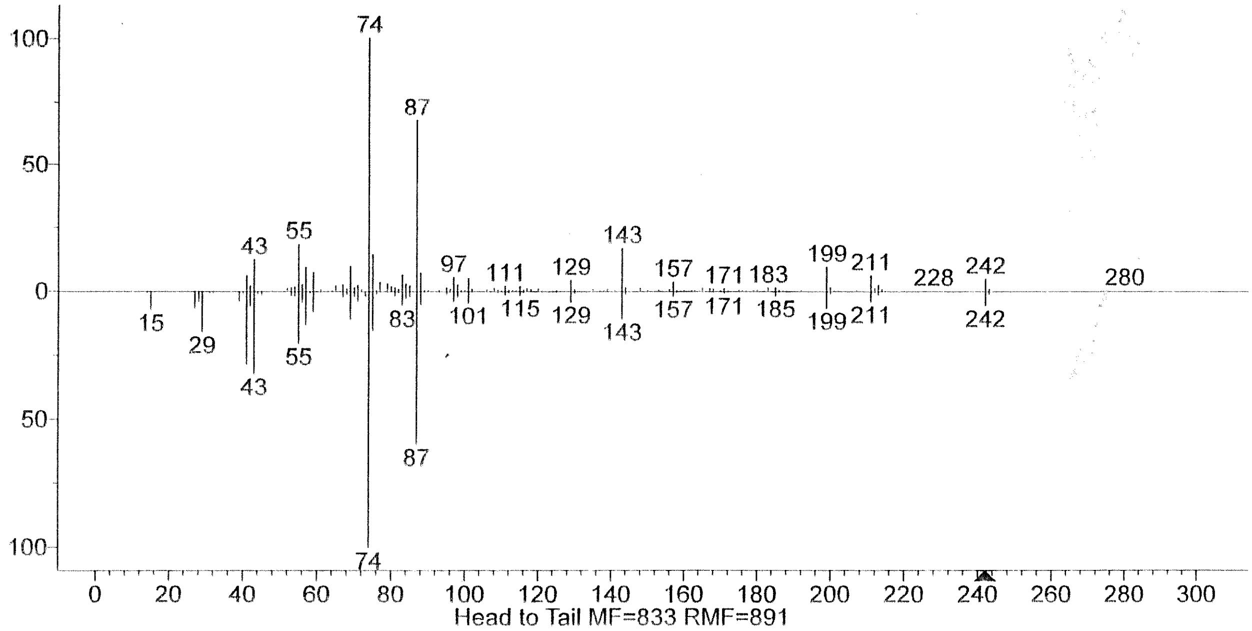

Head to Tail MF=833 RMF=891

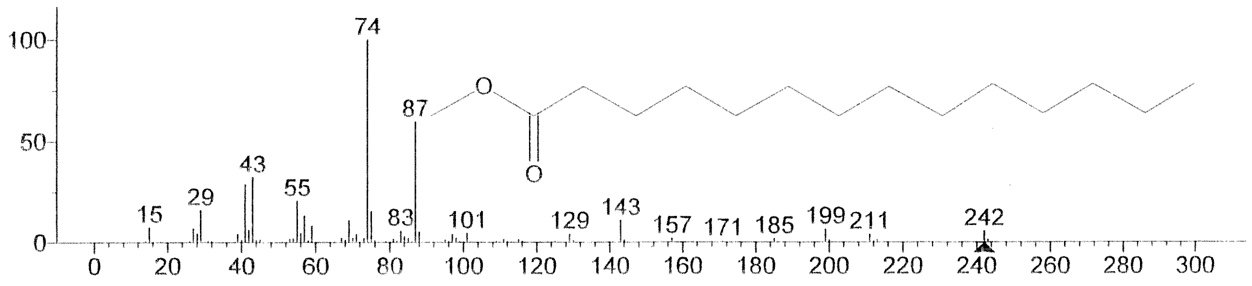

(replib) Methyl tetradecanoate

Name: Methyl tetradecanoate
Formula: $C_{15}H_{30}O_2$
MW: 242 CAS#: 124-10-7 NIST#: 79125 ID#: 9077 DB: replib
Other DBs: Fine, TSCA, EPA, HODOC, NIH, EINECS, IRDB
Contributor: O A MAMER, MCGILL UNIVERSITY, MONTREAL, HEM. P
10 largest peaks:
74 999 | 87 595 | 43 321 | 41 286 | 55 204 | 29 158 | 75 153 | 57 131 | 69 109 | 143 107 |
Synonyms:
1.Tetradecanoic acid, methyl ester
2.Myristic acid, methyl ester
3.Metholeneat 2495
4.Methyl myristate
5.Methyl n-tetradecanoate
6.Uniphat A50
7.Emery 2214

Estimated non-polar retention index (n-alkane scale):
Value: 1680 iu
Confidence interval (Esters): 47(50%) 201(95%) iu

Retention index.
1. Value: 1713 iu
Column Type: Capillary
Column Class: Standard non-polar
Active Phase: CP Sil 5 CB
Column
Length: 60 m
Carrier Gas: He
Column Diameter: 0.32 mm
Phase Thickness: 0.25 um
Data Type: Linear
RI
Program Type: Ramp
Start T: 60 C
End T: 280 C
Heat Rate: 3 K/min
Start Time: 10 min
End Time: 60
min
Source: Pino, J.; Almora, K.; Marbot, R., Volatile components of papaya (Carica papaya L., maradol variety) fruit, Flavour Fragr. J., 18, 2003, 492-496.

2. Value: 1714 iu
Column Type: Capillary
Column Class: Standard
non-polar
Active Phase: HP-1
Column Length: 30 m
Carrier Gas: He
Column Diameter: 0.25 mm
Phase
Thickness: 0.33 um
Data Type: Linear RI
Program Type: Ramp

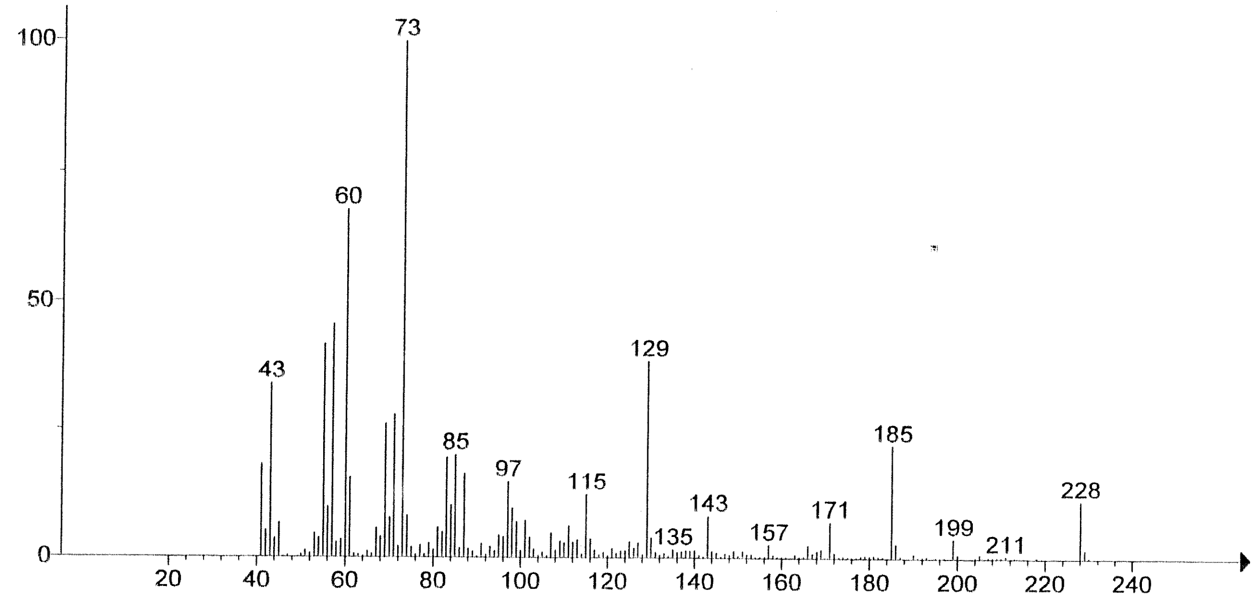

(Text File) +EI Scan (40.312 min) ALI-OLE-H7-220421-.D

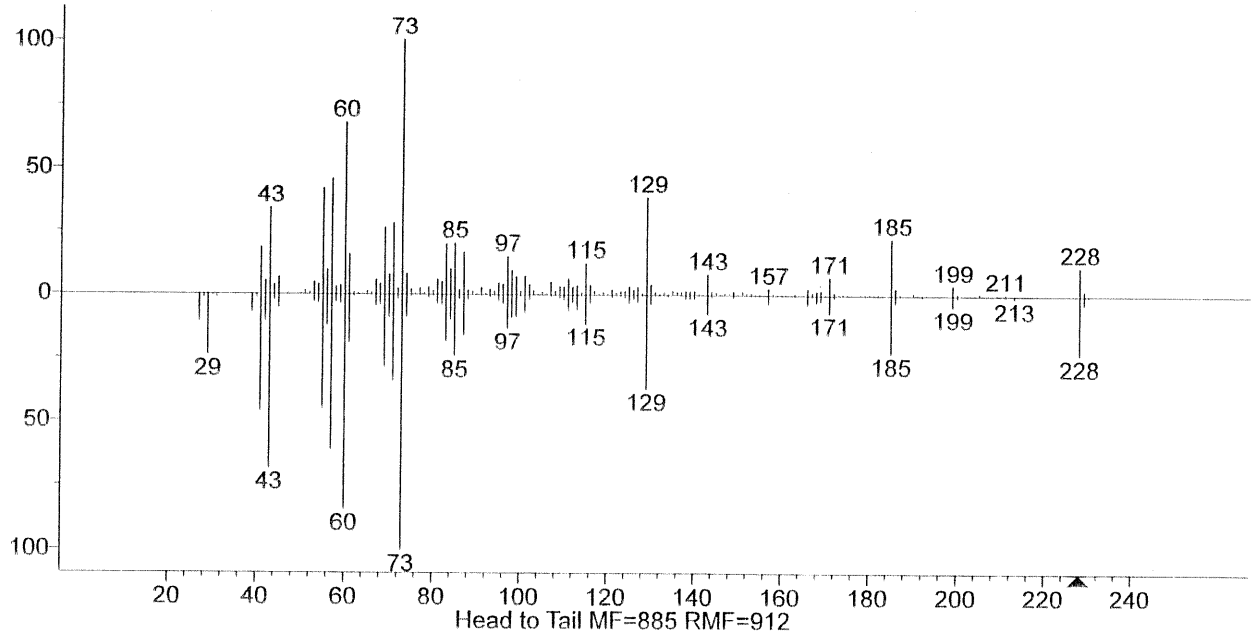

Head to Tail MF=885 RMF=912

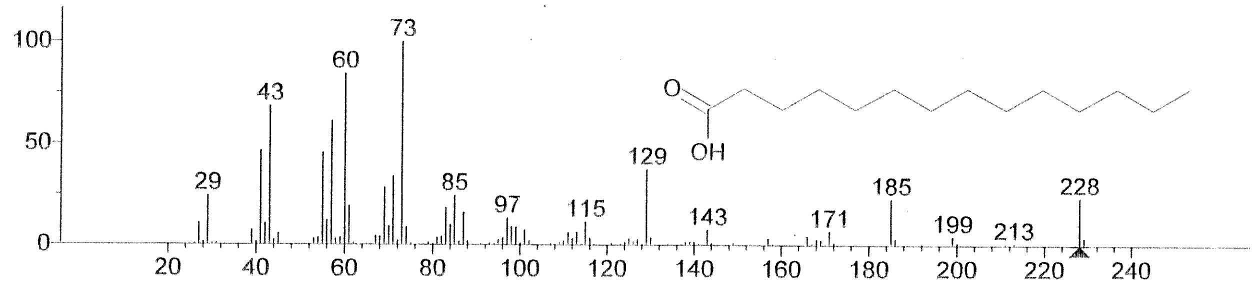

(replib) Tetradecanoic acid

77

Name: Tetradecanoic acid
Formula: $C_{14}H_{28}O_2$
MW: 228 CAS#: 544-63-8 NIST#: 189107 ID#: 8471 DB: replib
Other DBs: Fine, TSCA, RTECS, EPA, HODOC, NIH, EINECS, IRDB
Contributor: Chemical Concepts
10 largest peaks:
73 999 | 60 841 | 43 681 | 57 609 | 41 461 | 55 451 | 129 371 | 71 338 | 69 282 | 85 241 |
Synonyms:
1.Myristic acid
2.n-Tetradecanoic acid
3.n-Tetradecoic acid
4.Neo-Fat 14
5.Univol U 316S
6.1-Tridecanecarboxylic acid
7.Coconut oil fatty acids
8.Crodacid
9.Emery 655
10.Hydrofol acid 1495
11.Hystrene 9014
12.n-Tetradecan-1-oic acid
13.Emery 654
14.Hystrene 9514
15.Philacid 1400
16.Prifac 2940
17.Prifrac 2940
18.Univol U 3165
19.Univol U320

Estimated non-polar retention index (n-alkane scale):
Value: 1769 iu
Confidence interval (Carboxylic acids): 51(50%) 220(95%) iu

Retention index.
1. Value: 1748 iu
Column Type: Capillary
Column Class: Standard non-polar
Active Phase: RTX-1
Column
Length: 60 m
Carrier Gas: He
Column Diameter: 0.22 mm
Phase Thickness: 0.25 um
Data Type: Linear
RI
Program Type: Ramp
Start T: 60 C
End T: 230 C
Heat Rate: 2 K/min
End Time: 35 min
Source: Paolini, J.;
Muselli, A.; Bernardini, A.-F.; Bighelli, A.; Casanova, J.; Costa, J., Thymol derivatives from essential oil of Doronicum corsicum L., Flavour Fragr. J., 22, 2007, 479-487.

2. Value: 1772 iu

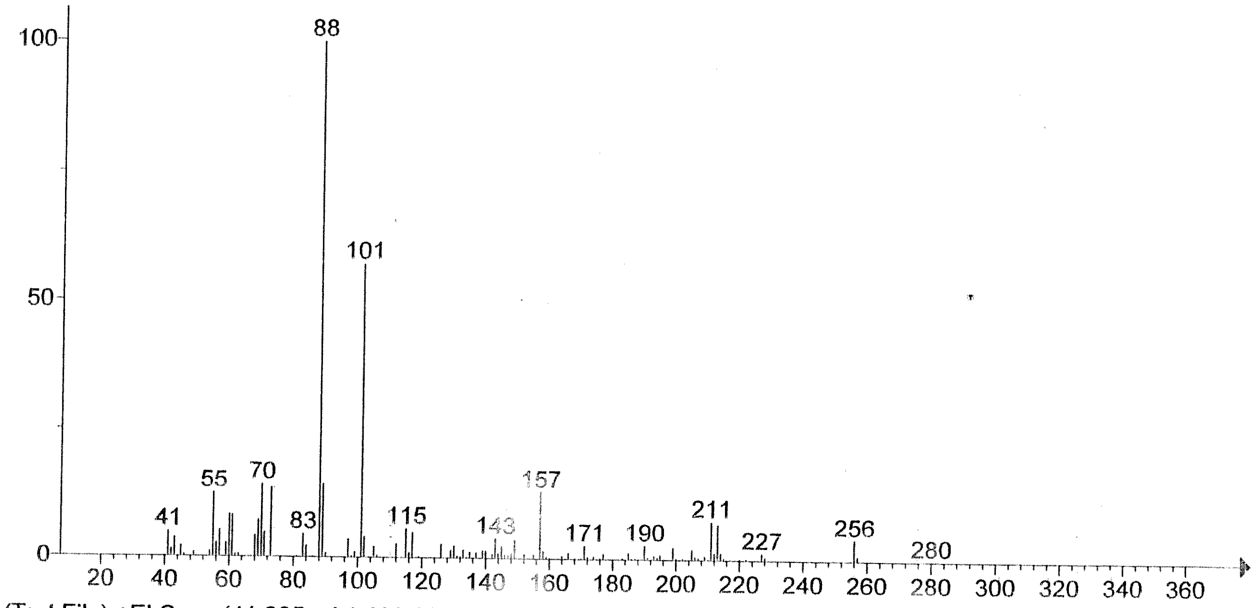

(Text File) +EI Scan (41.285 min) ALI-OLE-H7-220421-.D Subtract

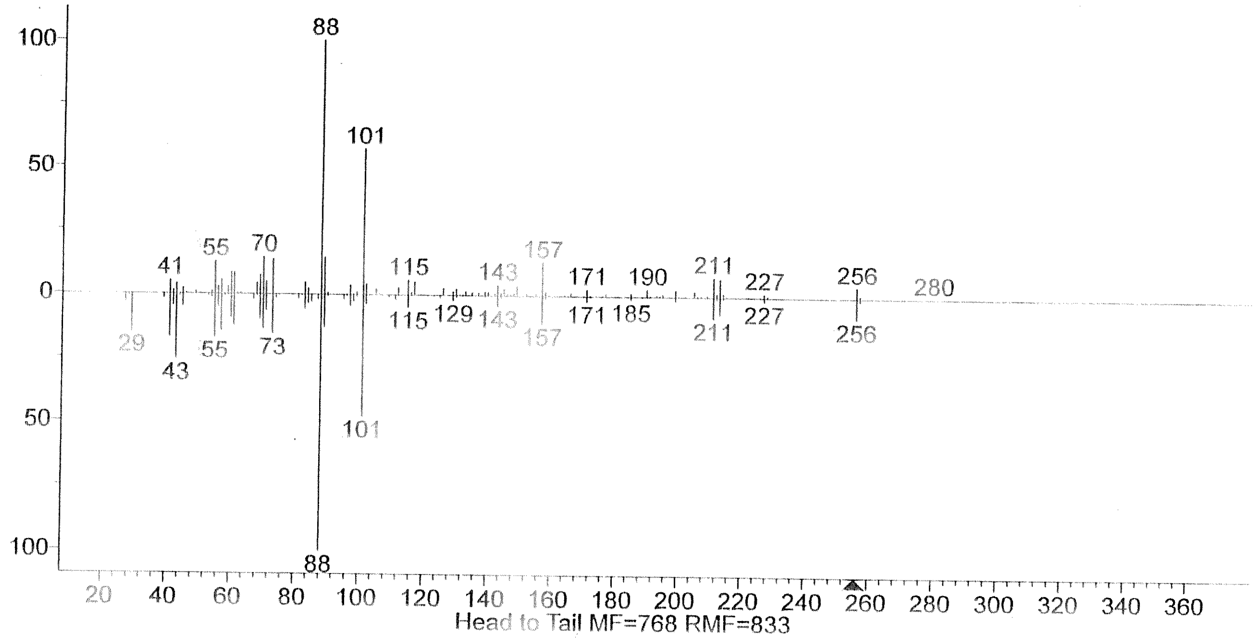

Head to Tail MF=768 RMF=833

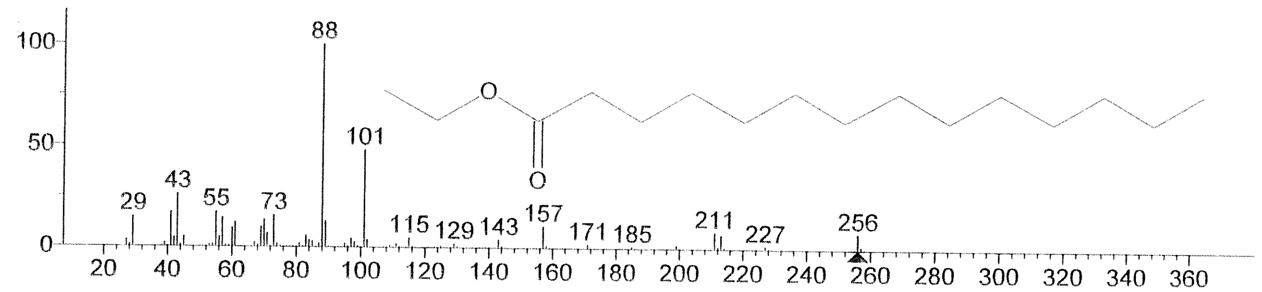

(replib) Tetradecanoic acid, ethyl ester

Name: Tetradecanoic acid, ethyl ester
Formula: $C_{16}H_{32}O_2$
MW: 256 CAS#: 124-06-1 NIST#: 156923 ID#: 11112 DB: replib
Other DBs: Fine, TSCA, EPA, HODOC, NIH, EINECS, IRDB
Contributor: Chemical Concepts
10 largest peaks:
88 999 | 101 477 | 43 257 | 55 170 | 41 168 | 73 154 | 29 147 | 57 143 | 70 134 | 89 128 |
Synonyms:
1.Myristic acid, ethyl ester
2.Ethyl myristate
3.Ethyl tetradecanoate
4.Ethyl ester of tetradecanoic acid
5.Ethyl N-tetradecanoate

Estimated non-polar retention index (n-alkane scale):
Value: 1779 iu
Confidence interval (Esters): 47(50%) 201(95%) iu

Retention index.
1. Value: 1778 iu
Column Type: Capillary
Column Class: Standard non-polar
Active Phase: BP-1
Column
Length: 30 m
Carrier Gas: N2
Column Diameter: 0.32 mm
Phase Thickness: 0.25 um
Data Type: Linear
RI
Program Type: Ramp
Start T: 60 C
End T: 220 C
Heat Rate: 5 K/min
End Time: 13 min
Source: Raina, V.
K.; Kumar, A.; Srivastava, S.K.; Syamsundar, K.V.; Kahol, A.P., Essential oil composition of 'kewda' (Pandanus odoratissimus) from India, Flavour Fragr. J., 19, 2004, 434-436.

2. Value: 1782 iu
Column Type:
Capillary
Column Class: Standard non-polar
Active Phase: CP Sil 5 CB
Column Length: 60 m
Carrier Gas:
He
Column Diameter: 0.32 mm
Phase Thickness: 0.25 um
Data Type: Linear RI
Program Type: Ramp
Start T:
60 C
End T: 280 C

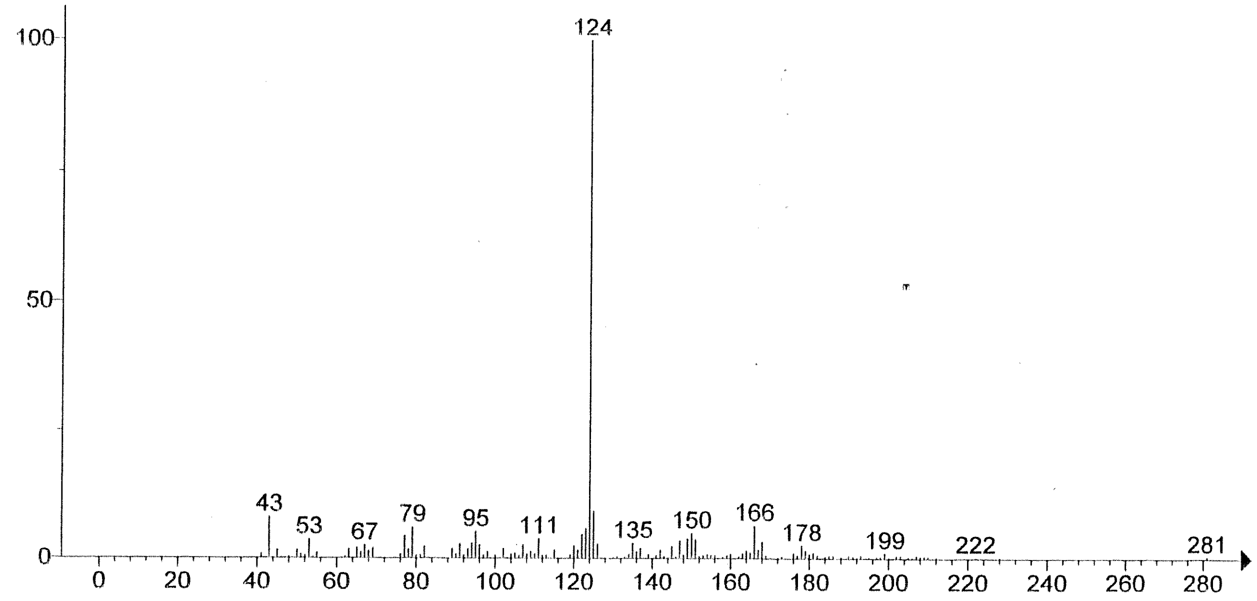

(Text File) +EI Scan (41.588 min) ALI-OLE-H7-220421-.D Subtract

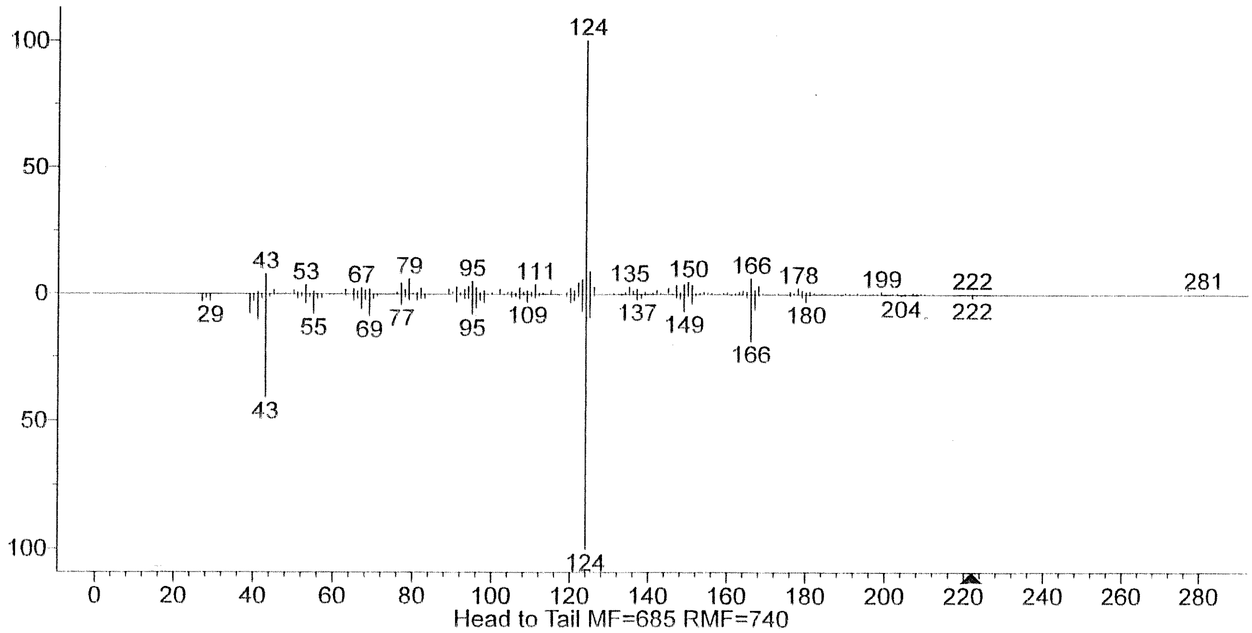

Head to Tail MF=685 RMF=740

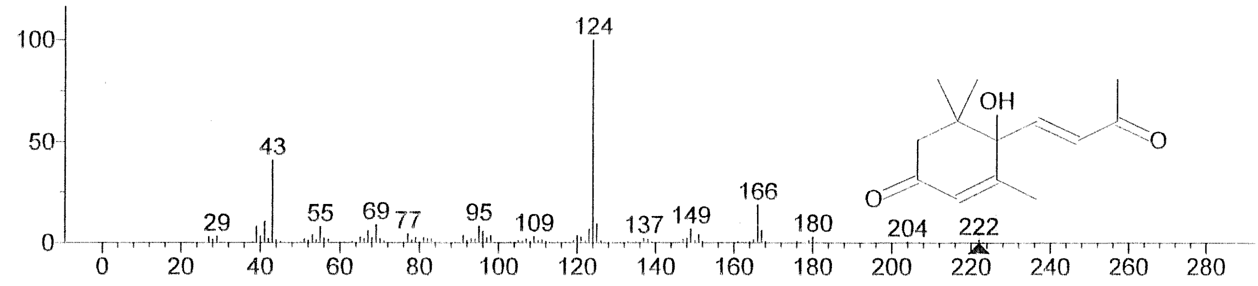

(replib) 2-Cyclohexen-1-one, 4-hydroxy-3,5,5-trimethyl-4-(3-oxo-1-butenyl)-

81

Name: 2-Cyclohexen-1-one, 4-hydroxy-3,5,5-trimethyl-4-(3-oxo-1-butenyl)-
Formula: $C_{13}H_{18}O_3$
MW: 222 CAS#: 7070-24-8 NIST#: 188889 ID#: 17031 DB: replib
Other DBs: None
Contributor: Chemical Concepts
10 largest peaks:
124 999 | 43 408 | 166 186 | 41 105 | 125 93 | 69 88 | 95 82 | 39 80 | 55 79 | 123 68 |
Synonyms:
1.4-Hydroxy-3,5,5-trimethyl-4-[(1E)-3-oxo-1-butenyl]-2-cyclohexen-1-one #

Estimated non-polar retention index (n-alkane scale):
Value: 1751 iu
Confidence interval (Diverse functional groups): 89(50%) 382(95%) iu

Retention index.
1. Value: 1729 iu
Column Type: Capillary
Column Class: Standard non-polar
Active Phase: BP-1
Column
Length: 12 m
Carrier Gas: H2
Data Type: Linear RI
Program Type: Ramp
Start T: 40 C
End T: 240 C
Heat
Rate: 4 K/min
Start Time: 2 min
End Time: 75 min
Source: Tan, S.T.; Wilkins, A.L.; Holland, P.T.; McGhie, T.K.,
Extractives from New Zealand unifloral honeys. 2. Degraded carotenoids and other substances from heather honey, J. Agric. Food Chem., 37, 1989, 1217-1221.

2. Value: 1760 iu
Column Type: Capillary
Column Class: Standard
non-polar
Active Phase: CP Sil 5 CB
Column Length: 50 m
Carrier Gas: He
Column Diameter: 0.32 mm
Phase
Thickness: 1.2 um
Data Type: Normal alkane RI
Program Type: Complex
Description: 36 0C ^ 20 K/min -> 85 0C
^ 1 K/min -> 145 oC ^ 3 K/min -> 250 0C
Source: Guyot-Declerck, C.; Chevance, F.; Lermusieau, G.; Collin, S.,
Optimized extraction procedure for quantifying norisoprenoids in honey and honey food products, J. Agric. Food Chem., 48, 2000, 5850-5855.

<...>

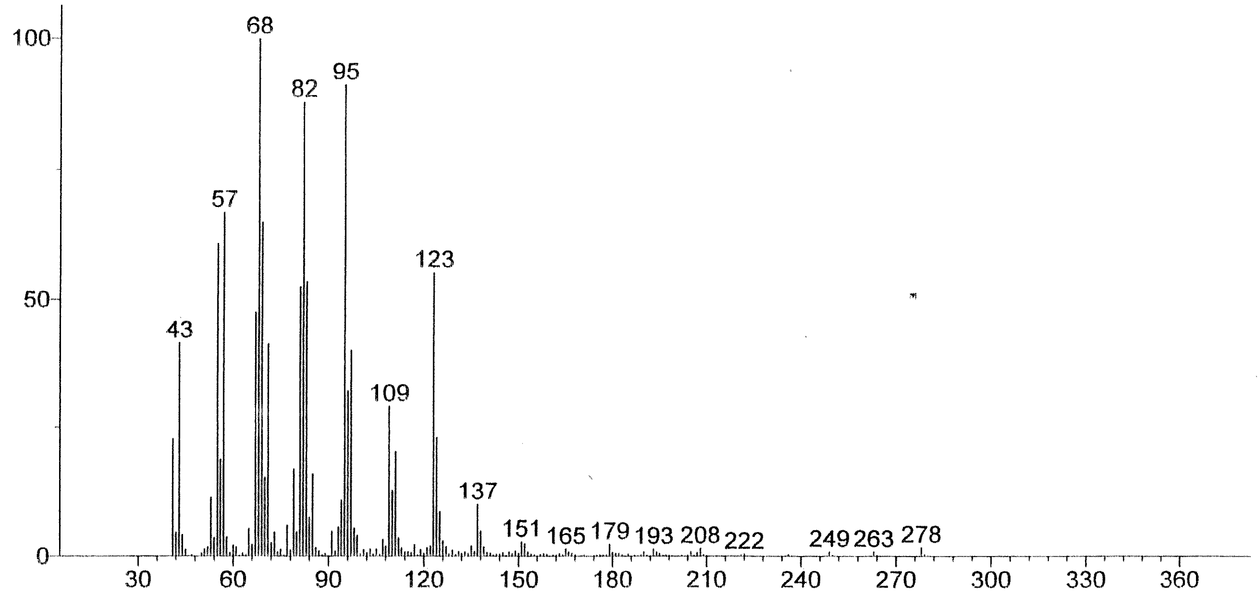

(Text File) +EI Scan (42.800 min) ALI-OLE-H7-220421-.D

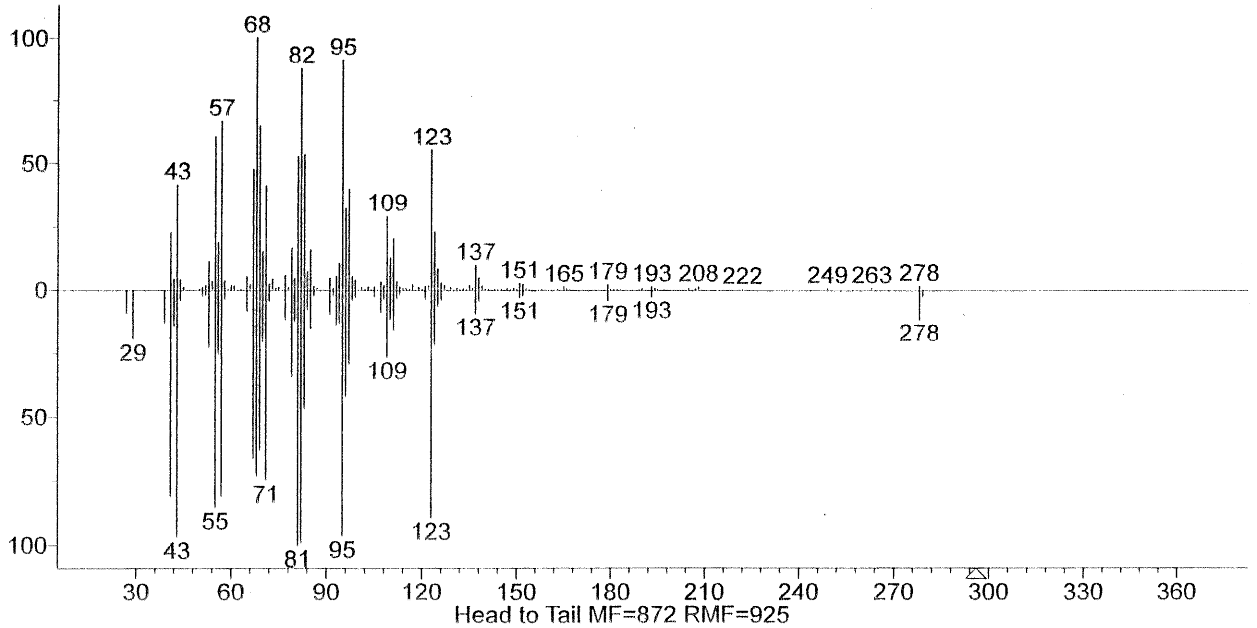

Head to Tail MF=872 RMF=925

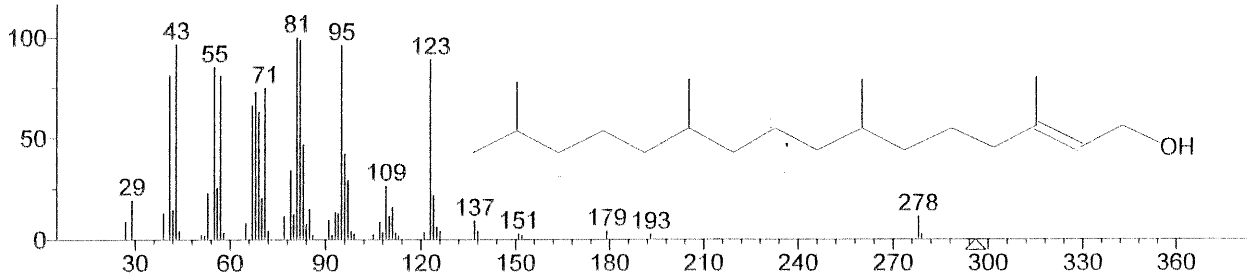

(mainlib) 3,7,11,15-Tetramethyl-2-hexadecen-1-ol

85

Name: 3,7,11,15-Tetramethyl-2-hexadecen-1-ol
Formula: $C_{20}H_{40}O$
MW: 296 CAS#: 102608-53-7 NIST#: 114703 ID#: 43206 DB: mainlib
Other DBs: IRDB
Contributor: NIST Mass Spectrometry Data Center, 1990.
10 largest peaks:
81 999 | 82 986 | 43 965 | 95 962 | 123 892 | 55 852 | 41 811 | 57 811 | 71 748 | 68 728 |
Synonyms:
1.(2E)-3,7,11,15-Tetramethyl-2-hexadecen-1-ol #

Estimated non-polar retention index (n-alkane scale):
Value: 2045 iu
Confidence interval (Alcohols): 41(50%) 176(95%) iu

Retention index.
1. Value: 2119.33 iu
Column Type: Capillary
Column Class: Semi-standard non-polar
Active Phase: SE
-54
Column Length: 25 m
Column Diameter: 0.31 mm
Data Type: Linear RI
Program Type: Ramp
Start T: 35
C
End T: 230 C
Heat Rate: 4 K/min
Start Time: 3 min
End Time: 10 min
Source: Yin, W.; Xiu, Z.; Aijin, H.,
Analysis of the volatile components in trogopterorum feces by capillary gas chromatography and gas chromatography/mass spectrometry, Fenxi Huaxue, 29(2), 2001, 195-198.

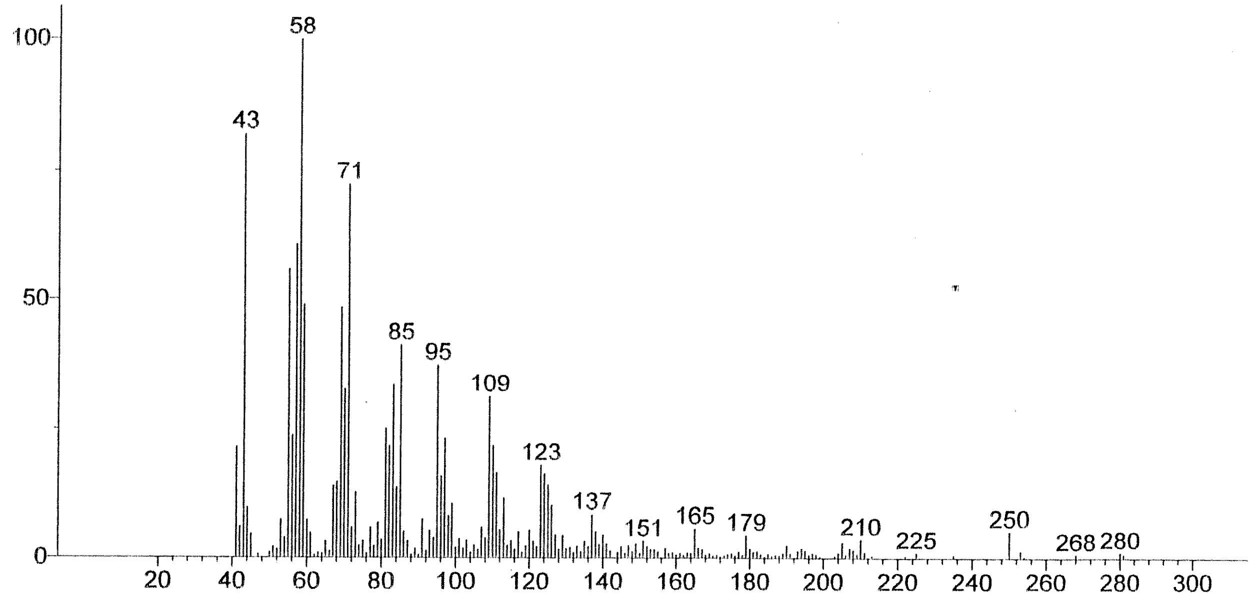

(Text File) +EI Scan (43.028 min) ALI-OLE-H7-220421-.D

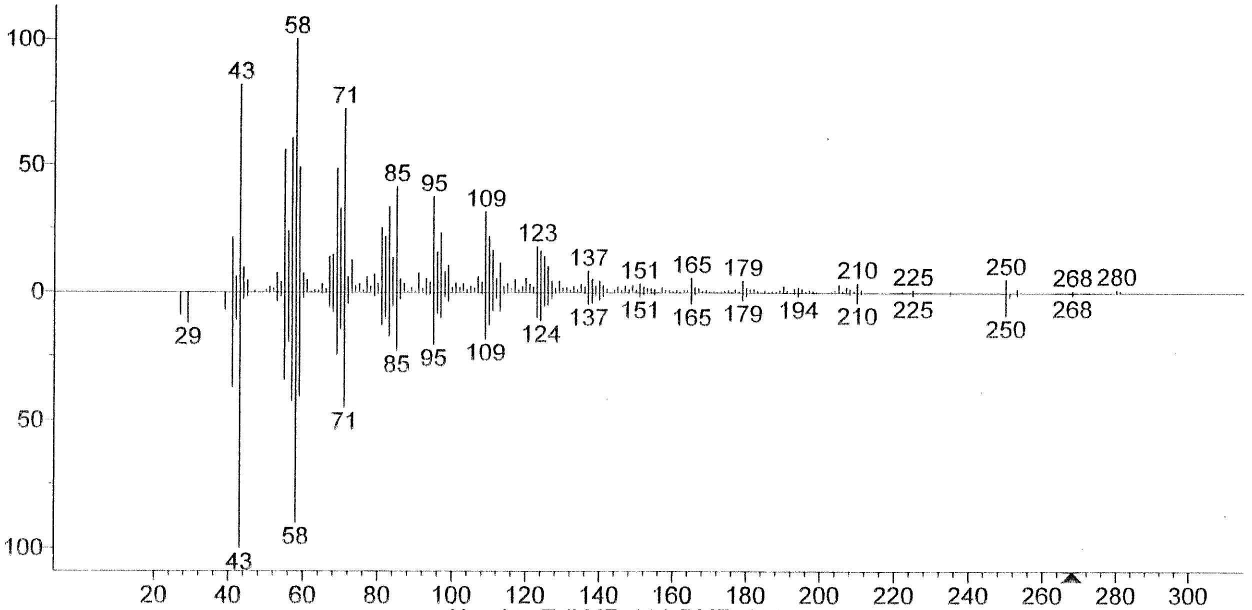

Head to Tail MF=808 RMF=892

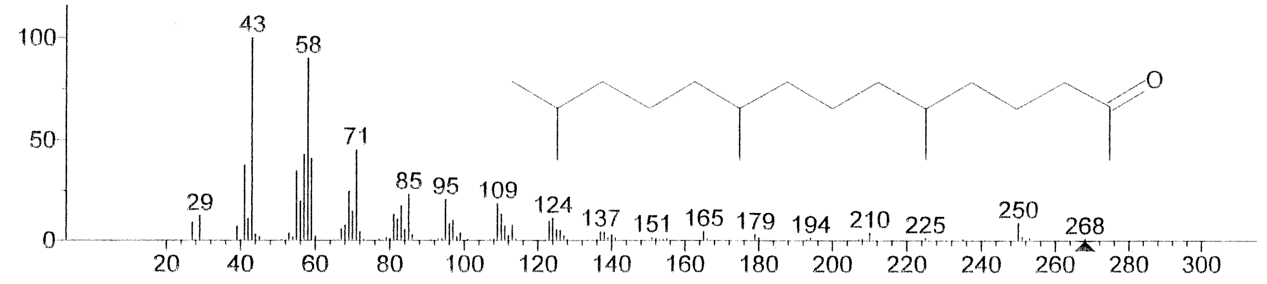

(mainlib) 2-Pentadecanone, 6,10,14-trimethyl-

85

Name: 2-Pentadecanone, 6,10,14-trimethyl-
Formula: $C_{18}H_{36}O$
MW: 268 CAS#: 502-69-2 NIST#: 12976 ID#: 7533 DB: mainlib
Other DBs: TSCA, EINECS
Contributor: STC 0011
10 largest peaks:
43 999 | 58 898 | 71 450 | 57 426 | 59 407 | 41 373 | 55 345 | 69 245 | 85 229 | 95 203 |
Synonyms:
1.Hexahydrofarnesyl acetone
2.6,10,14-Trimethyl-2-pentadecanone

Estimated non-polar retention index (n-alkane scale):
Value: 1754 iu
Confidence interval (Ketones): 57(50%) 246(95%) iu

Retention index.
1. Value: 1838 iu
Column Type: Capillary
Column Class: Standard non-polar
Active Phase: SPB-1
Column
Length: 30 m
Carrier Gas: He
Column Diameter: 0.25 mm
Phase Thickness: 0.25 um
Data Type: Linear
RI
Program Type: Ramp
Start T: 50 C
End T: 250 C
Heat Rate: 5 K/min
Start Time: 3 min
End Time: 15
min
Source: Blagojevic, P.; Radulovic, N.; Palic, R.; Stojanovic, G., Chemical composition of the essential oils of Serbian wild-growing Srtemisia absinthium and Artemisia vulgaris, J. Agric. Food Chem., 54, 2006, 4780-4789.

2.
Value: 1858 iu
Column Type: Capillary
Column Class: Standard non-polar
Active Phase: DB-1
Column Length:
30 m
Carrier Gas: He
Column Diameter: 0.25 mm
Phase Thickness: 0.25 um
Data Type: Linear RI
Program
Type: Ramp
Start T: 60 C
End T: 240 C
Heat Rate: 4 K/min
Source: Rezazadeh, S.; Hamedani, M.P.;

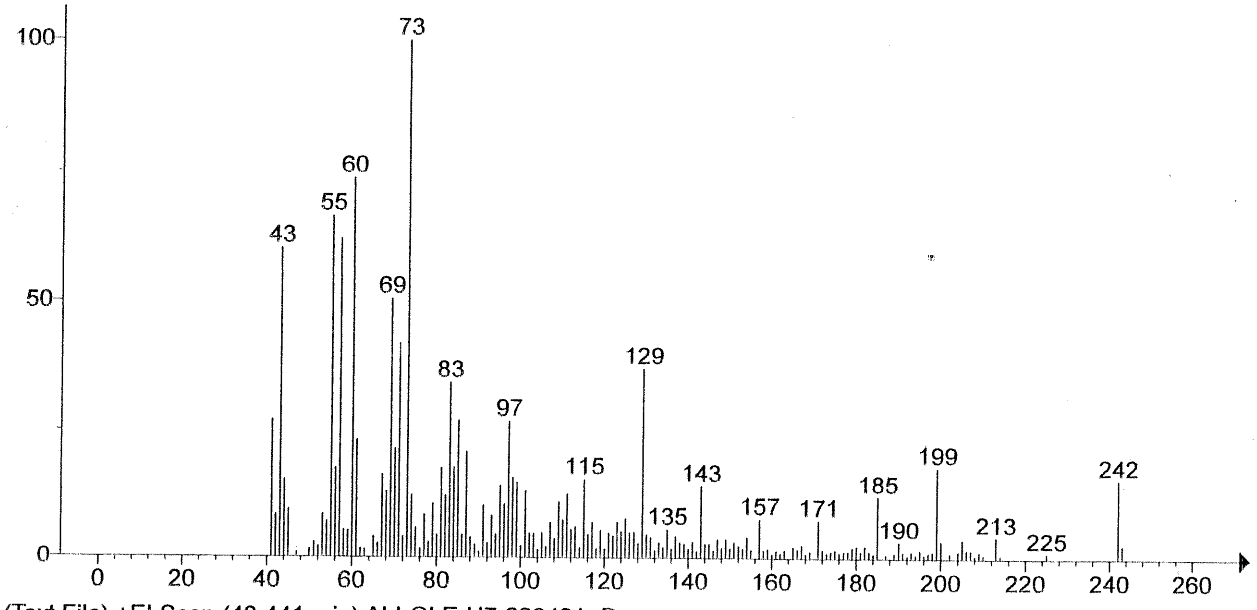

(Text File) +EI Scan (43.441 min) ALI-OLE-H7-220421-.D

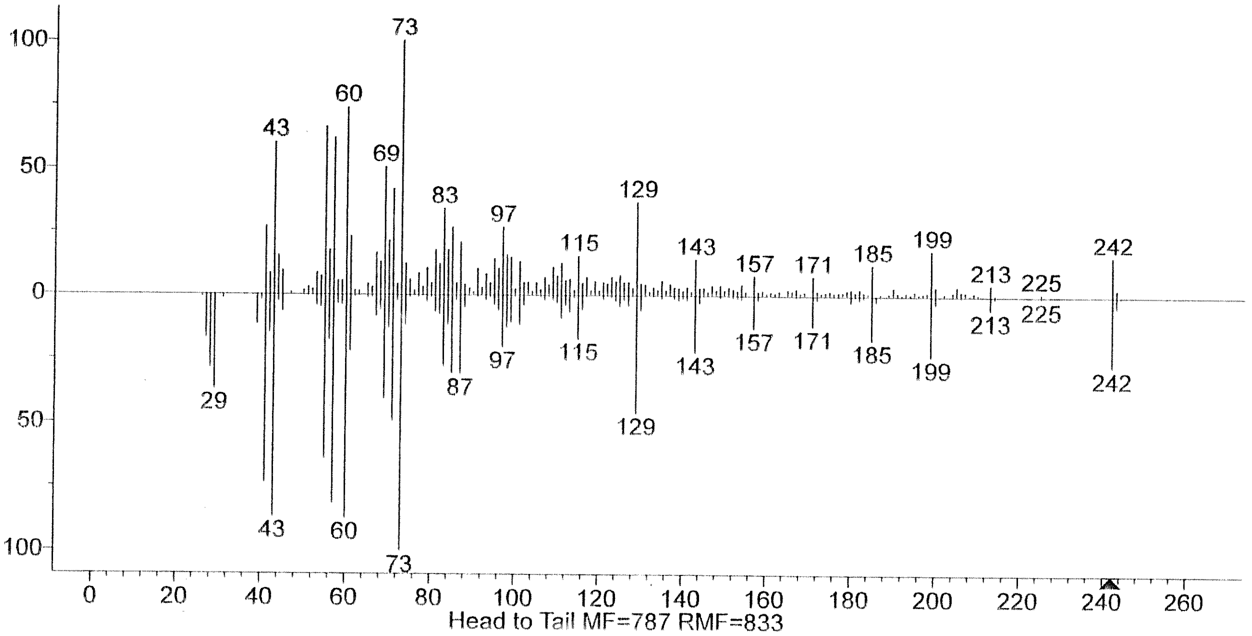

Head to Tail MF=787 RMF=833

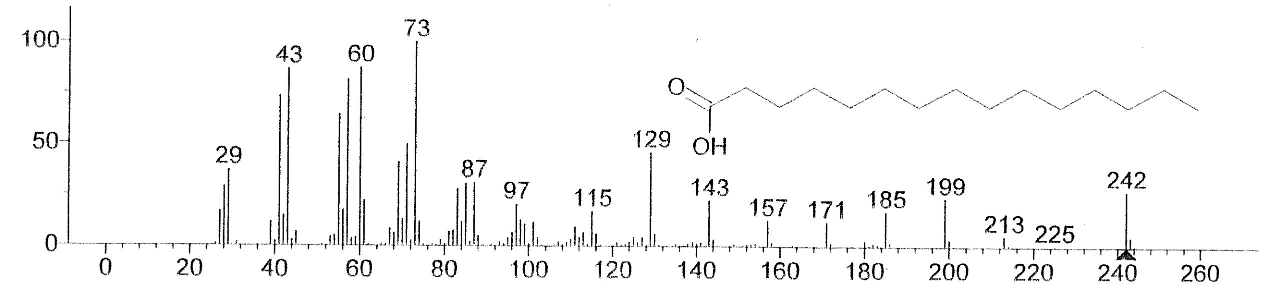

(replib) Pentadecanoic acid

87

Name: Pentadecanoic acid
Formula: $C_{15}H_{30}O_2$
MW: 242 CAS#: 1002-84-2 NIST#: 221146 ID#: 8469 DB: replib
Other DBs: Fine, TSCA, RTECS, HODOC, NIH, EINECS, IRDB
Contributor: Chemical Concepts
10 largest peaks:
73 999 | 60 871 | 43 865 | 57 814 | 41 734 | 55 641 | 71 493 | 129 457 | 69 405 | 29 367 |
Synonyms:
1.Pentadecylic acid
2.n-Pentadecanoic acid
3.n-Pentadecylic acid

Estimated non-polar retention index (n-alkane scale):
Value: 1869 iu
Confidence interval (Carboxylic acids): 51(50%) 220(95%) iu

Retention index.
1. Value: 1823 iu
Column Type: Capillary
Column Class: Standard non-polar
Active Phase: CP Sil 5 CB
Column
Length: 25 m
Carrier Gas: H2
Column Diameter: 0.25 mm
Data Type: Linear RI
Program Type: Ramp
Start T:
80 C
End T: 270 C
Heat Rate: 10 K/min
Source: Ziegenbein, F.C.; Hanssen, H.-P.; König, W.A., Secondary
metabolites from Ganoderma lucidum and Spongiporus leucomallellus, Phytochemistry, 67, 2006, 202-211.

2.
Value: 1823 iu
Column Type: Capillary
Column Class: Standard non-polar
Active Phase: CP Sil 5 CB
Column
Length: 25 m
Carrier Gas: He
Column Diameter: 0.25 mm
Phase Thickness: 0.4 um
Data Type: Linear
RI
Program Type: Ramp
Start T: 80 C
End T: 270 C
Heat Rate: 10 K/min
Source: Ziegenbein, F.C.; Hanssen,
H.-P.; König, W.A., Chemical constituents of the essential oils of three wood-rotting fungi, Flavour Fragr. J., 21, 2006, 813-816.

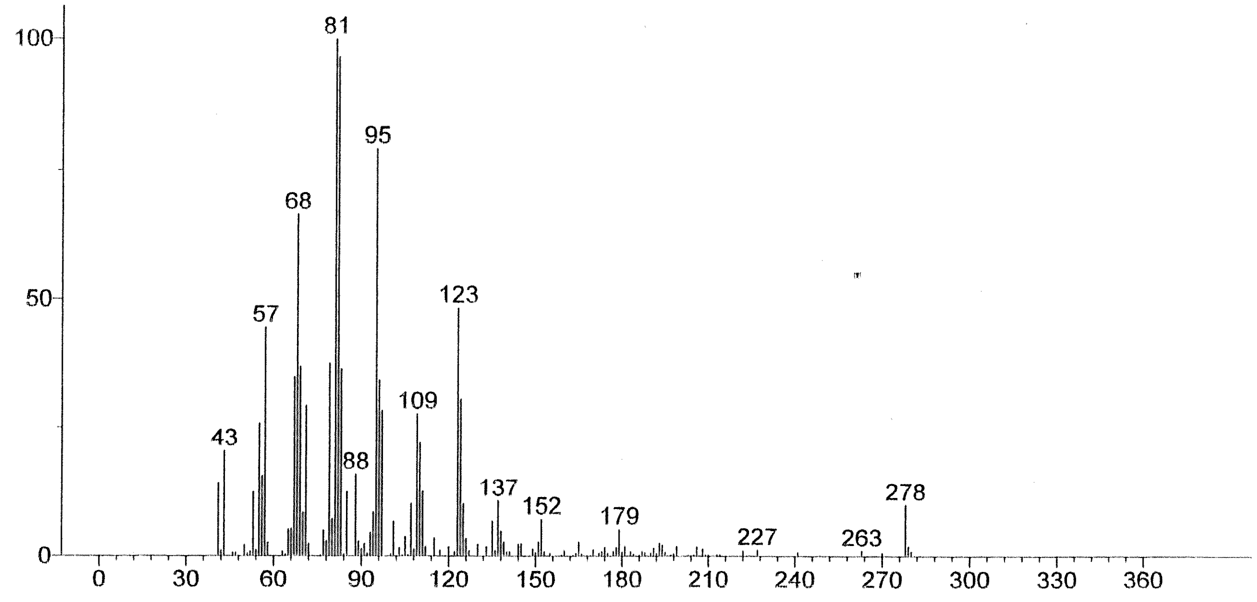

(Text File) +EI Scan (43.680 min) ALI-OLE-H7-220421-.D Subtract

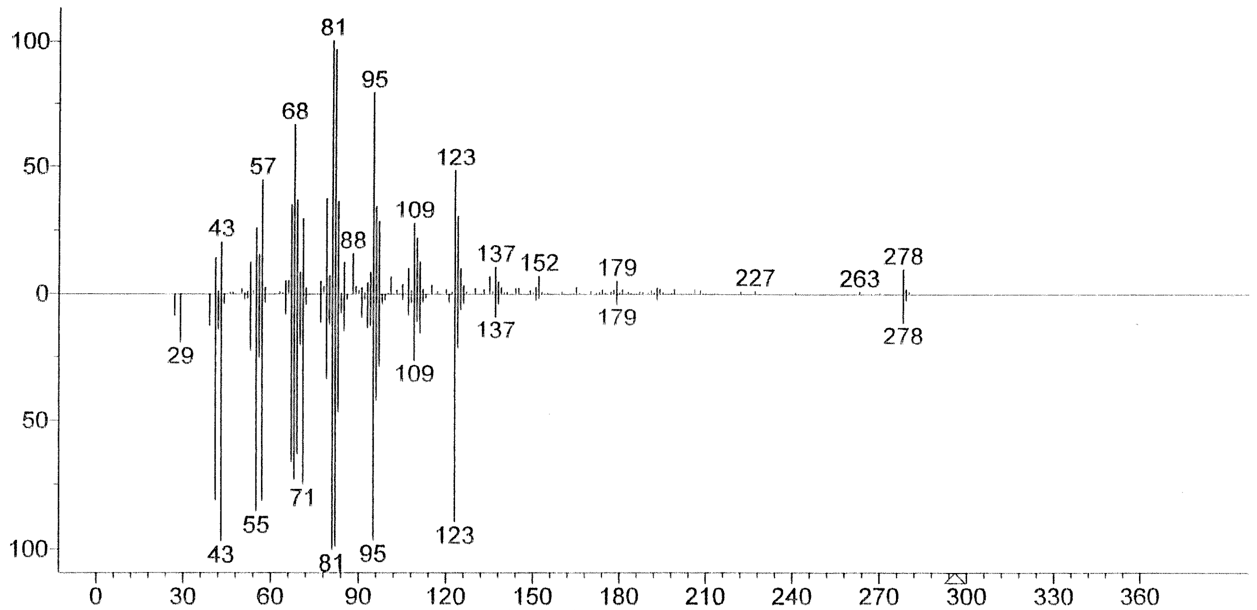

Head to Tail MF=785 RMF=867

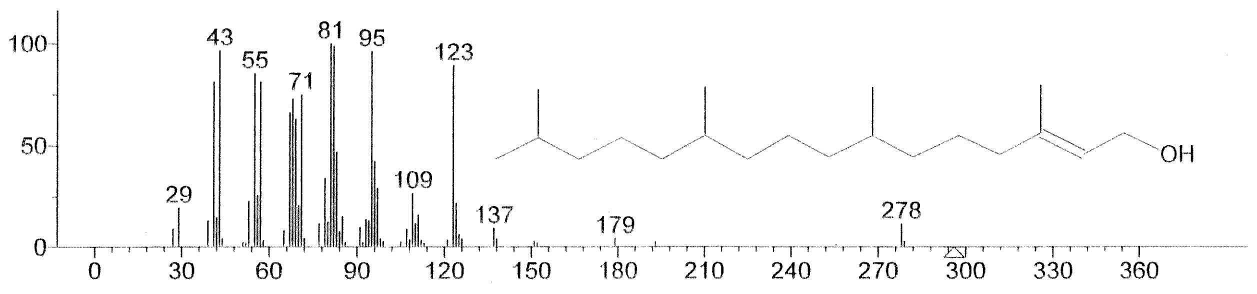

(mainlib) 3,7,11,15-Tetramethyl-2-hexadecen-1-ol

89

Name: 3,7,11,15-Tetramethyl-2-hexadecen-1-ol
Formula: $C_{20}H_{40}O$
MW: 296 CAS#: 102608-53-7 NIST#: 114703 ID#: 43206 DB: mainlib
Other DBs: IRDB
Contributor: NIST Mass Spectrometry Data Center, 1990.
10 largest peaks:
81 999 | 82 986 | 43 965 | 95 962 | 123 892 | 55 852 | 41 811 | 57 811 | 71 748 | 68 728 |
Synonyms:
1.(2E)-3,7,11,15-Tetramethyl-2-hexadecen-1-ol #

Estimated non-polar retention index (n-alkane scale):
Value: 2045 iu
Confidence interval (Alcohols): 41(50%) 176(95%) iu

Retention index.
1. Value: 2119.33 iu
Column Type: Capillary
Column Class: Semi-standard non-polar
Active Phase: SE
-54
Column Length: 25 m
Column Diameter: 0.31 mm
Data Type: Linear RI
Program Type: Ramp
Start T: 35
C
End T: 230 C
Heat Rate: 4 K/min
Start Time: 3 min
End Time: 10 min
Source: Yin, W.; Xiu, Z.; Aijin, H.,
Analysis of the volatile components in trogopterorum feces by capillary gas chromatography and gas chromatography/mass spectrometry, Fenxi Huaxue, 29(2), 2001, 195-198.

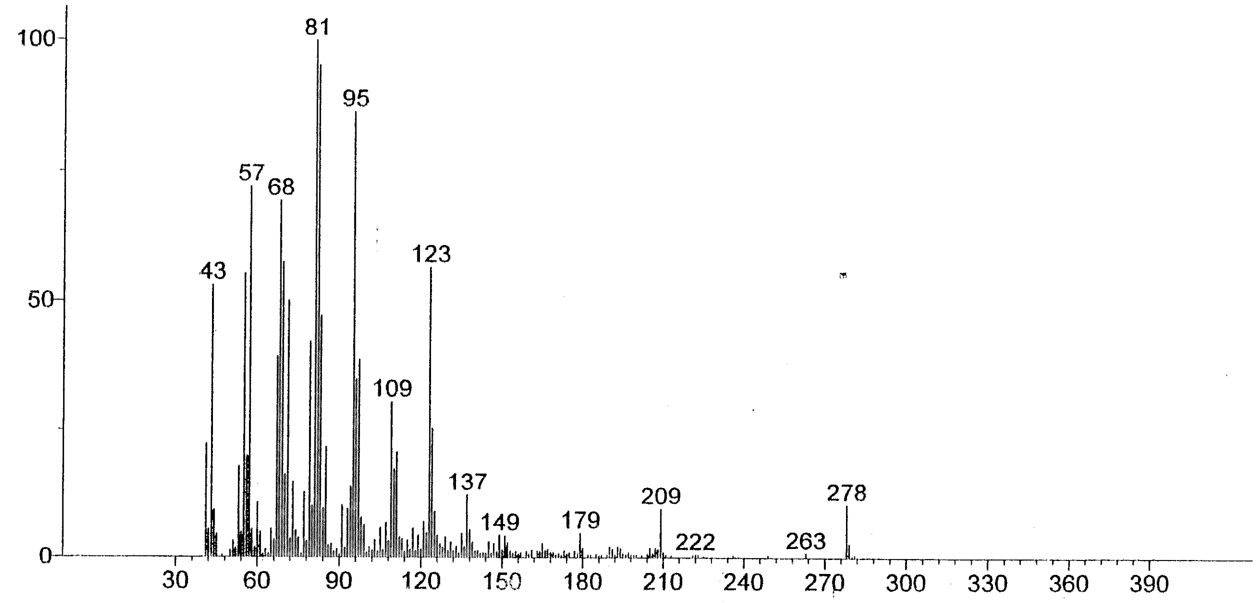

(Text File) +EI Scan (44.382 min) ALI-OLE-H7-220421-.D

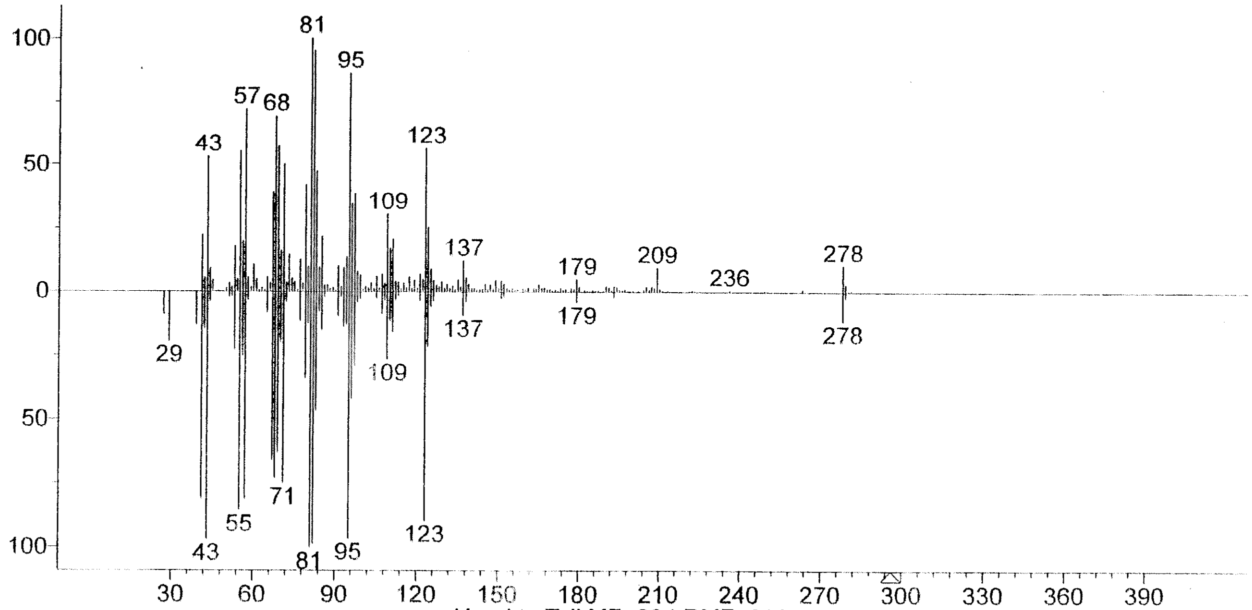

Head to Tail MF=824 RMF=938

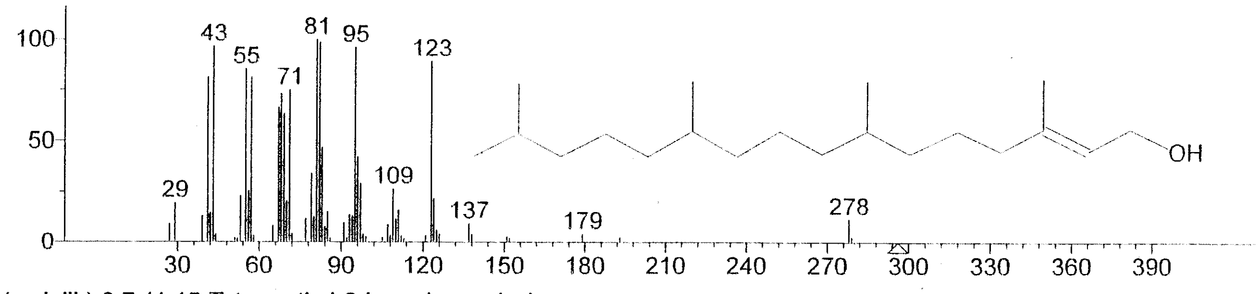

(mainlib) 3,7,11,15-Tetramethyl-2-hexadecen-1-ol

Name: 3,7,11,15-Tetramethyl-2-hexadecen-1-ol
Formula: $C_{20}H_{40}O$
MW: 296 CAS#: 102608-53-7 NIST#: 114703 ID#: 43206 DB: mainlib
Other DBs: IRDB
Contributor: NIST Mass Spectrometry Data Center, 1990.
10 largest peaks:
81 999 | 82 986 | 43 965 | 95 962 | 123 892 | 55 852 | 41 811 | 57 811 | 71 748 | 68 728 |
Synonyms:
1.(2E)-3,7,11,15-Tetramethyl-2-hexadecen-1-ol #

Estimated non-polar retention index (n-alkane scale):
Value: 2045 iu
Confidence interval (Alcohols): 41(50%) 176(95%) iu

Retention index.
1. Value: 2119.33 iu
Column Type: Capillary
Column Class: Semi-standard non-polar
Active Phase: SE
-54
Column Length: 25 m
Column Diameter: 0.31 mm
Data Type: Linear RI
Program Type: Ramp
Start T: 35
C
End T: 230 C
Heat Rate: 4 K/min
Start Time: 3 min
End Time: 10 min
Source: Yin, W.; Xiu, Z.; Aijin, H.,
Analysis of the volatile components in trogopterorum feces by capillary gas chromatography and gas chromatography/mass spectrometry, Fenxi Huaxue, 29(2), 2001, 195-198.

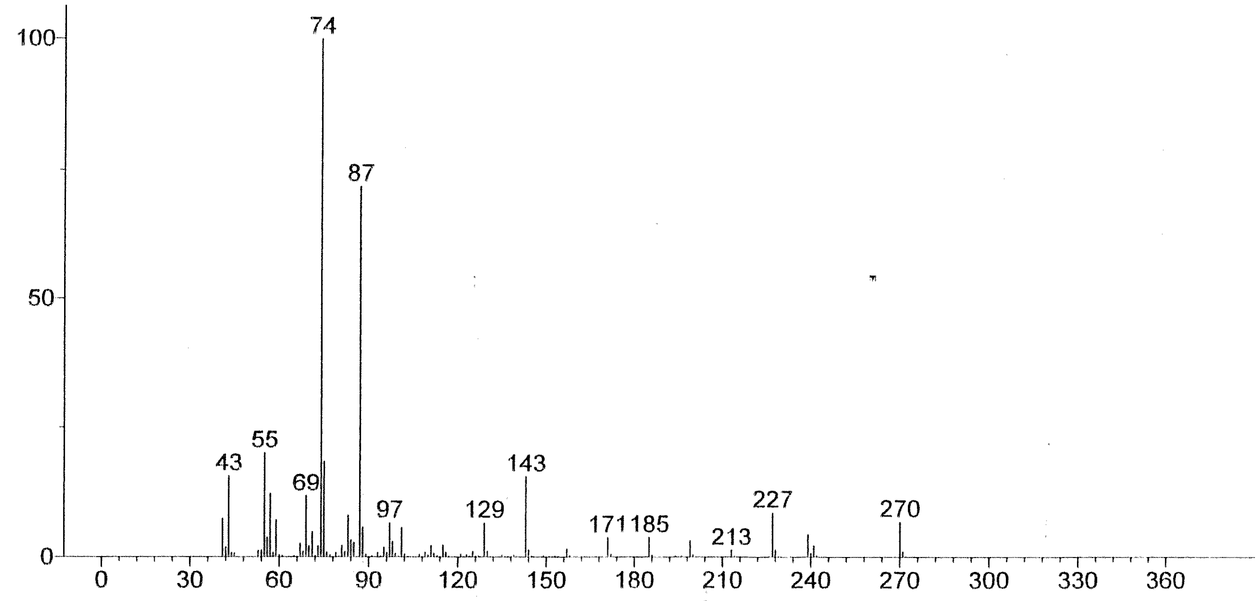

(Text File) +EI Scan (46.178 min) ALI-OLE-H7-220421-.D

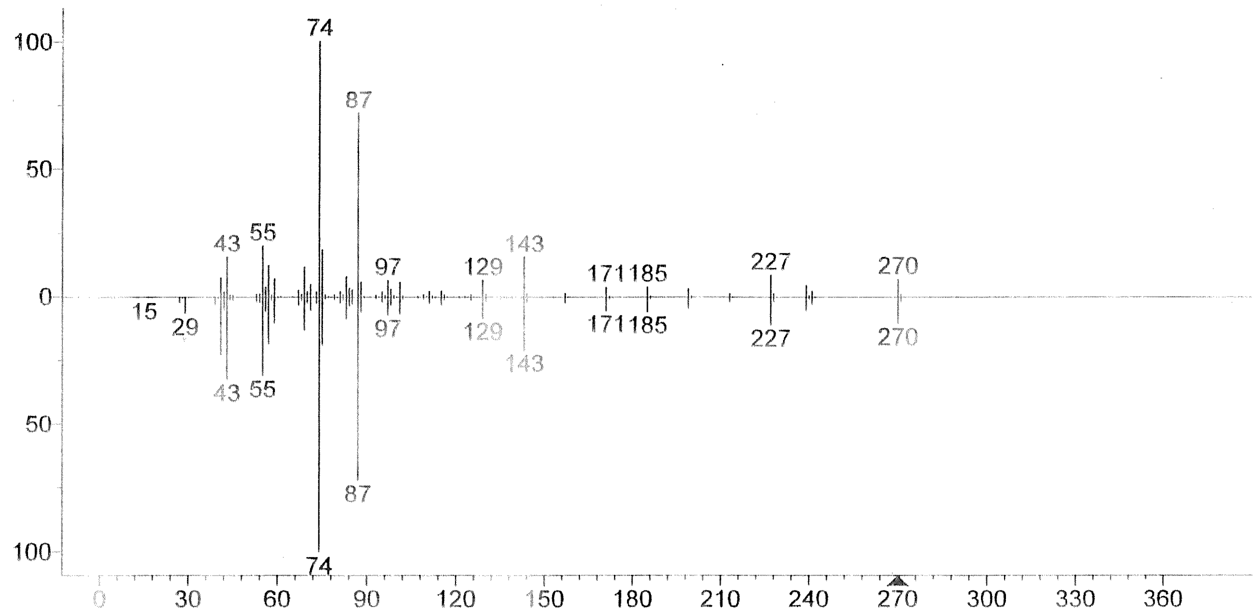

Head to Tail MF=945 RMF=945

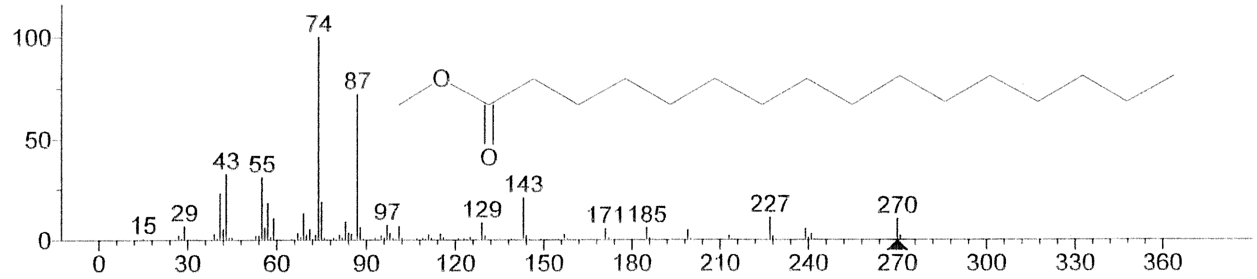

(mainlib) Hexadecanoic acid, methyl ester

43

Name: Hexadecanoic acid, methyl ester
Formula: $C_{17}H_{34}O_2$
MW: 270 CAS#: 112-39-0 NIST#: 333716 ID#: 38248 DB: mainlib
Other DBs: Fine, TSCA, EPA, HODOC, NIH, EINECS
Contributor: NIST Mass Spectrometry Data Center
10 largest peaks:
74 999 | 87 720 | 43 325 | 55 310 | 41 228 | 143 208 | 75 188 | 57 183 | 69 132 | 227 110 |
Synonyms:
1.Palmitic acid, methyl ester
2.n-Hexadecanoic acid methyl ester
3.Metholene 2216
4.Methyl hexadecanoate
5.Methyl n-hexadecanoate
6.Methyl palmitate
7.Uniphat A60
8.Emery 2216
9.Radia 7120

Estimated non-polar retention index (n-alkane scale):
Value: 1878 iu
Confidence interval (Esters): 47(50%) 201(95%) iu

Retention index.
1. Value: 1908 iu
Column Type: Capillary
Column Class: Standard non-polar
Active Phase: SPB-1
Column
Length: 30 m
Carrier Gas: He
Column Diameter: 0.25 mm
Phase Thickness: 0.25 um
Data Type: Linear
RI
Program Type: Ramp
Start T: 50 C
End T: 250 C
Heat Rate: 5 K/min
Start Time: 3 min
End Time: 15
min
Source: Blagojevic, P.; Radulovic, N.; Palic, R.; Stojanovic, G., Chemical composition of the essential oils of Serbian wild-growing Srtemisia absinthium and Artemisia vulgaris, J. Agric. Food Chem., 54, 2006, 4780-4789.

2.
Value: 1909 iu
Column Type: Capillary
Column Class: Standard non-polar
Active Phase: SPB-1
Column Length:
30 m
Carrier Gas: He
Column Diameter: 0.25 mm
Phase Thickness: 0.25 um

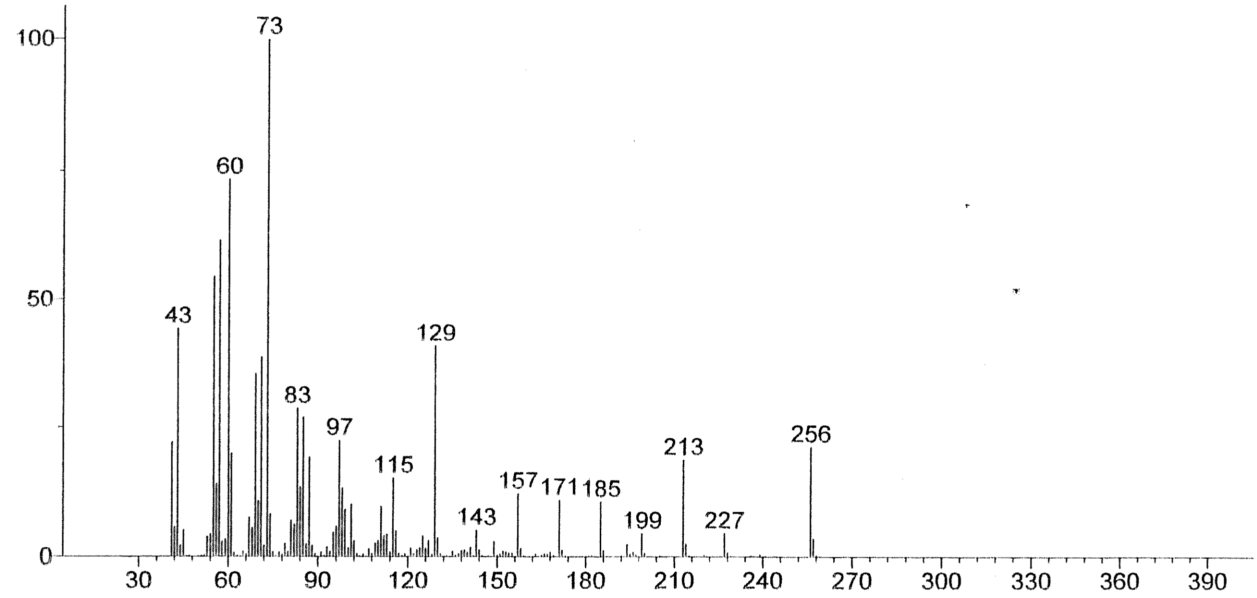

(Text File) +EI Scan (48.153 min) ALI-OLE-H7-220421-.D

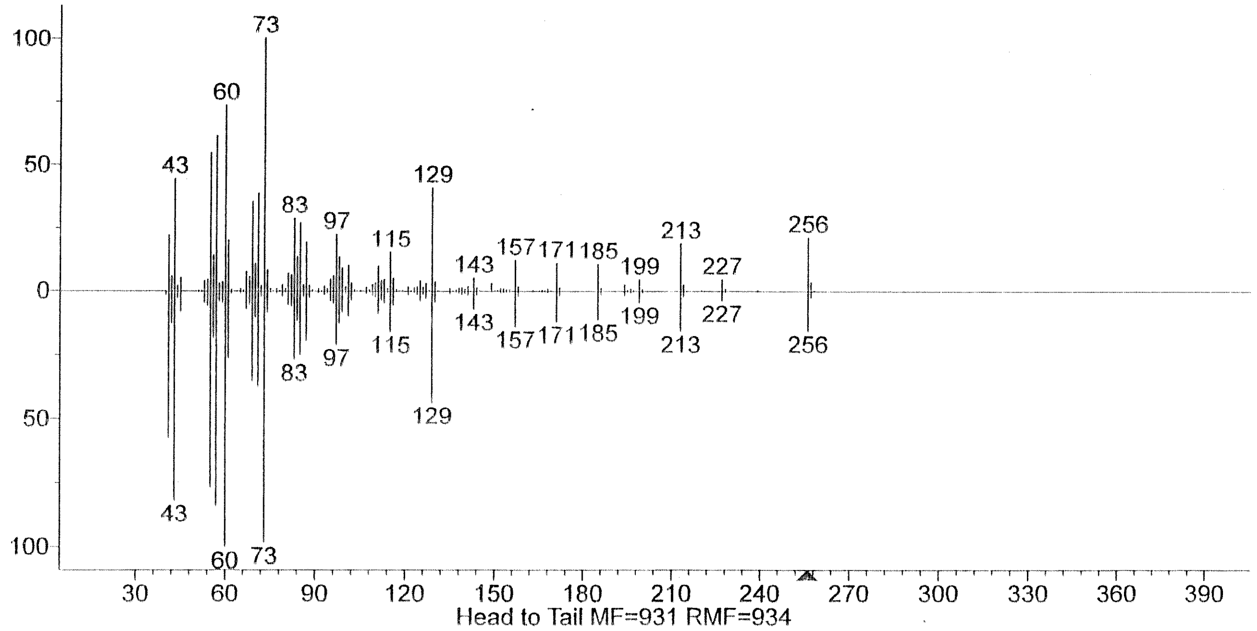

Head to Tail MF=931 RMF=934

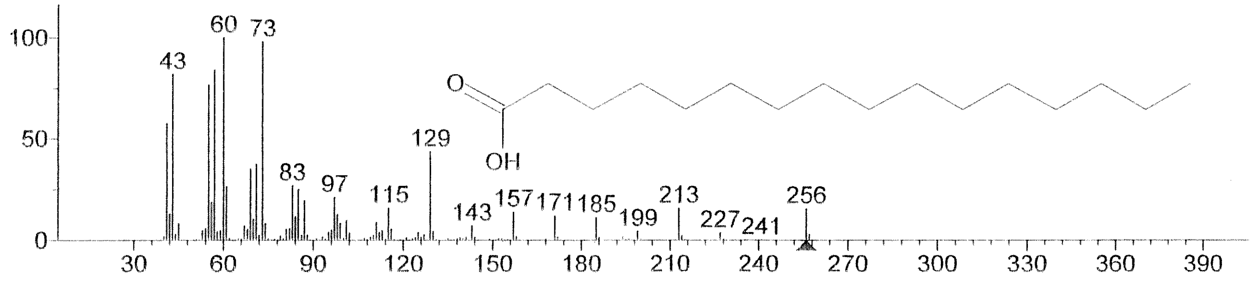

(replib) n-Hexadecanoic acid

95

Name: n-Hexadecanoic acid
Formula: $C_{16}H_{32}O_2$
MW: 256 CAS#: 57-10-3 NIST#: 335494 ID#: 6723 DB: replib
Other DBs: Fine, TSCA, RTECS, EPA, HODOC, NIH, EINECS, IRDB
Contributor: Drug Lab
10 largest peaks:
60 999 | 73 980 | 57 840 | 43 817 | 55 767 | 41 574 | 129 435 | 71 373 | 69 351 | 83 267 |
Synonyms:
1.Hexadecanoic acid
2.n-Hexadecoic acid
3.Palmitic acid
4.Pentadecanecarboxylic acid
5.1-Pentadecanecarboxylic acid
6.Cetylic acid
7.Emersol 140
8.Emersol 143
9.Hexadecylic acid
10.Hydrofol
11.Hystrene 8016
12.Hystrene 9016
13.Industrene 4516
14.Prifrac 2960
15.Glycon P-45
16.Prifac 2960
17.Univol U332

Estimated non-polar retention index (n-alkane scale):
Value: 1968 iu
Confidence interval (Carboxylic acids): 51(50%) 220(95%) iu

Retention index.
1. Value: 1942 iu
Column Type: Capillary
Column Class: Standard non-polar
Active Phase: RTX-1
Column
Length: 60 m
Carrier Gas: He
Column Diameter: 0.22 mm
Phase Thickness: 0.25 um
Data Type: Linear
RI
Program Type: Ramp
Start T: 60 C
End T: 230 C
Heat Rate: 2 K/min
End Time: 35 min
Source: Paolini, J.;
Muselli, A.; Bernardini, A.-F.; Bighelli, A.; Casanova, J.; Costa, J., Thymol derivatives from essential oil of Doronicum corsicum L., Flavour Fragr. J., 22, 2007, 479-487.

2. Value: 1972 iu
Column Type: Capillary
Column

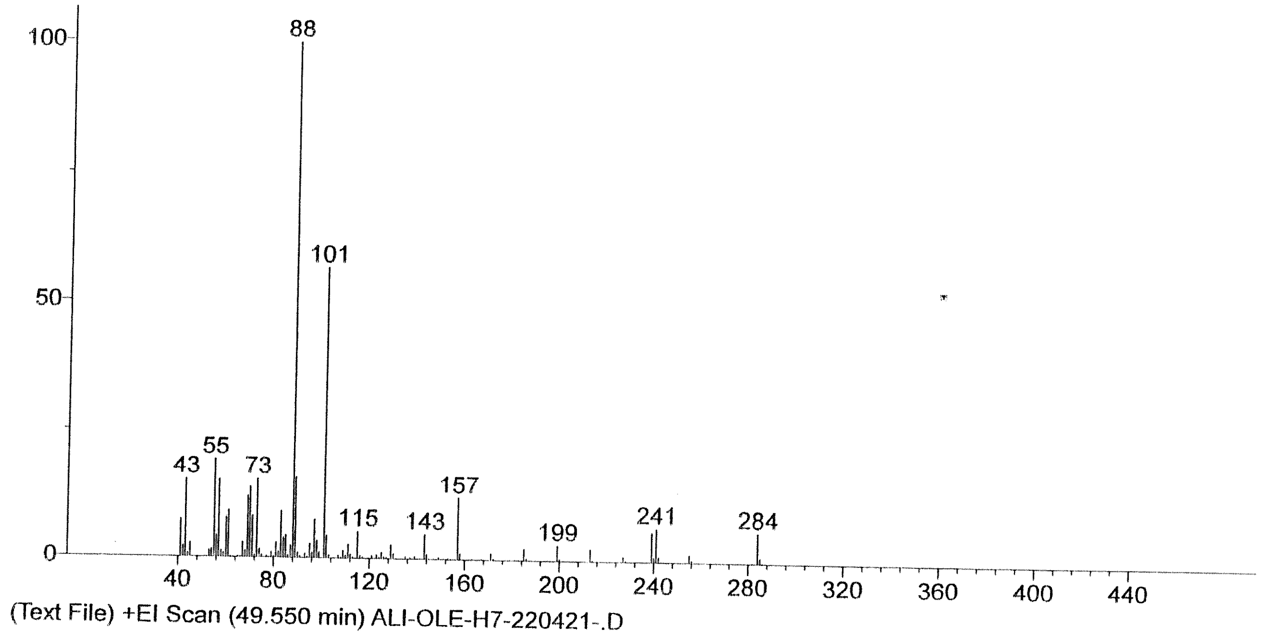

(Text File) +EI Scan (49.550 min) ALI-OLE-H7-220421-.D

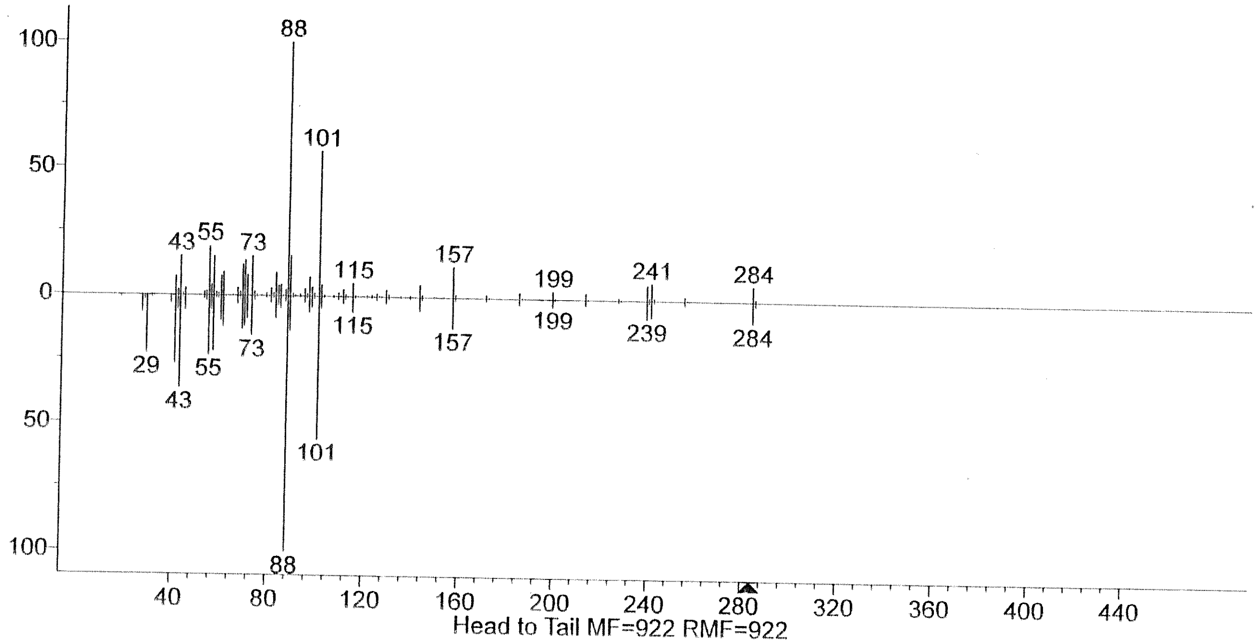

Head to Tail MF=922 RMF=922

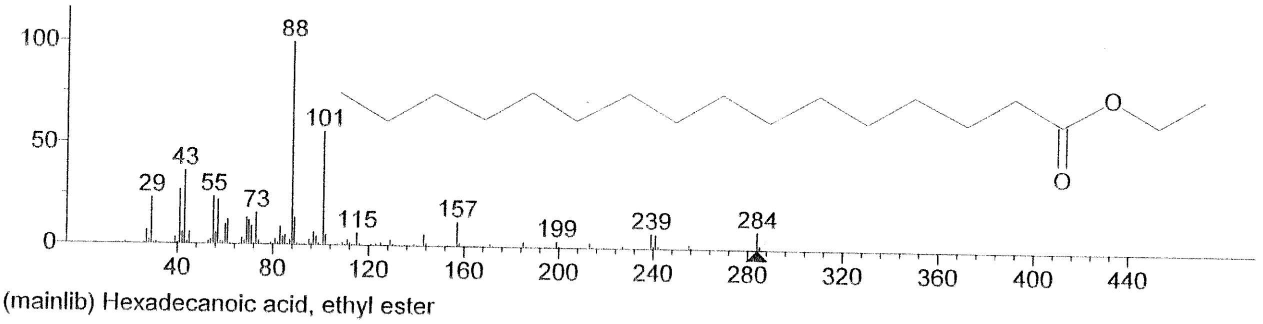

(mainlib) Hexadecanoic acid, ethyl ester

97

Name: Hexadecanoic acid, ethyl ester
Formula: $C_{18}H_{36}O_2$
MW: 284 CAS#: 628-97-7 NIST#: 233204 ID#: 49485 DB: mainlib
Other DBs: Fine, TSCA, EPA, HODOC, NIH, EINECS, IRDB
Contributor: Japan AIST/NIMC Database- Spectrum MS-NW-5396
10 largest peaks:
88 999 | 101 559 | 43 362 | 41 268 | 55 233 | 29 227 | 57 217 | 73 156 | 89 136 | 69 132 |
Synonyms:
1.Palmitic acid, ethyl ester
2.Ethyl hexadecanoate
3.Ethyl palmitate

Estimated non-polar retention index (n-alkane scale):
Value: 1978 iu
Confidence interval (Esters): 47(50%) 201(95%) iu

Retention index.
1. Value: 1968 iu
Column Type: Capillary
Column Class: Standard non-polar
Active Phase: SPB-1
Column
Length: 30 m
Carrier Gas: He
Column Diameter: 0.25 mm
Phase Thickness: 0.25 um
Data Type: Linear
RI
Program Type: Ramp
Start T: 50 C
End T: 250 C
Heat Rate: 5 K/min
Start Time: 3 min
End Time: 15
min
Source: Blagojevic, P.; Radulovic, N.; Palic, R.; Stojanovic, G., Chemical composition of the essential oils of Serbian wild-growing Srtemisia absinthium and Artemisia vulgaris, J. Agric. Food Chem., 54, 2006, 4780-4789.

2.
Value: 1966 iu
Column Type: Capillary
Column Class: Standard non-polar
Active Phase: HP-1
Column Length:
50 m
Carrier Gas: He
Column Diameter: 0.2 mm
Phase Thickness: 0.33 um
Data Type: Linear RI
Program
Type: Ramp
Start T: 60 C
End T: 250 C
Heat Rate: 2 K/min

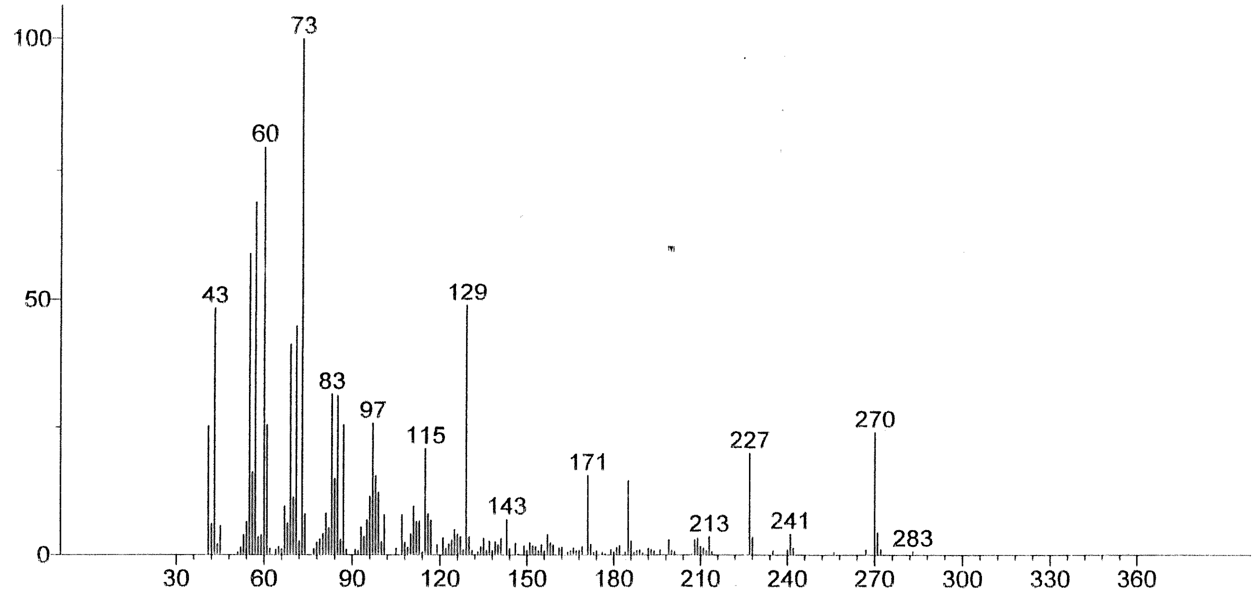

(Text File) +EI Scan (53.616 min) ALI-OLE-H7-220421-.D Subtract

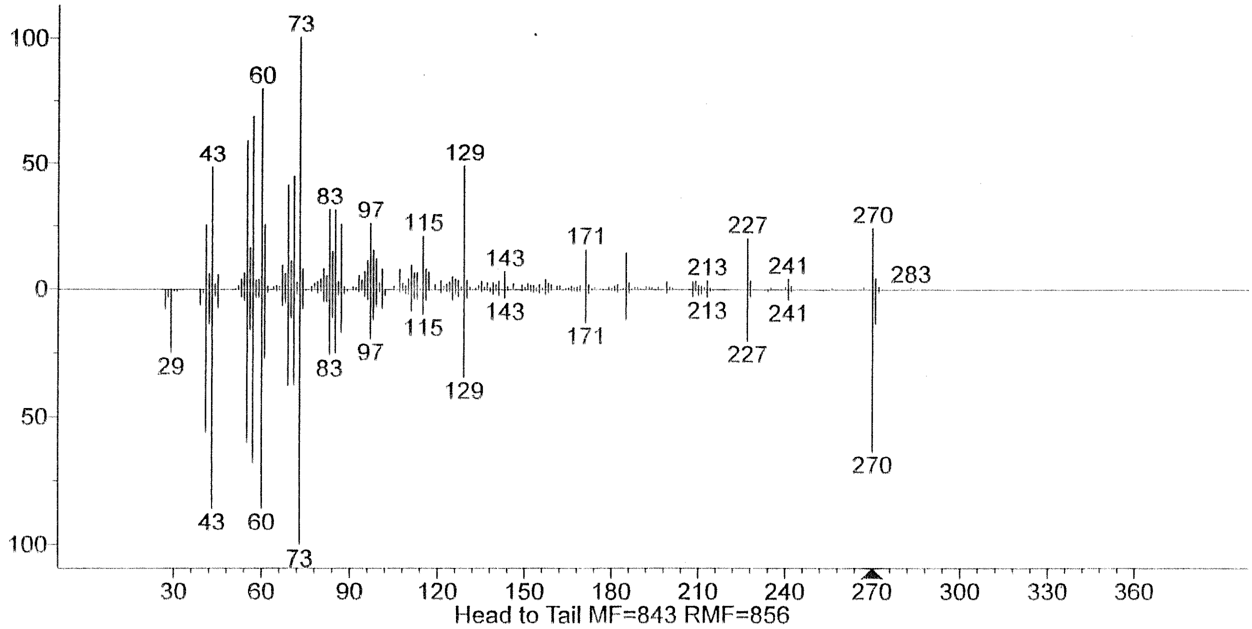

Head to Tail MF=843 RMF=856

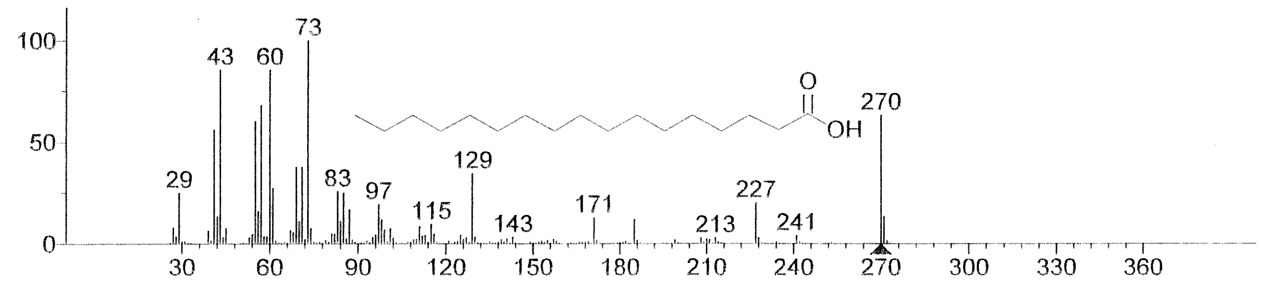

(replib) Heptadecanoic acid

Name: Heptadecanoic acid
Formula: $C_{17}H_{34}O_2$
MW: 270 CAS#: 506-12-7 NIST#: 36447 ID#: 8383 DB: replib
Other DBs: Fine, TSCA, RTECS, HODOC, NIH, EINECS, IRDB
Contributor: R.T.HOLMAN,UNIVERSITY OF MINNESOTA
10 largest peaks:
73 999 | 43 856 | 60 856 | 57 680 | 270 632 | 55 600 | 41 560 | 69 376 | 71 376 | 129 344 |
Synonyms:
1.n-Heptadecanoic acid
2.n-Heptadecoic acid
3.n-Heptadecylic acid
4.Margaric acid
5.Margarinic acid
6.Normal-heptadecanoic acid

Estimated non-polar retention index (n-alkane scale):
Value: 2067 iu
Confidence interval (Carboxylic acids): 51(50%) 220(95%) iu

Retention index.
1. Value: 2038 iu
Column Type: Capillary
Column Class: Standard non-polar
Active Phase: DB-1
Column
Length: 30 m
Column Diameter: 0.2 mm
Phase Thickness: 0.25 um
Data Type: Linear RI
Program Type:
Ramp
Start T: 50 C
End T: 300 C
Heat Rate: 5 K/min
Start Time: 2 min
End Time: 5 min
Source: Johnson, C.
I.; Urso, A.; Geleta, L., Broad spectrum analysis of municipal and industrial effluents discharged into the Peace, Athabasca and Slave river basins: characterization of effluent samples, 1994 - Volume 1 of 2, Northern River Basins Study Project Report No. 121, Norther River Basins Study, Edmonton, Alberta, 1997, 27.

2. Value: 2039
iu
Column Type: Capillary
Column Class: Standard non-polar
Active Phase: DB-1
Column Length: 30
m
Column Diameter: 0.2 mm
Phase Thickness: 0.25 um
Data Type: Linear RI
Program Type: Ramp
Start T: 50
C

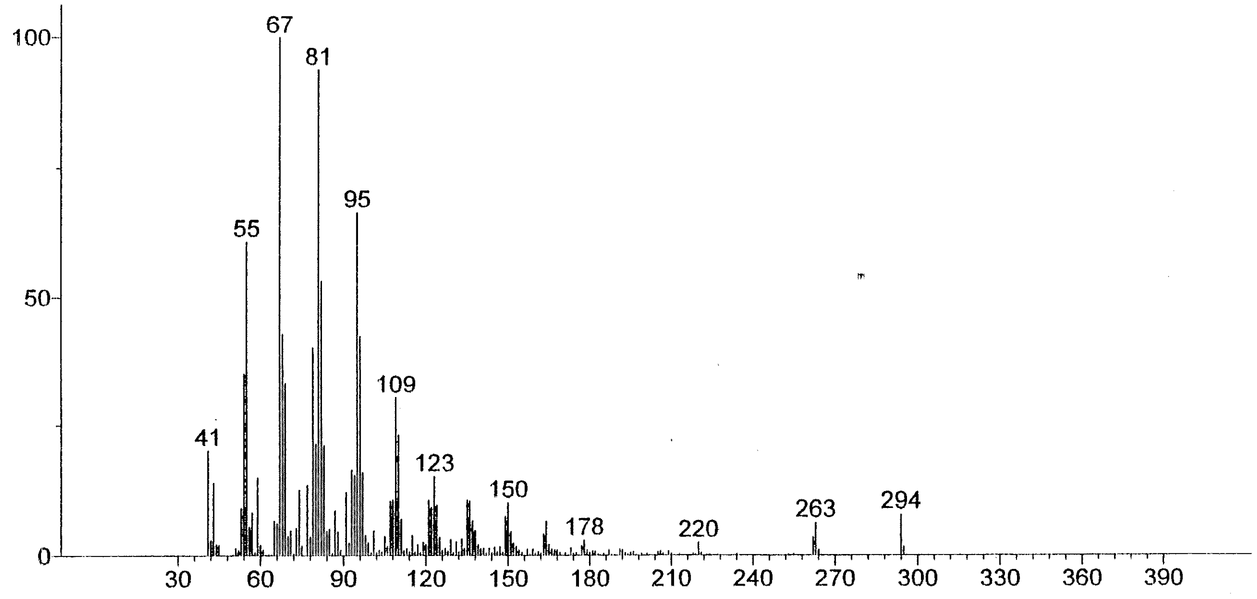

(Text File) +EI Scan (56.186 min) ALI-OLE-H7-220421-.D

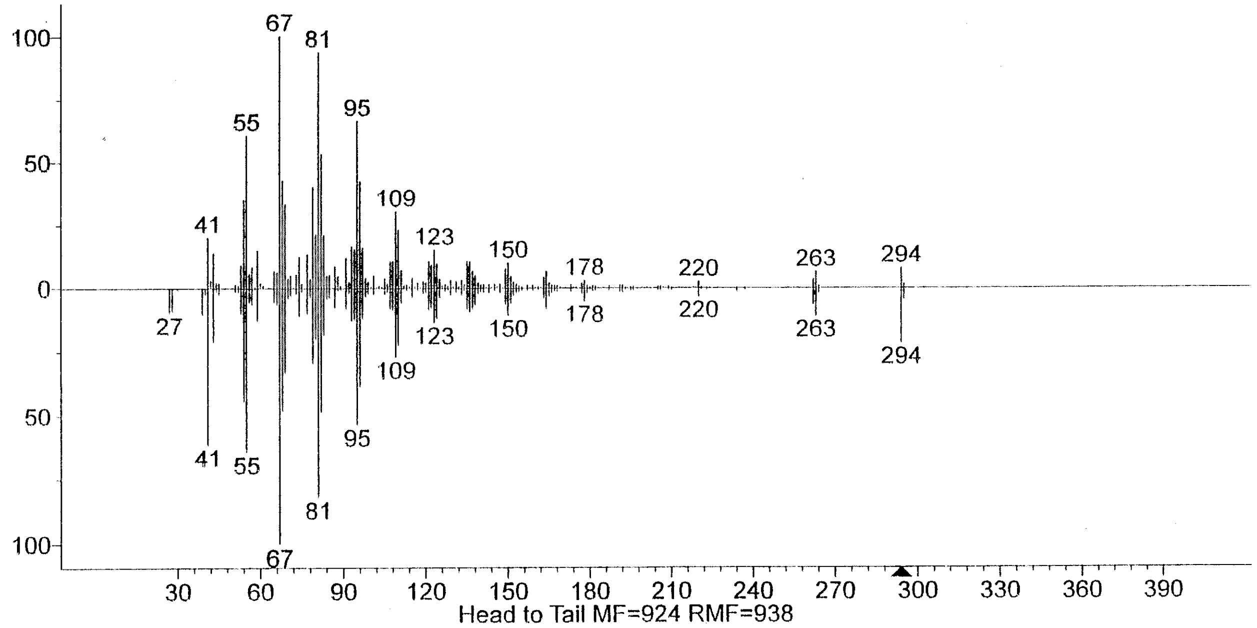

Head to Tail MF=924 RMF=938

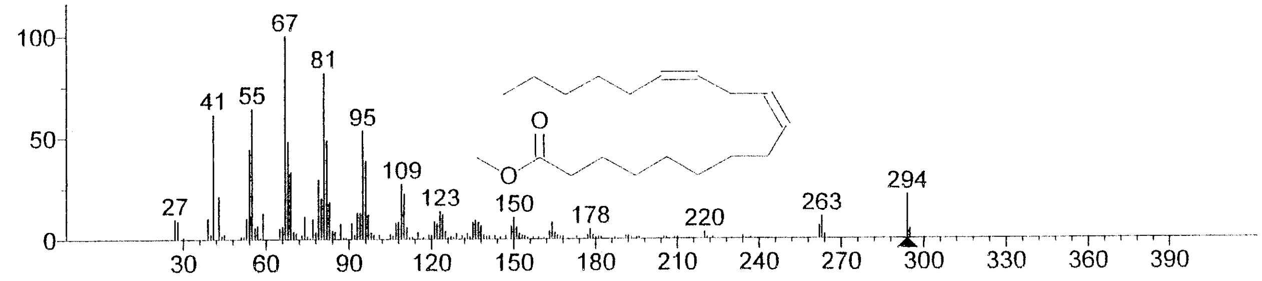

(replib) 9,12-Octadecadienoic acid (Z,Z)-, methyl ester

101

Name: 9,12-Octadecadienoic acid (Z,Z)-, methyl ester
Formula: $C_{19}H_{34}O_2$
MW: 294 CAS#: 112-63-0 NIST#: 79485 ID#: 7214 DB: replib
Other DBs: Fine, TSCA, EPA, HODOC, NIH, EINECS
Contributor: O A MAMER, MCGILL UNIVERSITY, MONTREAL, CANADA
10 largest peaks:
67 999 | 81 817 | 55 641 | 41 613 | 95 534 | 82 486 | 68 480 | 54 442 | 96 385 | 69 330 |
Synonyms:
1.Linoleic acid, methyl ester
2.Methyl cis,cis-9,12-octadecadienoate
3.Methyl linoleate
4.Methyl octadecadienoate
5.Methyl 9-cis,12-cis-octadecadienoate
6.Methyl (9Z,12Z)-9,12-octadecadienoate #

Estimated non-polar retention index (n-alkane scale):
Value: 2093 iu
Confidence interval (Esters): 47(50%) 201(95%) iu

Retention index.
1. Value: 2071 iu
Column Type: Capillary
Column Class: Standard non-polar
Active Phase: SPB-1
Column
Length: 30 m
Carrier Gas: He
Column Diameter: 0.25 mm
Phase Thickness: 0.25 um
Data Type: Linear
RI
Program Type: Ramp
Start T: 50 C
End T: 250 C
Heat Rate: 5 K/min
Start Time: 3 min
End Time: 15
min
Source: Blagojevic, P.; Radulovic, N.; Palic, R.; Stojanovic, G., Chemical composition of the essential oils of Serbian wild-growing Srtemisia absinthium and Artemisia vulgaris, J. Agric. Food Chem., 54, 2006, 4780-4789.

2.
Value: 2087 iu
Column Type: Capillary
Column Class: Standard non-polar
Active Phase: BP-1
Column Length:
30 m
Carrier Gas: N2
Column Diameter: 0.32 mm
Phase Thickness: 0.25 um
Data Type: Linear RI
Program
Type: Ramp

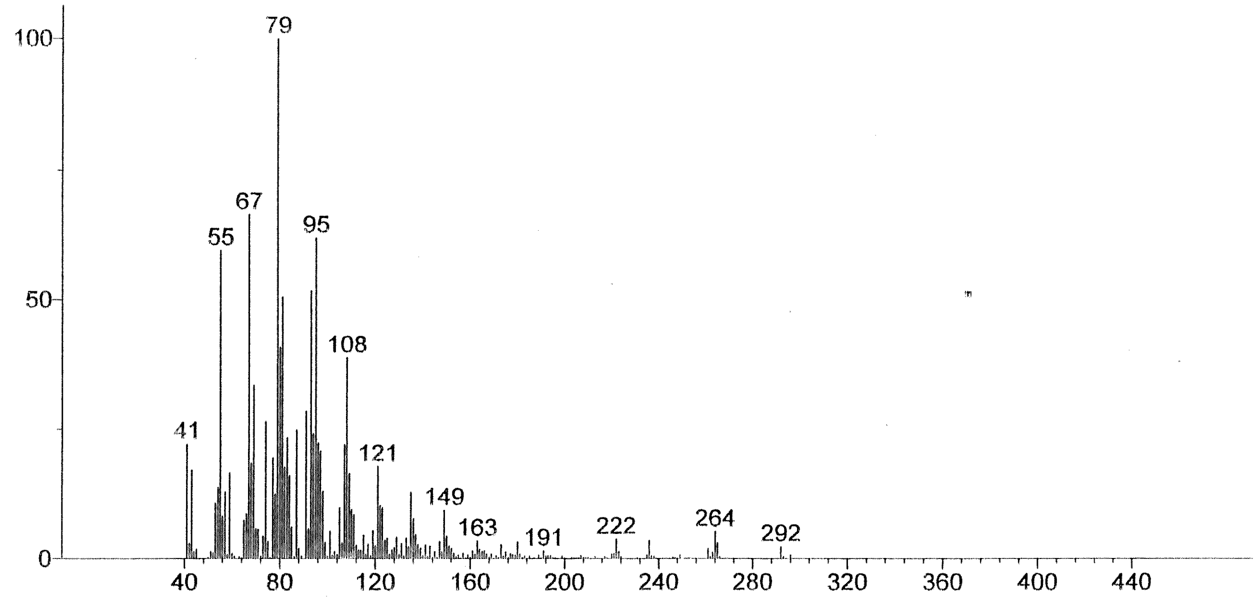

(Text File) +EI Scan (56.774 min) ALI-OLE-H7-220421-.D

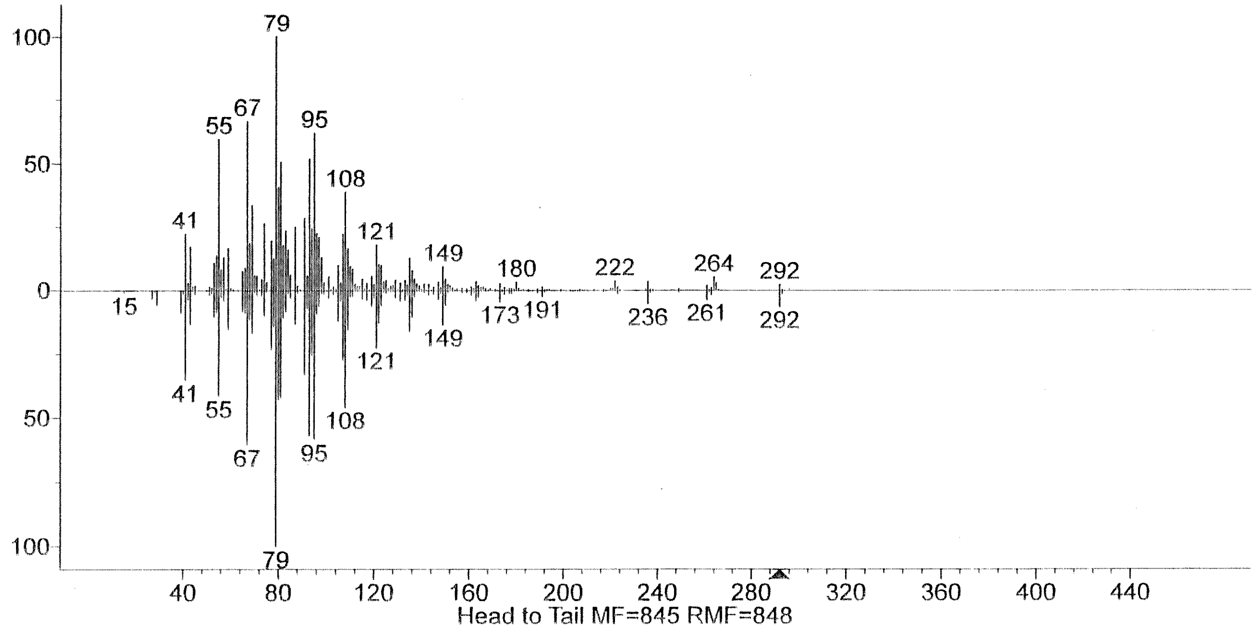

Head to Tail MF=845 RMF=848

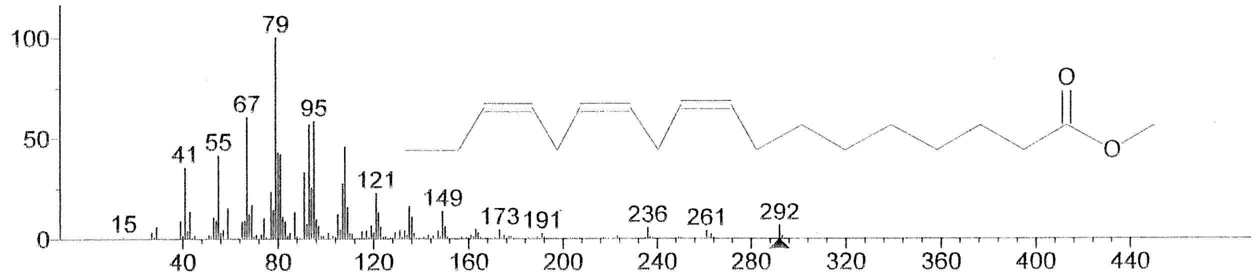

(mainlib) 9,12,15-Octadecatrienoic acid, methyl ester, (Z,Z,Z)-

Name: 9,12,15-Octadecatrienoic acid, methyl ester, (Z,Z,Z)-
Formula: $C_{19}H_{32}O_2$
MW: 292 CAS#: 301-00-8 NIST#: 333199 ID#: 41712 DB: mainlib
Other DBs: Fine, TSCA, HODOC, EINECS
Contributor: NIST Mass Spectrometry Data Center
10 largest peaks:
79 999 | 67 603 | 95 583 | 93 567 | 108 457 | 80 430 | 81 422 | 55 414 | 41 354 | 91 332 |
Synonyms:
1.Linolenic acid, methyl ester
2.Methyl all-cis-9,12,15-octadecatrienoate
3.Methyl linolenate
4.Methyl (9Z,12Z,15Z)-9,12,15-octadecatrienoate #

Estimated non-polar retention index (n-alkane scale):
Value: 2101 iu
Confidence interval (Esters): 47(50%) 201(95%) iu

Retention index.
1. Value: 2077 iu
Column Type: Capillary
Column Class: Standard non-polar
Active Phase: SPB-1
Column
Length: 30 m
Carrier Gas: He
Column Diameter: 0.25 mm
Phase Thickness: 0.25 um
Data Type: Linear
RI
Program Type: Ramp
Start T: 50 C
End T: 250 C
Heat Rate: 5 K/min
Start Time: 3 min
End Time: 15
min
Source: Radulovic, N.; Lazarevic, J.; Stojanovic, G.; Palic, R., Chemotaxonomically significant 2-ethyl substituted fatty acids from Stachys milanii Petrovic (Lamiaceae), Biochem. Syst. Ecol., 34, 2006, 341-344.

2.
Value: 2098 iu
Column Type: Capillary
Column Class: Standard non-polar
Active Phase: HP-1
Column Length:
30 m
Carrier Gas: He
Column Diameter: 0.25 mm
Phase Thickness: 0.33 um
Data Type: Linear RI
Program
Type: Ramp
Start T: 40 C
End T: 260 C

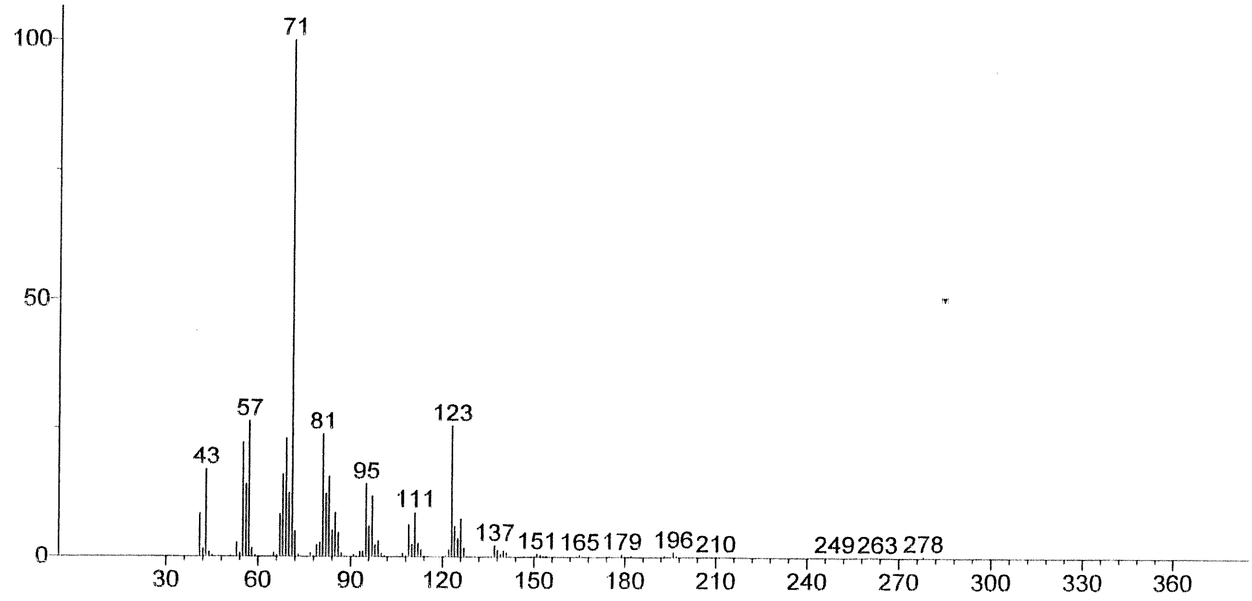

(Text File) +EI Scan (57.725 min) ALI-OLE-H7-220421-.D Subtract

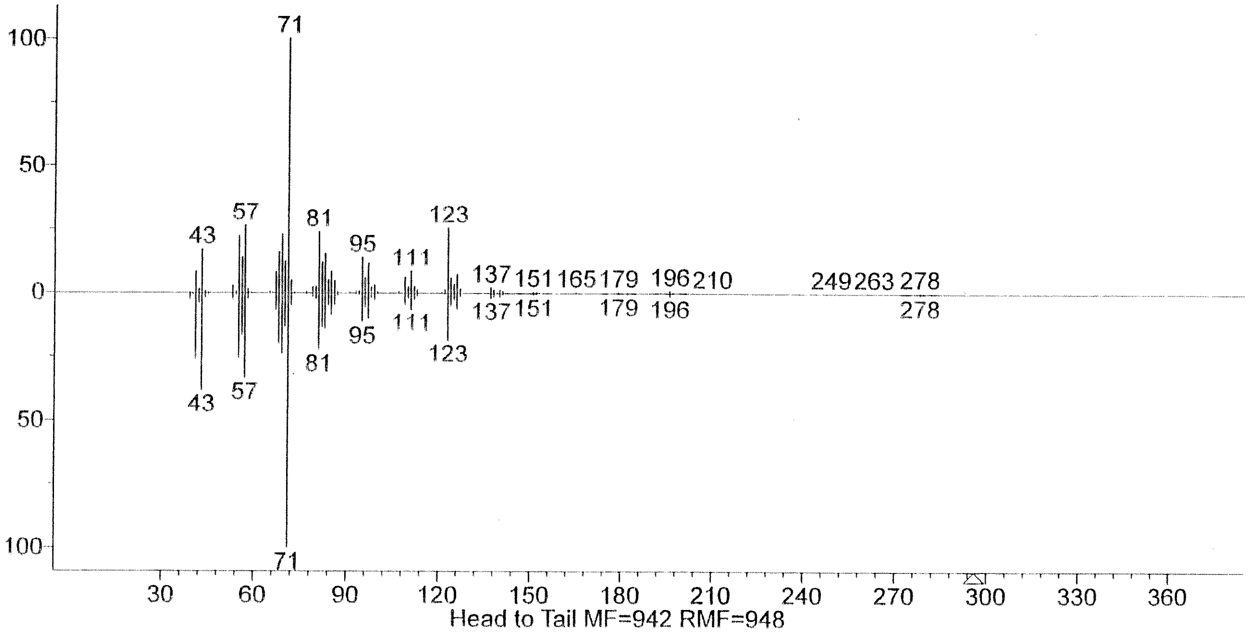

Head to Tail MF=942 RMF=948

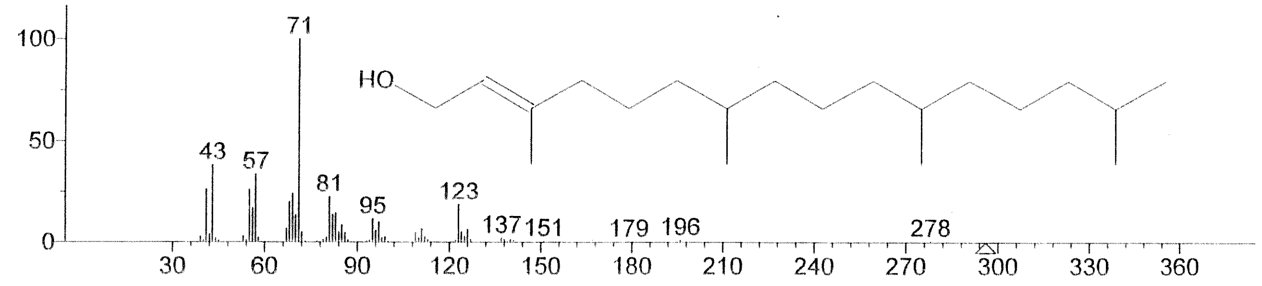

(replib) Phytol

105

Name: Phytol
Formula: $C_{20}H_{40}O$
MW: 296 CAS#: 150-86-7 NIST#: 108727 ID#: 8051 DB: replib
Other DBs: Fine, TSCA, RTECS, HODOC, EINECS
Contributor: Philip Morris R&D
10 largest peaks:
71 999 | 43 381 | 57 334 | 41 260 | 55 259 | 69 239 | 81 223 | 68 199 | 123 184 | 56 169 |
Synonyms:
1.2-Hexadecen-1-ol, 3,7,11,15-tetramethyl-, [R-[R*,R*-(E)]]-
2.trans-Phytol
3.3,7,11,15-Tetramethyl-2-hexadecen-1-ol
4.(2E)-3,7,11,15-Tetramethyl-2-hexadecen-1-ol #

Estimated non-polar retention index (n-alkane scale):
Value: 2045 iu
Confidence interval (Alcohols): 41(50%) 176(95%) iu

Retention index.
1. Value: 2104 iu
Column Type: Capillary
Column Class: Standard non-polar
Active Phase: SPB-1
Column
Length: 30 m
Carrier Gas: He
Column Diameter: 0.25 mm
Phase Thickness: 0.25 um
Data Type: Linear
RI
Program Type: Ramp
Start T: 50 C
End T: 250 C
Heat Rate: 5 K/min
Start Time: 3 min
End Time: 15
min
Source: Radulovic, N.; Lazarevic, J.; Stojanovic, G.; Palic, R., Chemotaxonomically significant 2-ethyl substituted fatty acids from Stachys milanii Petrovic (Lamiaceae), Biochem. Syst. Ecol., 34, 2006, 341-344.

2.
Value: 2099 iu
Column Type: Capillary
Column Class: Standard non-polar
Active Phase: RTX-1
Column Length:
60 m
Carrier Gas: He
Column Diameter: 0.22 mm
Phase Thickness: 0.25 um
Data Type: Linear RI
Program
Type: Ramp
Start T: 60 C
End T: 230 C

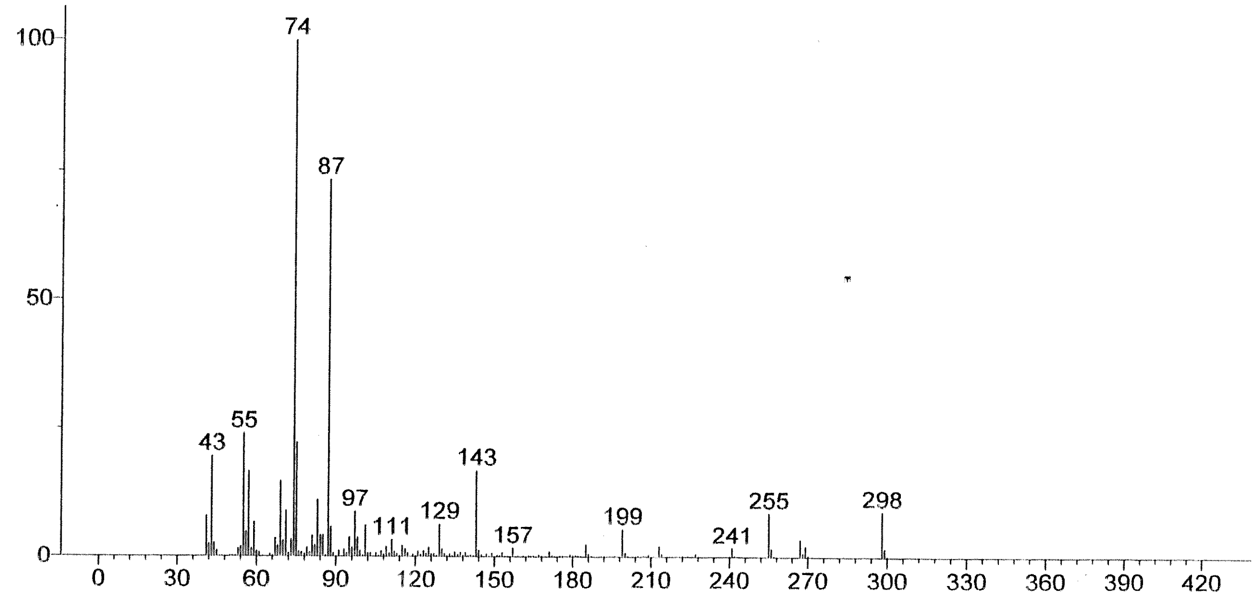

(Text File) +EI Scan (58.716 min) ALI-OLE-H7-220421-.D

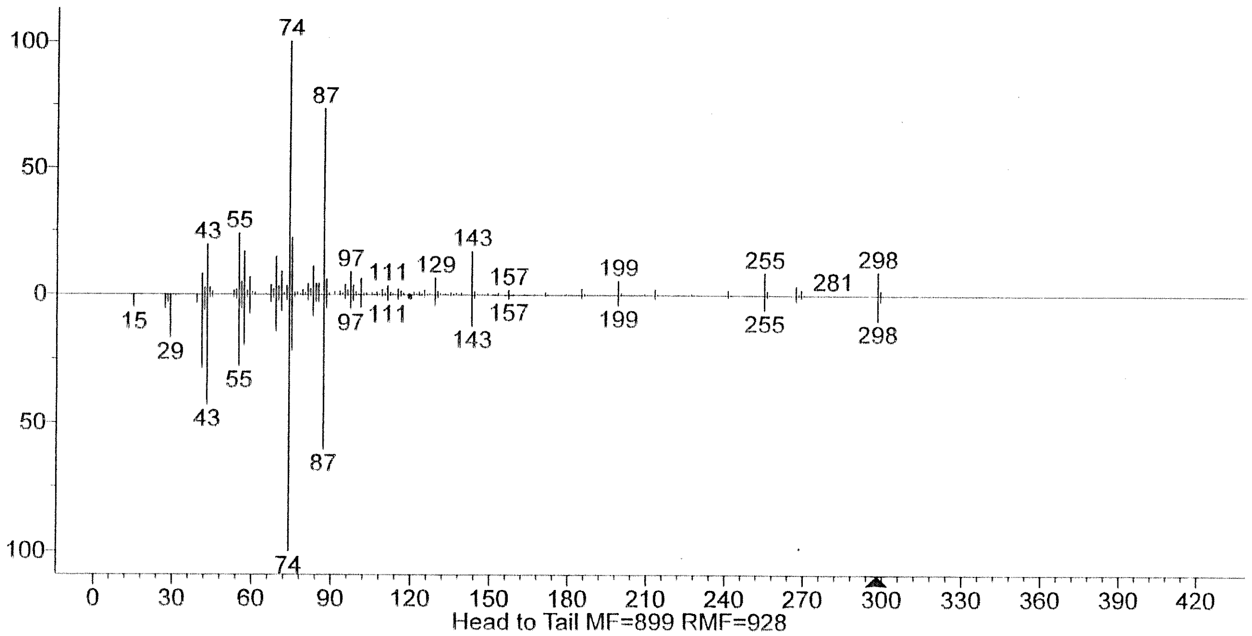

Head to Tail MF=899 RMF=928

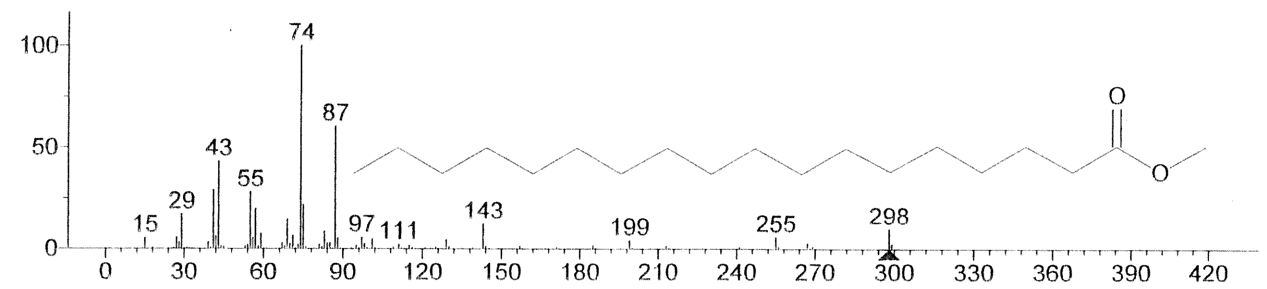

(replib) Octadecanoic acid, methyl ester

Name: Octadecanoic acid, methyl ester
Formula: $C_{19}H_{38}O_2$
MW: 298 CAS#: 112-61-8 NIST#: 79123 ID#: 9086 DB: replib
Other DBs: Fine, TSCA, RTECS, EPA, HODOC, NIH, EINECS, IRDB
Contributor: O A MAMER, MCGILL UNIVERSITY, MONTREAL,
10 largest peaks:
74 999 | 87 604 | 43 430 | 41 289 | 55 278 | 75 218 | 57 197 | 29 169 | 69 143 | 143 121 |
Synonyms:
1.Stearic acid, methyl ester
2.n-Octadecanoic acid, methyl ester
3.Kemester 9718
4.Methyl n-octadecanoate
5.Methyl octadecanoate
6.Methyl stearate
7.Metholene 2218
8.Emery 2218
9.Kemester 9018
10.Methyl ester of octadecanoic acid
11.Kemester 4516
12.Methyl (Z)-9-octadecenoate

Estimated non-polar retention index (n-alkane scale):
Value: 2077 iu
Confidence interval (Esters): 47(50%) 201(95%) iu

Retention index.
1. Value: 2133 iu
Column Type: Capillary
Column Class: Standard non-polar
Active Phase: HP-1
Column
Length: 30 m
Carrier Gas: He
Column Diameter: 0.25 mm
Phase Thickness: 0.33 um
Data Type: Linear
RI
Program Type: Ramp
Start T: 40 C
End T: 260 C
Heat Rate: 2 K/min
Start Time: 5 min
End Time: 20
min
Source: Senatore, F.; Rigano, D.; de Fusco, R.; Bruno, M., Volatile components of Centaurea cineraria L. subsp. umbrosa (lacaita) Pign. and Centaurea napifolia L. (Asteraceae), two species growing wild in Sicily, Flavour Fragr. J., 18, 2003, 248-251.

2. Value: 2111 iu
Column Type: Capillary
Column Class: Standard non
-polar
Active Phase: DB-1
Column Length: 15 m

Name: 9,12,15-Octadecatrienoic acid, (Z,Z,Z)-
Formula: $C_{18}H_{30}O_2$
MW: 278 CAS#: 463-40-1 NIST#: 333201 ID#: 41695 DB: mainlib
Other DBs: Fine, TSCA, HODOC, NIH, EINECS
Contributor: NIST Mass Spectrometry Data Center
10 largest peaks:

79 999	67 624	93 503	95 478	55 463	80 460	41 408	108 391	81 373	91 299

Synonyms:
1.Linolenic acid
2.α-Linolenic acid
3.All-cis-9,12,15-Octadecatrienoic acid
4.cis,cis,cis-9,12,15-Octadecatrienoic acid
5.(Z,Z,Z)-9,12,15-Octadecatrienoic acid
6.Industrene 120

Estimated non-polar retention index (n-alkane scale):
Value: 2191 iu
Confidence interval (Carboxylic acids): 51(50%) 220(95%) iu

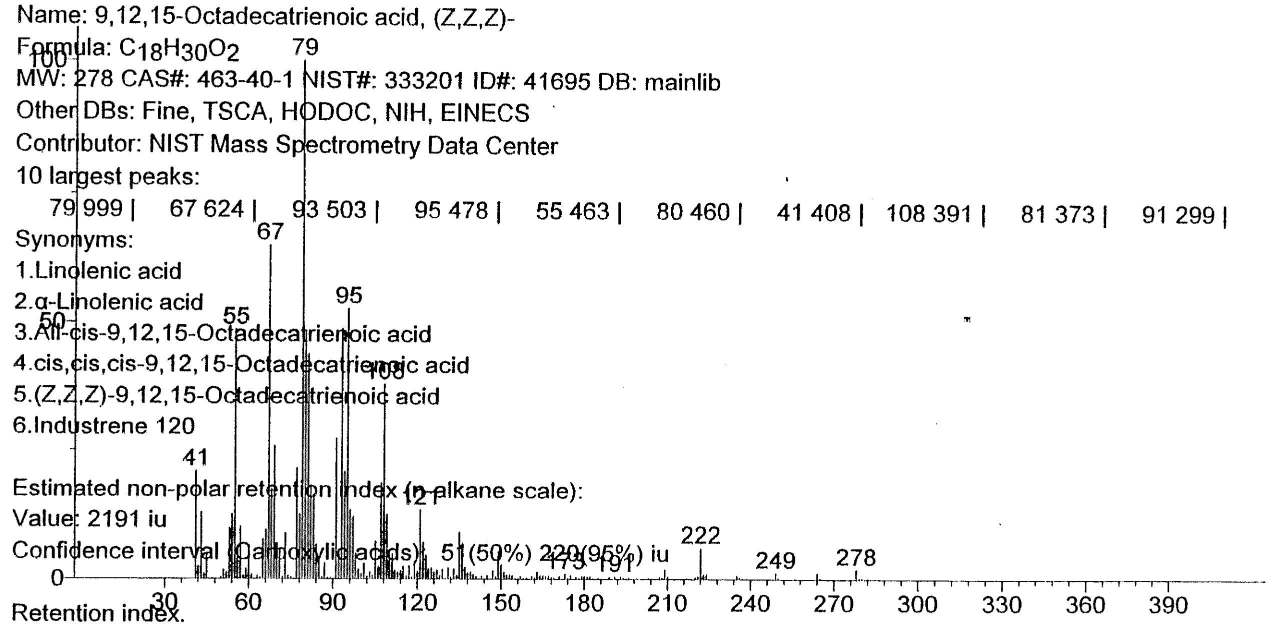

Retention index:
1. Value: 2102 iu
(Text File) +EI Scan (60.651 min) ALI-OLE-H7-220421-.D
Column Type: Capillary
Column Class: Standard non-polar
Active Phase: HP-101
Column
Length: 25 m
Carrier Gas: He
Column Diameter: 0.2 mm
Phase Thickness: 0.2 um
Data Type: Normal alkane
RI
Program Type: Ramp
Start T: 70 C
End T: 200 C
Heat Rate: 3 K/min
Start Time: 2 min
Source: Jerkovic, I.;
Mastelic, J.; Marijanovic, Z., Bound volatile compounds and essential oil from the fruit of Maclura pomifera (Raf.) Schneid. (osage orange), Flavour Fragr. J., 22, 2007, 84-88.

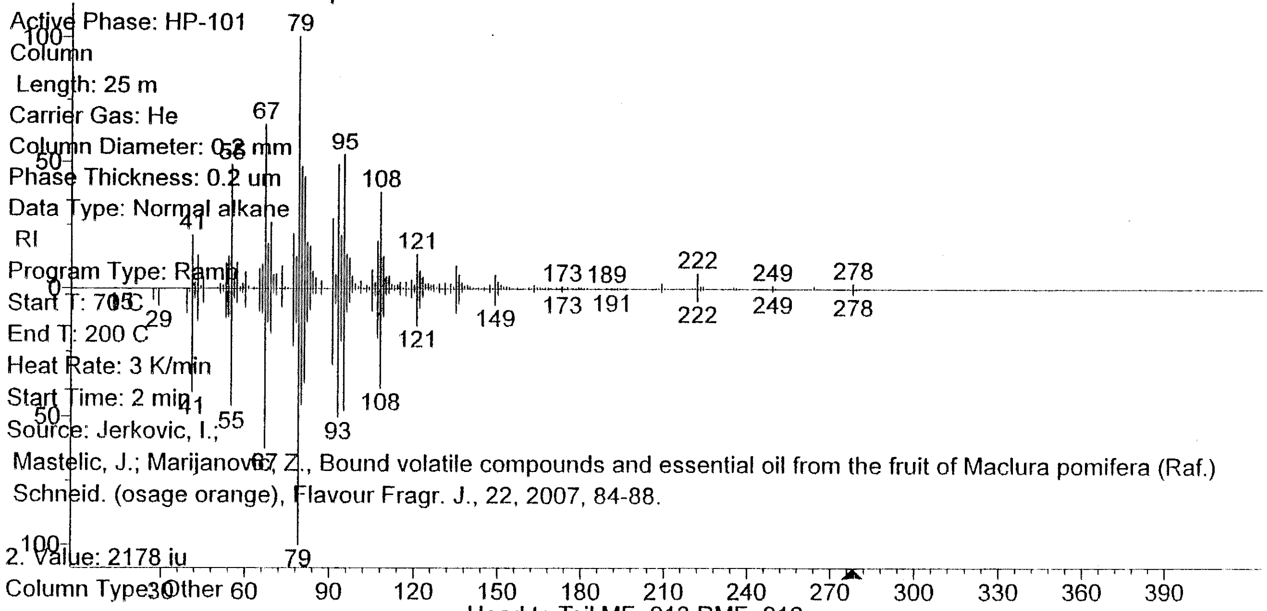

2. Value: 2178 iu
Column Type: Other
Column
Class: Standard non-polar
Active Phase: Methyl Silicone
Data Type: Normal alkane RI
Program Type:
Ramp
Source: Ardrey, R.E.; Moffat, A.C., Gas-liquid chromatographic retention indices of 1318 substances of toxicological interest on SE-30 or OV-1 stationary phase, J. Chromatogr., 220, 1981, 195-252.
<...>

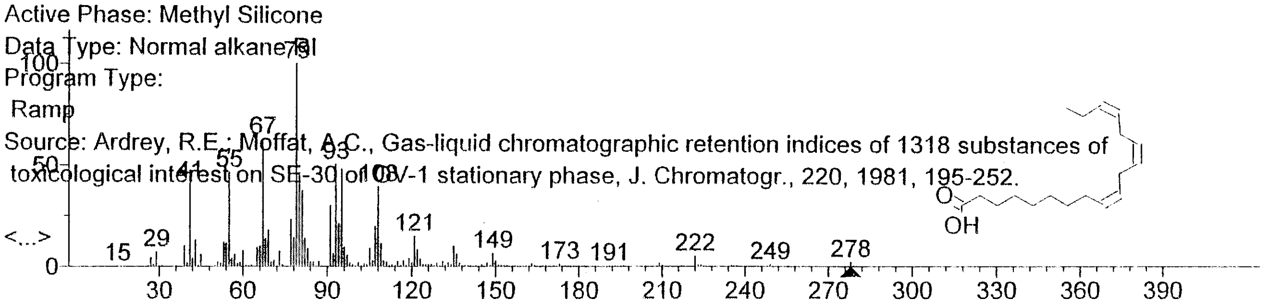

(mainlib) 9,12,15-Octadecatrienoic acid, (Z,Z,Z)-

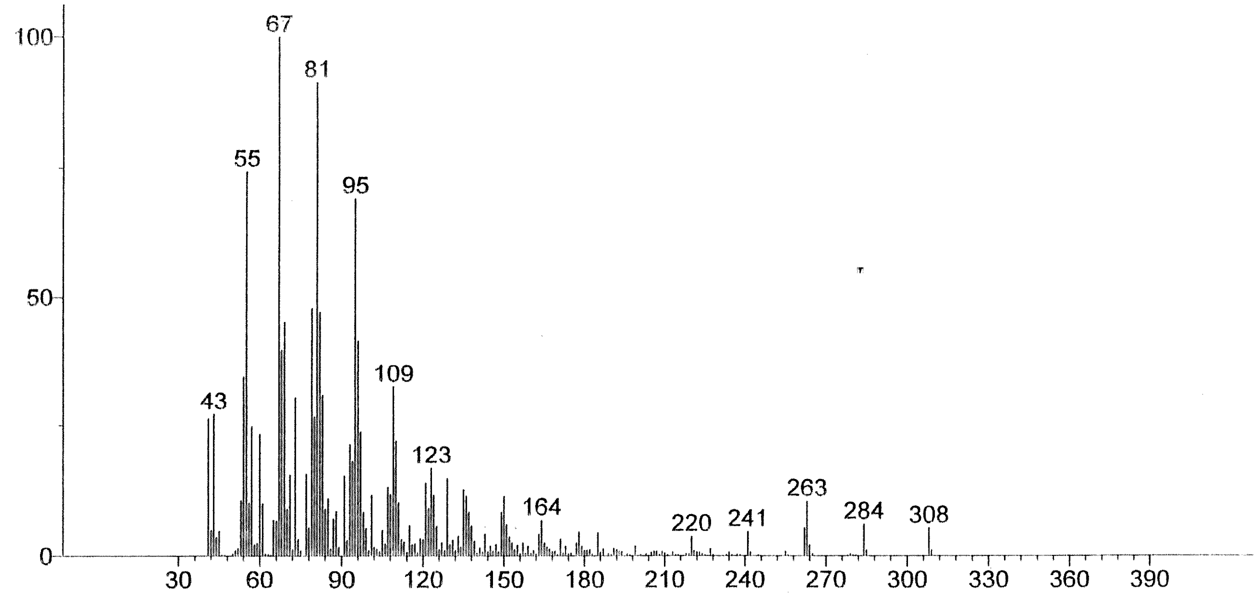

(Text File) +EI Scan (61.999 min) ALI-OLE-H7-220421-.D

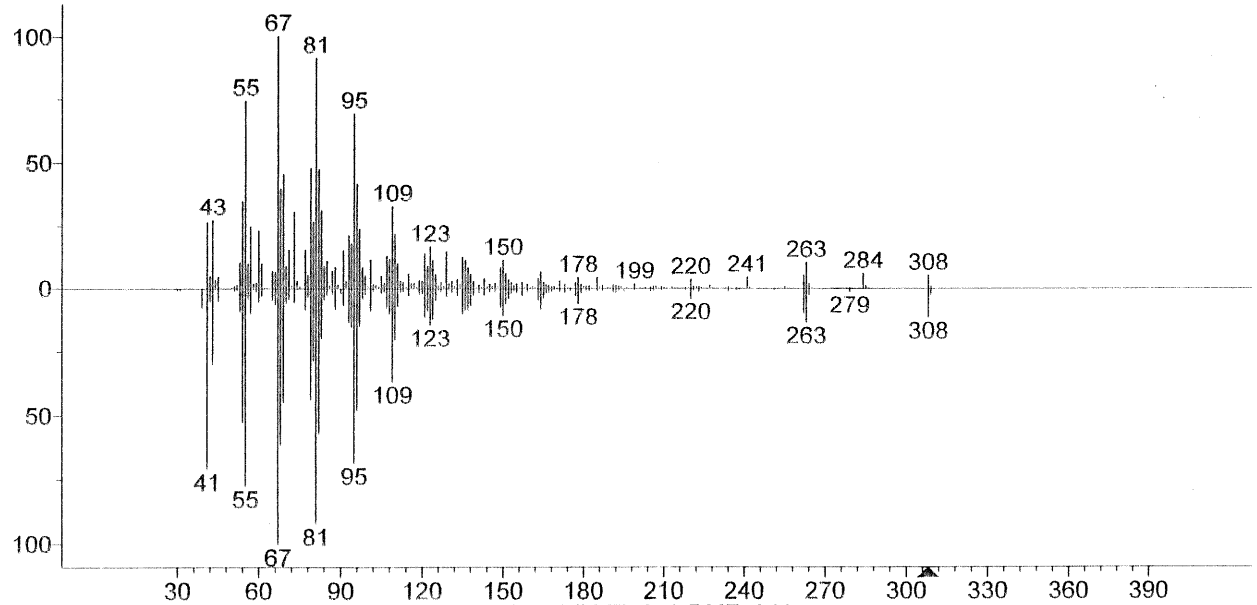

Head to Tail MF=873 RMF=913

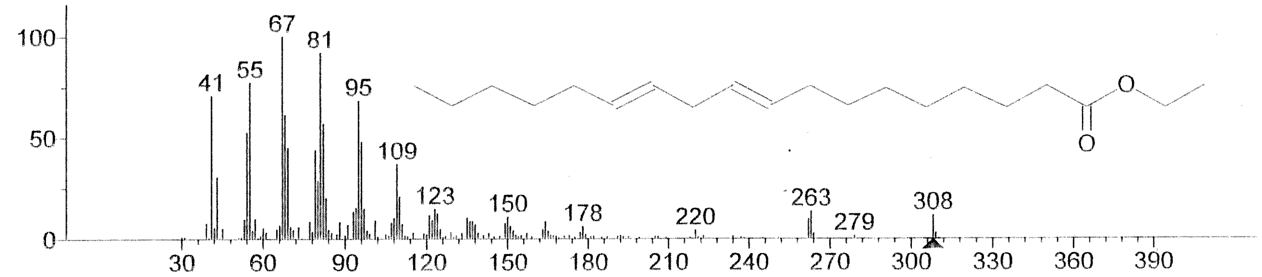

(mainlib) 9,12-Octadecadienoic acid, ethyl ester

Name: 9,12-Octadecadienoic acid, ethyl ester
Formula: $C_{20}H_{36}O_2$
MW: 308 CAS#: 7619-08-1 NIST#: 249157 ID#: 28827 DB: mainlib
Other DBs: HODOC
Contributor: TNO Volatile Compounds in Food - Chemical Concepts
10 largest peaks:
67 999 | 81 919 | 55 772 | 41 707 | 95 683 | 68 613 | 82 569 | 54 525 | 96 478 | 69 448 |
Synonyms:
1.Ethyl (9E,12E)-9,12-octadecadienoate #

Estimated non-polar retention index (n-alkane scale):
Value: 2193 iu
Confidence interval (Esters): 47(50%) 201(95%) iu

Retention index.
1. Value: 2171 iu
Column Type: Capillary
Column Class: Semi-standard non-polar
Active Phase: HP
-5MS
Column Length: 30 m
Carrier Gas: He
Column Diameter: 0.25 mm
Phase Thickness: 0.25 um
Data Type:
Normal alkane RI
Program Type: Complex
Description: 40C (10min) => 3C/min =>120C =>10C/min =>250C
(5min)
Source: Ansorena, D.; Gimeno, O.; Astiasarán, I.; Bello, J., Analysis of volatile compounds by GC-MS of a dry fermented sausage: chorizo de Pamplona, Food Res. Int., 34, 2001, 67-75.

2. Value: 2527 iu
Column Type:
Capillary
Column Class: Standard polar
Active Phase: CP-Wax 52CB
Column Length: 60 m
Carrier Gas:
He
Column Diameter: 0.25 mm
Phase Thickness: 0.25 um
Data Type: Linear RI
Program Type:
Complex
Description: 45C(5min) =>10C/min =>80C =>2C/min =>240C
Source: Romeo, V.; Ziino, M.; Giuffrrida,
D.; Condurso, C.; Verzera, A., Flavour profile of capers (Capparis spinosa L.) from the Eolian Archipelago by HS -SPME/GC?MS, Food Chem., 101, 2007, 1272-1278.

<...>

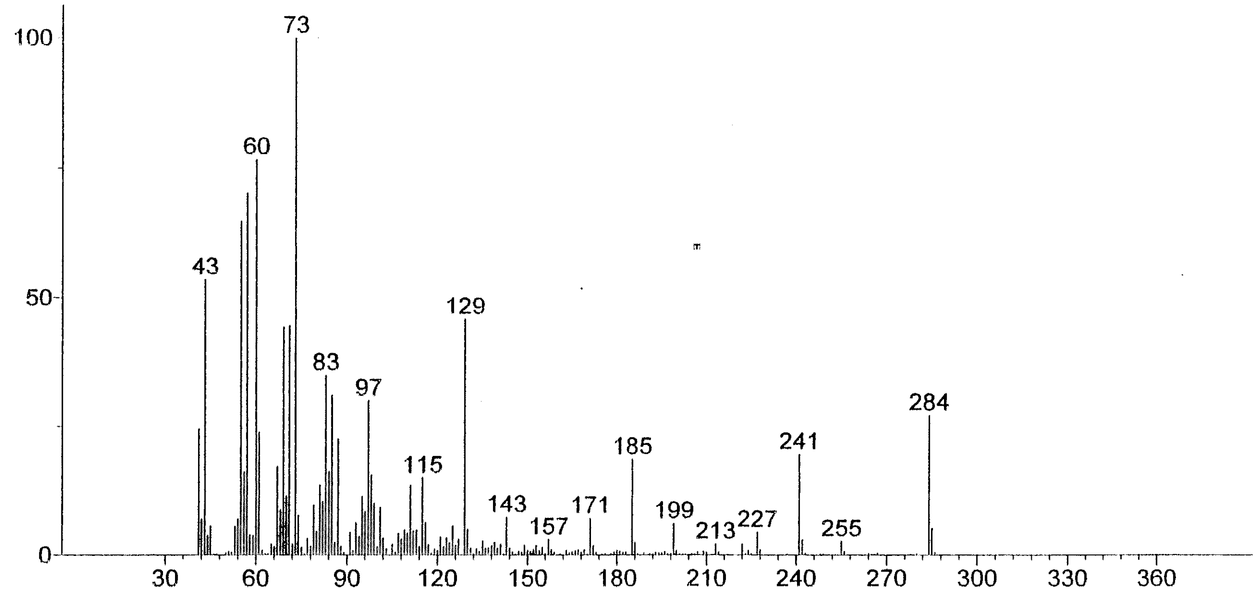

(Text File) +EI Scan (62.252 min) ALI-OLE-H7-220421-.D

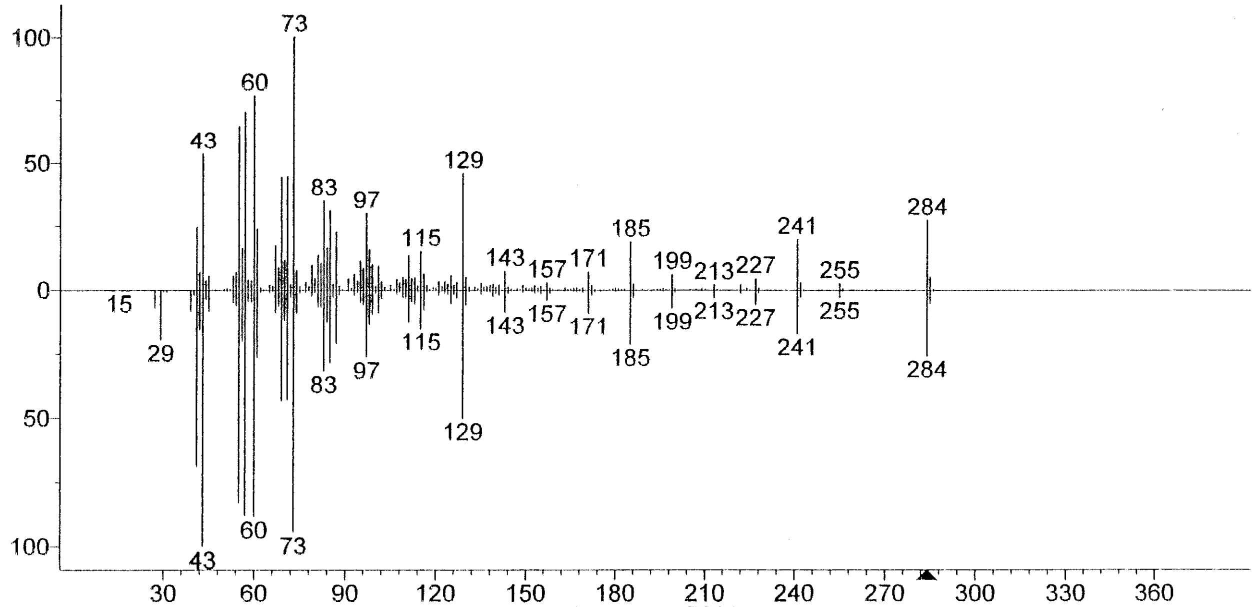

Head to Tail MF=894 RMF=897

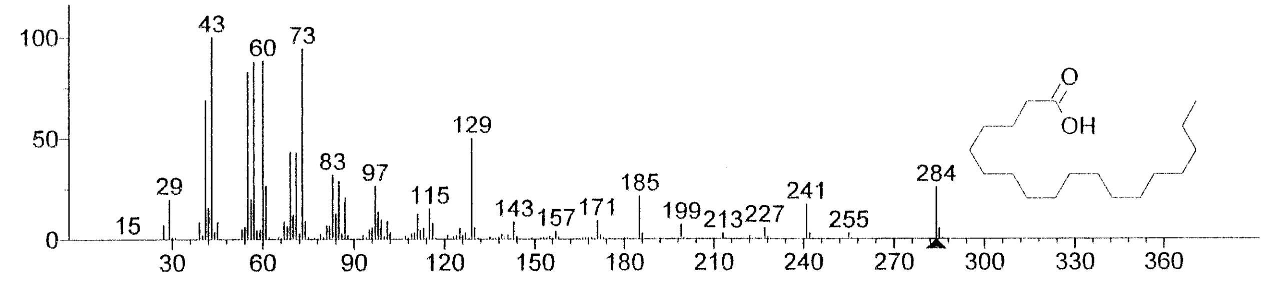

(mainlib) Octadecanoic acid

112

Name: Octadecanoic acid
Formula: $C_{18}H_{36}O_2$
MW: 284 CAS#: 57-11-4 NIST#: 290961 ID#: 8481 DB: mainlib
Other DBs: Fine, TSCA, RTECS, EPA, HODOC, NIH, EINECS, IRDB
Contributor: NIST Mass Spectrometry Data Center, 1998.
10 largest peaks:
43 999 | 73 942 | 60 881 | 57 875 | 55 827 | 41 688 | 129 500 | 69 432 | 71 429 | 83 317 |
Synonyms:
1.Stearic acid
2.n-Octadecanoic acid
3.Humko Industrene R
4.Hydrofol Acid 150
5.Hystrene S-97
6.Hystrene T-70
7.Hystrene 80
8.Industrene R
9.Kam 1000
10.Kam 2000
11.Kam 3000
12.Neo-Fat 18
13.Neo-Fat 18-53
14.Neo-Fat 18-54
15.Neo-Fat 18-55
16.Neo-Fat 18-59
17.NAA 173
18.PD 185
19.Stearex Beads
20.Stearophanic acid
21.Steric acid
22.Vanicol
23.1-Heptadecanecarboxylic acid
24.Heptadecanecarboxylic acid
25.Neo-fat 18-61
26.Pearl stearic
27.Century 1240
28.Dar-chem 14
29.Emersol 120
30.Emersol 132
31.Emersol 150
32.Formula 300
33.Glycon dp
34.Glycon tp
35.Glycon S-70
36.Glycon S-80
37.Glycon S-90
38.Groco 54
39.Groco 55
40.Groco 55l
41.Groco 58
42.Groco 59
43.Hy-phi 1199
44.Hy-phi 1205
45.Hy-phi 1303
46.Hy-phi 1401

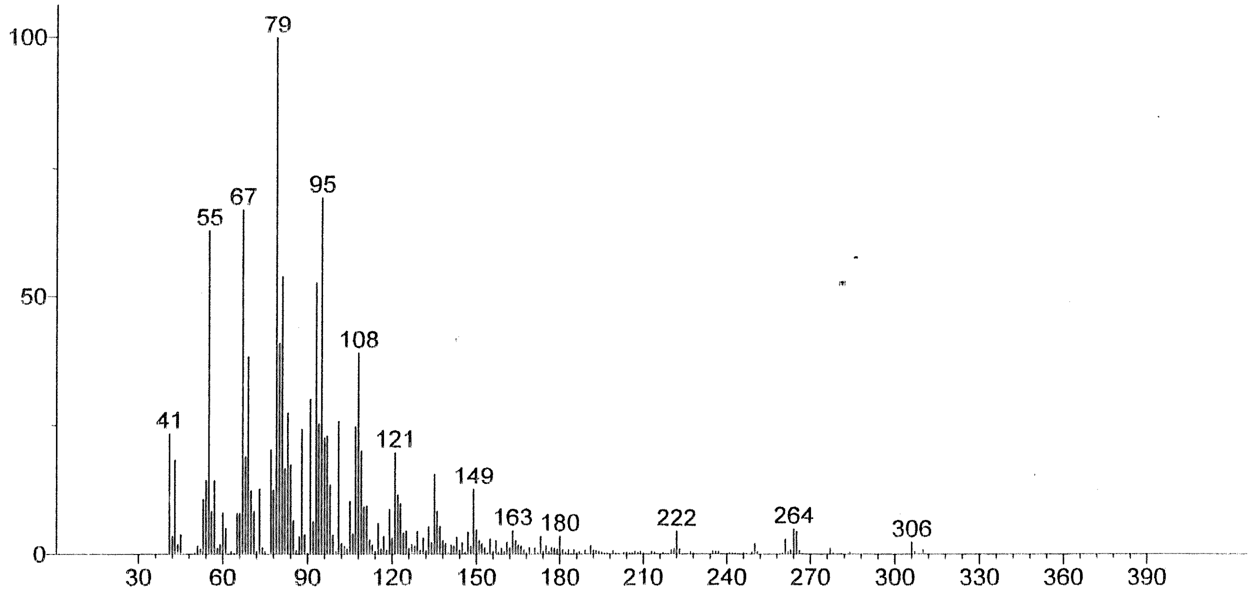

(Text File) +EI Scan (62.722 min) ALI-OLE-H7-220421-.D

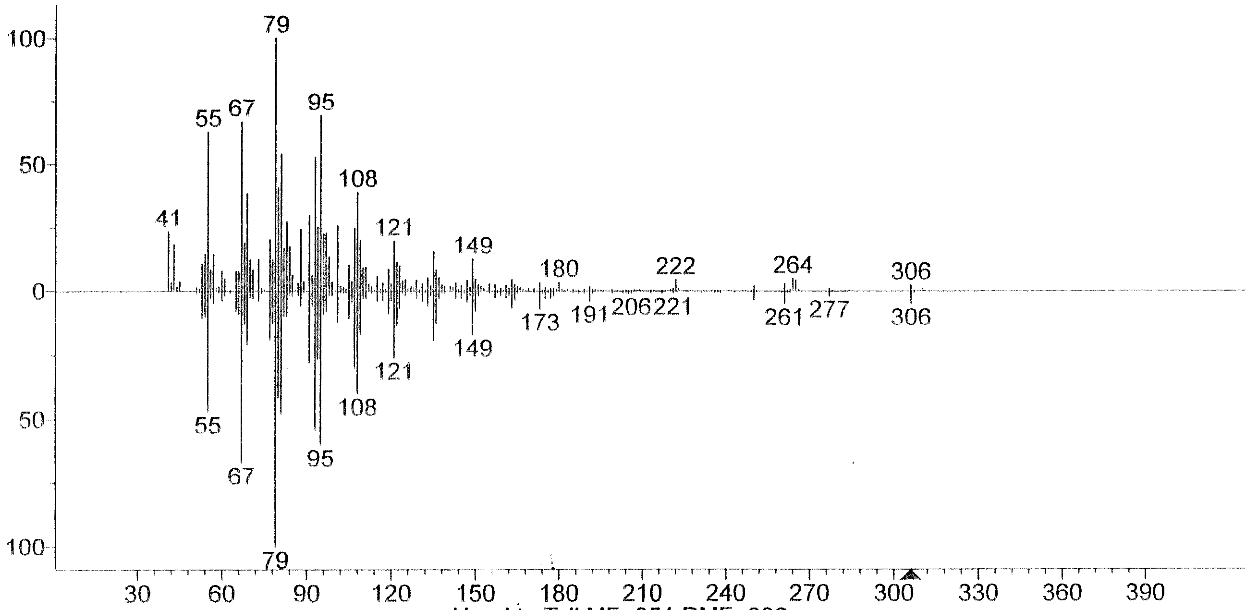

Head to Tail MF=851 RMF=896

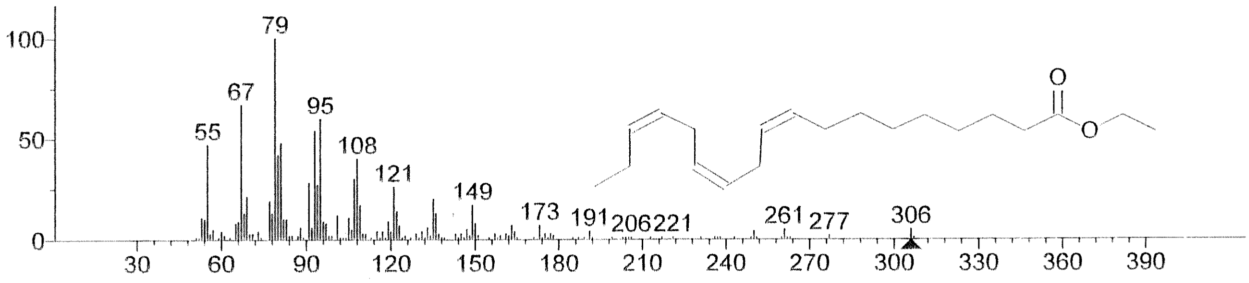

(mainlib) Ethyl 9,12,15-octadecatrienoate

Name: Ethyl 9,12,15-octadecatrienoate
Formula: $C_{20}H_{34}O_2$
MW: 306 NIST#: 336774 ID#: 41709 DB: mainlib
Contributor: William W. Christie, Mylnefield Lipid Analysis, Invergowrie, Dundee, Scotland, UK
10 largest peaks:
79 999 | 67 669 | 95 599 | 93 539 | 81 479 | 55 469 | 80 419 | 108 399 | 107 299 | 91 279 |
Synonyms:
no synonyms.

Estimated non-polar retention index (n-alkane scale):
Value: 2201 iu
Confidence interval (Esters): 47(50%) 201(95%) iu

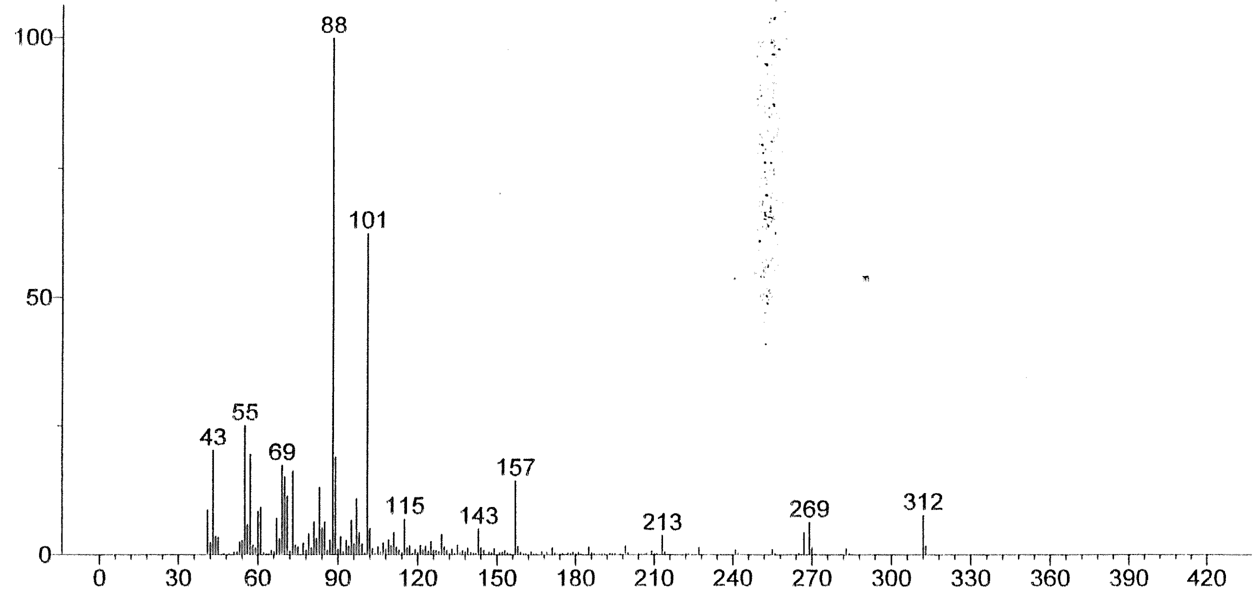

(Text File) +EI Scan (65.260 min) ALI-OLE-H7-220421-.D

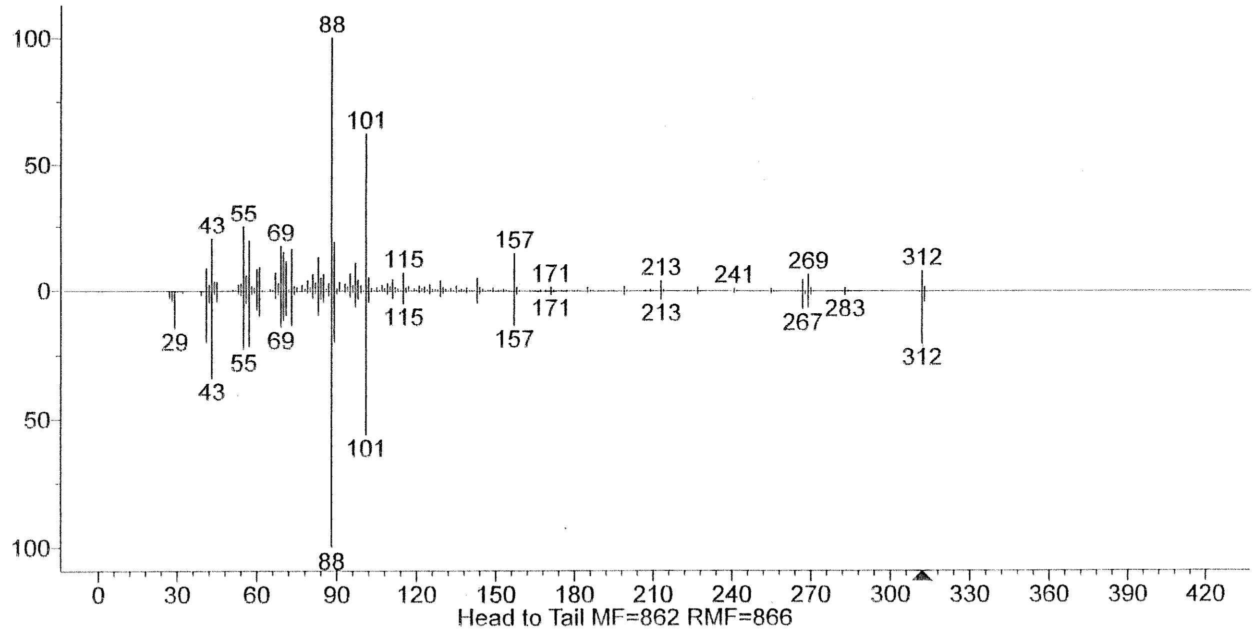

Head to Tail MF=862 RMF=866

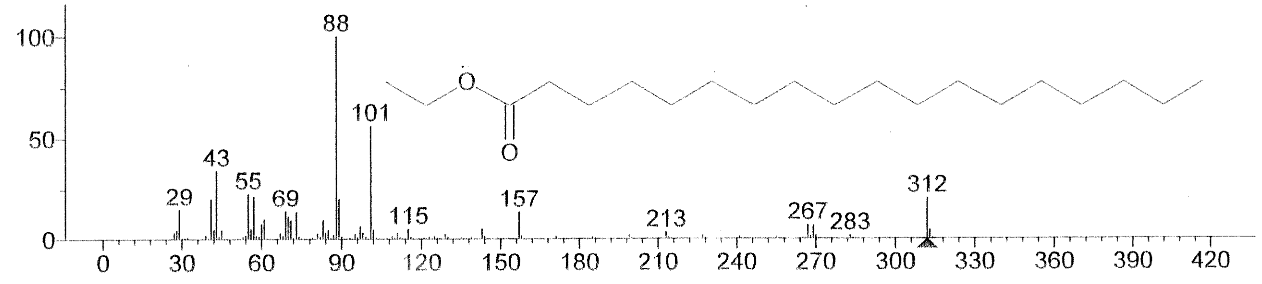

(replib) Octadecanoic acid, ethyl ester

Name: Octadecanoic acid, ethyl ester
Formula: $C_{20}H_{40}O_2$
MW: 312 CAS#: 111-61-5 NIST#: 36393 ID#: 11117 DB: replib
Other DBs: Fine, TSCA, RTECS, HODOC, NIH, EINECS, IRDB
Contributor: R.T.HOLMAN,UNIVERSITY OF MINNESOTA
10 largest peaks:
88 999 | 101 559 | 43 339 | 55 226 | 57 213 | 312 203 | 41 200 | 89 200 | 29 146 | 69 139 |
Synonyms:
1.Stearic acid, ethyl ester
2.Ethyl n-octadecanoate
3.Ethyl octadecanoate
4.Ethyl stearate
5.Radia 7185

Estimated non-polar retention index (n-alkane scale):
Value: 2177 iu
Confidence interval (Esters): 47(50%) 201(95%) iu

Retention index.
1. Value: 2181 iu
Column Type: Capillary
Column Class: Standard non-polar
Active Phase: CP Sil 5 CB
Column
Length: 50 m
Carrier Gas: He
Column Diameter: 0.32 mm
Phase Thickness: 0.4 um
Data Type: Linear
RI
Program Type: Ramp
Start T: 60 C
End T: 280 C
Heat Rate: 3 K/min
Start Time: 10 min
End Time: 60
min
Source: Pino, J.A.; Marbot, R.; Vázquez, C., Characterization of volatiles in strawberry guava (Psidium cattleianum Sabine) fruit, J. Agric. Food Chem., 49, 2001, 5883-5887.

2. Value: 2202 iu
Column Type:
Capillary
Column Class: Standard non-polar
Active Phase: OV-1
Column Length: 25 m
Carrier Gas:
He
Column Diameter: 0.32 mm
Data Type: Linear RI
Program Type: Ramp
Start T: 40 C
End T: 280 C
Heat

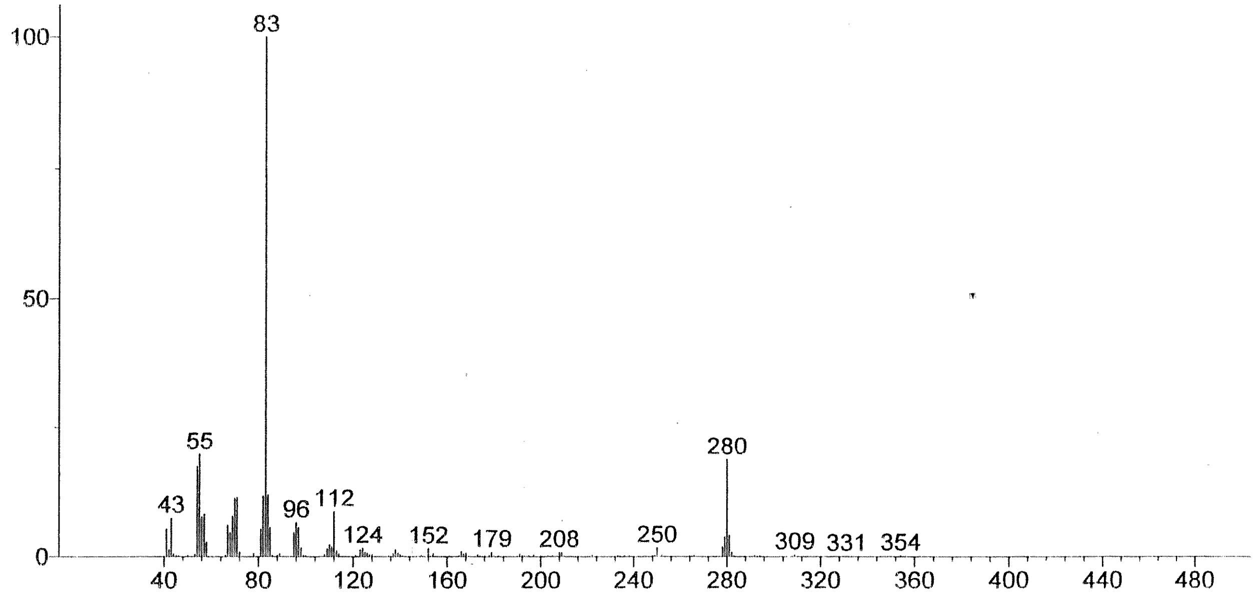

(Text File) +EI Scan (72.088 min) ALI-OLE-H7-220421-.D Subtract

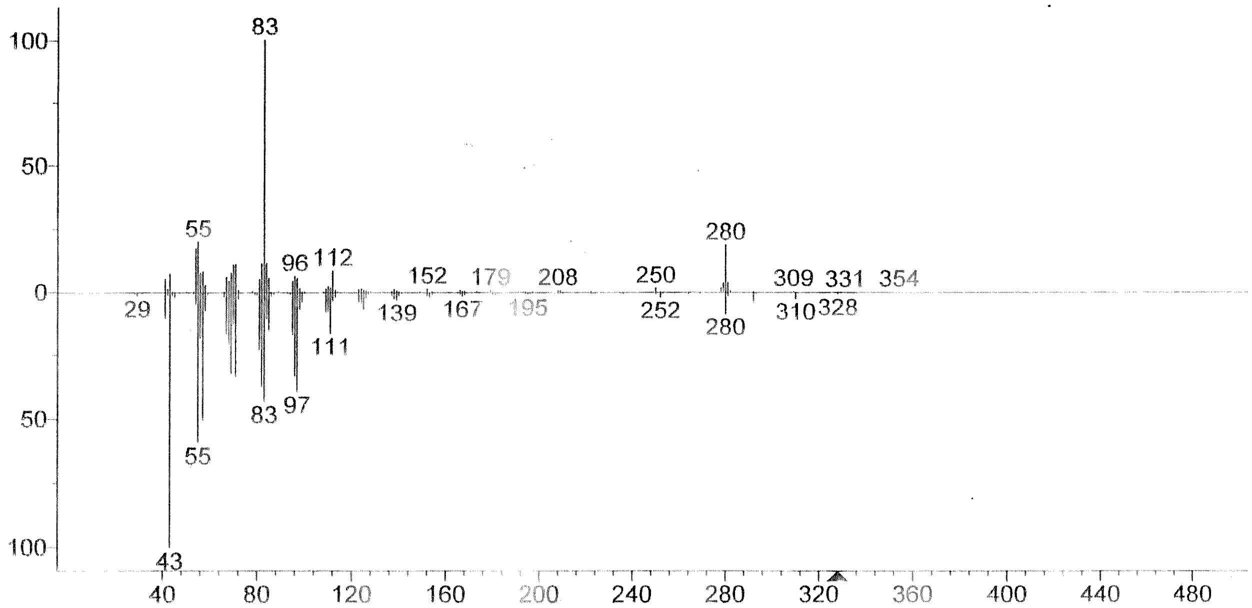

Head to Tail MF=656 RMF=666

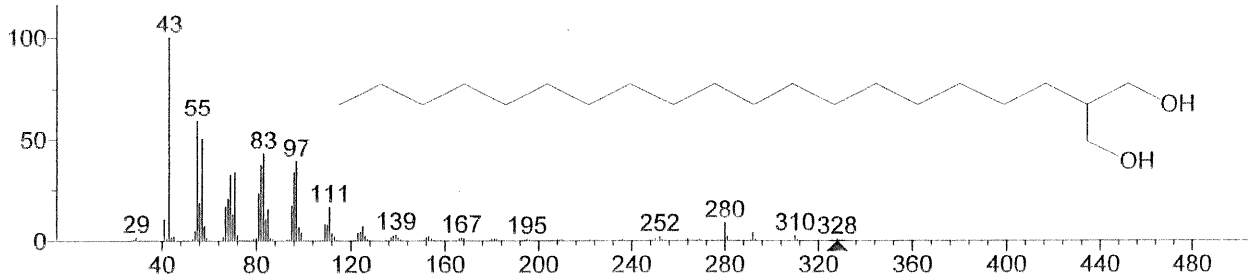

(mainlib) 2-Octadecyl-propane-1,3-diol

Name: 2-Octadecyl-propane-1,3-diol
Formula: $C_{21}H_{44}O_2$
MW: 328 CAS#: 5337-61-1 NIST#: 193417 ID#: 6741 DB: mainlib
Other DBs: None
Contributor: Chemical Concepts
10 largest peaks:
43 999 | 55 589 | 57 503 | 83 429 | 97 391 | 82 372 | 71 337 | 96 337 | 69 323 | 81 232 |
Synonyms:
1.2-Octadecyl-1,3-propanediol #

Estimated non-polar retention index (n-alkane scale):
Value: 2530 iu
Confidence interval (Alcohols): 41(50%) 176(95%) iu

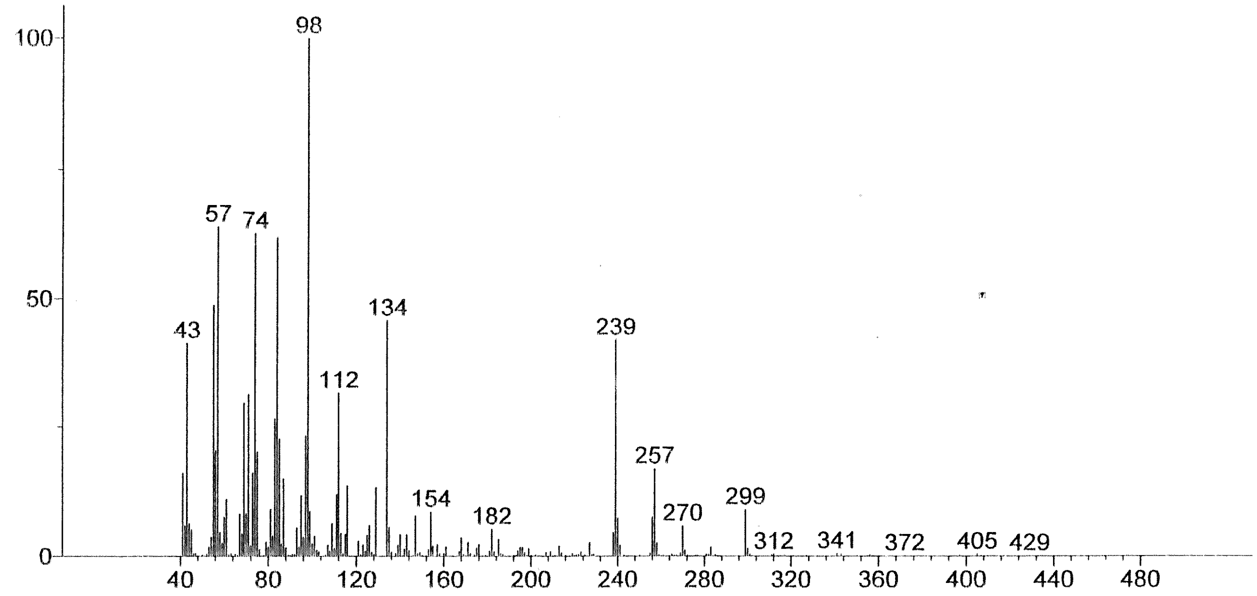

(Text File) +EI Scan (75.951 min) ALI-OLE-H7-220421-.D Subtract

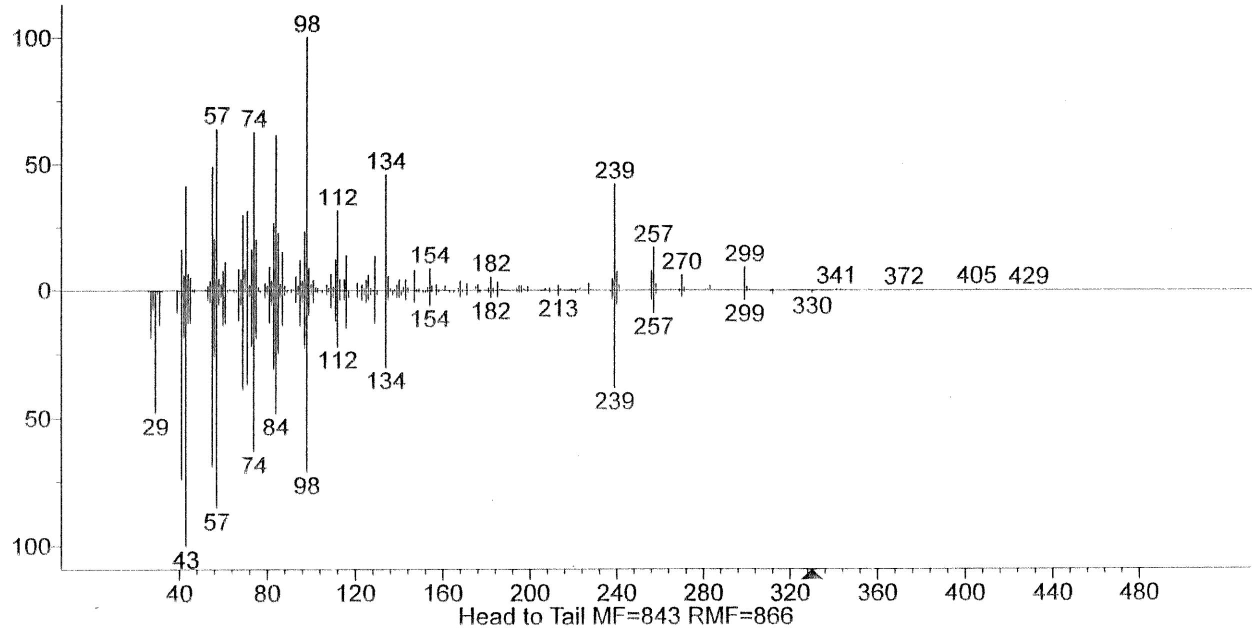

Head to Tail MF=843 RMF=866

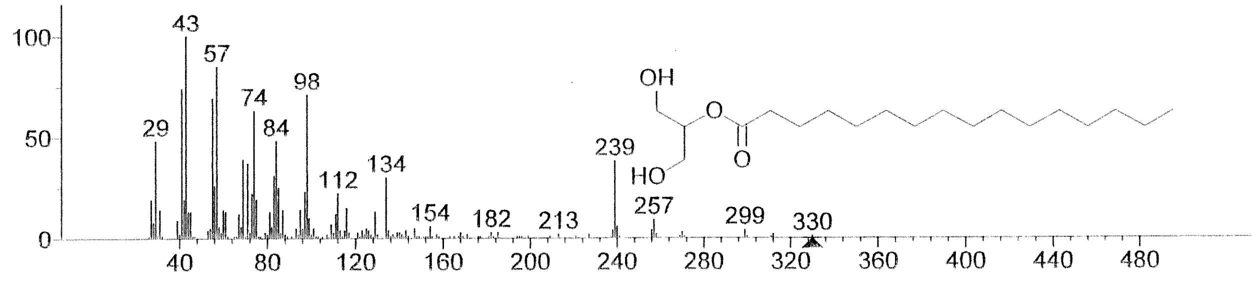

(mainlib) Hexadecanoic acid, 2-hydroxy-1-(hydroxymethyl)ethyl ester

Name: Hexadecanoic acid, 2-hydroxy-1-(hydroxymethyl)ethyl ester
Formula: $C_{19}H_{38}O_4$
MW: 330 CAS#: 23470-00-0 NIST#: 15400 ID#: 7156 DB: mainlib
Other DBs: None
10 largest peaks:
43 999 | 57 850 | 41 740 | 98 710 | 55 690 | 74 630 | 29 480 | 84 480 | 69 390 | 239 380 |
Synonyms:
1.Palmitin, 2-mono-
2.Palmitic acid β-monoglyceride
3.2-Hexadecanoyl glycerol
4.2-Monopalmitin
5.2-Monopalmitoyl-sn-glycerol
6.1,2,3-Propanetriol 2-hexandecanoyl ester
7.Glycerol β-palmitate
8.2-Hydroxy-1-(hydroxymethyl)ethyl palmitate #

Estimated non-polar retention index (n-alkane scale):
Value: 2498 iu
Confidence interval (Diverse functional groups): 89(50%) 382(95%) iu

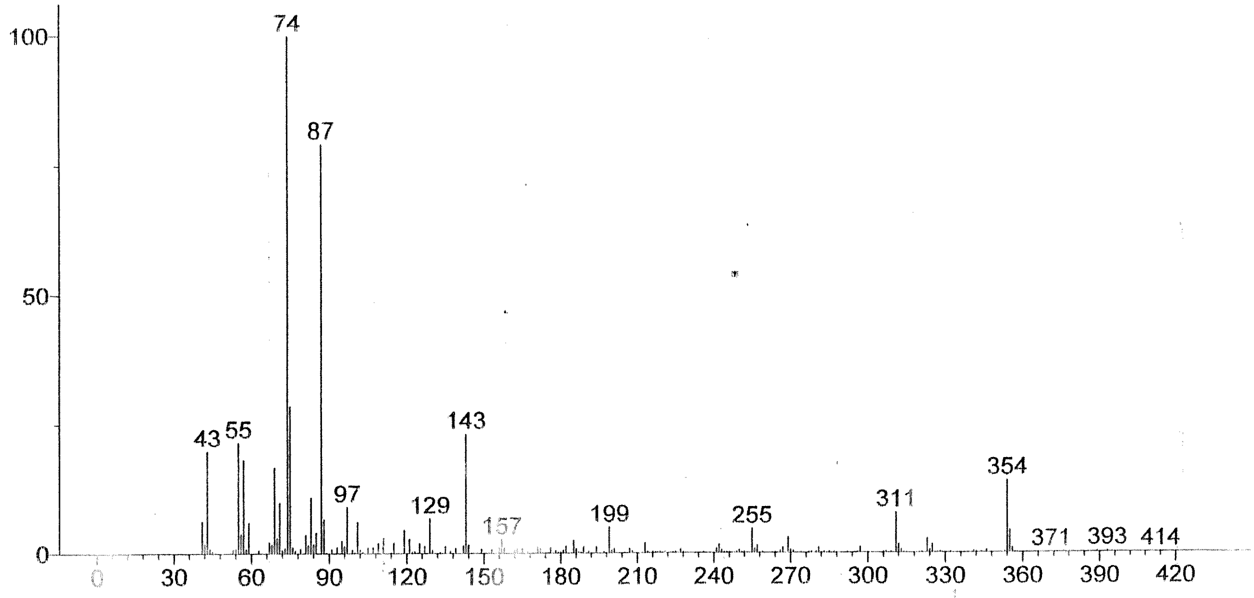

(Text File) +EI Scan (76.286 min) ALI-OLE-H7-220421-.D Subtract

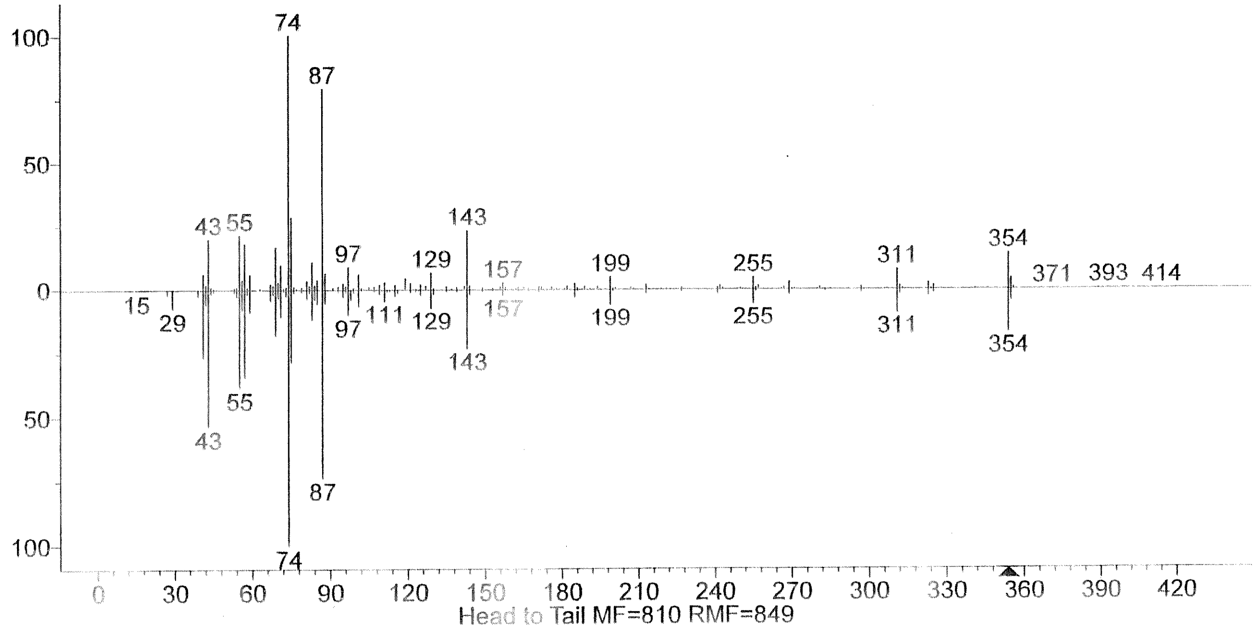

Head to Tail MF=810 RMF=849

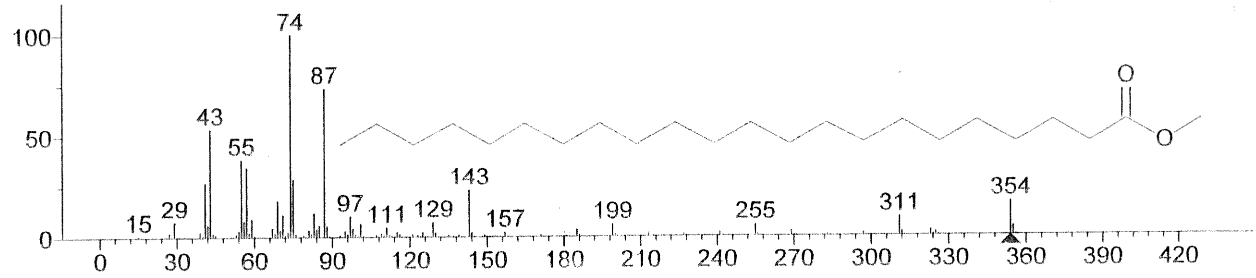

(mainlib) Docosanoic acid, methyl ester

Name: Docosanoic acid, methyl ester
Formula: $C_{23}H_{46}O_2$
MW: 354 CAS#: 929-77-1 NIST#: 333108 ID#: 38271 DB: mainlib
Other DBs: Fine, TSCA, HODOC, NIH, EINECS
Contributor: NIST Mass Spectrometry Data Center
10 largest peaks:
74 999 | 87 734 | 43 534 | 55 383 | 57 344 | 75 284 | 41 266 | 143 230 | 69 180 | 354 167 |
Synonyms:
1.Behenic acid, methyl ester
2.Methyl behenate
3.Methyl docosanoate
4.n-Docosanoic acid methyl ester
5.Kemester 9022

Estimated non-polar retention index (n-alkane scale):
Value: 2475 iu
Confidence interval (Esters): 47(50%) 201(95%) iu

Retention index.
1. Value: 2531 iu
Column Type: Capillary
Column Class: Standard non-polar
Active Phase: CP Sil 5 CB
Column
Length: 50 m
Carrier Gas: H2
Column Diameter: 0.25 mm
Phase Thickness: 0.15 um
Data Type: Linear
RI
Program Type: Ramp
Start T: 40 C
End T: 300 C
Heat Rate: 4 K/min
Start Time: 3 min
Source: Heinrich,
G.; Pfeifhofer, H.W.; Stabentheiner, E.; Sawidis, T., Glandular hairs of Sigesbeckia jorullensis Kunth (Asteraceae): morphology, histochemistry and composition of essential oil, Ann. Bot. Rome, 89, 2002, 459-469.

2. Value: 2502
iu
Column Type: Capillary
Column Class: Standard non-polar
Active Phase: DB-1
Column Length: 30
m
Column Diameter: 0.2 mm
Phase Thickness: 0.25 um
Data Type: Linear RI
Program Type: Ramp
Start T: 50
C
End T: 300 C
Heat Rate: 5 K/min

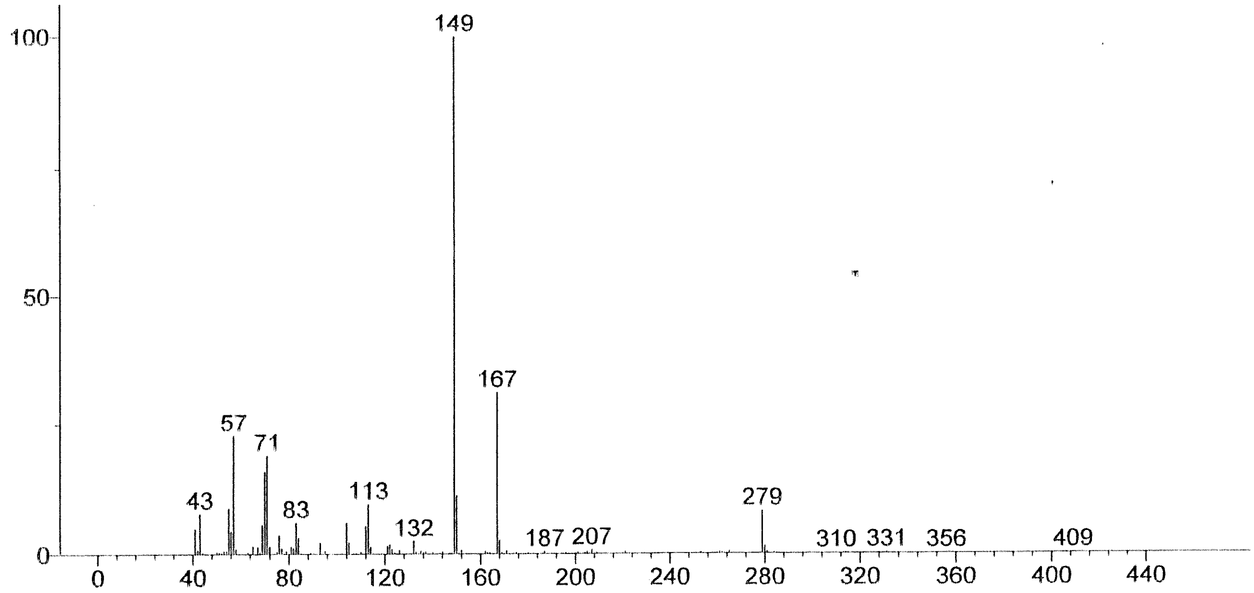

(Text File) +EI Scan (76.668 min) ALI-OLE-H7-220421-.D Subtract

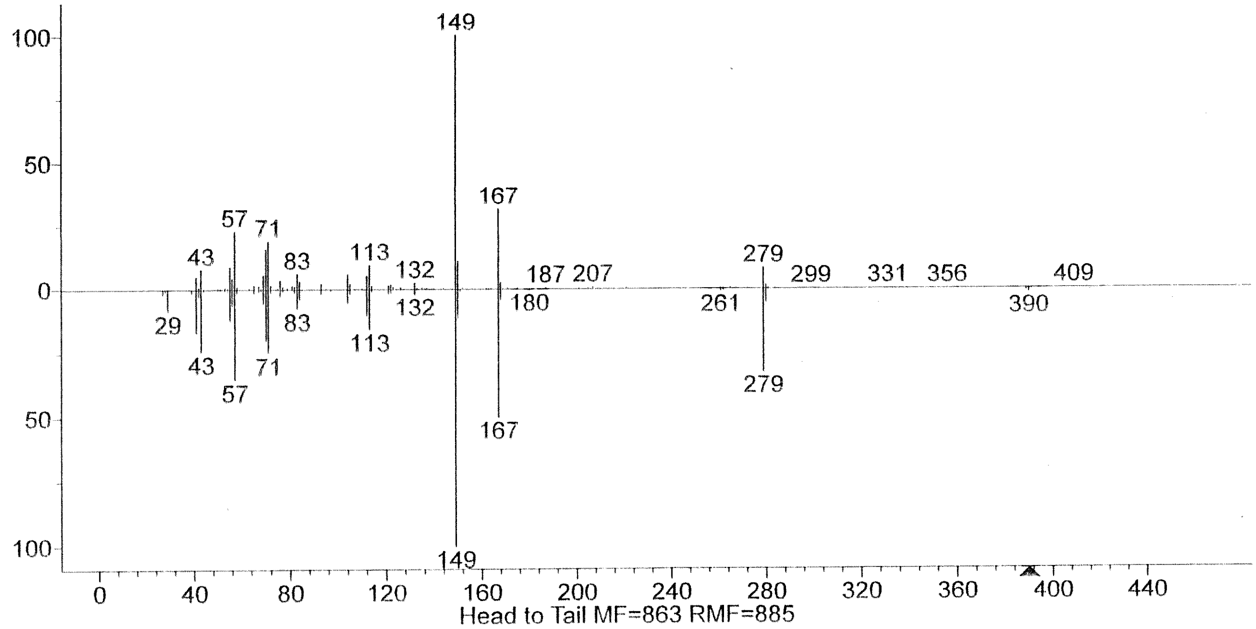

Head to Tail MF=863 RMF=885

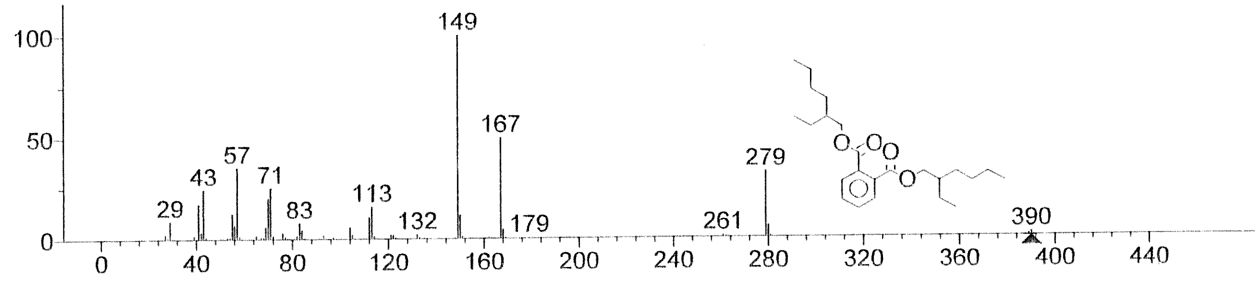

(mainlib) Bis(2-ethylhexyl) phthalate

Name: Bis(2-ethylhexyl) phthalate
Formula: $C_{24}H_{38}O_4$
MW: 390 CAS#: 117-81-7 NIST#: 229113 ID#: 110646 DB: mainlib
Other DBs: Fine, TSCA, RTECS, EPA, HODOC, NIH, EINECS, IRDB
Contributor: Japan AIST/NIMC Database- Spectrum MS-NW-8633
10 largest peaks:
149 999 | 167 497 | 57 351 | 279 322 | 71 249 | 43 242 | 70 200 | 41 170 | 113 156 | 55 125 |
Synonyms:
1.1,2-Benzenedicarboxylic acid, bis(2-ethylhexyl) ester
2.Phthalic acid, bis(2-ethylhexyl) ester
3.Bis(2-ethylhexyl) 1,2-benzenedicarboxylate
4.Bisoflex 81
5.Compound 889
6.Di(ethylhexyl) phthalate
7.Di(2-ethylhexyl) phthalate
8.Dioctyl phthalate
9.DEHP
10.DOP
11.Ethylhexyl Phthalate
12.Eviplast 80
13.Eviplast 81
14.Fleximel
15.Flexol DOP
16.Kodaflex DOP
17.Octoil
18.Octyl phthalate
19.Palatinol AH
20.Pittsburgh PX-138
21.Sicol 150
22.Staflex DOP
23.Truflex DOP
24.Vestinol AH
25.Vinicizer 80
26.Witcizer 312
27.1,2-Benzenedicarboxylic acid, bis(ethylhexyl) ester
28.2-Ethylhexyl phthalate
29.Dioctyl-o-benzenedicarboxylate
30.Phthalic acid di(2-ethylhexyl) ester
31.di-iso-Octyl phthalate
32.Bis(ethylhexyl) phthalate
33.Bisoflex DOP
34.Celluflex DOP
35.Di(2-ethylhexyl) o-phthalate
36.Di-sec-octyl phthalate
37.Flexol plasticizer DOP
38.Hercoflex 260
39.NCI-C52733
40.Polycizer 162
41.PX-138
42.RC plasticizer DOP
43.Behp
44.Bis-(2-ethylhexyl)ester kyseliny ftalove
45.DAF 68
46.Di(2-ethylhexyl)orthophthalate

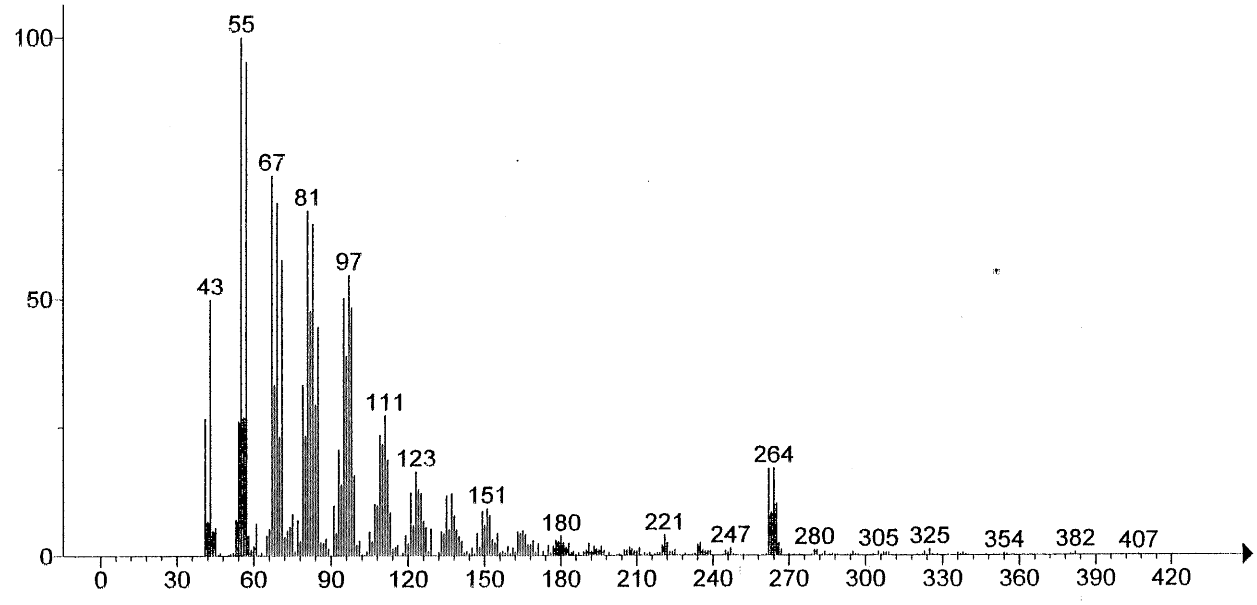

(Text File) +EI Scan (78.821 min) ALI-OLE-H7-220421-.D Subtract

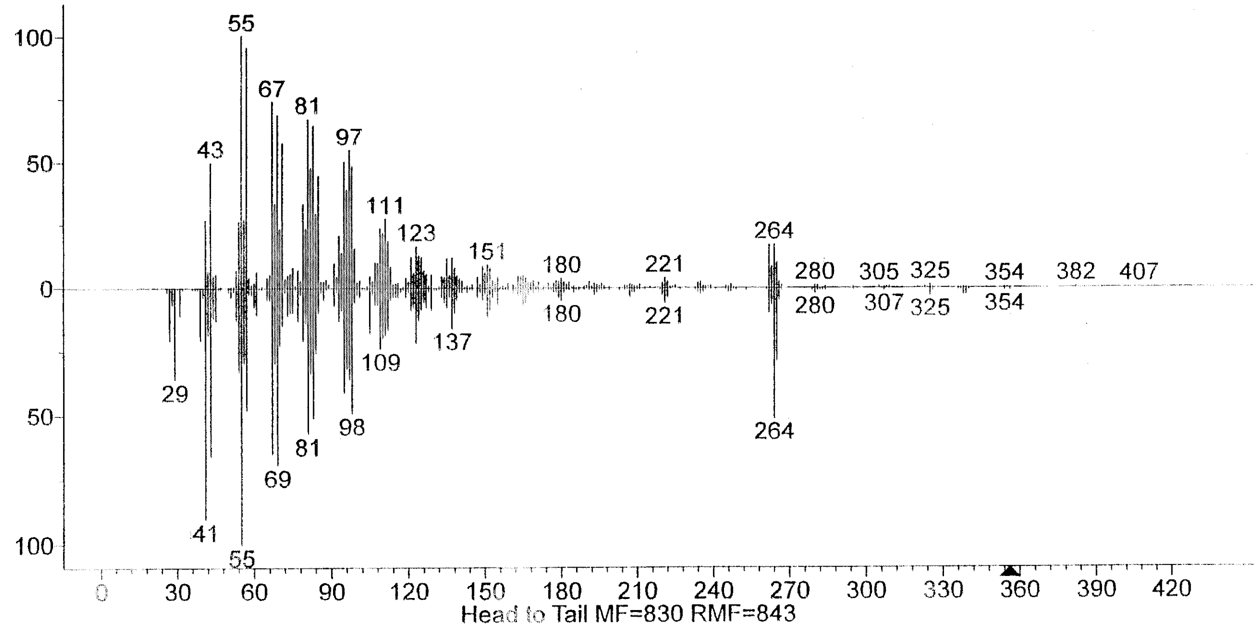

Head to Tail MF=830 RMF=843

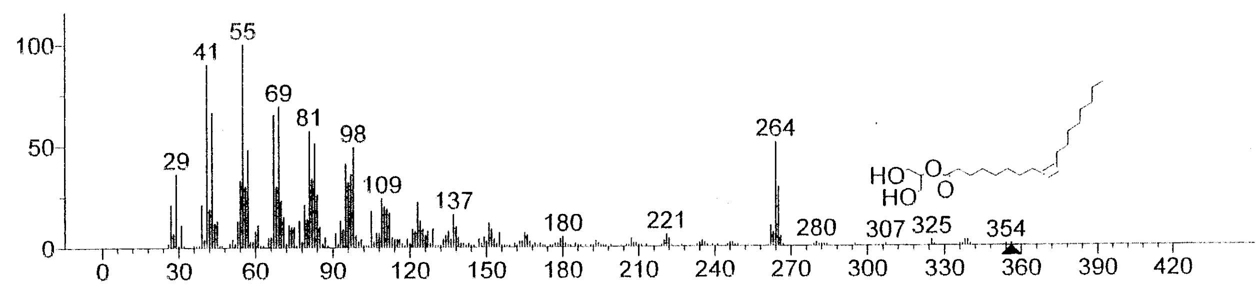

(mainlib) 9-Octadecenoic acid (Z)-, 2-hydroxy-1-(hydroxymethyl)ethyl ester

Name: 9-Octadecenoic acid (Z)-, 2-hydroxy-1-(hydroxymethyl)ethyl ester
Formula: $C_{21}H_{40}O_4$
MW: 356 CAS#: 3443-84-3 NIST#: 16061 ID#: 17482 DB: mainlib
Other DBs: None
10 largest peaks:
55 999 | 41 900 | 69 690 | 43 660 | 67 650 | 81 570 | 83 510 | 264 510 | 98 490 | 57 480 |
Synonyms:
1.Olein, 2-mono-
2.β-Monoolein
3.Glycerol 2-monooleate
4.2-Monoolein
5.2-Monooleoylglycerol
6.2-Oleoyl glycerol ether
7.2-Oleoylglycerol
8.2-Hydroxy-1-(hydroxymethyl)ethyl (9Z)-9-octadecenoate #

Estimated non-polar retention index (n-alkane scale):
Value: 2705 iu
Confidence interval (Diverse functional groups): 89(50%) 382(95%) iu

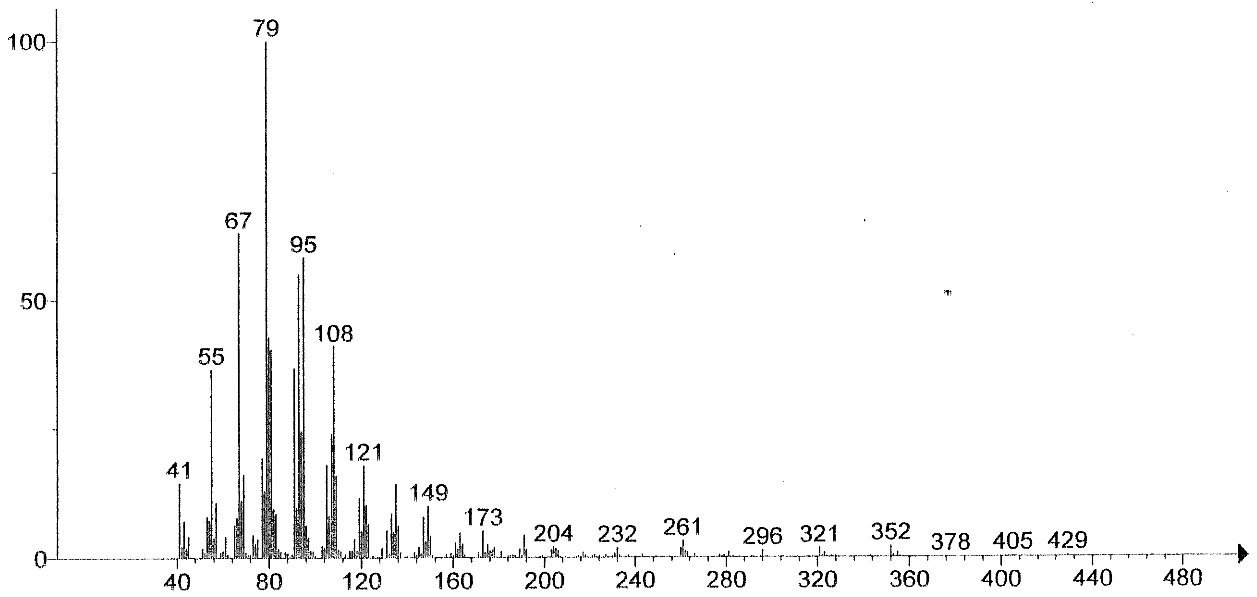

(Text File) +EI Scan (78.931 min) ALI-OLE-H7-220421-.D Subtract

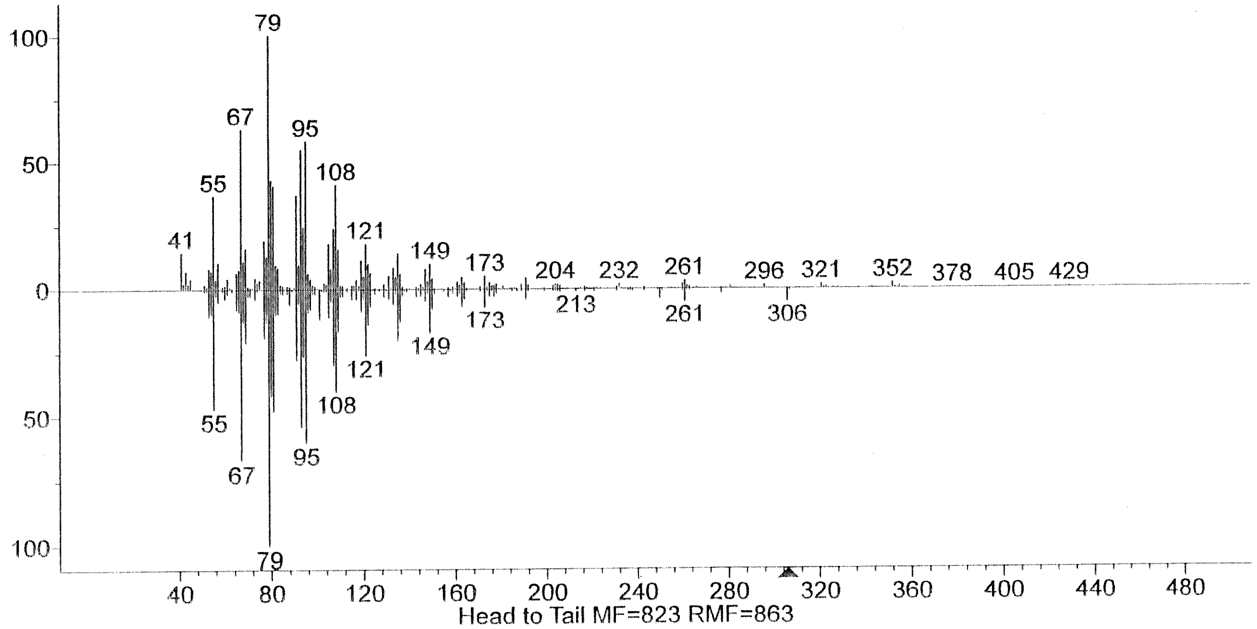

Head to Tail MF=823 RMF=863

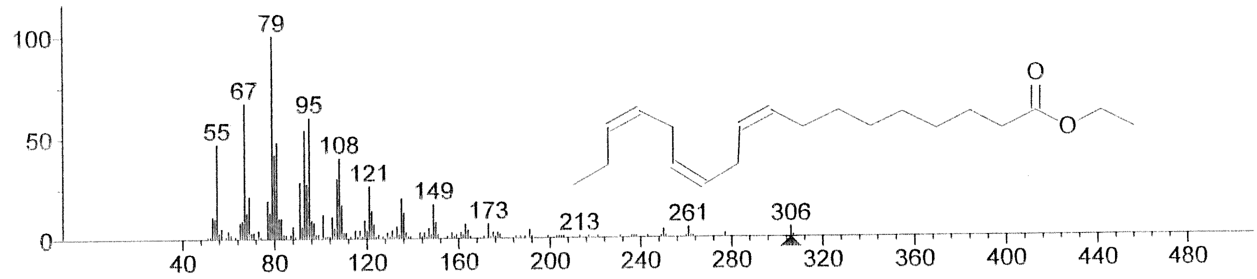

(mainlib) Ethyl 9,12,15-octadecatrienoate

Name: Ethyl 9,12,15-octadecatrienoate
Formula: $C_{20}H_{34}O_2$
MW: 306 NIST#: 336774 ID#: 41709 DB: mainlib
Contributor: William W. Christie, Mylnefield Lipid Analysis, Invergowrie, Dundee, Scotland, UK
10 largest peaks:
79 999 | 67 669 | 95 599 | 93 539 | 81 479 | 55 469 | 80 419 | 108 399 | 107 299 | 91 279 |
Synonyms:
no synonyms.

Estimated non-polar retention index (n-alkane scale):
Value: 2201 iu
Confidence interval (Esters): 47(50%) 201(95%) iu

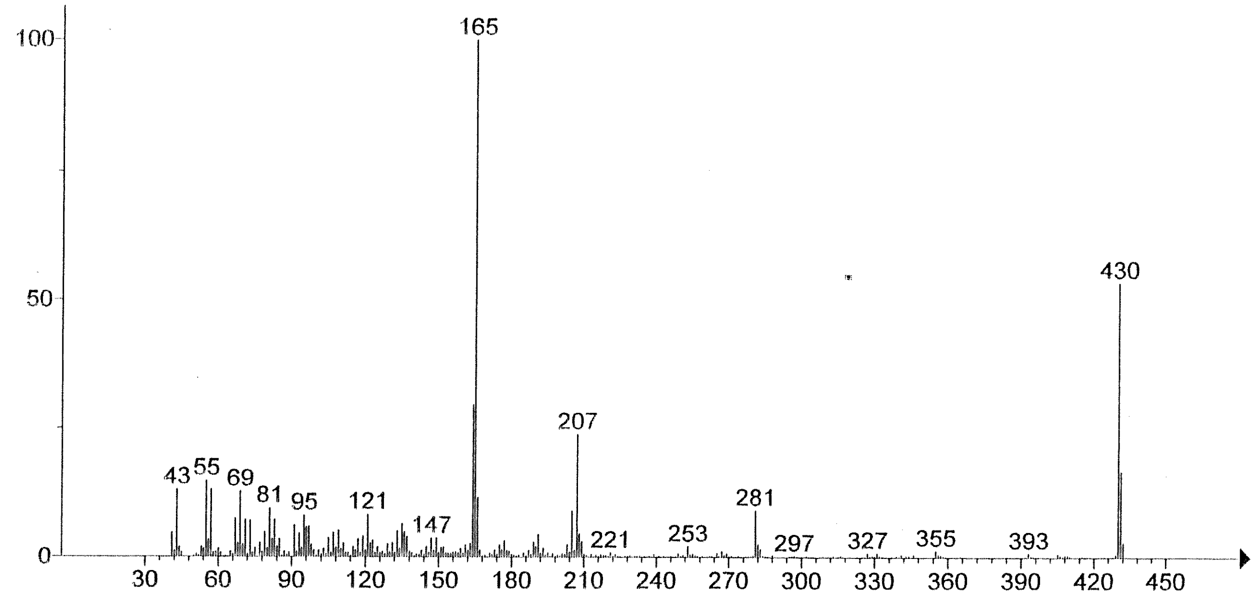

(Text File) +EI Scan (86.513 min) ALI-OLE-H7-220421-.D

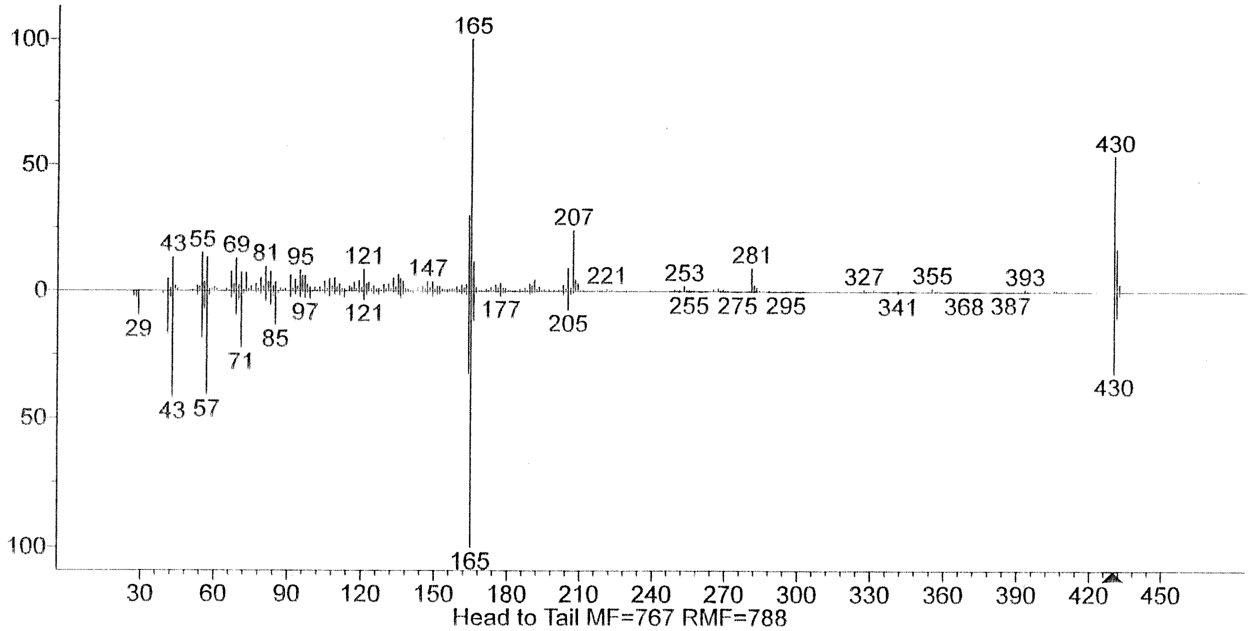

Head to Tail MF=767 RMF=788

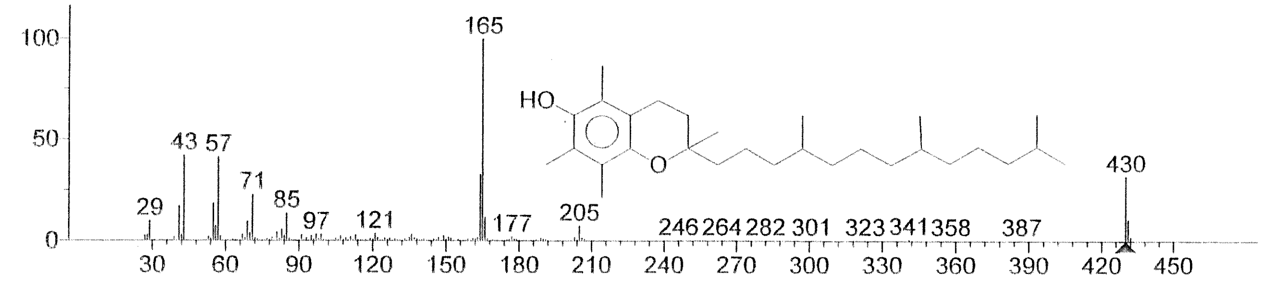

(mainlib) Vitamin E

Name: Vitamin E
Formula: $C_{29}H_{50}O_2$
MW: 430 CAS#: 59-02-9 NIST#: 151382 ID#: 122577 DB: mainlib
Other DBs: TSCA, RTECS, HODOC, NIH, EINECS
Contributor: Chemical Concepts
10 largest peaks:
165 999 | 43 419 | 57 410 | 164 326 | 430 318 | 71 226 | 55 185 | 41 171 | 85 135 | 166 117 |
Synonyms:
1.2H-1-Benzopyran-6-ol, 3,4-dihydro-2,5,7,8-tetramethyl-2-(4,8,12-trimethyltridecyl)-, [2R-[2R*(4R*,8R*)]]-
2.α-Tocopherol
3.α-Tokoferol
4.(All-R)-α-Tocopherol
5.(2R,4'R,8'R)-α-Tocopherol
6.D-α-tocopherol
7.Almefrol
8.Antisterility vitamin
9.Denamone
10.Emipherol
11.Endo E
12.Ephynal
13.Eprolin
14.Eprolin S
15.Epsilan
16.Esorb
17.Etamican
18.Etavit
19.Evion
20.Evitaminum
21.Ilitia
22.Phytogermine
23.Profecundin
24.Spavit E
25.Syntopherol
26.Tokopharm
27.Vascuals
28.Verrol
29.Vi-E
30.Vitaplex E
31.Vitayonon
32.Viteolin
33.5,7,8-Trimethyltocol
34.component of E and C-Level
35.component of Estopherol
36.Aquasol E
37.Lan-E
38.Med-E
39.Vita E
40.Covi-ox
41.Spavit
42.(R,R,R)-α-Tocopherol
43.(+)-α-Tocopherol
44.(+)-α-Tocopherolα-tocopherolantisterility vitamin
45.[2R-2R*(4R*,8R*)]-3,4-Dihydro-2,5,7,8-tetramethyl-2-(4,8,12-trimethyltridecyl)-2H-1-benzopyran-6-ol
46.2,5,7,8-Tetramethyl-2-(4',8',12'-trimethyltridecyl)-6-chromanol

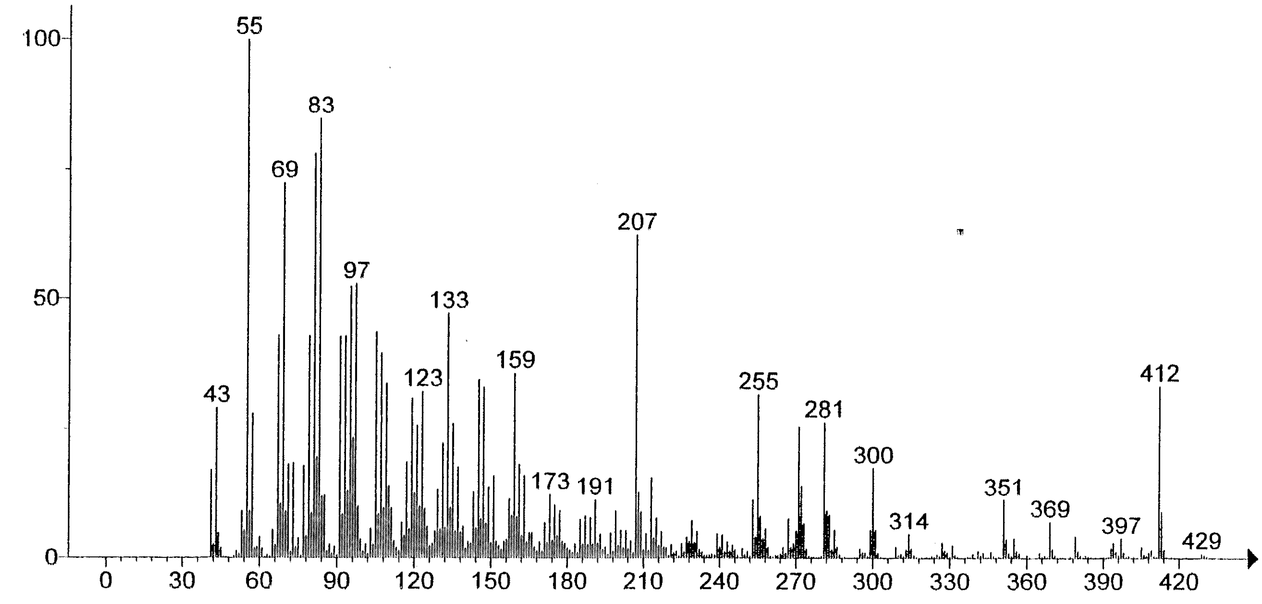

(Text File) +EI Scan (90.369 min) ALI-OLE-H7-220421-.D

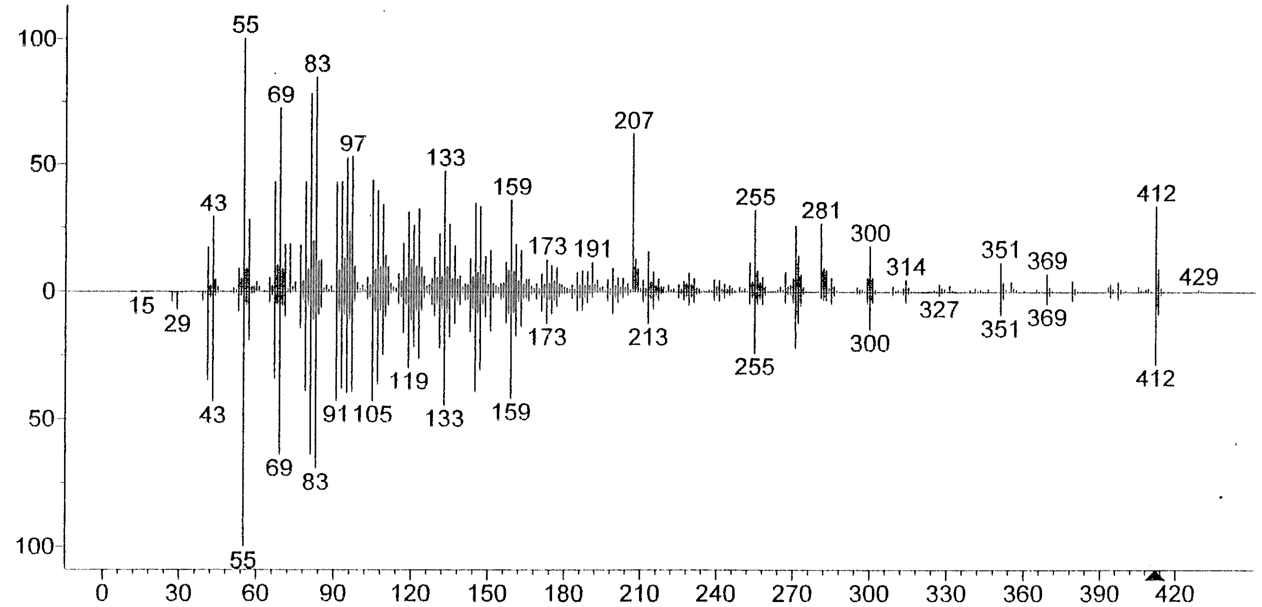

Head to Tail MF=861 RMF=902

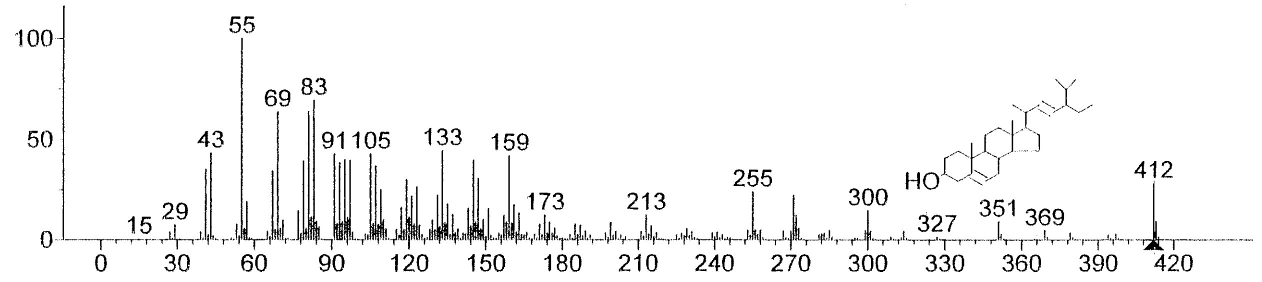

(mainlib) Stigmasterol

Name: Stigmasterol
Formula: $C_{29}H_{48}O$
MW: 412 CAS#: 83-48-7 NIST#: 352610 ID#: 18876 DB: mainlib
Other DBs: Fine, HODOC, NIH, EINECS
Contributor: NIST Mass Spectrometry Data Center
10 largest peaks:
55 999 | 83 692 | 81 638 | 69 636 | 133 444 | 43 431 | 91 427 | 105 427 | 159 418 | 95 398 |
Synonyms:
1.Stigmasta-5,22-dien-3-ol, (3β,22E)-
2.Stigmasta-5,22-dien-3β-ol
3.β-Stigmasterol
4.(24S)-5,22-Stigmastadien-3β-ol
5.Stigmasta-5,22-dien-3-ol, (3β)-
6.Stigmasterin
7.Phytosterol
8.5,22-Cholestadien-24-ethyl-3β-ol
9..DELTA.5,22-Stigmastadien-3β-ol
10.l-Stigmasterol
11.Stigmasta-5,22-dien-3-ol
12.(22E)-Stigmasta-5,22-dien-3-ol #

Estimated non-polar retention index (n-alkane scale):
Value: 2739 iu
Confidence interval (Low reliability): 174(50%) 752(95%) iu

Retention index.
1. Value: 3221.93 iu
Column Type: Capillary
Column Class: Standard non-polar
Active Phase: DB-1
Column
Length: 30 m
Carrier Gas: H2
Column Diameter: 0.25 mm
Phase Thickness: 0.25 um
Data Type: Kovats
RI
Program Type: Isothermal
Start T: 270 C
Source: Stránský, K.; Valterová, I.; Fiedler, P., Nonsaponifiable lipid components of the pollen of elder (Sambucus nigra L.), J. Chromatogr. A, 936, 2001, 173-181.

2. Value: 3222.15
iu
Column Type: Capillary
Column Class: Standard non-polar
Active Phase: DB-1
Column Length: 30
m
Carrier Gas: H2
Column Diameter: 0.25 mm
Phase Thickness: 0.25 um
Data Type: Kovats RI
Program

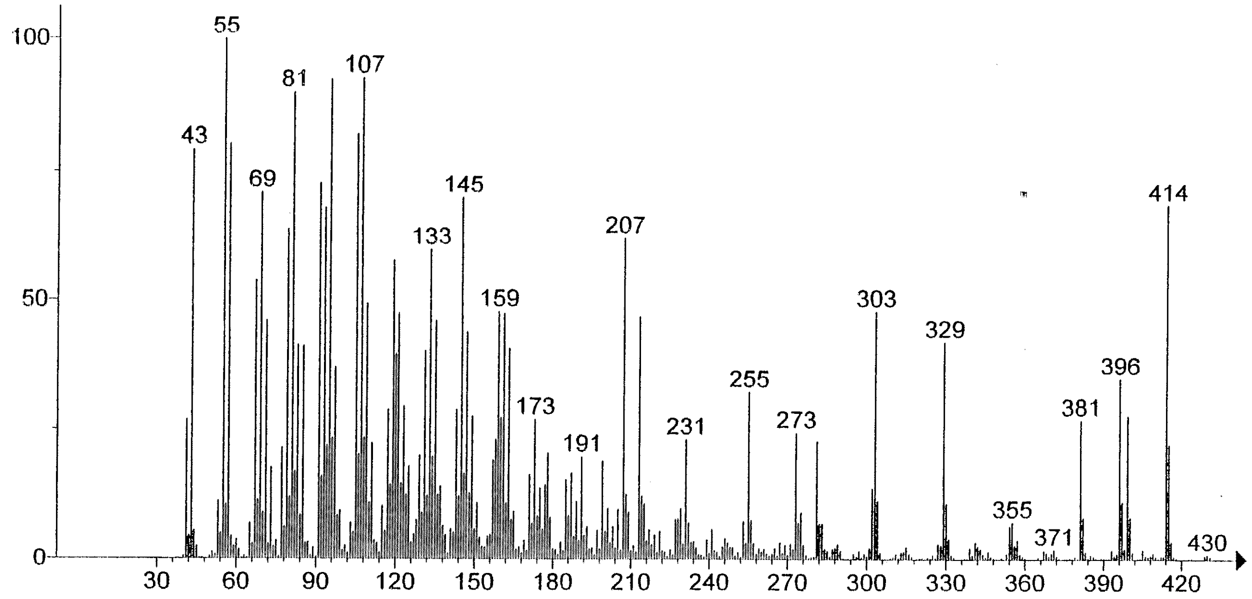

(Text File) +EI Scan (92.251 min) ALI-OLE-H7-220421-.D

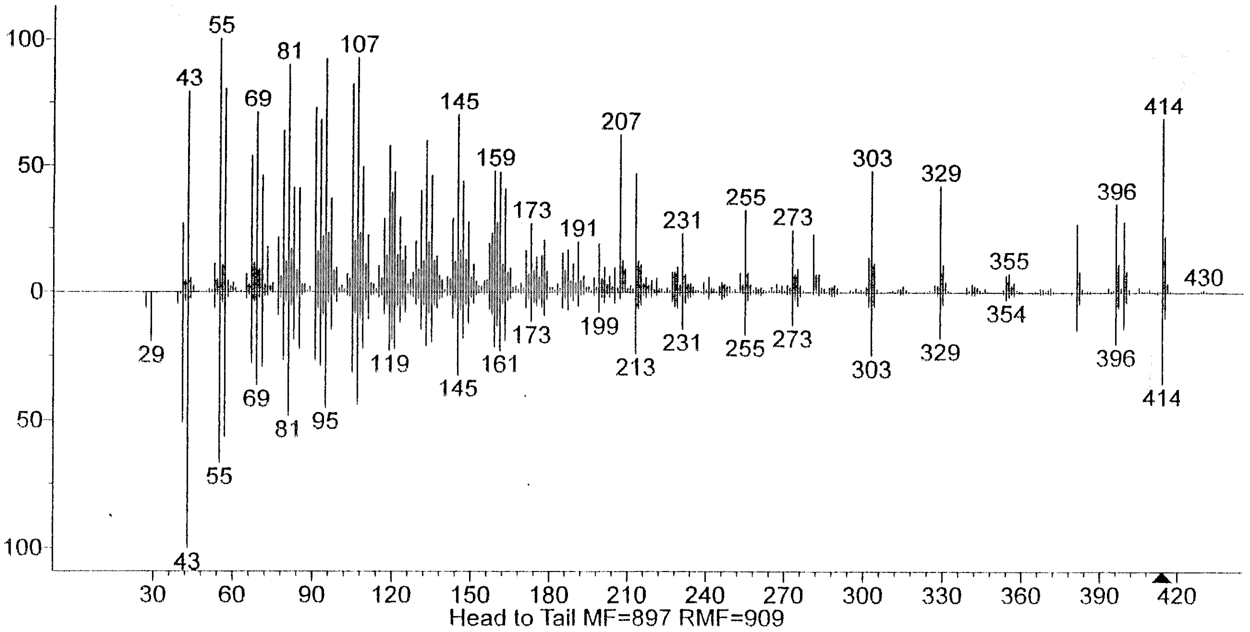

Head to Tail MF=897 RMF=909

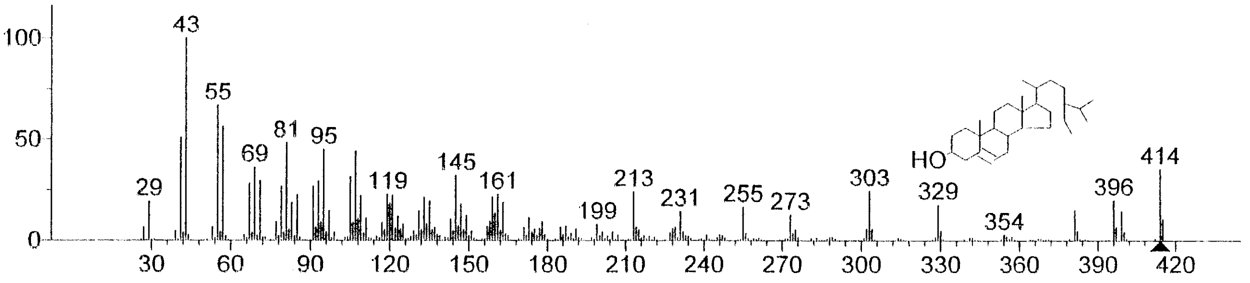

(mainlib) γ-Sitosterol

Name: γ-Sitosterol
Formula: $C_{29}H_{50}O$
MW: 414 CAS#: 83-47-6 NIST#: 151558 ID#: 6743 DB: mainlib
Other DBs: HODOC, EINECS
Contributor: Chemical Concepts
10 largest peaks:
43 999 | 55 667 | 57 563 | 41 507 | 81 481 | 95 449 | 107 440 | 69 362 | 414 354 | 145 321 |
Synonyms:
1.Stigmast-5-en-3-ol, (3β,24S)-
2.Stigmast-5-en-3β-ol, (24S)-
3.Clionasterol
4.Fucosterol, β-dihydro-
5.24β-Ethyl-5-cholesten-3β-ol
6.β-Dihydrofucosterol
7.22,23-Dihydroporiferasterol
8.24S-Ethylcholest-5-en-3β-ol
9.24β-Ethylcholesterol
10.Stigmast-5-en-3-ol #

Estimated non-polar retention index (n-alkane scale):
Value: 2731 iu
Confidence interval (Low reliability): 174(50%) 752(95%) iu

Retention index.
1. Value: 3066 iu
Column Type: Capillary
Column Class: Semi-standard non-polar
Active Phase: HP-5
Column
Length: 30 m
Carrier Gas: He
Column Diameter: 0.25 mm
Phase Thickness: 0.25 um
Data Type: Normal
alkane RI
Program Type: Complex
Description: 60C(5min) =>3C/min =>120C (2min) =>2C/min =>200C (2min)
=>3C/min =>320C
Source: Yasar, A.; Üçüncü, O.; Güleç, C.; Inceer, H.; Ayaz, S.; Yayh, N., GC-MS analysis of chloroform extracts in flowers, stems, and roots of Tripleurospermum callosum, Pharm. Biol., 43(2), 2005, 108-112.
