# Supplementary material for: Probing Antibacterial and Anticancer Potential of Selenicereus undatus, Pistacia vera L. and Olea europaea L. against Uropathogens, MCF-7 and A2780 Cancer Cells
Source: Molecules. 2023 Dec 18;28(24):8148. doi: 10.3390/molecules28248148 (PMC10746009; doi:10.3390/molecules28248148)
Supplement: Supplementary file 1 [file molecules-28-08148-s001.zip › SI- 2. GCMS OF Pistacia vera.pdf]

**Sample Name**	ALI-PIS-H1-210421	**Position**	12	**Instrument Name**	GCMS TQQQ	**User Name**	HEJ-G-104-03\Agilent
**Inj Vol**	1	**InjPosition**		**SampleType**		**IRM Calibration Status**	Not Applicable
**Data Filename**	ALI-PIS-H1-210421.D	**ACQ Method**	ALI-IMRAN-140421.M	**Comment**		**Acquired Time**	4/21/2021 2:42:58 PM

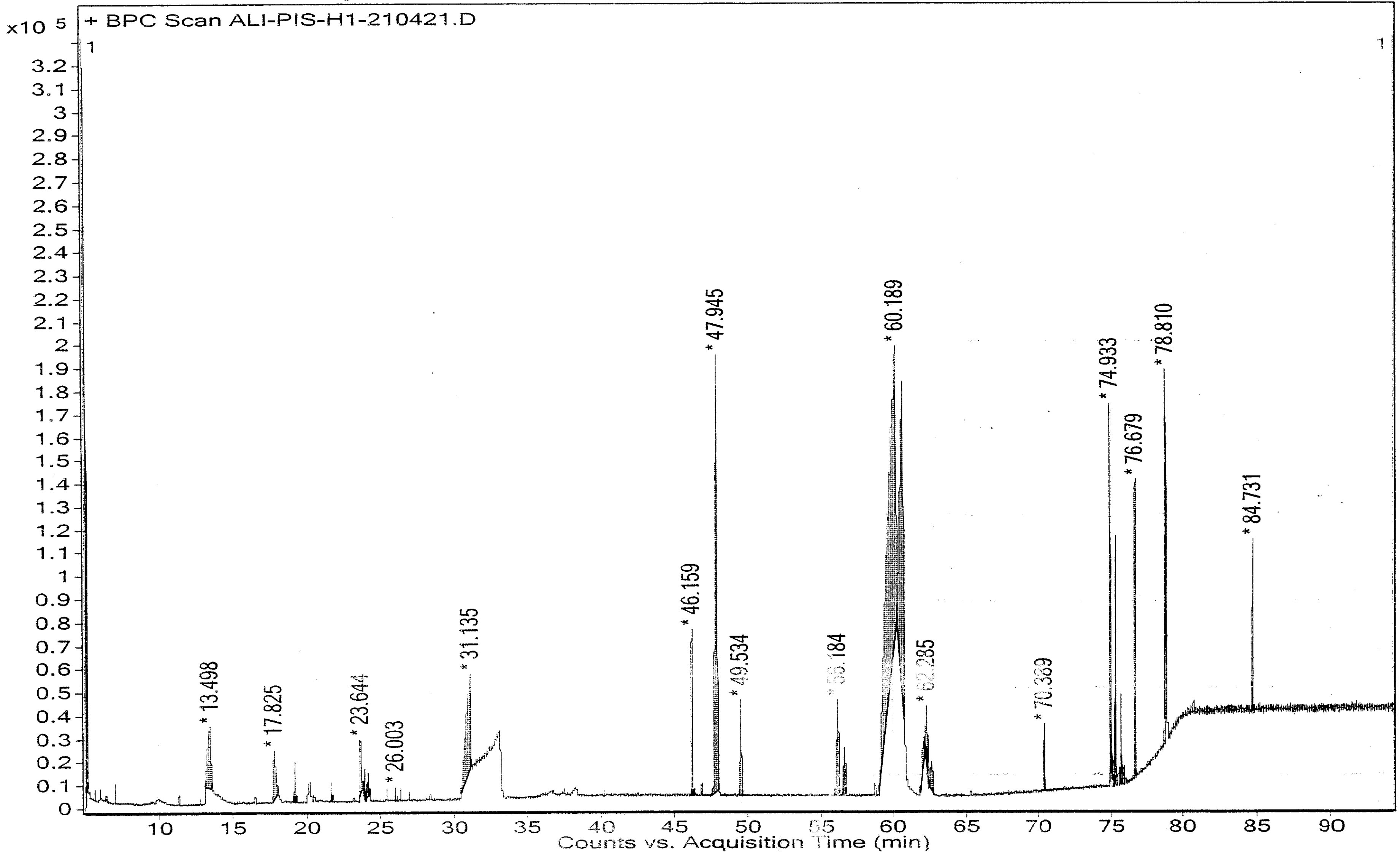

**Sample Name**	ALI-PIS-H1-210421	**Position**	12	**Instrument Name**	GCMS TQQQ	**User Name**	HEJ-G-104-03\Agilent
**Inj Vol**	1	**InjPosition**		**SampleType**		**IRM Calibration Status**	Not Applicable
**Data Filename**	ALI-PIS-H1-210421.D	**ACQ Method**	ALI-IMRAN-140421.M	**Comment**		**Acquired Time**	4/21/2021 2:42:58 PM

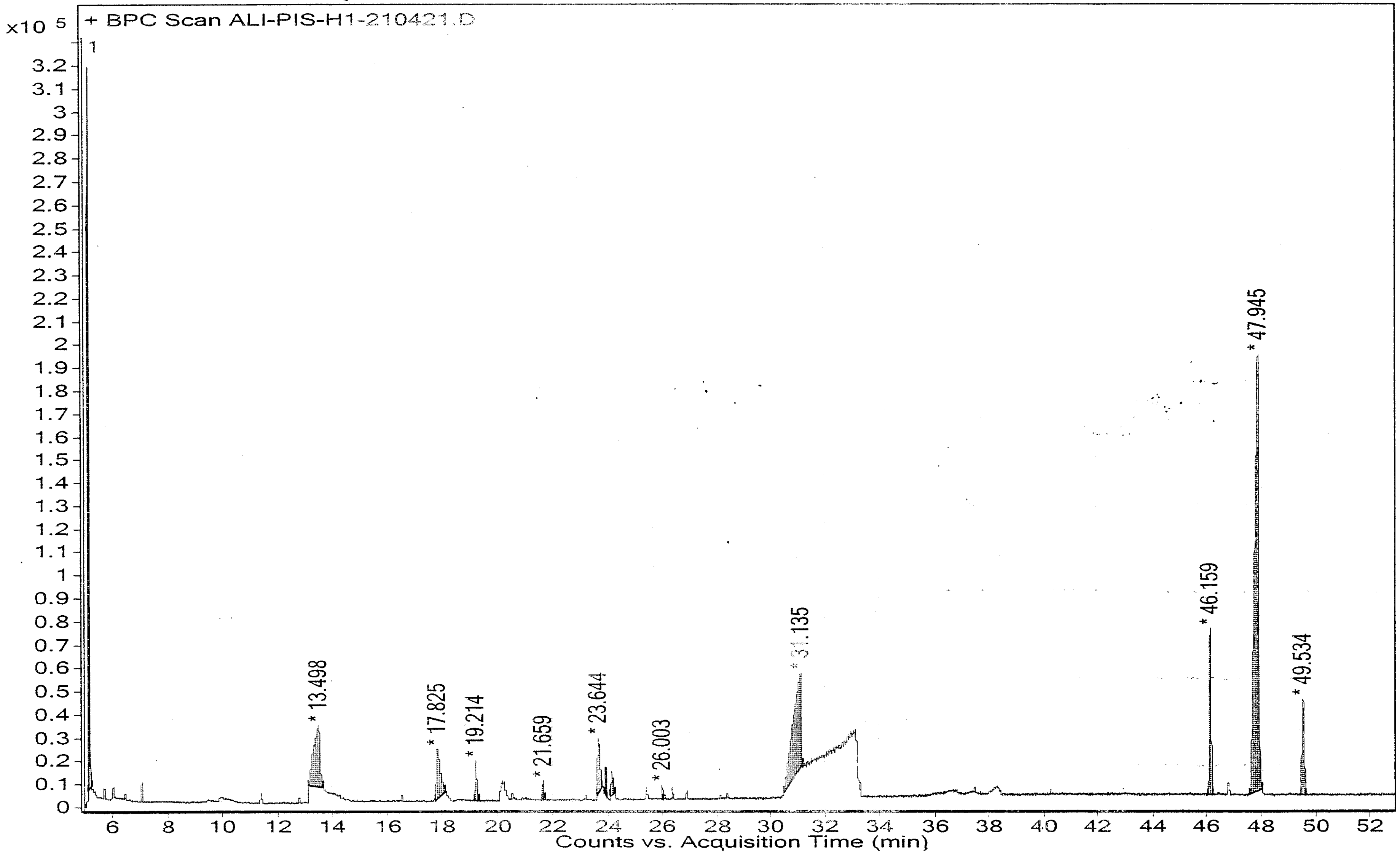

**Sample Name**	ALI-PIS-H1-210421	**Position**	12	**Instrument Name**	GCMS TQQQ	**User Name**	HEJ-G-104-03\Agilent
**Inj Vol**	1	**InjPosition**		**SampleType**		**IRM Calibration Status**	Not Applicable
**Data Filename**	ALI-PIS-H1-210421.D	**ACQ Method**	ALI-IMRAN-140421.M	**Comment**		**Acquired Time**	4/21/2021 2:42:58 PM

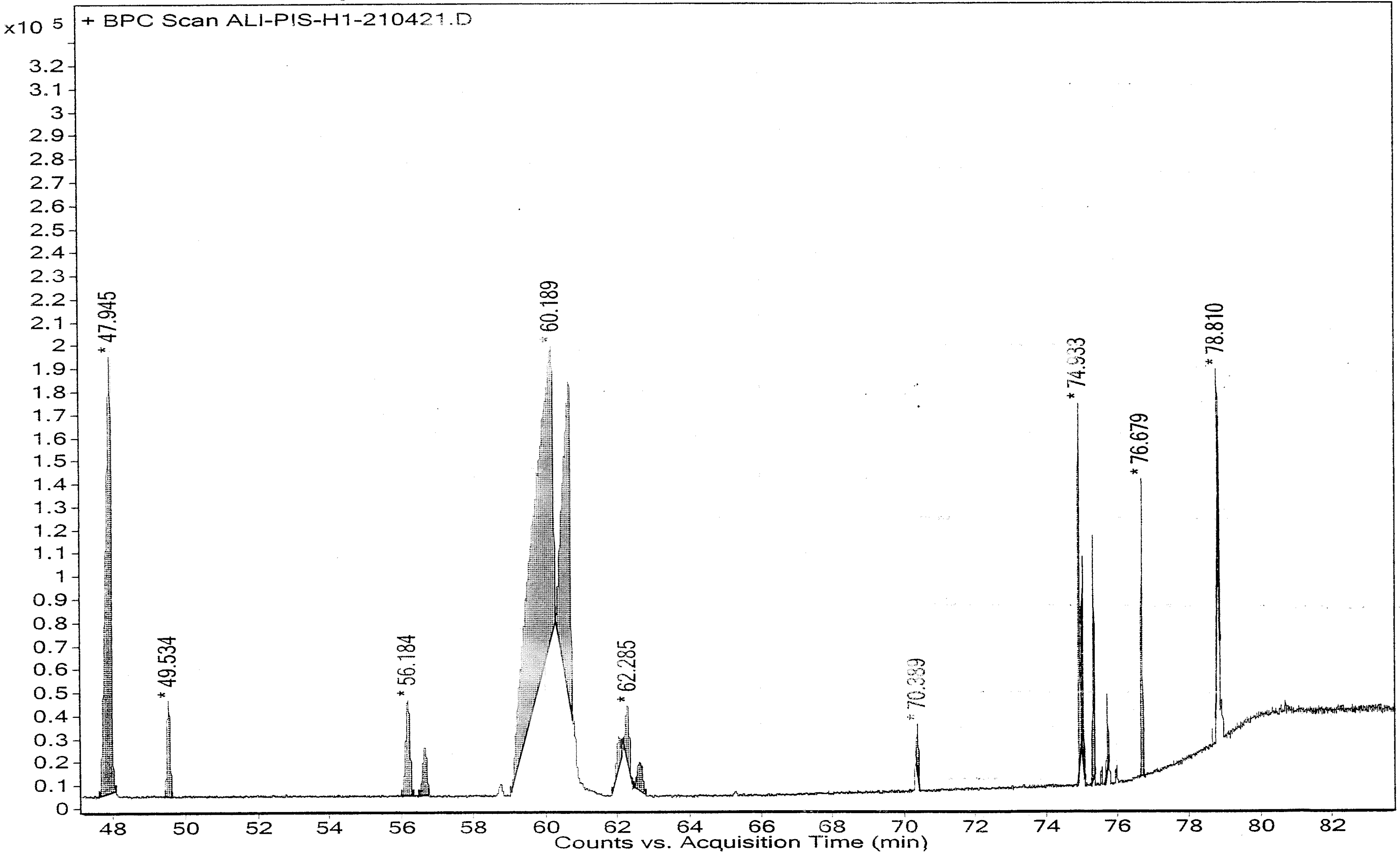

PIS-H1

Peak Number	RT	Area	Area %	Height	Width	Area Sum %	Height %
1	5.156	899253	17.24	311558	0.118	5.81	100
2	13.498	422849	8.1	26614	0.495	2.73	8.54
3	17.825	225671	4.33	21651	0.378	1.46	6.95
4	19.214	66013	1.27	17485	0.157	0.43	5.61
5	21.659	24497	0.47	8203	0.121	0.16	2.63
6	23.644	162104	3.11	24119	0.21	1.05	7.74
7	23.922	33601	0.64	12518	0.082	0.22	4.02
8	24.146	46874	0.9	10059	0.143	0.3	3.23
9	26.003	15507	0.3	6618	0.086	0.1	2.12
10	31.135	913550	17.51	40591	0.709	5.9	13.03
11	46.159	363673	6.97	71483	0.207	2.35	22.94
12	47.945	2034353	38.99	188491	0.438	13.14	60.5
13	49.534	243980	4.68	41234	0.21	1.58	13.23
14	56.184	350813	6.72	41310	0.328	2.27	13.26
15	56.651	170586	3.27	20383	0.285	1.1	6.54
16	60.189	5217392	100	125159	1.251	33.71	40.17
17	60.681	2001417	38.36	138717	0.456	12.93	44.52
18	62.028	108871	2.09	14280	0.271	0.7	4.58
19	62.285	176272	3.38	25055	0.221	1.14	8.04
20	62.602	125250	2.4	12394	0.324	0.81	3.98
21	70.389	73633	1.41	22322	0.096	0.48	7.16
22	74.933	457304	8.76	155143	0.1	2.95	49.8
23	75.025	235814	4.52	86666	0.089	1.52	27.82
24	75.328	298687	5.72	104714	0.096	1.93	33.61
25	75.699	78431	1.5	33155	0.096	0.51	10.64
26	76.679	345632	6.62	127955	0.093	2.23	41.07
27	78.81	143384	2.75	59801	0.064	0.93	19.19
28	78.842	15808	0.3	12121	0.036	0.1	3.89
29	84.731	227563	4.36	72045	0.1	1.47	23.12
						100.01	

5

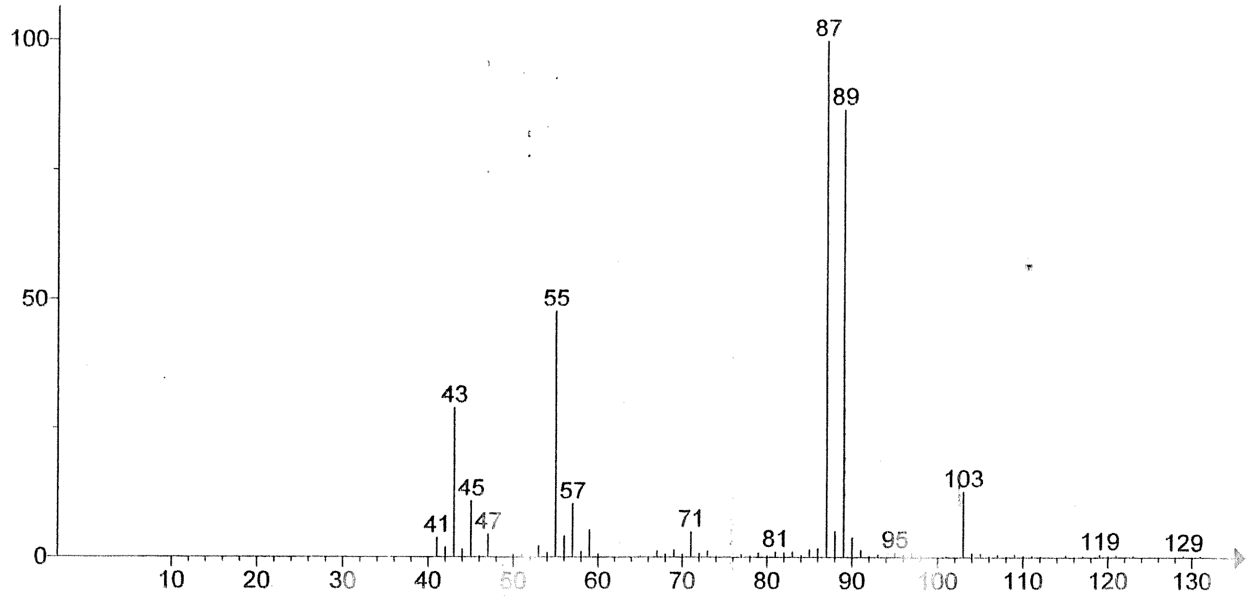

(Text File) +EI Scan (5.156 min) ALI-PIS-H1-210421.D

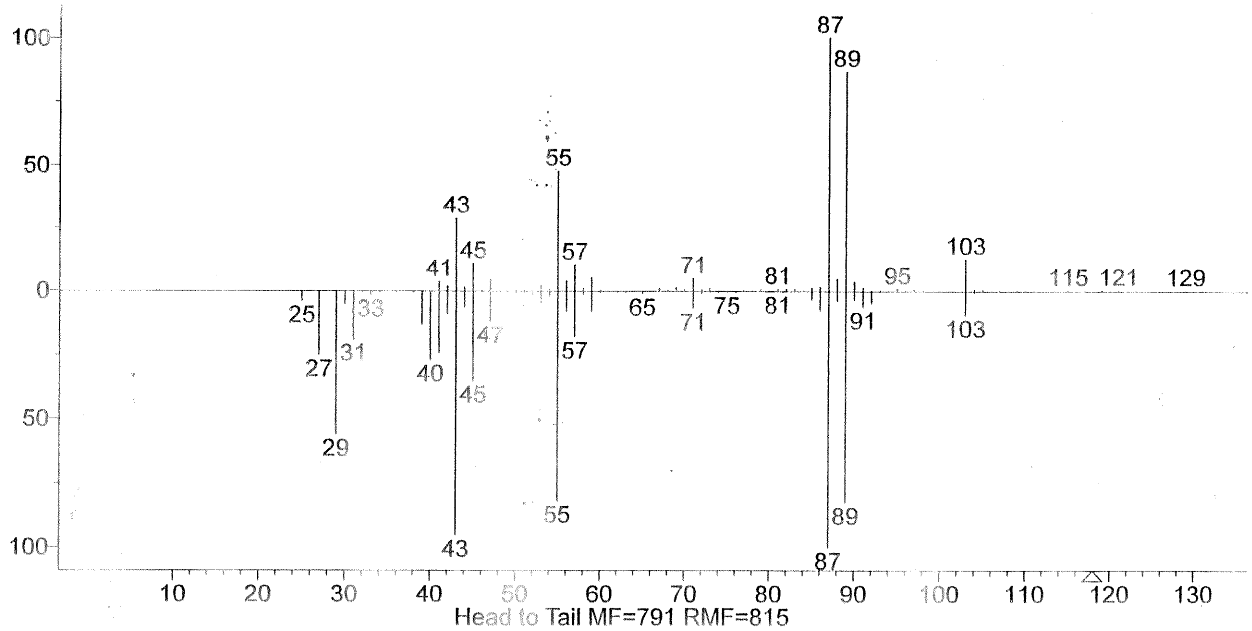

Head to Tail MF=791 RMF=815

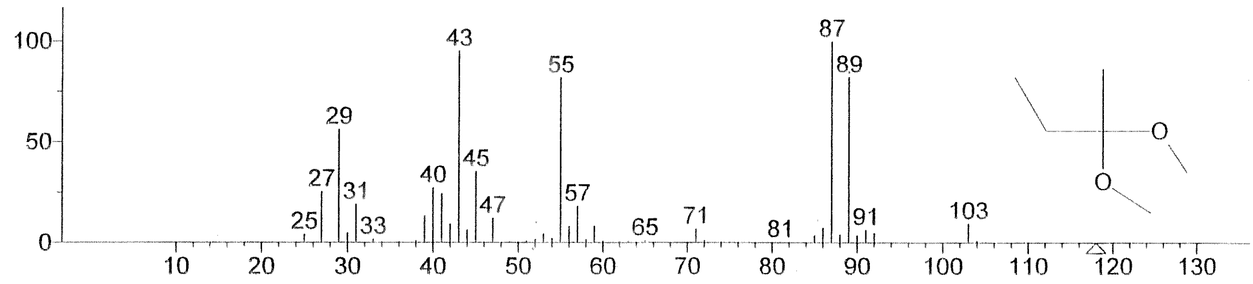

(mainlib) 2,2-Dimethoxybutane

6

Name: 2,2-Dimethoxybutane
Formula: $C_6H_{14}O_2$
MW: 118 CAS#: 3453-99-4 NIST#: 250250 ID#: 48775 DB: mainlib
Other DBs: None
Contributor: TNO Volatile Compounds in Food - Chemical Concepts
10 largest peaks:
87 999 | 43 950 | 89 822 | 55 817 | 29 560 | 45 351 | 40 270 | 27 251 | 41 242 | 31 188 |
Synonyms:
no synonyms.

Estimated non-polar retention index (n-alkane scale):
Value: 685 iu
Confidence interval (Ethers): 68(50%) 293(95%) iu

Retention index.
1. Value: 748.5 iu
Column Type: Capillary
Column Class: Semi-standard non-polar
Active Phase: SE-54
Column
Length: 30 m
Carrier Gas: He
Column Diameter: 0.25 mm
Phase Thickness: 0.25 um
Data Type: Normal
alkane RI
Program Type: Complex
Description: 40C(5min) =>4C/min => 230C(20min)=> 5C/min => 280C (5min)

Source: Gao, H.; Zhao, T.; Kong, Q.; Chen, X.; Hu, Z., Analysis of unknown organic pollutants in sewage by solid -phase extraction combined with gas chromatography-mass spectrometry, J. Chromatogr. Sci., 42, 2004, 91-99.

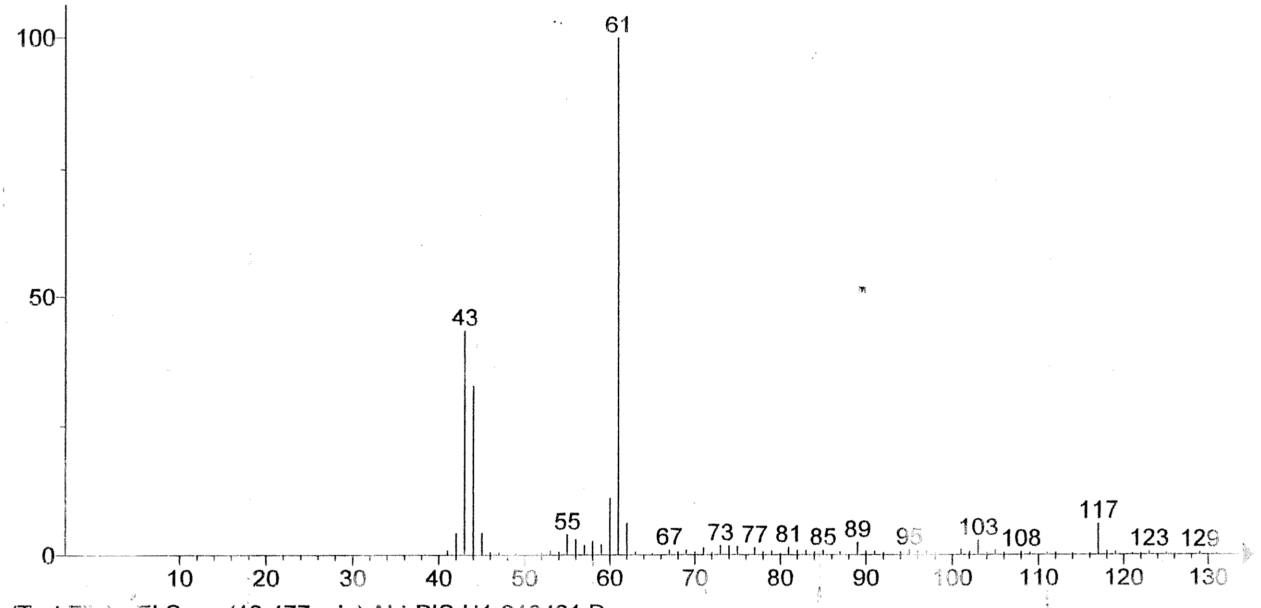

(Text File) +EI Scan (13.477 min) ALI-PIS-H1-210421.D

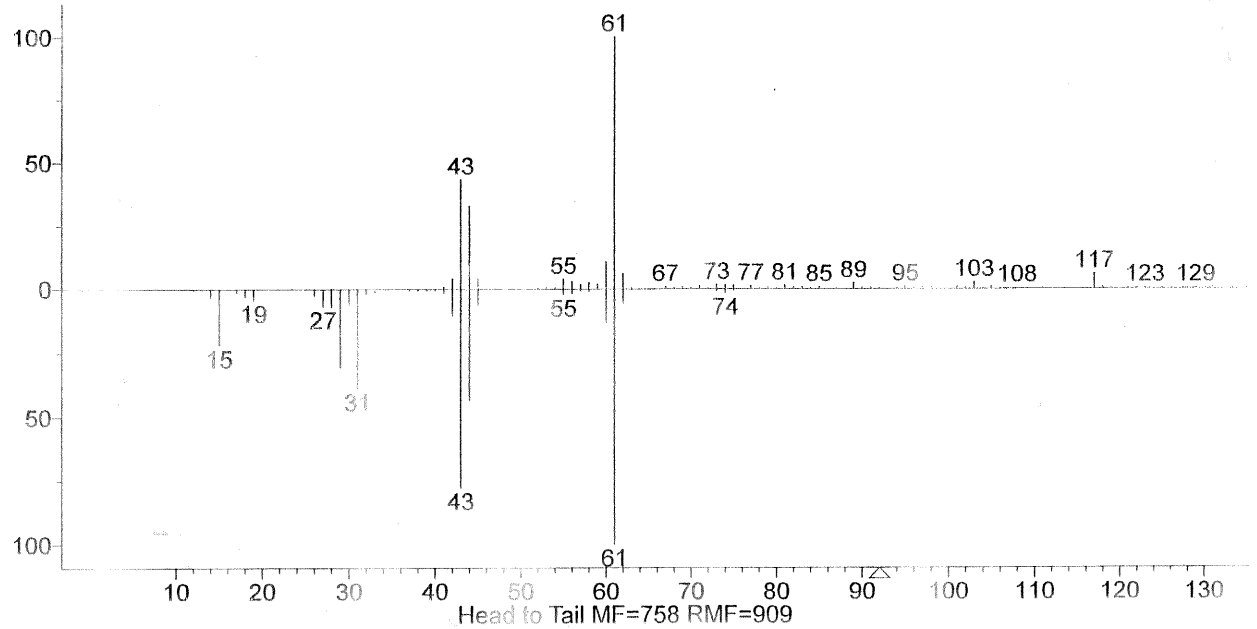

Head to Tail MF=758 RMF=909

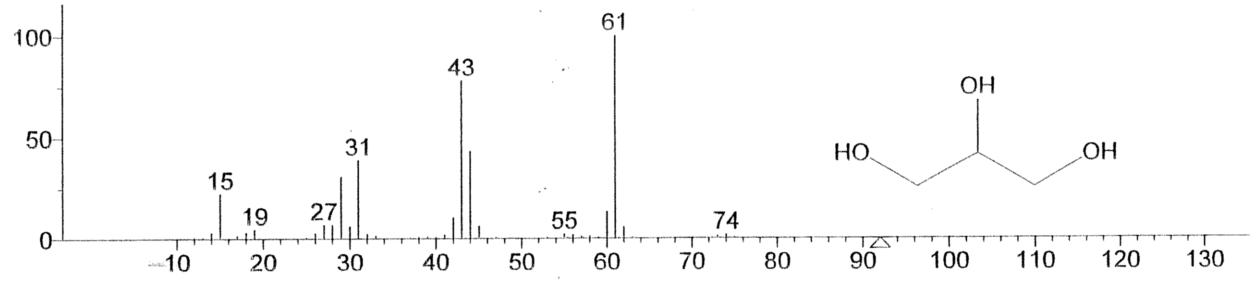

(mainlib) Glycerin

Name: Glycerin
Formula: $C_3H_8O_3$
MW: 92 CAS#: 56-81-5 NIST#: 118748 ID#: 27364 DB: mainlib
Other DBs: Fine, TSCA, RTECS, USP, HODOC, NIH, EINECS, IRDB
Contributor: NIST Mass Spectrometry Data Center, 1990.
10 largest peaks:
61 999 | 43 777 | 44 433 | 31 387 | 29 304 | 15 220 | 60 130 | 42 102 | 27 68 | 28 68 |
Synonyms:
1.1,2,3-Propanetriol
2.Glycerol
3.Glycerine
4.Glyceritol
5.Glycyl alcohol
6.Glyrol
7.Glysanin
8.Osmoglyn
9.Propanetriol
10.Trihydroxypropane
11.Synthetic glycerin
12.90 Technical glycerin
13.Dagralax
14.Glycerin, anhydrous
15.Glycerin, synthetic
16.Ophthalgan
17.Synthetic glycerine
18.Vitrosupos
19.1,2,3-Trihydroxypropane
20.90 Technical glycerine
21.Clyzerin, wasserfrei
22.Grocolene
23.Moon
24.Star
25.Superol
26.Optim
27.Bulbold
28.Cristal
29.Croderol G7000
30.Dynamite glycerin
31.Emery 912
32.Glyceol
33.Glycerin USP
34.Glycon G 100, G 300
35.Incorporation factor, IFP
36.Lye glycerin
37.Polyhydric alcohol
38.Pricerine 9071
39.Shur-coal FCA

Estimated non-polar retention index (n-alkane scale):
Value: 967 iu
Confidence interval (Alcohols): 41(50%) 176(95%) iu

Retention index.
1. Value: 940 iu

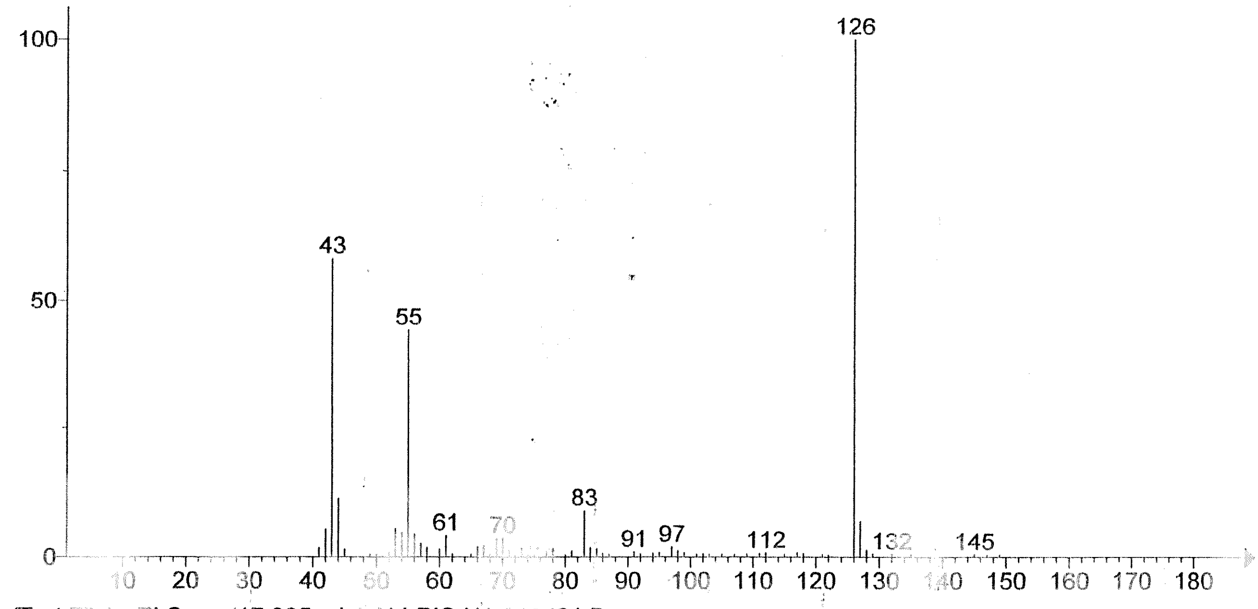

(Text File) +EI Scan (17.835 min) ALI-PIS-H1-210421.D

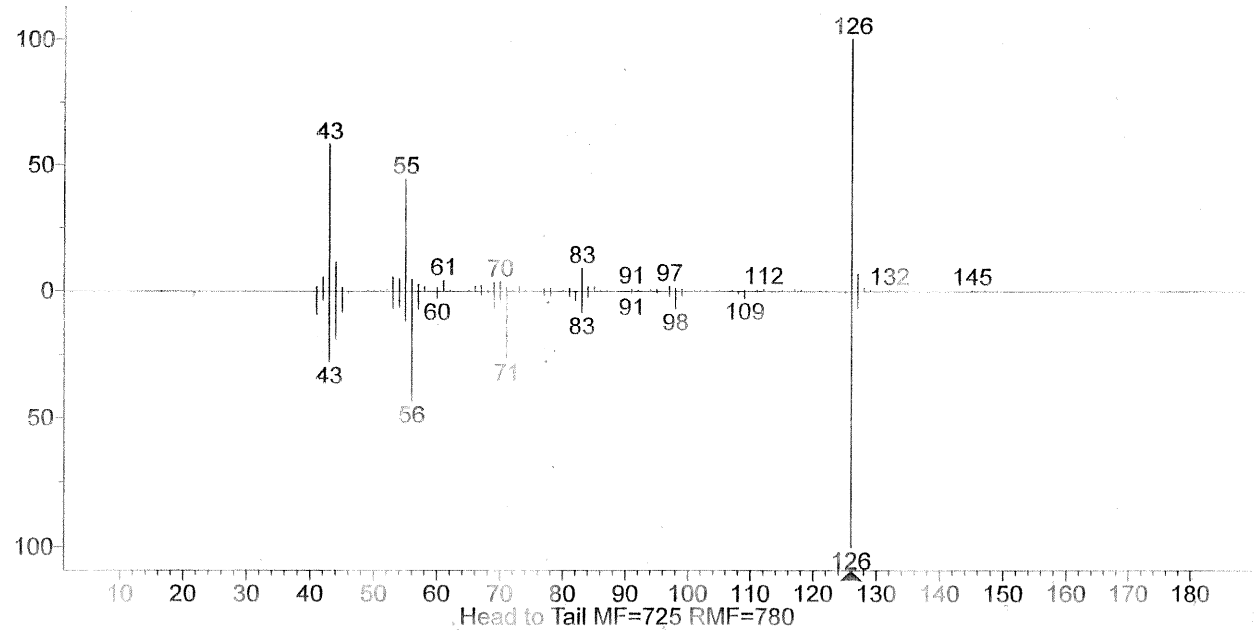

Head to Tail MF=725 RMF=780

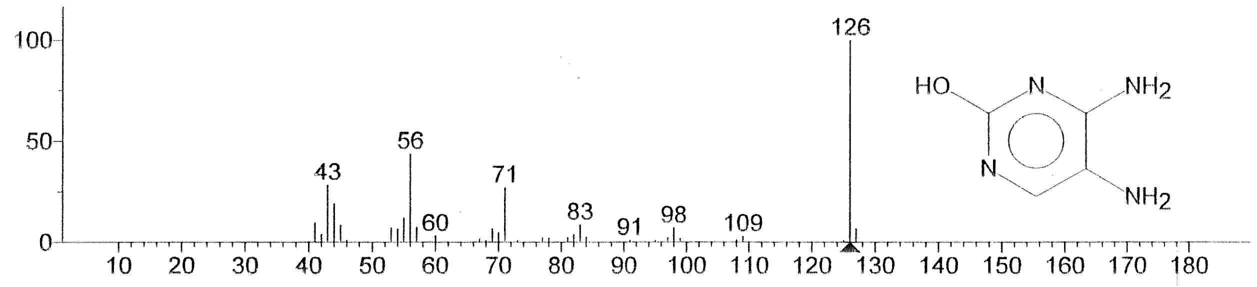

(replib) 4,5-Diamino-2-hydroxypyrimidine

Name: 4,5-Diamino-2-hydroxypyrimidine
Formula: $C_4H_6N_4O$
MW: 126 CAS#: 23899-73-2 NIST#: 127006 ID#: 17324 DB: replib
Other DBs: None
Contributor: LAC, NIDDK, NIH, Bethesda, MD 20892
10 largest peaks:
126 999 | 56 434 | 43 279 | 71 266 | 44 189 | 55 120 | 41 95 | 45 84 | 83 84 | 57 73 |
Synonyms:
1.4,5-Diamino-2-pyrimidinol #

Estimated non-polar retention index (n-alkane scale):
Value: 1512 iu
Confidence interval (Diverse functional groups): 89(50%) 382(95%) iu

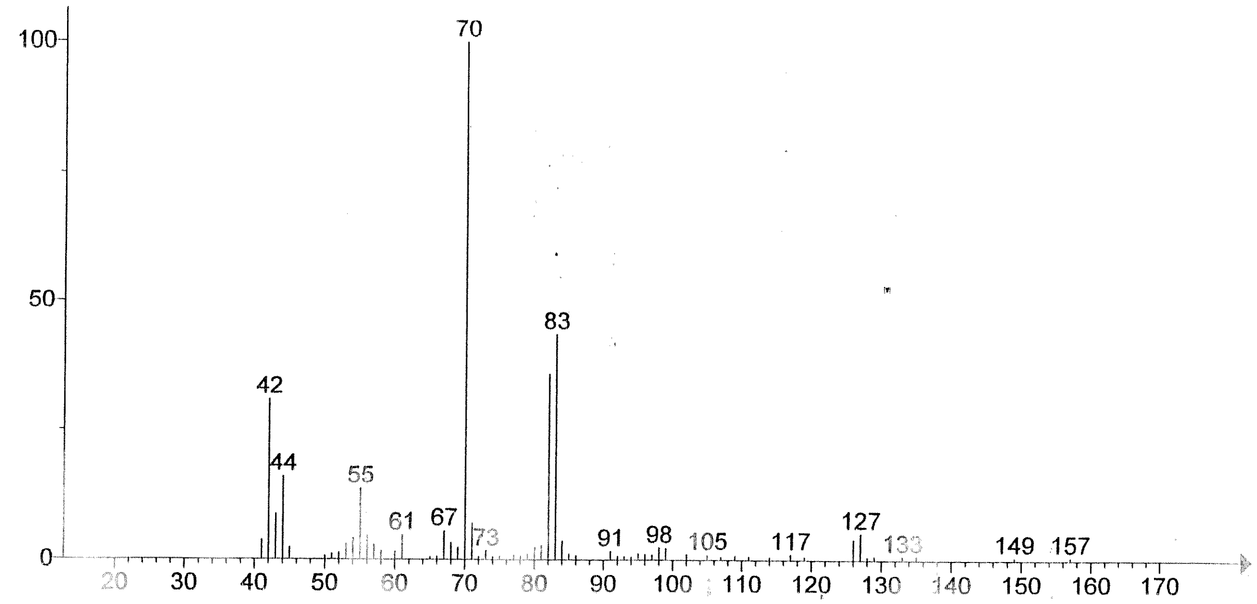

(Text File) +EI Scan (19.221 min) ALI-PIS-H1-210421.D

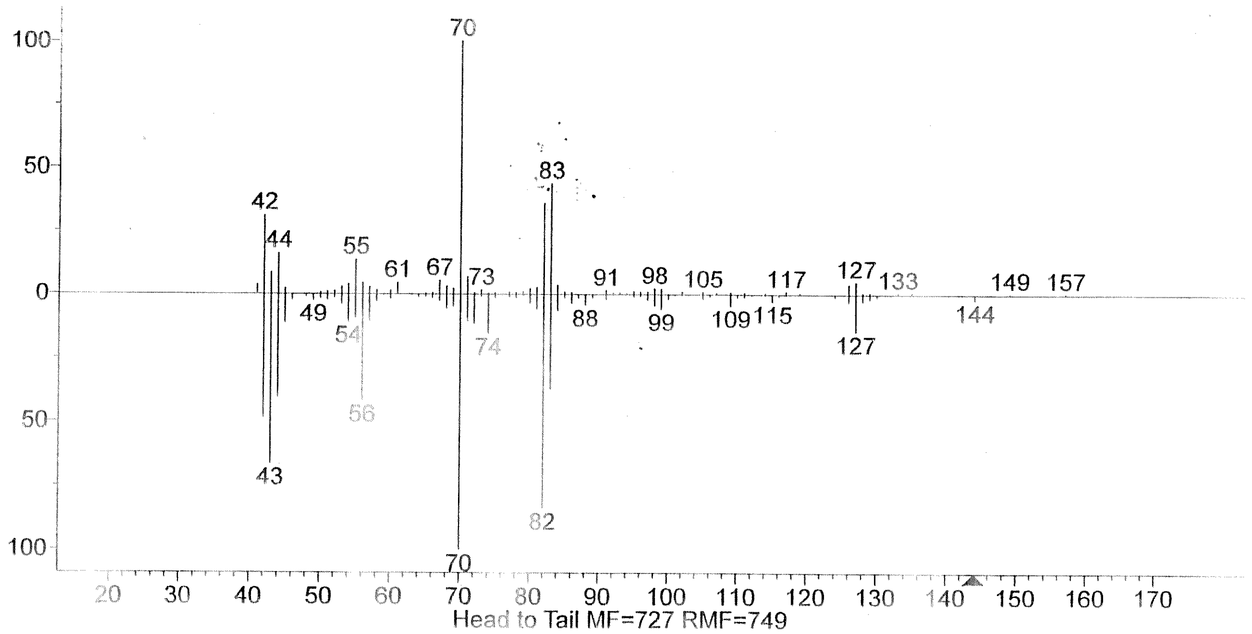

Head to Tail MF=727 RMF=749

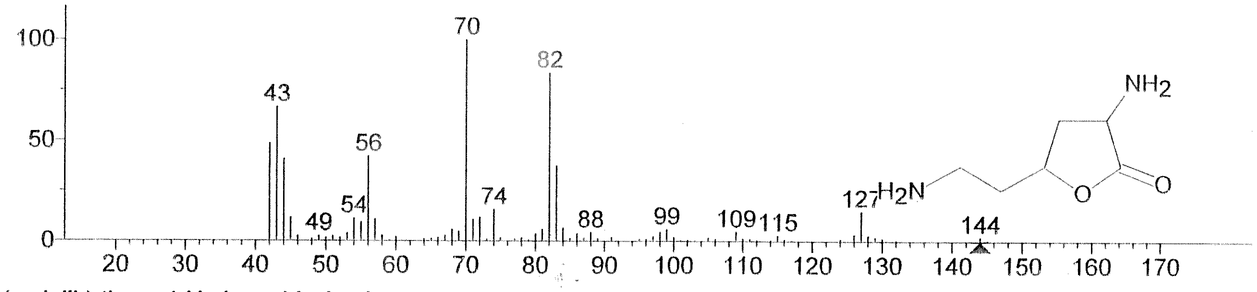

(mainlib) threo-4-Hydroxy-l-lysine lactone

Name: threo-4-Hydroxy-l-lysine lactone
Formula: $C_6H_{12}N_2O_2$
MW: 144 NIST#: 130378 ID#: 32477 DB: mainlib
Contributor: LAC, NIDDK, NIH, Bethesda, MD 20892
10 largest peaks:
70 999 | 82 835 | 43 663 | 42 484 | 56 418 | 44 406 | 83 372 | 74 154 | 127 146 | 72 116 |
Synonyms:
no synonyms.

Estimated non-polar retention index (n-alkane scale):
Value: 1433 iu
Confidence interval (Diverse functional groups): 89(50%) 382(95%) iu

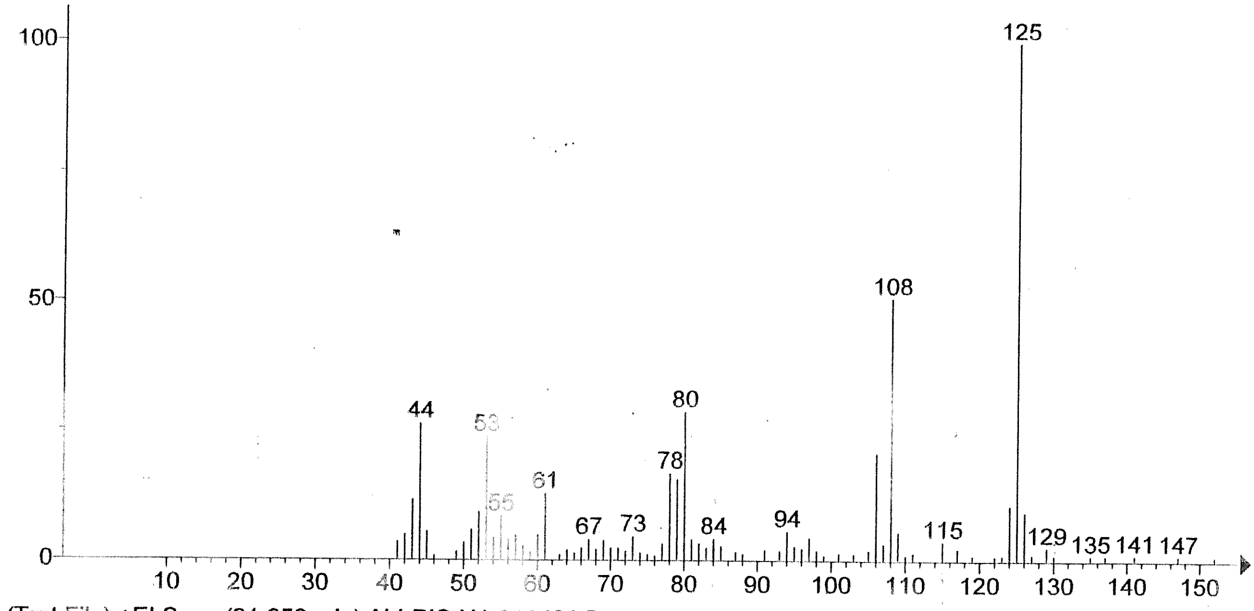

(Text File) +EI Scan (21.659 min) ALI-PIS-H1-210421.D

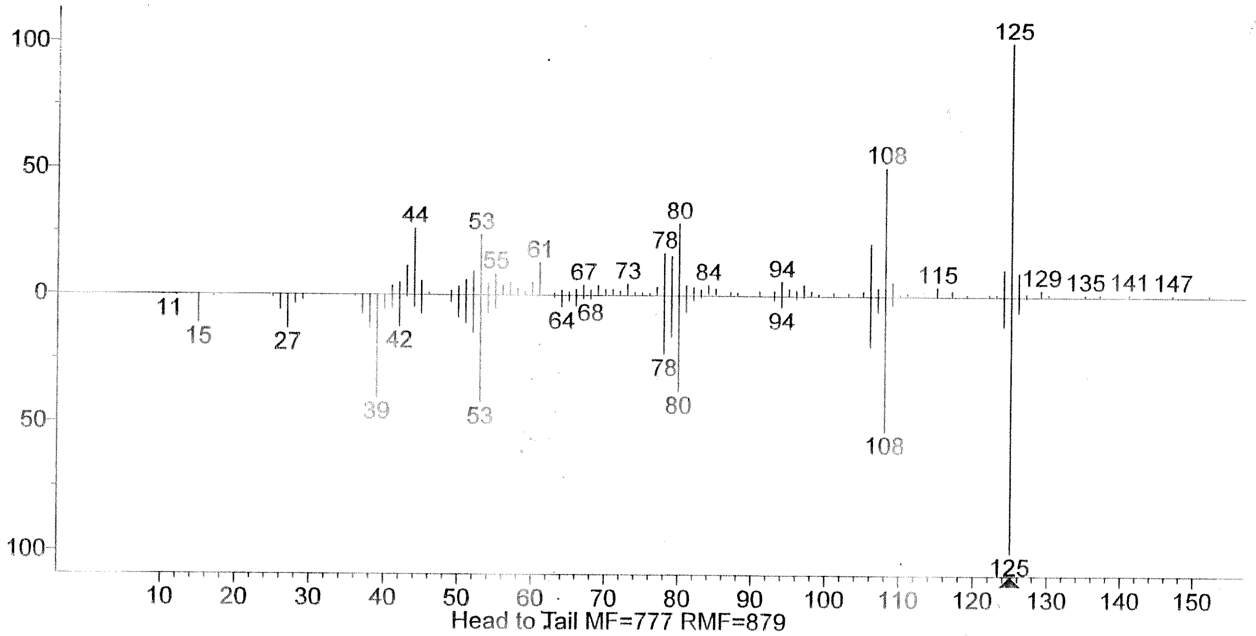

Head to Tail MF=777 RMF=879

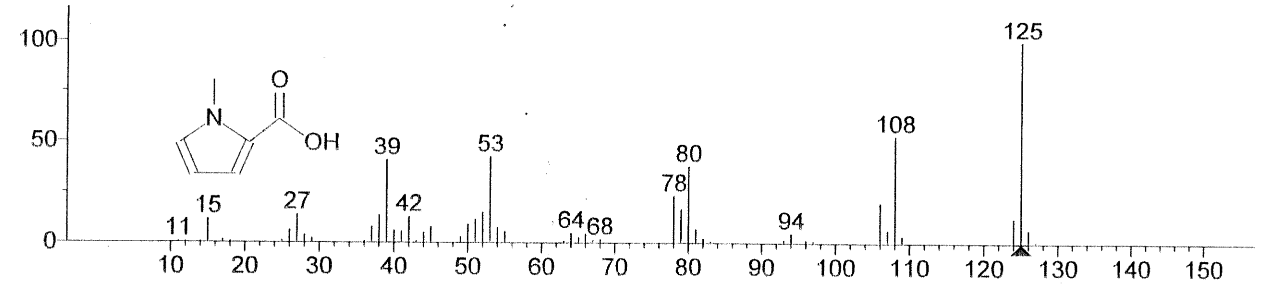

(replib) N-Methylpyrrole-2-carboxylic acid

Name: N-Methylpyrrole-2-carboxylic acid
Formula: $C_6H_7NO_2$
MW: 125 CAS#: 6973-60-0 NIST#: 136662 ID#: 17222 DB: replib
Other DBs: Fine, HODOC, EINECS
Contributor: NIST Mass Spectrometry Data Center, 1994
10 largest peaks:
125 999 | 108 527 | 53 421 | 39 404 | 80 376 | 78 230 | 106 198 | 79 166 | 52 149 | 27 135 |
Synonyms:
1.1H-Pyrrole-2-carboxylic acid, 1-methyl-
2.1-Methyl-1H-pyrrole-2-carboxylic acid #

Estimated non-polar retention index (n-alkane scale):
Value: 1123 iu
Confidence interval (Diverse functional groups): 89(50%) 382(95%) iu

15

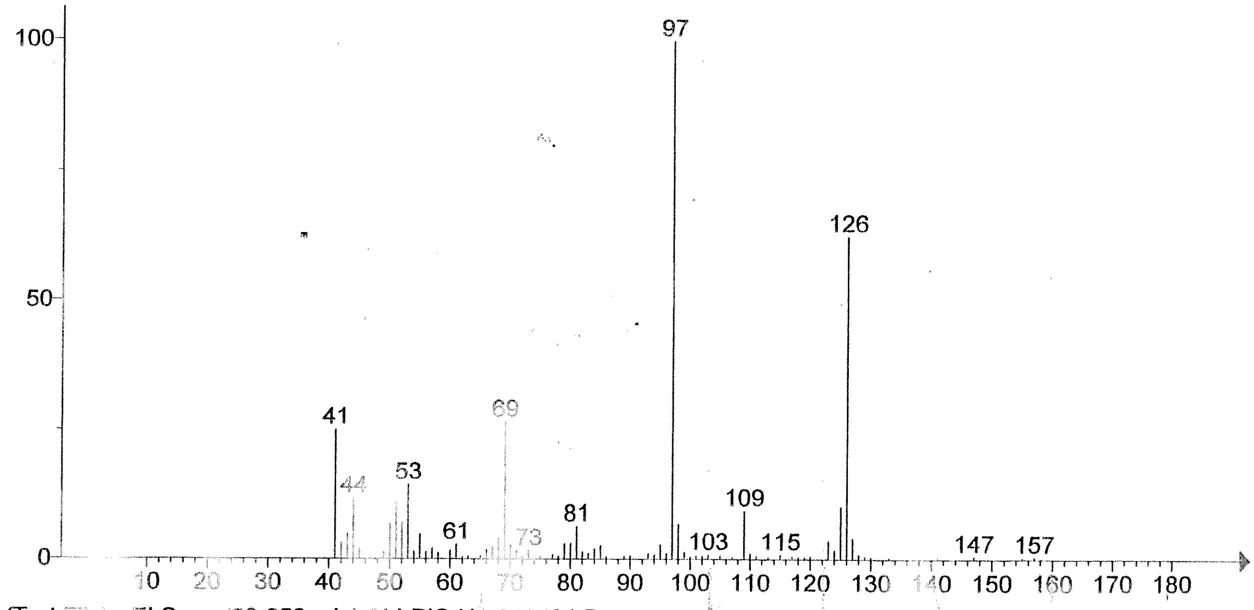

(Text File) +EI Scan (23.658 min) ALI-PIS-H1-210421.D

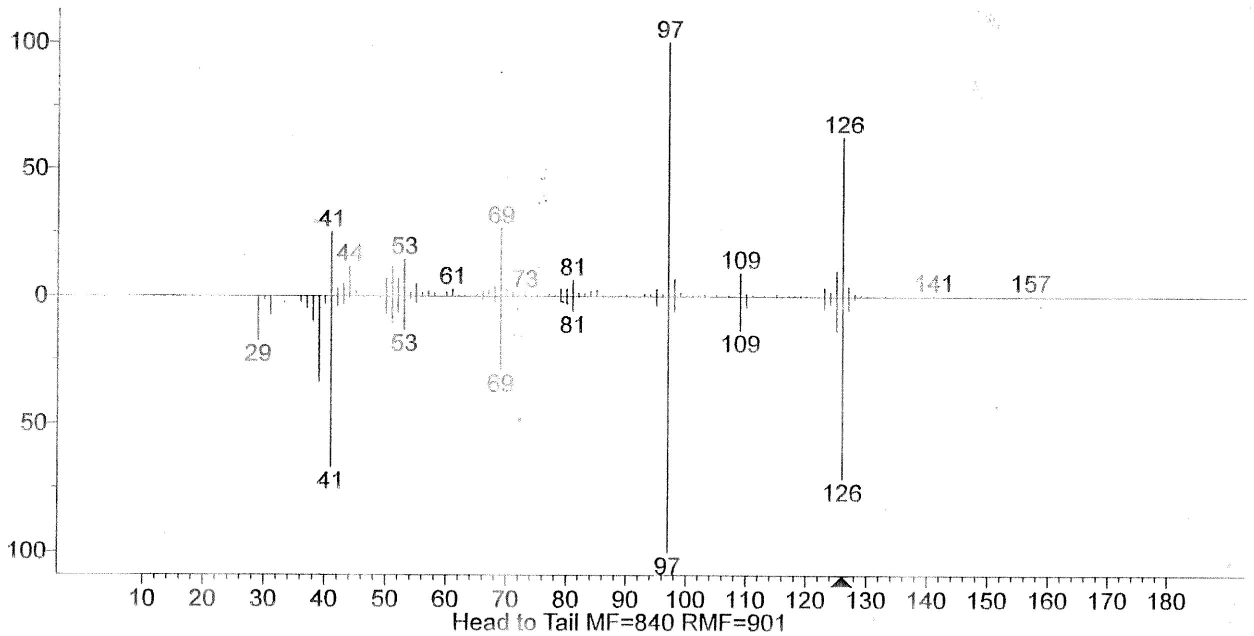

Head to Tail MF=840 RMF=901

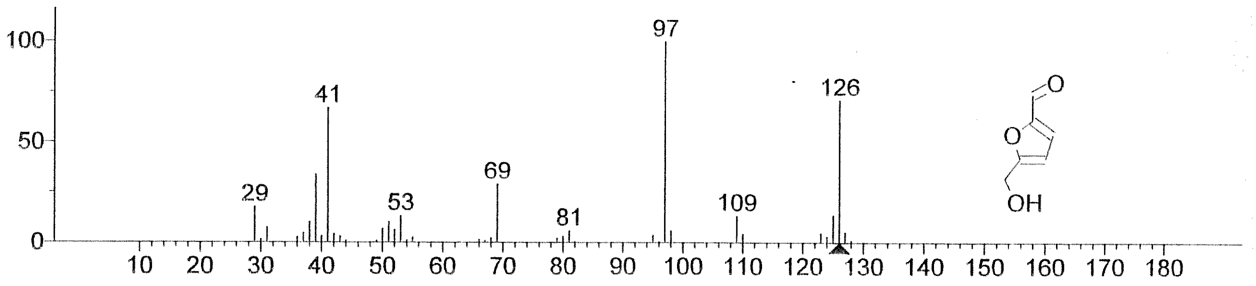

(replib) 2-Furancarboxaldehyde, 5-(hydroxymethyl)-

16

Name: 2-Furancarboxaldehyde, 5-(hydroxymethyl)-
Formula: $C_6H_6O_3$
MW: 126 CAS#: 67-47-0 NIST#: 60544 ID#: 12795 DB: replib
Other DBs: Fine, TSCA, RTECS, HODOC, NIH, EINECS
Contributor: D.HENNEBERG, MAX-PLANCK INSTITUTE, MULHEIM, WEST GERMANY
10 largest peaks:
97 999 | 126 710 | 41 668 | 39 336 | 69 289 | 29 173 | 125 136 | 53 131 | 109 131 | 38 100 |
Synonyms:
1.2-Furaldehyde, 5-(hydroxymethyl)-
2.5-Hydrxoymethylfurfural
3.Hydroxymethylfurfurole
4.HMF
5.5-(Hyddroxymethyl)Furfurole
6.5-(Hydroxymethyl)-2-formylfuran
7.5-(Hydroxymethyl)-2-furaldehyde
8.5-(Hydroxymethyl)-2-furancarbonal
9.5-(Hydroxymethyl)-2-furfural
10.5-(Hydroxymethyl)-2-furfuraldehyde
11.5-(Hydroxymethyl)furan-2-aldehyde
12.5-(Hydroxymethyl)furfural
13.5-Hydroxymethylfuraldehyde
14.5-Oxymethylfurfurole
15.5-Hydroxymethylfurfuraldehyde
16.5-Hydroxymethyl-2-furancarbaldehyde
17.Hydroxymethylfurfuraldehyde
18.5-(Hydroxymethyl)-2-furancarboxaldehyde
19.2-Hydroxymethyl-5-furfural

Estimated non-polar retention index (n-alkane scale):
Value: 1163 iu
Confidence interval (Diverse functional groups): 89(50%) 382(95%) iu

Retention index.
1. Value: 1176 iu
Column Type: Capillary
Column Class: Standard non-polar
Active Phase: DB-1
Column
Length: 60 m
Carrier Gas: N2
Column Diameter: 0.25 mm
Phase Thickness: 1 um
Data Type: Linear
RI
Program Type: Ramp
Start T: 30 C
End T: 200 C
Heat Rate: 5 K/min
End Time: 30 min
Source: Wu, C.-M.;
Wang, Z.; Wu, Q.H., Volatile compounds produced from monosodium glutamate in common food cooking, J. Agric. Food Chem., 48, 2000, 2438-2442.

2. Value: 1208 iu

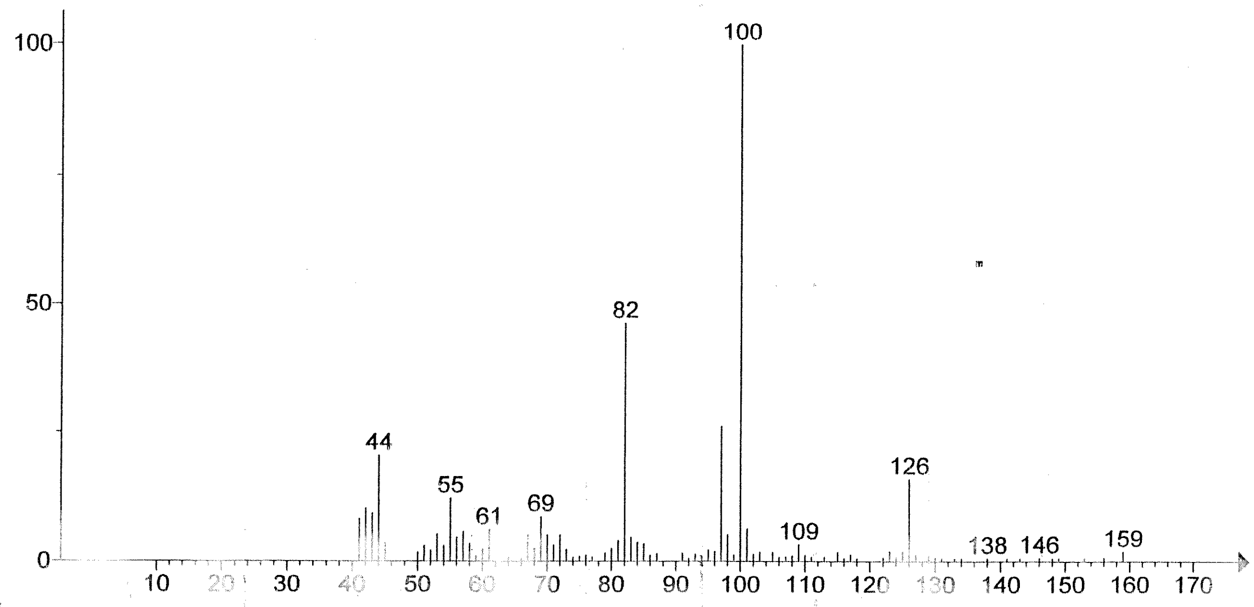

(Text File) +EI Scan (23.918 min) ALI-PIS-H1-210421.D

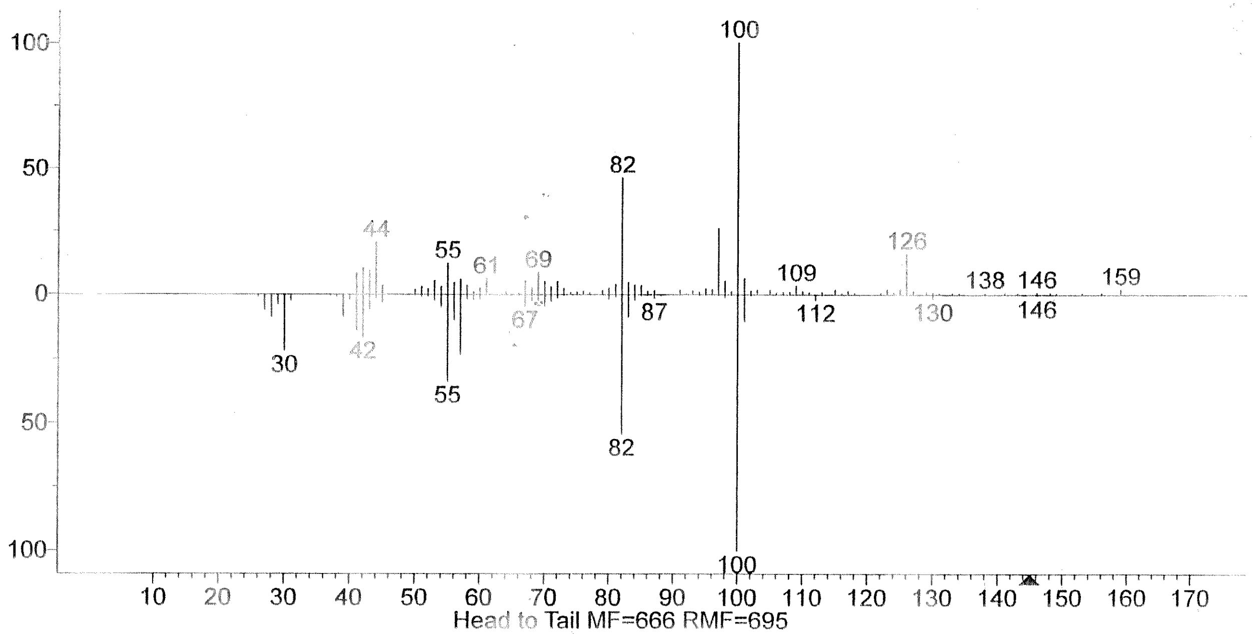

Head to Tail MF=666 RMF=695

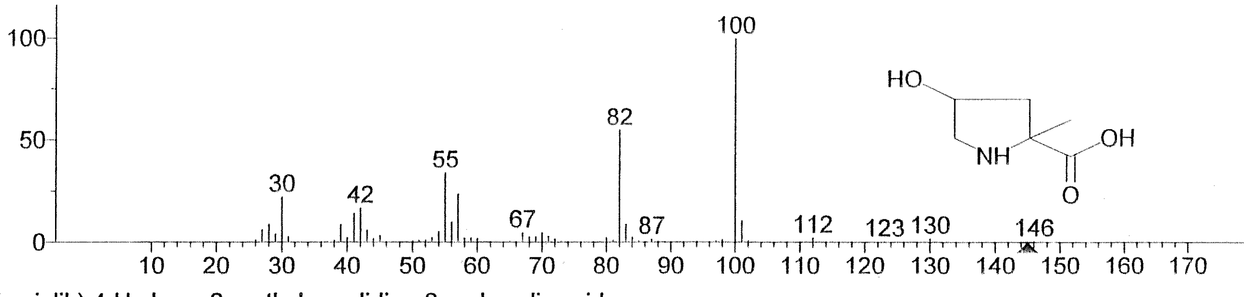

(mainlib) 4-Hydroxy-2-methylpyrrolidine-2-carboxylic acid

Name: 4-Hydroxy-2-methylpyrrolidine-2-carboxylic acid
Formula: $C_6H_{11}NO_3$
MW: 145 NIST#: 191377 ID#: 62684 DB: mainlib
Contributor: Chemical Concepts
10 largest peaks:
100 999 | 82 543 | 55 336 | 57 233 | 30 218 | 42 164 | 41 138 | 101 104 | 56 98 | 28 87 |
Synonyms:
no synonyms.

Estimated non-polar retention index (n-alkane scale):
Value: 1424 iu
Confidence interval (Diverse functional groups): 89(50%) 382(95%) iu

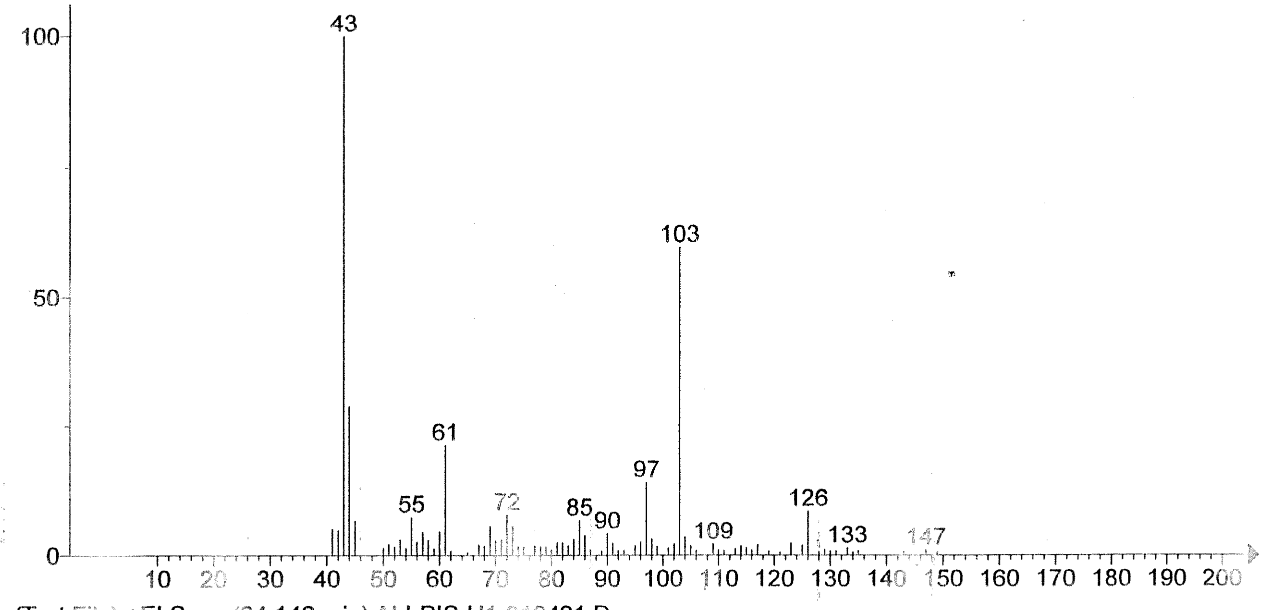

(Text File) +EI Scan (24.146 min) ALI-PIS-H1-210421.D

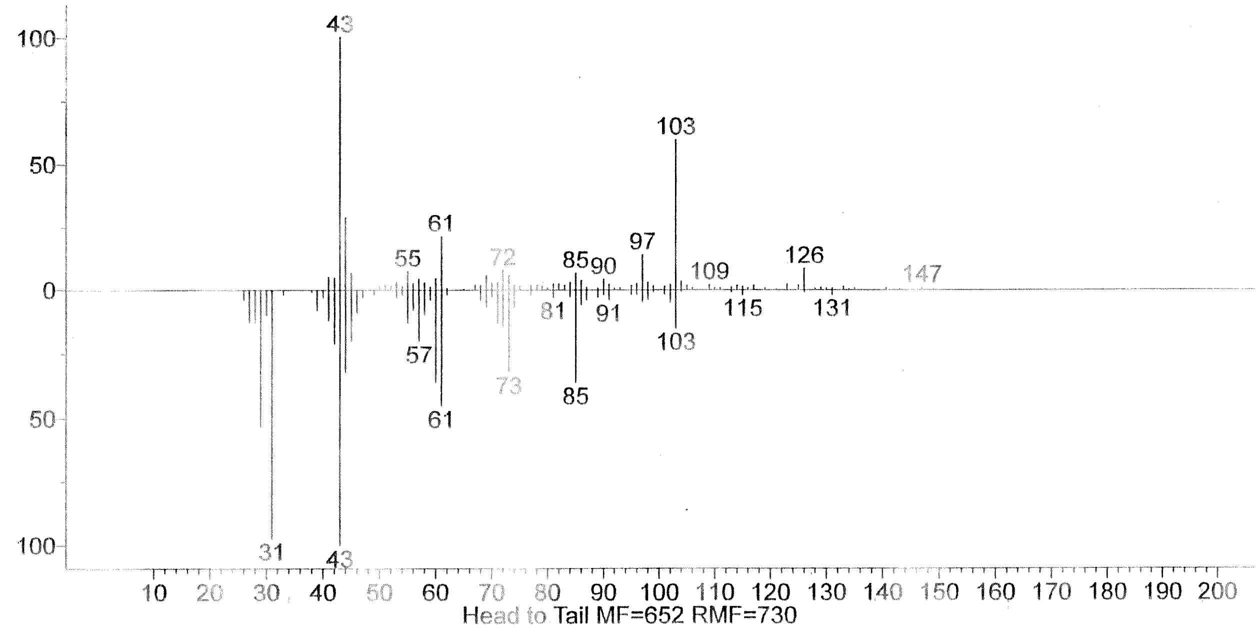

Head to Tail MF=652 RMF=730

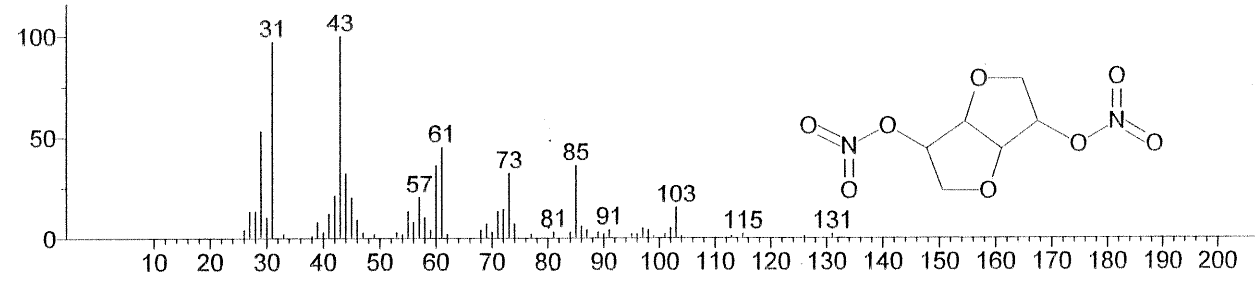

(mainlib) Isosorbide Dinitrate

Name: Isosorbide Dinitrate
Formula: $C_6H_8N_2O_8$
MW: 236 CAS#: 87-33-2 NIST#: 298706 ID#: 5262 DB: mainlib
Other DBs: Fine, TSCA, RTECS, USP, NIH, EINECS
Contributor: R.E.Ardrey ET AL.Pharmaceutical Mass Spectra,L.,1985
10 largest peaks:
43 999 | 31 970 | 29 530 | 61 450 | 60 360 | 85 360 | 44 320 | 73 320 | 42 210 | 45 200 |
Synonyms:
1.D-Glucitol, 1,4:3,6-dianhydro-, dinitrate
2.Cardio 10
3.Cardis
4.Carvanil
5.Carvasin
6.Cedocard
7.Claodical
8.Cornilat
9.Coronex
10.Corosorbide
11.Dianhydrosorbitol 2,5-dinitrate
12.Difutrat
13.Dilatrate-SR
14.Dinitrosorbide
15.Emoper
16.Flindix
17.Glucitol, 1,4:3,6-dianhydro-, dinitrate, D-
18.Harrical
19.iso-Bid
20.Isoket
21.Isorbid
22.Isordil
23.Isordil Tembids
24.Isostat
25.Isotrate
26.IBD 20
27.ISD
28.ISDN
29.Korodil
30.Lomilan
31.Maycor
32.Myorexon
33.Nitrosorbid
34.Nitrosorbide
35.Nitrosorbon
36.Nosim
37.Resoidan
38.Rifloc Retard
39.Rigedal
40.Sorbangil
41.Sorbide nitrate
42.Sorbide T.D.
43.Sorbide, dinitrate
44.Sorbidilat
45.Sorbidnitrate
46.Sorbislo

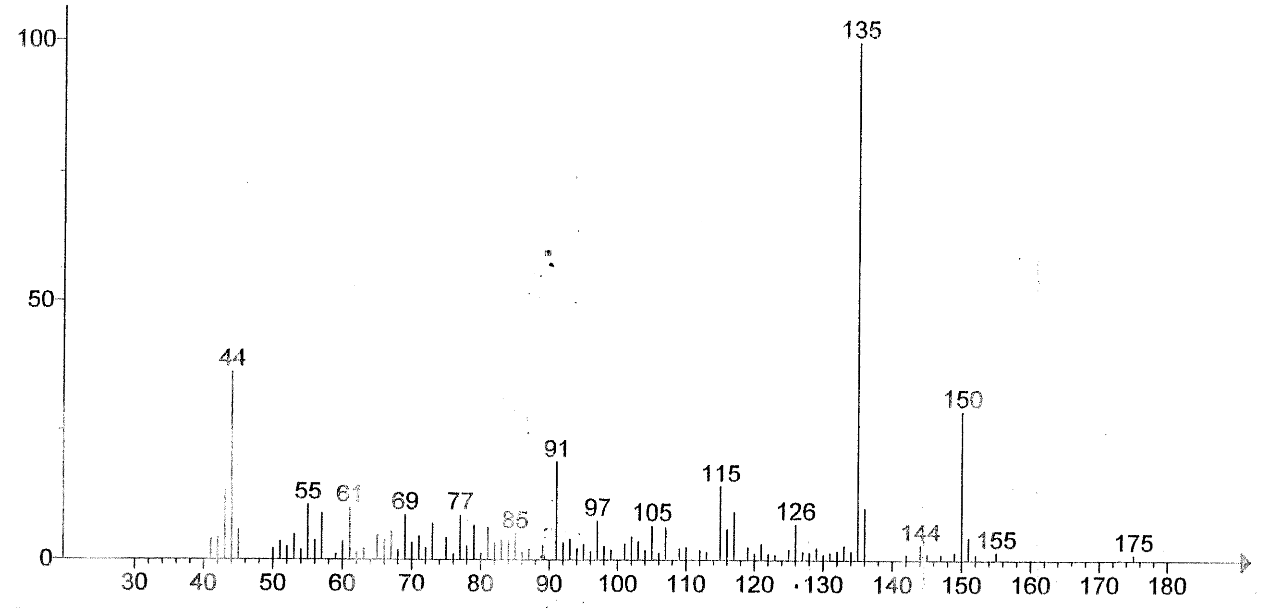

(Text File) +EI Scan (26.003 min) ALI-PIS-H1-210421.D

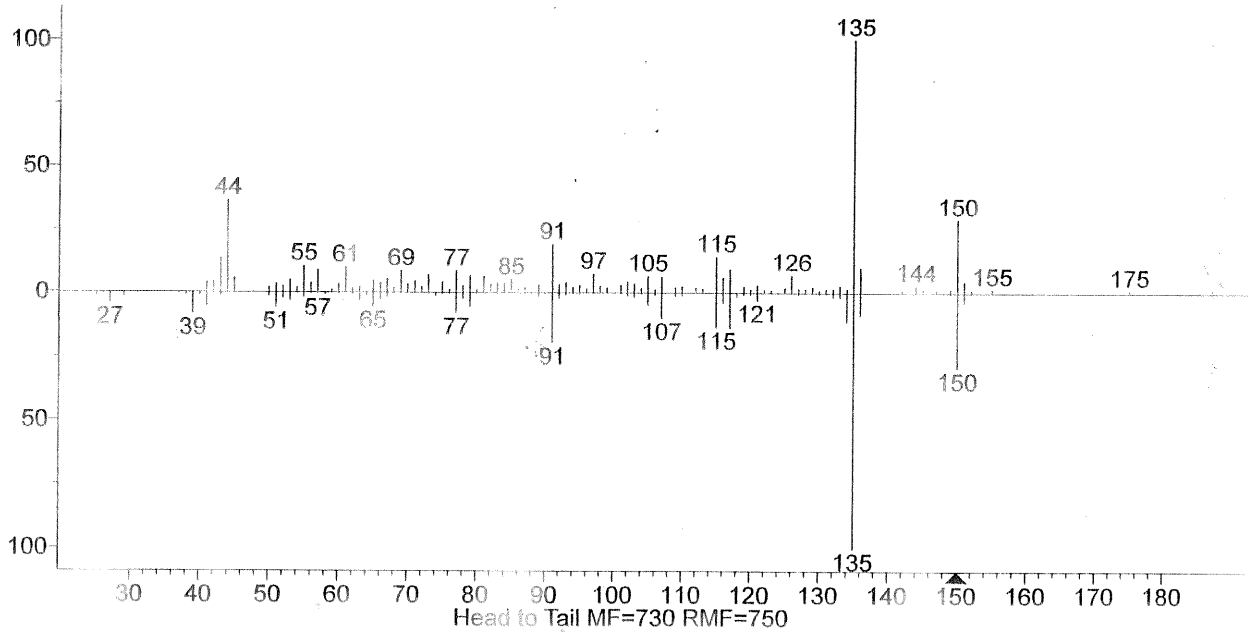

Head to Tail MF=730 RMF=750

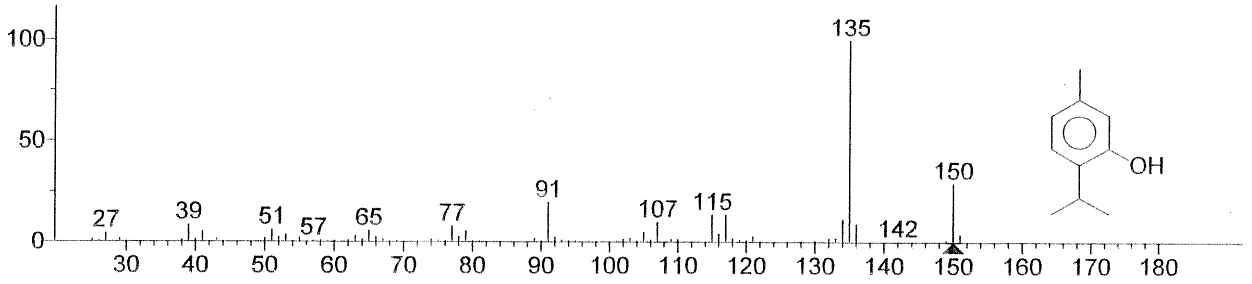

(replib) Thymol

Name: Thymol
Formula: $C_{10}H_{14}O$
MW: 150 CAS#: 89-83-8 NIST#: 191399 ID#: 18428 DB: replib
Other DBs: Fine, TSCA, RTECS, USP, HODOC, NIH, EINECS
Contributor: Chemical Concepts
10 largest peaks:
135 999 | 150 291 | 91 195 | 115 134 | 117 134 | 134 113 | 107 98 | 136 89 | 39 83 | 77 79 |
Synonyms:
1.Phenol, 5-methyl-2-(1-methylethyl)-
2.p-Cymen-3-ol
3.Thyme camphor
4.2-Isopropyl-5-methylphenol
5.3-Hydroxy-p-cymene
6.3-Methyl-6-isopropylphenol
7.5-Methyl-2-isopropylphenol
8.6-Isopropyl-m-cresol
9.6-Isopropyl-3-methylphenol
10.m-Cresol, 6-isopropyl-
11.p-Cymene, 3-hydroxy-
12.Isopropyl cresol
13.Phenol, 2-isopropyl-5-methyl-
14.Thymic acid
15.1-Hydroxy-5-methyl-2-isopropylbenzene
16.1-Methyl-3-hydroxy-4-isopropylbenzene
17.3-p-Cymenol
18.3-Hydroxy-1-methyl-4-isopropylbenzene
19.5-Methyl-2-isopropyl-1-phenol
20.5-Methyl-2-(1-methylethyl)phenol
21.Isopropyl-m-cresol
22.m-Thymol

Estimated non-polar retention index (n-alkane scale):
Value: 1262 iu
Confidence interval (Phenols): 70(50%) 301(95%) iu

Retention index.
1. Value: 1266 iu
Column Type: Capillary
Column Class: Standard non-polar
Active Phase: RTX-1
Column
Length: 60 m
Carrier Gas: He
Column Diameter: 0.22 mm
Phase Thickness: 0.25 um
Data Type: Linear
RI
Program Type: Ramp
Start T: 60 C
End T: 230 C
Heat Rate: 2 K/min
End Time: 30 min
Source: Bendahou,
M.; Muselli, A.; Grignon-Dubois, M.; Benyoucef, M.; Desjobert, J.-M.; Bernardini, A.-F.; Costa, J., Antimicrobial

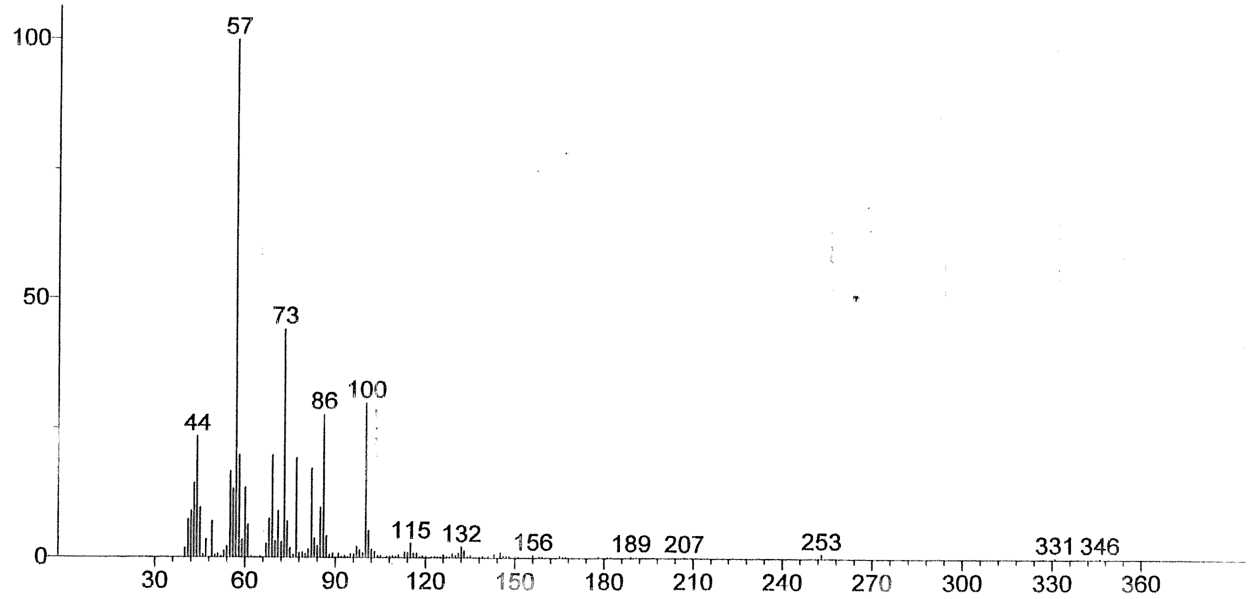

(Text File) +EI Scan (31.096 min) ALI-PIS-H1-210421.D

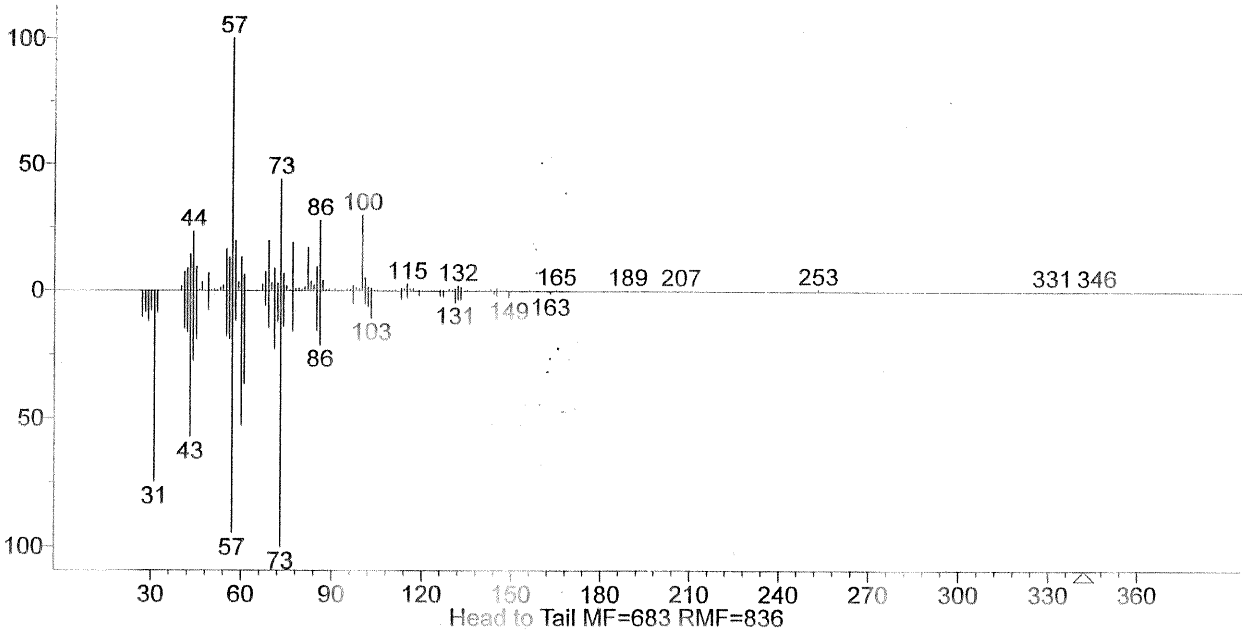

Head to Tail MF=683 RMF=836

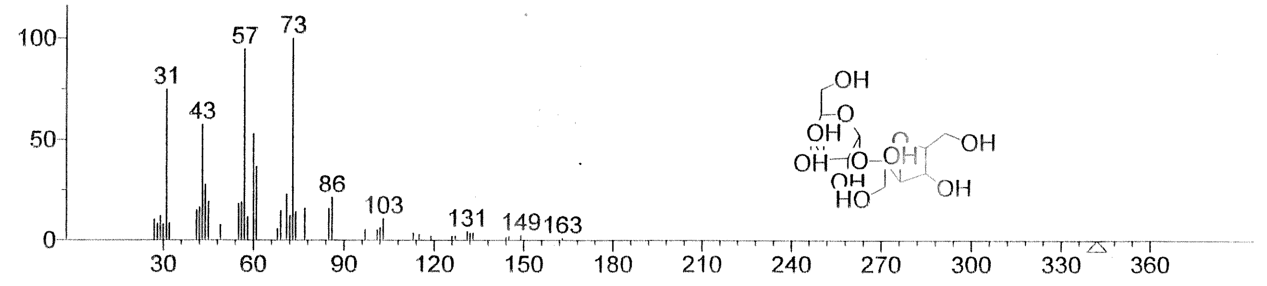

(mainlib) Sucrose

Name: Sucrose
Formula: $C_{12}H_{22}O_{11}$
MW: 342 CAS#: 57-50-1 NIST#: 25039 ID#: 35184 DB: mainlib
Other DBs: Fine, TSCA, RTECS, USP, HODOC, NIH, EINECS
Contributor: M.I.LEVENBERG,S.L.MUELLER,ABBOTT LABS,N.CHICAGO,ILL.60064.USA
10 largest peaks:
73 999 | 57 946 | 31 746 | 43 573 | 60 528 | 61 367 | 44 276 | 71 227 | 86 212 | 45 191 |
Synonyms:
1.α-D-Glucopyranoside, β-D-fructofuranosyl
2.β-D-Fructofuranosyl α-D-glucopyranoside
3.Amerfond
4.Beet sugar
5.Cane sugar
6.Confectioner's sugar
7.D-Sucrose
8.Granulated sugar
9.Microse
10.Rock candy
11.Saccharose
12.Saccharum
13.Sugar
14.White sugar
15.D-(+)-Sucrose
16.D-(+)-Saccharose
17.D-(+)-Saccharose bp ph.eur
18.α-D-Glucopyranosyl β-D-fructofuranoside
19.β-D-Fructofuranoside, α-D-glucopyranosyl
20.(α-D-Glucosido)-β-D-fructofuranoside
21.Fructofuranoside, α-D-glucopyranosyl, β-D
22.Glucopyranoside, β-D-fructofuranosyl, α-D
23.NCI-C56597
24.White sugar Enovit M
25.Sugartab
26.Table sugar
27.Hex-2-ulofuranosyl hexopyranoside #

Estimated non-polar retention index (n-alkane scale):
Value: 3139 iu
Confidence interval (Diverse functional groups): 89(50%) 382(95%) iu

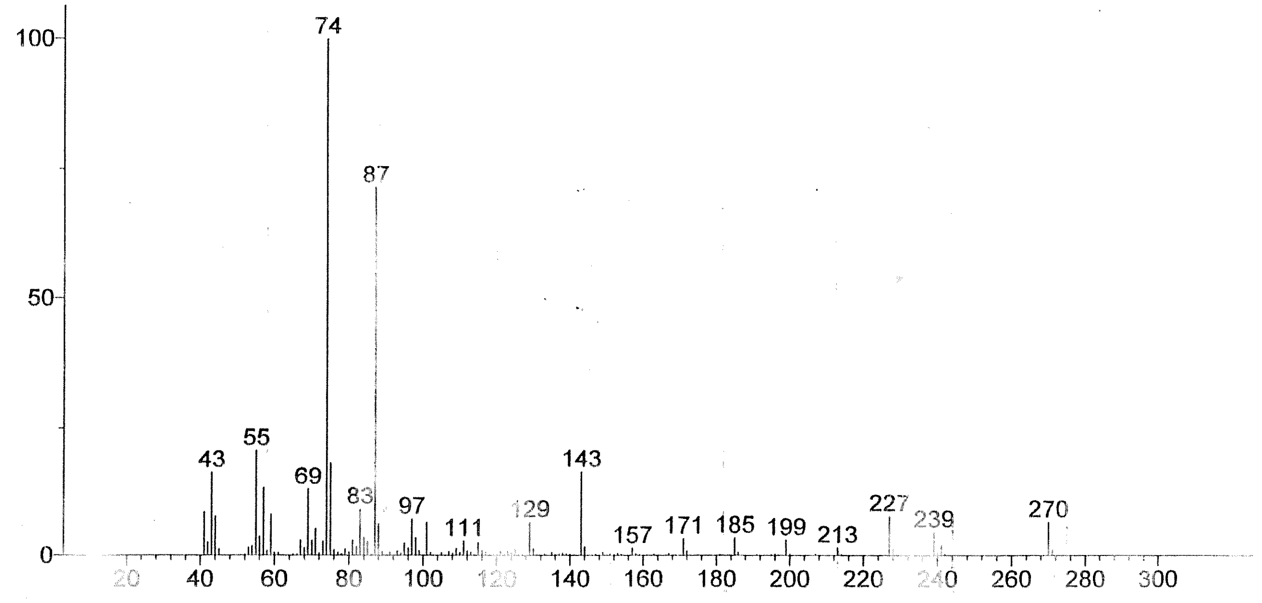

(Text File) +EI Scan (46.159 min) ALI-PIS-H1-210421.D

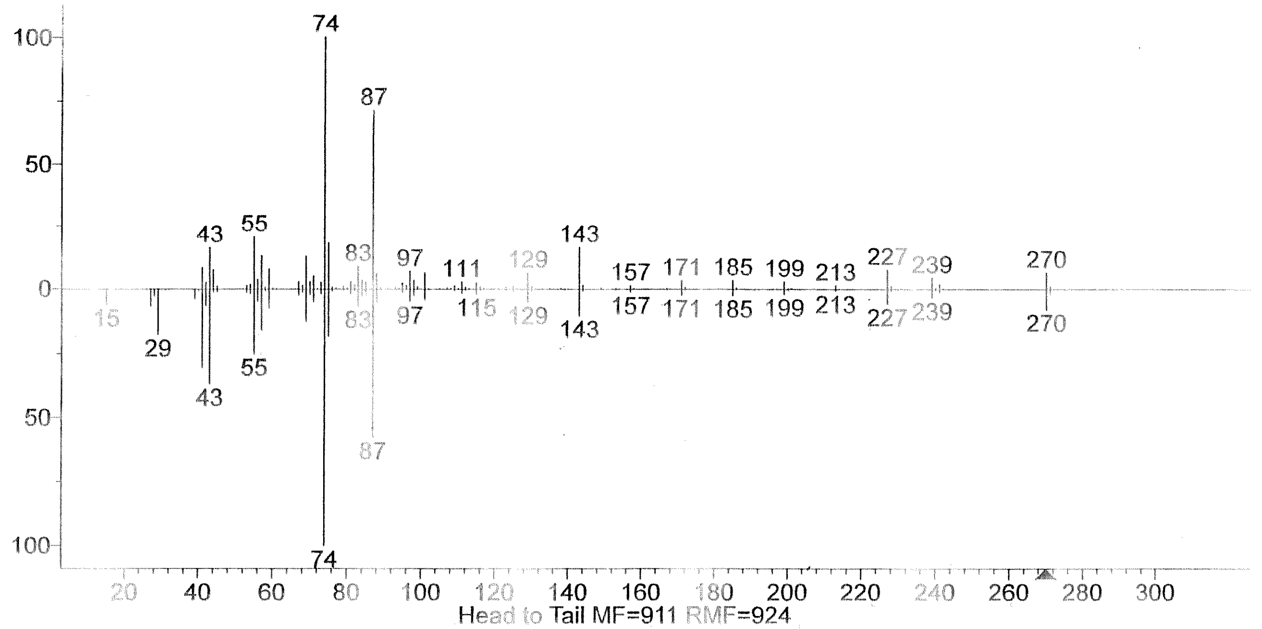

Head to Tail MF=911 RMF=924

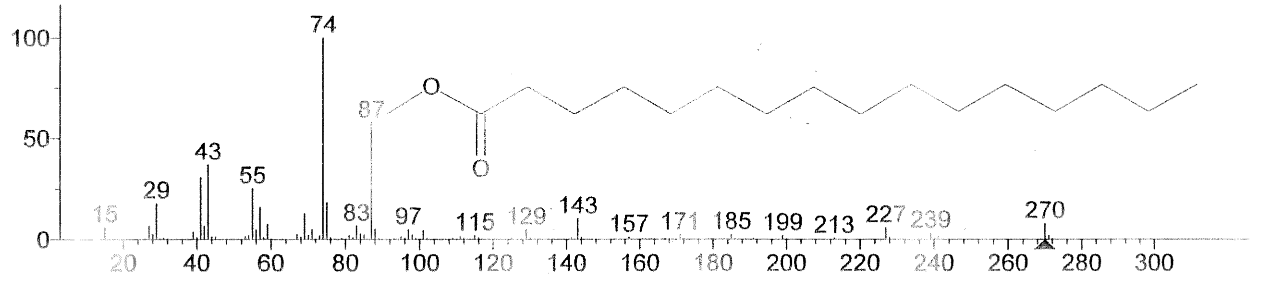

(replib) Hexadecanoic acid, methyl ester

Name: Hexadecanoic acid, methyl ester
Formula: $C_{17}H_{34}O_2$
MW: 270 CAS#: 112-39-0 NIST#: 79124 ID#: 9050 DB: replib
Other DBs: Fine, TSCA, EPA, HODOC, NIH, EINECS
Contributor: O A MAMER, MCGILL UNIVERSITY, MONTREAL, CANADA
10 largest peaks:
74 999 | 87 575 | 43 368 | 41 304 | 55 251 | 75 183 | 29 177 | 57 159 | 69 127 | 143 102 |
Synonyms:
1.Palmitic acid, methyl ester
2.n-Hexadecanoic acid methyl ester
3.Metholene 2216
4.Methyl hexadecanoate
5.Methyl n-hexadecanoate
6.Methyl palmitate
7.Uniphat A60
8.Emery 2216
9.Radia 7120

Estimated non-polar retention index (n-alkane scale):
Value: 1878 iu
Confidence interval (Esters): 47(50%) 201(95%) iu

Retention index.
1. Value: 1908 iu
Column Type: Capillary
Column Class: Standard non-polar
Active Phase: SPB-1
Column
Length: 30 m
Carrier Gas: He
Column Diameter: 0.25 mm
Phase Thickness: 0.25 um
Data Type: Linear
RI
Program Type: Ramp
Start T: 50 C
End T: 250 C
Heat Rate: 5 K/min
Start Time: 3 min
End Time: 15
min
Source: Blagojevic, P.; Radulovic, N.; Palic, R.; Stojanovic, G., Chemical composition of the essential oils of Serbian wild-growing Srtemisia absinthium and Artemisia vulgaris, J. Agric. Food Chem., 54, 2006, 4780-4789.

2.
Value: 1909 iu
Column Type: Capillary
Column Class: Standard non-polar
Active Phase: SPB-1
Column Length:
30 m
Carrier Gas: He
Column Diameter: 0.25 mm
Phase Thickness: 0.25 um

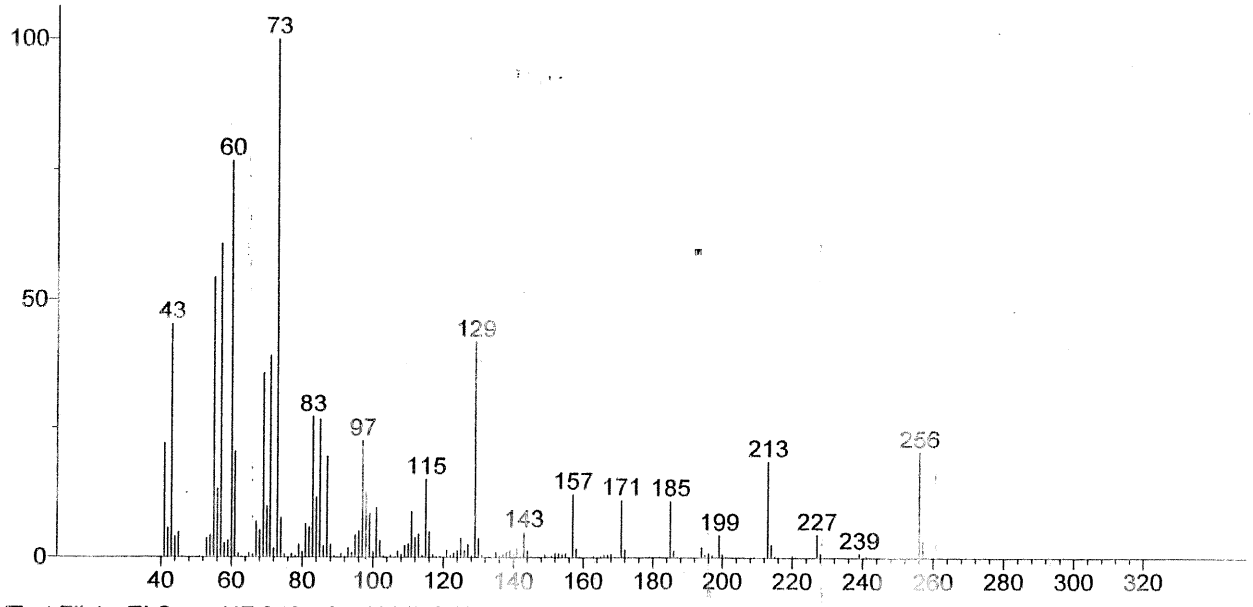

(Text File) +EI Scan (47.948 min) ALI-PIS-H1-210421.D

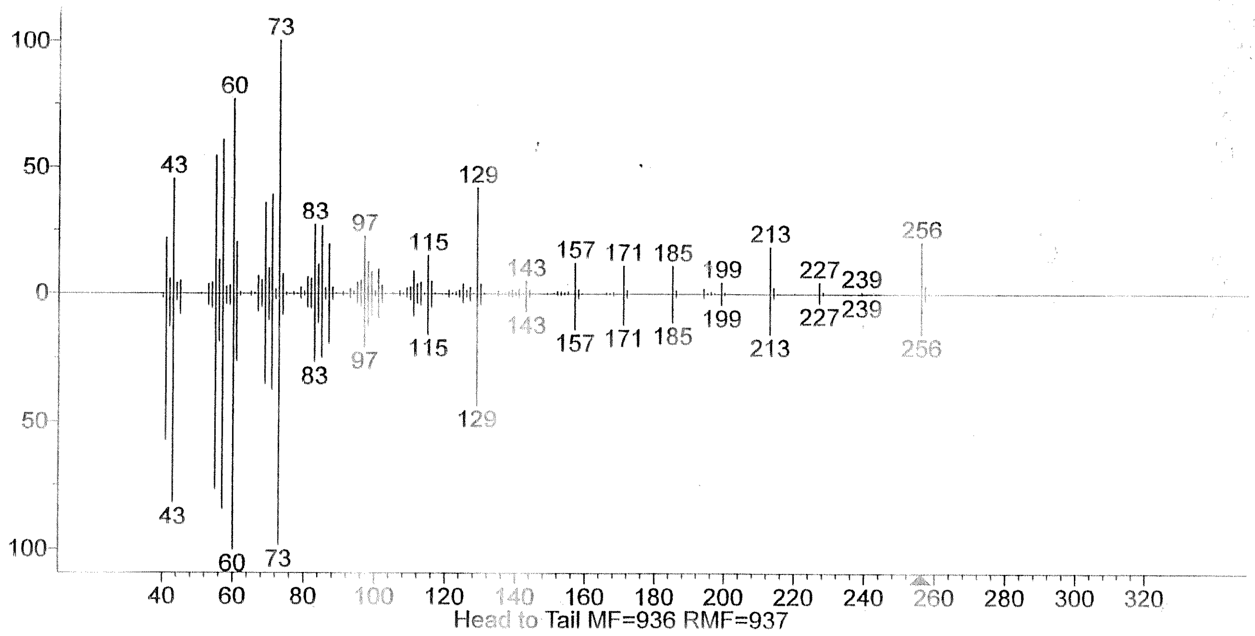

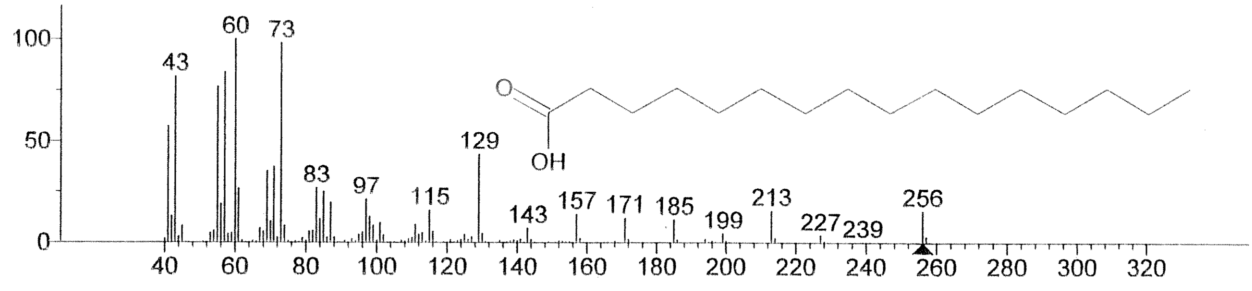

(replib) n-Hexadecanoic acid

Name: n-Hexadecanoic acid
Formula: $C_{16}H_{32}O_2$
MW: 256 CAS#: 57-10-3 NIST#: 335494 ID#: 6723 DB: replib
Other DBs: Fine, TSCA, RTECS, EPA, HODOC, NIH, EINECS, IRDB
Contributor: Drug Lab
10 largest peaks:
60 999 | 73 980 | 57 840 | 43 817 | 55 767 | 41 574 | 129 435 | 71 373 | 69 351 | 83 267 |
Synonyms:
1.Hexadecanoic acid
2.n-Hexadecoic acid
3.Palmitic acid
4.Pentadecanecarboxylic acid
5.1-Pentadecanecarboxylic acid
6.Cetylic acid
7.Emersol 140
8.Emersol 143
9.Hexadecylic acid
10.Hydrofol
11.Hystrene 8016
12.Hystrene 9016
13.Industrene 4516
14.Prifrac 2960
15.Glycon P-45
16.Prifac 2960
17.Univol U332

Estimated non-polar retention index (n-alkane scale):
Value: 1968 iu
Confidence interval (Carboxylic acids): 51(50%) 220(95%) iu

Retention index.
1. Value: 1942 iu
Column Type: Capillary
Column Class: Standard non-polar
Active Phase: RTX-1
Column
Length: 60 m
Carrier Gas: He
Column Diameter: 0.22 mm
Phase Thickness: 0.25 um
Data Type: Linear
RI
Program Type: Ramp
Start T: 60 C
End T: 230 C
Heat Rate: 2 K/min
End Time: 35 min
Source: Paolini, J.;
Muselli, A.; Bernardini, A.-F.; Bighelli, A.; Casanova, J.; Costa, J., Thymol derivatives from essential oil of Doronicum corsicum L., Flavour Fragr. J., 22, 2007, 479-487.

2. Value: 1972 iu
Column Type: Capillary
Column

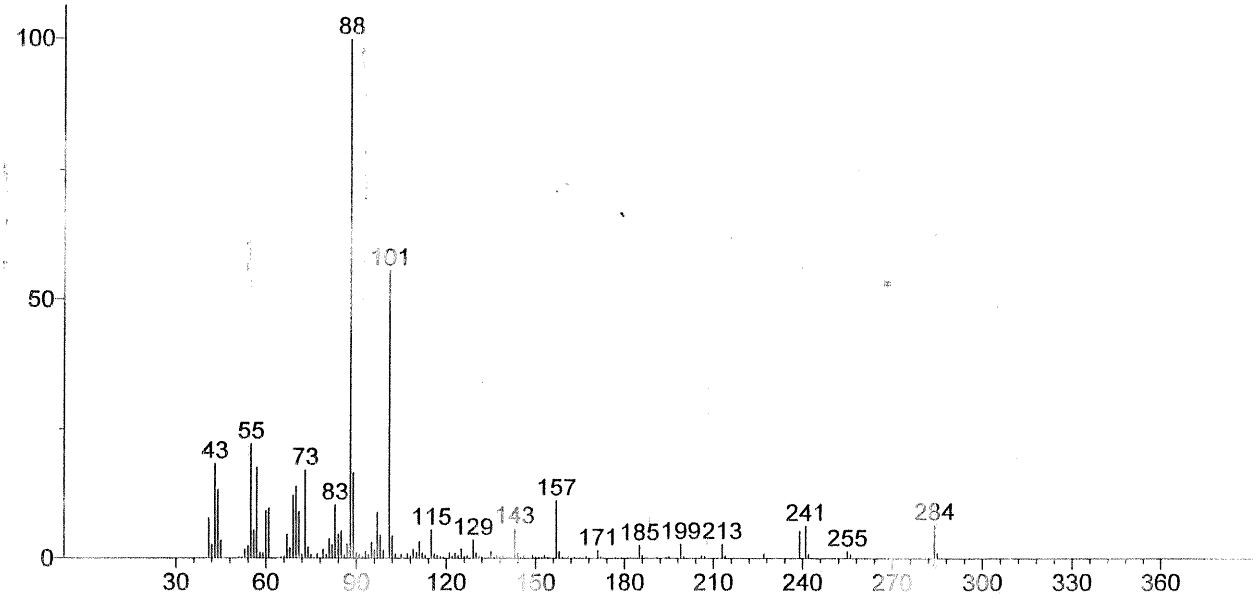

(Text File) +EI Scan (49.527 min) ALI-PIS-H1-210421.D

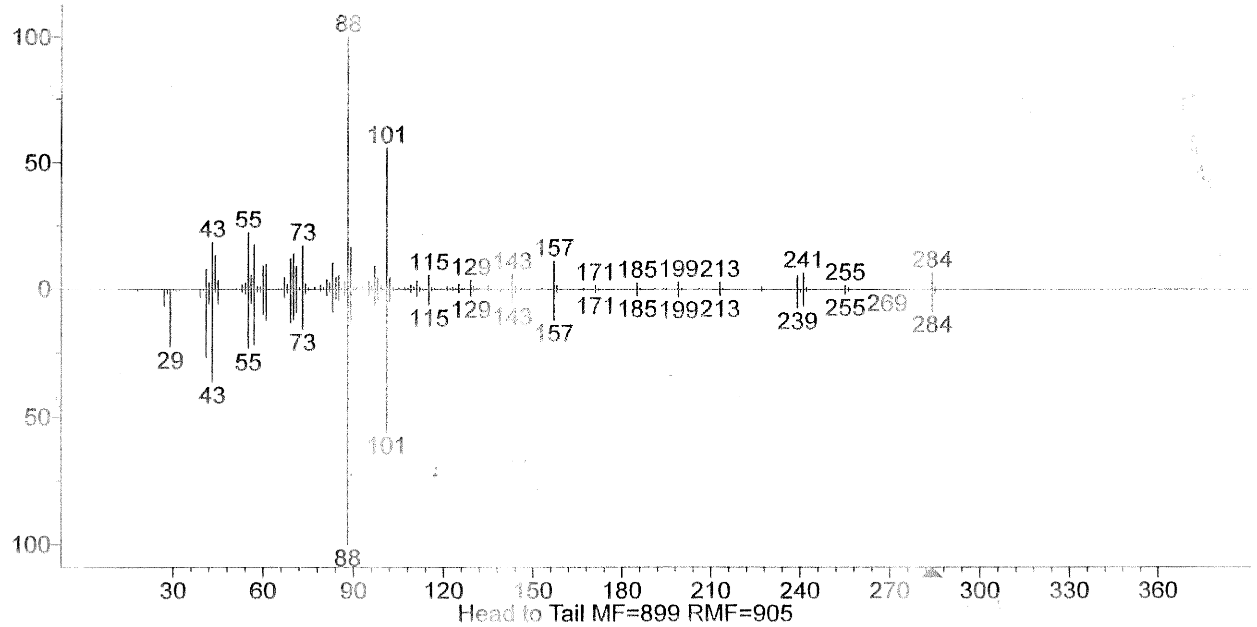

Head to Tail MF=899 RMF=905

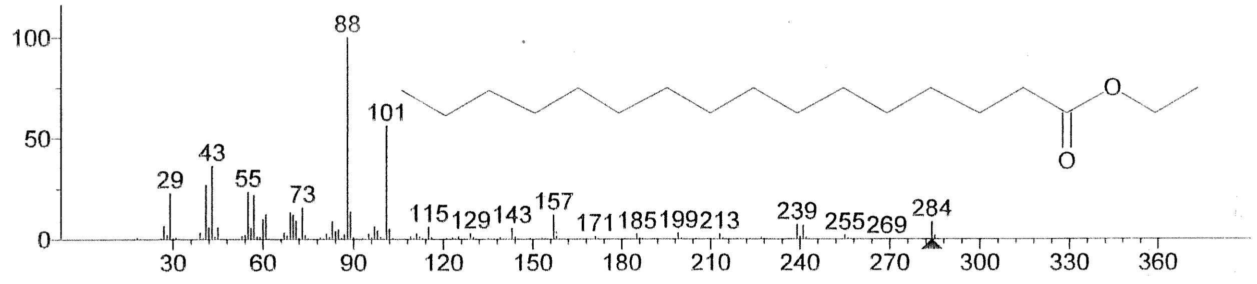

(mainlib) Hexadecanoic acid, ethyl ester

Name: Hexadecanoic acid, ethyl ester
Formula: $C_{18}H_{36}O_2$
MW: 284 CAS#: 628-97-7 NIST#: 233204 ID#: 49485 DB: mainlib
Other DBs: Fine, TSCA, EPA, HODOC, NIH, EINECS, IRDB
Contributor: Japan AIST/NIMC Database- Spectrum MS-NW-5396
10 largest peaks:
88 999 | 101 559 | 43 362 | 41 268 | 55 233 | 29 227 | 57 217 | 73 156 | 89 136 | 69 132 |
Synonyms:
1.Palmitic acid, ethyl ester
2.Ethyl hexadecanoate
3.Ethyl palmitate

Estimated non-polar retention index (n-alkane scale):
Value: 1978 iu
Confidence interval (Esters): 47(50%) 201(95%) iu

Retention index.
1. Value: 1968 iu
Column Type: Capillary
Column Class: Standard non-polar
Active Phase: SPB-1
Column
Length: 30 m
Carrier Gas: He
Column Diameter: 0.25 mm
Phase Thickness: 0.25 um
Data Type: Linear
RI
Program Type: Ramp
Start T: 50 C
End T: 250 C
Heat Rate: 5 K/min
Start Time: 3 min
End Time: 15
min
Source: Blagojevic, P.; Radulovic, N.; Palic, R.; Stojanovic, G., Chemical composition of the essential oils of Serbian wild-growing Srtemisia absinthium and Artemisia vulgaris, J. Agric. Food Chem., 54, 2006, 4780-4789.

2.
Value: 1966 iu
Column Type: Capillary
Column Class: Standard non-polar
Active Phase: HP-1
Column Length:
50 m
Carrier Gas: He
Column Diameter: 0.2 mm
Phase Thickness: 0.33 um
Data Type: Linear RI
Program
Type: Ramp
Start T: 60 C
End T: 250 C
Heat Rate: 2 K/min

31

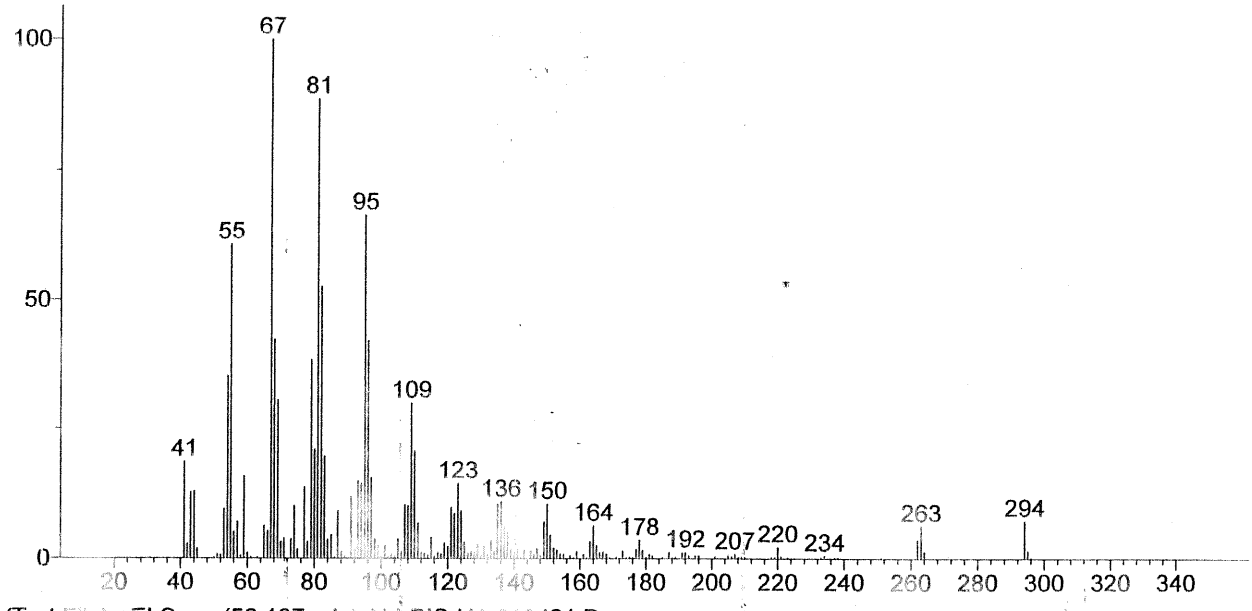

(Text File) +EI Scan (56.187 min) ALI-PIS-H1-210421.D

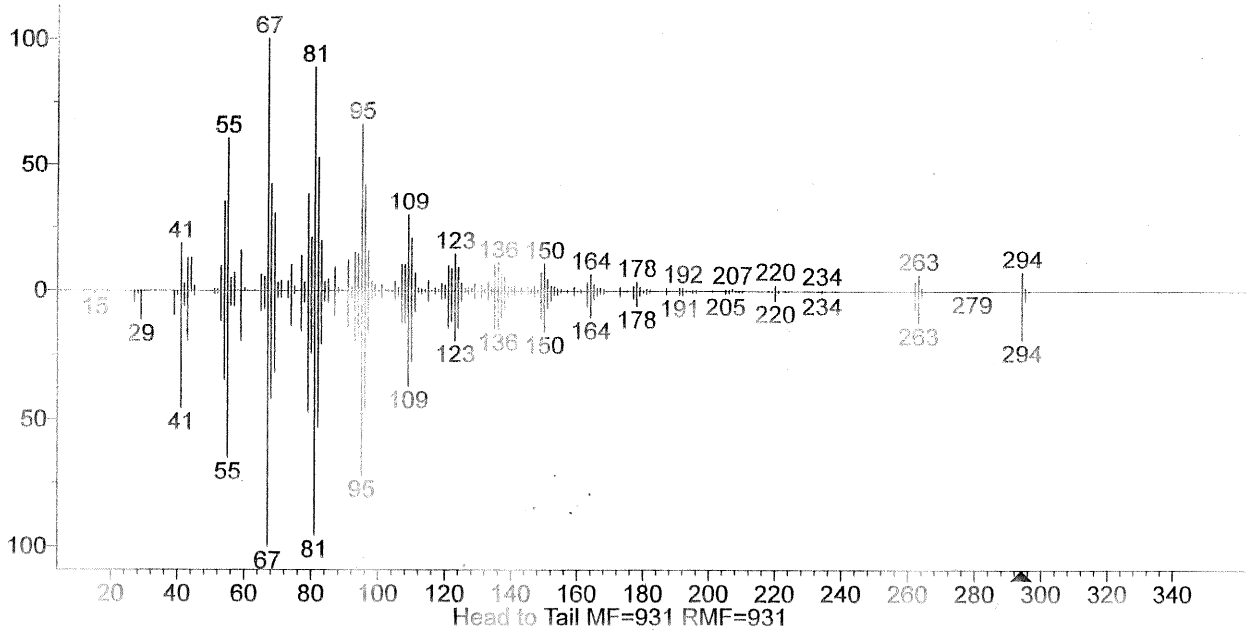

Head to Tail MF=931 RMF=931

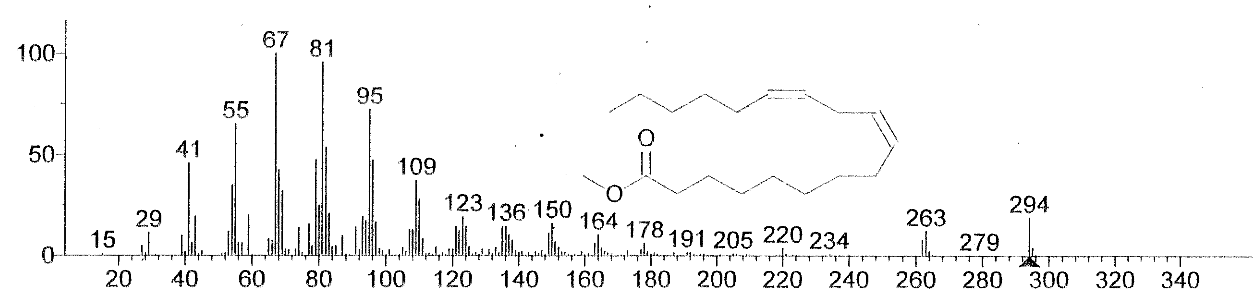

(mainlib) 9,12-Octadecadienoic acid (Z,Z)-, methyl ester

Name: 9,12-Octadecadienoic acid (Z,Z)-, methyl ester
Formula: $C_{19}H_{34}O_2$
MW: 294 CAS#: 112-63-0 NIST#: 333205 ID#: 28886 DB: mainlib
Other DBs: Fine, TSCA, EPA, HODOC, NIH, EINECS
Contributor: NIST Mass Spectrometry Data Center
10 largest peaks:
67 999 | 81 956 | 95 720 | 55 650 | 82 534 | 79 474 | 96 472 | 41 456 | 68 423 | 109 372 |
Synonyms:
1.Linoleic acid, methyl ester
2.Methyl cis,cis-9,12-octadecadienoate
3.Methyl linoleate
4.Methyl octadecadienoate
5.Methyl 9-cis,12-cis-octadecadienoate
6.Methyl (9Z,12Z)-9,12-octadecadienoate #

Estimated non-polar retention index (n-alkane scale):
Value: 2093 iu
Confidence interval (Esters): 47(50%) 201(95%) iu

Retention index.
1. Value: 2071 iu
Column Type: Capillary
Column Class: Standard non-polar
Active Phase: SPB-1
Column
Length: 30 m
Carrier Gas: He
Column Diameter: 0.25 mm
Phase Thickness: 0.25 um
Data Type: Linear
RI
Program Type: Ramp
Start T: 50 C
End T: 250 C
Heat Rate: 5 K/min
Start Time: 3 min
End Time: 15
min
Source: Blagojevic, P.; Radulovic, N.; Palic, R.; Stojanovic, G., Chemical composition of the essential oils of Serbian wild-growing Srtemisia absinthium and Artemisia vulgaris, J. Agric. Food Chem., 54, 2006, 4780-4789.

2.
Value: 2087 iu
Column Type: Capillary
Column Class: Standard non-polar
Active Phase: BP-1
Column Length:
30 m
Carrier Gas: N2
Column Diameter: 0.32 mm
Phase Thickness: 0.25 um
Data Type: Linear RI
Program
Type: Ramp

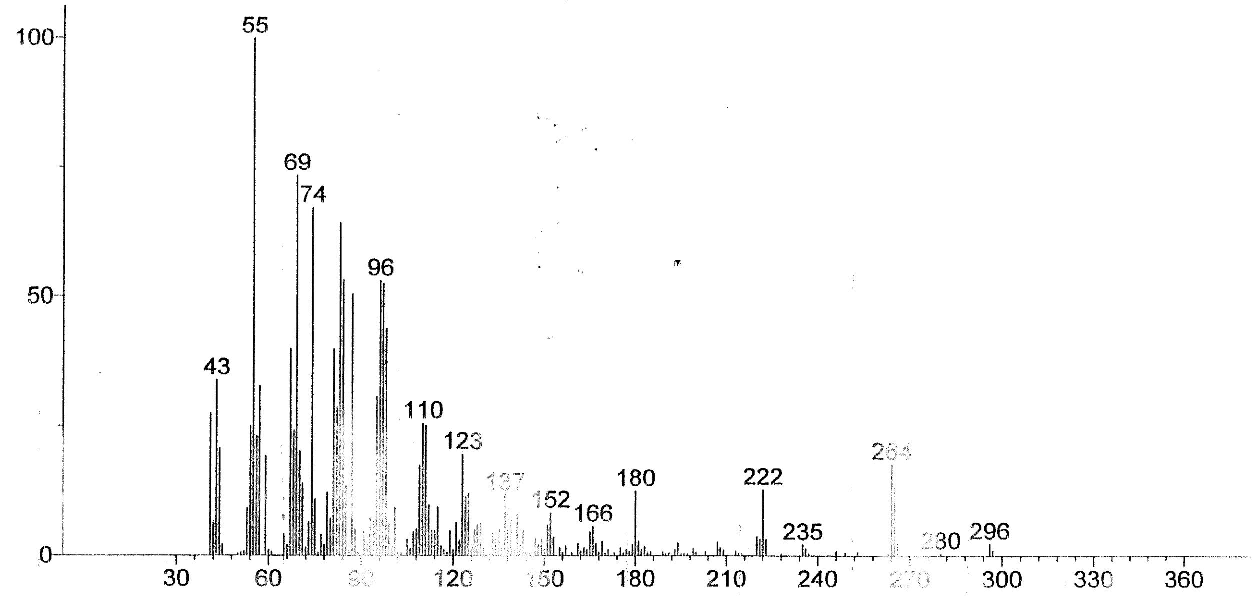

(Text File) +EI Scan (56.654 min) ALI-PIS-H1-210421.D

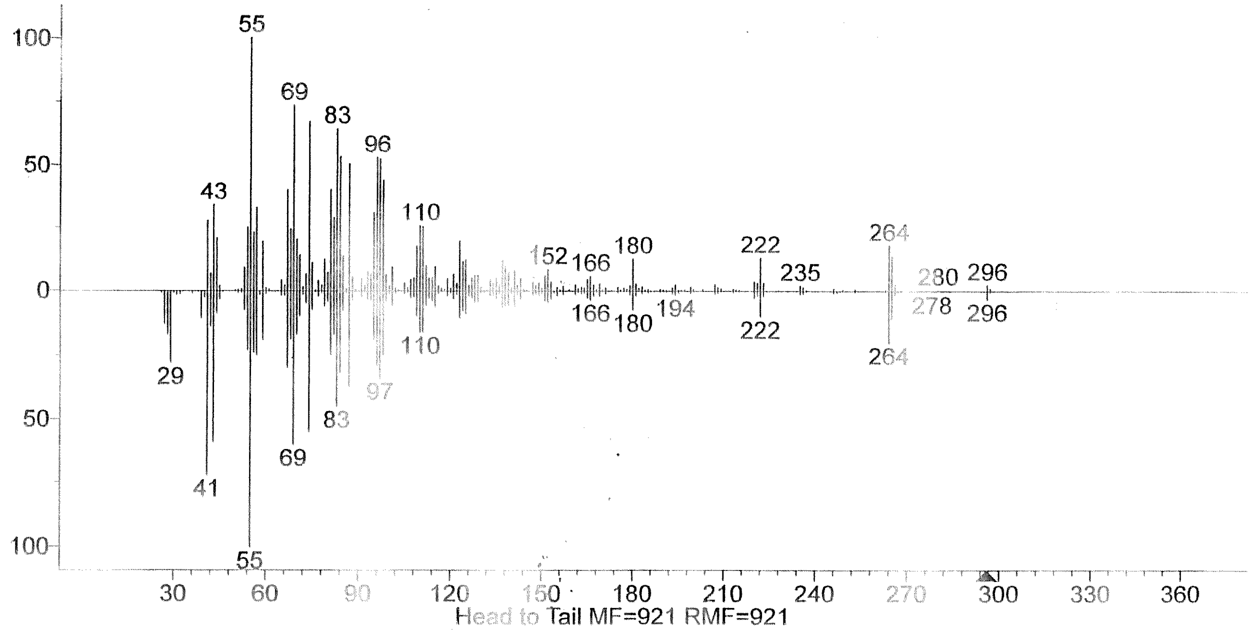

Head to Tail MF=921 RMF=921

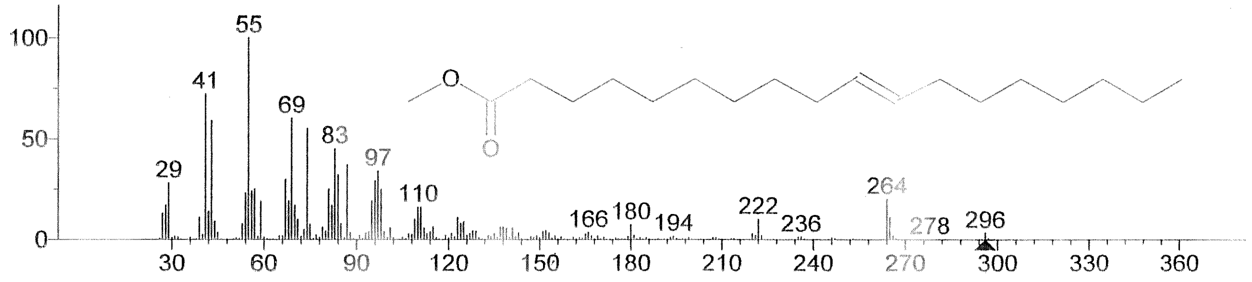

(mainlib) 10-Octadecenoic acid, methyl ester

Name: 10-Octadecenoic acid, methyl ester
Formula: $C_{19}H_{36}O_2$
MW: 296 CAS#: 13481-95-3 NIST#: 36207 ID#: 17521 DB: mainlib
Other DBs: None
Contributor: R.T.HOLMAN,UNIVERSITY OF MINNESOTA
10 largest peaks:
55 999 | 41 720 | 69 600 | 43 590 | 74 550 | 83 450 | 87 370 | 97 340 | 84 320 | 67 300 |
Synonyms:
1.Methyl (10E)-10-octadecenoate #

Estimated non-polar retention index (n-alkane scale):
Value: 2085 iu
Confidence interval (Esters): 47(50%) 201(95%) iu

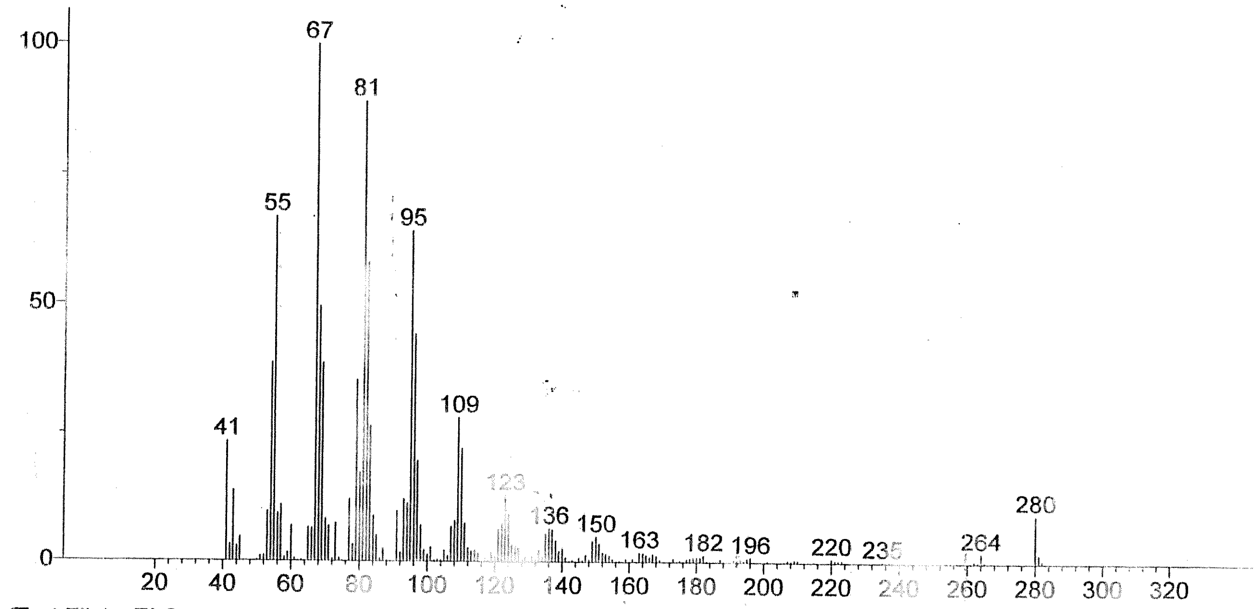

(Text File) +EI Scan (60.172 min) ALI-PIS-H1-210421.D

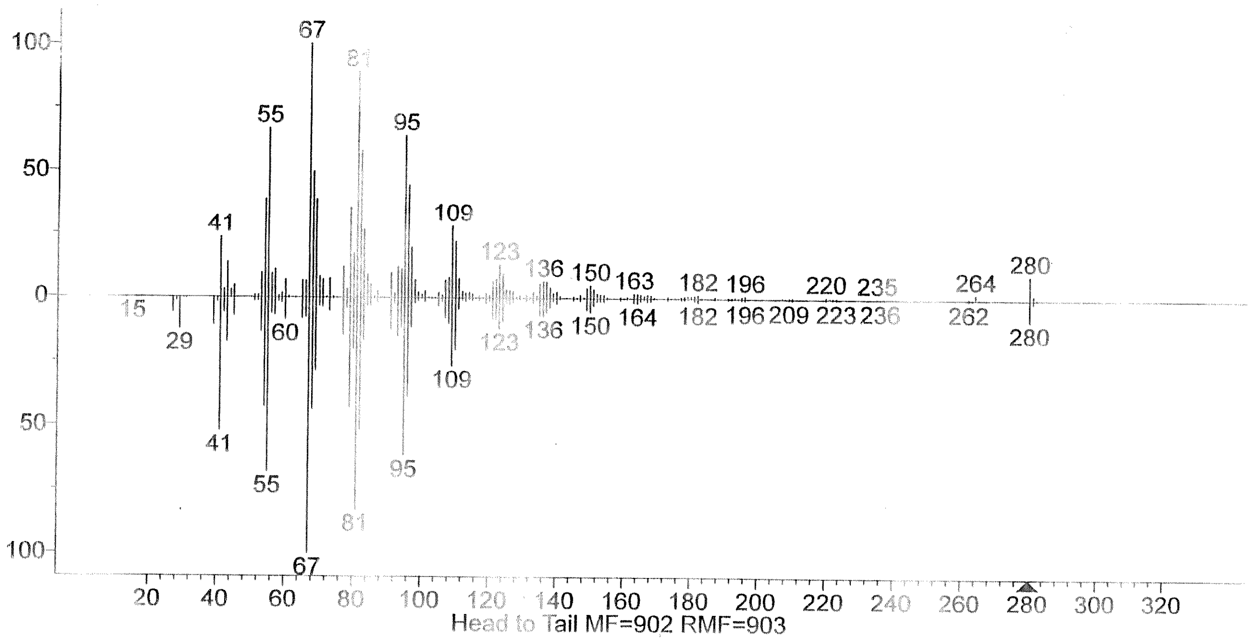

Head to Tail MF=902 RMF=903

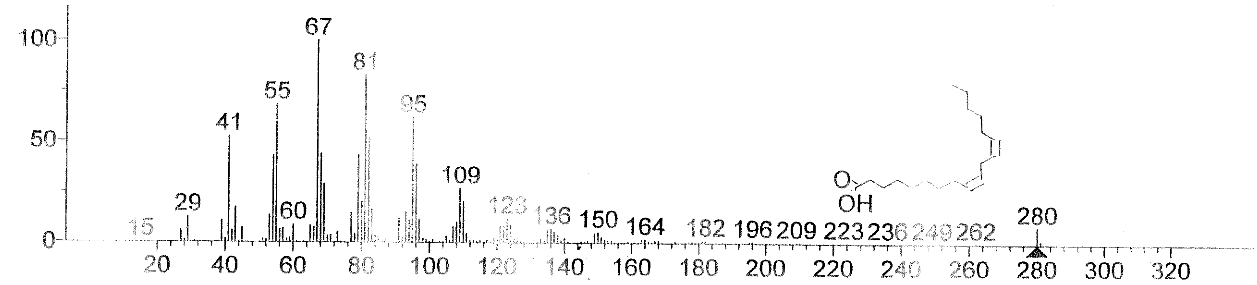

(replib) 9,12-Octadecadienoic acid (Z,Z)-

Name: 9,12-Octadecadienoic acid (Z,Z)-
Formula: $C_{18}H_{32}O_2$
MW: 280 CAS#: 60-33-3 NIST#: 333207 ID#: 7212 DB: replib
Other DBs: Fine, TSCA, RTECS, HODOC, NIH, EINECS
Contributor: NIST Mass Spectrometry Data Center
10 largest peaks:
67 999 | 81 827 | 55 680 | 95 617 | 41 523 | 82 516 | 68 437 | 79 430 | 54 429 | 96 388 |
Synonyms:
1.cis-9,cis-12-Octadecadienoic acid
2.cis,cis-Linoleic acid
3.Grape seed oil
4.Linoleic
5.Linoleic acid
6.Linolic acid
7.Polylin No. 515
8.Telfairic acid
9.Unifac 6550
10.9,12-Octadecadienoic acid
11.Leinoleic acid
12.9,12-Linoleic acid
13.cis,cis-9,12-octadecadienoic acid
14.Linoelaidic acid
15.Linoleic acid 95
16.Emersol 310
17.Emersol 315
18.Vespula pensylvanica b708568k063
19.Pamolyn
20.Pamolyn 125
21.Pamolyn 200, 240
22.Pamolyn 380

Estimated non-polar retention index (n-alkane scale):
Value: 2183 iu
Confidence interval (Carboxylic acids): 51(50%) 220(95%) iu

Retention index.
1. Value: 2095 iu
Column Type: Capillary
Column Class: Standard non-polar
Active Phase: CP Sil 5 CB
Column
Length: 25 m
Carrier Gas: H2
Column Diameter: 0.25 mm
Data Type: Linear RI
Program Type: Ramp
Start T:
80 C
End T: 270 C
Heat Rate: 10 K/min
Source: Ziegenbein, F.C.; Hanssen, H.-P.; König, W.A., Secondary metabolites from Ganoderma lucidum and Spongiporus leucomallellus, Phytochemistry, 67, 2006, 202-211.

2.

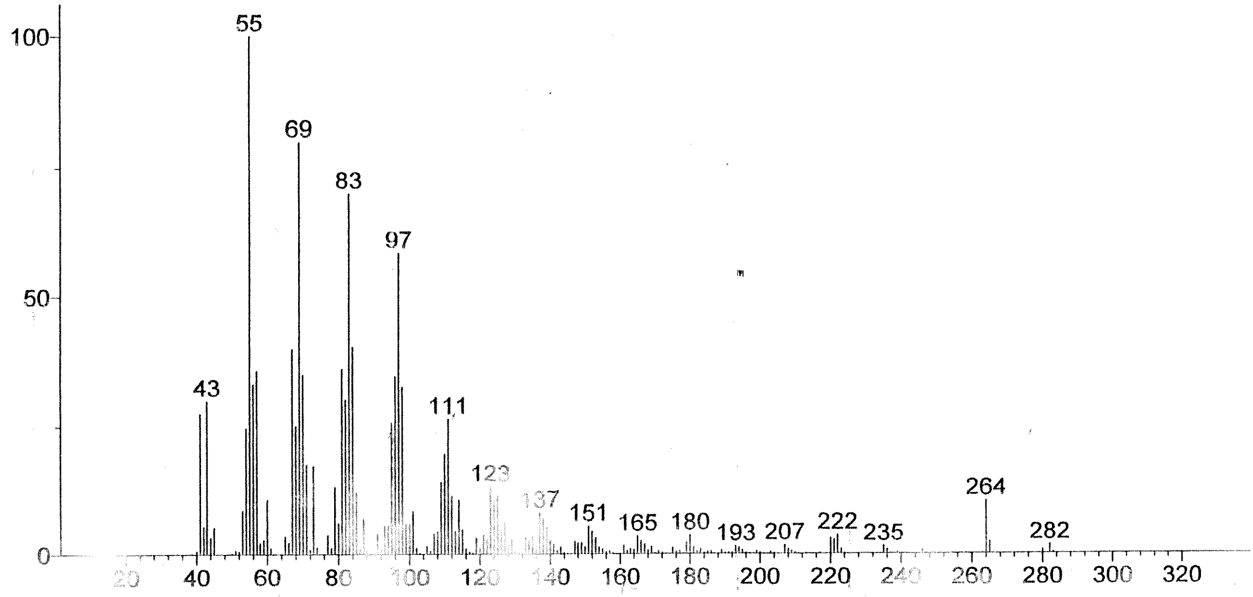

(Text File) +EI Scan (60.678 min) ALI-PIS-H1-210421.D

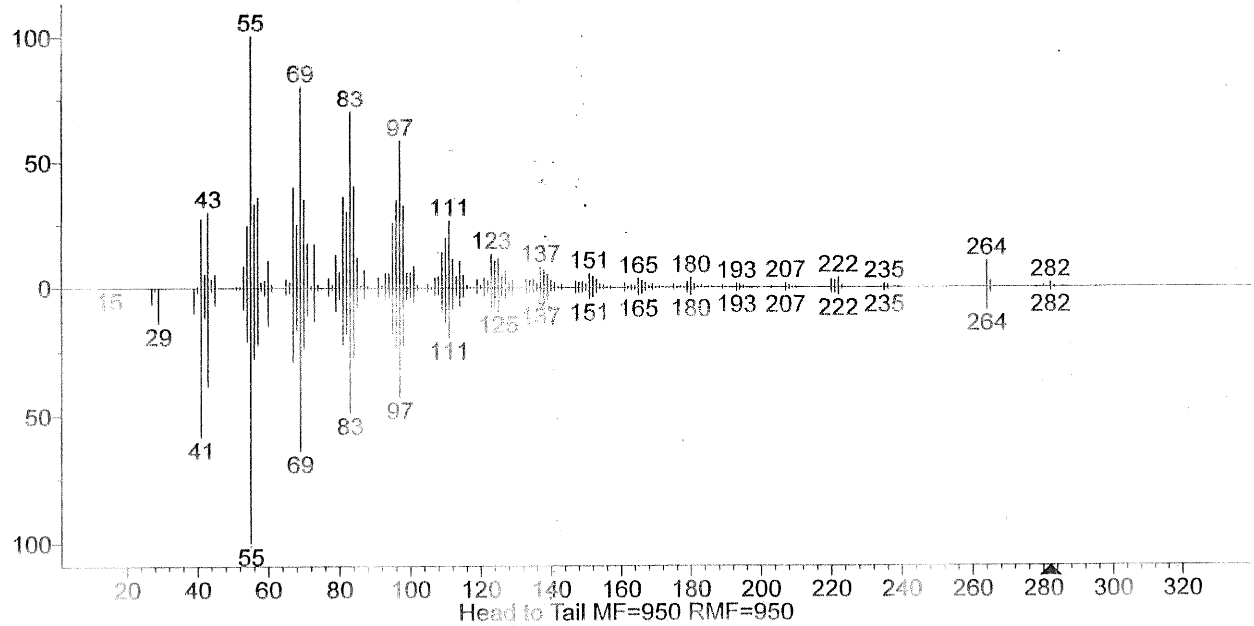

Head to Tail MF=950 RMF=950

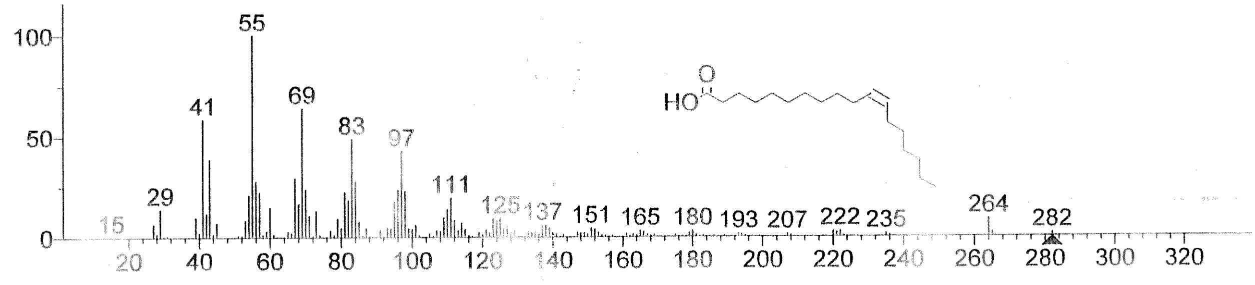

(mainlib) cis-Vaccenic acid

Name: cis-Vaccenic acid
Formula: $C_{18}H_{34}O_2$
MW: 282 CAS#: 506-17-2 NIST#: 333599 ID#: 18204 DB: mainlib
Other DBs: None
Contributor: NIST Mass Spectrometry Data Center
10 largest peaks:
55 999 | 69 638 | 41 583 | 83 487 | 97 426 | 43 386 | 67 290 | 56 276 | 84 273 | 70 237 |
Synonyms:
no synonyms.

Estimated non-polar retention index (n-alkane scale):
Value: 2175 iu
Confidence interval (Carboxylic acids): 51(50%) 220(95%) iu

Retention index.
1. Value: 2116.6 iu
Column Type: Capillary
Column Class: Semi-standard non-polar
Active Phase: VF
-5MS
Column Length: 30 m
Carrier Gas: He
Column Diameter: 0.25 mm
Phase Thickness: 0.25 um
Data Type:
Linear RI
Program Type: Ramp
Start T: 60 C
End T: 270 C
Source: Tret'yakov, K.V., Retention Data. NIST
Mass Spectrometry Data Center., 2007.

2. Value: 2161.8 iu
Column Type: Capillary
Column Class: Semi
-standard non-polar
Active Phase: VF-5MS
Column Length: 30 m
Carrier Gas: He
Column Diameter: 0.25
mm
Phase Thickness: 0.25 um
Data Type: Linear RI
Program Type: Ramp
Start T: 60 C
End T: 270
C
Source: Tret'yakov, K.V., Retention Data. NIST Mass Spectrometry Data Center., 2007.

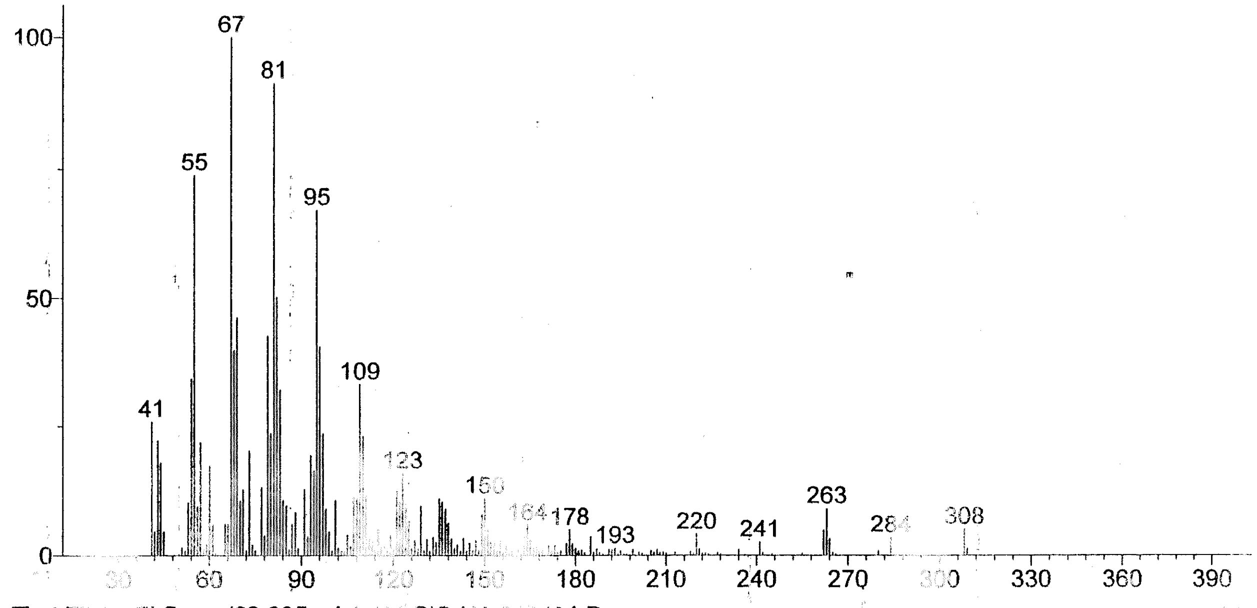

(Text File) +EI Scan (62.025 min) ALI-PIS-H1-210421.D

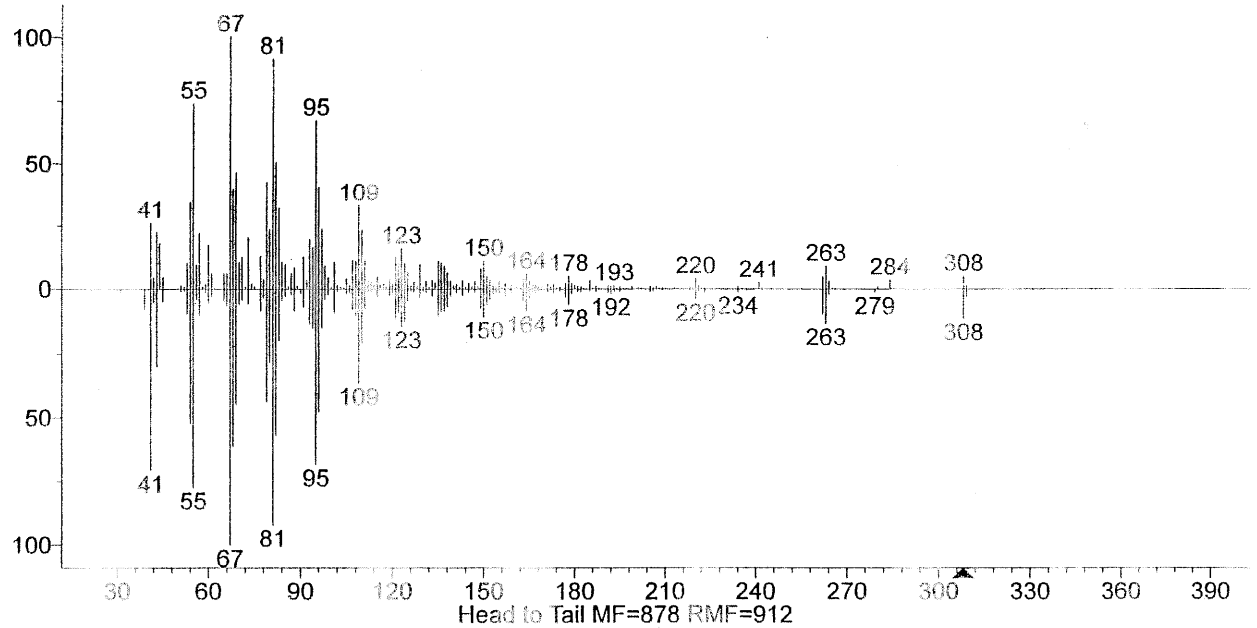

Head to Tail MF=878 RMF=912

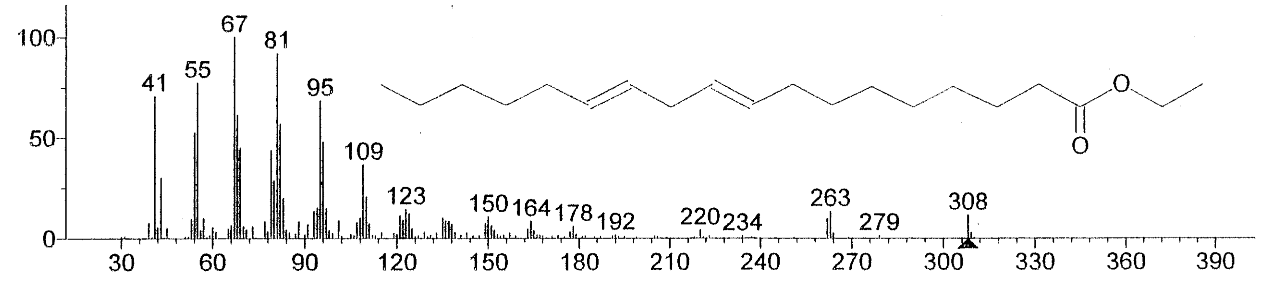

(mainlib) 9,12-Octadecadienoic acid, ethyl ester

Name: 9,12-Octadecadienoic acid, ethyl ester
Formula: $C_{20}H_{36}O_2$
MW: 308 CAS#: 7619-08-1 NIST#: 249157 ID#: 28827 DB: mainlib
Other DBs: HODOC
Contributor: TNO Volatile Compounds in Food - Chemical Concepts
10 largest peaks:
67 999 | 81 919 | 55 772 | 41 707 | 95 683 | 68 613 | 82 569 | 54 525 | 96 478 | 69 448 |
Synonyms:
1.Ethyl (9E,12E)-9,12-octadecadienoate #

Estimated non-polar retention index (n-alkane scale):
Value: 2193 iu
Confidence interval (Esters): 47(50%) 201(95%) iu

Retention index.
1. Value: 2171 iu
Column Type: Capillary
Column Class: Semi-standard non-polar
Active Phase: HP
-5MS
Column Length: 30 m
Carrier Gas: He
Column Diameter: 0.25 mm
Phase Thickness: 0.25 um
Data Type:
Normal alkane RI
Program Type: Complex
Description: 40C (10min) => 3C/min =>120C =>10C/min =>250C
(5min)
Source: Ansorena, D.; Gimeno, O.; Astiasarán, I.; Bello, J., Analysis of volatile compounds by GC-MS of a dry fermented sausage: chorizo de Pamplona, Food Res. Int., 34, 2001, 67-75.

2. Value: 2527 iu
Column Type:
Capillary
Column Class: Standard polar
Active Phase: CP-Wax 52CB
Column Length: 60 m
Carrier Gas:
He
Column Diameter: 0.25 mm
Phase Thickness: 0.25 um
Data Type: Linear RI
Program Type:
Complex
Description: 45C(5min) =>10C/min =>80C =>2C/min =>240C
Source: Romeo, V.; Ziino, M.; Giuffrida,
D.; Condurso, C.; Verzera, A., Flavour profile of capers (Capparis spinosa L.) from the Eolian Archipelago by HS
-SPME/GC?MS, Food Chem., 101, 2007, 1272-1278.

<...>

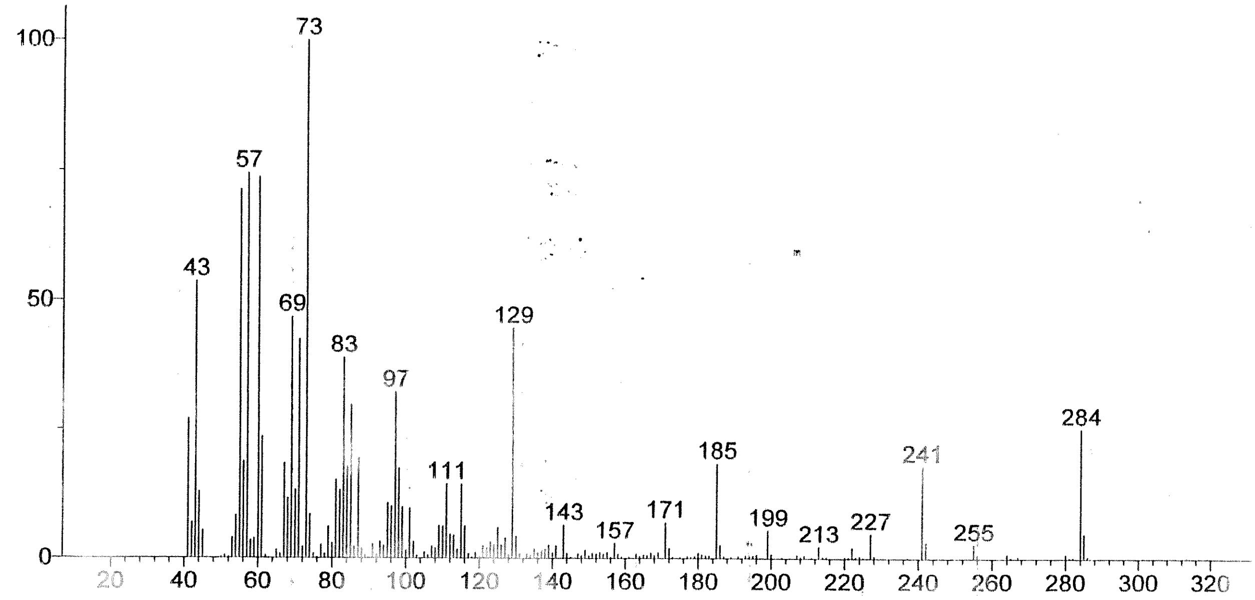

(Text File) +EI Scan (62.292 min) ALI-PIS-H1-210421.D

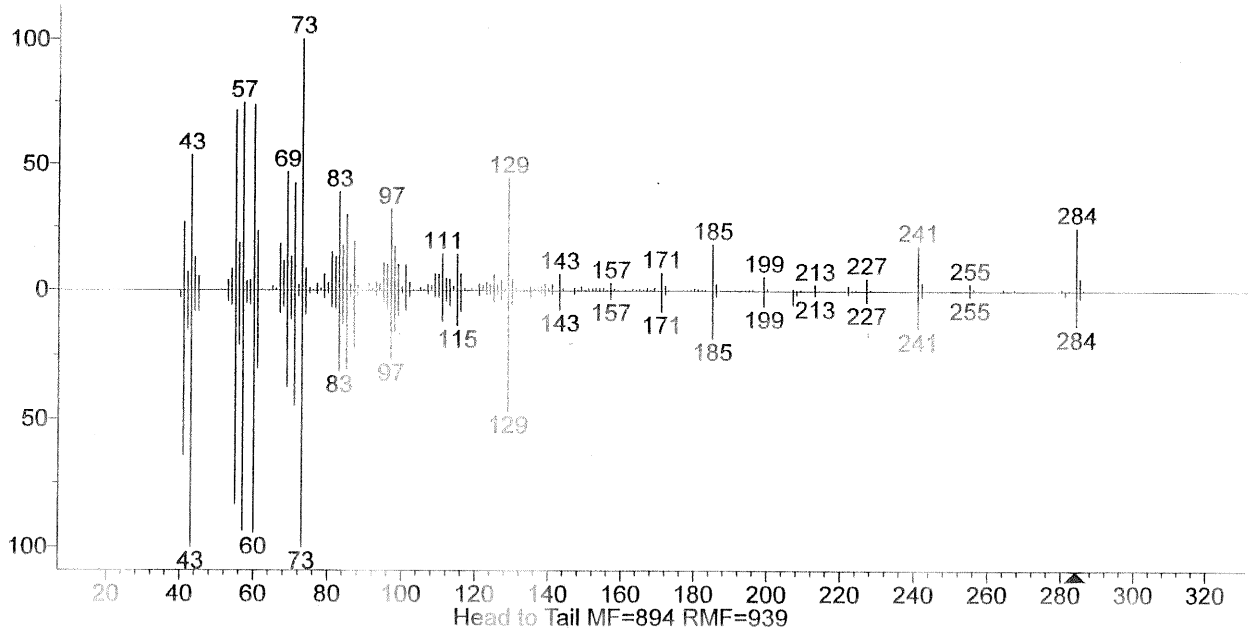

Head to Tail MF=894 RMF=939

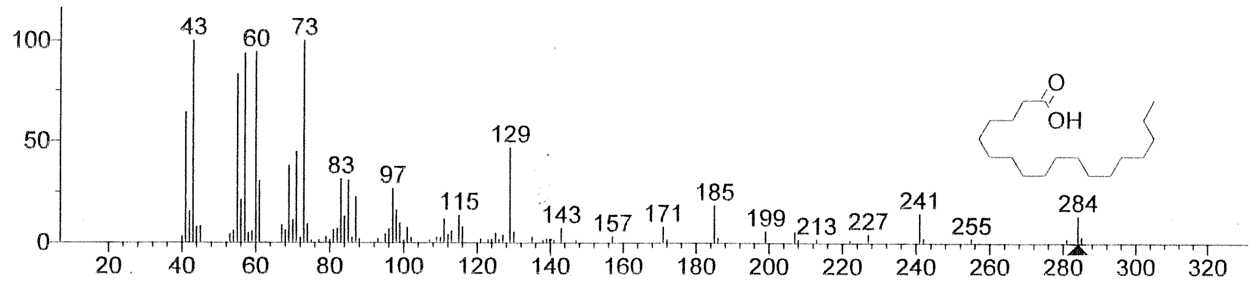

(replib) Octadecanoic acid

Name: Octadecanoic acid
Formula: $C_{18}H_{36}O_2$
MW: 284 CAS#: 57-11-4 NIST#: 334866 ID#: 2560 DB: replib
Other DBs: Fine, TSCA, RTECS, EPA, HODOC, NIH, EINECS, IRDB
Contributor: Drug Lab
10 largest peaks:
43 999 | 73 999 | 60 942 | 57 934 | 55 832 | 41 642 | 129 468 | 71 447 | 69 376 | 83 311 |
Synonyms:
1.Stearic acid
2.n-Octadecanoic acid
3.Humko Industrene R
4.Hydrofol Acid 150
5.Hystrene S-97
6.Hystrene T-70
7.Hystrene 80
8.Industrene R
9.Kam 1000
10.Kam 2000
11.Kam 3000
12.Neo-Fat 18
13.Neo-Fat 18-53
14.Neo-Fat 18-54
15.Neo-Fat 18-55
16.Neo-Fat 18-59
17.NAA 173
18.PD 185
19.Stearex Beads
20.Stearophanic acid
21.Steric acid
22.Vanicol
23.1-Heptadecanecarboxylic acid
24.Heptadecanecarboxylic acid
25.Neo-fat 18-61
26.Pearl stearic
27.Century 1240
28.Dar-chem 14
29.Emersol 120
30.Emersol 132
31.Emersol 150
32.Formula 300
33.Glycon dp
34.Glycon tp
35.Glycon S-70
36.Glycon S-80
37.Glycon S-90
38.Groco 54
39.Groco 55
40.Groco 55l
41.Groco 58
42.Groco 59
43.Hy-phi 1199
44.Hy-phi 1205
45.Hy-phi 1303
46.Hy-phi 1401

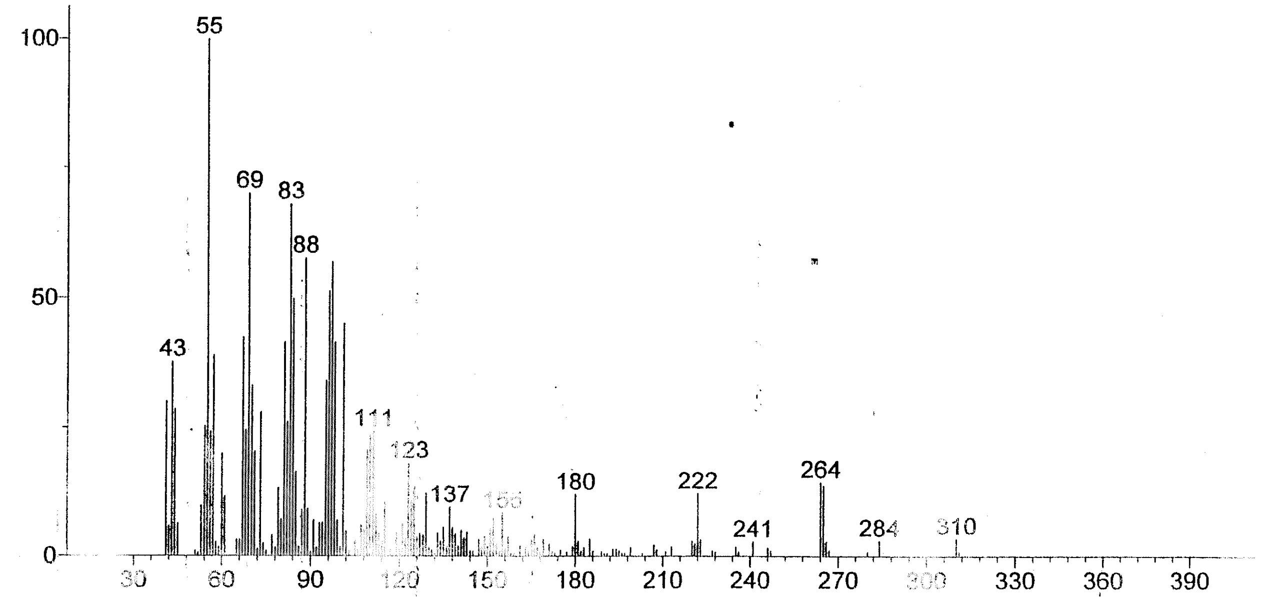

(Text File) +EI Scan (62.609 min) ALI-PIS-H1-210421.D

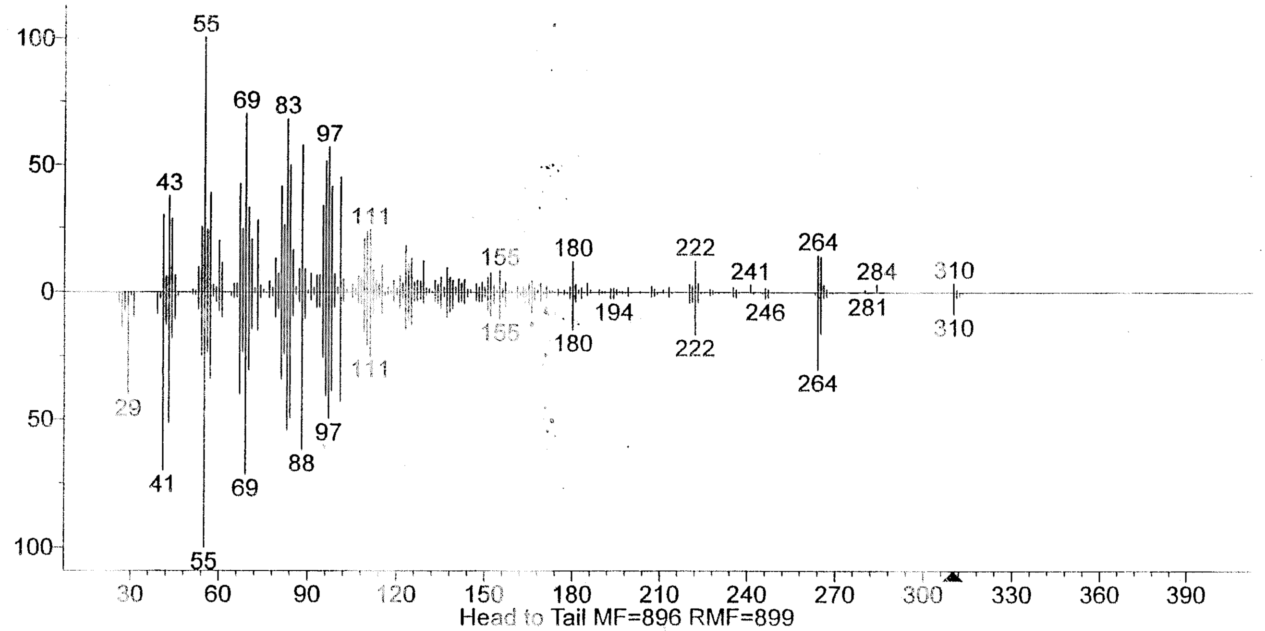

Head to Tail MF=896 RMF=899

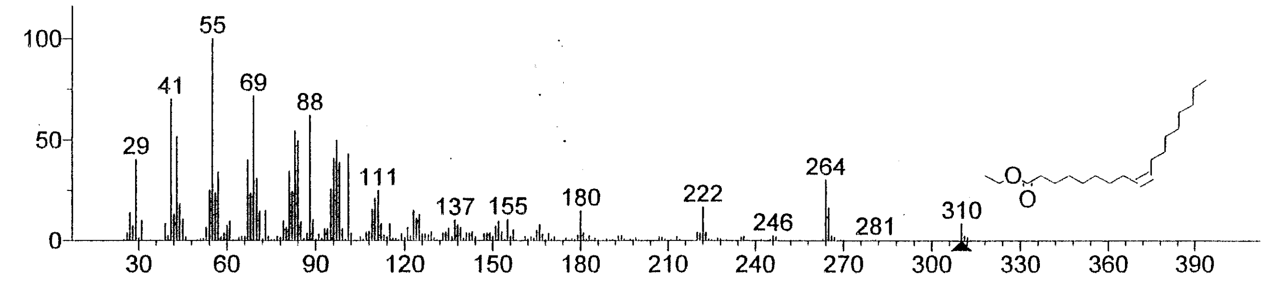

(replib) Ethyl Oleate

Name: Ethyl Oleate
Formula: $C_{20}H_{38}O_2$
MW: 310 CAS#: 111-62-6 NIST#: 150151 ID#: 4466 DB: replib
Other DBs: Fine, TSCA, RTECS, USP, HODOC, NIH, EINECS, IRDB
Contributor: Chemical Concepts
10 largest peaks:
55 999 | 69 714 | 41 699 | 88 618 | 83 543 | 43 515 | 97 496 | 84 495 | 101 429 | 96 407 |
Synonyms:
1.9-Octadecenoic acid (Z)-, ethyl ester
2.Oleic acid, ethyl ester
3.(Z)-9-Octadecenoic acid ethyl ester
4.Ethyl cis-9-octadecenoate
5.Ethyl Z-9-octadecenoate
6.Ethyl (9Z)-9-octadecenoate #

Estimated non-polar retention index (n-alkane scale):
Value: 2185 iu
Confidence interval (Esters): 47(50%) 201(95%) iu

Retention index.
1. Value: 2171 iu
Column Type: Capillary
Column Class: Standard non-polar
Active Phase: CP Sil 5 CB
Column
Length: 60 m
Carrier Gas: He
Column Diameter: 0.32 mm
Phase Thickness: 0.25 um
Data Type: Linear
RI
Program Type: Ramp
Start T: 60 C
End T: 280 C
Heat Rate: 3 K/min
Start Time: 10 min
End Time: 60
min
Source: Pino, J.; Almora, K.; Marbot, R., Volatile components of papaya (Carica papaya L., maradol variety) fruit, Flavour Fragr. J., 18, 2003, 492-496.

2. Value: 2160 iu
Column Type: Capillary
Column Class: Standard
non-polar
Active Phase: OV-1
Column Length: 25 m
Carrier Gas: He
Column Diameter: 0.32 mm
Data Type:
Linear RI
Program Type: Ramp
Start T: 40 C
End T: 280 C

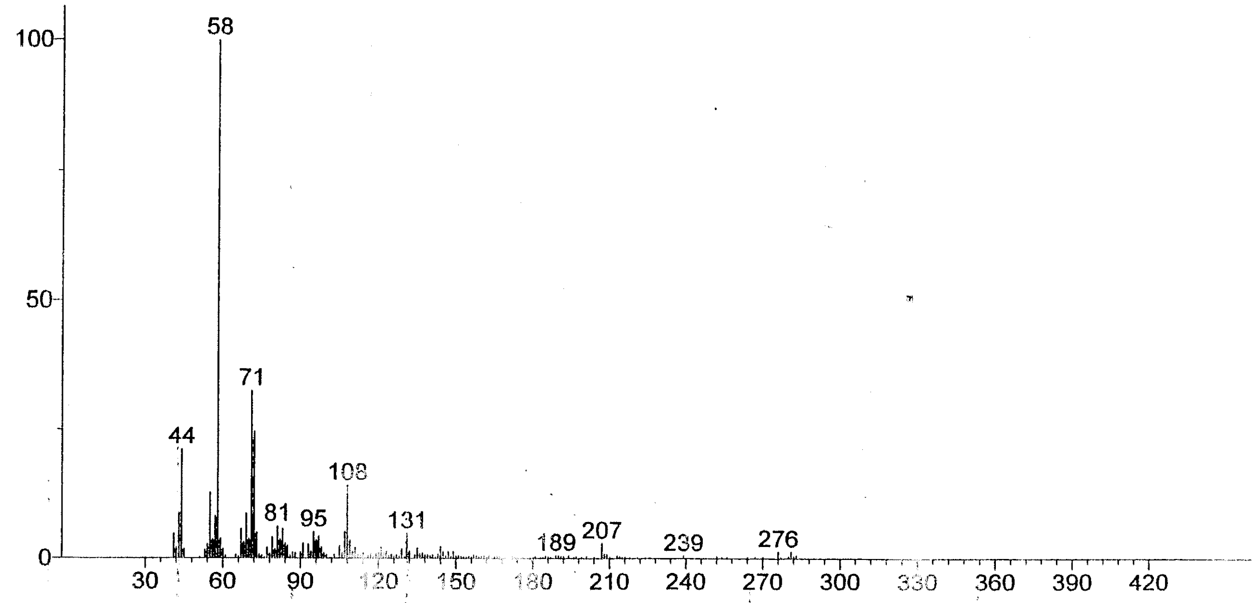

(Text File) +EI Scan (70.392 min) ALI-PIS-H1-210421.D

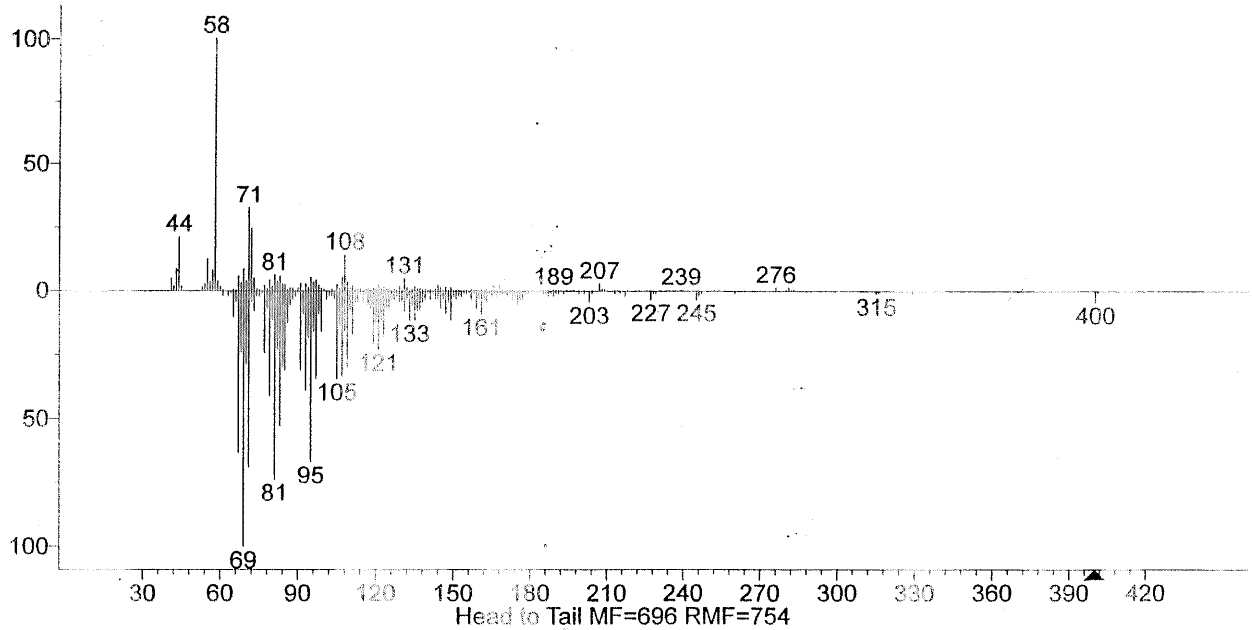

Head to Tail MF=696 RMF=754

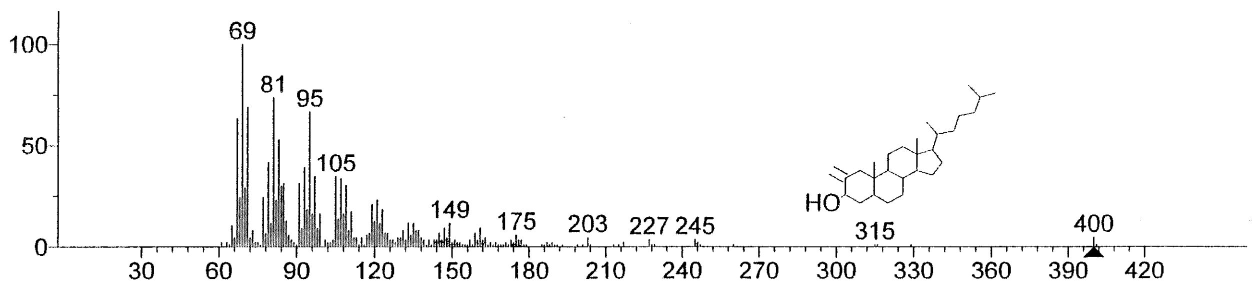

(mainlib) Cholestan-3-ol, 2-methylene-, (3β,5α)-

Name: Cholestan-3-ol, 2-methylene-, (3β,5α)-
Formula: $C_{28}H_{48}O$
MW: 400 CAS#: 22599-96-8 NIST#: 48741 ID#: 30965 DB: mainlib
Other DBs: None
Contributor: CARL DJERASSI DEPT OF CHEM STANFORD UNIV STANFORD CALIF 94305
10 largest peaks:
69 999 | 81 735 | 71 689 | 95 666 | 67 632 | 83 528 | 79 413 | 93 390 | 97 344 | 105 344 |
Synonyms:
1.5α-Cholestan-3β-ol, 2-methylene-
2.2-Methylenecholestan-3-ol #

Estimated non-polar retention index (n-alkane scale):
Value: 2652 iu
Confidence interval (Low reliability): 174(50%) 752(95%) iu

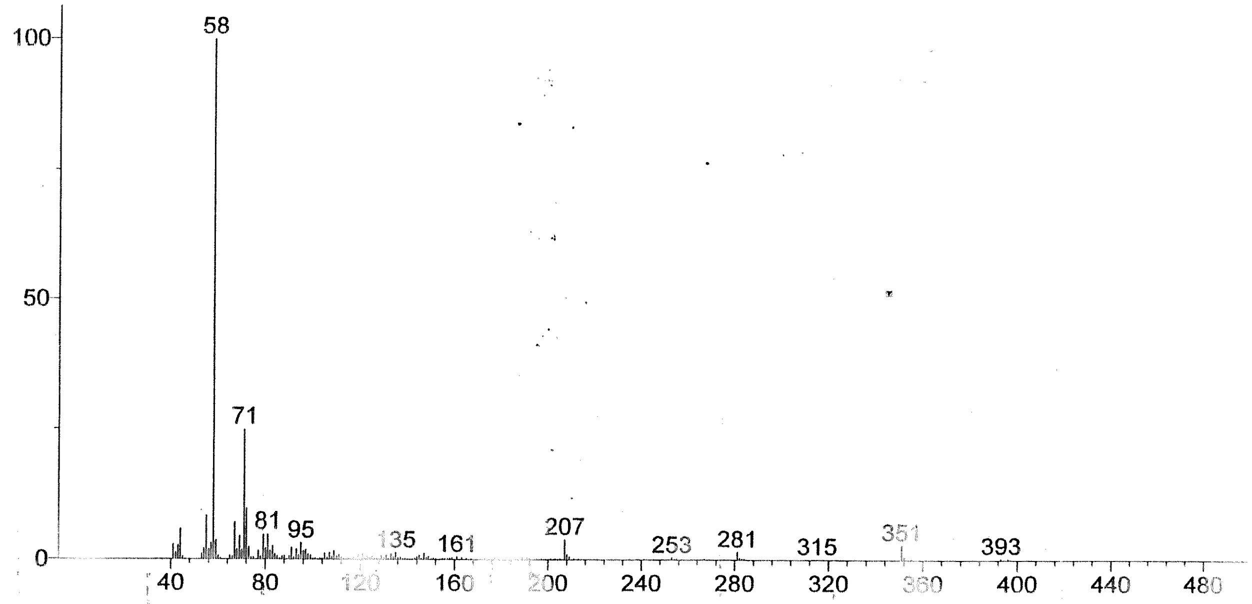

(Text File) +EI Scan (74.929 min) ALI-PIS-H1-210421.D

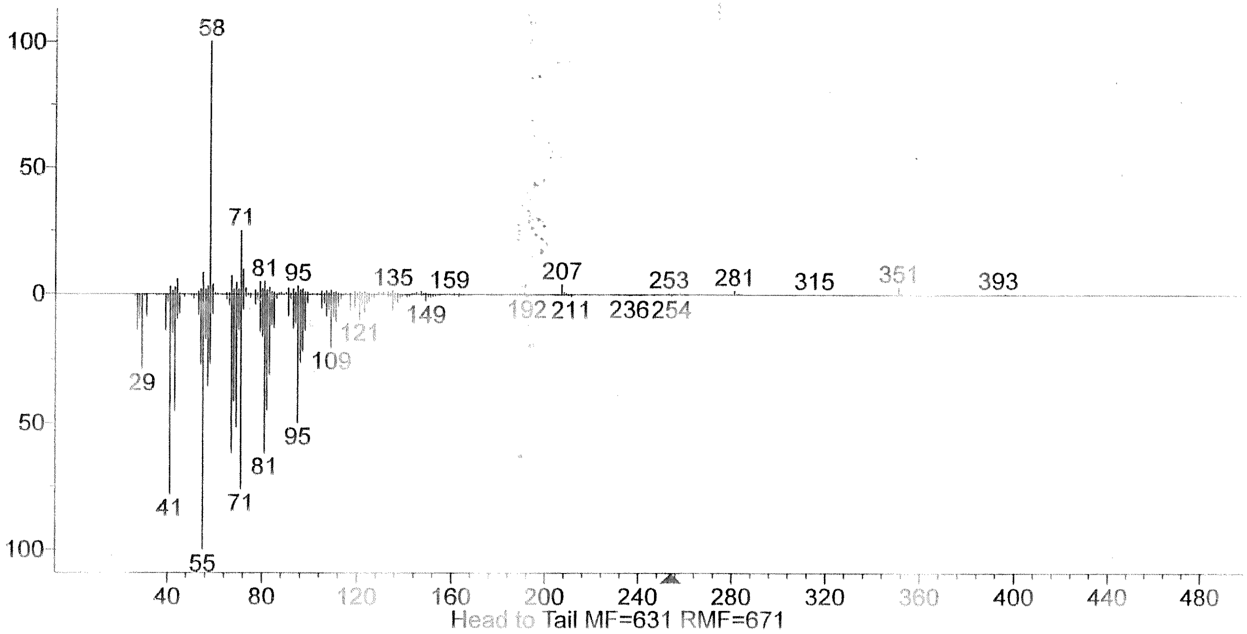

Head to Tail MF=631 RMF=671

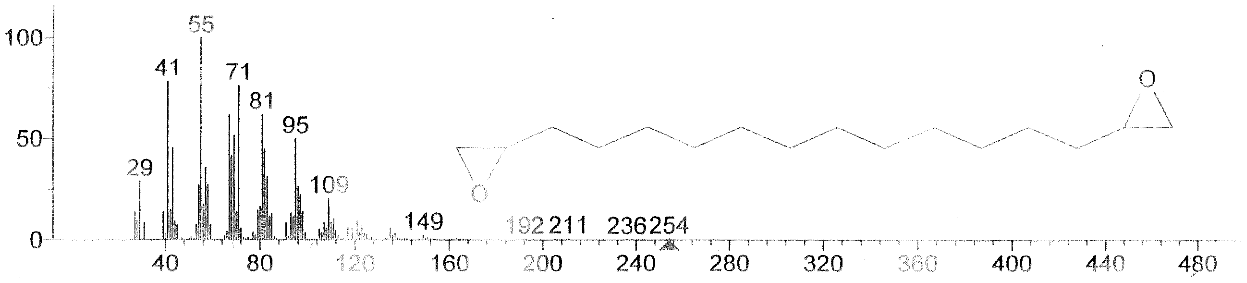

(mainlib) 1,2-15,16-Diepoxyhexadecane

Name: 1,2-15,16-Diepoxyhexadecane
Formula: $C_{16}H_{30}O_2$
MW: 254 NIST#: 192650 ID#: 17537 DB: mainlib
Contributor: Chemical Concepts
10 largest peaks:
55 999 | 41 782 | 71 762 | 81 618 | 67 616 | 69 516 | 95 499 | 43 453 | 82 449 | 68 417 |
Synonyms:
no synonyms.

Estimated non-polar retention index (n-alkane scale):
Value: 1792 iu
Confidence interval (Ethers): 68(50%) 293(95%) iu

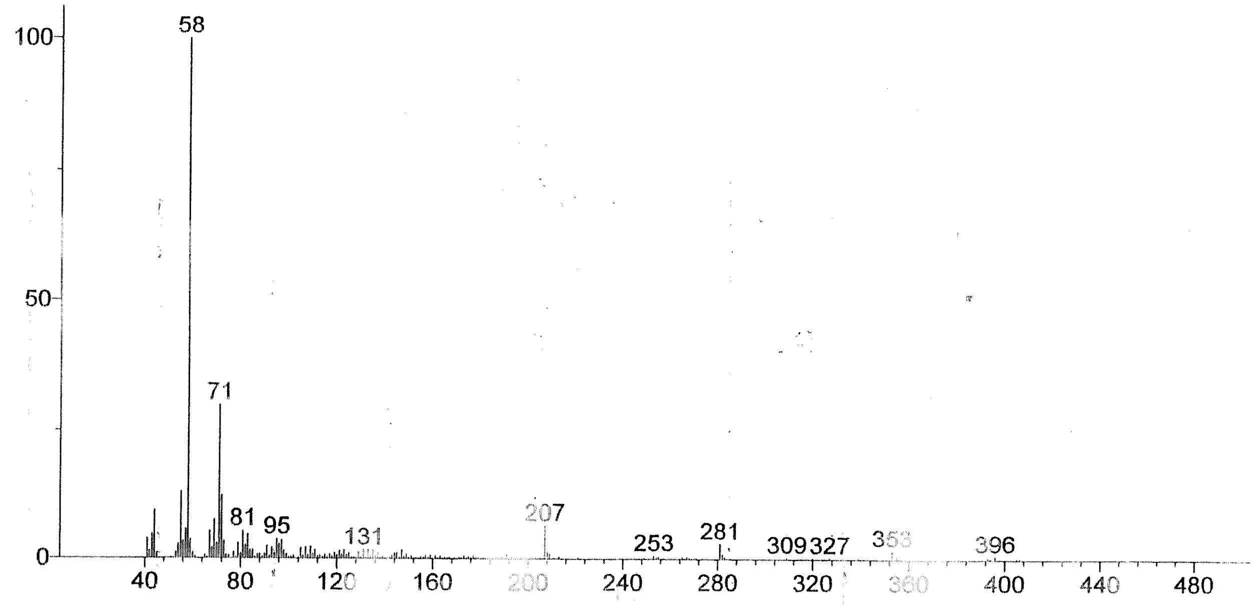

(Text File) +EI Scan (75.025 min) ALI-PIS-H1-210421.D

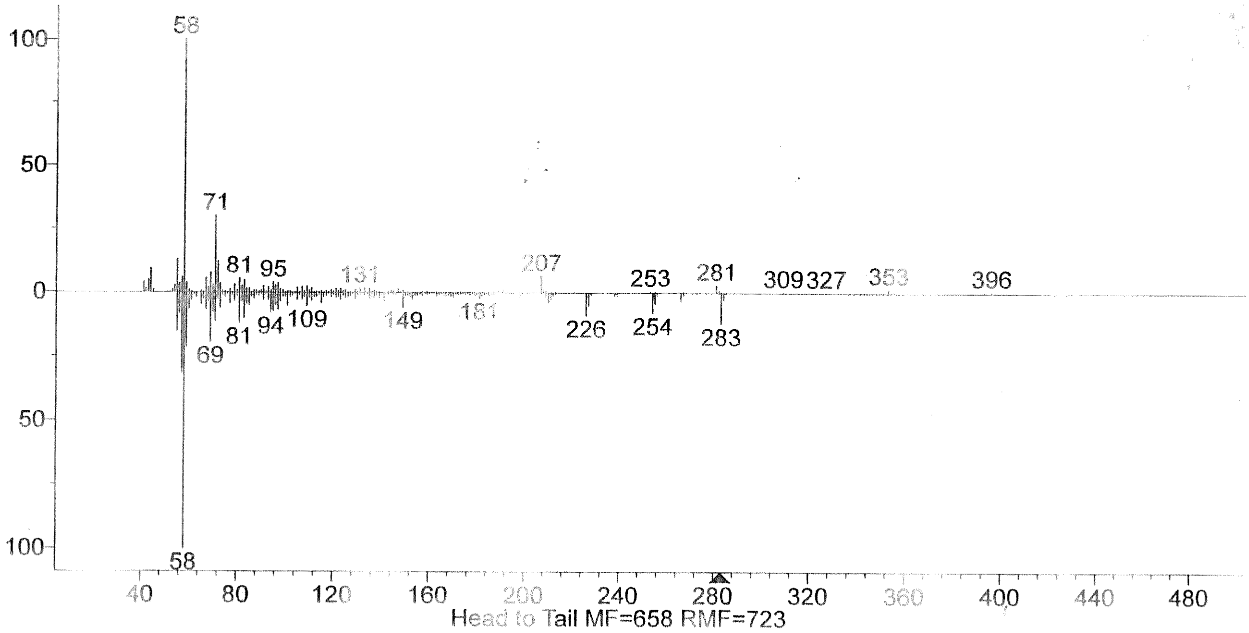

Head to Tail MF=658 RMF=723

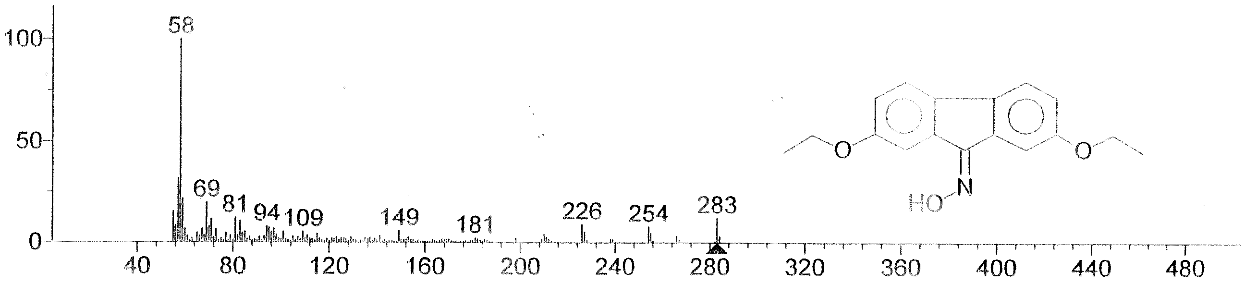

(mainlib) 9-Oximino-2,7-diethoxyfluorene

50

Name: 9-Oximino-2,7-diethoxyfluorene
Formula: $C_{17}H_{17}NO_3$
MW: 283 CAS#: 327041-58-7 NIST#: 111061 ID#: 24922 DB: mainlib
Other DBs: None
Contributor: Institute of Organic Chemistry, USSR, 1990
10 largest peaks:
58 999 | 57 316 | 59 216 | 69 195 | 55 153 | 81 121 | 283 121 | 71 116 | 83 105 | 226 90 |
Synonyms:
1.2,7-Diethoxy-9H-fluoren-9-one oxime #

Estimated non-polar retention index (n-alkane scale):
Value: 2403 iu
Confidence interval (Diverse functional groups): 89(50%) 382(95%) iu

S1

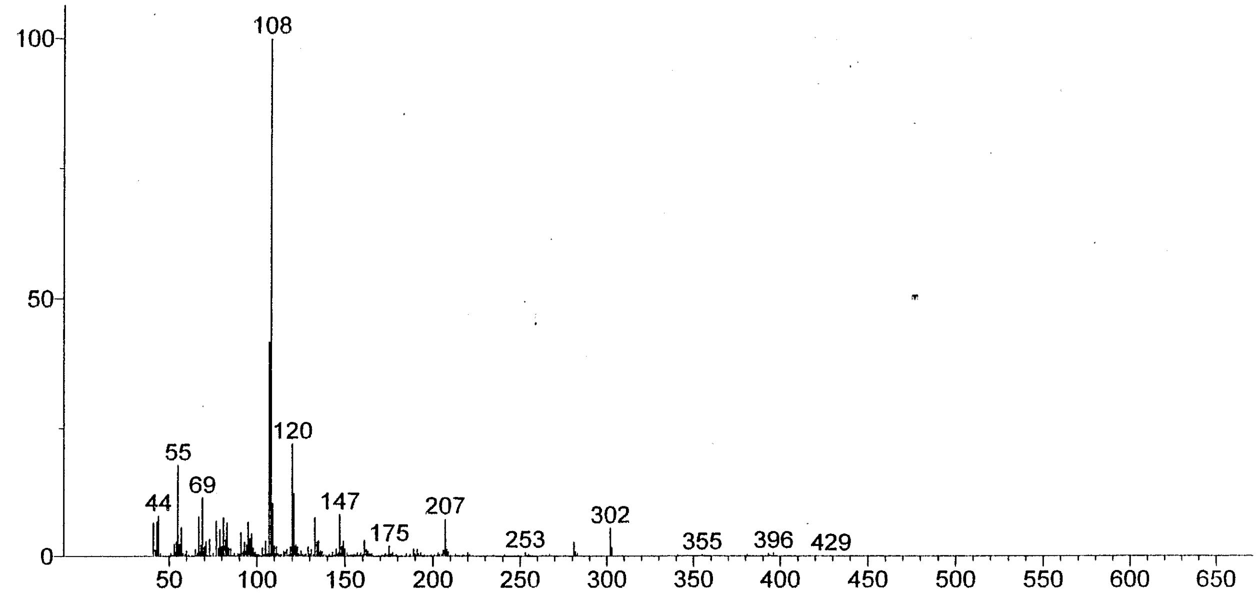

(Text File) +EI Scan (75.328 min) ALI-PIS-H1-210421.D

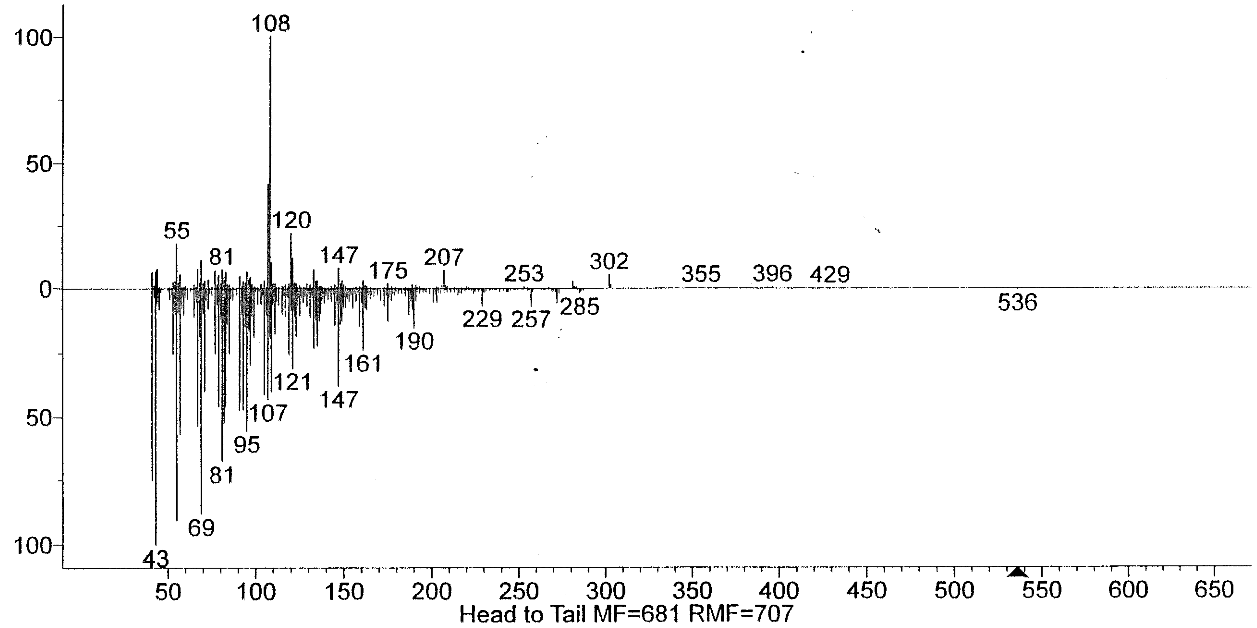

Head to Tail MF=681 RMF=707

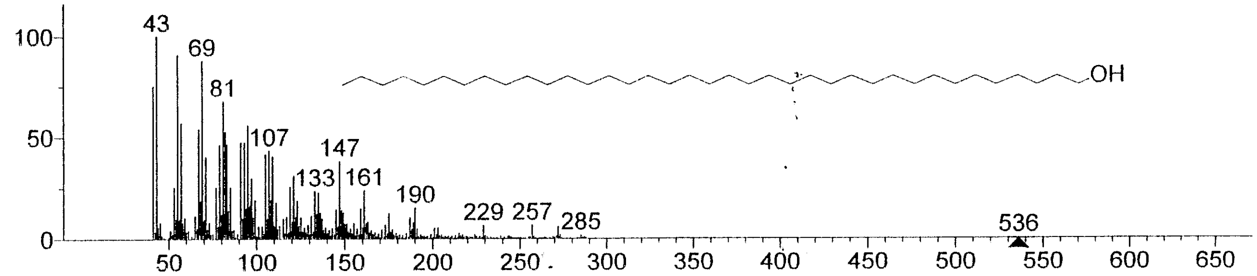

(mainlib) 1-Heptatriacotanol

52

Name: 1-Heptatriacotanol
Formula: $C_{37}H_{76}O$
MW: 536 CAS#: 105794-58-9 NIST#: 127968 ID#: 6806 DB: mainlib
Other DBs: None
Contributor: LAC, NIDDK, NIH, Bethesda, MD 20892
10 largest peaks:
43 999 | 55 907 | 69 878 | 41 750 | 81 675 | 57 567 | 95 556 | 67 537 | 82 525 | 91 474 |
Synonyms:
1.1-Heptatriacontanol #

Estimated non-polar retention index (n-alkane scale):
Value: 3942 iu
Confidence interval (Alcohols): 41(50%) 176(95%) iu

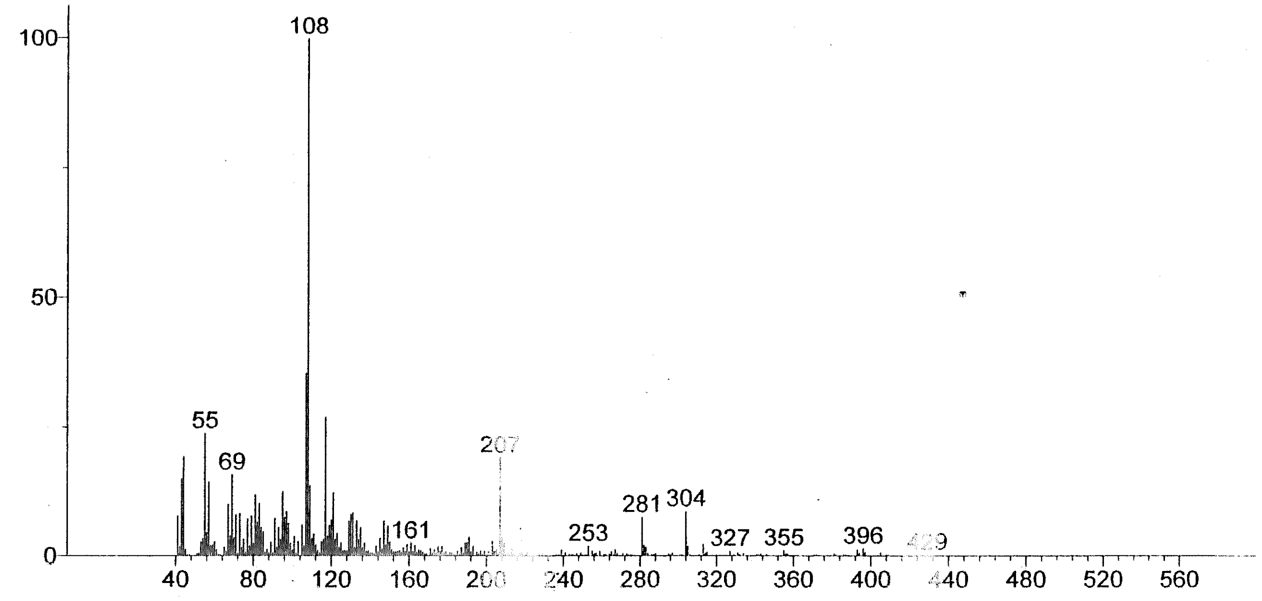

(Text File) +EI Scan (75.702 min) ALI-PIS-H1-210421.D

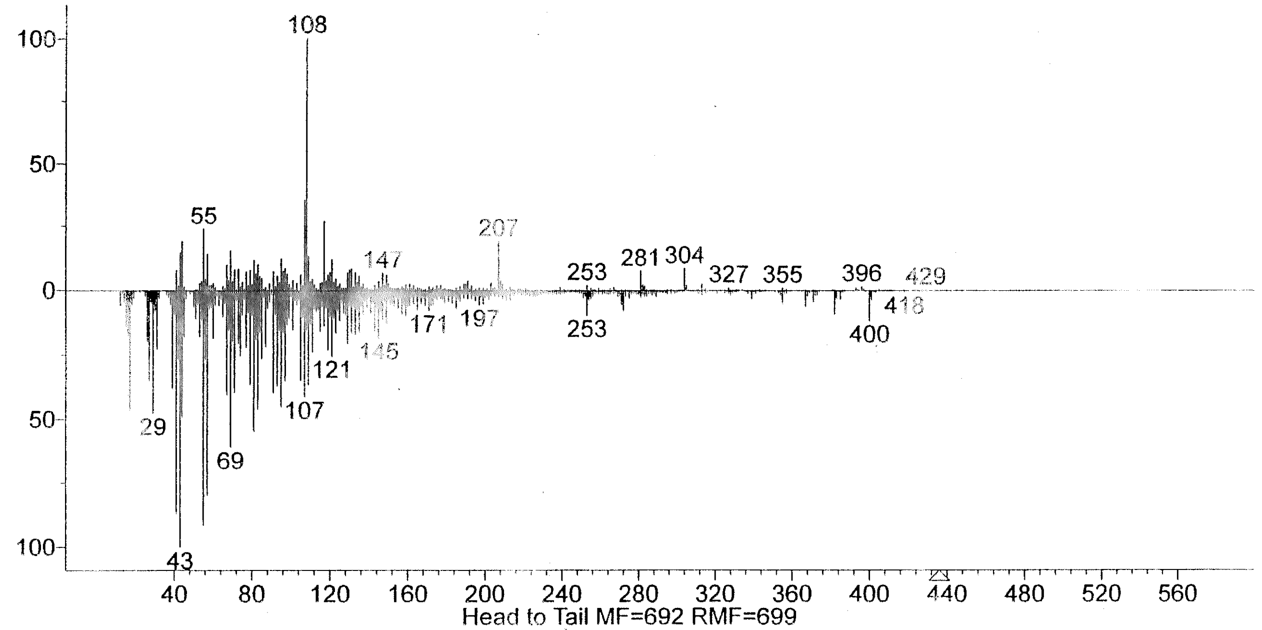

Head to Tail MF=692 RMF=699

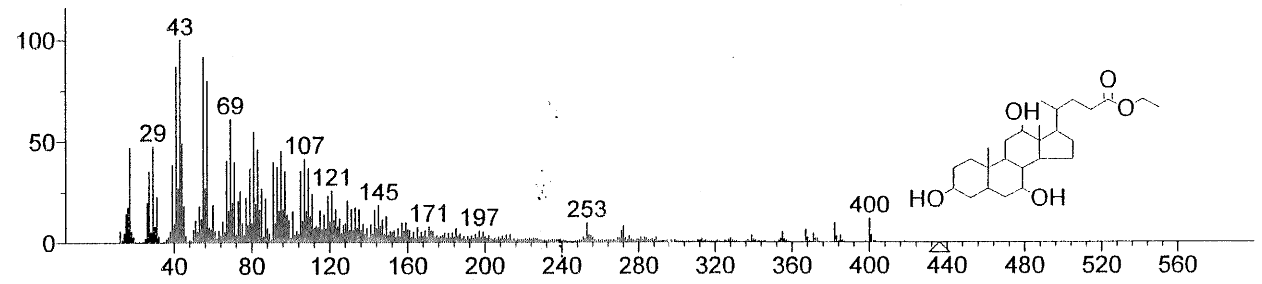

(mainlib) Ethyl iso-allocholate

Name: Ethyl iso-allocholate
Formula: $C_{26}H_{44}O_5$
MW: 436 NIST#: 43053 ID#: 6556 DB: mainlib
Contributor: R RYHAGE MS-LAB KAROLINSKA INSTITUTET STOCKHOLM SWEDEN
10 largest peaks:
43 999 | 55 914 | 41 867 | 57 797 | 69 609 | 81 547 | 44 492 | 29 476 | 17 469 | 83 460 |
Synonyms:
no synonyms.

Estimated non-polar retention index (n-alkane scale):
Value: 3094 iu
Confidence interval (Low reliability): 174(50%) 752(95%) iu

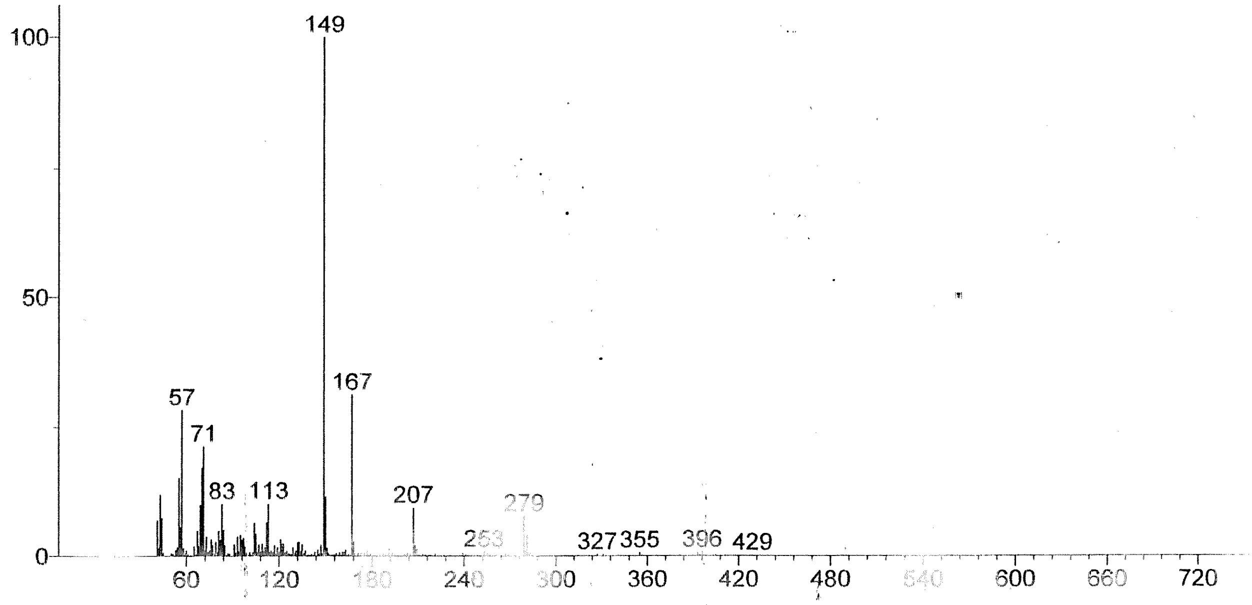

(Text File) +EI Scan (76.675 min) ALI-PIS-H1-210421.D

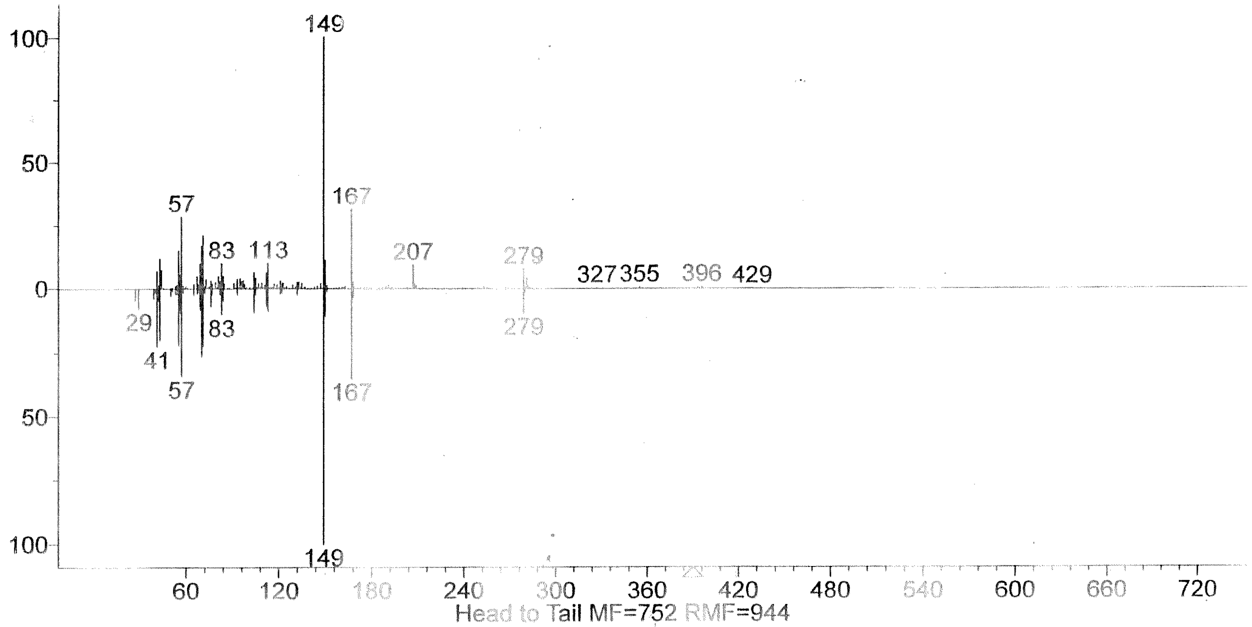

Head to Tail MF=752 RMF=944

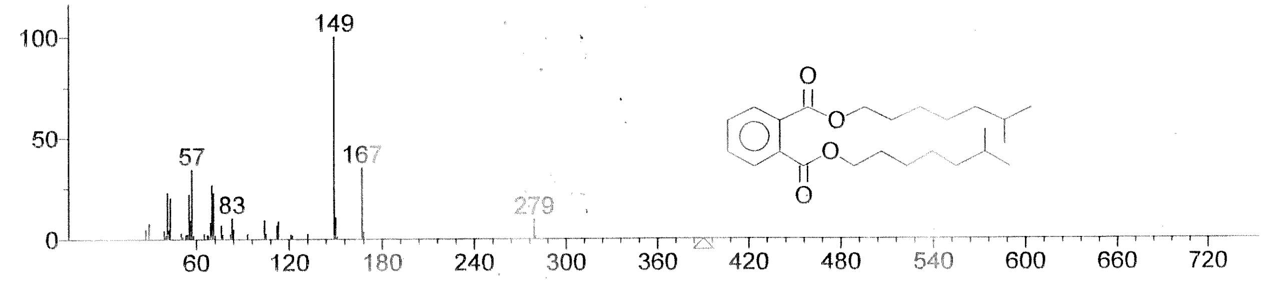

(replib) 1,2-Benzenedicarboxylic acid, diisooctyl ester

56

Name: 1,2-Benzenedicarboxylic acid, diisooctyl ester
Formula: $C_{24}H_{38}O_4$
MW: 390 CAS#: 27554-26-3 NIST#: 113206 ID#: 20061 DB: replib
Other DBs: Fine, TSCA, RTECS, EINECS, IRDB
Contributor: NIST Mass Spectrometry Data Center, 1990.
10 largest peaks:
149 999 | 167 350 | 57 341 | 70 264 | 41 225 | 71 224 | 55 218 | 43 200 | 150 107 | 83 100 |
Synonyms:
1.Diisooctyl phthalate
2.Hexaplas M/O
3.Isooctyl phthalate
4.Corflex 880
5.DIOP
6.Flexol plasticizer diop
7.Morflex 100
8.Palatinol D10
9.Phthalic acid, bis(6-methylheptyl) ester
10.Phthalic acid, diisooctyl ester
11.Witcizer 313
12.Bis(6-methylheptyl) phthalate #

Estimated non-polar retention index (n-alkane scale):
Value: 2704 iu
Confidence interval (Esters): 47(50%) 201(95%) iu

57

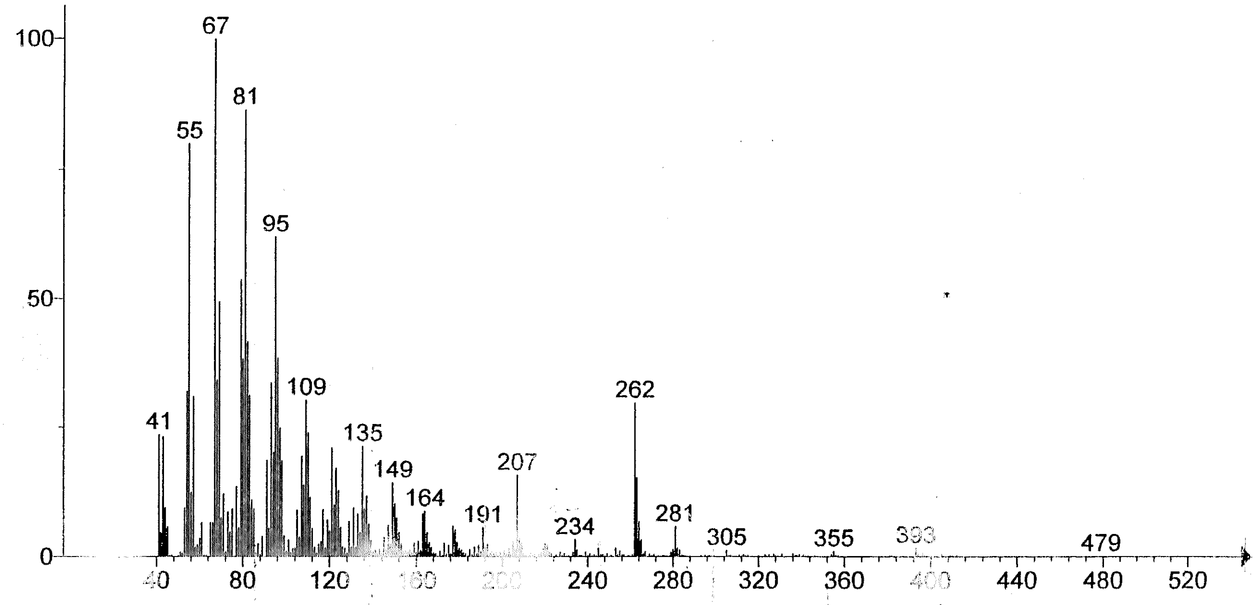

(Text File) +EI Scan (78.810 min) ALI-PIS-H1-210421.D

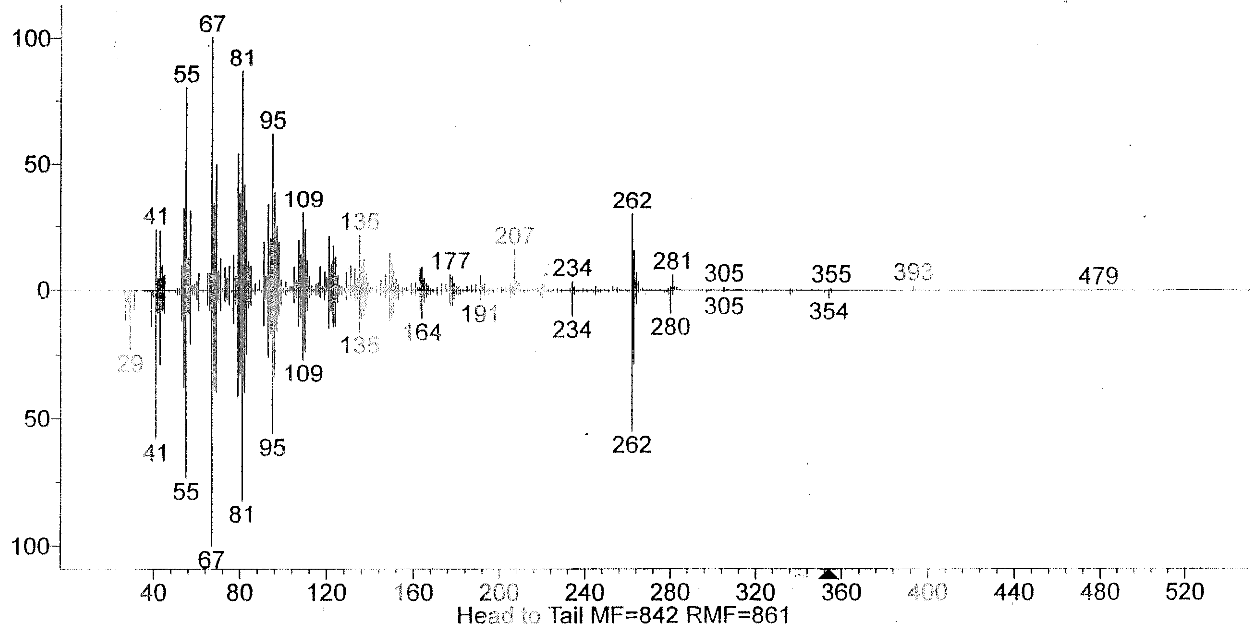

Head to Tail MF=842 RMF=861

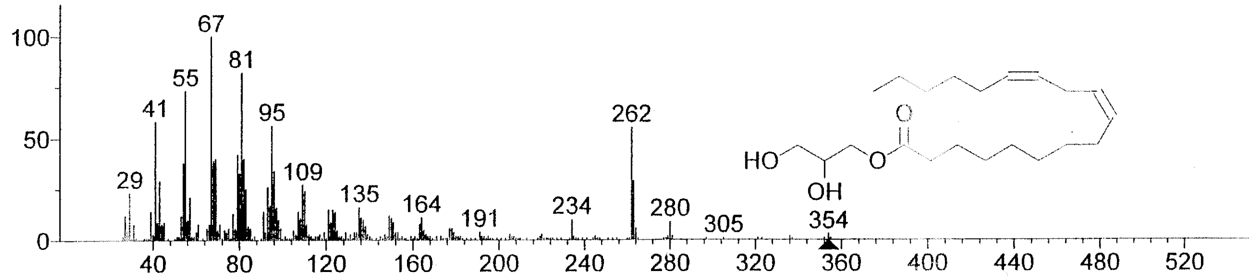

(mainlib) 9,12-Octadecadienoic acid (Z,Z)-, 2,3-dihydroxypropyl ester

Name: 9,12-Octadecadienoic acid (Z,Z)-, 2,3-dihydroxypropyl ester
Formula: $C_{21}H_{38}O_4$
MW: 354 CAS#: 2277-28-3 NIST#: 16012 ID#: 28839 DB: mainlib
Other DBs: EINECS
10 largest peaks:
67 999 | 81 820 | 55 730 | 41 580 | 95 560 | 262 550 | 79 420 | 69 400 | 82 400 | 68 390 |
Synonyms:
1.Linolein, 1-mono-
2.α-Glyceryl linoleate
3.Glycerol 1-monolinolate
4.Oleinate 288
5.1-Glyceryl linoleate
6.1-Monolinolein
7.2,3-Dihydroxypropyl (9Z,12Z)-9,12-octadecadienoate #

Estimated non-polar retention index (n-alkane scale):
Value: 2697 iu
Confidence interval (Diverse functional groups): 89(50%) 382(95%) iu

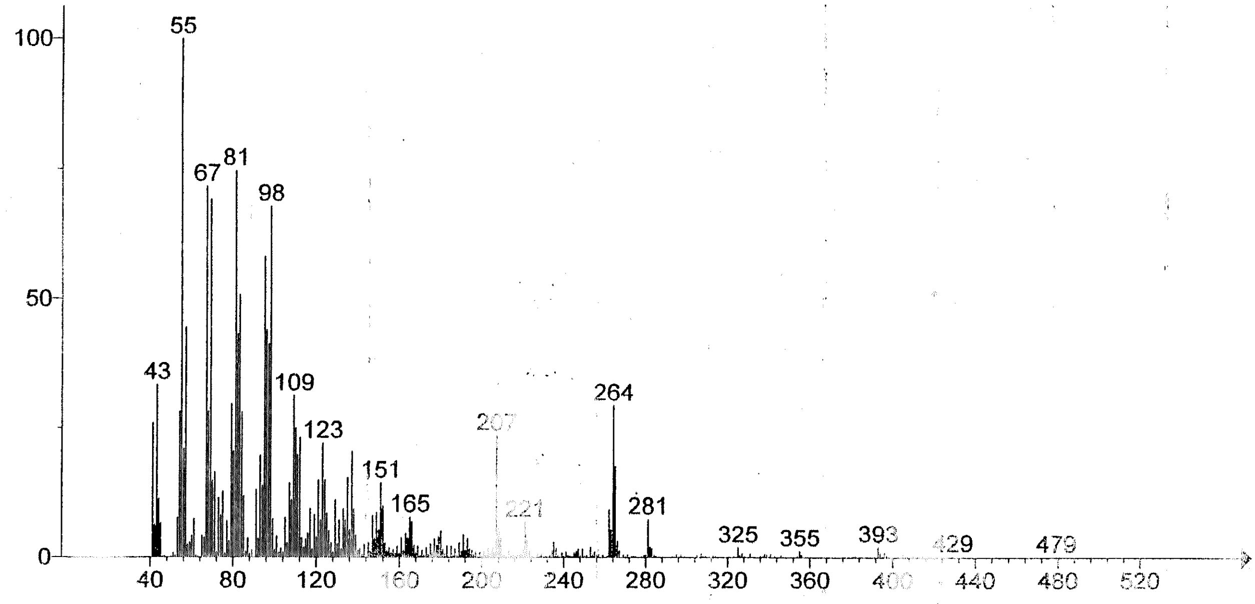

(Text File) +EI Scan (78.853 min) ALI-PIS-H1-210421.D

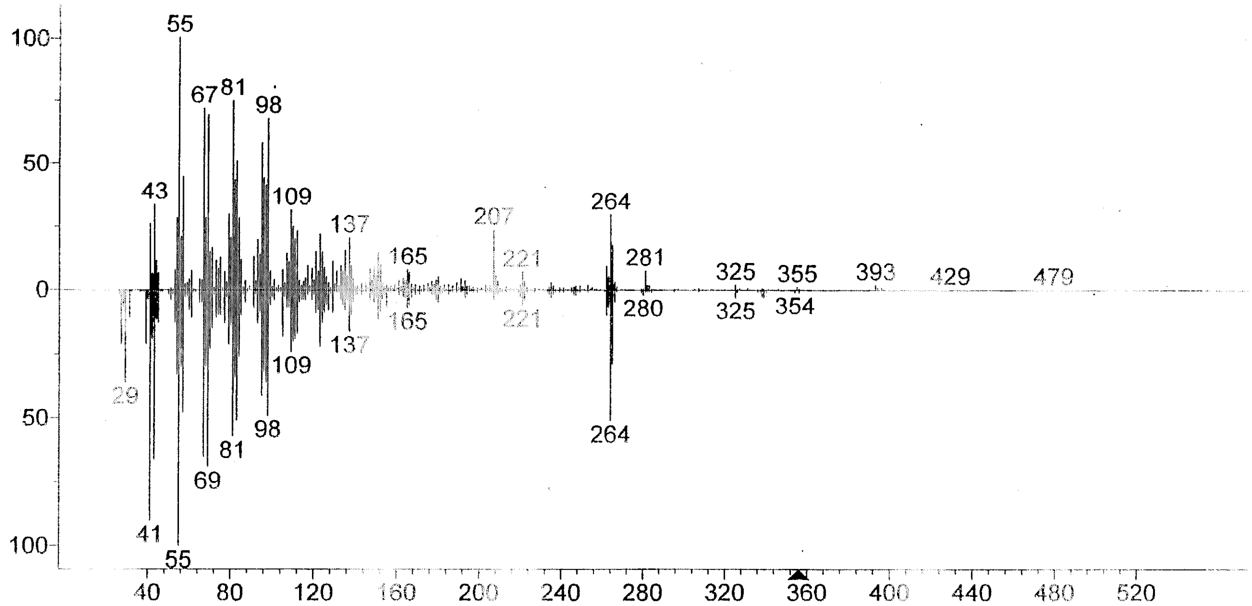

Head to Tail MF=850 RMF=871

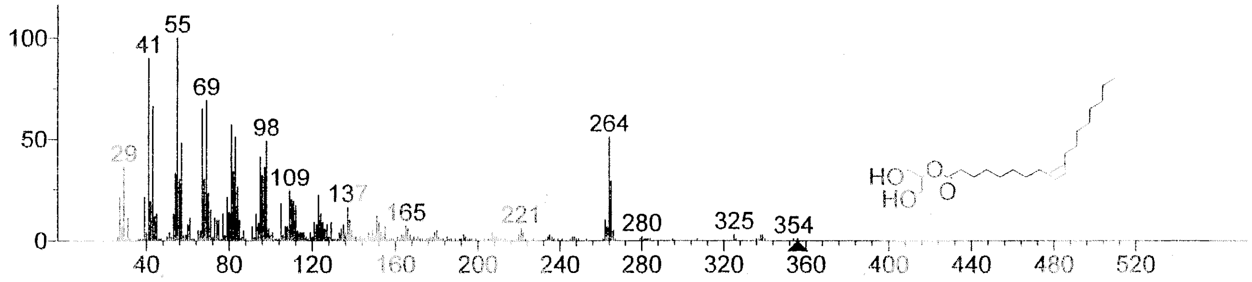

(mainlib) 9-Octadecenoic acid (Z)-, 2-hydroxy-1-(hydroxymethyl)ethyl ester

Name: 9-Octadecenoic acid (Z)-, 2-hydroxy-1-(hydroxymethyl)ethyl ester
Formula: $C_{21}H_{40}O_4$
MW: 356 CAS#: 3443-84-3 NIST#: 16061 ID#: 17482 DB: mainlib
Other DBs: None
10 largest peaks:
55 999 | 41 900 | 69 690 | 43 660 | 67 650 | 81 570 | 83 510 | 264 510 | 98 490 | 57 480 |
Synonyms:
1.Olein, 2-mono-
2.β-Monoolein
3.Glycerol 2-monooleate
4.2-Monoolein
5.2-Monooleoylglycerol
6.2-Oleoyl glycerol ether
7.2-Oleoylglycerol
8.2-Hydroxy-1-(hydroxymethyl)ethyl (9Z)-9-octadecenoate #

Estimated non-polar retention index (n-alkane scale):
Value: 2705 iu
Confidence interval (Diverse functional groups): 89(50%) 382(95%) iu

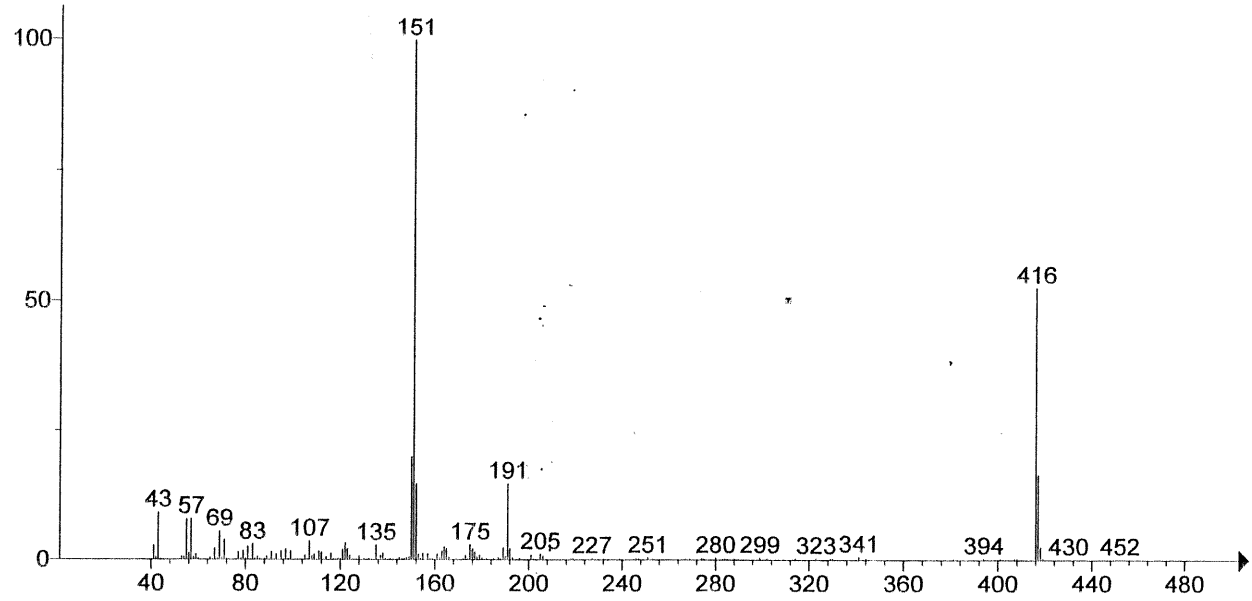

(Text File) +EI Scan (84.734 min) ALI-PIS-H1-210421.D Subtract

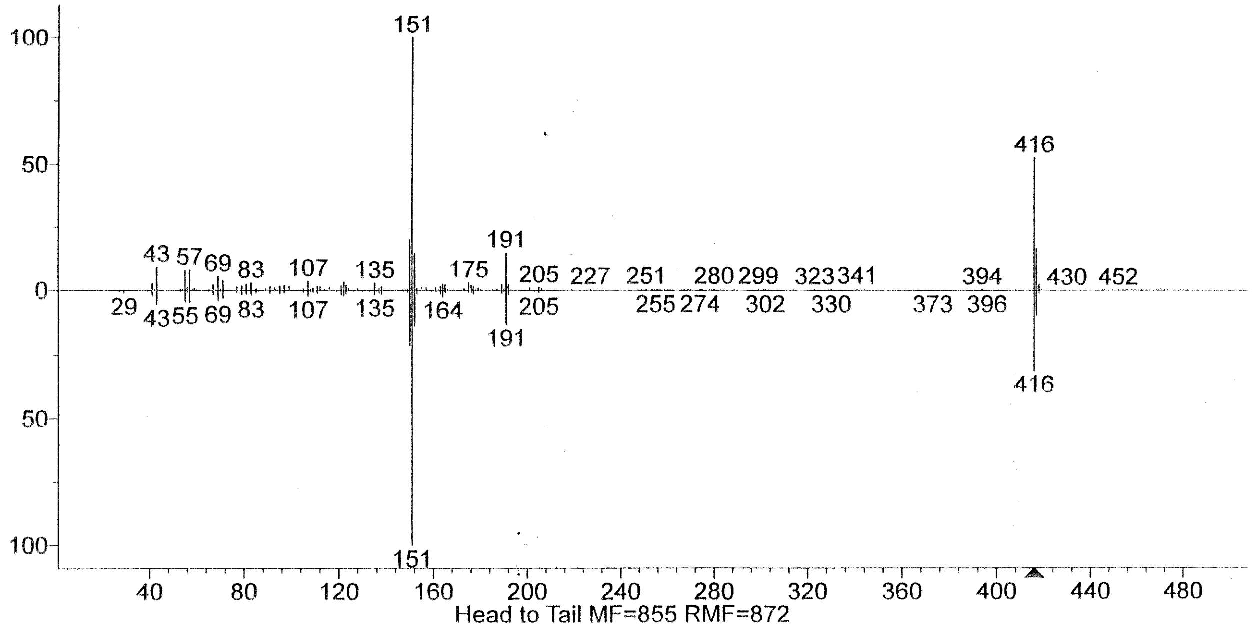

Head to Tail MF=855 RMF=872

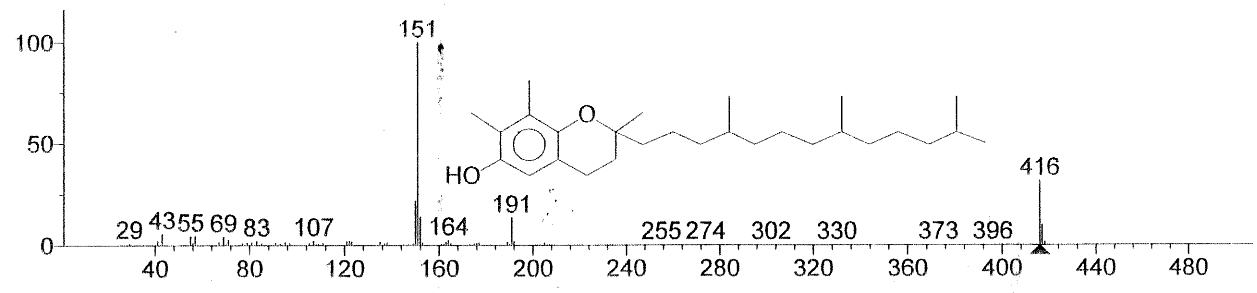

(replib) γ-Tocopherol

Name: γ-Tocopherol
Formula: $C_{28}H_{48}O_2$
MW: 416 CAS#: 7616-22-0 NIST#: 151381 ID#: 20323 DB: replib
Other DBs: TSCA, HODOC, EINECS
Contributor: Chemical Concepts
10 largest peaks:
151 999 | 416 312 | 150 216 | 152 137 | 191 132 | 417 98 | 43 55 | 55 44 | 57 44 | 69 40 |
Synonyms:
1.2H-1-Benzopyran-6-ol, 3,4-dihydro-2,7,8-trimethyl-2-(4,8,12-trimethyltridecyl)-
2.6-Chromanol, 2,7,8-trimethyl-2-(4,8,12-trimethyltridecyl)-
3.γ-Tokoferol
4.o-Xylotocopherol
5.7,8-Dimethyltocol
6.3,4-Dihydro-2,7,8-trimethyl-2-(4,8,12-trimethyltridecyl)-2H-1-benzopyran-6-ol
7.7,8-Dimethyltocolo-xylotocopherol
8.2,7,8-Trimethyl-2-(4,8,12-trimethyltridecyl)-6-chromanol #

Estimated non-polar retention index (n-alkane scale):
Value: 3036 iu
Confidence interval (Diverse functional groups): 89(50%) 382(95%) iu

Retention index.
1. Value: 3994 iu
Column Type: Capillary
Column Class: Standard non-polar
Active Phase: SE-30
Column
Length: 30 m
Carrier Gas: He
Column Diameter: 0.25 mm
Phase Thickness: 0.25 um
Data Type: Linear
RI
Program Type: Ramp
Start T: 60 C
End T: 280 C
Heat Rate: 16 K/min
Start Time: 3 min
End Time: 10
min
Source: Tundis, R.; Passalacqua, N.G.; Peruzzi, L.; Statti, G.A.; Bonesi, M.; Loizzo, M.R.; Conforti, F.; Cesca, G.; Menichini, F., Comparative chemical variability of the non-polar extracts from Senecio cineraria group (Asteraceae), Biochem. Syst. Ecol., 33, 2005, 1071-1076.
